# Impact of the 2008 economic crisis on the burden of hepatitis B and C diseases in Southern European countries

**DOI:** 10.1186/s12889-024-18912-0

**Published:** 2024-06-20

**Authors:** Claudia Palladino, Rebeca Ramis, Ifeanyi Jude Ezeonwumelu, Antonio Biondi, Giulia Carreras, Florian Fischer, Silvano Gallus, Davide Golinelli, Giuseppe Gorini, Shoaib Hassan, Zubair Kabir, Ai Koyanagi, Jeffrey V. Lazarus, Alexios-Fotios A. Mentis, Tuomo J. Meretoja, Ali H. Mokdad, Lorenzo Monasta, Francesk Mulita, Maarten J. Postma, Rafael Tabarés-Seisdedos, Arulmani Thiyagarajan, Nuno Taveira, Verónica Briz, GBD 2019 Southern Europe Hepatitis B & C Collaborators

**Affiliations:** 1 Research Institute for Medicines, Faculty of Pharmacy, Universidade de Lisboa, Avenida Professor Gama Pinto, Lisbon, 1649-003 Portugal; 2 Nacional Center for Epidemiology, Institute of Health Carlos III, Madrid, Spain; 3 Institute for Health Science Research Germans Trias I Pujol, Autonomous University of Barcelona, Badalona, Spain; 4 IrsiCaixa AIDS Research Institute, Badalona, Spain; 5 Department of General Surgery and Medical-Surgical Specialties, University of Catania, Catania, Italy; 6 Institute for Cancer Research, Prevention, and Clinical Network, Florence, Italy; 7 Institute of Public Health, Charité Universitätsmedizin Berlin, Charité Medical University Berlin), Berlin, Germany; 8 Department of Environmental Health Sciences, Mario Negri Institute for Pharmacological Research, Milan, Mario Italy; 9 Department of Biomedical and Neuromotor Sciences, University of Bologna, Bologna, Italy; 10 Oncological Network, Prevention and Research Institute, Institute for Cancer Research, Prevention, and Clinical Network, Florence, Italy; 11 Center for International Health (CIH), University of Bergen, Bergen, Norway; 12 Bergen Center for Ethics and Priority Setting (BCEPS), University of Bergen, Bergen, Norway; 13 School of Public Health, University College Cork, Cork, Ireland; 14 San Juan de Dios Sanitary Park, Barcelona, Spain; 15 Barcelona Institute for Global Health, Barcelona, Spain; 16 BGI-Shenzhen, Shenzhen, Guangdong China; 17 Breast Surgery Unit, Helsinki University Hospital, Helsinki, Finland; 18 University of Helsinki, Helsinki, Finland; 19 Institute for Health Metrics and Evaluation, University of Washington, Seattle, WA USA; 20 Department of Health Metrics Sciences, School of Medicine, University of Washington, Seattle, WA USA; 21 Clinical Epidemiology and Public Health Research Unit, Burlo Garofolo Institute for Maternal and Child Health, Trieste, Italy; 22 Department of Surgery, General University Hospital of Patras, Patras, Greece; 23 Faculty of Medicine, University of Thessaly, Larissa, Greece; 24 University Medical Center Groningen, University of Groningen, Groningen, Netherlands; 25 Center of Excellence in Higher Education for Pharmaceutical Care Innovation, Universitas Padjadjaran (Padjadjaran University), Bandung, Indonesia; 26 Department of Medicine, University of Valencia, Valencia, Spain; 27 Carlos III Health Institute, Biomedical Research Networking Center for Mental Health Network (CiberSAM), Madrid, Spain; 28 Clinical Epidemiology, Leibniz Institute for Prevention Research and Epidemiology, Bremen, Germany; 29 University Institute “Egas Moniz”, Monte da Caparica, Portugal; 30 Research Institute for Medicines, University of Lisbon, Lisbon, Portugal; 31 Laboratory of Viral Hepatitis, National Center of Microbiology, Institute of Health Carlos III, Carretera Majadahonda-Pozuelo Km 2.2, 28220 Majadahonda, Madrid, Spain

**Keywords:** Global burden of disease, Hepatitis B, Hepatitis C, Southern European countries, Cirrhosis, Chronic liver diseases, Liver cancer, Epidemiology, Economic crisis, Drugs

## Abstract

**Background:**

The economic crisis that began in 2008 has severely affected Southern (Greece, Italy, Portugal, Spain) Western European (SWE) countries of Western Europe (WE) and may have affected ongoing efforts to eliminate viral hepatitis. This study was conducted to investigate the impact of the economic crisis on the burden of HBV and HCV disease.

**Methods:**

Global Burden of Diseases 2019 data were used to analyse the rates of epidemiological metrics of HBV and HCV acute and chronic infections in SWE and WE. Time series modelling was performed to quantify the impact of healthcare expenditure on the time trend of HBV and HCV disease burden in 2000–2019.

**Results:**

Declining trends in incidence and prevalence rates of acute HBV (aHBV) and chronic HBV were observed in SWE and WE, with the pace of decline being slower in the post-austerity period (2010–2019) and mortality due to HBV stabilised in SWE. Acute HCV (aHCV) metrics and chronic HCV incidence and mortality showed a stable trend in SWE and WE, whereas the prevalence of chronic HCV showed an oscillating trend, decreasing in WE in 2010–2019 (*p* < 0.001). Liver cancer due to both hepatitis infections showed a stagnant burden over time. An inverse association was observed between health expenditure and metrics of both acute and chronic HBV and HCV.

**Conclusions:**

Epidemiological metrics for HBV and HCV showed a slower pace of decline in the post-austerity period with better improvement for HBV, a stabilisation of mortality and a stagnant burden for liver cancer due to both hepatitis infections. The economic crisis of 2008 had a negative impact on the burden of hepatitis B and C. Elimination of HBV and HCV by 2030 will be a major challenge in the SWE countries.

**Supplementary Information:**

The online version contains supplementary material available at 10.1186/s12889-024-18912-0.

## Background

Viral hepatitis has been recognised as a health and development priority with the adoption of two United Nations resolutions in 2010 and 2014 [1]. In response to these resolutions, WHO launched the first global health sector strategy for viral hepatitis in 2016 [2]. The strategy aims to articulate the synergistic actions needed to coordinate national responses to eliminate hepatitis and support the 2030 Agenda for Sustainable Development [3, 4]. Specifically, WHO has set two targets for 2030: to reduce the incidence of chronic viral infection by 90% and the number of deaths from viral hepatitis by 65%, and to ensure equitable access to prevention, testing, care and treatment services for all [2]. Viral hepatitis is caused by five hepatitis viruses (A to E) and is responsible for an estimated 1.3 million deaths per year, mainly from chronic liver disease and liver cancer due to hepatitis B virus (HBV) and hepatitis C virus (HCV) [5].

Despite the availability of a preventive HBV vaccine since 1982, recent data on global HBV prevalence estimate that there are 316 million chronic hepatitis B surface antigen (HBsAg) carriers (4.1% of the world’s population), including 6 million children under the age of five, with peak prevalence observed in Africa (6.5%) and the Western Pacific region (7.1%) [6]. It is estimated that 15–40% of people living with HBV will develop serious sequelae of infection, mainly affecting the liver [7]. HCV has a lower prevalence compared to HBV, with 58 million chronic infections in 2019 (0.8% of the world population), with peak prevalence observed in the Eastern Mediterranean (1.6%) and European regions (1.3%) [5, 8]. Up to 85% of people with acute HCV will develop chronic infection [9]. Estimated incidence in 2019 was similar for both viruses (1.5 million new infections each) [5]. Recent data from the Global Burden of Diseases, Injuries, and Risk Factors Study (GBD) from 2010 to 2019 showed declining trends in the incidence of acute HBV and HCV, and HBV cirrhosis worldwide until 2015, as well as overall declines in disability-adjusted life years (DALYs) and mortality for HBV and HCV cirrhosis, although analysis of macro areas showed disparities in disease epidemiology [10, 11].

The Great Recession that hit Europe in 2008–2009 particularly affected Greece, Italy, Portugal and Spain, weakening their economic systems and worsening their deficits [12]. As a result, a sovereign debt crisis was triggered, forcing their governments to implement austerity measures and increase tax revenues from 2010 onwards [13, 14]. In these countries, health systems have been severely affected by the crisis, with significant reductions in public health expenditure between 2009 and 2017 (between -7% and -4%) [14]. A number of examples of the impact of the crisis can be highlighted. In Italy, the number of hospital beds fell from 4.6 to 3.6 per 1,000 inhabitants between 2000 and 2010, lower than the European average from the same period (5.5), and continued to fall, reaching 3.2 in 2015, after which it stabilised at 3.1 [15]. In Portugal, self-reported access to healthcare deteriorated [16] and excess mortality associated with influenza and cold weather was reported in early 2012, probably related to low home heating capacity [17]. The health status of the population worsened significantly, especially among vulnerable groups (e.g. increased rates of stillbirth [18], anxiety and alcohol-related disorders [17, 19], heart attacks [20], suicide [21–23] and some communicable diseases, among others). For example, an HIV outbreak among people who inject drugs (PWID) occurred in Greece in 2011–2012, mainly due to a reduction in preventive measures [24, 25], while delays in the approval of innovative drugs for HCV have been denounced in Portugal [26]. In this context, we have performed a time series analysis of the burden of HBV- and HCV-associated diseases and investigated whether austerity measures have affected the burden of these diseases in Greece, Italy, Portugal and Spain.

## Methods

### Overview and metrics

GBD 2019 provides a standardised approach to estimating annual updates of epidemiological trends in the global burden of 369 diseases and injuries and 87 risk factors from 1990 to 2019 [27, 28]. Raw input data from 2000 to 2019 were obtained from a comprehensive set of sources, including population censuses, disease notifications and registries, household surveys, civil registration and vital statistics, health service utilisation, and others. The data are made available through the Global Health Data Exchange, a catalogue of global health and demographic data. Six GBD codes were used to extract estimates: acute HBV (A.5.8.2) and acute HCV (A.5.8.3) causes; cirrhosis and other chronic liver diseases due to hepatitis B (B.4.1.1) and due to hepatitis C (B.4.1.2), collectively referred to as cirrhosis in this paper; liver cancer due to hepatitis B (B.1.7.1) and due to hepatitis C (B.1.7.2). Cases of cirrhosis and other chronic liver diseases due to alcohol consumption (code B.4.1.3) were excluded. The burden of these diseases was assessed using the following epidemiological metrics which were the primary outcomes of analysis: prevalence, incidence, mortality, years lived with disability (YLDs), years of life lost to premature mortality (YLLs), and disability-adjusted life years (DALYs) for HBV and HCV diseases.

The sites studied were four Southern (Greece, Italy, Portugal, Spain) Western European (SWE) countries. For comparison, we also extracted metrics for the GBD Western Europe (WE) region: Andorra, Austria, Belgium, Cyprus, Denmark, Finland, France, Germany, Greece, Iceland, Ireland, Israel, Italy, Luxembourg, Malta, the Netherlands, Norway, Portugal and Spain (Appendix). The four SWE countries analysed were also included in the trends for Western Europe because GBD estimates are available for the whole of the Western Europe region. All metrics were disaggregated by sex: female, male or both, and by 5 age groups. The upper and lower limits of the 95% confidence interval (95% CI) were also extracted for each parameter. These were derived from 1000 draws of the distribution of each estimation step by age, sex and location for each year included in the analysis and represent the ordinal 25th and 975th draws of each quantity [27]. In order to assess the impact of austerity measures, we used current health expenditure as defined by the World Bank as a percentage of gross domestic product (%GDP) for each country studied [29]. This variable was not included in the analysis for WE due to the lack of %GDP for the WE region as a whole. HIV burden was also included in the analysis. HIV incidence and prevalence data were extracted from the Global Health Data Exchange, using GBD code B.1.7.2, to control for potential confounding by the relationship between HIV and viral hepatitis, due to the prevalence of co-infection and the impact of HIV on the clinical course of hepatitis [30].

### Statistical analysis

To quantify the role of austerity, the impact of current health expenditure on the time trend of HBV and HCV disease burden, interrupted time series models were performed. The primary outcomes were considered as the dependent variables (prevalence, incidence, mortality, YLDs, YLLs, and DALYs) and the independent variables or covariates were %GDP, sex, age group, period trend, post-austerity period trend and HIV burden. To account for the delayed effect of austerity, %GDP was introduced with a five-year lag compared to the epidemiological metrics. For HIV burden, HIV incidence was used in the models for acute HBV and HCV, whereas HIV prevalence was used in the models for cirrhosis and cancer due to HBV and HCV. Once the model was defined, multiple linear regressions were fitted to estimate the effect, 95% CI and p values for each independent variable for outcome, causes and countries [27, 28]. The period from 2000 to 2010 represents the pre-austerity period, while the years from 2010 to 2019 include both the austerity and post-austerity periods [12]. Detailed methods for the entire study are available in the GBD 2019 summary publications [27, 28], each of which follows the recommendations of the Guidelines for Accurate and Transparent Health Estimates Reporting (GATHER) to ensure that data and methods are adequately documented [31]. Table 1 provides brief descriptions of the methods used to derive each of the GBD metrics included in this analysis. All data were retrieved from the Global Burden of Disease Study 2019 between September and December 2020 [32]. Analyses and plots were performed using R version R-4.0.3 [33].

**Table 1:** Metric definitions—definition of the metrics included in the present analysis (Global Burden of Disease Study 2019)

Measure	Number	Percent	Rate
Prevalence	Number of cases in the population	Proportion of cases of a particular disease relative to cases from all diseases	Cases per 100,000 individuals
Incidence	Number of new cases in the population	Proportion of cases of a particular disease relative to cases from all diseases	Cases per 100,000 individuals
Deaths	Number of deaths in the population	Proportion of deaths for a particular disease relative to deaths from all disease	Deaths per 100,000 individuals
Disability adjusted life years (DALYs)	Number of DALYs in the population	Proportion of DALYs for a particular disease relative to DALYs for all diseases	DALYs per 100,000 individuals
Years lived with disability (YLDs)	Number of YLDs in the population	Proportion of YLDs for a particular disease relative to YLDs for all diseases	YLDs per 100,000 individuals
Years of life lost (YLLs)	Number of YLLs in the population	Proportion of YLLs for a particular disease relative to YLLs for all diseases	YLLs per 100,000 individuals

### Ethics approval and consent to participate

This study does not require ethical approval or informed consent. It does not contain data on individuals, but only epidemiological estimates.

### Consent for publication

Not applicable.

### Role of funding source

Funding was provided by the Bill & Melinda Gates Foundation. The funder of this study had no role in study design, data collection, data analysis, data interpretation, or writing of the report.

## Results

### Hepatitis B Virus

#### Acute hepatitis B virus infection

**Fig. 1:**
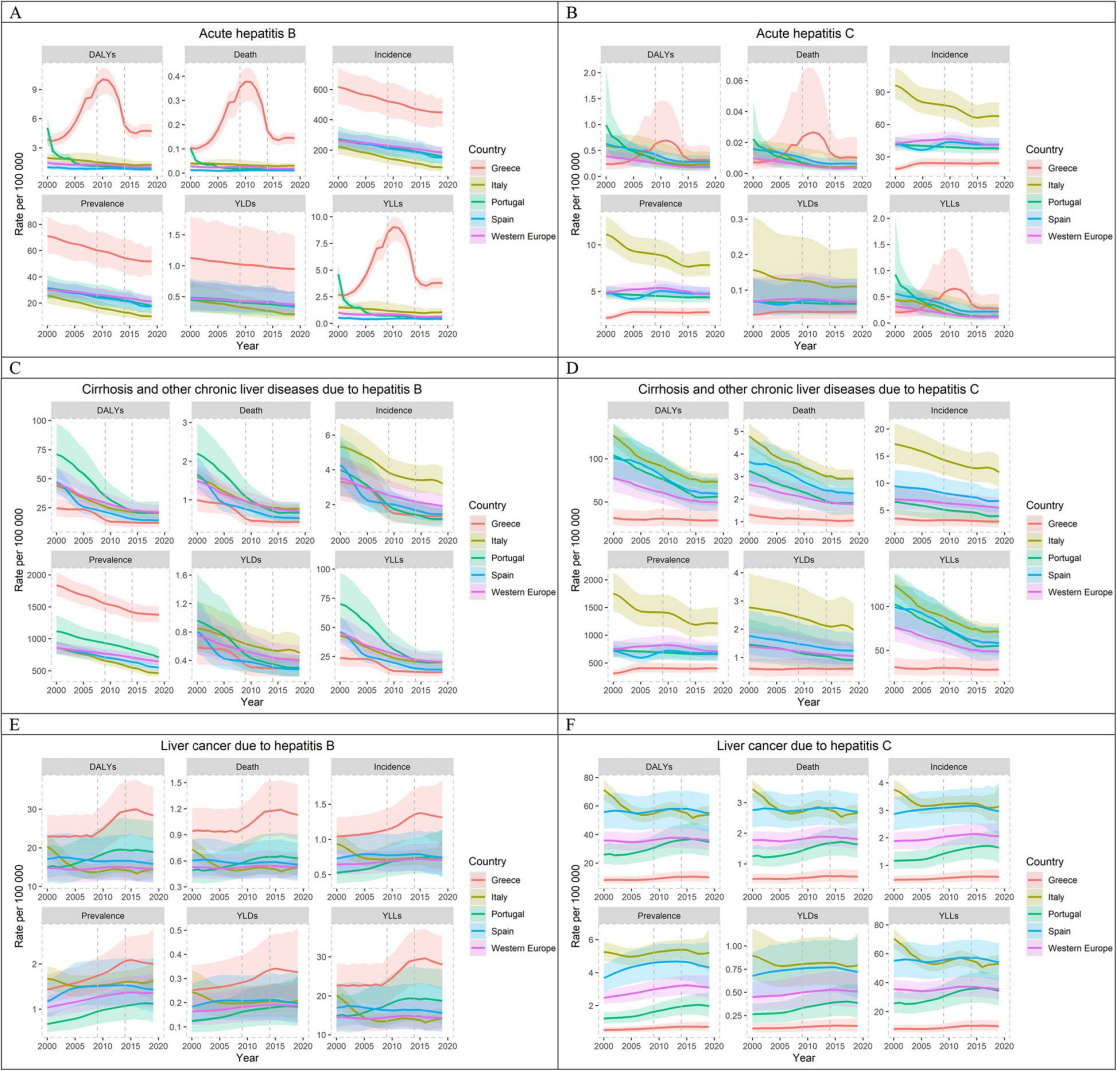
Trends of acute (**A**, **B**) and chronic (**C**-**F**) hepatitis B and hepatitis C age-standardized rates of prevalence, incidence, mortality, years lived with disability (YLDs), years of life lost (YLLs) and disability-adjusted life years (DALYs) per 100 000 population in Greece, Italy, Portugal, Spain and Western Europe from 2000 to 2019 (Global Burden of Disease Study 2019). The shadows around the lines represent the 95% confidence interval of the estimates

Overall, a decreasing trend in incidence and prevalence rates of acute hepatitis B virus (aHBV) in WE was observed during the study period (Fig. 1A, Table 2). In 2019, aHBV incidence and prevalence rates in Greece were 448.9 (95% CI: 354.3 – 555.2) and 51.7 (40.8; 64.0), respectively, three times higher than in Portugal and Spain and five times higher than in Italy (Appendix). Regarding aHBV incidence, a significant downward trend was observed in Greece from 2000 to 2019 [-23.17 cases per 100,000 for each consecutive year (-40.41 – -5.94); *p* = 0.01], despite the upward trend during the post-austerity period [24.00 (4.03 – 43.97); *p* = 0.02]. The downward trend was also observed in the other countries analysed, although it was not statistically significant. Incidence rates were higher in males in WE, Greece, Italy, Portugal, reaching up to 40.11 (9.77 – 70.46) cases per 100,000 for each case in females (*p* < 0.001) (Table 4). Each age group showed significantly lower incidence rates compared with the previous age group in WE [-6.01 (-10.05 – -1.96); *p* < 0.001], while an opposite trend was observed in SWE [from 14.70 (7.88 – 21.53) cases per 100,000 in Portugal to 86.92 cases (76.32 – 97.52) in Greece (*p* < 0.001)]. The incidence rate increased significantly from 8.10 (7.22 – 8.99) to 19.16 (17.93 – 20.40) cases per 100,000 for each HIV case reported in Portugal, Italy, Spain and WE, and up to 159.30 (149.06 – 169.53) in Greece. aHBV prevalence showed a similar trend, although at lower rates. Regarding mortality, a stable trend was observed in WE and SWE during 2000 – 2019, despite the significant upward trend observed in Greece [0.19 (0.08 – 0.29) cases per 100,000 for each consecutive year; *p* < 0.001], which was compensated during the post-austerity period [-0.26 (-0.38 – -0.13); *p* < 0.001] (Table 4). DALYs and YLLs showed a similar trend in Greece (*p* < 0.001). Notably, the rates have stabilised since 2015 (Fig. 1A, Supplementary Appendix). No changes were observed in the other countries. Males and older age groups were more affected in WE and SWE (*p* < 0.001) (Table 4, Appendix).

**Table 2:** Mortality estimates of acute and chronic hepatitis B for Greece, Italy, Portugal, Spain and Western Europe in 2000, 2010 and 2019 (Global Burden of Disease Study 2019). 95% CI: 95% confidence interval

**Acute hepatitis B**									
	**Number of deaths (all ages)**	**95% CI (lower)**	**95% CI (upper)**	**Rate of deaths (age-standardized, per 100,000)**	**95% CI (lower)**	**95% CI (upper)**	**Rate of deaths (all ages, per 100,000)**	**95% CI (lower)**	**95% CI (upper)**
	All Ages			Age-standardized			All Ages		
**Greece**									
2000	17	14	20	0.10	0.08	0.12	0.16	0.12	0.18
2010	81	61	96	0.38	0.30	0.43	0.73	0.55	0.86
2019	33	26	39	0.14	0.12	0.17	0.32	0.25	0.38
**Italy**									
2000	33	22	39	0.04	0.03	0.05	0.06	0.04	0.07
2010	33	20	53	0.03	0.02	0.05	0.05	0.03	0.09
2019	34	23	44	0.03	0.02	0.04	0.06	0.04	0.07
**Portugal**									
2000	13	9	16	0.10	0.07	0.12	0.12	0.09	0.15
2010	3	2	4	0.02	0.02	0.02	0.03	0.02	0.03
2019	3	2	3	0.02	0.01	0.02	0.02	0.02	0.03
**Spain**									
2000	6	5	9	0.01	0.01	0.02	0.01	0.01	0.02
2010	7	5	9	0.01	0.01	0.01	0.01	0.01	0.02
2019	7	5	9	0.01	0.01	0.01	0.02	0.01	0.02
**Western Europe**									
2000	156	119	176	0.03	0.02	0.03	0.04	0.03	0.04
2010	183	147	212	0.03	0.02	0.03	0.04	0.03	0.05
2019	138	114	161	0.02	0.02	0.02	0.03	0.03	0.04
**Cirrhosis and other chronic liver diseases due to hepatitis B**									
	**Number of deaths (all ages)**	**95% CI (lower)**	**95% CI (upper)**	**Rate of deaths (age-standardized, per 100,000)**	**95% CI (lower)**	**95% CI (upper)**	**Rate of deaths (all ages, per 100,000)**	**95% CI (lower)**	**95% CI (upper)**
	All Ages			Age-standardized			All Ages		
**Greece**									
2000	169	117	235	1.00	0.70	1.35	1.52	1.05	2.12
2010	88	60	124	0.46	0.32	0.64	0.79	0.54	1.12
2019	91	63	127	0.43	0.29	0.59	0.88	0.61	1.23
**Italy**									
2000	1 549	1 370	1 744	1.59	1.43	1.77	2.73	2.42	3.08
2010	1 067	921	1 233	0.91	0.79	1.03	1.77	1.53	2.04
2019	1 025	871	1 197	0.77	0.67	0.89	1.70	1.44	1.99
**Portugal**									
2000	323	227	442	2.20	1.56	2.97	3.06	2.15	4.19
2010	168	116	232	0.98	0.67	1.36	1.56	1.07	2.15
2019	131	90	183	0.67	0.46	0.94	1.23	0.85	1.72
**Spain**									
2000	1 033	801	1 336	1.66	1.30	2.12	2.53	1.96	3.27
2010	560	421	728	0.72	0.55	0.92	1.19	0.90	1.55
2019	480	353	644	0.53	0.40	0.69	1.04	0.77	1.40
**Western Europe**									
2000	8 831	6 819	11 236	1.49	1.15	1.90	2.21	1.71	2.82
2010	6 455	4 905	8 288	0.93	0.71	1.19	1.53	1.16	1.96
2019	5 620	4 288	7 253	0.71	0.54	0.92	1.29	0.98	1.66
**Liver cancer due to hepatitis B**									
	**Number of deaths (all ages)**	**95% CI (lower)**	**95% CI (upper)**	**Rate of deaths (age-standardized, per 100,000)**	**95% CI (lower)**	**95% CI (upper)**	**Rate of deaths (all ages, per 100,000)**	**95% CI (lower)**	**95% CI (upper)**
	All Ages			Age-standardized			All Ages		
**Greece**									
2000	166	124	216	0.94	0.72	1.20	1.50	1.12	1.95
2010	204	151	267	1.00	0.76	1.29	1.85	1.37	2.41
2019	249	182	333	1.13	0.85	1.48	2.41	1.76	3.22
**Italy**									
2000	708	591	838	0.73	0.62	0.86	1.25	1.04	1.48
2010	568	482	675	0.51	0.43	0.59	0.94	0.80	1.12
2019	654	536	799	0.52	0.43	0.64	1.08	0.89	1.32
**Portugal**									
2000	76	51	108	0.49	0.34	0.68	0.72	0.49	1.02
2010	106	72	150	0.60	0.42	0.84	0.98	0.67	1.39
2019	125	84	183	0.63	0.43	0.91	1.17	0.79	1.72
**Spain**									
2000	376	255	544	0.60	0.42	0.85	0.92	0.62	1.33
2010	426	292	609	0.57	0.39	0.80	0.91	0.62	1.30
2019	467	310	685	0.56	0.37	0.81	1.02	0.67	1.49
**Western Europe**									
2000	3 274	2 455	4 319	0.53	0.40	0.69	0.82	0.62	1.08
2010	3 818	2 840	5 087	0.53	0.40	0.70	0.90	0.67	1.20
2019	4 299	3 127	5 823	0.53	0.39	0.71	0.99	0.72	1.33

#### Chronic hepatitis B virus infection

Both WE and SWE showed a decreasing trend between 2000 and 2019 in the six investigated metrics of cirrhosis and other chronic liver diseases (CCLD), which was significant for WE and Greece (Fig. 1C, Table 2, Appendix). Greece showed a decrease in incidence [-0.29 (-0.43 – -0.15); *p* < 0.001] and prevalence [-55.81 (-95.15 – -16.47); *p* = 0.006] cases per 100,000 inhabitants each consecutive year, followed by a stabilisation during the post-austerity period (*p* < 0.001) (Table 4). Italy had the highest incidence rate of CCLD throughout the study period [3.20 cases per 100,000 persons (2.42 – 4.15) in 2019] (Appendix). Incidence rates were lower in males in Greece, Portugal and Spain, reaching up to -0.44 (-0.78 – -0.10) cases per 100,000 for each case in females (*p* < 0.01), whereas prevalence rates were much higher in males in WE and SWE countries, reaching up to 223.23 (150.31 – 296.16) cases per 100,000 for each case in females (*p* < 0.001) in Greece, which was two times higher than in Portugal and four times higher than in Italy and Spain. In addition, age group was negatively associated with CCLD incidence in Greece [-0.12 (-0.21 – -0.03); *p* = 0.01] and Portugal [-0.20 (-0.28 – -0.11); *p* < 0.001]. The incidence of CCLD due to HBV was weakly positively associated with HIV prevalence. For CCLD prevalence, age group showed a positive effect, highlighting Greece with 596.48 cases per 100,000 (571.06 – 621.89; *p* < 0.001), twice more than the other SWE countries and 3.5 times more than WE. CCLD prevalence was positively associated with HIV prevalence in WE and SWE, ranging from 1.24 (1.14 – 1.34) in Portugal to 17.86 (16.23 – 19.50) in Greece (*p* < 0.001).

Regarding CCLD mortality, a decreasing trend was observed from 2000 to 2019 in WE and SWE, reaching statistical significance in WE [-0.11 (-0.18 – -0.05) cases per 100,000 for each consecutive year; *p* < 0.001] and Greece [-0.20 (-0.35 – -0.05; *p* = 0.01], despite a slight increase during the post-austerity period, which was significant only in Greece [0.20 (0.03 – 0.38); *p* = 0.02]. YLLs showed a similar trend. Males had significantly higher mortality rates than females in all countries, reaching the highest rates in Italy [2.04 (1.59 – 2.49) cases per 100,000 for each female case (*p* < 0.001)] and Portugal [2.08 (0.23 – 9.23); *p* < 0.001], similar to YLLs. Older age groups showed higher mortality and YLLs rates in WE and SWE, up to 1.86 [(1.73 – 2.00; *p* < 0.001] and 26.66 [(23.07; 30.25); *p* < 0.001], respectively, in WE (Table 4).

The prevalence rates of liver cancer (LC) due to HBV were at least 290–690-fold lower than CCLD across all sites in 2019 (Appendix). This is comparable to WE, where LC prevalence was 360-fold lower in 2019. LC burden remained stable for the six metrics evaluated over the period 2000 – 2019 (Fig. 1E), while males had higher rates compared to females in WE and SWE, particularly in Greece, where mortality was more than twofold higher compared to other SWE countries [4.02 (3.33 – 4.72); *p* < 0.001]. When stratified by age, a significant increase in rates was associated with older age groups for the six metrics, which were always about 2.5 times higher in Greece [mortality: 2.78 (2.50 – 3.07); *p* < 0.001] (Appendix, Table 4). A negligible effect of HIV on the LC metrics was observed (Table 4).

### Hepatitis C virus

#### Acute hepatitis C virus infection

The incidence and prevalence rates of acute HCV (aHCV) showed a generally stable trend in WE and SWE. Italy had the highest prevalence and incidence rates in both sexes in 2000, and estimates were still 1.7 times higher than in WE in 2019 [7.81 (6.68 – 9.27) vs 4.72 (4.13 – 5.45)] (Fig. 1B; Appendix).

Males had significantly higher incidence and prevalence rates compared to females in WE [8.17 (6.85 – 9.49) and 0.94 (0.79 – 1.10) respectively; *p* < 0. 001], Portugal [10.58 (5.25 – 15.91) and 1.22 (0.61 – 1.84) respectively; *p* < 0.001] and Spain [11.61 (5.21 – 18.02) and 1.34 (0.60; 2.08) respectively; *p* < 0.001]. Conversely, in Greece, the incidence and prevalence rates were comparable between sexes although slightly higher in females than in males [-1.65 (-5.94; 2.65) and -0.19 (-0.69; 0.31) respectively; *p* = 0.45]. These metrics reached statistical significance for age, with estimates increasing in older classes (*p* < 0.001). Stable trends in mortality, DALYs and YLLs due to aHCV in WE and SWE were observed from 2000 to 2019 (Table 3, Table 4, Appendix). Although statistically significant, there were few differences between sex and age groups (*p* < 0.001). A negligible effect of HIV on aHCV metrics was observed, with the exception of a negative association between HIV and aHCV incidence in WE and SWE (Table 4).

**Table 3:** Mortality estimates of acute and chronic hepatitis C for Greece, Italy, Portugal, Spain and Western Europe in 2000, 2010 and 2019 (Global Burden of Disease Study 2019). 95% CI: 95% confidence interval

**Acute hepatitis C**									
	**Number of deaths (all ages)**	**95% CI (lower)**	**95% CI (upper)**	**Rate of deaths (age-standardized, per 100,000)**	**95% CI (lower)**	**95% CI (upper)**	**Rate of deaths (all ages, per 100,000)**	**95% CI (lower)**	**95% CI (upper)**
	All Ages			Age-standardized			All Ages		
**Greece**									
2000	1	0	3	0.01	0.00	0.02	0.01	0.00	0.03
2010	6	2	17	0.03	0.01	0.07	0.05	0.02	0.15
2019	2	1	6	0.01	0.01	0.02	0.02	0.01	0.06
**Italy**									
2000	15	6	22	0.02	0.01	0.02	0.03	0.01	0.04
2010	7	4	18	0.01	0.00	0.02	0.01	0.01	0.03
2019	6	4	14	0.00	0.00	0.01	0.01	0.01	0.02
**Portugal**									
2000	3	2	6	0.02	0.01	0.05	0.03	0.02	0.06
2010	1	0	1	0.01	0.00	0.01	0.01	0.00	0.01
2019	1	0	1	0.00	0.00	0.00	0.01	0.00	0.01
**Spain**									
2000	9	6	11	0.02	0.01	0.02	0.02	0.01	0.03
2010	6	4	8	0.01	0.01	0.01	0.01	0.01	0.02
2019	5	3	7	0.01	0.00	0.01	0.01	0.01	0.02
**Western Europe**									
2000	58	31	81	0.01	0.01	0.01	0.01	0.01	0.02
2010	35	25	66	0.01	0.00	0.01	0.01	0.01	0.02
2019	26	18	51	0.00	0.00	0.01	0.01	0.00	0.01
**Cirrhosis and other chronic liver diseases due to hepatitis C**									
	**Number of deaths (all ages)**	**95% CI (lower)**	**95% CI (upper)**	**Rate of deaths (age-standardized, per 100,000)**	**95% CI (lower)**	**95% CI (upper)**	**Rate of deaths (all ages, per 100,000)**	**95% CI (lower)**	**95% CI (upper)**
	All Ages			Age-standardized			All Ages		
**Greece**									
2000	225	159	308	1.32	0.95	1.78	2.03	1.43	2.78
2010	213	152	295	1.11	0.80	1.49	1.93	1.37	2.66
2019	229	161	310	1.06	0.77	1.44	2.22	1.56	3.00
**Italy**									
2000	4 730	4 194	5 313	4.79	4.31	5.35	8.35	7.40	9.38
2010	4 092	3 510	4 700	3.41	2.98	3.88	6.78	5.82	7.79
2019	4 012	3 442	4 632	2.91	2.53	3.31	6.65	5.71	7.68
**Portugal**									
2000	479	346	653	3.25	2.38	4.36	4.55	3.28	6.19
2010	399	291	545	2.31	1.69	3.13	3.70	2.69	5.05
2019	364	263	495	1.83	1.32	2.53	3.41	2.47	4.64
**Spain**									
2000	2 289	1 727	2 970	3.66	2.82	4.72	5.61	4.23	7.28
2010	2 151	1 620	2 788	2.75	2.10	3.56	4.58	3.45	5.93
2019	2 077	1 548	2 699	2.25	1.73	2.90	4.51	3.36	5.87
**Western Europe**									
2000	16 101	12 992	20 019	2.65	2.15	3.30	4.03	3.26	5.02
2010	14 796	11 709	18 583	2.06	1.65	2.60	3.50	2.77	4.40
2019	14 719	11 480	18 398	1.79	1.41	2.25	3.37	2.63	4.22
**Liver cancer due to hepatitis C**									
	**Number of deaths (all ages)**	**95% CI (lower)**	**95% CI (upper)**	**Rate of deaths (age-standardized, per 100,000)**	**95% CI (lower)**	**95% CI (upper)**	**Rate of deaths (all ages, per 100,000)**	**95% CI (lower)**	**95% CI (upper)**
	All Ages			Age-standardized			All Ages		
**Greece**									
2000	91	64	122	0.51	0.36	0.66	0.82	0.58	1.10
2010	129	89	176	0.55	0.39	0.74	1.17	0.80	1.59
2019	166	115	227	0.59	0.41	0.80	1.60	1.12	2.19
**Italy**									
2000	3 739	3 407	4 052	3.44	3.15	3.74	6.60	6.01	7.15
2010	3 673	3 282	3 992	2.77	2.50	3.00	6.09	5.44	6.62
2019	4 032	3 535	4 452	2.66	2.34	2.93	6.69	5.86	7.38
**Portugal**									
2000	212	157	272	1.25	0.94	1.59	2.01	1.49	2.58
2010	316	236	400	1.51	1.13	1.89	2.93	2.18	3.70
2019	404	302	512	1.63	1.21	2.09	3.80	2.83	4.81
**Spain**									
2000	1 942	1 524	2 348	2.75	2.19	3.31	4.76	3.73	5.75
2010	2 436	1 968	2 894	2.78	2.23	3.33	5.19	4.19	6.16
2019	2 735	2 182	3 275	2.70	2.10	3.30	5.94	4.74	7.12
**Western Europe**									
2000	12 269	10 298	14 280	1.78	1.49	2.06	3.07	2.58	3.58
2010	15 253	12 759	17 860	1.84	1.54	2.14	3.61	3.02	4.23
2019	17 568	14 479	20 788	1.81	1.49	2.15	4.03	3.32	4.76

**Table 4:** Estimates of the impact of current health expenditure on the time trend of HBV and HCV disease burden

Location	Cause	Measure	Variable	Estimate	*p*.value	Lower limit of the 95% CI	Upper limit of the 95% CI
Greece	Acute hepatitis B	Incidence	(Intercept)	188.09	1.1E-01	-44.27	420.45
**Greece**	**Acute hepatitis B**	**Incidence**	**Yearly trend**	**-23.17**	**9.3E-03**	**-40.41**	**-5.94**
**Greece**	**Acute hepatitis B**	**Incidence**	**Yearly trend 2010–2019**	**24.00**	**2.0E-02**	**4.03**	**43.97**
**Greece**	**Acute hepatitis B**	**Incidence**	**Sex_Male**	**40.11**	**1.1E-02**	**9.77**	**70.46**
**Greece**	**Acute hepatitis B**	**Incidence**	**Age group**	**86.92**	**7.8E-34**	**76.32**	**97.52**
Greece	Acute hepatitis B	Incidence	% GPD 5Lag	-6.54	7.0E-01	-39.48	26.40
**Greece**	**Acute hepatitis B**	**Incidence**	**HIV incidence**	**159.30**	**1.8E-64**	**149.06**	**169.53**
Greece	Acute hepatitis B	Prevalence	(Intercept)	21.70	1.1E-01	-5.11	48.51
**Greece**	**Acute hepatitis B**	**Prevalence**	**Yearly trend**	**-2.67**	**9.3E-03**	**-4.66**	**-0.69**
**Greece**	**Acute hepatitis B**	**Prevalence**	**Yearly trend 2010–2019**	**2.77**	**2.0E-02**	**0.46**	**5.07**
**Greece**	**Acute hepatitis B**	**Prevalence**	**Sex_Male**	**4.63**	**1.1E-02**	**1.13**	**8.13**
**Greece**	**Acute hepatitis B**	**Prevalence**	**Age group**	**10.03**	**7.8E-34**	**8.81**	**11.25**
Greece	Acute hepatitis B	Prevalence	% GPD 5Lag	-0.75	7.0E-01	-4.56	3.05
**Greece**	**Acute hepatitis B**	**Prevalence**	**HIV incidence**	**18.38**	**1.8E-64**	**17.20**	**19.56**
Greece	Acute hepatitis B	Deaths	(Intercept)	-1.15	1.3E-01	-2.62	0.31
**Greece**	**Acute hepatitis B**	**Deaths**	**Yearly trend**	**0.19**	**1.0E-03**	**0.08**	**0.29**
**Greece**	**Acute hepatitis B**	**Deaths**	**Yearly trend 2010–2019**	**-0.26**	**1.0E-04**	**-0.38**	**-0.13**
**Greece**	**Acute hepatitis B**	**Deaths**	**Sex_Male**	**0.42**	**3.3E-05**	**0.23**	**0.61**
**Greece**	**Acute hepatitis B**	**Deaths**	**Age group**	**0.49**	**2.7E-29**	**0.42**	**0.55**
Greece	Acute hepatitis B	Deaths	% GPD 5Lag	-0.15	1.6E-01	-0.36	0.06
**Greece**	**Acute hepatitis B**	**Deaths**	**HIV incidence**	**-0.18**	**1.7E-07**	**-0.25**	**-0.12**
Greece	Acute hepatitis B	DALYs (Disability-Adjusted Life Years)	(Intercept)	-15.02	1.0E-01	-33.01	2.97
**Greece**	**Acute hepatitis B**	**DALYs (Disability-Adjusted Life Years)**	**Yearly trend**	**2.94**	**3.0E-05**	**1.60**	**4.27**
**Greece**	**Acute hepatitis B**	**DALYs (Disability-Adjusted Life Years)**	**Yearly trend 2010–2019**	**-4.11**	**6.4E-07**	**-5.66**	**-2.57**
**Greece**	**Acute hepatitis B**	**DALYs (Disability-Adjusted Life Years)**	**Sex_Male**	**9.05**	**4.6E-12**	**6.70**	**11.40**
**Greece**	**Acute hepatitis B**	**DALYs (Disability-Adjusted Life Years)**	**Age group**	**7.06**	**9.2E-36**	**6.23**	**7.88**
**Greece**	**Acute hepatitis B**	**DALYs (Disability-Adjusted Life Years)**	**% GPD 5Lag**	**-2.54**	**5.3E-02**	**-5.09**	**0.01**
**Greece**	**Acute hepatitis B**	**DALYs (Disability-Adjusted Life Years)**	**HIV incidence**	**-1.69**	**4.9E-05**	**-2.49**	**-0.90**
Greece	Acute hepatitis B	YLDs (Years Lived with Disability)	(Intercept)	-0.04	9.0E-01	-0.66	0.58
Greece	Acute hepatitis B	YLDs (Years Lived with Disability)	Yearly trend	-0.03	1.8E-01	-0.08	0.01
Greece	Acute hepatitis B	YLDs (Years Lived with Disability)	Yearly trend 2010–2019	0.04	1.7E-01	-0.02	0.09
**Greece**	**Acute hepatitis B**	**YLDs (Years Lived with Disability)**	**Sex_Male**	**0.10**	**1.9E-02**	**0.02**	**0.18**
**Greece**	**Acute hepatitis B**	**YLDs (Years Lived with Disability)**	**Age group**	**0.28**	**2.6E-41**	**0.25**	**0.30**
Greece	Acute hepatitis B	YLDs (Years Lived with Disability)	% GPD 5Lag	-0.01	8.6E-01	-0.10	0.08
**Greece**	**Acute hepatitis B**	**YLDs (Years Lived with Disability)**	**HIV incidence**	**0.31**	**9.2E-49**	**0.28**	**0.34**
Greece	Acute hepatitis B	YLLs (Years of Life Lost)	(Intercept)	-14.98	1.1E-01	-33.00	3.05
**Greece**	**Acute hepatitis B**	**YLLs (Years of Life Lost)**	**Yearly trend**	**2.97**	**2.6E-05**	**1.63**	**4.30**
**Greece**	**Acute hepatitis B**	**YLLs (Years of Life Lost)**	**Yearly trend 2010–2019**	**-4.15**	**5.4E-07**	**-5.70**	**-2.60**
**Greece**	**Acute hepatitis B**	**YLLs (Years of Life Lost)**	**Sex_Male**	**8.96**	**7.8E-12**	**6.60**	**11.31**
**Greece**	**Acute hepatitis B**	**YLLs (Years of Life Lost)**	**Age group**	**6.78**	**4.6E-34**	**5.96**	**7.60**
**Greece**	**Acute hepatitis B**	**YLLs (Years of Life Lost)**	**% GPD 5Lag**	**-2.53**	**5.4E-02**	**-5.09**	**0.03**
**Greece**	**Acute hepatitis B**	**YLLs (Years of Life Lost)**	**HIV incidence**	**-2.00**	**2.1E-06**	**-2.80**	**-1.21**
Greece	Acute hepatitis C	Incidence	(Intercept)	4.90	7.7E-01	-28.01	37.81
Greece	Acute hepatitis C	Incidence	Yearly trend	0.27	8.3E-01	-2.17	2.71
Greece	Acute hepatitis C	Incidence	Yearly trend 2010–2019	-0.31	8.3E-01	-3.14	2.52
Greece	Acute hepatitis C	Incidence	Sex_Male	-1.65	4.5E-01	-5.94	2.65
**Greece**	**Acute hepatitis C**	**Incidence**	**Age group**	**11.12**	**6.7E-30**	**9.62**	**12.62**
Greece	Acute hepatitis C	Incidence	% GPD 5Lag	-0.11	9.6E-01	-4.78	4.55
**Greece**	**Acute hepatitis C**	**Incidence**	**HIV incidence**	**-5.17**	**9.6E-11**	**-6.62**	**-3.72**
Greece	Acute hepatitis C	Prevalence	(Intercept)	0.57	7.7E-01	-3.23	4.36
Greece	Acute hepatitis C	Prevalence	Yearly trend	0.03	8.3E-01	-0.25	0.31
Greece	Acute hepatitis C	Prevalence	Yearly trend 2010–2019	-0.04	8.3E-01	-0.36	0.29
Greece	Acute hepatitis C	Prevalence	Sex_Male	-0.19	4.5E-01	-0.69	0.31
**Greece**	**Acute hepatitis C**	**Prevalence**	**Age group**	**1.28**	**6.7E-30**	**1.11**	**1.46**
Greece	Acute hepatitis C	Prevalence	% GPD 5Lag	-0.01	9.6E-01	-0.55	0.53
**Greece**	**Acute hepatitis C**	**Prevalence**	**HIV incidence**	**-0.60**	**9.6E-11**	**-0.76**	**-0.43**
Greece	Acute hepatitis C	Deaths	(Intercept)	-0.08	3.5E-01	-0.23	0.08
**Greece**	**Acute hepatitis C**	**Deaths**	**Yearly trend**	**0.01**	**1.5E-02**	**0.00**	**0.03**
**Greece**	**Acute hepatitis C**	**Deaths**	**Yearly trend 2010–2019**	**-0.02**	**5.2E-03**	**-0.03**	**-0.01**
**Greece**	**Acute hepatitis C**	**Deaths**	**Sex_Male**	**-0.03**	**8.5E-03**	**-0.05**	**-0.01**
**Greece**	**Acute hepatitis C**	**Deaths**	**Age group**	**0.03**	**3.1E-16**	**0.03**	**0.04**
Greece	Acute hepatitis C	Deaths	% GPD 5Lag	-0.01	4.1E-01	-0.03	0.01
**Greece**	**Acute hepatitis C**	**Deaths**	**HIV incidence**	**-0.01**	**1.2E-03**	**-0.02**	**0.00**
Greece	Acute hepatitis C	DALYs (Disability-Adjusted Life Years)	(Intercept)	-0.07	9.5E-01	-2.56	2.42
**Greece**	**Acute hepatitis C**	**DALYs (Disability-Adjusted Life Years)**	**Yearly trend**	**0.26**	**6.9E-03**	**0.07**	**0.44**
**Greece**	**Acute hepatitis C**	**DALYs (Disability-Adjusted Life Years)**	**Yearly trend 2010–2019**	**-0.35**	**1.6E-03**	**-0.57**	**-0.14**
**Greece**	**Acute hepatitis C**	**DALYs (Disability-Adjusted Life Years)**	**Sex_Male**	**-0.65**	**1.3E-04**	**-0.98**	**-0.33**
**Greece**	**Acute hepatitis C**	**DALYs (Disability-Adjusted Life Years)**	**Age group**	**0.27**	**5.1E-06**	**0.16**	**0.39**
Greece	Acute hepatitis C	DALYs (Disability-Adjusted Life Years)	% GPD 5Lag	-0.17	3.4E-01	-0.52	0.18
**Greece**	**Acute hepatitis C**	**DALYs (Disability-Adjusted Life Years)**	**HIV incidence**	**-0.16**	**3.9E-03**	**-0.27**	**-0.05**
Greece	Acute hepatitis C	YLDs (Years Lived with Disability)	(Intercept)	0.01	7.7E-01	-0.05	0.06
Greece	Acute hepatitis C	YLDs (Years Lived with Disability)	Yearly trend	0.00	8.3E-01	0.00	0.00
Greece	Acute hepatitis C	YLDs (Years Lived with Disability)	Yearly trend 2010–2019	0.00	8.3E-01	-0.01	0.00
Greece	Acute hepatitis C	YLDs (Years Lived with Disability)	Sex_Male	0.00	4.6E-01	-0.01	0.00
**Greece**	**Acute hepatitis C**	**YLDs (Years Lived with Disability)**	**Age group**	**0.02**	**6.4E-30**	**0.02**	**0.02**
Greece	Acute hepatitis C	YLDs (Years Lived with Disability)	% GPD 5Lag	0.00	9.6E-01	-0.01	0.01
**Greece**	**Acute hepatitis C**	**YLDs (Years Lived with Disability)**	**HIV incidence**	**-0.01**	**8.4E-11**	**-0.01**	**-0.01**
Greece	Acute hepatitis C	YLLs (Years of Life Lost)	(Intercept)	-0.08	9.5E-01	-2.53	2.37
**Greece**	**Acute hepatitis C**	**YLLs (Years of Life Lost)**	**Yearly trend**	**0.26**	**6.1E-03**	**0.08**	**0.44**
**Greece**	**Acute hepatitis C**	**YLLs (Years of Life Lost)**	**Yearly trend 2010–2019**	**-0.35**	**1.3E-03**	**-0.56**	**-0.14**
**Greece**	**Acute hepatitis C**	**YLLs (Years of Life Lost)**	**Sex_Male**	**-0.65**	**1.1E-04**	**-0.97**	**-0.33**
**Greece**	**Acute hepatitis C**	**YLLs (Years of Life Lost)**	**Age group**	**0.26**	**1.4E-05**	**0.14**	**0.37**
Greece	Acute hepatitis C	YLLs (Years of Life Lost)	% GPD 5Lag	-0.17	3.4E-01	-0.52	0.18
**Greece**	**Acute hepatitis C**	**YLLs (Years of Life Lost)**	**HIV incidence**	**-0.16**	**5.4E-03**	**-0.26**	**-0.05**
Italy	Acute hepatitis B	Incidence	(Intercept)	13.82	9.1E-01	-237.57	265.22
Italy	Acute hepatitis B	Incidence	Yearly trend	-7.39	8.5E-02	-15.76	0.97
Italy	Acute hepatitis B	Incidence	Yearly trend 2010–2019	2.39	5.4E-01	-5.28	10.06
**Italy**	**Acute hepatitis B**	**Incidence**	**Sex_Male**	**31.80**	**3.5E-07**	**20.14**	**43.47**
**Italy**	**Acute hepatitis B**	**Incidence**	**Age group**	**27.19**	**1.1E-25**	**23.06**	**31.33**
Italy	Acute hepatitis B	Incidence	% GPD 5Lag	4.27	8.2E-01	-33.28	41.82
**Italy**	**Acute hepatitis B**	**Incidence**	**HIV incidence**	**15.15**	**5.1E-40**	**13.55**	**16.75**
Italy	Acute hepatitis B	Prevalence	(Intercept)	1.60	9.1E-01	-27.41	30.60
Italy	Acute hepatitis B	Prevalence	Yearly trend	-0.85	8.5E-02	-1.82	0.11
Italy	Acute hepatitis B	Prevalence	Yearly trend 2010–2019	0.28	5.4E-01	-0.61	1.16
**Italy**	**Acute hepatitis B**	**Prevalence**	**Sex_Male**	**3.67**	**3.5E-07**	**2.32**	**5.02**
**Italy**	**Acute hepatitis B**	**Prevalence**	**Age group**	**3.14**	**1.1E-25**	**2.66**	**3.61**
Italy	Acute hepatitis B	Prevalence	% GPD 5Lag	0.49	8.2E-01	-3.84	4.82
**Italy**	**Acute hepatitis B**	**Prevalence**	**HIV incidence**	**1.75**	**5.1E-40**	**1.56**	**1.93**
Italy	Acute hepatitis B	Deaths	(Intercept)	-0.01	9.4E-01	-0.23	0.21
Italy	Acute hepatitis B	Deaths	Yearly trend	0.00	9.9E-01	-0.01	0.01
Italy	Acute hepatitis B	Deaths	Yearly trend 2010–2019	0.00	8.9E-01	-0.01	0.01
**Italy**	**Acute hepatitis B**	**Deaths**	**Sex_Male**	**0.04**	**1.5E-13**	**0.03**	**0.05**
**Italy**	**Acute hepatitis B**	**Deaths**	**Age group**	**0.04**	**1.7E-46**	**0.04**	**0.04**
Italy	Acute hepatitis B	Deaths	% GPD 5Lag	-0.01	6.6E-01	-0.04	0.03
**Italy**	**Acute hepatitis B**	**Deaths**	**HIV incidence**	**-0.01**	**4.5E-12**	**-0.01**	**0.00**
Italy	Acute hepatitis B	DALYs (Disability-Adjusted Life Years)	(Intercept)	1.69	6.0E-01	-4.53	7.91
Italy	Acute hepatitis B	DALYs (Disability-Adjusted Life Years)	Yearly trend	-0.05	6.4E-01	-0.26	0.16
Italy	Acute hepatitis B	DALYs (Disability-Adjusted Life Years)	Yearly trend 2010–2019	0.03	7.6E-01	-0.16	0.22
**Italy**	**Acute hepatitis B**	**DALYs (Disability-Adjusted Life Years)**	**Sex_Male**	**1.11**	**4.2E-12**	**0.83**	**1.40**
**Italy**	**Acute hepatitis B**	**DALYs (Disability-Adjusted Life Years)**	**Age group**	**0.48**	**3.8E-16**	**0.38**	**0.58**
Italy	Acute hepatitis B	DALYs (Disability-Adjusted Life Years)	% GPD 5Lag	-0.17	7.2E-01	-1.10	0.76
Italy	Acute hepatitis B	DALYs (Disability-Adjusted Life Years)	HIV incidence	0.00	9.2E-01	-0.04	0.04
Italy	Acute hepatitis B	YLDs (Years Lived with Disability)	(Intercept)	-0.08	8.0E-01	-0.74	0.57
Italy	Acute hepatitis B	YLDs (Years Lived with Disability)	Yearly trend	-0.02	1.7E-01	-0.04	0.01
Italy	Acute hepatitis B	YLDs (Years Lived with Disability)	Yearly trend 2010–2019	0.00	7.3E-01	-0.02	0.02
**Italy**	**Acute hepatitis B**	**YLDs (Years Lived with Disability)**	**Sex_Male**	**0.08**	**3.8E-07**	**0.05**	**0.11**
**Italy**	**Acute hepatitis B**	**YLDs (Years Lived with Disability)**	**Age group**	**0.09**	**1.2E-34**	**0.08**	**0.10**
Italy	Acute hepatitis B	YLDs (Years Lived with Disability)	% GPD 5Lag	0.01	7.9E-01	-0.08	0.11
**Italy**	**Acute hepatitis B**	**YLDs (Years Lived with Disability)**	**HIV incidence**	**0.04**	**1.1E-36**	**0.03**	**0.04**
Italy	Acute hepatitis B	YLLs (Years of Life Lost)	(Intercept)	1.77	5.6E-01	-4.11	7.66
Italy	Acute hepatitis B	YLLs (Years of Life Lost)	Yearly trend	-0.03	7.3E-01	-0.23	0.16
Italy	Acute hepatitis B	YLLs (Years of Life Lost)	Yearly trend 2010–2019	0.03	7.8E-01	-0.15	0.21
**Italy**	**Acute hepatitis B**	**YLLs (Years of Life Lost)**	**Sex_Male**	**1.03**	**1.0E-11**	**0.76**	**1.30**
**Italy**	**Acute hepatitis B**	**YLLs (Years of Life Lost)**	**Age group**	**0.39**	**6.3E-13**	**0.29**	**0.49**
Italy	Acute hepatitis B	YLLs (Years of Life Lost)	% GPD 5Lag	-0.18	6.9E-01	-1.06	0.70
Italy	Acute hepatitis B	YLLs (Years of Life Lost)	HIV incidence	-0.03	7.3E-02	-0.07	0.00
Italy	Acute hepatitis C	Incidence	(Intercept)	124.17	1.5E-01	-45.77	294.10
Italy	Acute hepatitis C	Incidence	Yearly trend	0.50	8.6E-01	-5.15	6.15
Italy	Acute hepatitis C	Incidence	Yearly trend 2010–2019	-0.37	8.9E-01	-5.55	4.82
Italy	Acute hepatitis C	Incidence	Sex_Male	0.38	9.3E-01	-7.51	8.27
**Italy**	**Acute hepatitis C**	**Incidence**	**Age group**	**18.45**	**8.0E-26**	**15.65**	**21.24**
Italy	Acute hepatitis C	Incidence	% GPD 5Lag	-8.93	4.9E-01	-34.31	16.46
**Italy**	**Acute hepatitis C**	**Incidence**	**HIV incidence**	**-5.06**	**4.3E-16**	**-6.13**	**-3.98**
Italy	Acute hepatitis C	Prevalence	(Intercept)	14.33	1.5E-01	-5.28	33.93
Italy	Acute hepatitis C	Prevalence	Yearly trend	0.06	8.6E-01	-0.59	0.71
Italy	Acute hepatitis C	Prevalence	Yearly trend 2010–2019	-0.04	8.9E-01	-0.64	0.56
Italy	Acute hepatitis C	Prevalence	Sex_Male	0.04	9.3E-01	-0.87	0.95
**Italy**	**Acute hepatitis C**	**Prevalence**	**Age group**	**2.13**	**8.0E-26**	**1.81**	**2.45**
Italy	Acute hepatitis C	Prevalence	% GPD 5Lag	-1.03	4.9E-01	-3.96	1.90
**Italy**	**Acute hepatitis C**	**Prevalence**	**HIV incidence**	**-0.58**	**4.3E-16**	**-0.71**	**-0.46**
Italy	Acute hepatitis C	Deaths	(Intercept)	0.04	3.5E-01	-0.05	0.13
**Italy**	**Acute hepatitis C**	**Deaths**	**Yearly trend**	**0.00**	**1.8E-02**	**-0.01**	**0.00**
**Italy**	**Acute hepatitis C**	**Deaths**	**Yearly trend 2010–2019**	**0.00**	**9.5E-03**	**0.00**	**0.01**
**Italy**	**Acute hepatitis C**	**Deaths**	**Sex_Male**	**0.01**	**8.6E-03**	**0.00**	**0.01**
**Italy**	**Acute hepatitis C**	**Deaths**	**Age group**	**0.01**	**1.1E-31**	**0.01**	**0.01**
Italy	Acute hepatitis C	Deaths	% GPD 5Lag	0.00	6.4E-01	-0.02	0.01
**Italy**	**Acute hepatitis C**	**Deaths**	**HIV incidence**	**0.00**	**5.1E-09**	**0.00**	**0.00**
Italy	Acute hepatitis C	DALYs (Disability-Adjusted Life Years)	(Intercept)	1.30	1.2E-01	-0.34	2.93
**Italy**	**Acute hepatitis C**	**DALYs (Disability-Adjusted Life Years)**	**Yearly trend**	**-0.07**	**1.1E-02**	**-0.13**	**-0.02**
**Italy**	**Acute hepatitis C**	**DALYs (Disability-Adjusted Life Years)**	**Yearly trend 2010–2019**	**0.07**	**8.0E-03**	**0.02**	**0.12**
**Italy**	**Acute hepatitis C**	**DALYs (Disability-Adjusted Life Years)**	**Sex_Male**	**0.12**	**2.2E-03**	**0.04**	**0.20**
**Italy**	**Acute hepatitis C**	**DALYs (Disability-Adjusted Life Years)**	**Age group**	**0.16**	**4.5E-22**	**0.13**	**0.18**
Italy	Acute hepatitis C	DALYs (Disability-Adjusted Life Years)	% GPD 5Lag	-0.07	5.6E-01	-0.32	0.17
**Italy**	**Acute hepatitis C**	**DALYs (Disability-Adjusted Life Years)**	**HIV incidence**	**-0.03**	**2.6E-07**	**-0.04**	**-0.02**
Italy	Acute hepatitis C	YLDs (Years Lived with Disability)	(Intercept)	0.20	1.5E-01	-0.07	0.48
Italy	Acute hepatitis C	YLDs (Years Lived with Disability)	Yearly trend	0.00	8.6E-01	-0.01	0.01
Italy	Acute hepatitis C	YLDs (Years Lived with Disability)	Yearly trend 2010–2019	0.00	8.9E-01	-0.01	0.01
Italy	Acute hepatitis C	YLDs (Years Lived with Disability)	Sex_Male	0.00	9.2E-01	-0.01	0.01
**Italy**	**Acute hepatitis C**	**YLDs (Years Lived with Disability)**	**Age group**	**0.03**	**7.7E-26**	**0.03**	**0.03**
Italy	Acute hepatitis C	YLDs (Years Lived with Disability)	% GPD 5Lag	-0.01	4.9E-01	-0.06	0.03
**Italy**	**Acute hepatitis C**	**YLDs (Years Lived with Disability)**	**HIV incidence**	**-0.01**	**3.7E-16**	**-0.01**	**-0.01**
Italy	Acute hepatitis C	YLLs (Years of Life Lost)	(Intercept)	1.10	1.4E-01	-0.35	2.54
**Italy**	**Acute hepatitis C**	**YLLs (Years of Life Lost)**	**Yearly trend**	**-0.07**	**3.7E-03**	**-0.12**	**-0.02**
**Italy**	**Acute hepatitis C**	**YLLs (Years of Life Lost)**	**Yearly trend 2010–2019**	**0.07**	**2.5E-03**	**0.03**	**0.11**
**Italy**	**Acute hepatitis C**	**YLLs (Years of Life Lost)**	**Sex_Male**	**0.12**	**6.0E-04**	**0.05**	**0.19**
**Italy**	**Acute hepatitis C**	**YLLs (Years of Life Lost)**	**Age group**	**0.13**	**1.3E-19**	**0.10**	**0.15**
Italy	Acute hepatitis C	YLLs (Years of Life Lost)	% GPD 5Lag	-0.06	5.9E-01	-0.27	0.16
**Italy**	**Acute hepatitis C**	**YLLs (Years of Life Lost)**	**HIV incidence**	**-0.02**	**2.4E-05**	**-0.03**	**-0.01**
Portugal	Acute hepatitis B	Incidence	(Intercept)	143.31	3.2E-01	-139.01	425.64
Portugal	Acute hepatitis B	Incidence	Yearly trend	-1.84	8.0E-01	-16.20	12.52
Portugal	Acute hepatitis B	Incidence	Yearly trend 2010–2019	-4.96	5.3E-01	-20.52	10.60
**Portugal**	**Acute hepatitis B**	**Incidence**	**Sex_Male**	**39.08**	**1.8E-04**	**19.18**	**58.99**
**Portugal**	**Acute hepatitis B**	**Incidence**	**Age group**	**14.70**	**4.3E-05**	**7.88**	**21.53**
Portugal	Acute hepatitis B	Incidence	% GPD 5Lag	-8.36	6.8E-01	-48.10	31.38
**Portugal**	**Acute hepatitis B**	**Incidence**	**HIV incidence**	**8.10**	**2.0E-38**	**7.22**	**8.99**
Portugal	Acute hepatitis B	Prevalence	(Intercept)	16.54	3.2E-01	-16.04	49.11
Portugal	Acute hepatitis B	Prevalence	Yearly trend	-0.21	8.0E-01	-1.87	1.44
Portugal	Acute hepatitis B	Prevalence	Yearly trend 2010–2019	-0.57	5.3E-01	-2.37	1.22
**Portugal**	**Acute hepatitis B**	**Prevalence**	**Sex_Male**	**4.51**	**1.8E-04**	**2.21**	**6.81**
**Portugal**	**Acute hepatitis B**	**Prevalence**	**Age group**	**1.70**	**4.3E-05**	**0.91**	**2.48**
Portugal	Acute hepatitis B	Prevalence	% GPD 5Lag	-0.96	6.8E-01	-5.55	3.62
**Portugal**	**Acute hepatitis B**	**Prevalence**	**HIV incidence**	**0.94**	**2.0E-38**	**0.83**	**1.04**
Portugal	Acute hepatitis B	Deaths	(Intercept)	-0.01	7.2E-01	-0.07	0.05
Portugal	Acute hepatitis B	Deaths	Yearly trend	0.00	2.5E-01	-0.01	0.00
Portugal	Acute hepatitis B	Deaths	Yearly trend 2010–2019	0.00	3.3E-01	0.00	0.01
**Portugal**	**Acute hepatitis B**	**Deaths**	**Sex_Male**	**0.02**	**2.3E-19**	**0.02**	**0.03**
**Portugal**	**Acute hepatitis B**	**Deaths**	**Age group**	**0.01**	**1.8E-41**	**0.01**	**0.02**
Portugal	Acute hepatitis B	Deaths	% GPD 5Lag	0.00	9.7E-01	-0.01	0.01
**Portugal**	**Acute hepatitis B**	**Deaths**	**HIV incidence**	**0.00**	**9.4E-04**	**0.00**	**0.00**
Portugal	Acute hepatitis B	DALYs (Disability-Adjusted Life Years)	(Intercept)	0.49	6.8E-01	-1.82	2.81
Portugal	Acute hepatitis B	DALYs (Disability-Adjusted Life Years)	Yearly trend	-0.04	4.6E-01	-0.16	0.07
Portugal	Acute hepatitis B	DALYs (Disability-Adjusted Life Years)	Yearly trend 2010–2019	0.02	7.7E-01	-0.11	0.15
**Portugal**	**Acute hepatitis B**	**DALYs (Disability-Adjusted Life Years)**	**Sex_Male**	**0.63**	**4.4E-12**	**0.47**	**0.79**
**Portugal**	**Acute hepatitis B**	**DALYs (Disability-Adjusted Life Years)**	**Age group**	**0.30**	**1.8E-19**	**0.24**	**0.36**
Portugal	Acute hepatitis B	DALYs (Disability-Adjusted Life Years)	% GPD 5Lag	-0.04	8.0E-01	-0.37	0.28
**Portugal**	**Acute hepatitis B**	**DALYs (Disability-Adjusted Life Years)**	**HIV incidence**	**0.02**	**5.5E-10**	**0.02**	**0.03**
Portugal	Acute hepatitis B	YLDs (Years Lived with Disability)	(Intercept)	0.07	8.2E-01	-0.53	0.67
Portugal	Acute hepatitis B	YLDs (Years Lived with Disability)	Yearly trend	0.00	8.2E-01	-0.03	0.03
Portugal	Acute hepatitis B	YLDs (Years Lived with Disability)	Yearly trend 2010–2019	-0.01	3.9E-01	-0.05	0.02
**Portugal**	**Acute hepatitis B**	**YLDs (Years Lived with Disability)**	**Sex_Male**	**0.09**	**2.7E-05**	**0.05**	**0.14**
**Portugal**	**Acute hepatitis B**	**YLDs (Years Lived with Disability)**	**Age group**	**0.07**	**3.0E-17**	**0.06**	**0.09**
Portugal	Acute hepatitis B	YLDs (Years Lived with Disability)	% GPD 5Lag	-0.01	7.3E-01	-0.10	0.07
**Portugal**	**Acute hepatitis B**	**YLDs (Years Lived with Disability)**	**HIV incidence**	**0.02**	**8.2E-37**	**0.01**	**0.02**
Portugal	Acute hepatitis B	YLLs (Years of Life Lost)	(Intercept)	0.42	6.5E-01	-1.42	2.27
Portugal	Acute hepatitis B	YLLs (Years of Life Lost)	Yearly trend	-0.05	3.2E-01	-0.14	0.05
Portugal	Acute hepatitis B	YLLs (Years of Life Lost)	Yearly trend 2010–2019	0.03	5.2E-01	-0.07	0.13
**Portugal**	**Acute hepatitis B**	**YLLs (Years of Life Lost)**	**Sex_Male**	**0.54**	**2.4E-13**	**0.41**	**0.67**
**Portugal**	**Acute hepatitis B**	**YLLs (Years of Life Lost)**	**Age group**	**0.23**	**2.8E-18**	**0.18**	**0.27**
Portugal	Acute hepatitis B	YLLs (Years of Life Lost)	% GPD 5Lag	-0.03	8.3E-01	-0.29	0.23
**Portugal**	**Acute hepatitis B**	**YLLs (Years of Life Lost)**	**HIV incidence**	**0.01**	**7.0E-03**	**0.00**	**0.01**
Portugal	Acute hepatitis C	Incidence	(Intercept)	18.09	6.4E-01	-57.52	93.71
Portugal	Acute hepatitis C	Incidence	Yearly trend	-0.40	8.4E-01	-4.25	3.45
Portugal	Acute hepatitis C	Incidence	Yearly trend 2010–2019	0.75	7.2E-01	-3.41	4.92
**Portugal**	**Acute hepatitis C**	**Incidence**	**Sex_Male**	**10.58**	**1.5E-04**	**5.25**	**15.91**
**Portugal**	**Acute hepatitis C**	**Incidence**	**Age group**	**12.07**	**7.6E-26**	**10.24**	**13.90**
Portugal	Acute hepatitis C	Incidence	% GPD 5Lag	-0.11	9.8E-01	-10.75	10.54
**Portugal**	**Acute hepatitis C**	**Incidence**	**HIV incidence**	**-0.83**	**2.0E-10**	**-1.07**	**-0.59**
Portugal	Acute hepatitis C	Prevalence	(Intercept)	2.09	6.4E-01	-6.64	10.81
Portugal	Acute hepatitis C	Prevalence	Yearly trend	-0.05	8.4E-01	-0.49	0.40
Portugal	Acute hepatitis C	Prevalence	Yearly trend 2010–2019	0.09	7.2E-01	-0.39	0.57
**Portugal**	**Acute hepatitis C**	**Prevalence**	**Sex_Male**	**1.22**	**1.5E-04**	**0.61**	**1.84**
**Portugal**	**Acute hepatitis C**	**Prevalence**	**Age group**	**1.39**	**7.6E-26**	**1.18**	**1.60**
Portugal	Acute hepatitis C	Prevalence	% GPD 5Lag	-0.01	9.8E-01	-1.24	1.22
**Portugal**	**Acute hepatitis C**	**Prevalence**	**HIV incidence**	**-0.10**	**2.0E-10**	**-0.12**	**-0.07**
Portugal	Acute hepatitis C	Deaths	(Intercept)	0.02	8.8E-02	0.00	0.04
**Portugal**	**Acute hepatitis C**	**Deaths**	**Yearly trend**	**0.00**	**4.4E-02**	**0.00**	**0.00**
Portugal	Acute hepatitis C	Deaths	Yearly trend 2010–2019	0.00	2.7E-01	0.00	0.00
**Portugal**	**Acute hepatitis C**	**Deaths**	**Sex_Male**	**0.00**	**3.1E-03**	**0.00**	**0.00**
**Portugal**	**Acute hepatitis C**	**Deaths**	**Age group**	**0.00**	**1.4E-37**	**0.00**	**0.01**
Portugal	Acute hepatitis C	Deaths	% GPD 5Lag	0.00	3.6E-01	0.00	0.00
Portugal	Acute hepatitis C	Deaths	HIV incidence	0.00	5.1E-01	0.00	0.00
**Portugal**	**Acute hepatitis C**	**DALYs (Disability-Adjusted Life Years)**	**(Intercept)**	**0.83**	**2.4E-02**	**0.12**	**1.54**
Portugal	Acute hepatitis C	DALYs (Disability-Adjusted Life Years)	Yearly trend	-0.03	8.4E-02	-0.07	0.00
Portugal	Acute hepatitis C	DALYs (Disability-Adjusted Life Years)	Yearly trend 2010–2019	0.02	4.0E-01	-0.02	0.06
**Portugal**	**Acute hepatitis C**	**DALYs (Disability-Adjusted Life Years)**	**Sex_Male**	**0.09**	**8.0E-04**	**0.04**	**0.14**
**Portugal**	**Acute hepatitis C**	**DALYs (Disability-Adjusted Life Years)**	**Age group**	**0.06**	**4.4E-11**	**0.05**	**0.08**
Portugal	Acute hepatitis C	DALYs (Disability-Adjusted Life Years)	% GPD 5Lag	-0.04	4.0E-01	-0.14	0.06
**Portugal**	**Acute hepatitis C**	**DALYs (Disability-Adjusted Life Years)**	**HIV incidence**	**0.00**	**2.8E-02**	**0.00**	**0.00**
Portugal	Acute hepatitis C	YLDs (Years Lived with Disability)	(Intercept)	0.03	6.4E-01	-0.09	0.15
Portugal	Acute hepatitis C	YLDs (Years Lived with Disability)	Yearly trend	0.00	8.4E-01	-0.01	0.01
Portugal	Acute hepatitis C	YLDs (Years Lived with Disability)	Yearly trend 2010–2019	0.00	7.2E-01	-0.01	0.01
**Portugal**	**Acute hepatitis C**	**YLDs (Years Lived with Disability)**	**Sex_Male**	**0.02**	**1.5E-04**	**0.01**	**0.03**
**Portugal**	**Acute hepatitis C**	**YLDs (Years Lived with Disability)**	**Age group**	**0.02**	**7.7E-26**	**0.02**	**0.02**
Portugal	Acute hepatitis C	YLDs (Years Lived with Disability)	% GPD 5Lag	0.00	9.9E-01	-0.02	0.02
**Portugal**	**Acute hepatitis C**	**YLDs (Years Lived with Disability)**	**HIV incidence**	**0.00**	**1.8E-10**	**0.00**	**0.00**
**Portugal**	**Acute hepatitis C**	**YLLs (Years of Life Lost)**	**(Intercept)**	**0.80**	**1.7E-02**	**0.15**	**1.44**
Portugal	Acute hepatitis C	YLLs (Years of Life Lost)	Yearly trend	-0.03	6.4E-02	-0.06	0.00
Portugal	Acute hepatitis C	YLLs (Years of Life Lost)	Yearly trend 2010–2019	0.02	3.9E-01	-0.02	0.05
**Portugal**	**Acute hepatitis C**	**YLLs (Years of Life Lost)**	**Sex_Male**	**0.07**	**3.0E-03**	**0.02**	**0.12**
**Portugal**	**Acute hepatitis C**	**YLLs (Years of Life Lost)**	**Age group**	**0.04**	**3.2E-07**	**0.03**	**0.06**
Portugal	Acute hepatitis C	YLLs (Years of Life Lost)	% GPD 5Lag	-0.04	3.6E-01	-0.13	0.05
**Portugal**	**Acute hepatitis C**	**YLLs (Years of Life Lost)**	**HIV incidence**	**0.00**	**2.6E-04**	**0.00**	**0.01**
Spain	Acute hepatitis B	Incidence	(Intercept)	46.56	7.1E-01	-197.79	290.90
Spain	Acute hepatitis B	Incidence	Yearly trend	-1.79	8.3E-01	-17.80	14.22
Spain	Acute hepatitis B	Incidence	Yearly trend 2010–2019	-4.38	5.4E-01	-18.54	9.77
Spain	Acute hepatitis B	Incidence	Sex_Male	14.11	2.4E-01	-9.55	37.78
**Spain**	**Acute hepatitis B**	**Incidence**	**Age group**	**34.51**	**5.0E-14**	**26.42**	**42.61**
Spain	Acute hepatitis B	Incidence	% GPD 5Lag	-1.23	9.6E-01	-45.46	42.99
**Spain**	**Acute hepatitis B**	**Incidence**	**HIV incidence**	**10.84**	**7.8E-24**	**9.10**	**12.59**
Spain	Acute hepatitis B	Prevalence	(Intercept)	5.37	7.1E-01	-22.82	33.57
Spain	Acute hepatitis B	Prevalence	Yearly trend	-0.21	8.3E-01	-2.05	1.64
Spain	Acute hepatitis B	Prevalence	Yearly trend 2010–2019	-0.51	5.4E-01	-2.14	1.13
Spain	Acute hepatitis B	Prevalence	Sex_Male	1.63	2.4E-01	-1.10	4.36
**Spain**	**Acute hepatitis B**	**Prevalence**	**Age group**	**3.98**	**5.0E-14**	**3.05**	**4.92**
Spain	Acute hepatitis B	Prevalence	% GPD 5Lag	-0.14	9.6E-01	-5.25	4.96
**Spain**	**Acute hepatitis B**	**Prevalence**	**HIV incidence**	**1.25**	**7.8E-24**	**1.05**	**1.45**
Spain	Acute hepatitis B	Deaths	(Intercept)	-0.03	1.4E-01	-0.06	0.01
Spain	Acute hepatitis B	Deaths	Yearly trend	0.00	6.2E-01	0.00	0.00
Spain	Acute hepatitis B	Deaths	Yearly trend 2010–2019	0.00	6.4E-01	0.00	0.00
**Spain**	**Acute hepatitis B**	**Deaths**	**Sex_Male**	**0.01**	**6.4E-14**	**0.01**	**0.02**
**Spain**	**Acute hepatitis B**	**Deaths**	**Age group**	**0.01**	**6.7E-31**	**0.01**	**0.01**
Spain	Acute hepatitis B	Deaths	% GPD 5Lag	0.00	8.0E-01	-0.01	0.01
**Spain**	**Acute hepatitis B**	**Deaths**	**HIV incidence**	**0.00**	**3.1E-06**	**0.00**	**0.00**
Spain	Acute hepatitis B	DALYs (Disability-Adjusted Life Years)	(Intercept)	0.13	8.5E-01	-1.23	1.49
Spain	Acute hepatitis B	DALYs (Disability-Adjusted Life Years)	Yearly trend	0.02	7.0E-01	-0.07	0.11
Spain	Acute hepatitis B	DALYs (Disability-Adjusted Life Years)	Yearly trend 2010–2019	-0.03	3.9E-01	-0.11	0.04
**Spain**	**Acute hepatitis B**	**DALYs (Disability-Adjusted Life Years)**	**Sex_Male**	**0.36**	**3.1E-07**	**0.23**	**0.49**
**Spain**	**Acute hepatitis B**	**DALYs (Disability-Adjusted Life Years)**	**Age group**	**0.11**	**6.0E-06**	**0.06**	**0.15**
Spain	Acute hepatitis B	DALYs (Disability-Adjusted Life Years)	% GPD 5Lag	0.01	9.5E-01	-0.24	0.25
**Spain**	**Acute hepatitis B**	**DALYs (Disability-Adjusted Life Years)**	**HIV incidence**	**0.02**	**3.0E-04**	**0.01**	**0.03**
Spain	Acute hepatitis B	YLDs (Years Lived with Disability)	(Intercept)	-0.08	7.8E-01	-0.62	0.47
Spain	Acute hepatitis B	YLDs (Years Lived with Disability)	Yearly trend	0.00	9.6E-01	-0.04	0.03
Spain	Acute hepatitis B	YLDs (Years Lived with Disability)	Yearly trend 2010–2019	-0.01	5.0E-01	-0.04	0.02
Spain	Acute hepatitis B	YLDs (Years Lived with Disability)	Sex_Male	0.03	2.2E-01	-0.02	0.09
**Spain**	**Acute hepatitis B**	**YLDs (Years Lived with Disability)**	**Age group**	**0.11**	**1.3E-24**	**0.10**	**0.13**
Spain	Acute hepatitis B	YLDs (Years Lived with Disability)	% GPD 5Lag	0.00	9.4E-01	-0.09	0.10
**Spain**	**Acute hepatitis B**	**YLDs (Years Lived with Disability)**	**HIV incidence**	**0.02**	**2.0E-23**	**0.02**	**0.03**
Spain	Acute hepatitis B	YLLs (Years of Life Lost)	(Intercept)	0.21	7.2E-01	-0.92	1.34
Spain	Acute hepatitis B	YLLs (Years of Life Lost)	Yearly trend	0.02	6.3E-01	-0.06	0.09
Spain	Acute hepatitis B	YLLs (Years of Life Lost)	Yearly trend 2010–2019	-0.02	4.8E-01	-0.09	0.04
**Spain**	**Acute hepatitis B**	**YLLs (Years of Life Lost)**	**Sex_Male**	**0.33**	**3.1E-08**	**0.22**	**0.44**
Spain	Acute hepatitis B	YLLs (Years of Life Lost)	Age group	-0.01	7.3E-01	-0.04	0.03
Spain	Acute hepatitis B	YLLs (Years of Life Lost)	% GPD 5Lag	0.00	9.6E-01	-0.20	0.21
Spain	Acute hepatitis B	YLLs (Years of Life Lost)	HIV incidence	-0.01	1.9E-01	-0.01	0.00
Spain	Acute hepatitis C	Incidence	(Intercept)	-16.48	6.3E-01	-82.62	49.66
Spain	Acute hepatitis C	Incidence	Yearly trend	2.49	2.6E-01	-1.85	6.82
Spain	Acute hepatitis C	Incidence	Yearly trend 2010–2019	-2.01	3.1E-01	-5.84	1.82
**Spain**	**Acute hepatitis C**	**Incidence**	**Sex_Male**	**11.61**	**5.2E-04**	**5.21**	**18.02**
**Spain**	**Acute hepatitis C**	**Incidence**	**Age group**	**17.64**	**3.9E-33**	**15.45**	**19.83**
Spain	Acute hepatitis C	Incidence	% GPD 5Lag	-0.73	9.0E-01	-12.70	11.24
**Spain**	**Acute hepatitis C**	**Incidence**	**HIV incidence**	**-1.46**	**1.1E-08**	**-1.94**	**-0.99**
Spain	Acute hepatitis C	Prevalence	(Intercept)	-1.90	6.3E-01	-9.53	5.73
Spain	Acute hepatitis C	Prevalence	Yearly trend	0.29	2.6E-01	-0.21	0.79
Spain	Acute hepatitis C	Prevalence	Yearly trend 2010–2019	-0.23	3.1E-01	-0.67	0.21
**Spain**	**Acute hepatitis C**	**Prevalence**	**Sex_Male**	**1.34**	**5.2E-04**	**0.60**	**2.08**
**Spain**	**Acute hepatitis C**	**Prevalence**	**Age group**	**2.04**	**3.9E-33**	**1.78**	**2.29**
Spain	Acute hepatitis C	Prevalence	% GPD 5Lag	-0.08	9.0E-01	-1.47	1.30
**Spain**	**Acute hepatitis C**	**Prevalence**	**HIV incidence**	**-0.17**	**1.1E-08**	**-0.22**	**-0.11**
Spain	Acute hepatitis C	Deaths	(Intercept)	0.01	4.6E-01	-0.02	0.04
Spain	Acute hepatitis C	Deaths	Yearly trend	0.00	1.2E-01	0.00	0.00
Spain	Acute hepatitis C	Deaths	Yearly trend 2010–2019	0.00	1.2E-01	0.00	0.00
**Spain**	**Acute hepatitis C**	**Deaths**	**Sex_Male**	**0.01**	**2.3E-04**	**0.00**	**0.01**
**Spain**	**Acute hepatitis C**	**Deaths**	**Age group**	**0.01**	**1.1E-40**	**0.01**	**0.01**
Spain	Acute hepatitis C	Deaths	% GPD 5Lag	0.00	6.1E-01	-0.01	0.00
**Spain**	**Acute hepatitis C**	**Deaths**	**HIV incidence**	**0.00**	**1.5E-04**	**0.00**	**0.00**
Spain	Acute hepatitis C	DALYs (Disability-Adjusted Life Years)	(Intercept)	0.77	6.3E-02	-0.04	1.57
Spain	Acute hepatitis C	DALYs (Disability-Adjusted Life Years)	Yearly trend	-0.03	1.9E-01	-0.09	0.02
Spain	Acute hepatitis C	DALYs (Disability-Adjusted Life Years)	Yearly trend 2010–2019	0.03	2.5E-01	-0.02	0.07
**Spain**	**Acute hepatitis C**	**DALYs (Disability-Adjusted Life Years)**	**Sex_Male**	**0.12**	**4.2E-03**	**0.04**	**0.19**
**Spain**	**Acute hepatitis C**	**DALYs (Disability-Adjusted Life Years)**	**Age group**	**0.12**	**2.4E-15**	**0.09**	**0.15**
Spain	Acute hepatitis C	DALYs (Disability-Adjusted Life Years)	% GPD 5Lag	-0.04	5.6E-01	-0.19	0.10
Spain	Acute hepatitis C	DALYs (Disability-Adjusted Life Years)	HIV incidence	0.00	1.5E-01	-0.01	0.00
Spain	Acute hepatitis C	YLDs (Years Lived with Disability)	(Intercept)	-0.03	6.3E-01	-0.13	0.08
Spain	Acute hepatitis C	YLDs (Years Lived with Disability)	Yearly trend	0.00	2.6E-01	0.00	0.01
Spain	Acute hepatitis C	YLDs (Years Lived with Disability)	Yearly trend 2010–2019	0.00	3.1E-01	-0.01	0.00
**Spain**	**Acute hepatitis C**	**YLDs (Years Lived with Disability)**	**Sex_Male**	**0.02**	**5.1E-04**	**0.01**	**0.03**
**Spain**	**Acute hepatitis C**	**YLDs (Years Lived with Disability)**	**Age group**	**0.03**	**4.5E-33**	**0.03**	**0.03**
Spain	Acute hepatitis C	YLDs (Years Lived with Disability)	% GPD 5Lag	0.00	9.0E-01	-0.02	0.02
**Spain**	**Acute hepatitis C**	**YLDs (Years Lived with Disability)**	**HIV incidence**	**0.00**	**1.0E-08**	**0.00**	**0.00**
**Spain**	**Acute hepatitis C**	**YLLs (Years of Life Lost)**	**(Intercept)**	**0.79**	**3.6E-02**	**0.06**	**1.53**
Spain	Acute hepatitis C	YLLs (Years of Life Lost)	Yearly trend	-0.04	1.2E-01	-0.09	0.01
Spain	Acute hepatitis C	YLLs (Years of Life Lost)	Yearly trend 2010–2019	0.03	1.6E-01	-0.01	0.07
**Spain**	**Acute hepatitis C**	**YLLs (Years of Life Lost)**	**Sex_Male**	**0.10**	**8.9E-03**	**0.03**	**0.17**
**Spain**	**Acute hepatitis C**	**YLLs (Years of Life Lost)**	**Age group**	**0.09**	**1.2E-11**	**0.07**	**0.12**
Spain	Acute hepatitis C	YLLs (Years of Life Lost)	% GPD 5Lag	-0.04	5.4E-01	-0.17	0.09
Spain	Acute hepatitis C	YLLs (Years of Life Lost)	HIV incidence	0.00	5.0E-01	-0.01	0.00
Greece	Cirrhosis and other chronic liver diseases due to hepatitis B	DALYs (Disability-Adjusted Life Years)	(Intercept)	11.22	5.7E-01	-27.82	50.26
**Greece**	**Cirrhosis and other chronic liver diseases due to hepatitis B**	**DALYs (Disability-Adjusted Life Years)**	**Age group**	**13.45**	**7.1E-29**	**11.58**	**15.32**
**Greece**	**Cirrhosis and other chronic liver diseases due to hepatitis B**	**DALYs (Disability-Adjusted Life Years)**	**Yearly trend 2010–2019**	**3.81**	**2.7E-02**	**0.46**	**7.15**
Greece	Cirrhosis and other chronic liver diseases due to hepatitis B	DALYs (Disability-Adjusted Life Years)	% GPD 5Lag	-0.31	9.1E-01	-5.84	5.21
**Greece**	**Cirrhosis and other chronic liver diseases due to hepatitis B**	**DALYs (Disability-Adjusted Life Years)**	**Sex_Male**	**21.13**	**1.8E-12**	**15.77**	**26.49**
**Greece**	**Cirrhosis and other chronic liver diseases due to hepatitis B**	**DALYs (Disability-Adjusted Life Years)**	**Yearly trend**	**-4.14**	**5.7E-03**	**-7.04**	**-1.25**
Greece	Cirrhosis and other chronic liver diseases due to hepatitis B	DALYs (Disability-Adjusted Life Years)	HIV prevalence_P	0.07	2.6E-01	-0.05	0.19
Greece	Cirrhosis and other chronic liver diseases due to hepatitis B	Deaths	(Intercept)	0.05	9.6E-01	-1.99	2.10
**Greece**	**Cirrhosis and other chronic liver diseases due to hepatitis B**	**Deaths**	**Age group**	**0.92**	**1.3E-39**	**0.82**	**1.02**
**Greece**	**Cirrhosis and other chronic liver diseases due to hepatitis B**	**Deaths**	**Yearly trend 2010–2019**	**0.21**	**2.2E-02**	**0.03**	**0.38**
Greece	Cirrhosis and other chronic liver diseases due to hepatitis B	Deaths	% GPD 5Lag	-0.02	8.8E-01	-0.31	0.27
**Greece**	**Cirrhosis and other chronic liver diseases due to hepatitis B**	**Deaths**	**Sex_Male**	**1.15**	**2.9E-13**	**0.87**	**1.43**
**Greece**	**Cirrhosis and other chronic liver diseases due to hepatitis B**	**Deaths**	**Yearly trend**	**-0.20**	**1.1E-02**	**-0.35**	**-0.05**
**Greece**	**Cirrhosis and other chronic liver diseases due to hepatitis B**	**Deaths**	**HIV prevalence_P**	**-0.02**	**2.0E-07**	**-0.02**	**-0.01**
**Greece**	**Cirrhosis and other chronic liver diseases due to hepatitis B**	**Incidence**	**(Intercept)**	**3.47**	**4.4E-04**	**1.58**	**5.35**
**Greece**	**Cirrhosis and other chronic liver diseases due to hepatitis B**	**Incidence**	**Age group**	**-0.12**	**1.0E-02**	**-0.21**	**-0.03**
**Greece**	**Cirrhosis and other chronic liver diseases due to hepatitis B**	**Incidence**	**Yearly trend 2010–2019**	**0.22**	**1.0E-02**	**0.05**	**0.38**
Greece	Cirrhosis and other chronic liver diseases due to hepatitis B	Incidence	% GPD 5Lag	-0.01	9.5E-01	-0.28	0.26
**Greece**	**Cirrhosis and other chronic liver diseases due to hepatitis B**	**Incidence**	**Sex_Male**	**-0.29**	**2.8E-02**	**-0.55**	**-0.03**
**Greece**	**Cirrhosis and other chronic liver diseases due to hepatitis B**	**Incidence**	**Yearly trend**	**-0.29**	**7.8E-05**	**-0.43**	**-0.15**
**Greece**	**Cirrhosis and other chronic liver diseases due to hepatitis B**	**Incidence**	**HIV prevalence_P**	**0.06**	**8.3E-45**	**0.06**	**0.07**
Greece	Cirrhosis and other chronic liver diseases due to hepatitis B	Prevalence	(Intercept)	-129.98	6.3E-01	-661.06	401.09
**Greece**	**Cirrhosis and other chronic liver diseases due to hepatitis B**	**Prevalence**	**Age group**	**596.48**	**1.4E-87**	**571.06**	**621.89**
Greece	Cirrhosis and other chronic liver diseases due to hepatitis B	Prevalence	Yearly trend 2010–2019	29.96	2.0E-01	-15.57	75.50
Greece	Cirrhosis and other chronic liver diseases due to hepatitis B	Prevalence	% GPD 5Lag	-8.33	8.3E-01	-83.51	66.86
**Greece**	**Cirrhosis and other chronic liver diseases due to hepatitis B**	**Prevalence**	**Sex_Male**	**223.24**	**1.5E-08**	**150.31**	**296.16**
**Greece**	**Cirrhosis and other chronic liver diseases due to hepatitis B**	**Prevalence**	**Yearly trend**	**-55.81**	**6.2E-03**	**-95.15**	**-16.47**
**Greece**	**Cirrhosis and other chronic liver diseases due to hepatitis B**	**Prevalence**	**HIV prevalence_P**	**17.86**	**1.9E-46**	**16.23**	**19.50**
Greece	Cirrhosis and other chronic liver diseases due to hepatitis B	YLDs (Years Lived with Disability)	(Intercept)	0.34	2.9E-01	-0.29	0.96
**Greece**	**Cirrhosis and other chronic liver diseases due to hepatitis B**	**YLDs (Years Lived with Disability)**	**Age group**	**0.27**	**1.3E-38**	**0.24**	**0.30**
**Greece**	**Cirrhosis and other chronic liver diseases due to hepatitis B**	**YLDs (Years Lived with Disability)**	**Yearly trend 2010–2019**	**0.08**	**5.6E-03**	**0.02**	**0.13**
Greece	Cirrhosis and other chronic liver diseases due to hepatitis B	YLDs (Years Lived with Disability)	% GPD 5Lag	-0.01	9.1E-01	-0.09	0.08
**Greece**	**Cirrhosis and other chronic liver diseases due to hepatitis B**	**YLDs (Years Lived with Disability)**	**Sex_Male**	**0.27**	**9.0E-09**	**0.18**	**0.35**
**Greece**	**Cirrhosis and other chronic liver diseases due to hepatitis B**	**YLDs (Years Lived with Disability)**	**Yearly trend**	**-0.09**	**3.9E-04**	**-0.13**	**-0.04**
Greece	Cirrhosis and other chronic liver diseases due to hepatitis B	YLDs (Years Lived with Disability)	HIV prevalence_P	0.00	1.0E-01	0.00	0.00
Greece	Cirrhosis and other chronic liver diseases due to hepatitis B	YLLs (Years of Life Lost)	(Intercept)	10.88	5.8E-01	-27.55	49.32
**Greece**	**Cirrhosis and other chronic liver diseases due to hepatitis B**	**YLLs (Years of Life Lost)**	**Age group**	**13.18**	**1.1E-28**	**11.34**	**15.02**
**Greece**	**Cirrhosis and other chronic liver diseases due to hepatitis B**	**YLLs (Years of Life Lost)**	**Yearly trend 2010–2019**	**3.73**	**2.8E-02**	**0.43**	**7.02**
Greece	Cirrhosis and other chronic liver diseases due to hepatitis B	YLLs (Years of Life Lost)	% GPD 5Lag	-0.31	9.1E-01	-5.75	5.13
**Greece**	**Cirrhosis and other chronic liver diseases due to hepatitis B**	**YLLs (Years of Life Lost)**	**Sex_Male**	**20.86**	**1.6E-12**	**15.59**	**26.14**
**Greece**	**Cirrhosis and other chronic liver diseases due to hepatitis B**	**YLLs (Years of Life Lost)**	**Yearly trend**	**-4.06**	**5.9E-03**	**-6.91**	**-1.21**
Greece	Cirrhosis and other chronic liver diseases due to hepatitis B	YLLs (Years of Life Lost)	HIV prevalence_P	0.07	2.6E-01	-0.05	0.19
Greece	Cirrhosis and other chronic liver diseases due to hepatitis C	DALYs (Disability-Adjusted Life Years)	(Intercept)	-55.56	1.0E-01	-121.87	10.75
**Greece**	**Cirrhosis and other chronic liver diseases due to hepatitis C**	**DALYs (Disability-Adjusted Life Years)**	**Age group**	**27.89**	**1.1E-36**	**24.72**	**31.06**
Greece	Cirrhosis and other chronic liver diseases due to hepatitis C	DALYs (Disability-Adjusted Life Years)	Yearly trend 2010–2019	-0.32	9.1E-01	-6.01	5.37
Greece	Cirrhosis and other chronic liver diseases due to hepatitis C	DALYs (Disability-Adjusted Life Years)	% GPD 5Lag	-0.76	8.7E-01	-10.15	8.63
**Greece**	**Cirrhosis and other chronic liver diseases due to hepatitis C**	**DALYs (Disability-Adjusted Life Years)**	**Sex_Male**	**39.70**	**1.7E-14**	**30.60**	**48.81**
Greece	Cirrhosis and other chronic liver diseases due to hepatitis C	DALYs (Disability-Adjusted Life Years)	Yearly trend	0.00	1.0E + 00	-4.92	4.91
Greece	Cirrhosis and other chronic liver diseases due to hepatitis C	DALYs (Disability-Adjusted Life Years)	HIV prevalence_P	0.12	2.5E-01	-0.08	0.32
**Greece**	**Cirrhosis and other chronic liver diseases due to hepatitis C**	**Deaths**	**(Intercept)**	**-4.00**	**2.4E-02**	**-7.44**	**-0.57**
**Greece**	**Cirrhosis and other chronic liver diseases due to hepatitis C**	**Deaths**	**Age group**	**1.97**	**5.4E-51**	**1.81**	**2.14**
Greece	Cirrhosis and other chronic liver diseases due to hepatitis C	Deaths	Yearly trend 2010–2019	0.01	9.2E-01	-0.28	0.31
Greece	Cirrhosis and other chronic liver diseases due to hepatitis C	Deaths	% GPD 5Lag	-0.06	8.2E-01	-0.54	0.43
**Greece**	**Cirrhosis and other chronic liver diseases due to hepatitis C**	**Deaths**	**Sex_Male**	**2.24**	**2.1E-16**	**1.77**	**2.71**
Greece	Cirrhosis and other chronic liver diseases due to hepatitis C	Deaths	Yearly trend	0.02	8.6E-01	-0.23	0.28
**Greece**	**Cirrhosis and other chronic liver diseases due to hepatitis C**	**Deaths**	**HIV prevalence_P**	**-0.04**	**1.2E-11**	**-0.05**	**-0.03**
**Greece**	**Cirrhosis and other chronic liver diseases due to hepatitis C**	**Incidence**	**(Intercept)**	**2.40**	**2.9E-02**	**0.26**	**4.54**
**Greece**	**Cirrhosis and other chronic liver diseases due to hepatitis C**	**Incidence**	**Age group**	**-0.29**	**1.7E-07**	**-0.39**	**-0.18**
Greece	Cirrhosis and other chronic liver diseases due to hepatitis C	Incidence	Yearly trend 2010–2019	-0.02	8.4E-01	-0.20	0.16
Greece	Cirrhosis and other chronic liver diseases due to hepatitis C	Incidence	% GPD 5Lag	-0.01	9.6E-01	-0.31	0.30
**Greece**	**Cirrhosis and other chronic liver diseases due to hepatitis C**	**Incidence**	**Sex_Male**	**-0.70**	**6.9E-06**	**-0.99**	**-0.41**
Greece	Cirrhosis and other chronic liver diseases due to hepatitis C	Incidence	Yearly trend	-0.11	1.8E-01	-0.27	0.05
**Greece**	**Cirrhosis and other chronic liver diseases due to hepatitis C**	**Incidence**	**HIV prevalence_P**	**0.12**	**5.4E-72**	**0.11**	**0.12**
**Greece**	**Cirrhosis and other chronic liver diseases due to hepatitis C**	**Prevalence**	**(Intercept)**	**-443.50**	**2.7E-03**	**-728.27**	**-158.73**
**Greece**	**Cirrhosis and other chronic liver diseases due to hepatitis C**	**Prevalence**	**Age group**	**339.71**	**4.2E-91**	**326.08**	**353.34**
Greece	Cirrhosis and other chronic liver diseases due to hepatitis C	Prevalence	Yearly trend 2010–2019	2.39	8.5E-01	-22.03	26.81
Greece	Cirrhosis and other chronic liver diseases due to hepatitis C	Prevalence	% GPD 5Lag	-1.89	9.3E-01	-42.21	38.42
Greece	Cirrhosis and other chronic liver diseases due to hepatitis C	Prevalence	Sex_Male	-1.10	9.6E-01	-40.20	38.00
Greece	Cirrhosis and other chronic liver diseases due to hepatitis C	Prevalence	Yearly trend	3.97	7.1E-01	-17.12	25.07
**Greece**	**Cirrhosis and other chronic liver diseases due to hepatitis C**	**Prevalence**	**HIV prevalence_P**	**-4.61**	**5.8E-19**	**-5.49**	**-3.73**
**Greece**	**Cirrhosis and other chronic liver diseases due to hepatitis C**	**YLDs (Years Lived with Disability)**	**(Intercept)**	**-0.74**	**4.5E-02**	**-1.46**	**-0.02**
**Greece**	**Cirrhosis and other chronic liver diseases due to hepatitis C**	**YLDs (Years Lived with Disability)**	**Age group**	**0.43**	**3.1E-53**	**0.40**	**0.47**
Greece	Cirrhosis and other chronic liver diseases due to hepatitis C	YLDs (Years Lived with Disability)	Yearly trend 2010–2019	-0.01	8.1E-01	-0.07	0.05
Greece	Cirrhosis and other chronic liver diseases due to hepatitis C	YLDs (Years Lived with Disability)	% GPD 5Lag	0.00	9.2E-01	-0.11	0.10
**Greece**	**Cirrhosis and other chronic liver diseases due to hepatitis C**	**YLDs (Years Lived with Disability)**	**Sex_Male**	**0.35**	**1.7E-10**	**0.25**	**0.45**
Greece	Cirrhosis and other chronic liver diseases due to hepatitis C	YLDs (Years Lived with Disability)	Yearly trend	0.00	9.8E-01	-0.05	0.05
**Greece**	**Cirrhosis and other chronic liver diseases due to hepatitis C**	**YLDs (Years Lived with Disability)**	**HIV prevalence_P**	**0.00**	**7.7E-04**	**0.00**	**0.01**
Greece	Cirrhosis and other chronic liver diseases due to hepatitis C	YLLs (Years of Life Lost)	(Intercept)	-54.81	1.0E-01	-120.42	10.79
**Greece**	**Cirrhosis and other chronic liver diseases due to hepatitis C**	**YLLs (Years of Life Lost)**	**Age group**	**27.46**	**1.7E-36**	**24.32**	**30.60**
Greece	Cirrhosis and other chronic liver diseases due to hepatitis C	YLLs (Years of Life Lost)	Yearly trend 2010–2019	-0.31	9.1E-01	-5.94	5.31
Greece	Cirrhosis and other chronic liver diseases due to hepatitis C	YLLs (Years of Life Lost)	% GPD 5Lag	-0.76	8.7E-01	-10.05	8.53
**Greece**	**Cirrhosis and other chronic liver diseases due to hepatitis C**	**YLLs (Years of Life Lost)**	**Sex_Male**	**39.36**	**1.6E-14**	**30.35**	**48.37**
Greece	Cirrhosis and other chronic liver diseases due to hepatitis C	YLLs (Years of Life Lost)	Yearly trend	0.00	1.0E + 00	-4.86	4.86
Greece	Cirrhosis and other chronic liver diseases due to hepatitis C	YLLs (Years of Life Lost)	HIV prevalence_P	0.12	2.6E-01	-0.09	0.32
**Greece**	**Liver cancer due to hepatitis B**	**DALYs (Disability-Adjusted Life Years)**	**(Intercept)**	**-111.65**	**1.8E-02**	**-203.39**	**-19.91**
**Greece**	**Liver cancer due to hepatitis B**	**DALYs (Disability-Adjusted Life Years)**	**Age group**	**33.88**	**1.8E-31**	**29.49**	**38.27**
Greece	Liver cancer due to hepatitis B	DALYs (Disability-Adjusted Life Years)	Yearly trend 2010–2019	0.49	9.0E-01	-7.37	8.36
Greece	Liver cancer due to hepatitis B	DALYs (Disability-Adjusted Life Years)	% GPD 5Lag	2.82	6.7E-01	-10.17	15.81
**Greece**	**Liver cancer due to hepatitis B**	**DALYs (Disability-Adjusted Life Years)**	**Sex_Male**	**55.17**	**1.4E-14**	**42.57**	**67.77**
Greece	Liver cancer due to hepatitis B	DALYs (Disability-Adjusted Life Years)	Yearly trend	0.49	8.9E-01	-6.31	7.28
Greece	Liver cancer due to hepatitis B	DALYs (Disability-Adjusted Life Years)	HIV prevalence_P	-0.24	1.0E-01	-0.52	0.04
**Greece**	**Liver cancer due to hepatitis B**	**Deaths**	**(Intercept)**	**-10.38**	**8.3E-05**	**-15.37**	**-5.40**
**Greece**	**Liver cancer due to hepatitis B**	**Deaths**	**Age group**	**2.78**	**2.5E-37**	**2.50**	**3.07**
Greece	Liver cancer due to hepatitis B	Deaths	Yearly trend 2010–2019	0.03	9.0E-01	-0.39	0.45
Greece	Liver cancer due to hepatitis B	Deaths	% GPD 5Lag	0.18	6.2E-01	-0.52	0.88
**Greece**	**Liver cancer due to hepatitis B**	**Deaths**	**Sex_Male**	**4.02**	**2.2E-20**	**3.33**	**4.72**
Greece	Liver cancer due to hepatitis B	Deaths	Yearly trend	0.08	6.6E-01	-0.28	0.45
**Greece**	**Liver cancer due to hepatitis B**	**Deaths**	**HIV prevalence_P**	**-0.05**	**3.3E-09**	**-0.06**	**-0.03**
**Greece**	**Liver cancer due to hepatitis B**	**Incidence**	**(Intercept)**	**-6.90**	**5.8E-03**	**-11.73**	**-2.07**
**Greece**	**Liver cancer due to hepatitis B**	**Incidence**	**Age group**	**2.14**	**6.2E-39**	**1.91**	**2.37**
Greece	Liver cancer due to hepatitis B	Incidence	Yearly trend 2010–2019	0.03	9.0E-01	-0.39	0.44
Greece	Liver cancer due to hepatitis B	Incidence	% GPD 5Lag	0.15	6.7E-01	-0.53	0.83
**Greece**	**Liver cancer due to hepatitis B**	**Incidence**	**Sex_Male**	**3.26**	**3.1E-17**	**2.60**	**3.92**
Greece	Liver cancer due to hepatitis B	Incidence	Yearly trend	0.05	7.8E-01	-0.31	0.41
**Greece**	**Liver cancer due to hepatitis B**	**Incidence**	**HIV prevalence_P**	**-0.04**	**6.6E-07**	**-0.05**	**-0.02**
**Greece**	**Liver cancer due to hepatitis B**	**Prevalence**	**(Intercept)**	**-7.87**	**1.0E-02**	**-13.82**	**-1.93**
**Greece**	**Liver cancer due to hepatitis B**	**Prevalence**	**Age group**	**2.37**	**1.7E-34**	**2.09**	**2.66**
Greece	Liver cancer due to hepatitis B	Prevalence	Yearly trend 2010–2019	0.01	9.8E-01	-0.50	0.52
Greece	Liver cancer due to hepatitis B	Prevalence	% GPD 5Lag	0.19	6.6E-01	-0.65	1.03
**Greece**	**Liver cancer due to hepatitis B**	**Prevalence**	**Sex_Male**	**3.80**	**6.5E-16**	**2.98**	**4.61**
Greece	Liver cancer due to hepatitis B	Prevalence	Yearly trend	0.06	7.9E-01	-0.38	0.50
**Greece**	**Liver cancer due to hepatitis B**	**Prevalence**	**HIV prevalence_P**	**-0.02**	**3.1E-02**	**-0.04**	**0.00**
**Greece**	**Liver cancer due to hepatitis B**	**YLDs (Years Lived with Disability)**	**(Intercept)**	**-1.61**	**6.2E-03**	**-2.75**	**-0.47**
**Greece**	**Liver cancer due to hepatitis B**	**YLDs (Years Lived with Disability)**	**Age group**	**0.50**	**2.7E-38**	**0.44**	**0.55**
Greece	Liver cancer due to hepatitis B	YLDs (Years Lived with Disability)	Yearly trend 2010–2019	0.00	9.2E-01	-0.09	0.10
Greece	Liver cancer due to hepatitis B	YLDs (Years Lived with Disability)	% GPD 5Lag	0.04	6.7E-01	-0.13	0.20
**Greece**	**Liver cancer due to hepatitis B**	**YLDs (Years Lived with Disability)**	**Sex_Male**	**0.76**	**3.8E-17**	**0.61**	**0.92**
Greece	Liver cancer due to hepatitis B	YLDs (Years Lived with Disability)	Yearly trend	0.01	7.8E-01	-0.07	0.10
**Greece**	**Liver cancer due to hepatitis B**	**YLDs (Years Lived with Disability)**	**HIV prevalence_P**	**-0.01**	**9.5E-06**	**-0.01**	**0.00**
**Greece**	**Liver cancer due to hepatitis B**	**YLLs (Years of Life Lost)**	**(Intercept)**	**-160.35**	**1.7E-03**	**-258.21**	**-62.48**
**Greece**	**Liver cancer due to hepatitis B**	**YLLs (Years of Life Lost)**	**Age group**	**42.34**	**2.5E-28**	**36.75**	**47.93**
Greece	Liver cancer due to hepatitis B	YLLs (Years of Life Lost)	Yearly trend 2010–2019	0.61	8.9E-01	-7.70	8.92
Greece	Liver cancer due to hepatitis B	YLLs (Years of Life Lost)	% GPD 5Lag	3.47	6.2E-01	-10.25	17.19
**Greece**	**Liver cancer due to hepatitis B**	**YLLs (Years of Life Lost)**	**Sex_Male**	**67.06**	**2.2E-16**	**53.41**	**80.70**
Greece	Liver cancer due to hepatitis B	YLLs (Years of Life Lost)	Yearly trend	0.53	8.9E-01	-6.65	7.71
Greece	Liver cancer due to hepatitis B	YLLs (Years of Life Lost)	HIV prevalence_P	-0.18	2.2E-01	-0.47	0.10
**Greece**	**Liver cancer due to hepatitis C**	**DALYs (Disability-Adjusted Life Years)**	**(Intercept)**	**-57.50**	**2.6E-03**	**-94.28**	**-20.73**
**Greece**	**Liver cancer due to hepatitis C**	**DALYs (Disability-Adjusted Life Years)**	**Age group**	**22.44**	**4.9E-54**	**20.68**	**24.20**
Greece	Liver cancer due to hepatitis C	DALYs (Disability-Adjusted Life Years)	Yearly trend 2010–2019	-0.25	8.8E-01	-3.41	2.90
Greece	Liver cancer due to hepatitis C	DALYs (Disability-Adjusted Life Years)	% GPD 5Lag	1.04	7.0E-01	-4.17	6.24
Greece	Liver cancer due to hepatitis C	DALYs (Disability-Adjusted Life Years)	Sex_Male	1.35	6.0E-01	-3.70	6.40
Greece	Liver cancer due to hepatitis C	DALYs (Disability-Adjusted Life Years)	Yearly trend	1.18	4.0E-01	-1.54	3.90
**Greece**	**Liver cancer due to hepatitis C**	**DALYs (Disability-Adjusted Life Years)**	**HIV prevalence_P**	**-0.61**	**1.5E-19**	**-0.72**	**-0.50**
**Greece**	**Liver cancer due to hepatitis C**	**Deaths**	**(Intercept)**	**-6.64**	**2.3E-03**	**-10.81**	**-2.48**
**Greece**	**Liver cancer due to hepatitis C**	**Deaths**	**Age group**	**2.18**	**7.2E-35**	**1.94**	**2.42**
Greece	Liver cancer due to hepatitis C	Deaths	Yearly trend 2010–2019	-0.01	9.5E-01	-0.37	0.34
Greece	Liver cancer due to hepatitis C	Deaths	% GPD 5Lag	0.08	7.9E-01	-0.51	0.66
Greece	Liver cancer due to hepatitis C	Deaths	Sex_Male	-0.01	9.7E-01	-0.59	0.57
Greece	Liver cancer due to hepatitis C	Deaths	Yearly trend	0.11	4.8E-01	-0.20	0.42
**Greece**	**Liver cancer due to hepatitis C**	**Deaths**	**HIV prevalence_P**	**-0.05**	**1.7E-11**	**-0.06**	**-0.03**
**Greece**	**Liver cancer due to hepatitis C**	**Incidence**	**(Intercept)**	**-4.03**	**1.4E-02**	**-7.21**	**-0.85**
**Greece**	**Liver cancer due to hepatitis C**	**Incidence**	**Age group**	**1.59**	**2.1E-44**	**1.44**	**1.74**
Greece	Liver cancer due to hepatitis C	Incidence	Yearly trend 2010–2019	-0.01	9.2E-01	-0.29	0.26
Greece	Liver cancer due to hepatitis C	Incidence	% GPD 5Lag	0.06	8.0E-01	-0.39	0.51
Greece	Liver cancer due to hepatitis C	Incidence	Sex_Male	0.11	6.2E-01	-0.33	0.55
Greece	Liver cancer due to hepatitis C	Incidence	Yearly trend	0.09	4.6E-01	-0.15	0.32
**Greece**	**Liver cancer due to hepatitis C**	**Incidence**	**HIV prevalence_P**	**-0.05**	**1.7E-17**	**-0.06**	**-0.04**
**Greece**	**Liver cancer due to hepatitis C**	**Prevalence**	**(Intercept)**	**-4.27**	**4.7E-03**	**-7.18**	**-1.36**
**Greece**	**Liver cancer due to hepatitis C**	**Prevalence**	**Age group**	**1.65**	**2.3E-50**	**1.51**	**1.79**
Greece	Liver cancer due to hepatitis C	Prevalence	Yearly trend 2010–2019	-0.03	8.0E-01	-0.28	0.22
Greece	Liver cancer due to hepatitis C	Prevalence	% GPD 5Lag	0.07	7.5E-01	-0.35	0.48
Greece	Liver cancer due to hepatitis C	Prevalence	Sex_Male	0.09	6.6E-01	-0.31	0.49
Greece	Liver cancer due to hepatitis C	Prevalence	Yearly trend	0.10	3.6E-01	-0.12	0.32
**Greece**	**Liver cancer due to hepatitis C**	**Prevalence**	**HIV prevalence_P**	**-0.05**	**1.7E-18**	**-0.06**	**-0.04**
**Greece**	**Liver cancer due to hepatitis C**	**YLDs (Years Lived with Disability)**	**(Intercept)**	**-0.93**	**1.2E-02**	**-1.64**	**-0.22**
**Greece**	**Liver cancer due to hepatitis C**	**YLDs (Years Lived with Disability)**	**Age group**	**0.36**	**1.8E-45**	**0.33**	**0.40**
Greece	Liver cancer due to hepatitis C	YLDs (Years Lived with Disability)	Yearly trend 2010–2019	0.00	9.0E-01	-0.06	0.06
Greece	Liver cancer due to hepatitis C	YLDs (Years Lived with Disability)	% GPD 5Lag	0.01	7.9E-01	-0.09	0.11
Greece	Liver cancer due to hepatitis C	YLDs (Years Lived with Disability)	Sex_Male	0.03	6.1E-01	-0.07	0.12
Greece	Liver cancer due to hepatitis C	YLDs (Years Lived with Disability)	Yearly trend	0.02	4.4E-01	-0.03	0.07
**Greece**	**Liver cancer due to hepatitis C**	**YLDs (Years Lived with Disability)**	**HIV prevalence_P**	**-0.01**	**1.1E-17**	**-0.01**	**-0.01**
**Greece**	**Liver cancer due to hepatitis C**	**YLLs (Years of Life Lost)**	**(Intercept)**	**-84.40**	**1.4E-05**	**-120.84**	**-47.97**
**Greece**	**Liver cancer due to hepatitis C**	**YLLs (Years of Life Lost)**	**Age group**	**27.69**	**1.2E-49**	**25.61**	**29.77**
Greece	Liver cancer due to hepatitis C	YLLs (Years of Life Lost)	Yearly trend 2010–2019	-0.32	8.4E-01	-3.41	2.78
Greece	Liver cancer due to hepatitis C	YLLs (Years of Life Lost)	% GPD 5Lag	1.25	6.3E-01	-3.86	6.36
Greece	Liver cancer due to hepatitis C	YLLs (Years of Life Lost)	Sex_Male	-0.47	8.6E-01	-5.55	4.61
Greece	Liver cancer due to hepatitis C	YLLs (Years of Life Lost)	Yearly trend	1.31	3.4E-01	-1.36	3.99
**Greece**	**Liver cancer due to hepatitis C**	**YLLs (Years of Life Lost)**	**HIV prevalence_P**	**-0.49**	**6.7E-15**	**-0.59**	**-0.38**
Italy	Cirrhosis and other chronic liver diseases due to hepatitis B	DALYs (Disability-Adjusted Life Years)	(Intercept)	-9.74	9.0E-01	-165.52	146.04
**Italy**	**Cirrhosis and other chronic liver diseases due to hepatitis B**	**DALYs (Disability-Adjusted Life Years)**	**Age group**	**26.29**	**1.4E-41**	**23.61**	**28.96**
Italy	Cirrhosis and other chronic liver diseases due to hepatitis B	DALYs (Disability-Adjusted Life Years)	Yearly trend 2010–2019	2.07	3.9E-01	-2.68	6.83
Italy	Cirrhosis and other chronic liver diseases due to hepatitis B	DALYs (Disability-Adjusted Life Years)	% GPD 5Lag	-2.68	8.2E-01	-25.95	20.59
**Italy**	**Cirrhosis and other chronic liver diseases due to hepatitis B**	**DALYs (Disability-Adjusted Life Years)**	**Sex_Male**	**37.14**	**1.4E-17**	**29.68**	**44.59**
Italy	Cirrhosis and other chronic liver diseases due to hepatitis B	DALYs (Disability-Adjusted Life Years)	Yearly trend	-2.65	3.2E-01	-7.83	2.53
Italy	Cirrhosis and other chronic liver diseases due to hepatitis B	DALYs (Disability-Adjusted Life Years)	HIV prevalence_P	-0.01	6.3E-01	-0.05	0.03
Italy	Cirrhosis and other chronic liver diseases due to hepatitis B	Deaths	(Intercept)	-1.77	7.1E-01	-11.15	7.62
**Italy**	**Cirrhosis and other chronic liver diseases due to hepatitis B**	**Deaths**	**Age group**	**1.83**	**2.3E-48**	**1.67**	**1.99**
Italy	Cirrhosis and other chronic liver diseases due to hepatitis B	Deaths	Yearly trend 2010–2019	0.11	4.6E-01	-0.18	0.40
Italy	Cirrhosis and other chronic liver diseases due to hepatitis B	Deaths	% GPD 5Lag	-0.14	8.5E-01	-1.54	1.26
**Italy**	**Cirrhosis and other chronic liver diseases due to hepatitis B**	**Deaths**	**Sex_Male**	**2.04**	**2.3E-15**	**1.59**	**2.49**
Italy	Cirrhosis and other chronic liver diseases due to hepatitis B	Deaths	Yearly trend	-0.10	5.5E-01	-0.41	0.22
**Italy**	**Cirrhosis and other chronic liver diseases due to hepatitis B**	**Deaths**	**HIV prevalence_P**	**-0.01**	**4.6E-14**	**-0.01**	**-0.01**
Italy	Cirrhosis and other chronic liver diseases due to hepatitis B	Incidence	(Intercept)	2.97	7.3E-01	-13.93	19.88
Italy	Cirrhosis and other chronic liver diseases due to hepatitis B	Incidence	Age group	-0.17	2.5E-01	-0.46	0.12
Italy	Cirrhosis and other chronic liver diseases due to hepatitis B	Incidence	Yearly trend 2010–2019	0.04	8.8E-01	-0.48	0.55
Italy	Cirrhosis and other chronic liver diseases due to hepatitis B	Incidence	% GPD 5Lag	-0.02	9.8E-01	-2.55	2.50
Italy	Cirrhosis and other chronic liver diseases due to hepatitis B	Incidence	Sex_Male	0.17	6.9E-01	-0.64	0.98
Italy	Cirrhosis and other chronic liver diseases due to hepatitis B	Incidence	Yearly trend	-0.18	5.3E-01	-0.74	0.38
**Italy**	**Cirrhosis and other chronic liver diseases due to hepatitis B**	**Incidence**	**HIV prevalence_P**	**0.03**	**6.8E-28**	**0.03**	**0.04**
Italy	Cirrhosis and other chronic liver diseases due to hepatitis B	Prevalence	(Intercept)	-92.74	8.3E-01	-937.68	752.20
**Italy**	**Cirrhosis and other chronic liver diseases due to hepatitis B**	**Prevalence**	**Age group**	**261.51**	**1.6E-72**	**247.00**	**276.02**
Italy	Cirrhosis and other chronic liver diseases due to hepatitis B	Prevalence	Yearly trend 2010–2019	0.21	9.9E-01	-25.57	26.00
Italy	Cirrhosis and other chronic liver diseases due to hepatitis B	Prevalence	% GPD 5Lag	-5.15	9.4E-01	-131.35	121.05
**Italy**	**Cirrhosis and other chronic liver diseases due to hepatitis B**	**Prevalence**	**Sex_Male**	**60.90**	**3.7E-03**	**20.46**	**101.34**
Italy	Cirrhosis and other chronic liver diseases due to hepatitis B	Prevalence	Yearly trend	-17.11	2.3E-01	-45.21	10.99
**Italy**	**Cirrhosis and other chronic liver diseases due to hepatitis B**	**Prevalence**	**HIV prevalence_P**	**2.00**	**8.9E-38**	**1.77**	**2.22**
Italy	Cirrhosis and other chronic liver diseases due to hepatitis B	YLDs (Years Lived with Disability)	(Intercept)	-0.51	7.2E-01	-3.25	2.24
**Italy**	**Cirrhosis and other chronic liver diseases due to hepatitis B**	**YLDs (Years Lived with Disability)**	**Age group**	**0.62**	**1.2E-55**	**0.57**	**0.67**
Italy	Cirrhosis and other chronic liver diseases due to hepatitis B	YLDs (Years Lived with Disability)	Yearly trend 2010–2019	0.02	6.2E-01	-0.06	0.11
Italy	Cirrhosis and other chronic liver diseases due to hepatitis B	YLDs (Years Lived with Disability)	% GPD 5Lag	-0.05	8.2E-01	-0.46	0.36
**Italy**	**Cirrhosis and other chronic liver diseases due to hepatitis B**	**YLDs (Years Lived with Disability)**	**Sex_Male**	**0.61**	**7.3E-16**	**0.48**	**0.74**
Italy	Cirrhosis and other chronic liver diseases due to hepatitis B	YLDs (Years Lived with Disability)	Yearly trend	-0.03	4.9E-01	-0.12	0.06
Italy	Cirrhosis and other chronic liver diseases due to hepatitis B	YLDs (Years Lived with Disability)	HIV prevalence_P	0.00	1.9E-01	0.00	0.00
Italy	Cirrhosis and other chronic liver diseases due to hepatitis B	YLLs (Years of Life Lost)	(Intercept)	-9.23	9.1E-01	-162.33	143.87
**Italy**	**Cirrhosis and other chronic liver diseases due to hepatitis B**	**YLLs (Years of Life Lost)**	**Age group**	**25.67**	**2.8E-41**	**23.04**	**28.30**
Italy	Cirrhosis and other chronic liver diseases due to hepatitis B	YLLs (Years of Life Lost)	Yearly trend 2010–2019	2.05	3.9E-01	-2.62	6.72
Italy	Cirrhosis and other chronic liver diseases due to hepatitis B	YLLs (Years of Life Lost)	% GPD 5Lag	-2.63	8.2E-01	-25.50	20.23
**Italy**	**Cirrhosis and other chronic liver diseases due to hepatitis B**	**YLLs (Years of Life Lost)**	**Sex_Male**	**36.53**	**1.4E-17**	**29.20**	**43.85**
Italy	Cirrhosis and other chronic liver diseases due to hepatitis B	YLLs (Years of Life Lost)	Yearly trend	-2.62	3.1E-01	-7.71	2.47
Italy	Cirrhosis and other chronic liver diseases due to hepatitis B	YLLs (Years of Life Lost)	HIV prevalence_P	-0.01	6.4E-01	-0.05	0.03
Italy	Cirrhosis and other chronic liver diseases due to hepatitis C	DALYs (Disability-Adjusted Life Years)	(Intercept)	-102.68	6.9E-01	-606.92	401.56
**Italy**	**Cirrhosis and other chronic liver diseases due to hepatitis C**	**DALYs (Disability-Adjusted Life Years)**	**Age group**	**99.99**	**4.0E-49**	**91.33**	**108.64**
Italy	Cirrhosis and other chronic liver diseases due to hepatitis C	DALYs (Disability-Adjusted Life Years)	Yearly trend 2010–2019	1.09	8.9E-01	-14.30	16.48
Italy	Cirrhosis and other chronic liver diseases due to hepatitis C	DALYs (Disability-Adjusted Life Years)	% GPD 5Lag	-9.20	8.1E-01	-84.51	66.12
**Italy**	**Cirrhosis and other chronic liver diseases due to hepatitis C**	**DALYs (Disability-Adjusted Life Years)**	**Sex_Male**	**113.83**	**3.0E-16**	**89.70**	**137.97**
Italy	Cirrhosis and other chronic liver diseases due to hepatitis C	DALYs (Disability-Adjusted Life Years)	Yearly trend	-2.72	7.5E-01	-19.49	14.05
Italy	Cirrhosis and other chronic liver diseases due to hepatitis C	DALYs (Disability-Adjusted Life Years)	HIV prevalence_P	-0.13	5.3E-02	-0.26	0.00
Italy	Cirrhosis and other chronic liver diseases due to hepatitis C	Deaths	(Intercept)	-9.87	5.5E-01	-41.93	22.19
**Italy**	**Cirrhosis and other chronic liver diseases due to hepatitis C**	**Deaths**	**Age group**	**7.12**	**8.5E-55**	**6.57**	**7.67**
Italy	Cirrhosis and other chronic liver diseases due to hepatitis C	Deaths	Yearly trend 2010–2019	0.10	8.4E-01	-0.88	1.08
Italy	Cirrhosis and other chronic liver diseases due to hepatitis C	Deaths	% GPD 5Lag	-0.49	8.4E-01	-5.27	4.30
**Italy**	**Cirrhosis and other chronic liver diseases due to hepatitis C**	**Deaths**	**Sex_Male**	**6.36**	**1.9E-13**	**4.83**	**7.89**
Italy	Cirrhosis and other chronic liver diseases due to hepatitis C	Deaths	Yearly trend	-0.03	9.6E-01	-1.09	1.04
**Italy**	**Cirrhosis and other chronic liver diseases due to hepatitis C**	**Deaths**	**HIV prevalence_P**	**-0.04**	**1.4E-18**	**-0.05**	**-0.04**
Italy	Cirrhosis and other chronic liver diseases due to hepatitis C	Incidence	(Intercept)	8.81	7.7E-01	-49.82	67.44
Italy	Cirrhosis and other chronic liver diseases due to hepatitis C	Incidence	Age group	-0.44	4.0E-01	-1.44	0.57
Italy	Cirrhosis and other chronic liver diseases due to hepatitis C	Incidence	Yearly trend 2010–2019	-0.10	9.1E-01	-1.89	1.69
Italy	Cirrhosis and other chronic liver diseases due to hepatitis C	Incidence	% GPD 5Lag	-0.10	9.8E-01	-8.85	8.66
Italy	Cirrhosis and other chronic liver diseases due to hepatitis C	Incidence	Sex_Male	-0.45	7.5E-01	-3.26	2.35
Italy	Cirrhosis and other chronic liver diseases due to hepatitis C	Incidence	Yearly trend	-0.40	6.9E-01	-2.35	1.55
**Italy**	**Cirrhosis and other chronic liver diseases due to hepatitis C**	**Incidence**	**HIV prevalence_P**	**0.11**	**3.8E-29**	**0.10**	**0.13**
Italy	Cirrhosis and other chronic liver diseases due to hepatitis C	Prevalence	(Intercept)	312.29	7.7E-01	-1739.31	2363.89
**Italy**	**Cirrhosis and other chronic liver diseases due to hepatitis C**	**Prevalence**	**Age group**	**972.41**	**4.0E-97**	**937.19**	**1007.64**
Italy	Cirrhosis and other chronic liver diseases due to hepatitis C	Prevalence	Yearly trend 2010–2019	-20.23	5.3E-01	-82.84	42.38
Italy	Cirrhosis and other chronic liver diseases due to hepatitis C	Prevalence	% GPD 5Lag	-175.57	2.6E-01	-482.00	130.85
Italy	Cirrhosis and other chronic liver diseases due to hepatitis C	Prevalence	Sex_Male	-56.15	2.6E-01	-154.34	42.04
Italy	Cirrhosis and other chronic liver diseases due to hepatitis C	Prevalence	Yearly trend	13.46	7.0E-01	-54.78	81.69
**Italy**	**Cirrhosis and other chronic liver diseases due to hepatitis C**	**Prevalence**	**HIV prevalence_P**	**-2.31**	**3.4E-14**	**-2.85**	**-1.77**
Italy	Cirrhosis and other chronic liver diseases due to hepatitis C	YLDs (Years Lived with Disability)	(Intercept)	-3.19	5.5E-01	-13.52	7.14
**Italy**	**Cirrhosis and other chronic liver diseases due to hepatitis C**	**YLDs (Years Lived with Disability)**	**Age group**	**2.59**	**5.6E-61**	**2.41**	**2.76**
Italy	Cirrhosis and other chronic liver diseases due to hepatitis C	YLDs (Years Lived with Disability)	Yearly trend 2010–2019	-0.01	9.7E-01	-0.32	0.31
Italy	Cirrhosis and other chronic liver diseases due to hepatitis C	YLDs (Years Lived with Disability)	% GPD 5Lag	-0.16	8.4E-01	-1.71	1.38
**Italy**	**Cirrhosis and other chronic liver diseases due to hepatitis C**	**YLDs (Years Lived with Disability)**	**Sex_Male**	**2.25**	**1.9E-15**	**1.76**	**2.75**
Italy	Cirrhosis and other chronic liver diseases due to hepatitis C	YLDs (Years Lived with Disability)	Yearly trend	-0.03	8.6E-01	-0.38	0.31
**Italy**	**Cirrhosis and other chronic liver diseases due to hepatitis C**	**YLDs (Years Lived with Disability)**	**HIV prevalence_P**	**0.00**	**5.9E-04**	**-0.01**	**0.00**
Italy	Cirrhosis and other chronic liver diseases due to hepatitis C	YLLs (Years of Life Lost)	(Intercept)	-99.49	6.9E-01	-593.57	394.59
**Italy**	**Cirrhosis and other chronic liver diseases due to hepatitis C**	**YLLs (Years of Life Lost)**	**Age group**	**97.40**	**7.7E-49**	**88.92**	**105.88**
Italy	Cirrhosis and other chronic liver diseases due to hepatitis C	YLLs (Years of Life Lost)	Yearly trend 2010–2019	1.09	8.9E-01	-13.99	16.17
Italy	Cirrhosis and other chronic liver diseases due to hepatitis C	YLLs (Years of Life Lost)	% GPD 5Lag	-9.03	8.1E-01	-82.83	64.76
**Italy**	**Cirrhosis and other chronic liver diseases due to hepatitis C**	**YLLs (Years of Life Lost)**	**Sex_Male**	**111.58**	**3.0E-16**	**87.93**	**135.23**
Italy	Cirrhosis and other chronic liver diseases due to hepatitis C	YLLs (Years of Life Lost)	Yearly trend	-2.69	7.5E-01	-19.12	13.74
Italy	Cirrhosis and other chronic liver diseases due to hepatitis C	YLLs (Years of Life Lost)	HIV prevalence_P	-0.13	5.7E-02	-0.26	0.00
Italy	Liver cancer due to hepatitis B	DALYs (Disability-Adjusted Life Years)	(Intercept)	-30.59	5.9E-01	-140.94	79.76
**Italy**	**Liver cancer due to hepatitis B**	**DALYs (Disability-Adjusted Life Years)**	**Age group**	**13.85**	**2.0E-29**	**11.95**	**15.74**
Italy	Liver cancer due to hepatitis B	DALYs (Disability-Adjusted Life Years)	Yearly trend 2010–2019	0.05	9.8E-01	-3.32	3.42
Italy	Liver cancer due to hepatitis B	DALYs (Disability-Adjusted Life Years)	% GPD 5Lag	-0.17	9.8E-01	-16.65	16.32
**Italy**	**Liver cancer due to hepatitis B**	**DALYs (Disability-Adjusted Life Years)**	**Sex_Male**	**22.71**	**3.4E-14**	**17.43**	**27.99**
Italy	Liver cancer due to hepatitis B	DALYs (Disability-Adjusted Life Years)	Yearly trend	-0.15	9.4E-01	-3.82	3.52
**Italy**	**Liver cancer due to hepatitis B**	**DALYs (Disability-Adjusted Life Years)**	**HIV prevalence_P**	**0.03**	**4.0E-02**	**0.00**	**0.06**
Italy	Liver cancer due to hepatitis B	Deaths	(Intercept)	-3.19	2.4E-01	-8.46	2.09
**Italy**	**Liver cancer due to hepatitis B**	**Deaths**	**Age group**	**1.13**	**1.5E-39**	**1.02**	**1.24**
Italy	Liver cancer due to hepatitis B	Deaths	Yearly trend 2010–2019	0.00	9.6E-01	-0.16	0.16
Italy	Liver cancer due to hepatitis B	Deaths	% GPD 5Lag	-0.01	9.8E-01	-0.80	0.78
**Italy**	**Liver cancer due to hepatitis B**	**Deaths**	**Sex_Male**	**1.46**	**4.4E-20**	**1.21**	**1.72**
Italy	Liver cancer due to hepatitis B	Deaths	Yearly trend	0.01	9.1E-01	-0.17	0.19
**Italy**	**Liver cancer due to hepatitis B**	**Deaths**	**HIV prevalence_P**	**0.00**	**4.4E-05**	**0.00**	**0.00**
Italy	Liver cancer due to hepatitis B	Incidence	(Intercept)	-2.26	4.7E-01	-8.42	3.90
**Italy**	**Liver cancer due to hepatitis B**	**Incidence**	**Age group**	**0.92**	**2.0E-36**	**0.82**	**1.03**
Italy	Liver cancer due to hepatitis B	Incidence	Yearly trend 2010–2019	0.01	9.5E-01	-0.18	0.19
Italy	Liver cancer due to hepatitis B	Incidence	% GPD 5Lag	0.01	9.7E-01	-0.91	0.93
**Italy**	**Liver cancer due to hepatitis B**	**Incidence**	**Sex_Male**	**1.39**	**2.8E-16**	**1.10**	**1.69**
Italy	Liver cancer due to hepatitis B	Incidence	Yearly trend	0.00	9.8E-01	-0.21	0.20
Italy	Liver cancer due to hepatitis B	Incidence	HIV prevalence_P	0.00	2.2E-01	0.00	0.00
Italy	Liver cancer due to hepatitis B	Prevalence	(Intercept)	-3.22	5.5E-01	-13.71	7.28
**Italy**	**Liver cancer due to hepatitis B**	**Prevalence**	**Age group**	**1.16**	**7.2E-25**	**0.98**	**1.34**
Italy	Liver cancer due to hepatitis B	Prevalence	Yearly trend 2010–2019	-0.01	9.5E-01	-0.33	0.31
Italy	Liver cancer due to hepatitis B	Prevalence	% GPD 5Lag	0.05	9.5E-01	-1.52	1.62
**Italy**	**Liver cancer due to hepatitis B**	**Prevalence**	**Sex_Male**	**2.15**	**3.9E-14**	**1.65**	**2.66**
Italy	Liver cancer due to hepatitis B	Prevalence	Yearly trend	-0.01	9.6E-01	-0.36	0.34
**Italy**	**Liver cancer due to hepatitis B**	**Prevalence**	**HIV prevalence_P**	**0.01**	**2.7E-06**	**0.00**	**0.01**
Italy	Liver cancer due to hepatitis B	YLDs (Years Lived with Disability)	(Intercept)	-0.56	4.9E-01	-2.13	1.01
**Italy**	**Liver cancer due to hepatitis B**	**YLDs (Years Lived with Disability)**	**Age group**	**0.22**	**7.3E-34**	**0.19**	**0.25**
Italy	Liver cancer due to hepatitis B	YLDs (Years Lived with Disability)	Yearly trend 2010–2019	0.00	9.8E-01	-0.05	0.05
Italy	Liver cancer due to hepatitis B	YLDs (Years Lived with Disability)	% GPD 5Lag	0.00	9.7E-01	-0.23	0.24
**Italy**	**Liver cancer due to hepatitis B**	**YLDs (Years Lived with Disability)**	**Sex_Male**	**0.35**	**4.6E-16**	**0.28**	**0.43**
Italy	Liver cancer due to hepatitis B	YLDs (Years Lived with Disability)	Yearly trend	0.00	9.8E-01	-0.05	0.05
Italy	Liver cancer due to hepatitis B	YLDs (Years Lived with Disability)	HIV prevalence_P	0.00	6.3E-01	0.00	0.00
Italy	Liver cancer due to hepatitis B	YLLs (Years of Life Lost)	(Intercept)	-48.97	4.1E-01	-164.78	66.85
**Italy**	**Liver cancer due to hepatitis B**	**YLLs (Years of Life Lost)**	**Age group**	**17.79**	**1.1E-27**	**15.40**	**20.19**
Italy	Liver cancer due to hepatitis B	YLLs (Years of Life Lost)	Yearly trend 2010–2019	0.04	9.8E-01	-3.49	3.57
Italy	Liver cancer due to hepatitis B	YLLs (Years of Life Lost)	% GPD 5Lag	-0.21	9.8E-01	-17.48	17.07
**Italy**	**Liver cancer due to hepatitis B**	**YLLs (Years of Life Lost)**	**Sex_Male**	**27.29**	**3.5E-16**	**21.69**	**32.90**
Italy	Liver cancer due to hepatitis B	YLLs (Years of Life Lost)	Yearly trend	-0.22	9.1E-01	-4.07	3.63
**Italy**	**Liver cancer due to hepatitis B**	**YLLs (Years of Life Lost)**	**HIV prevalence_P**	**0.04**	**5.0E-03**	**0.01**	**0.07**
Italy	Liver cancer due to hepatitis C	DALYs (Disability-Adjusted Life Years)	(Intercept)	-254.58	3.4E-01	-775.64	266.48
**Italy**	**Liver cancer due to hepatitis C**	**DALYs (Disability-Adjusted Life Years)**	**Age group**	**110.47**	**2.0E-52**	**101.52**	**119.41**
Italy	Liver cancer due to hepatitis C	DALYs (Disability-Adjusted Life Years)	Yearly trend 2010–2019	-0.84	9.2E-01	-16.74	15.06
Italy	Liver cancer due to hepatitis C	DALYs (Disability-Adjusted Life Years)	% GPD 5Lag	1.11	9.8E-01	-76.72	78.93
**Italy**	**Liver cancer due to hepatitis C**	**DALYs (Disability-Adjusted Life Years)**	**Sex_Male**	**106.25**	**5.3E-14**	**81.31**	**131.19**
Italy	Liver cancer due to hepatitis C	DALYs (Disability-Adjusted Life Years)	Yearly trend	1.98	8.2E-01	-15.35	19.31
**Italy**	**Liver cancer due to hepatitis C**	**DALYs (Disability-Adjusted Life Years)**	**HIV prevalence_P**	**-0.55**	**5.8E-13**	**-0.69**	**-0.42**
Italy	Liver cancer due to hepatitis C	Deaths	(Intercept)	-27.82	1.2E-01	-62.58	6.95
**Italy**	**Liver cancer due to hepatitis C**	**Deaths**	**Age group**	**9.69**	**3.4E-50**	**8.97**	**10.41**
Italy	Liver cancer due to hepatitis C	Deaths	Yearly trend 2010–2019	-0.05	9.2E-01	-1.11	1.01
Italy	Liver cancer due to hepatitis C	Deaths	% GPD 5Lag	0.11	9.7E-01	-5.08	5.29
**Italy**	**Liver cancer due to hepatitis C**	**Deaths**	**Sex_Male**	**7.84**	**3.0E-15**	**6.16**	**9.52**
Italy	Liver cancer due to hepatitis C	Deaths	Yearly trend	0.24	6.8E-01	-0.91	1.40
**Italy**	**Liver cancer due to hepatitis C**	**Deaths**	**HIV prevalence_P**	**-0.05**	**3.3E-21**	**-0.06**	**-0.04**
Italy	Liver cancer due to hepatitis C	Incidence	(Intercept)	-19.06	3.0E-01	-54.63	16.51
**Italy**	**Liver cancer due to hepatitis C**	**Incidence**	**Age group**	**7.74**	**1.1E-53**	**7.12**	**8.35**
Italy	Liver cancer due to hepatitis C	Incidence	Yearly trend 2010–2019	-0.02	9.7E-01	-1.10	1.07
Italy	Liver cancer due to hepatitis C	Incidence	% GPD 5Lag	0.23	9.3E-01	-5.08	5.55
**Italy**	**Liver cancer due to hepatitis C**	**Incidence**	**Sex_Male**	**7.01**	**2.5E-13**	**5.31**	**8.72**
Italy	Liver cancer due to hepatitis C	Incidence	Yearly trend	0.15	8.0E-01	-1.03	1.33
**Italy**	**Liver cancer due to hepatitis C**	**Incidence**	**HIV prevalence_P**	**-0.05**	**3.4E-19**	**-0.06**	**-0.04**
Italy	Liver cancer due to hepatitis C	Prevalence	(Intercept)	-24.40	3.0E-01	-70.12	21.32
**Italy**	**Liver cancer due to hepatitis C**	**Prevalence**	**Age group**	**9.26**	**3.7E-50**	**8.48**	**10.05**
Italy	Liver cancer due to hepatitis C	Prevalence	Yearly trend 2010–2019	-0.13	8.6E-01	-1.52	1.27
Italy	Liver cancer due to hepatitis C	Prevalence	% GPD 5Lag	0.40	9.1E-01	-6.43	7.23
**Italy**	**Liver cancer due to hepatitis C**	**Prevalence**	**Sex_Male**	**9.17**	**1.2E-13**	**6.98**	**11.36**
Italy	Liver cancer due to hepatitis C	Prevalence	Yearly trend	0.20	8.0E-01	-1.33	1.72
**Italy**	**Liver cancer due to hepatitis C**	**Prevalence**	**HIV prevalence_P**	**-0.04**	**2.4E-08**	**-0.05**	**-0.02**
Italy	Liver cancer due to hepatitis C	YLDs (Years Lived with Disability)	(Intercept)	-4.37	2.9E-01	-12.46	3.71
**Italy**	**Liver cancer due to hepatitis C**	**YLDs (Years Lived with Disability)**	**Age group**	**1.78**	**2.9E-54**	**1.64**	**1.92**
Italy	Liver cancer due to hepatitis C	YLDs (Years Lived with Disability)	Yearly trend 2010–2019	-0.01	9.5E-01	-0.25	0.24
Italy	Liver cancer due to hepatitis C	YLDs (Years Lived with Disability)	% GPD 5Lag	0.05	9.3E-01	-1.16	1.26
**Italy**	**Liver cancer due to hepatitis C**	**YLDs (Years Lived with Disability)**	**Sex_Male**	**1.61**	**1.6E-13**	**1.22**	**2.00**
Italy	Liver cancer due to hepatitis C	YLDs (Years Lived with Disability)	Yearly trend	0.03	8.0E-01	-0.23	0.30
**Italy**	**Liver cancer due to hepatitis C**	**YLDs (Years Lived with Disability)**	**HIV prevalence_P**	**-0.01**	**7.5E-17**	**-0.01**	**-0.01**
Italy	Liver cancer due to hepatitis C	YLLs (Years of Life Lost)	(Intercept)	-390.27	1.0E-01	-857.89	77.35
**Italy**	**Liver cancer due to hepatitis C**	**YLLs (Years of Life Lost)**	**Age group**	**137.93**	**1.4E-52**	**128.25**	**147.60**
Italy	Liver cancer due to hepatitis C	YLLs (Years of Life Lost)	Yearly trend 2010–2019	-1.25	8.6E-01	-15.51	13.00
Italy	Liver cancer due to hepatitis C	YLLs (Years of Life Lost)	% GPD 5Lag	1.30	9.7E-01	-68.45	71.05
**Italy**	**Liver cancer due to hepatitis C**	**YLLs (Years of Life Lost)**	**Sex_Male**	**125.28**	**3.2E-19**	**102.65**	**147.91**
Italy	Liver cancer due to hepatitis C	YLLs (Years of Life Lost)	Yearly trend	2.19	7.8E-01	-13.34	17.72
**Italy**	**Liver cancer due to hepatitis C**	**YLLs (Years of Life Lost)**	**HIV prevalence_P**	**-0.45**	**9.6E-12**	**-0.56**	**-0.33**
Portugal	Cirrhosis and other chronic liver diseases due to hepatitis B	DALYs (Disability-Adjusted Life Years)	(Intercept)	47.38	5.5E-01	-105.77	200.53
**Portugal**	**Cirrhosis and other chronic liver diseases due to hepatitis B**	**DALYs (Disability-Adjusted Life Years)**	**Age group**	**19.72**	**1.5E-18**	**15.91**	**23.53**
Portugal	Cirrhosis and other chronic liver diseases due to hepatitis B	DALYs (Disability-Adjusted Life Years)	Yearly trend 2010–2019	3.40	4.3E-01	-5.02	11.83
Portugal	Cirrhosis and other chronic liver diseases due to hepatitis B	DALYs (Disability-Adjusted Life Years)	% GPD 5Lag	-4.40	6.9E-01	-25.96	17.16
**Portugal**	**Cirrhosis and other chronic liver diseases due to hepatitis B**	**DALYs (Disability-Adjusted Life Years)**	**Sex_Male**	**40.02**	**1.3E-10**	**28.69**	**51.34**
Portugal	Cirrhosis and other chronic liver diseases due to hepatitis B	DALYs (Disability-Adjusted Life Years)	Yearly trend	-5.14	2.0E-01	-12.93	2.65
**Portugal**	**Cirrhosis and other chronic liver diseases due to hepatitis B**	**DALYs (Disability-Adjusted Life Years)**	**HIV prevalence_P**	**0.04**	**3.0E-03**	**0.01**	**0.06**
Portugal	Cirrhosis and other chronic liver diseases due to hepatitis B	Deaths	(Intercept)	1.20	7.0E-01	-4.78	7.18
**Portugal**	**Cirrhosis and other chronic liver diseases due to hepatitis B**	**Deaths**	**Age group**	**1.19**	**5.3E-33**	**1.05**	**1.34**
Portugal	Cirrhosis and other chronic liver diseases due to hepatitis B	Deaths	Yearly trend 2010–2019	0.16	3.3E-01	-0.17	0.49
Portugal	Cirrhosis and other chronic liver diseases due to hepatitis B	Deaths	% GPD 5Lag	-0.20	6.4E-01	-1.04	0.64
**Portugal**	**Cirrhosis and other chronic liver diseases due to hepatitis B**	**Deaths**	**Sex_Male**	**2.08**	**3.3E-16**	**1.64**	**2.52**
Portugal	Cirrhosis and other chronic liver diseases due to hepatitis B	Deaths	Yearly trend	-0.21	1.8E-01	-0.51	0.09
**Portugal**	**Cirrhosis and other chronic liver diseases due to hepatitis B**	**Deaths**	**HIV prevalence_P**	**0.00**	**4.6E-03**	**0.00**	**0.00**
Portugal	Cirrhosis and other chronic liver diseases due to hepatitis B	Incidence	(Intercept)	2.10	2.5E-01	-1.44	5.63
**Portugal**	**Cirrhosis and other chronic liver diseases due to hepatitis B**	**Incidence**	**Age group**	**-0.20**	**2.3E-05**	**-0.28**	**-0.11**
Portugal	Cirrhosis and other chronic liver diseases due to hepatitis B	Incidence	Yearly trend 2010–2019	0.08	4.3E-01	-0.12	0.27
Portugal	Cirrhosis and other chronic liver diseases due to hepatitis B	Incidence	% GPD 5Lag	0.03	9.1E-01	-0.47	0.53
**Portugal**	**Cirrhosis and other chronic liver diseases due to hepatitis B**	**Incidence**	**Sex_Male**	**-0.40**	**2.9E-03**	**-0.67**	**-0.14**
Portugal	Cirrhosis and other chronic liver diseases due to hepatitis B	Incidence	Yearly trend	-0.17	7.4E-02	-0.34	0.01
**Portugal**	**Cirrhosis and other chronic liver diseases due to hepatitis B**	**Incidence**	**HIV prevalence_P**	**0.01**	**7.6E-57**	**0.01**	**0.01**
Portugal	Cirrhosis and other chronic liver diseases due to hepatitis B	Prevalence	(Intercept)	-117.63	7.1E-01	-737.58	502.32
**Portugal**	**Cirrhosis and other chronic liver diseases due to hepatitis B**	**Prevalence**	**Age group**	**255.95**	**5.8E-68**	**240.52**	**271.37**
Portugal	Cirrhosis and other chronic liver diseases due to hepatitis B	Prevalence	Yearly trend 2010–2019	0.15	9.9E-01	-33.96	34.27
Portugal	Cirrhosis and other chronic liver diseases due to hepatitis B	Prevalence	% GPD 5Lag	8.76	8.4E-01	-78.50	96.03
**Portugal**	**Cirrhosis and other chronic liver diseases due to hepatitis B**	**Prevalence**	**Sex_Male**	**135.93**	**3.8E-08**	**90.10**	**181.76**
Portugal	Cirrhosis and other chronic liver diseases due to hepatitis B	Prevalence	Yearly trend	-19.93	2.2E-01	-51.45	11.59
**Portugal**	**Cirrhosis and other chronic liver diseases due to hepatitis B**	**Prevalence**	**HIV prevalence_P**	**1.24**	**5.8E-54**	**1.14**	**1.34**
Portugal	Cirrhosis and other chronic liver diseases due to hepatitis B	YLDs (Years Lived with Disability)	(Intercept)	0.49	5.7E-01	-1.20	2.19
**Portugal**	**Cirrhosis and other chronic liver diseases due to hepatitis B**	**YLDs (Years Lived with Disability)**	**Age group**	**0.28**	**9.4E-26**	**0.24**	**0.32**
Portugal	Cirrhosis and other chronic liver diseases due to hepatitis B	YLDs (Years Lived with Disability)	Yearly trend 2010–2019	0.06	2.3E-01	-0.04	0.15
Portugal	Cirrhosis and other chronic liver diseases due to hepatitis B	YLDs (Years Lived with Disability)	% GPD 5Lag	-0.03	8.3E-01	-0.26	0.21
**Portugal**	**Cirrhosis and other chronic liver diseases due to hepatitis B**	**YLDs (Years Lived with Disability)**	**Sex_Male**	**0.37**	**3.5E-08**	**0.25**	**0.50**
Portugal	Cirrhosis and other chronic liver diseases due to hepatitis B	YLDs (Years Lived with Disability)	Yearly trend	-0.08	7.4E-02	-0.17	0.01
**Portugal**	**Cirrhosis and other chronic liver diseases due to hepatitis B**	**YLDs (Years Lived with Disability)**	**HIV prevalence_P**	**0.00**	**1.8E-03**	**0.00**	**0.00**
Portugal	Cirrhosis and other chronic liver diseases due to hepatitis B	YLLs (Years of Life Lost)	(Intercept)	46.89	5.5E-01	-104.60	198.37
**Portugal**	**Cirrhosis and other chronic liver diseases due to hepatitis B**	**YLLs (Years of Life Lost)**	**Age group**	**19.44**	**1.8E-18**	**15.68**	**23.21**
Portugal	Cirrhosis and other chronic liver diseases due to hepatitis B	YLLs (Years of Life Lost)	Yearly trend 2010–2019	3.35	4.3E-01	-4.99	11.68
Portugal	Cirrhosis and other chronic liver diseases due to hepatitis B	YLLs (Years of Life Lost)	% GPD 5Lag	-4.38	6.9E-01	-25.70	16.95
**Portugal**	**Cirrhosis and other chronic liver diseases due to hepatitis B**	**YLLs (Years of Life Lost)**	**Sex_Male**	**39.64**	**1.3E-10**	**28.44**	**50.84**
Portugal	Cirrhosis and other chronic liver diseases due to hepatitis B	YLLs (Years of Life Lost)	Yearly trend	-5.06	2.0E-01	-12.76	2.64
**Portugal**	**Cirrhosis and other chronic liver diseases due to hepatitis B**	**YLLs (Years of Life Lost)**	**HIV prevalence_P**	**0.04**	**3.0E-03**	**0.01**	**0.06**
Portugal	Cirrhosis and other chronic liver diseases due to hepatitis C	DALYs (Disability-Adjusted Life Years)	(Intercept)	-28.98	8.5E-01	-325.23	267.27
**Portugal**	**Cirrhosis and other chronic liver diseases due to hepatitis C**	**DALYs (Disability-Adjusted Life Years)**	**Age group**	**45.62**	**9.8E-24**	**38.25**	**52.98**
Portugal	Cirrhosis and other chronic liver diseases due to hepatitis C	DALYs (Disability-Adjusted Life Years)	Yearly trend 2010–2019	-0.92	9.1E-01	-17.22	15.38
Portugal	Cirrhosis and other chronic liver diseases due to hepatitis C	DALYs (Disability-Adjusted Life Years)	% GPD 5Lag	-5.81	7.9E-01	-47.51	35.89
**Portugal**	**Cirrhosis and other chronic liver diseases due to hepatitis C**	**DALYs (Disability-Adjusted Life Years)**	**Sex_Male**	**85.07**	**3.3E-12**	**63.17**	**106.97**
Portugal	Cirrhosis and other chronic liver diseases due to hepatitis C	DALYs (Disability-Adjusted Life Years)	Yearly trend	-1.70	8.3E-01	-16.76	13.37
**Portugal**	**Cirrhosis and other chronic liver diseases due to hepatitis C**	**DALYs (Disability-Adjusted Life Years)**	**HIV prevalence_P**	**0.08**	**1.1E-03**	**0.03**	**0.13**
Portugal	Cirrhosis and other chronic liver diseases due to hepatitis C	Deaths	(Intercept)	-3.17	5.9E-01	-14.66	8.32
**Portugal**	**Cirrhosis and other chronic liver diseases due to hepatitis C**	**Deaths**	**Age group**	**2.83**	**7.1E-42**	**2.54**	**3.11**
Portugal	Cirrhosis and other chronic liver diseases due to hepatitis C	Deaths	Yearly trend 2010–2019	0.01	9.8E-01	-0.62	0.64
Portugal	Cirrhosis and other chronic liver diseases due to hepatitis C	Deaths	% GPD 5Lag	-0.28	7.4E-01	-1.90	1.34
**Portugal**	**Cirrhosis and other chronic liver diseases due to hepatitis C**	**Deaths**	**Sex_Male**	**4.54**	**2.0E-19**	**3.69**	**5.39**
Portugal	Cirrhosis and other chronic liver diseases due to hepatitis C	Deaths	Yearly trend	-0.06	8.5E-01	-0.64	0.53
**Portugal**	**Cirrhosis and other chronic liver diseases due to hepatitis C**	**Deaths**	**HIV prevalence_P**	**0.00**	**2.2E-04**	**-0.01**	**0.00**
Portugal	Cirrhosis and other chronic liver diseases due to hepatitis C	Incidence	(Intercept)	0.78	8.3E-01	-6.28	7.83
**Portugal**	**Cirrhosis and other chronic liver diseases due to hepatitis C**	**Incidence**	**Age group**	**-0.55**	**7.1E-09**	**-0.73**	**-0.38**
Portugal	Cirrhosis and other chronic liver diseases due to hepatitis C	Incidence	Yearly trend 2010–2019	-0.06	7.7E-01	-0.45	0.33
Portugal	Cirrhosis and other chronic liver diseases due to hepatitis C	Incidence	% GPD 5Lag	0.28	5.8E-01	-0.71	1.27
**Portugal**	**Cirrhosis and other chronic liver diseases due to hepatitis C**	**Incidence**	**Sex_Male**	**-1.20**	**1.3E-05**	**-1.72**	**-0.68**
Portugal	Cirrhosis and other chronic liver diseases due to hepatitis C	Incidence	Yearly trend	-0.13	4.8E-01	-0.49	0.23
**Portugal**	**Cirrhosis and other chronic liver diseases due to hepatitis C**	**Incidence**	**HIV prevalence_P**	**0.02**	**1.5E-73**	**0.02**	**0.02**
Portugal	Cirrhosis and other chronic liver diseases due to hepatitis C	Prevalence	(Intercept)	-517.61	1.4E-01	-1201.73	166.52
**Portugal**	**Cirrhosis and other chronic liver diseases due to hepatitis C**	**Prevalence**	**Age group**	**500.87**	**6.3E-101**	**483.86**	**517.89**
Portugal	Cirrhosis and other chronic liver diseases due to hepatitis C	Prevalence	Yearly trend 2010–2019	2.40	9.0E-01	-35.25	40.04
Portugal	Cirrhosis and other chronic liver diseases due to hepatitis C	Prevalence	% GPD 5Lag	-11.84	8.1E-01	-108.13	84.46
**Portugal**	**Cirrhosis and other chronic liver diseases due to hepatitis C**	**Prevalence**	**Sex_Male**	**120.14**	**7.3E-06**	**69.56**	**170.71**
Portugal	Cirrhosis and other chronic liver diseases due to hepatitis C	Prevalence	Yearly trend	-1.00	9.5E-01	-35.79	33.78
**Portugal**	**Cirrhosis and other chronic liver diseases due to hepatitis C**	**Prevalence**	**HIV prevalence_P**	**-0.43**	**7.4E-13**	**-0.54**	**-0.32**
Portugal	Cirrhosis and other chronic liver diseases due to hepatitis C	YLDs (Years Lived with Disability)	(Intercept)	-0.85	6.1E-01	-4.11	2.41
**Portugal**	**Cirrhosis and other chronic liver diseases due to hepatitis C**	**YLDs (Years Lived with Disability)**	**Age group**	**0.64**	**1.5E-32**	**0.56**	**0.72**
Portugal	Cirrhosis and other chronic liver diseases due to hepatitis C	YLDs (Years Lived with Disability)	Yearly trend 2010–2019	0.02	8.7E-01	-0.16	0.19
Portugal	Cirrhosis and other chronic liver diseases due to hepatitis C	YLDs (Years Lived with Disability)	% GPD 5Lag	0.02	9.4E-01	-0.44	0.48
**Portugal**	**Cirrhosis and other chronic liver diseases due to hepatitis C**	**YLDs (Years Lived with Disability)**	**Sex_Male**	**0.74**	**1.6E-08**	**0.50**	**0.98**
Portugal	Cirrhosis and other chronic liver diseases due to hepatitis C	YLDs (Years Lived with Disability)	Yearly trend	-0.05	5.6E-01	-0.21	0.12
**Portugal**	**Cirrhosis and other chronic liver diseases due to hepatitis C**	**YLDs (Years Lived with Disability)**	**HIV prevalence_P**	**0.00**	**1.3E-06**	**0.00**	**0.00**
Portugal	Cirrhosis and other chronic liver diseases due to hepatitis C	YLLs (Years of Life Lost)	(Intercept)	-28.13	8.5E-01	-321.18	264.93
**Portugal**	**Cirrhosis and other chronic liver diseases due to hepatitis C**	**YLLs (Years of Life Lost)**	**Age group**	**44.97**	**1.3E-23**	**37.68**	**52.26**
Portugal	Cirrhosis and other chronic liver diseases due to hepatitis C	YLLs (Years of Life Lost)	Yearly trend 2010–2019	-0.93	9.1E-01	-17.06	15.19
Portugal	Cirrhosis and other chronic liver diseases due to hepatitis C	YLLs (Years of Life Lost)	% GPD 5Lag	-5.83	7.8E-01	-47.08	35.42
**Portugal**	**Cirrhosis and other chronic liver diseases due to hepatitis C**	**YLLs (Years of Life Lost)**	**Sex_Male**	**84.33**	**3.0E-12**	**62.67**	**106.00**
Portugal	Cirrhosis and other chronic liver diseases due to hepatitis C	YLLs (Years of Life Lost)	Yearly trend	-1.65	8.3E-01	-16.55	13.25
**Portugal**	**Cirrhosis and other chronic liver diseases due to hepatitis C**	**YLLs (Years of Life Lost)**	**HIV prevalence_P**	**0.08**	**1.1E-03**	**0.03**	**0.12**
Portugal	Liver cancer due to hepatitis B	DALYs (Disability-Adjusted Life Years)	(Intercept)	-57.18	3.2E-01	-170.37	56.00
**Portugal**	**Liver cancer due to hepatitis B**	**DALYs (Disability-Adjusted Life Years)**	**Age group**	**14.86**	**4.6E-19**	**12.04**	**17.67**
Portugal	Liver cancer due to hepatitis B	DALYs (Disability-Adjusted Life Years)	Yearly trend 2010–2019	-0.49	8.8E-01	-6.71	5.74
Portugal	Liver cancer due to hepatitis B	DALYs (Disability-Adjusted Life Years)	% GPD 5Lag	1.45	8.6E-01	-14.48	17.38
**Portugal**	**Liver cancer due to hepatitis B**	**DALYs (Disability-Adjusted Life Years)**	**Sex_Male**	**30.73**	**3.2E-11**	**22.36**	**39.09**
Portugal	Liver cancer due to hepatitis B	DALYs (Disability-Adjusted Life Years)	Yearly trend	0.63	8.3E-01	-5.12	6.38
Portugal	Liver cancer due to hepatitis B	DALYs (Disability-Adjusted Life Years)	HIV prevalence_P	0.01	1.4E-01	0.00	0.03
Portugal	Liver cancer due to hepatitis B	Deaths	(Intercept)	-4.10	6.1E-02	-8.33	0.14
**Portugal**	**Liver cancer due to hepatitis B**	**Deaths**	**Age group**	**1.06**	**4.7E-31**	**0.93**	**1.19**
Portugal	Liver cancer due to hepatitis B	Deaths	Yearly trend 2010–2019	-0.01	9.3E-01	-0.24	0.22
Portugal	Liver cancer due to hepatitis B	Deaths	% GPD 5Lag	0.06	8.3E-01	-0.53	0.66
**Portugal**	**Liver cancer due to hepatitis B**	**Deaths**	**Sex_Male**	**1.90**	**6.4E-21**	**1.58**	**2.22**
Portugal	Liver cancer due to hepatitis B	Deaths	Yearly trend	0.03	7.5E-01	-0.18	0.25
**Portugal**	**Liver cancer due to hepatitis B**	**Deaths**	**HIV prevalence_P**	**0.00**	**1.3E-03**	**0.00**	**0.00**
Portugal	Liver cancer due to hepatitis B	Incidence	(Intercept)	-2.71	2.2E-01	-7.06	1.63
**Portugal**	**Liver cancer due to hepatitis B**	**Incidence**	**Age group**	**0.78**	**4.3E-29**	**0.68**	**0.89**
Portugal	Liver cancer due to hepatitis B	Incidence	Yearly trend 2010–2019	-0.02	9.0E-01	-0.25	0.22
Portugal	Liver cancer due to hepatitis B	Incidence	% GPD 5Lag	0.04	8.9E-01	-0.57	0.66
**Portugal**	**Liver cancer due to hepatitis B**	**Incidence**	**Sex_Male**	**1.49**	**9.1E-16**	**1.16**	**1.81**
Portugal	Liver cancer due to hepatitis B	Incidence	Yearly trend	0.03	7.6E-01	-0.19	0.26
Portugal	Liver cancer due to hepatitis B	Incidence	HIV prevalence_P	0.00	1.8E-01	0.00	0.00
Portugal	Liver cancer due to hepatitis B	Prevalence	(Intercept)	-3.17	2.9E-01	-9.05	2.70
**Portugal**	**Liver cancer due to hepatitis B**	**Prevalence**	**Age group**	**0.79**	**8.9E-20**	**0.65**	**0.94**
Portugal	Liver cancer due to hepatitis B	Prevalence	Yearly trend 2010–2019	-0.02	8.9E-01	-0.35	0.30
Portugal	Liver cancer due to hepatitis B	Prevalence	% GPD 5Lag	0.07	8.6E-01	-0.75	0.90
**Portugal**	**Liver cancer due to hepatitis B**	**Prevalence**	**Sex_Male**	**1.65**	**8.7E-12**	**1.21**	**2.08**
Portugal	Liver cancer due to hepatitis B	Prevalence	Yearly trend	0.04	7.8E-01	-0.26	0.34
Portugal	Liver cancer due to hepatitis B	Prevalence	HIV prevalence_P	0.00	8.8E-02	0.00	0.00
Portugal	Liver cancer due to hepatitis B	YLDs (Years Lived with Disability)	(Intercept)	-0.63	2.3E-01	-1.67	0.41
**Portugal**	**Liver cancer due to hepatitis B**	**YLDs (Years Lived with Disability)**	**Age group**	**0.18**	**4.4E-27**	**0.15**	**0.20**
Portugal	Liver cancer due to hepatitis B	YLDs (Years Lived with Disability)	Yearly trend 2010–2019	0.00	9.0E-01	-0.06	0.05
Portugal	Liver cancer due to hepatitis B	YLDs (Years Lived with Disability)	% GPD 5Lag	0.01	8.8E-01	-0.13	0.16
**Portugal**	**Liver cancer due to hepatitis B**	**YLDs (Years Lived with Disability)**	**Sex_Male**	**0.34**	**5.9E-15**	**0.27**	**0.42**
Portugal	Liver cancer due to hepatitis B	YLDs (Years Lived with Disability)	Yearly trend	0.01	7.6E-01	-0.04	0.06
Portugal	Liver cancer due to hepatitis B	YLDs (Years Lived with Disability)	HIV prevalence_P	0.00	5.3E-01	0.00	0.00
Portugal	Liver cancer due to hepatitis B	YLLs (Years of Life Lost)	(Intercept)	-78.82	2.4E-01	-209.35	51.71
**Portugal**	**Liver cancer due to hepatitis B**	**YLLs (Years of Life Lost)**	**Age group**	**18.10**	**9.5E-15**	**14.12**	**22.07**
Portugal	Liver cancer due to hepatitis B	YLLs (Years of Life Lost)	Yearly trend 2010–2019	-0.60	8.7E-01	-7.75	6.56
Portugal	Liver cancer due to hepatitis B	YLLs (Years of Life Lost)	% GPD 5Lag	1.79	8.5E-01	-16.53	20.10
**Portugal**	**Liver cancer due to hepatitis B**	**YLLs (Years of Life Lost)**	**Sex_Male**	**38.22**	**1.2E-11**	**28.30**	**48.14**
Portugal	Liver cancer due to hepatitis B	YLLs (Years of Life Lost)	Yearly trend	0.78	8.2E-01	-5.83	7.39
Portugal	Liver cancer due to hepatitis B	YLLs (Years of Life Lost)	HIV prevalence_P	0.01	2.1E-01	-0.01	0.03
**Portugal**	**Liver cancer due to hepatitis C**	**DALYs (Disability-Adjusted Life Years)**	**(Intercept)**	**-183.29**	**4.8E-02**	**-363.31**	**-3.27**
**Portugal**	**Liver cancer due to hepatitis C**	**DALYs (Disability-Adjusted Life Years)**	**Age group**	**57.28**	**3.4E-54**	**52.80**	**61.76**
Portugal	Liver cancer due to hepatitis C	DALYs (Disability-Adjusted Life Years)	Yearly trend 2010–2019	-0.07	9.9E-01	-9.98	9.84
Portugal	Liver cancer due to hepatitis C	DALYs (Disability-Adjusted Life Years)	% GPD 5Lag	4.54	7.3E-01	-20.80	29.88
**Portugal**	**Liver cancer due to hepatitis C**	**DALYs (Disability-Adjusted Life Years)**	**Sex_Male**	**62.38**	**4.3E-16**	**49.07**	**75.68**
Portugal	Liver cancer due to hepatitis C	DALYs (Disability-Adjusted Life Years)	Yearly trend	1.76	7.1E-01	-7.39	10.91
**Portugal**	**Liver cancer due to hepatitis C**	**DALYs (Disability-Adjusted Life Years)**	**HIV prevalence_P**	**-0.11**	**4.1E-12**	**-0.14**	**-0.08**
**Portugal**	**Liver cancer due to hepatitis C**	**Deaths**	**(Intercept)**	**-17.35**	**9.0E-03**	**-30.16**	**-4.55**
**Portugal**	**Liver cancer due to hepatitis C**	**Deaths**	**Age group**	**4.89**	**3.4E-47**	**4.50**	**5.28**
Portugal	Liver cancer due to hepatitis C	Deaths	Yearly trend 2010–2019	0.02	9.5E-01	-0.68	0.72
Portugal	Liver cancer due to hepatitis C	Deaths	% GPD 5Lag	0.31	7.4E-01	-1.49	2.10
**Portugal**	**Liver cancer due to hepatitis C**	**Deaths**	**Sex_Male**	**4.41**	**1.2E-14**	**3.44**	**5.38**
Portugal	Liver cancer due to hepatitis C	Deaths	Yearly trend	0.14	6.8E-01	-0.51	0.79
**Portugal**	**Liver cancer due to hepatitis C**	**Deaths**	**HIV prevalence_P**	**-0.01**	**3.4E-15**	**-0.01**	**-0.01**
Portugal	Liver cancer due to hepatitis C	Incidence	(Intercept)	-10.27	7.0E-02	-21.31	0.77
**Portugal**	**Liver cancer due to hepatitis C**	**Incidence**	**Age group**	**3.38**	**3.1E-52**	**3.10**	**3.65**
Portugal	Liver cancer due to hepatitis C	Incidence	Yearly trend 2010–2019	0.00	9.9E-01	-0.61	0.61
Portugal	Liver cancer due to hepatitis C	Incidence	% GPD 5Lag	0.19	8.1E-01	-1.37	1.74
**Portugal**	**Liver cancer due to hepatitis C**	**Incidence**	**Sex_Male**	**3.44**	**8.9E-14**	**2.62**	**4.26**
Portugal	Liver cancer due to hepatitis C	Incidence	Yearly trend	0.12	6.7E-01	-0.44	0.68
**Portugal**	**Liver cancer due to hepatitis C**	**Incidence**	**HIV prevalence_P**	**-0.01**	**2.6E-16**	**-0.01**	**-0.01**
**Portugal**	**Liver cancer due to hepatitis C**	**Prevalence**	**(Intercept)**	**-10.50**	**4.9E-02**	**-20.85**	**-0.15**
**Portugal**	**Liver cancer due to hepatitis C**	**Prevalence**	**Age group**	**3.20**	**1.1E-52**	**2.94**	**3.45**
Portugal	Liver cancer due to hepatitis C	Prevalence	Yearly trend 2010–2019	0.00	1.0E + 00	-0.57	0.57
Portugal	Liver cancer due to hepatitis C	Prevalence	% GPD 5Lag	0.24	7.5E-01	-1.22	1.70
**Portugal**	**Liver cancer due to hepatitis C**	**Prevalence**	**Sex_Male**	**3.47**	**2.3E-15**	**2.71**	**4.24**
Portugal	Liver cancer due to hepatitis C	Prevalence	Yearly trend	0.13	6.4E-01	-0.40	0.65
**Portugal**	**Liver cancer due to hepatitis C**	**Prevalence**	**HIV prevalence_P**	**-0.01**	**3.9E-12**	**-0.01**	**0.00**
Portugal	Liver cancer due to hepatitis C	YLDs (Years Lived with Disability)	(Intercept)	-2.33	6.3E-02	-4.77	0.11
**Portugal**	**Liver cancer due to hepatitis C**	**YLDs (Years Lived with Disability)**	**Age group**	**0.75**	**8.6E-53**	**0.69**	**0.81**
Portugal	Liver cancer due to hepatitis C	YLDs (Years Lived with Disability)	Yearly trend 2010–2019	0.00	1.0E + 00	-0.13	0.13
Portugal	Liver cancer due to hepatitis C	YLDs (Years Lived with Disability)	% GPD 5Lag	0.04	8.0E-01	-0.30	0.39
**Portugal**	**Liver cancer due to hepatitis C**	**YLDs (Years Lived with Disability)**	**Sex_Male**	**0.78**	**2.6E-14**	**0.60**	**0.96**
Portugal	Liver cancer due to hepatitis C	YLDs (Years Lived with Disability)	Yearly trend	0.03	6.6E-01	-0.10	0.15
**Portugal**	**Liver cancer due to hepatitis C**	**YLDs (Years Lived with Disability)**	**HIV prevalence_P**	**0.00**	**9.7E-16**	**0.00**	**0.00**
**Portugal**	**Liver cancer due to hepatitis C**	**YLLs (Years of Life Lost)**	**(Intercept)**	**-265.76**	**1.3E-03**	**-423.57**	**-107.95**
**Portugal**	**Liver cancer due to hepatitis C**	**YLLs (Years of Life Lost)**	**Age group**	**71.28**	**2.9E-54**	**66.47**	**76.09**
Portugal	Liver cancer due to hepatitis C	YLLs (Years of Life Lost)	Yearly trend 2010–2019	-0.16	9.7E-01	-8.82	8.49
Portugal	Liver cancer due to hepatitis C	YLLs (Years of Life Lost)	% GPD 5Lag	5.77	6.1E-01	-16.36	27.91
**Portugal**	**Liver cancer due to hepatitis C**	**YLLs (Years of Life Lost)**	**Sex_Male**	**73.88**	**4.6E-22**	**61.89**	**85.87**
Portugal	Liver cancer due to hepatitis C	YLLs (Years of Life Lost)	Yearly trend	2.13	6.0E-01	-5.87	10.12
**Portugal**	**Liver cancer due to hepatitis C**	**YLLs (Years of Life Lost)**	**HIV prevalence_P**	**-0.09**	**4.3E-12**	**-0.12**	**-0.07**
Spain	Cirrhosis and other chronic liver diseases due to hepatitis B	DALYs (Disability-Adjusted Life Years)	(Intercept)	-10.14	7.6E-01	-74.22	53.93
**Spain**	**Cirrhosis and other chronic liver diseases due to hepatitis B**	**DALYs (Disability-Adjusted Life Years)**	**Age group**	**17.30**	**4.2E-32**	**15.09**	**19.50**
Spain	Cirrhosis and other chronic liver diseases due to hepatitis B	DALYs (Disability-Adjusted Life Years)	Yearly trend 2010–2019	0.53	7.8E-01	-3.18	4.24
Spain	Cirrhosis and other chronic liver diseases due to hepatitis B	DALYs (Disability-Adjusted Life Years)	% GPD 5Lag	-2.13	7.2E-01	-13.72	9.47
**Spain**	**Cirrhosis and other chronic liver diseases due to hepatitis B**	**DALYs (Disability-Adjusted Life Years)**	**Sex_Male**	**27.00**	**2.8E-13**	**20.43**	**33.57**
Spain	Cirrhosis and other chronic liver diseases due to hepatitis B	DALYs (Disability-Adjusted Life Years)	Yearly trend	-1.18	5.8E-01	-5.38	3.01
**Spain**	**Cirrhosis and other chronic liver diseases due to hepatitis B**	**DALYs (Disability-Adjusted Life Years)**	**HIV prevalence_P**	**0.02**	**4.5E-02**	**0.00**	**0.04**
Spain	Cirrhosis and other chronic liver diseases due to hepatitis B	Deaths	(Intercept)	-1.51	4.0E-01	-4.97	1.95
**Spain**	**Cirrhosis and other chronic liver diseases due to hepatitis B**	**Deaths**	**Age group**	**1.22**	**1.2E-43**	**1.11**	**1.34**
Spain	Cirrhosis and other chronic liver diseases due to hepatitis B	Deaths	Yearly trend 2010–2019	0.04	6.6E-01	-0.16	0.24
Spain	Cirrhosis and other chronic liver diseases due to hepatitis B	Deaths	% GPD 5Lag	-0.10	7.5E-01	-0.73	0.52
**Spain**	**Cirrhosis and other chronic liver diseases due to hepatitis B**	**Deaths**	**Sex_Male**	**1.67**	**3.3E-16**	**1.32**	**2.02**
Spain	Cirrhosis and other chronic liver diseases due to hepatitis B	Deaths	Yearly trend	-0.04	7.0E-01	-0.27	0.18
**Spain**	**Cirrhosis and other chronic liver diseases due to hepatitis B**	**Deaths**	**HIV prevalence_P**	**0.00**	**9.4E-09**	**0.00**	**0.00**
Spain	Cirrhosis and other chronic liver diseases due to hepatitis B	Incidence	(Intercept)	1.26	4.6E-01	-2.08	4.60
Spain	Cirrhosis and other chronic liver diseases due to hepatitis B	Incidence	Age group	-0.10	1.0E-01	-0.21	0.02
Spain	Cirrhosis and other chronic liver diseases due to hepatitis B	Incidence	Yearly trend 2010–2019	-0.06	5.2E-01	-0.26	0.13
Spain	Cirrhosis and other chronic liver diseases due to hepatitis B	Incidence	% GPD 5Lag	-0.02	9.5E-01	-0.62	0.59
**Spain**	**Cirrhosis and other chronic liver diseases due to hepatitis B**	**Incidence**	**Sex_Male**	**-0.44**	**1.3E-02**	**-0.78**	**-0.10**
Spain	Cirrhosis and other chronic liver diseases due to hepatitis B	Incidence	Yearly trend	-0.02	8.7E-01	-0.24	0.20
**Spain**	**Cirrhosis and other chronic liver diseases due to hepatitis B**	**Incidence**	**HIV prevalence_P**	**0.01**	**3.6E-35**	**0.01**	**0.01**
Spain	Cirrhosis and other chronic liver diseases due to hepatitis B	Prevalence	(Intercept)	-174.09	3.7E-01	-555.53	207.36
**Spain**	**Cirrhosis and other chronic liver diseases due to hepatitis B**	**Prevalence**	**Age group**	**249.93**	**1.3E-75**	**236.81**	**263.06**
Spain	Cirrhosis and other chronic liver diseases due to hepatitis B	Prevalence	Yearly trend 2010–2019	-4.69	6.8E-01	-26.75	17.38
Spain	Cirrhosis and other chronic liver diseases due to hepatitis B	Prevalence	% GPD 5Lag	4.99	8.9E-01	-64.03	74.01
**Spain**	**Cirrhosis and other chronic liver diseases due to hepatitis B**	**Prevalence**	**Sex_Male**	**63.07**	**1.9E-03**	**23.97**	**102.18**
Spain	Cirrhosis and other chronic liver diseases due to hepatitis B	Prevalence	Yearly trend	-11.50	3.7E-01	-36.49	13.49
**Spain**	**Cirrhosis and other chronic liver diseases due to hepatitis B**	**Prevalence**	**HIV prevalence_P**	**1.38**	**5.3E-45**	**1.25**	**1.51**
Spain	Cirrhosis and other chronic liver diseases due to hepatitis B	YLDs (Years Lived with Disability)	(Intercept)	-0.24	5.4E-01	-0.99	0.52
**Spain**	**Cirrhosis and other chronic liver diseases due to hepatitis B**	**YLDs (Years Lived with Disability)**	**Age group**	**0.28**	**6.8E-45**	**0.25**	**0.30**
Spain	Cirrhosis and other chronic liver diseases due to hepatitis B	YLDs (Years Lived with Disability)	Yearly trend 2010–2019	-0.01	7.6E-01	-0.05	0.04
Spain	Cirrhosis and other chronic liver diseases due to hepatitis B	YLDs (Years Lived with Disability)	% GPD 5Lag	-0.03	7.0E-01	-0.16	0.11
**Spain**	**Cirrhosis and other chronic liver diseases due to hepatitis B**	**YLDs (Years Lived with Disability)**	**Sex_Male**	**0.26**	**6.5E-10**	**0.19**	**0.34**
Spain	Cirrhosis and other chronic liver diseases due to hepatitis B	YLDs (Years Lived with Disability)	Yearly trend	-0.01	8.2E-01	-0.06	0.04
**Spain**	**Cirrhosis and other chronic liver diseases due to hepatitis B**	**YLDs (Years Lived with Disability)**	**HIV prevalence_P**	**0.00**	**9.6E-04**	**0.00**	**0.00**
Spain	Cirrhosis and other chronic liver diseases due to hepatitis B	YLLs (Years of Life Lost)	(Intercept)	-9.91	7.6E-01	-73.25	53.43
**Spain**	**Cirrhosis and other chronic liver diseases due to hepatitis B**	**YLLs (Years of Life Lost)**	**Age group**	**17.02**	**6.3E-32**	**14.84**	**19.20**
Spain	Cirrhosis and other chronic liver diseases due to hepatitis B	YLLs (Years of Life Lost)	Yearly trend 2010–2019	0.54	7.7E-01	-3.13	4.20
Spain	Cirrhosis and other chronic liver diseases due to hepatitis B	YLLs (Years of Life Lost)	% GPD 5Lag	-2.10	7.2E-01	-13.56	9.36
**Spain**	**Cirrhosis and other chronic liver diseases due to hepatitis B**	**YLLs (Years of Life Lost)**	**Sex_Male**	**26.73**	**2.6E-13**	**20.24**	**33.23**
Spain	Cirrhosis and other chronic liver diseases due to hepatitis B	YLLs (Years of Life Lost)	Yearly trend	-1.18	5.8E-01	-5.33	2.97
**Spain**	**Cirrhosis and other chronic liver diseases due to hepatitis B**	**YLLs (Years of Life Lost)**	**HIV prevalence_P**	**0.02**	**4.7E-02**	**0.00**	**0.04**
Spain	Cirrhosis and other chronic liver diseases due to hepatitis C	DALYs (Disability-Adjusted Life Years)	(Intercept)	-75.38	5.3E-01	-310.84	160.07
**Spain**	**Cirrhosis and other chronic liver diseases due to hepatitis C**	**DALYs (Disability-Adjusted Life Years)**	**Age group**	**69.15**	**1.7E-35**	**61.05**	**77.26**
Spain	Cirrhosis and other chronic liver diseases due to hepatitis C	DALYs (Disability-Adjusted Life Years)	Yearly trend 2010–2019	2.88	6.8E-01	-10.74	16.50
Spain	Cirrhosis and other chronic liver diseases due to hepatitis C	DALYs (Disability-Adjusted Life Years)	% GPD 5Lag	-3.36	8.8E-01	-45.97	39.24
**Spain**	**Cirrhosis and other chronic liver diseases due to hepatitis C**	**DALYs (Disability-Adjusted Life Years)**	**Sex_Male**	**92.95**	**4.8E-12**	**68.81**	**117.08**
Spain	Cirrhosis and other chronic liver diseases due to hepatitis C	DALYs (Disability-Adjusted Life Years)	Yearly trend	-4.98	5.3E-01	-20.40	10.45
**Spain**	**Cirrhosis and other chronic liver diseases due to hepatitis C**	**DALYs (Disability-Adjusted Life Years)**	**HIV prevalence_P**	**0.09**	**3.3E-02**	**0.01**	**0.17**
Spain	Cirrhosis and other chronic liver diseases due to hepatitis C	Deaths	(Intercept)	-7.91	2.2E-01	-20.56	4.75
**Spain**	**Cirrhosis and other chronic liver diseases due to hepatitis C**	**Deaths**	**Age group**	**4.96**	**2.0E-48**	**4.52**	**5.39**
Spain	Cirrhosis and other chronic liver diseases due to hepatitis C	Deaths	Yearly trend 2010–2019	0.22	5.5E-01	-0.51	0.96
Spain	Cirrhosis and other chronic liver diseases due to hepatitis C	Deaths	% GPD 5Lag	-0.13	9.1E-01	-2.42	2.16
**Spain**	**Cirrhosis and other chronic liver diseases due to hepatitis C**	**Deaths**	**Sex_Male**	**5.84**	**3.5E-15**	**4.54**	**7.14**
Spain	Cirrhosis and other chronic liver diseases due to hepatitis C	Deaths	Yearly trend	-0.20	6.4E-01	-1.03	0.63
**Spain**	**Cirrhosis and other chronic liver diseases due to hepatitis C**	**Deaths**	**HIV prevalence_P**	**-0.01**	**5.6E-10**	**-0.02**	**-0.01**
Spain	Cirrhosis and other chronic liver diseases due to hepatitis C	Incidence	(Intercept)	4.26	5.4E-01	-9.26	17.78
Spain	Cirrhosis and other chronic liver diseases due to hepatitis C	Incidence	Age group	-0.34	1.5E-01	-0.81	0.13
Spain	Cirrhosis and other chronic liver diseases due to hepatitis C	Incidence	Yearly trend 2010–2019	-0.17	6.8E-01	-0.95	0.61
Spain	Cirrhosis and other chronic liver diseases due to hepatitis C	Incidence	% GPD 5Lag	0.12	9.2E-01	-2.32	2.57
**Spain**	**Cirrhosis and other chronic liver diseases due to hepatitis C**	**Incidence**	**Sex_Male**	**-2.32**	**1.3E-03**	**-3.70**	**-0.93**
Spain	Cirrhosis and other chronic liver diseases due to hepatitis C	Incidence	Yearly trend	-0.13	7.7E-01	-1.02	0.75
**Spain**	**Cirrhosis and other chronic liver diseases due to hepatitis C**	**Incidence**	**HIV prevalence_P**	**0.04**	**6.0E-36**	**0.04**	**0.04**
**Spain**	**Cirrhosis and other chronic liver diseases due to hepatitis C**	**Prevalence**	**(Intercept)**	**-1162.22**	**6.2E-04**	**-1813.26**	**-511.17**
**Spain**	**Cirrhosis and other chronic liver diseases due to hepatitis C**	**Prevalence**	**Age group**	**538.33**	**5.7E-89**	**515.93**	**560.73**
**Spain**	**Cirrhosis and other chronic liver diseases due to hepatitis C**	**Prevalence**	**Yearly trend 2010–2019**	**-53.03**	**6.5E-03**	**-90.69**	**-15.37**
Spain	Cirrhosis and other chronic liver diseases due to hepatitis C	Prevalence	% GPD 5Lag	-9.71	8.7E-01	-127.51	108.10
**Spain**	**Cirrhosis and other chronic liver diseases due to hepatitis C**	**Prevalence**	**Sex_Male**	**123.60**	**3.9E-04**	**56.86**	**190.34**
**Spain**	**Cirrhosis and other chronic liver diseases due to hepatitis C**	**Prevalence**	**Yearly trend**	**55.56**	**1.2E-02**	**12.90**	**98.21**
**Spain**	**Cirrhosis and other chronic liver diseases due to hepatitis C**	**Prevalence**	**HIV prevalence_P**	**-0.76**	**4.0E-10**	**-0.99**	**-0.54**
Spain	Cirrhosis and other chronic liver diseases due to hepatitis C	YLDs (Years Lived with Disability)	(Intercept)	-1.29	3.5E-01	-3.96	1.39
**Spain**	**Cirrhosis and other chronic liver diseases due to hepatitis C**	**YLDs (Years Lived with Disability)**	**Age group**	**1.05**	**1.2E-48**	**0.96**	**1.15**
Spain	Cirrhosis and other chronic liver diseases due to hepatitis C	YLDs (Years Lived with Disability)	Yearly trend 2010–2019	0.00	9.6E-01	-0.15	0.16
Spain	Cirrhosis and other chronic liver diseases due to hepatitis C	YLDs (Years Lived with Disability)	% GPD 5Lag	-0.03	9.1E-01	-0.51	0.46
**Spain**	**Cirrhosis and other chronic liver diseases due to hepatitis C**	**YLDs (Years Lived with Disability)**	**Sex_Male**	**0.83**	**2.2E-08**	**0.56**	**1.10**
Spain	Cirrhosis and other chronic liver diseases due to hepatitis C	YLDs (Years Lived with Disability)	Yearly trend	-0.04	6.6E-01	-0.21	0.14
**Spain**	**Cirrhosis and other chronic liver diseases due to hepatitis C**	**YLDs (Years Lived with Disability)**	**HIV prevalence_P**	**0.00**	**1.2E-05**	**0.00**	**0.00**
Spain	Cirrhosis and other chronic liver diseases due to hepatitis C	YLLs (Years of Life Lost)	(Intercept)	-74.09	5.3E-01	-306.94	158.75
**Spain**	**Cirrhosis and other chronic liver diseases due to hepatitis C**	**YLLs (Years of Life Lost)**	**Age group**	**68.10**	**2.6E-35**	**60.09**	**76.11**
Spain	Cirrhosis and other chronic liver diseases due to hepatitis C	YLLs (Years of Life Lost)	Yearly trend 2010–2019	2.88	6.8E-01	-10.59	16.35
Spain	Cirrhosis and other chronic liver diseases due to hepatitis C	YLLs (Years of Life Lost)	% GPD 5Lag	-3.33	8.8E-01	-45.47	38.80
**Spain**	**Cirrhosis and other chronic liver diseases due to hepatitis C**	**YLLs (Years of Life Lost)**	**Sex_Male**	**92.12**	**4.4E-12**	**68.24**	**115.99**
Spain	Cirrhosis and other chronic liver diseases due to hepatitis C	YLLs (Years of Life Lost)	Yearly trend	-4.94	5.3E-01	-20.19	10.32
**Spain**	**Cirrhosis and other chronic liver diseases due to hepatitis C**	**YLLs (Years of Life Lost)**	**HIV prevalence_P**	**0.09**	**3.6E-02**	**0.01**	**0.17**
Spain	Liver cancer due to hepatitis B	DALYs (Disability-Adjusted Life Years)	(Intercept)	-37.35	2.7E-01	-103.39	28.68
**Spain**	**Liver cancer due to hepatitis B**	**DALYs (Disability-Adjusted Life Years)**	**Age group**	**14.22**	**4.3E-24**	**11.95**	**16.49**
Spain	Liver cancer due to hepatitis B	DALYs (Disability-Adjusted Life Years)	Yearly trend 2010–2019	0.11	9.5E-01	-3.71	3.93
Spain	Liver cancer due to hepatitis B	DALYs (Disability-Adjusted Life Years)	% GPD 5Lag	1.09	8.6E-01	-10.86	13.04
**Spain**	**Liver cancer due to hepatitis B**	**DALYs (Disability-Adjusted Life Years)**	**Sex_Male**	**23.51**	**2.5E-10**	**16.74**	**30.28**
Spain	Liver cancer due to hepatitis B	DALYs (Disability-Adjusted Life Years)	Yearly trend	-0.56	8.0E-01	-4.89	3.76
**Spain**	**Liver cancer due to hepatitis B**	**DALYs (Disability-Adjusted Life Years)**	**HIV prevalence_P**	**0.05**	**1.2E-04**	**0.02**	**0.07**
**Spain**	**Liver cancer due to hepatitis B**	**Deaths**	**(Intercept)**	**-3.69**	**1.5E-02**	**-6.63**	**-0.75**
**Spain**	**Liver cancer due to hepatitis B**	**Deaths**	**Age group**	**1.17**	**1.6E-36**	**1.05**	**1.30**
Spain	Liver cancer due to hepatitis B	Deaths	Yearly trend 2010–2019	0.02	8.1E-01	-0.15	0.19
Spain	Liver cancer due to hepatitis B	Deaths	% GPD 5Lag	0.06	8.3E-01	-0.47	0.59
**Spain**	**Liver cancer due to hepatitis B**	**Deaths**	**Sex_Male**	**1.71**	**4.0E-19**	**1.40**	**2.02**
Spain	Liver cancer due to hepatitis B	Deaths	Yearly trend	-0.02	8.3E-01	-0.21	0.17
**Spain**	**Liver cancer due to hepatitis B**	**Deaths**	**HIV prevalence_P**	**0.00**	**6.4E-03**	**0.00**	**0.00**
Spain	Liver cancer due to hepatitis B	Incidence	(Intercept)	-2.46	1.6E-01	-5.87	0.95
**Spain**	**Liver cancer due to hepatitis B**	**Incidence**	**Age group**	**0.91**	**1.4E-31**	**0.79**	**1.03**
Spain	Liver cancer due to hepatitis B	Incidence	Yearly trend 2010–2019	0.01	9.2E-01	-0.19	0.21
Spain	Liver cancer due to hepatitis B	Incidence	% GPD 5Lag	0.06	8.4E-01	-0.55	0.68
**Spain**	**Liver cancer due to hepatitis B**	**Incidence**	**Sex_Male**	**1.50**	**3.7E-14**	**1.15**	**1.85**
Spain	Liver cancer due to hepatitis B	Incidence	Yearly trend	-0.03	8.3E-01	-0.25	0.20
Spain	Liver cancer due to hepatitis B	Incidence	HIV prevalence_P	0.00	6.9E-01	0.00	0.00
Spain	Liver cancer due to hepatitis B	Prevalence	(Intercept)	-3.13	2.6E-01	-8.59	2.33
**Spain**	**Liver cancer due to hepatitis B**	**Prevalence**	**Age group**	**1.07**	**3.0E-21**	**0.88**	**1.26**
Spain	Liver cancer due to hepatitis B	Prevalence	Yearly trend 2010–2019	-0.02	9.0E-01	-0.34	0.29
Spain	Liver cancer due to hepatitis B	Prevalence	% GPD 5Lag	0.10	8.4E-01	-0.89	1.09
**Spain**	**Liver cancer due to hepatitis B**	**Prevalence**	**Sex_Male**	**2.02**	**5.8E-11**	**1.46**	**2.58**
Spain	Liver cancer due to hepatitis B	Prevalence	Yearly trend	-0.03	8.7E-01	-0.39	0.33
**Spain**	**Liver cancer due to hepatitis B**	**Prevalence**	**HIV prevalence_P**	**0.01**	**1.9E-07**	**0.00**	**0.01**
Spain	Liver cancer due to hepatitis B	YLDs (Years Lived with Disability)	(Intercept)	-0.59	1.8E-01	-1.45	0.27
**Spain**	**Liver cancer due to hepatitis B**	**YLDs (Years Lived with Disability)**	**Age group**	**0.21**	**3.9E-29**	**0.18**	**0.24**
Spain	Liver cancer due to hepatitis B	YLDs (Years Lived with Disability)	Yearly trend 2010–2019	0.00	9.7E-01	-0.05	0.05
Spain	Liver cancer due to hepatitis B	YLDs (Years Lived with Disability)	% GPD 5Lag	0.02	8.4E-01	-0.14	0.17
**Spain**	**Liver cancer due to hepatitis B**	**YLDs (Years Lived with Disability)**	**Sex_Male**	**0.37**	**1.6E-13**	**0.28**	**0.45**
Spain	Liver cancer due to hepatitis B	YLDs (Years Lived with Disability)	Yearly trend	-0.01	8.4E-01	-0.06	0.05
Spain	Liver cancer due to hepatitis B	YLDs (Years Lived with Disability)	HIV prevalence_P	0.00	7.7E-02	0.00	0.00
Spain	Liver cancer due to hepatitis B	YLLs (Years of Life Lost)	(Intercept)	-58.55	1.2E-01	-131.35	14.25
**Spain**	**Liver cancer due to hepatitis B**	**YLLs (Years of Life Lost)**	**Age group**	**18.61**	**6.1E-22**	**15.57**	**21.64**
Spain	Liver cancer due to hepatitis B	YLLs (Years of Life Lost)	Yearly trend 2010–2019	0.13	9.5E-01	-4.06	4.32
Spain	Liver cancer due to hepatitis B	YLLs (Years of Life Lost)	% GPD 5Lag	1.35	8.4E-01	-11.75	14.45
**Spain**	**Liver cancer due to hepatitis B**	**YLLs (Years of Life Lost)**	**Sex_Male**	**28.53**	**4.5E-11**	**20.86**	**36.20**
Spain	Liver cancer due to hepatitis B	YLLs (Years of Life Lost)	Yearly trend	-0.71	7.7E-01	-5.45	4.04
**Spain**	**Liver cancer due to hepatitis B**	**YLLs (Years of Life Lost)**	**HIV prevalence_P**	**0.05**	**1.1E-04**	**0.02**	**0.07**
Spain	Liver cancer due to hepatitis C	DALYs (Disability-Adjusted Life Years)	(Intercept)	-267.27	6.1E-02	-544.89	10.35
**Spain**	**Liver cancer due to hepatitis C**	**DALYs (Disability-Adjusted Life Years)**	**Age group**	**107.37**	**8.2E-48**	**97.82**	**116.92**
Spain	Liver cancer due to hepatitis C	DALYs (Disability-Adjusted Life Years)	Yearly trend 2010–2019	0.19	9.8E-01	-15.87	16.25
Spain	Liver cancer due to hepatitis C	DALYs (Disability-Adjusted Life Years)	% GPD 5Lag	3.42	8.9E-01	-46.82	53.65
**Spain**	**Liver cancer due to hepatitis C**	**DALYs (Disability-Adjusted Life Years)**	**Sex_Male**	**121.57**	**4.7E-14**	**93.11**	**150.03**
Spain	Liver cancer due to hepatitis C	DALYs (Disability-Adjusted Life Years)	Yearly trend	0.50	9.6E-01	-17.69	18.68
**Spain**	**Liver cancer due to hepatitis C**	**DALYs (Disability-Adjusted Life Years)**	**HIV prevalence_P**	**-0.29**	**1.6E-08**	**-0.39**	**-0.20**
**Spain**	**Liver cancer due to hepatitis C**	**Deaths**	**(Intercept)**	**-30.00**	**2.5E-03**	**-49.04**	**-10.96**
**Spain**	**Liver cancer due to hepatitis C**	**Deaths**	**Age group**	**9.88**	**6.8E-47**	**9.09**	**10.68**
Spain	Liver cancer due to hepatitis C	Deaths	Yearly trend 2010–2019	0.06	9.1E-01	-1.03	1.16
Spain	Liver cancer due to hepatitis C	Deaths	% GPD 5Lag	0.25	8.9E-01	-3.18	3.67
**Spain**	**Liver cancer due to hepatitis C**	**Deaths**	**Sex_Male**	**10.03**	**8.6E-17**	**8.03**	**12.04**
Spain	Liver cancer due to hepatitis C	Deaths	Yearly trend	0.12	8.5E-01	-1.12	1.36
**Spain**	**Liver cancer due to hepatitis C**	**Deaths**	**HIV prevalence_P**	**-0.03**	**8.2E-18**	**-0.04**	**-0.03**
Spain	Liver cancer due to hepatitis C	Incidence	(Intercept)	-18.92	5.6E-02	-38.15	0.31
**Spain**	**Liver cancer due to hepatitis C**	**Incidence**	**Age group**	**7.38**	**2.0E-47**	**6.71**	**8.04**
Spain	Liver cancer due to hepatitis C	Incidence	Yearly trend 2010–2019	0.03	9.5E-01	-1.08	1.15
Spain	Liver cancer due to hepatitis C	Incidence	% GPD 5Lag	0.26	8.9E-01	-3.22	3.74
**Spain**	**Liver cancer due to hepatitis C**	**Incidence**	**Sex_Male**	**8.55**	**2.2E-14**	**6.58**	**10.53**
Spain	Liver cancer due to hepatitis C	Incidence	Yearly trend	0.06	9.3E-01	-1.20	1.32
**Spain**	**Liver cancer due to hepatitis C**	**Incidence**	**HIV prevalence_P**	**-0.03**	**2.4E-14**	**-0.04**	**-0.02**
Spain	Liver cancer due to hepatitis C	Prevalence	(Intercept)	-21.71	6.5E-02	-44.61	1.20
**Spain**	**Liver cancer due to hepatitis C**	**Prevalence**	**Age group**	**8.19**	**4.1E-44**	**7.40**	**8.98**
Spain	Liver cancer due to hepatitis C	Prevalence	Yearly trend 2010–2019	-0.07	9.1E-01	-1.40	1.25
Spain	Liver cancer due to hepatitis C	Prevalence	% GPD 5Lag	0.33	8.8E-01	-3.82	4.47
**Spain**	**Liver cancer due to hepatitis C**	**Prevalence**	**Sex_Male**	**10.38**	**8.9E-15**	**8.03**	**12.73**
Spain	Liver cancer due to hepatitis C	Prevalence	Yearly trend	0.08	9.2E-01	-1.42	1.58
**Spain**	**Liver cancer due to hepatitis C**	**Prevalence**	**HIV prevalence_P**	**-0.02**	**2.4E-06**	**-0.03**	**-0.01**
Spain	Liver cancer due to hepatitis C	YLDs (Years Lived with Disability)	(Intercept)	-4.24	5.7E-02	-8.57	0.09
**Spain**	**Liver cancer due to hepatitis C**	**YLDs (Years Lived with Disability)**	**Age group**	**1.67**	**1.3E-47**	**1.52**	**1.82**
Spain	Liver cancer due to hepatitis C	YLDs (Years Lived with Disability)	Yearly trend 2010–2019	0.00	9.8E-01	-0.25	0.25
Spain	Liver cancer due to hepatitis C	YLDs (Years Lived with Disability)	% GPD 5Lag	0.05	8.9E-01	-0.73	0.84
**Spain**	**Liver cancer due to hepatitis C**	**YLDs (Years Lived with Disability)**	**Sex_Male**	**1.94**	**1.5E-14**	**1.50**	**2.39**
Spain	Liver cancer due to hepatitis C	YLDs (Years Lived with Disability)	Yearly trend	0.01	9.3E-01	-0.27	0.30
**Spain**	**Liver cancer due to hepatitis C**	**YLDs (Years Lived with Disability)**	**HIV prevalence_P**	**-0.01**	**1.7E-12**	**-0.01**	**0.00**
**Spain**	**Liver cancer due to hepatitis C**	**YLLs (Years of Life Lost)**	**(Intercept)**	**-413.63**	**1.5E-03**	**-662.60**	**-164.65**
**Spain**	**Liver cancer due to hepatitis C**	**YLLs (Years of Life Lost)**	**Age group**	**137.31**	**2.1E-49**	**126.94**	**147.69**
Spain	Liver cancer due to hepatitis C	YLLs (Years of Life Lost)	Yearly trend 2010–2019	0.07	9.9E-01	-14.25	14.40
Spain	Liver cancer due to hepatitis C	YLLs (Years of Life Lost)	% GPD 5Lag	4.27	8.5E-01	-40.54	49.07
**Spain**	**Liver cancer due to hepatitis C**	**YLLs (Years of Life Lost)**	**Sex_Male**	**144.58**	**4.1E-19**	**118.35**	**170.82**
Spain	Liver cancer due to hepatitis C	YLLs (Years of Life Lost)	Yearly trend	0.52	9.5E-01	-15.70	16.74
**Spain**	**Liver cancer due to hepatitis C**	**YLLs (Years of Life Lost)**	**HIV prevalence_P**	**-0.25**	**1.2E-08**	**-0.33**	**-0.17**
**Western Europe**	**Acute hepatitis B**	**Incidence**	**Yearly trend**	**-2.66**	**0.01**	**-4.50**	**-0.83**
Western Europe	Acute hepatitis B	Incidence	Yearly trend 2010–2019	-1.42	0.37	-4.51	1.67
**Western Europe**	**Acute hepatitis B**	**Incidence**	**Sex_Male**	**29.36**	**0.00**	**20.02**	**38.70**
**Western Europe**	**Acute hepatitis B**	**Incidence**	**Age group**	**-6.01**	**0.00**	**-10.05**	**-1.96**
**Western Europe**	**Acute hepatitis B**	**Incidence**	**HIV incidence**	**19.16**	**0.00**	**17.93**	**20.40**
**Western Europe**	**Acute hepatitis B**	**Prevalence**	**Yearly trend**	**-0.31**	**0.01**	**-0.52**	**-0.10**
Western Europe	Acute hepatitis B	Prevalence	Yearly trend 2010–2019	-0.16	0.37	-0.52	0.19
**Western Europe**	**Acute hepatitis B**	**Prevalence**	**Sex_Male**	**3.39**	**0.00**	**2.31**	**4.47**
**Western Europe**	**Acute hepatitis B**	**Prevalence**	**Age group**	**-0.69**	**0.00**	**-1.16**	**-0.23**
**Western Europe**	**Acute hepatitis B**	**Prevalence**	**HIV incidence**	**2.21**	**0.00**	**2.07**	**2.35**
Western Europe	Acute hepatitis B	Deaths	Yearly trend	0.00	0.09	0.00	0.00
**Western Europe**	**Acute hepatitis B**	**Deaths**	**Yearly trend 2010–2019**	**0.00**	**0.01**	**-0.01**	**0.00**
**Western Europe**	**Acute hepatitis B**	**Deaths**	**Sex_Male**	**0.04**	**0.00**	**0.03**	**0.05**
**Western Europe**	**Acute hepatitis B**	**Deaths**	**Age group**	**0.04**	**0.00**	**0.04**	**0.04**
**Western Europe**	**Acute hepatitis B**	**Deaths**	**HIV incidence**	**0.00**	**0.00**	**0.00**	**0.00**
Western Europe	Acute hepatitis B	DALYs (Disability-Adjusted Life Years)	Yearly trend	0.01	0.60	-0.02	0.03
**Western Europe**	**Acute hepatitis B**	**DALYs (Disability-Adjusted Life Years)**	**Yearly trend 2010–2019**	**-0.06**	**0.01**	**-0.10**	**-0.01**
**Western Europe**	**Acute hepatitis B**	**DALYs (Disability-Adjusted Life Years)**	**Sex_Male**	**0.79**	**0.00**	**0.66**	**0.92**
**Western Europe**	**Acute hepatitis B**	**DALYs (Disability-Adjusted Life Years)**	**Age group**	**0.70**	**0.00**	**0.65**	**0.76**
**Western Europe**	**Acute hepatitis B**	**DALYs (Disability-Adjusted Life Years)**	**HIV incidence**	**0.06**	**0.00**	**0.04**	**0.08**
Western Europe	Acute hepatitis B	YLDs (Years Lived with Disability)	Yearly trend	0.00	0.37	-0.01	0.00
Western Europe	Acute hepatitis B	YLDs (Years Lived with Disability)	Yearly trend 2010–2019	0.00	0.30	-0.01	0.00
**Western Europe**	**Acute hepatitis B**	**YLDs (Years Lived with Disability)**	**Sex_Male**	**0.04**	**0.00**	**0.02**	**0.07**
**Western Europe**	**Acute hepatitis B**	**YLDs (Years Lived with Disability)**	**Age group**	**0.08**	**0.00**	**0.07**	**0.10**
**Western Europe**	**Acute hepatitis B**	**YLDs (Years Lived with Disability)**	**HIV incidence**	**0.05**	**0.00**	**0.05**	**0.05**
Western Europe	Acute hepatitis B	YLLs (Years of Life Lost)	Yearly trend	0.01	0.43	-0.01	0.03
**Western Europe**	**Acute hepatitis B**	**YLLs (Years of Life Lost)**	**Yearly trend 2010–2019**	**-0.05**	**0.01**	**-0.09**	**-0.01**
**Western Europe**	**Acute hepatitis B**	**YLLs (Years of Life Lost)**	**Sex_Male**	**0.74**	**0.00**	**0.62**	**0.86**
**Western Europe**	**Acute hepatitis B**	**YLLs (Years of Life Lost)**	**Age group**	**0.62**	**0.00**	**0.57**	**0.67**
Western Europe	Acute hepatitis B	YLLs (Years of Life Lost)	HIV incidence	0.01	0.11	0.00	0.03
**Western Europe**	**Acute hepatitis C**	**Incidence**	**Yearly trend**	**0.47**	**0.00**	**0.21**	**0.73**
**Western Europe**	**Acute hepatitis C**	**Incidence**	**Yearly trend 2010–2019**	**-0.87**	**0.00**	**-1.31**	**-0.44**
**Western Europe**	**Acute hepatitis C**	**Incidence**	**Sex_Male**	**8.17**	**0.00**	**6.85**	**9.49**
**Western Europe**	**Acute hepatitis C**	**Incidence**	**Age group**	**25.95**	**0.00**	**25.38**	**26.52**
**Western Europe**	**Acute hepatitis C**	**Incidence**	**HIV incidence**	**-2.28**	**0.00**	**-2.45**	**-2.10**
**Western Europe**	**Acute hepatitis C**	**Prevalence**	**Yearly trend**	**0.05**	**0.00**	**0.02**	**0.08**
**Western Europe**	**Acute hepatitis C**	**Prevalence**	**Yearly trend 2010–2019**	**-0.10**	**0.00**	**-0.15**	**-0.05**
**Western Europe**	**Acute hepatitis C**	**Prevalence**	**Sex_Male**	**0.94**	**0.00**	**0.79**	**1.10**
**Western Europe**	**Acute hepatitis C**	**Prevalence**	**Age group**	**2.99**	**0.00**	**2.93**	**3.06**
**Western Europe**	**Acute hepatitis C**	**Prevalence**	**HIV incidence**	**-0.26**	**0.00**	**-0.28**	**-0.24**
**Western Europe**	**Acute hepatitis C**	**Deaths**	**Yearly trend**	**0.00**	**0.00**	**0.00**	**0.00**
Western Europe	Acute hepatitis C	Deaths	Yearly trend 2010–2019	0.00	0.09	0.00	0.00
**Western Europe**	**Acute hepatitis C**	**Deaths**	**Sex_Male**	**0.00**	**0.00**	**0.00**	**0.01**
**Western Europe**	**Acute hepatitis C**	**Deaths**	**Age group**	**0.01**	**0.00**	**0.01**	**0.01**
**Western Europe**	**Acute hepatitis C**	**Deaths**	**HIV incidence**	**0.00**	**0.00**	**0.00**	**0.00**
**Western Europe**	**Acute hepatitis C**	**DALYs (Disability-Adjusted Life Years)**	**Yearly trend**	**-0.02**	**0.00**	**-0.03**	**-0.02**
**Western Europe**	**Acute hepatitis C**	**DALYs (Disability-Adjusted Life Years)**	**Yearly trend 2010–2019**	**0.01**	**0.02**	**0.00**	**0.02**
**Western Europe**	**Acute hepatitis C**	**DALYs (Disability-Adjusted Life Years)**	**Sex_Male**	**0.10**	**0.00**	**0.07**	**0.13**
**Western Europe**	**Acute hepatitis C**	**DALYs (Disability-Adjusted Life Years)**	**Age group**	**0.21**	**0.00**	**0.19**	**0.22**
Western Europe	Acute hepatitis C	DALYs (Disability-Adjusted Life Years)	HIV incidence	0.00	0.89	0.00	0.00
**Western Europe**	**Acute hepatitis C**	**YLDs (Years Lived with Disability)**	**Yearly trend**	**0.00**	**0.00**	**0.00**	**0.00**
**Western Europe**	**Acute hepatitis C**	**YLDs (Years Lived with Disability)**	**Yearly trend 2010–2019**	**0.00**	**0.00**	**0.00**	**0.00**
**Western Europe**	**Acute hepatitis C**	**YLDs (Years Lived with Disability)**	**Sex_Male**	**0.01**	**0.00**	**0.01**	**0.02**
**Western Europe**	**Acute hepatitis C**	**YLDs (Years Lived with Disability)**	**Age group**	**0.04**	**0.00**	**0.04**	**0.04**
**Western Europe**	**Acute hepatitis C**	**YLDs (Years Lived with Disability)**	**HIV incidence**	**0.00**	**0.00**	**0.00**	**0.00**
**Western Europe**	**Acute hepatitis C**	**YLLs (Years of Life Lost)**	**Yearly trend**	**-0.02**	**0.00**	**-0.03**	**-0.02**
**Western Europe**	**Acute hepatitis C**	**YLLs (Years of Life Lost)**	**Yearly trend 2010–2019**	**0.01**	**0.01**	**0.00**	**0.02**
**Western Europe**	**Acute hepatitis C**	**YLLs (Years of Life Lost)**	**Sex_Male**	**0.08**	**0.00**	**0.06**	**0.11**
**Western Europe**	**Acute hepatitis C**	**YLLs (Years of Life Lost)**	**Age group**	**0.16**	**0.00**	**0.15**	**0.18**
Western Europe	Acute hepatitis C	YLLs (Years of Life Lost)	HIV incidence	0.00	0.08	0.00	0.01
**Western Europe**	**Cirrhosis and other chronic liver diseases due to hepatitis B**	**Incidence**	**Yearly trend**	**-0.14**	**0.01**	**-0.23**	**-0.04**
Western Europe	Cirrhosis and other chronic liver diseases due to hepatitis B	Incidence	Yearly trend 2010–2019	0.00	0.97	-0.16	0.16
**Western Europe**	**Cirrhosis and other chronic liver diseases due to hepatitis B**	**Incidence**	**Sex_Male**	**-0.80**	**0.00**	**-1.32**	**-0.28**
**Western Europe**	**Cirrhosis and other chronic liver diseases due to hepatitis B**	**Incidence**	**Age group**	**-0.57**	**0.00**	**-0.78**	**-0.36**
**Western Europe**	**Cirrhosis and other chronic liver diseases due to hepatitis B**	**Incidence**	**HIV prevalence**	**0.02**	**0.00**	**0.02**	**0.02**
**Western Europe**	**Cirrhosis and other chronic liver diseases due to hepatitis B**	**Prevalence**	**Yearly trend**	**-14.40**	**0.00**	**-17.98**	**-10.82**
Western Europe	Cirrhosis and other chronic liver diseases due to hepatitis B	Prevalence	Yearly trend 2010–2019	-0.84	0.78	-6.85	5.17
**Western Europe**	**Cirrhosis and other chronic liver diseases due to hepatitis B**	**Prevalence**	**Sex_Male**	**141.53**	**0.00**	**121.99**	**161.06**
**Western Europe**	**Cirrhosis and other chronic liver diseases due to hepatitis B**	**Prevalence**	**Age group**	**169.15**	**0.00**	**161.34**	**176.96**
**Western Europe**	**Cirrhosis and other chronic liver diseases due to hepatitis B**	**Prevalence**	**HIV prevalence**	**1.62**	**0.00**	**1.52**	**1.73**
**Western Europe**	**Cirrhosis and other chronic liver diseases due to hepatitis B**	**Deaths**	**Yearly trend**	**-0.11**	**0.00**	**-0.18**	**-0.05**
Western Europe	Cirrhosis and other chronic liver diseases due to hepatitis B	Deaths	Yearly trend 2010–2019	0.08	0.12	-0.02	0.19
**Western Europe**	**Cirrhosis and other chronic liver diseases due to hepatitis B**	**Deaths**	**Sex_Male**	**2.16**	**0.00**	**1.82**	**2.51**
**Western Europe**	**Cirrhosis and other chronic liver diseases due to hepatitis B**	**Deaths**	**Age group**	**1.86**	**0.00**	**1.73**	**2.00**
**Western Europe**	**Cirrhosis and other chronic liver diseases due to hepatitis B**	**Deaths**	**HIV prevalence**	**0.00**	**0.00**	**-0.01**	**0.00**
**Western Europe**	**Cirrhosis and other chronic liver diseases due to hepatitis B**	**DALYs (Disability-Adjusted Life Years)**	**Yearly trend**	**-3.24**	**0.00**	**-4.90**	**-1.57**
Western Europe	Cirrhosis and other chronic liver diseases due to hepatitis B	DALYs (Disability-Adjusted Life Years)	Yearly trend 2010–2019	1.48	0.30	-1.32	4.28
**Western Europe**	**Cirrhosis and other chronic liver diseases due to hepatitis B**	**DALYs (Disability-Adjusted Life Years)**	**Sex_Male**	**34.95**	**0.00**	**25.86**	**44.03**
**Western Europe**	**Cirrhosis and other chronic liver diseases due to hepatitis B**	**DALYs (Disability-Adjusted Life Years)**	**Age group**	**27.13**	**0.00**	**23.49**	**30.76**
**Western Europe**	**Cirrhosis and other chronic liver diseases due to hepatitis B**	**DALYs (Disability-Adjusted Life Years)**	**HIV prevalence**	**0.12**	**0.00**	**0.08**	**0.17**
**Western Europe**	**Cirrhosis and other chronic liver diseases due to hepatitis B**	**YLDs (Years Lived with Disability)**	**Yearly trend**	**-0.04**	**0.00**	**-0.06**	**-0.02**
Western Europe	Cirrhosis and other chronic liver diseases due to hepatitis B	YLDs (Years Lived with Disability)	Yearly trend 2010–2019	0.01	0.50	-0.02	0.05
**Western Europe**	**Cirrhosis and other chronic liver diseases due to hepatitis B**	**YLDs (Years Lived with Disability)**	**Sex_Male**	**0.33**	**0.00**	**0.22**	**0.44**
**Western Europe**	**Cirrhosis and other chronic liver diseases due to hepatitis B**	**YLDs (Years Lived with Disability)**	**Age group**	**0.47**	**0.00**	**0.42**	**0.51**
**Western Europe**	**Cirrhosis and other chronic liver diseases due to hepatitis B**	**YLDs (Years Lived with Disability)**	**HIV prevalence**	**0.00**	**0.00**	**0.00**	**0.00**
**Western Europe**	**Cirrhosis and other chronic liver diseases due to hepatitis B**	**YLLs (Years of Life Lost)**	**Yearly trend**	**-3.20**	**0.00**	**-4.84**	**-1.55**
Western Europe	Cirrhosis and other chronic liver diseases due to hepatitis B	YLLs (Years of Life Lost)	Yearly trend 2010–2019	1.47	0.30	-1.29	4.23
**Western Europe**	**Cirrhosis and other chronic liver diseases due to hepatitis B**	**YLLs (Years of Life Lost)**	**Sex_Male**	**34.62**	**0.00**	**25.65**	**43.59**
**Western Europe**	**Cirrhosis and other chronic liver diseases due to hepatitis B**	**YLLs (Years of Life Lost)**	**Age group**	**26.66**	**0.00**	**23.07**	**30.25**
**Western Europe**	**Cirrhosis and other chronic liver diseases due to hepatitis B**	**YLLs (Years of Life Lost)**	**HIV prevalence**	**0.12**	**0.00**	**0.07**	**0.17**
Western Europe	Cirrhosis and other chronic liver diseases due to hepatitis C	Incidence	Yearly trend	-0.19	0.08	-0.40	0.02
Western Europe	Cirrhosis and other chronic liver diseases due to hepatitis C	Incidence	Yearly trend 2010–2019	-0.09	0.64	-0.45	0.27
**Western Europe**	**Cirrhosis and other chronic liver diseases due to hepatitis C**	**Incidence**	**Sex_Male**	**-2.20**	**0.00**	**-3.37**	**-1.03**
**Western Europe**	**Cirrhosis and other chronic liver diseases due to hepatitis C**	**Incidence**	**Age group**	**-1.28**	**0.00**	**-1.74**	**-0.81**
**Western Europe**	**Cirrhosis and other chronic liver diseases due to hepatitis C**	**Incidence**	**HIV prevalence**	**0.05**	**0.00**	**0.05**	**0.06**
**Western Europe**	**Cirrhosis and other chronic liver diseases due to hepatitis C**	**Prevalence**	**Yearly trend**	**17.50**	**0.00**	**11.31**	**23.70**
**Western Europe**	**Cirrhosis and other chronic liver diseases due to hepatitis C**	**Prevalence**	**Yearly trend 2010–2019**	**-31.20**	**0.00**	**-41.62**	**-20.79**
**Western Europe**	**Cirrhosis and other chronic liver diseases due to hepatitis C**	**Prevalence**	**Sex_Male**	**124.34**	**0.00**	**90.51**	**158.16**
**Western Europe**	**Cirrhosis and other chronic liver diseases due to hepatitis C**	**Prevalence**	**Age group**	**704.51**	**0.00**	**690.99**	**718.03**
**Western Europe**	**Cirrhosis and other chronic liver diseases due to hepatitis C**	**Prevalence**	**HIV prevalence**	**-1.15**	**0.00**	**-1.32**	**-0.97**
Western Europe	Cirrhosis and other chronic liver diseases due to hepatitis C	Deaths	Yearly trend	-0.09	0.15	-0.21	0.03
Western Europe	Cirrhosis and other chronic liver diseases due to hepatitis C	Deaths	Yearly trend 2010–2019	0.08	0.44	-0.12	0.28
**Western Europe**	**Cirrhosis and other chronic liver diseases due to hepatitis C**	**Deaths**	**Sex_Male**	**4.61**	**0.00**	**3.96**	**5.26**
**Western Europe**	**Cirrhosis and other chronic liver diseases due to hepatitis C**	**Deaths**	**Age group**	**4.59**	**0.00**	**4.33**	**4.84**
**Western Europe**	**Cirrhosis and other chronic liver diseases due to hepatitis C**	**Deaths**	**HIV prevalence**	**-0.01**	**0.00**	**-0.02**	**-0.01**
**Western Europe**	**Cirrhosis and other chronic liver diseases due to hepatitis C**	**DALYs (Disability-Adjusted Life Years)**	**Yearly trend**	**-3.55**	**0.02**	**-6.42**	**-0.68**
Western Europe	Cirrhosis and other chronic liver diseases due to hepatitis C	DALYs (Disability-Adjusted Life Years)	Yearly trend 2010–2019	0.97	0.70	-3.86	5.79
**Western Europe**	**Cirrhosis and other chronic liver diseases due to hepatitis C**	**DALYs (Disability-Adjusted Life Years)**	**Sex_Male**	**71.48**	**0.00**	**55.80**	**87.16**
**Western Europe**	**Cirrhosis and other chronic liver diseases due to hepatitis C**	**DALYs (Disability-Adjusted Life Years)**	**Age group**	**65.10**	**0.00**	**58.83**	**71.37**
**Western Europe**	**Cirrhosis and other chronic liver diseases due to hepatitis C**	**DALYs (Disability-Adjusted Life Years)**	**HIV prevalence**	**0.19**	**0.00**	**0.11**	**0.27**
Western Europe	Cirrhosis and other chronic liver diseases due to hepatitis C	YLDs (Years Lived with Disability)	Yearly trend	-0.03	0.09	-0.07	0.00
Western Europe	Cirrhosis and other chronic liver diseases due to hepatitis C	YLDs (Years Lived with Disability)	Yearly trend 2010–2019	-0.01	0.65	-0.07	0.04
**Western Europe**	**Cirrhosis and other chronic liver diseases due to hepatitis C**	**YLDs (Years Lived with Disability)**	**Sex_Male**	**0.81**	**0.00**	**0.62**	**1.00**
**Western Europe**	**Cirrhosis and other chronic liver diseases due to hepatitis C**	**YLDs (Years Lived with Disability)**	**Age group**	**1.17**	**0.00**	**1.09**	**1.25**
**Western Europe**	**Cirrhosis and other chronic liver diseases due to hepatitis C**	**YLDs (Years Lived with Disability)**	**HIV prevalence**	**0.00**	**0.00**	**0.00**	**0.00**
**Western Europe**	**Cirrhosis and other chronic liver diseases due to hepatitis C**	**YLLs (Years of Life Lost)**	**Yearly trend**	**-3.52**	**0.02**	**-6.36**	**-0.69**
Western Europe	Cirrhosis and other chronic liver diseases due to hepatitis C	YLLs (Years of Life Lost)	Yearly trend 2010–2019	0.98	0.69	-3.79	5.75
**Western Europe**	**Cirrhosis and other chronic liver diseases due to hepatitis C**	**YLLs (Years of Life Lost)**	**Sex_Male**	**70.68**	**0.00**	**55.18**	**86.17**
**Western Europe**	**Cirrhosis and other chronic liver diseases due to hepatitis C**	**YLLs (Years of Life Lost)**	**Age group**	**63.93**	**0.00**	**57.74**	**70.12**
**Western Europe**	**Cirrhosis and other chronic liver diseases due to hepatitis C**	**YLLs (Years of Life Lost)**	**HIV prevalence**	**0.19**	**0.00**	**0.11**	**0.27**
Western Europe	Liver cancer due to hepatitis B	Incidence	Yearly trend	0.02	0.56	-0.04	0.07
Western Europe	Liver cancer due to hepatitis B	Incidence	Yearly trend 2010–2019	-0.01	0.90	-0.09	0.08
**Western Europe**	**Liver cancer due to hepatitis B**	**Incidence**	**Sex_Male**	**1.69**	**0.00**	**1.41**	**1.96**
**Western Europe**	**Liver cancer due to hepatitis B**	**Incidence**	**Age group**	**1.22**	**0.00**	**1.11**	**1.33**
Western Europe	Liver cancer due to hepatitis B	Incidence	HIV prevalence	0.00	0.08	0.00	0.00
Western Europe	Liver cancer due to hepatitis B	Prevalence	Yearly trend	0.03	0.40	-0.04	0.11
Western Europe	Liver cancer due to hepatitis B	Prevalence	Yearly trend 2010–2019	-0.03	0.63	-0.16	0.10
**Western Europe**	**Liver cancer due to hepatitis B**	**Prevalence**	**Sex_Male**	**2.22**	**0.00**	**1.81**	**2.64**
**Western Europe**	**Liver cancer due to hepatitis B**	**Prevalence**	**Age group**	**1.50**	**0.00**	**1.33**	**1.66**
**Western Europe**	**Liver cancer due to hepatitis B**	**Prevalence**	**HIV prevalence**	**0.00**	**0.00**	**0.00**	**0.01**
Western Europe	Liver cancer due to hepatitis B	Deaths	Yearly trend	0.01	0.64	-0.03	0.05
Western Europe	Liver cancer due to hepatitis B	Deaths	Yearly trend 2010–2019	0.00	0.91	-0.07	0.08
**Western Europe**	**Liver cancer due to hepatitis B**	**Deaths**	**Sex_Male**	**1.56**	**0.00**	**1.32**	**1.80**
**Western Europe**	**Liver cancer due to hepatitis B**	**Deaths**	**Age group**	**1.18**	**0.00**	**1.09**	**1.28**
**Western Europe**	**Liver cancer due to hepatitis B**	**Deaths**	**HIV prevalence**	**0.00**	**0.00**	**0.00**	**0.00**
Western Europe	Liver cancer due to hepatitis B	DALYs (Disability-Adjusted Life Years)	Yearly trend	-0.14	0.78	-1.13	0.85
Western Europe	Liver cancer due to hepatitis B	DALYs (Disability-Adjusted Life Years)	Yearly trend 2010–2019	-0.03	0.98	-1.69	1.64
**Western Europe**	**Liver cancer due to hepatitis B**	**DALYs (Disability-Adjusted Life Years)**	**Sex_Male**	**25.68**	**0.00**	**20.28**	**31.08**
**Western Europe**	**Liver cancer due to hepatitis B**	**DALYs (Disability-Adjusted Life Years)**	**Age group**	**18.44**	**0.00**	**16.28**	**20.60**
**Western Europe**	**Liver cancer due to hepatitis B**	**DALYs (Disability-Adjusted Life Years)**	**HIV prevalence**	**0.04**	**0.00**	**0.02**	**0.07**
Western Europe	Liver cancer due to hepatitis B	YLDs (Years Lived with Disability)	Yearly trend	0.00	0.49	-0.01	0.02
Western Europe	Liver cancer due to hepatitis B	YLDs (Years Lived with Disability)	Yearly trend 2010–2019	0.00	0.82	-0.02	0.02
**Western Europe**	**Liver cancer due to hepatitis B**	**YLDs (Years Lived with Disability)**	**Sex_Male**	**0.41**	**0.00**	**0.34**	**0.48**
**Western Europe**	**Liver cancer due to hepatitis B**	**YLDs (Years Lived with Disability)**	**Age group**	**0.29**	**0.00**	**0.26**	**0.32**
Western Europe	Liver cancer due to hepatitis B	YLDs (Years Lived with Disability)	HIV prevalence	0.00	0.58	0.00	0.00
Western Europe	Liver cancer due to hepatitis B	YLLs (Years of Life Lost)	Yearly trend	-0.14	0.77	-1.12	0.83
Western Europe	Liver cancer due to hepatitis B	YLLs (Years of Life Lost)	Yearly trend 2010–2019	-0.02	0.98	-1.67	1.62
**Western Europe**	**Liver cancer due to hepatitis B**	**YLLs (Years of Life Lost)**	**Sex_Male**	**25.27**	**0.00**	**19.93**	**30.61**
**Western Europe**	**Liver cancer due to hepatitis B**	**YLLs (Years of Life Lost)**	**Age group**	**18.15**	**0.00**	**16.02**	**20.28**
**Western Europe**	**Liver cancer due to hepatitis B**	**YLLs (Years of Life Lost)**	**HIV prevalence**	**0.04**	**0.00**	**0.02**	**0.07**
Western Europe	Liver cancer due to hepatitis C	Incidence	Yearly trend	0.16	0.07	-0.01	0.33
Western Europe	Liver cancer due to hepatitis C	Incidence	Yearly trend 2010–2019	-0.01	0.95	-0.30	0.28
**Western Europe**	**Liver cancer due to hepatitis C**	**Incidence**	**Sex_Male**	**5.64**	**0.00**	**4.70**	**6.57**
**Western Europe**	**Liver cancer due to hepatitis C**	**Incidence**	**Age group**	**6.60**	**0.00**	**6.23**	**6.97**
**Western Europe**	**Liver cancer due to hepatitis C**	**Incidence**	**HIV prevalence**	**-0.03**	**0.00**	**-0.04**	**-0.03**
**Western Europe**	**Liver cancer due to hepatitis C**	**Prevalence**	**Yearly trend**	**0.24**	**0.02**	**0.04**	**0.44**
Western Europe	Liver cancer due to hepatitis C	Prevalence	Yearly trend 2010–2019	-0.08	0.63	-0.42	0.26
**Western Europe**	**Liver cancer due to hepatitis C**	**Prevalence**	**Sex_Male**	**6.87**	**0.00**	**5.76**	**7.97**
**Western Europe**	**Liver cancer due to hepatitis C**	**Prevalence**	**Age group**	**7.78**	**0.00**	**7.34**	**8.22**
**Western Europe**	**Liver cancer due to hepatitis C**	**Prevalence**	**HIV prevalence**	**-0.03**	**0.00**	**-0.03**	**-0.02**
Western Europe	Liver cancer due to hepatitis C	Deaths	Yearly trend	0.14	0.13	-0.04	0.32
Western Europe	Liver cancer due to hepatitis C	Deaths	Yearly trend 2010–2019	0.03	0.87	-0.28	0.33
**Western Europe**	**Liver cancer due to hepatitis C**	**Deaths**	**Sex_Male**	**5.44**	**0.00**	**4.45**	**6.43**
**Western Europe**	**Liver cancer due to hepatitis C**	**Deaths**	**Age group**	**6.67**	**0.00**	**6.27**	**7.06**
**Western Europe**	**Liver cancer due to hepatitis C**	**Deaths**	**HIV prevalence**	**-0.04**	**0.00**	**-0.04**	**-0.03**
Western Europe	Liver cancer due to hepatitis C	DALYs (Disability-Adjusted Life Years)	Yearly trend	1.15	0.31	-1.05	3.34
Western Europe	Liver cancer due to hepatitis C	DALYs (Disability-Adjusted Life Years)	Yearly trend 2010–2019	0.27	0.89	-3.42	3.96
**Western Europe**	**Liver cancer due to hepatitis C**	**DALYs (Disability-Adjusted Life Years)**	**Sex_Male**	**79.16**	**0.00**	**67.18**	**91.14**
**Western Europe**	**Liver cancer due to hepatitis C**	**DALYs (Disability-Adjusted Life Years)**	**Age group**	**94.00**	**0.00**	**89.21**	**98.79**
**Western Europe**	**Liver cancer due to hepatitis C**	**DALYs (Disability-Adjusted Life Years)**	**HIV prevalence**	**-0.33**	**0.00**	**-0.39**	**-0.26**
Western Europe	Liver cancer due to hepatitis C	YLDs (Years Lived with Disability)	Yearly trend	0.04	0.05	0.00	0.08
Western Europe	Liver cancer due to hepatitis C	YLDs (Years Lived with Disability)	Yearly trend 2010–2019	-0.01	0.87	-0.07	0.06
**Western Europe**	**Liver cancer due to hepatitis C**	**YLDs (Years Lived with Disability)**	**Sex_Male**	**1.30**	**0.00**	**1.09**	**1.51**
**Western Europe**	**Liver cancer due to hepatitis C**	**YLDs (Years Lived with Disability)**	**Age group**	**1.53**	**0.00**	**1.45**	**1.61**
**Western Europe**	**Liver cancer due to hepatitis C**	**YLDs (Years Lived with Disability)**	**HIV prevalence**	**-0.01**	**0.00**	**-0.01**	**-0.01**
Western Europe	Liver cancer due to hepatitis C	YLLs (Years of Life Lost)	Yearly trend	1.11	0.32	-1.05	3.27
Western Europe	Liver cancer due to hepatitis C	YLLs (Years of Life Lost)	Yearly trend 2010–2019	0.27	0.88	-3.36	3.90
**Western Europe**	**Liver cancer due to hepatitis C**	**YLLs (Years of Life Lost)**	**Sex_Male**	**77.86**	**0.00**	**66.07**	**89.65**
**Western Europe**	**Liver cancer due to hepatitis C**	**YLLs (Years of Life Lost)**	**Age group**	**92.47**	**0.00**	**87.76**	**97.18**
**Western Europe**	**Liver cancer due to hepatitis C**	**YLLs (Years of Life Lost)**	**HIV prevalence**	**-0.32**	**0.00**	**-0.38**	**-0.26**

Results of the interrupted time series models that included the primary outcome as the dependent variable and the following independent variables or covariates: percentage of gross domestic product (%GDP) for each country studied, sex, age group, period trend for the years 2000 to 2019, post-austerity period trend and HIV burden. To account for the delayed effect of austerity, %GDP was introduced with a five-year lag (5Lag) compared to the epidemiological metrics

#### Chronic hepatitis C virus infection

The incidence rates of CCLD showed a generally stable trend in WE and SWE, whereas the prevalence showed an oscillating trend, resulting in a decrease in WE, which differed from the stabilisation observed in Portugal and Spain at the end of the study period (Fig. 1D, Table 4). Greece showed an increasing prevalence rate, while Italy showed a decreasing trend that did not reach statistical significance. Nevertheless, Italy still had the highest prevalence of CCLD in 2019 with 1214.1 (981.9 – 1497.7) cases per 100,000 population, 3 times higher than Greece and almost 2 times higher than Portugal, Spain and WE (Appendix). Males had significantly higher prevalence rates than females in WE, Portugal and Spain [124.34 (90.51 – 158.16), 120.14 (69.56 – 170.71) and 123.60 (56.86 – 190.34), respectively; *p* < 0.001]. Older individuals showed greater prevalence rate, reaching values of 972.41 cases per 100,000 (937.19; 1007.64; *p* < 0.001) in Italy, 2 to 3 times higher than in the other countries. Overall, a stable trend was observed for mortality and a slight reduction was observed for WE and SWE for DALYs, YLLs, YLDs, and males were more affected than females, reaching the highest values in Italy [(mortality: 6.36 (4.83 – 7.89) and DALYs: 113.83 (89.70 – 137.97); *p* < 0.001]. Older individuals showed greater mortality rate, with Italy standing out with 7.12 cases per 100,000 (6.57; 7.67; *p* < 0.001), between 1.4 and 3.6 times more than WE and the other SWE countries.

The incidence and prevalence rates of LC showed a stable trend in WE and SWE (Table 3, Table 4, Appendix). Age groups and males showed a positive association, although to a lesser extent than for CCLD. A general slight increase was observed for mortality, DALYs, YLL and YLD in WE and SWE. Males showed significantly higher mortality and DALY rates compared to females in Italy, Portugal and Spain, with mortality being 8- and tenfold higher in Italy [7.84 (6.16 – 9.52)] and Spain [10.03 (8.03 – 12.04)] and twofold higher in Portugal [4.41 (3.44 – 5.38)] (*p* < 0.001). Similar trends in mortality and DALYs were observed for age groups, with Italy and Spain being 2 – 5 times higher than Portugal and Greece (*p* < 0.001) (Table 4; Appendix). A negligible effect of HIV on CCLD and LC metrics was observed (Table 4).

#### Association between the burden of viral hepatitis and health expenditure

Overall, an inverse association was observed between health expenditure and both HBV and HCV acute infections, with results close to significance for aHBV DALYs [-2.54 (-5.09 – 0.01); *p* = 0.05)] and aHBV YLLs [-2.53 (-5.09 – 0.03); *p* = 0.05)] in Greece. For aHCV, the results showed that one unit (percentage) increase in health expenditure was associated with a more beneficial effect on reducing incidence cases in Italy [-8.93 (-34.31 – 16.46); *p* = 0.492], up to 80 times higher than in the other SWE countries. A similar inverse association between health expenditure and CCLD metrics was found for both HBV and HCV, except for CCLD-HBV prevalence in Portugal and Spain. In addition, a positive but not significant association was found between health expenditure and LC metrics for both HBV and HCV (Table 4).

## Discussion

This is the first study to analyse the impact of austerity measures related to the 2008 economic crisis on the burden of hepatitis B and hepatitis C infections and related diseases in WE from 2000 to 2019, focusing on the EU countries most affected by austerity measures, namely Greece, Italy, Portugal and Spain. Overall, an inverse association was observed between health expenditure and both HBV and HCV acute infection and CCLD metrics, with a stronger impact on reducing aHBV DALYs and YLLs in Greece and aHCV incidence in Italy. Epidemiological metrics for HBV and HCV showed mixed trends, with better improvement for HBV than HCV, albeit with some country-specific differences, a slower pace of decline in the post-austerity period (2010–2019) and a stabilisation of mortality. An exception was observed for liver cancer due to both hepatitis, with a stagnant burden over time.

In WE, despite a much higher prevalence rate of acute HBV compared to acute HCV, the prevalence of cirrhosis was comparable between HBV and HCV over the study period (2000 – 2019). The higher rate of spontaneous HBV seroclearance (up to 95%) compared to HCV (up to 20% – 30%) [34, 35] may explain the observed convergence in cirrhosis burden between the two infections. The introduction of the HBV vaccine has led to significant improvement in all metrics related to CCLD-HBV [35]. The continuous development and availability of effective antiviral treatment for chronic HBV since the mid-1990s has also contributed to the decline in the burden of sequelae of HBV infection. It has been one of the main factors explaining the disparity between HBV and HCV burdens for decades, until the availability of direct-acting antiviral agents (DAAs), which cure over 95% of HCV cases. The incidence and prevalence rates of acute HBV infection decreased over time in WE and SWE, but most slowly in Greece. Similarly, DALYs, YLLs, YLDs and mortality due to aHBV remained stable over time in WE and SWE, except in Greece, where a sharp increase was observed until 2010, followed by an abrupt decrease. This is likely to be related to the observed inverse association between health expenditure and DALYs, YLLs, YLDs and mortality metrics in SWE, notably in Greece, where austerity measures implemented during the economic crisis had subsequent negative health consequences [36]. Moreover, the highest aHBV mortality in Greece may be at least partly explained by the late introduction of HBV vaccination in 1998 [37], which remained below the recommended 95% coverage for 24-month-old children until 2015 [38, 39]. In contrast, Italy showed the lowest incidence trend, which may be due to the early introduction of universal HBV vaccination in infants in 1991 and in children aged 12 years until 2003 [40]. In Spain, mandatory vaccination was introduced in 1992 for children aged ≥ 12 years and from birth in 1998 [41], and in Portugal in 1995 [42].

However, during the last decade, universal HBV vaccination coverage in children has improved remarkably in all four SWE countries, reaching the 2020 prevention target (95% or more) [38]. In addition, the general improvement in hygiene and healthcare standards, safety standards for blood and its components used for transfusion [43], the use of disposable syringes, the implementation of universal HBsAg screening during pregnancy and prophylaxis of vertical transmission, and information campaigns on HIV/AIDS may also have contributed to the decline in aHBV incidence in the study countries, and more markedly in Italy [44]. The introduction of universal HBV vaccination probably affected the chronic stage of cirrhosis, where a general downward trend, less pronounced in recent years, was observed in WE and SWE, although mortality estimates were extremely low and stable.

Acute HCV incidence and prevalence remained stable over time in WE and SWE, whereas a similar trend to aHBV was found for DALYs, YLLs, YLDs and mortality, with a point of inflection in the post-austerity period for Greece, although to a lesser extent compared to aHBV. The lower burden of aHCV could be related to the marginalisation of high-risk groups, leading to under-diagnosis of HCV disease [34]. Secondly, the later implementation of mandatory notification may explain the different rate between countries, highlighting the earlier mandatory surveillance in Italy in 1990 as opposed to Spain in 2015 [45, 46]. The later mandatory reporting of HCV in some countries may have biased the estimation of the association with HIV incidence, resulting in a negligible effect of HIV on HCV metrics. On the contrary, HIV incidence was positively associated with both aHBV and CCLD-HBV incidence and prevalence. This finding could be explained by the fact that HIV screening contributes to the detection of hepatitis B cases. Similar to aHBV, an inverse association was observed between health expenditure and epidemiological metrics.

The prevalence of HCV cirrhosis stage showed a smaller decrease than HBV cirrhosis stage in WE and SWE over the period 2000 – 2019. Three main factors could explain this observation. First, the constant incidence of acute HCV and the lack of a preventive vaccine. Second, the low rate of spontaneous seroclearance of chronic HCV infection (about 1%) [47, 48]. Third, the low efficacy of conventional treatment based on ribavirin and interferon (about 40% for HCV genotype 1) [49]. However, during the post-austerity period 2010–2019, a more pronounced decrease was observed for CCLD-HCV, which could be explained by the implementation of national HCV treatment plans based on DAAs since 2012 in Greece [50], 2015 in Italy, Portugal and Spain [51–53]. The viral eradication rate reached levels above 90% in the four countries, meeting the 2020 target for the proportion of treated patients achieving a sustained viral response [38, 50, 54].

Recent global GBD data showed a decline in the prevalence of chronic HBV infection over time [6]. On the contrary, our data showed a stable trend of HBV- and HCV-induced liver cancer in WE and SWE. Under reporting of chronic viral hepatitis may partly explain the differences in burden. For example, in Greece 80% of chronic HCV patients are unaware of their status [55], while in Spain the undiagnosed proportion of active HCV infection is 29% [56]. Italy has an estimated 280,000 undiagnosed patients [54]. The new global cancer data from the International Agency for Research on Cancer have estimated that the number of new cases and deaths from liver cancer could increase by > 55% over the next twenty years. However, these estimates are not directly comparable with our results because they include all causes of liver cancer [57].

Sex and age have a major impact on the epidemiological burden of HBV and HCV disease. Males and older people have the highest rates of HBV and HCV disease, except for chronic HBV, which is higher in females in Greece, Portugal and Spain, in line with previously reported data from US veterans [58]. Highest peak of prevalence in older adults as observed in Italy is most probably associated to high transmission in the past through unsafe injections, blood transfusions or other nosocomial transmission routes which has been reported to be the case in Italy. Nowadays, that specific screening test was available for identifying infected donors, people who inject drugs are presently the main target population for infection along with other risk groups, such as migrants.

Notably, health expenditure as a percentage of GDP, used as a proxy for austerity, had a negative impact on acute HBV and HCV infection, mainly on aHBV DALYs and YLLs in Greece and on aHCV incident in Italy. Similar results were found between health expenditure and CCLD metrics for both HBV and HCV.

With the advent of DAAs, new “test and treat” interventions have been introduced recently for key populations at high risk of infection [59]. There are several key populations, such as people living with HIV, sex workers, migrant populations, MSM, and also people who belong to more than one group [60]. For example, HCV prevalence in Portugal is 84% among PWID [61]. In addition, there is growing concern about men who have sex with men (MSM) as a risk group in the HBV/HCV epidemic [62–67]. In 2017, Greece launched the first plan to respond to hepatitis C among PWID, prisoners, sex workers, MSM, refugees and immigrants [68]. In the same year, a key policy document in Portugal recognised the principle of equivalence (United Nations Resolution 45/111 of 14 December 1990), i.e. that prisoners have a right to infectious disease healthcare equivalent to that provided to the general population [69]. This should lead to a significant reduction in the burden of HCV and HBV. For example, interventions piloted in the Italian prison system have shown that micro-elimination [70] of hepatitis C is feasible in both PWID and non-PWID prisoners [71, 72]. The promotion of micro-elimination has also been implemented in Spain over the last decade. For example, a universal test-and-treat intervention supported by telemedicine showed high acceptance among people living in prison and achieved a high HCV cure rate [73–75]. In Portugal, since 2018, a new model of care has been implemented in Portuguese prisons to provide on-site healthcare to eliminate hepatitis C among the vulnerable population of people living in prison [76]. However, several barriers still limit the provision of healthcare in prisons [73, 74].

The impact of DAAs on HCV infection worldwide should be evaluated with longer follow-up, as it may have a major beneficial effect at the public health level. However, despite the high efficacy of DAAs, the risk of reinfection is not eliminated. In Spain, for example, 1.1% of those cured were reinfected [77]. The COVID-19 pandemic could further jeopardise the elimination of HCV and HBV hepatitis by 2030 [78, 79] in several ways, affecting national health systems and their resilience. Approximately 43% – 48% of countries responding to a WHO global survey reported between 5% and > 50% disruption in HBV and HCV diagnosis and treatment in 2021 [80, 81]. In addition, in reallocating resources in response to COVID-19, many countries deprioritised their national responses for critical harm reduction services [80, 81]. In this context, renewed efforts and structured and harmonised policies are needed to provide the framework for eliminating viral hepatitis as a public health threat. Importantly, scaling up testing should be prioritised. This will benefit both the infected person and the community by preventing further transmission. A multi-dimensional approach based on the development of novel point-of-care (POC) diagnostic virological tests and improved screening and linkage-to-care strategies for those most at risk should be promoted. New studies should assess the feasibility and impact of rapid, simple and cost-effective POC technologies on the enrolment and retention of infected individuals in the treatment cascade. POC can change the paradigm of conventional blood testing based on centralised laboratory facilities, widen access to testing and self-testing in community and harm reduction settings or low-resource settings, and engage hard-to-reach populations. At the same time, opioid substitution therapy and needle and syringe programmes should be scaled up in both community and prison settings to counter the negative impact of the current global economic slowdown on people with substance use disorders and vulnerable groups.

The main limitation of this study is the availability of primary data on which the GBD estimates depend. The data sources used in the GBD study are large and comprehensive, including censuses, population registers, vital registration, sample registration (i.e., vital registration covering a sample), demographic data, surveillance systems, verbal autopsies, hospital data, health insurance claims data, surveys, disease registries, morbidity notification data, police records, published literature. In particular, the completeness of the vital registration system in recent years was > 99% in the four countries studied. However, there are still limitations. In fact, the much lower rates of both acute and chronic HCV in Greece, especially at the beginning of the study period, may reflect the scarcity of available data. The accuracy of cause of death and verbal autopsy data depends on death certificates being coded correctly according to international standards and the practices of physicians completing them. Co-morbidities at the time of death can complicate this process, potentially impacting the accuracy of these data sources. The ability of a country to report acute hepatitis may also depend on its testing practices. This may have a direct impact on the confidence intervals, which are wide for Greece for aHBV and aHCV compared to the other countries. Geographical subnational patterns or subgroup-specific analyses were not included in this work, partly because of limited data availability. In terms of methods, the trends for Western Europe included the four countries studied because GBD estimates are available for the whole of Western Europe, which may have introduced a bias in the interpretation of the results. The study was not designed to assess the specific impact of each national policy implemented on the observed epidemiological trends, while a longer study period might have improved the assessment of the impact of DAAs introduction. Surveillance data, such as timely access to therapies for both HBV and HCV, should be examined in future studies as they are released by the relevant national institutions.

## Conclusions

In conclusion, an inverse association was observed between health expenditure and both HBV and HCV acute infection and CCLD metrics, with a stronger impact on reducing aHBV DALYs and YLLs in Greece and aHCV incidence in Italy. Epidemiological metrics for HBV and HCV showed mixed trends, a slower pace of decline in the post-austerity period (2010–2019), a stabilisation of mortality and a stagnant burden for liver cancer due to both hepatitis over time. The 90% reduction in the incidence of chronic HBV and HCV infection and the 65% reduction in attributable mortality from the 2015 baseline recommended by WHO for the 2030 Hepatitis elimination plan have not been achieved [2]. Thus, the elimination of HBV and HCV infection, as endorsed by the Global Health Sector Strategy, remains a challenge, highlighting the critical importance of strong health systems and sustainable funding to address these persistent public health issues.

### Supplementary Information

Supplementary Material 1.

## Data Availability

The data presented in this manuscript have been made publicly available through the Global Health Data Exchange (http://ghdx.healthdata.org/).

## Abbreviations

aHBV: Acute HBV
aHCV: Acute HCV
CCLD: Cirrhosis and other chronic liver diseases
95% CI: 95% Confidence interval
DAAs: Direct-acting antiviral agents
DALYs: Disability-adjusted life years
EU: European Union
GATHER: Guidelines for Accurate and Transparent Health Estimates Reporting
GBD: Global Burden of Diseases, Injuries, and Risk Factors Study
HBV: Hepatitis B virus
HBsAg: Hepatitis B surface antigen
HCV: Hepatitis C virus
HIV: Human immunodeficiency virus
LC: Liver cancer
MSM: Men who have sex with men
PWID: People who inject drugs
SWE: Southern (Greece, Italy, Portugal and Spain) Western European countries
YLDs: Years lived with disability
YLLs: Years of life lost to premature mortality
WE: Western Europe
WHO: World Health Organization
