# Supplementary material for: Impact of the 2008 economic crisis on the burden of hepatitis B and C diseases in Southern European countries
Source: BMC Public Health. 2024 Jun 20;24:1642. doi: 10.1186/s12889-024-18912-0 (PMC11188182; doi:10.1186/s12889-024-18912-0)
Supplement: Supplementary file 1 — Supplementary Material 1. [file 12889_2024_18912_MOESM1_ESM.pdf]

Supplementary appendix

Supplement to:

Impact of the 2008 economic crisis on the burden of HBV and HCV disease in Southern European Countries

Authors

Claudia Palladino*, Rebeca Ramis, Ifeanyi Jude Ezeonwumelu, Antonio Biondi, Giulia Carreras, Florian Fischer, Silvano Gallus, Davide Golinelli, Giuseppe Gorini, Shoaib Hassan, Zubair Kabir, Ai Koyanagi, Jeffrey V Lazarus, Alexios-Fotios A Mentis, Tuomo J Meretoja, Ali H Mokdad, Lorenzo Monasta, Francesk Mulita, Maarten J Postma, Rafael Tabarés-Seisdedos, Arulmani Thiyagarajan, Nuno Taveira¶, Verónica Briz¶

*Corresponding author: cpalladino@edu.ulisboa.pt

¶Contributed equally to the work

GBD 2019 Southern Europe Hepatitis B & C Collaborators

This appendix provides supplemental figures and more detailed results for "Hepatitis B and C burden in Southern European countries (Greece, Italy, Portugal and Spain) between 2000 and 2019: results from the Global Burden of Disease Study 2019".

Table of contents

Methods	3
List of figures and tables	4
Figures	4
Tables	5
Section 1. Statement of GATHER compliance and checklist	12
Section 2. Data seeking and access	15
Section 3. Figures	16
Section 4. Tables	40

Methods

The YLDs metric is estimated multiplying the number of incident disability cases by the average duration of the disability in years and a weight factor that reflects the severity of the disability. The YLLs metric is estimated multiplying the number of deaths by standard life expectancy at age of death (in years). DALYs measure the difference between a current situation and an ideal situation where everyone lives up to the age of the standard life expectancy, and in perfect health. It is calculated as the sum of the time lived with disability and the time lost due to premature mortality (1).

1. WHO. Environmental Burden of Disease Series, No. 1. Assessing the environmental burden of disease at national and local levels [Internet]. 2003. Available from: https://apps.who.int/iris/bitstream/handle/10665/42750/9241546204.pdf

Statistical, analytical, processing, and estimation code used to generate the GBD results are available on the websites: https://ghdx.healthdata.org/gbd-2019/code/cod-3 (Causes of Death 3); https://ghdx.healthdata.org/gbd-2019/code/cod-5 (Causes of Death 5); https://ghdx.healthdata.org/gbd-2019/code/nonfatal-1 (nonfatal health outcome estimation 1); https://ghdx.healthdata.org/gbd-2019/code/nonfatal-3 (nonfatal health outcome estimation 3).

List of figures and tables

Figures and tables are available in section 3 and section 4, respectively.

Figures

Figure S1 - Trends of acute hepatitis B age-standardized rates of prevalence, incidence, mortality, years lived with disability (YLDs), years of life lost (YLLs) and disability-adjusted life years (DALYs) per 100,000 population by sex in Greece, Italy, Portugal, Spain, Western Europe from 2000 to 2019 (Global Burden of Disease Study 2019).

Figure S2 - Trends of acute hepatitis B rates of prevalence, incidence, mortality, years lived with disability (YLDs), years of life lost (YLLs) and disability-adjusted life years (DALYs) per 100,000 population by age group in Greece, Italy, Portugal, Spain and Western Europe from 2000 to 2019 (Global Burden of Disease Study 2019).

Figure S3 - Trends of acute hepatitis C age-standardized rates of prevalence, incidence, mortality, years lived with disability (YLDs), years of life lost (YLLs) and disability-adjusted life years (DALYs) per 100,000 population by sex in Greece, Italy, Portugal, Spain, Western Europe from 2000 to 2019 (Global Burden of Disease Study 2019).

Figure S4 - Trends of acute hepatitis C rates of prevalence, incidence, mortality, years lived with disability (YLDs), years of life lost (YLLs) and disability-adjusted life years (DALYs) per 100,000 population by age group in Greece, Italy, Portugal, Spain and Western Europe from 2000 to 2019 (Global Burden of Disease Study 2019).

Figure S5 - Trends of age-standardized rates of cirrhosis and other chronic liver diseases due to hepatitis B prevalence, incidence, mortality, years lived with disability (YLDs), years of life lost (YLLs) and disability-adjusted life years (DALYs) per 100,000 population by sex in Greece, Italy, Portugal, Spain, Western Europe from 2000 to 2019 (Global Burden of Disease Study 2019).

Figure S6 - Trends of rates of cirrhosis and other chronic liver diseases due to hepatitis B prevalence, incidence, mortality, years lived with disability (YLDs), years of life lost (YLLs) and disability-adjusted life years (DALYs) per 100,000 population by age group in Greece, Italy, Portugal, Spain and Western Europe from 2000 to 2019 (Global Burden of Disease Study 2019).

Figure S7 - Trends of age-standardized rates of cirrhosis and other chronic liver diseases due to hepatitis C prevalence, incidence, mortality, years lived with disability (YLDs), years of life lost (YLLs) and disability-adjusted life years (DALYs) per 100,000 population by sex in Greece, Italy, Portugal, Spain, Western Europe from 2000 to 2019 (Global Burden of Disease Study 2019).

Figure S8 - Trends of rates of cirrhosis and other chronic liver diseases due to hepatitis C prevalence, incidence, mortality, years lived with disability (YLDs), years of life lost (YLLs) and disability-adjusted life years (DALYs) per 100,000 population by age group in Greece, Italy, Portugal, Spain and Western Europe from 2000 to 2019 (Global Burden of Disease Study 2019).

Figure S9 - Trends of age-standardized rates of liver cancer due to hepatitis B prevalence, incidence, mortality, years lived with disability (YLDs), years of life lost (YLLs) and disability-adjusted life years (DALYs) per 100,000 population by sex in Greece, Italy, Portugal, Spain, Western Europe from 2000 to 2019 (Global Burden of Disease Study 2019).

Figure S10 - Trends of rates of liver cancer due to hepatitis B prevalence, incidence, mortality, years lived with disability (YLDs), years of life lost (YLLs) and disability-adjusted life years (DALYs) per 100,000 population by age group in Greece, Italy, Portugal, Spain and Western Europe from 2000 to 2019 (Global Burden of Disease Study 2019).

Figure S11 - Trends of age-standardized rates of liver cancer due to hepatitis C prevalence, incidence, mortality, years lived with disability (YLDs), years of life lost (YLLs) and disability-adjusted life years (DALYs) per 100,000 population by sex in Greece, Italy, Portugal, Spain, Western Europe from 2000 to 2019 (Global Burden of Disease Study 2019).

Figure S12 - Trends of rates of liver cancer due to hepatitis C prevalence, incidence, mortality, years lived with disability (YLDs), years of life lost (YLLs) and disability-adjusted life years (DALYs) per 100,000 population by age group in Greece, Italy, Portugal, Spain and Western Europe from 2000 to 2019 (Global Burden of Disease Study 2019).

Tables

Table S1- Age-standardized rates and 95% uncertainty levels (UL) of acute HBV prevalence, per 100,000 population in Greece, Italy, Portugal and Spain from 2000 to 2019 by sex classes (Global Burden of Disease Study 2019).

Table S2- Age-standardized rates and 95% uncertainty levels (UL) of acute HBV incidence, per 100,000 population in Greece, Italy, Portugal and Spain from 2000 to 2019 by sex classes (Global Burden of Disease Study 2019).

Table S3- Age-standardized rates and 95% uncertainty levels (UL) of acute HBV deaths per 100,000 population in Greece, Italy, Portugal and Spain from 2000 to 2019 by sex classes (Global Burden of Disease Study 2019).

Table S4- Age-standardized rates and 95% uncertainty levels (UL) of acute HBV years of life lost (YLLs) per 100,000 population in Greece, Italy, Portugal and Spain from 2000 to 2019 by sex classes (Global Burden of Disease Study 2019).

Table S5- Age-standardized rates and 95% uncertainty levels (UL) of acute HBV years lived with disability (YLDs) per 100,000 population in Greece, Italy, Portugal and Spain from 2000 to 2019 by sex classes (Global Burden of Disease Study 2019).

Table S6- Age-standardized rates and 95% uncertainty levels (UL) of acute HBV disability-adjusted life years (DALYs) per 100,000 population in Greece, Italy, Portugal and Spain from 2000 to 2019 by sex classes (Global Burden of Disease Study 2019).

Table S7- Age-standardized rates and 95% uncertainty levels (UL) of acute HCV prevalence, per 100,000 population in Greece, Italy, Portugal and Spain from 2000 to 2019 by sex classes (Global Burden of Disease Study 2019).

Table S8- Age-standardized rates and 95% uncertainty levels (UL) of acute HCV incidence, per 100,000 population in Greece, Italy, Portugal and Spain from 2000 to 2019 by sex classes (Global Burden of Disease Study 2019).

Table S9- Age-standardized rates and 95% uncertainty levels (UL) of acute HCV deaths per 100,000 population in Greece, Italy, Portugal and Spain from 2000 to 2019 by sex classes (Global Burden of Disease Study 2019).

Table S10- Age-standardized rates and 95% uncertainty levels (UL) of acute HCV years of life lost (YLLs) per 100,000 population in Greece, Italy, Portugal and Spain from 2000 to 2019 by sex classes (Global Burden of Disease Study 2019).

Table S11- Age-standardized rates and 95% uncertainty levels (UL) of acute HCV years lived with disability (YLDs) per 100,000 population in Greece, Italy, Portugal and Spain from 2000 to 2019 by sex classes (Global Burden of Disease Study 2019).

Table S12- Age-standardized rates and 95% uncertainty levels (UL) of acute HCV disability-adjusted life years (DALYs) per 100,000 population in Greece, Italy, Portugal and Spain from 2000 to 2019 by sex classes (Global Burden of Disease Study 2019).

Table S13- Age-standardized rates and 95% uncertainty levels (UL) of prevalence of cirrhosis and other chronic liver diseases due to hepatitis B per 100,000 population in Greece, Italy, Portugal and Spain from 2000 to 2019 by sex classes (Global Burden of Disease Study 2019).

Table S14- Age-standardized rates and 95% uncertainty levels (UL) of incidence of cirrhosis and other chronic liver diseases due to hepatitis B per 100,000 population in Greece, Italy, Portugal and Spain from 2000 to 2019 by sex classes (Global Burden of Disease Study 2019).

Table S15- Age-standardized rates and 95% uncertainty levels (UL) of deaths due to cirrhosis and other chronic liver diseases due to hepatitis B per 100,000 population in Greece, Italy, Portugal and Spain from 2000 to 2019 by sex classes (Global Burden of Disease Study 2019).

Table S16- Age-standardized rates and 95% uncertainty levels (UL) of years of life lost (YLLs) due to cirrhosis and other chronic liver diseases due to hepatitis B per 100,000 population in Greece, Italy, Portugal and Spain from 2000 to 2019 by sex classes (Global Burden of Disease Study 2019).

Table S17- Age-standardized rates and 95% uncertainty levels (UL) of years lived with disability (YLDs) due to cirrhosis and other chronic liver diseases due to hepatitis B per 100,000 population in Greece, Italy, Portugal and Spain from 2000 to 2019 by sex classes (Global Burden of Disease Study 2019).

Table S18- Age-standardized rates and 95% uncertainty levels (UL) of disability-adjusted life years (DALYs) due to cirrhosis and other chronic liver diseases due to hepatitis B per 100,000 population in Greece, Italy, Portugal and Spain from 2000 to 2019 by sex classes (Global Burden of Disease Study 2019).

Table S19- Age-standardized rates and 95% uncertainty levels (UL) of prevalence of cirrhosis and other chronic liver diseases due to hepatitis C per 100,000 population in Greece, Italy, Portugal and Spain from 2000 to 2019 by sex classes (Global Burden of Disease Study 2019).

Table S20- Age-standardized rates and 95% uncertainty levels (UL) of incidence of cirrhosis and other chronic liver diseases due to hepatitis C per 100,000 population in Greece, Italy, Portugal and Spain from 2000 to 2019 by sex classes (Global Burden of Disease Study 2019).

Table S21- Age-standardized rates and 95% uncertainty levels (UL) of deaths due to cirrhosis and other chronic liver diseases due to hepatitis C per 100,000 population in Greece, Italy, Portugal and Spain from 2000 to 2019 by sex classes (Global Burden of Disease Study 2019).

Table S22- Age-standardized rates and 95% uncertainty levels (UL) of years of life lost (YLLs) due to cirrhosis and other chronic liver diseases due to hepatitis C per 100,000 population in Greece, Italy, Portugal and Spain from 2000 to 2019 by sex classes (Global Burden of Disease Study 2019).

Table S23- Age-standardized rates and 95% uncertainty levels (UL) of years lived with disability (YLDs) due to cirrhosis and other chronic liver diseases due to hepatitis C per 100,000 population in Greece, Italy, Portugal and Spain from 2000 to 2019 by sex classes (Global Burden of Disease Study 2019).

Table S24- Age-standardized rates and 95% uncertainty levels (UL) of disability-adjusted life years (DALYs) due to cirrhosis and other chronic liver diseases due to hepatitis C per 100,000 population in Greece, Italy, Portugal and Spain from 2000 to 2019 by sex classes (Global Burden of Disease Study 2019).

Table S25- Age-standardized rates and 95% uncertainty levels (UL) of prevalence of liver cancer due to hepatitis B per 100,000 population in Greece, Italy, Portugal and Spain from 2000 to 2019 by sex classes (Global Burden of Disease Study 2019).

Table S26- Age-standardized rates and 95% uncertainty levels (UL) of incidence of liver cancer due to hepatitis B per 100,000 population in Greece, Italy, Portugal and Spain from 2000 to 2019 by sex classes (Global Burden of Disease Study 2019).

Table S27- Age-standardized rates and 95% uncertainty levels (UL) of deaths due to liver cancer due to hepatitis B per 100,000 population in Greece, Italy, Portugal and Spain from 2000 to 2019 by sex classes (Global Burden of Disease Study 2019).

Table S28- Age-standardized rates and 95% uncertainty levels (UL) of years of life lost (YLLs) due to liver cancer due to hepatitis B per 100,000 population in Greece, Italy, Portugal and Spain from 2000 to 2019 by sex classes (Global Burden of Disease Study 2019).

Table S29- Age-standardized rates and 95% uncertainty levels (UL) of years lived with disability (YLDs) due to liver cancer due to hepatitis B per 100,000 population in Greece, Italy, Portugal and Spain from 2000 to 2019 by sex classes (Global Burden of Disease Study 2019).

Table S30- Age-standardized rates and 95% uncertainty levels (UL) of disability-adjusted life years (DALYs) due to liver cancer due to hepatitis B per 100,000 population in Greece, Italy, Portugal and Spain from 2000 to 2019 by sex classes (Global Burden of Disease Study 2019).

Table S31- Age-standardized rates and 95% uncertainty levels (UL) of prevalence of liver cancer due to hepatitis C per 100,000 population in Greece, Italy, Portugal and Spain from 2000 to 2019 by sex classes (Global Burden of Disease Study 2019).

Table S32- Age-standardized rates and 95% uncertainty levels (UL) of incidence of liver cancer due to hepatitis C per 100,000 population in Greece, Italy, Portugal and Spain from 2000 to 2019 by sex classes (Global Burden of Disease Study 2019).

Table S33- Age-standardized rates and 95% uncertainty levels (UL) of deaths due to liver cancer due to hepatitis C per 100,000 population in Greece, Italy, Portugal and Spain from 2000 to 2019 by sex classes (Global Burden of Disease Study 2019).

Table S34- Age-standardized rates and 95% uncertainty levels (UL) of years of life lost (YLLs) due to liver cancer due to hepatitis C per 100,000 population in Greece, Italy, Portugal and Spain from 2000 to 2019 by sex classes (Global Burden of Disease Study 2019).

Table S35- Age-standardized rates and 95% uncertainty levels (UL) of years lived with disability (YLDs) due to liver cancer due to hepatitis C per 100,000 population in Greece, Italy, Portugal and Spain from 2000 to 2019 by sex classes (Global Burden of Disease Study 2019).

Table S36- Age-standardized rates and 95% uncertainty levels (UL) of disability-adjusted life years (DALYs) due to liver cancer due to hepatitis C per 100,000 population in Greece, Italy, Portugal and Spain from 2000 to 2019 by sex classes (Global Burden of Disease Study 2019).

Table S37- Rates and 95% uncertainty levels (UL) of acute HBV prevalence per 100,000 population in Greece, Italy, Portugal and Spain from 2000 to 2019 by age group (Global Burden of Disease Study 2019).

Table S38- Rates and 95% uncertainty levels (UL) of acute HBV incidence per 100,000 population in Greece, Italy, Portugal and Spain from 2000 to 2019 by age group (Global Burden of Disease Study 2019).

Table S39- Rates and 95% uncertainty levels (UL) of acute HBV deaths per 100,000 population in Greece, Italy, Portugal and Spain from 2000 to 2019 by age group (Global Burden of Disease Study 2019).

Table S40- Rates and 95% uncertainty levels (UL) of acute HBV years of life lost (YLLs) per 100,000 population in Greece, Italy, Portugal and Spain from 2000 to 2019 by age group (Global Burden of Disease Study 2019).

Table S41- Rates and 95% uncertainty levels (UL) of acute HBV years lived with disability (YLDs) per 100,000 population in Greece, Italy, Portugal and Spain from 2000 to 2019 by age group (Global Burden of Disease Study 2019).

Table S42- Rates and 95% uncertainty levels (UL) of acute HBV disability-adjusted life years (DALYs) per 100,000 population in Greece, Italy, Portugal and Spain from 2000 to 2019 by age group (Global Burden of Disease Study 2019).

Table S43- Rates and 95% uncertainty levels (UL) of acute HCV prevalence per 100,000 population in Greece, Italy, Portugal and Spain from 2000 to 2019 by age group (Global Burden of Disease Study 2019).

Table S44- Rates and 95% uncertainty levels (UL) of acute HCV incidence per 100,000 population in Greece, Italy, Portugal and Spain from 2000 to 2019 by age group (Global Burden of Disease Study 2019).

Table S45- Rates and 95% uncertainty levels (UL) of acute HCV deaths per 100,000 population in Greece, Italy, Portugal and Spain from 2000 to 2019 by age group (Global Burden of Disease Study 2019).

Table S46- Rates and 95% uncertainty levels (UL) of acute HCV years of life lost (YLLs) per 100,000 population in Greece, Italy, Portugal and Spain from 2000 to 2019 by age group (Global Burden of Disease Study 2019).

Table S47- Rates and 95% uncertainty levels (UL) of acute HCV years lived with disability (YLDs) per 100,000 population in Greece, Italy, Portugal and Spain from 2000 to 2019 by age group (Global Burden of Disease Study 2019).

Table S48- Rates and 95% uncertainty levels (UL) of acute HCV disability-adjusted life years (DALYs) per 100,000 population in Greece, Italy, Portugal and Spain from 2000 to 2019 by age group (Global Burden of Disease Study 2019).

Table S49- Rates and 95% uncertainty levels (UL) of prevalence of cirrhosis and other chronic liver diseases due to hepatitis B per 100,000 population in Greece, Italy, Portugal and Spain from 2000 to 2019 by age group (Global Burden of Disease Study 2019).

Table S50- Rates and 95% uncertainty levels (UL) of incidence of cirrhosis and other chronic liver diseases due to hepatitis B per 100,000 population in Greece, Italy, Portugal and Spain from 2000 to 2019 by age group (Global Burden of Disease Study 2019).

Table S51- Rates and 95% uncertainty levels (UL) of deaths due to cirrhosis and other chronic liver diseases due to hepatitis B per 100,000 population in Greece, Italy, Portugal and Spain from 2000 to 2019 by age group (Global Burden of Disease Study 2019).

Table S52- Rates and 95% uncertainty levels (UL) of years of life lost (YLLs) due to cirrhosis and other chronic liver diseases due to hepatitis B per 100,000 population in Greece, Italy, Portugal and Spain from 2000 to 2019 by age group (Global Burden of Disease Study 2019).

Table S53- Rates and 95% uncertainty levels (UL) of years lived with disability (YLDs) due to cirrhosis and other chronic liver diseases due to hepatitis B per 100,000 population in Greece, Italy, Portugal and Spain from 2000 to 2019 by age group (Global Burden of Disease Study 2019).

Table S54- Rates and 95% uncertainty levels (UL) of disability-adjusted life years (DALYs) due to cirrhosis and other chronic liver diseases due to hepatitis B per 100,000 population in Greece, Italy, Portugal and Spain from 2000 to 2019 by age group (Global Burden of Disease Study 2019).

Table S55- Rates and 95% uncertainty levels (UL) of prevalence of cirrhosis and other chronic liver diseases due to hepatitis C per 100,000 population in Greece, Italy, Portugal and Spain from 2000 to 2019 by age group (Global Burden of Disease Study 2019).

Table S56- Rates and 95% uncertainty levels (UL) of incidence of cirrhosis and other chronic liver diseases due to hepatitis C per 100,000 population in Greece, Italy, Portugal and Spain from 2000 to 2019 by age group (Global Burden of Disease Study 2019).

Table S57- Rates and 95% uncertainty levels (UL) of deaths due to cirrhosis and other chronic liver diseases due to hepatitis C per 100,000 population in Greece, Italy, Portugal and Spain from 2000 to 2019 by age group (Global Burden of Disease Study 2019).

Table S58- Rates and 95% uncertainty levels (UL) of years of life lost (YLLs) due to cirrhosis and other chronic liver diseases due to hepatitis C per 100,000 population in Greece, Italy, Portugal and Spain from 2000 to 2019 by age group (Global Burden of Disease Study 2019).

Table S59- Rates and 95% uncertainty levels (UL) of years lived with disability (YLDs) due to cirrhosis and other chronic liver diseases due to hepatitis C per 100,000 population in Greece, Italy, Portugal and Spain from 2000 to 2019 by age group (Global Burden of Disease Study 2019).

Table S60- Rates and 95% uncertainty levels (UL) of disability-adjusted life years (DALYs) due to cirrhosis and other chronic liver diseases due to hepatitis C per 100,000 population in

Greece, Italy, Portugal and Spain from 2000 to 2019 by age group (Global Burden of Disease Study 2019).

Table S61- Rates and 95% uncertainty levels (UL) of prevalence of liver cancer due to hepatitis B per 100,000 population in Greece, Italy, Portugal and Spain from 2000 to 2019 by age group (Global Burden of Disease Study 2019).

Table S62- Rates and 95% uncertainty levels (UL) of incidence of liver cancer due to hepatitis B per 100,000 population in Greece, Italy, Portugal and Spain from 2000 to 2019 by age group (Global Burden of Disease Study 2019).

Table S63- Rates and 95% uncertainty levels (UL) of deaths due to liver cancer due to hepatitis B per 100,000 population in Greece, Italy, Portugal and Spain from 2000 to 2019 by age group (Global Burden of Disease Study 2019).

Table S64- Rates and 95% uncertainty levels (UL) of years of life lost (YLLs) due to liver cancer due to hepatitis B per 100,000 population in Greece, Italy, Portugal and Spain from 2000 to 2019 by age group (Global Burden of Disease Study 2019).

Table S65- Rates and 95% uncertainty levels (UL) of years lived with disability (YLDs) due to liver cancer due to hepatitis B per 100,000 population in Greece, Italy, Portugal and Spain from 2000 to 2019 by age group (Global Burden of Disease Study 2019).

Table S66- Rates and 95% uncertainty levels (UL) of disability-adjusted life years (DALYs) due to liver cancer due to hepatitis B per 100,000 population in Greece, Italy, Portugal and Spain from 2000 to 2019 by age group (Global Burden of Disease Study 2019).

Table S67- Rates and 95% uncertainty levels (UL) of prevalence of liver cancer due to hepatitis C per 100,000 population in Greece, Italy, Portugal and Spain from 2000 to 2019 by age group (Global Burden of Disease Study 2019).

Table S68- Rates and 95% uncertainty levels (UL) of incidence of liver cancer due to hepatitis C per 100,000 population in Greece, Italy, Portugal and Spain from 2000 to 2019 by age group (Global Burden of Disease Study 2019).

Table S69- Rates and 95% uncertainty levels (UL) of deaths due to liver cancer due to hepatitis C per 100,000 population in Greece, Italy, Portugal and Spain from 2000 to 2019 by age group (Global Burden of Disease Study 2019).

Table S70- Rates and 95% uncertainty levels (UL) of years of life lost (YLLs) due to liver cancer due to hepatitis C per 100,000 population in Greece, Italy, Portugal and Spain from 2000 to 2019 by age group (Global Burden of Disease Study 2019).

Table S71- Rates and 95% uncertainty levels (UL) of years lived with disability (YLDs) due to liver cancer due to hepatitis C per 100,000 population in Greece, Italy, Portugal and Spain from 2000 to 2019 by age group (Global Burden of Disease Study 2019).

Table S72- Rates and 95% uncertainty levels (UL) of disability-adjusted life years (DALYs) due to liver cancer due to hepatitis C per 100,000 population in Greece, Italy, Portugal and Spain from 2000 to 2019 by age group (Global Burden of Disease Study 2019).

Table S73- Prevalence of acute and chronic hepatitis B for Greece, Italy, Portugal and Spain, Western Europe in 2000, 2010, and 2019 (Global Burden of Disease Study 2019).

Table S74- Prevalence of acute and chronic hepatitis C for Greece, Italy, Portugal and Spain, Western Europe in 2000, 2010, and 2019 (Global Burden of Disease Study 2019).

Table S75- Incidence of acute and chronic hepatitis B for Greece, Italy, Portugal and Spain, Western Europe in 2000, 2010, and 2019 (Global Burden of Disease Study 2019).

Table S76- Incidence of acute and chronic hepatitis C for Greece, Italy, Portugal and Spain, Western Europe in 2000, 2010, and 2019 (Global Burden of Disease Study 2019).

Table S77- YLDs of acute and chronic hepatitis B for Greece, Italy, Portugal and Spain, Western Europe in 2000, 2010, and 2019 (Global Burden of Disease Study 2019).

Table S78- YLDs of acute and chronic hepatitis C for Greece, Italy, Portugal and Spain, Western Europe in 2000, 2010, and 2019 (Global Burden of Disease Study 2019).

Table S79- YLLs of acute and chronic hepatitis B for Greece, Italy, Portugal and Spain, Western Europe in 2000, 2010, and 2019 (Global Burden of Disease Study 2019).

Table S80- YLLs of acute and chronic hepatitis C for Greece, Italy, Portugal and Spain, Western Europe in 2000, 2010, and 2019 (Global Burden of Disease Study 2019).

Table S81- DALYs of acute and chronic hepatitis B for Greece, Italy, Portugal and Spain, Western Europe in 2000, 2010, and 2019 (Global Burden of Disease Study 2019).

Table S82- DALYs of acute and chronic hepatitis C for Greece, Italy, Portugal and Spain, Western Europe in 2000, 2010, and 2019 (Global Burden of Disease Study 2019).

Section 1. Statement of GATHER compliance and checklist

This study complies with the Guidelines for Accurate and Transparent Health Estimates Reporting (GATHER) recommendations.

GATHER checklist of information that should be included in reports of global health estimates

Item number	Checklist item	Reported on page #
**Objectives and funding**		
1	Define the indicator(s), populations (including age, sex, and geographic entities), and time period(s) for which estimates were made.	Main text (Methods, pg. 6-7, Supplementary Appendix pag.3)
2	List the funding sources for the work.	Main text (Methods, pg. 8; Acknowledgments, pg. 17)
**Data inputs**		
For all data inputs from multiple sources that are synthesised as part of the study:		
3	Describe how the data were identified and how the data were accessed.	Main text (Methods, pg. 6-7, Data sharing, pg. 20, Supplementary Appendix pag.15
4	Specify the inclusion and exclusion criteria. Identify all ad-hoc exclusions.	Main text (Methods, pg. 6-7)
5	Provide information about all included data sources and their main characteristics. For each data source used, report reference information or contact name/institution, population represented, data collection method, year(s) of data collection, sex and age range, diagnostic criteria or measurement method, and sample size, as relevant.	Main text (Methods, pg. 6-7, Supplementary Appendix pag.14
6	Identify and describe any categories of input data that have potentially important biases (eg, based on characteristics listed in item 5).	No problems were observed

For data inputs that contribute to the analysis but were not synthesised as part of the study:		
7	Describe and give sources for any other data inputs.	Not applicable
For all data inputs:		
8	Provide all data inputs in a file format from which data can be efficiently extracted (eg, a spreadsheet rather than a PDF), including all relevant meta-data listed in item 5. For any data inputs that cannot be shared because of ethical or legal reasons, such as third-party ownership, provide a contact name or the name of the institution that retains the right to the data.	Online data visualization tools, data query tools, and the Global Health Data Exchange http://ghdx.healthdata.org
**Data analysis**		
9	Provide a conceptual overview of the data analysis method. A diagram may be helpful.	Main text (Methods; pg. 6-7)
10	Provide a detailed description of all steps of the analysis, including mathematical formulae. This description should cover, as relevant, data cleaning, data pre-processing, data adjustments and weighting of data sources, and mathematical or statistical model(s).	Main text (Methods; pg. 6-7)
11	Describe how candidate models were evaluated and how the final model(s) were selected.	Not applicable
12	Provide the results of an evaluation of model performance, if done, as well as the results of any relevant sensitivity analysis.	Not applicable
13	Describe methods of calculating uncertainty of the estimates. State which sources of uncertainty were, and were not, accounted for in the uncertainty analysis.	Main text (Methods; pg. 7)
14	State how analytical or statistical source code used to generate estimates can be accessed.	Supplementary Appendix pg. 3. Code is provided in an online repository, http://ghdx.healthdata.org/gbd-2019/code

**Results and discussion**		
15	Provide published estimates in a file format from which data can be efficiently extracted.	Visualization tools, data query tools, and the Global Health Data Exchange, http://ghdx.healthdata.org/gbd-2019
16	Report a quantitative measure of the uncertainty of the estimates (eg, uncertainty intervals).	Main text (Results, pg. 8-12; all tables and figures also in the Supplementary Appendix)
17	Interpret results in light of existing evidence. If updating a previous set of estimates, describe the reasons for changes in estimates.	Main text (Discussion, pg 12-17)
18	Discuss limitations of the estimates. Include a discussion of any modelling assumptions or data limitations that affect interpretation of the estimates.	Main text (Discussion, pg 15-16)

Section 2. Data seeking and access

Raw data for the study countries and diseases may be retrieved through the Global Health Data Exchange at the following links which provides full citation information for all sources:
http://ghdx.healthdata.org/geography/greece;
http://ghdx.healthdata.org/geography/italy;
http://ghdx.healthdata.org/geography/portugal;
http://ghdx.healthdata.org/geography/spain;
http://ghdx.healthdata.org/keyword/hepatitis-b;
http://ghdx.healthdata.org/keyword/hepatitis-c;
http://ghdx.healthdata.org/keyword/cirrhosis-liver
http://ghdx.healthdata.org/keyword/liver-cancer.
Data inputs are made publicly available through the Global Health Data Exchange (http://ghdx.healthdata.org/).

Section 3. Figures

Figure S1 - Trends of **acute hepatitis B age-standardized rates** of prevalence, incidence, mortality, years lived with disability (YLDs), years of life lost (YLLs) and disability-adjusted life years (DALYs) per 100,000 population by sex in Greece, Italy, Portugal, Spain, Western Europe from 2000 to 2019 (Global Burden of Disease Study 2019).

A

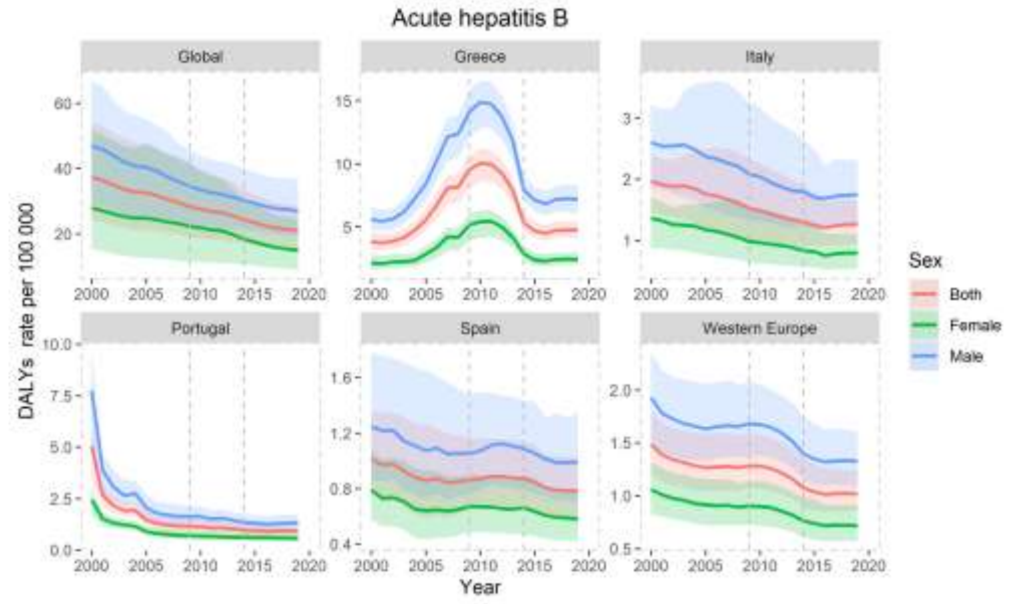

B

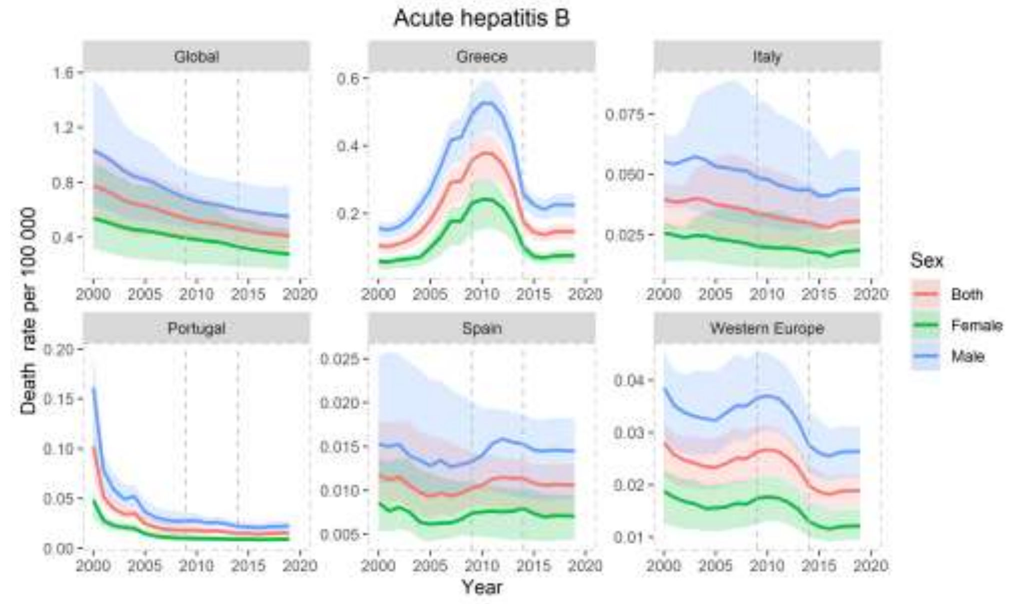

C

D

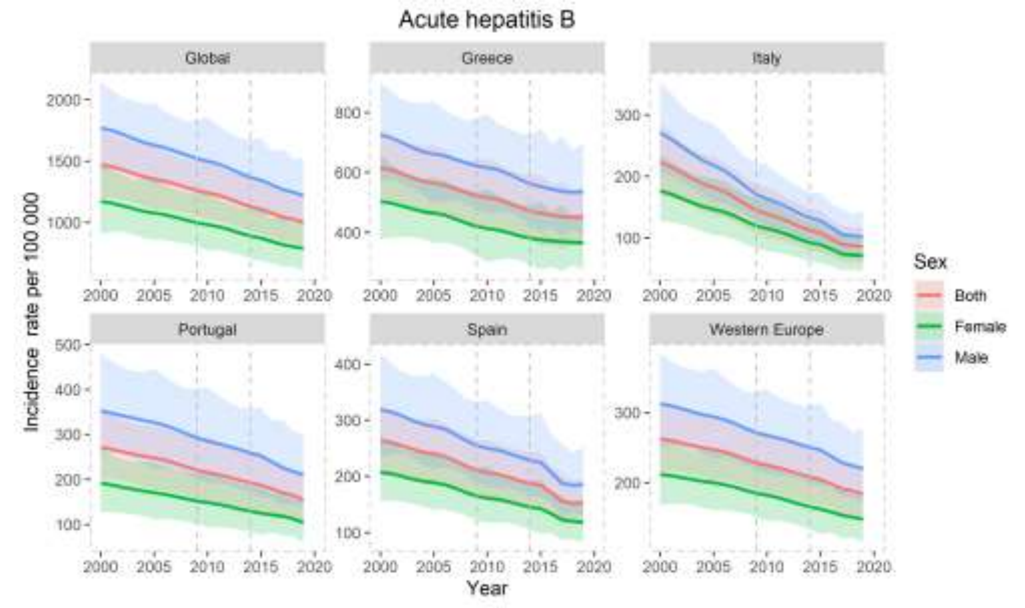
Acute hepatitis B
Global
Greece
Italy
Portugal
Spain
Western Europe
Incidence rate per 100 000
Year
Sex
Both
Female
Male

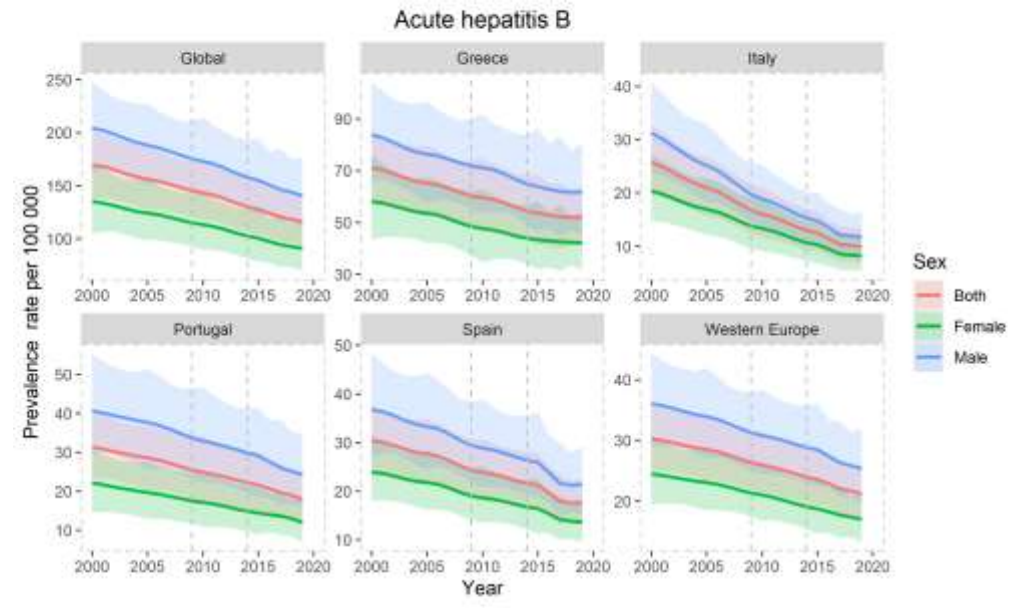
Acute hepatitis B
Global
Greece
Italy
Portugal
Spain
Western Europe
Prevalence rate per 100 000
Year
Sex
Both
Female
Male

E

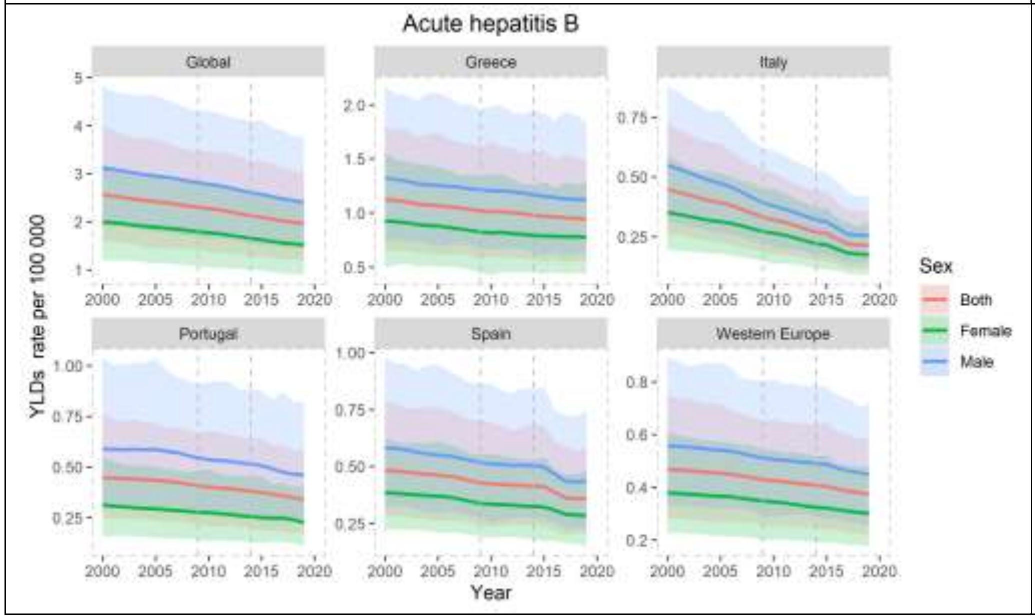
Acute hepatitis B
Global
Greece
Italy
Portugal
Spain
Western Europe
YLDs rate per 100 000
Year
Sex
Both
Female
Male

F

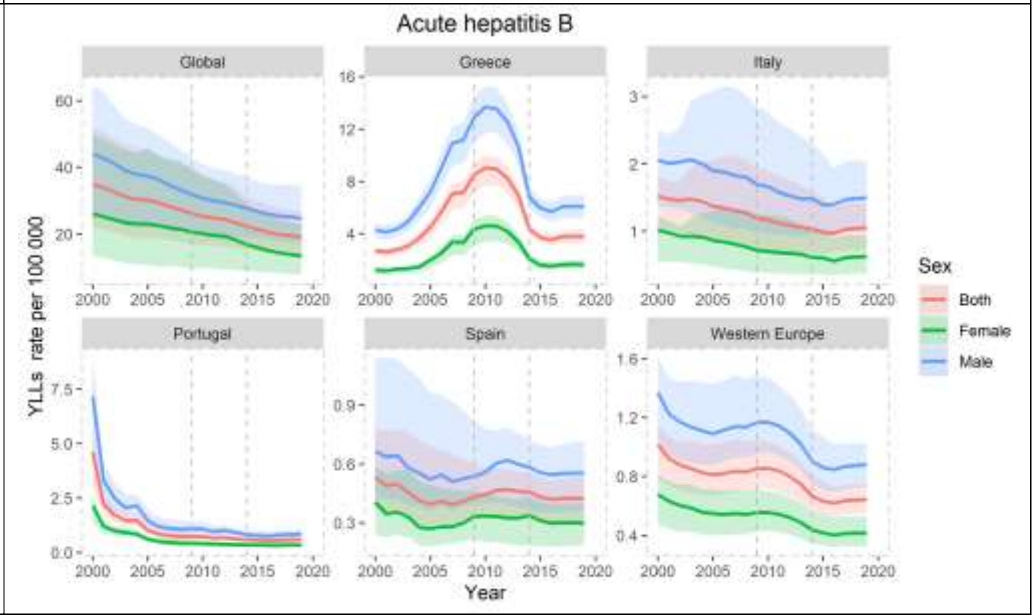
Acute hepatitis B
Global
Greece
Italy
Portugal
Spain
Western Europe
YLLs rate per 100 000
Year
Sex
Both
Female
Male

Figure S2 - Trends of **acute hepatitis B rates** of prevalence, incidence, mortality, years lived with disability (YLDs), years of life lost (YLLs) and disability-adjusted life years (DALYs) per 100,000 population by age group in Greece, Italy, Portugal, Spain and Western Europe from 2000 to 2019 (Global Burden of Disease Study 2019).

A	B
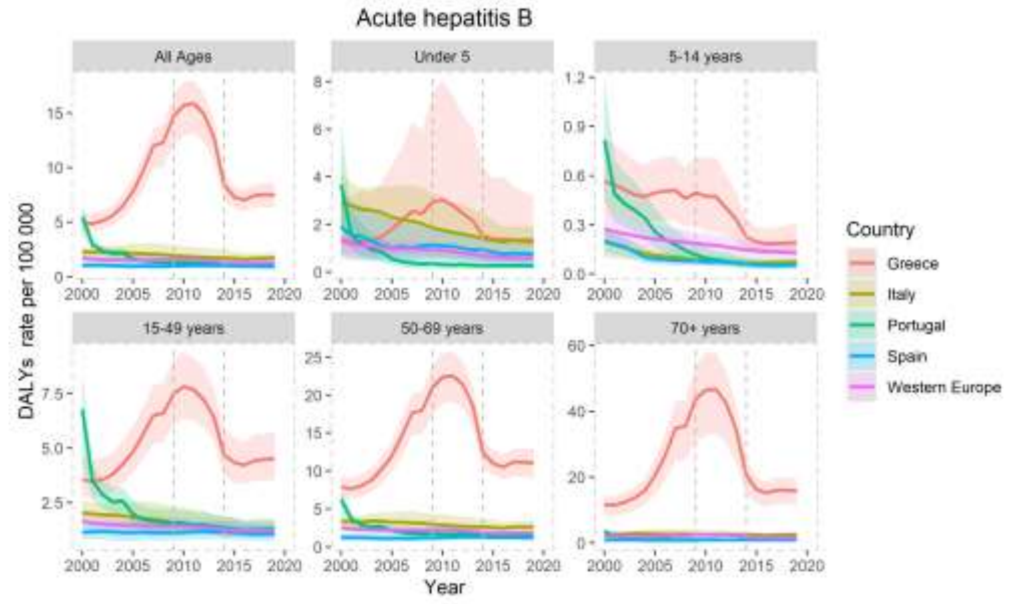	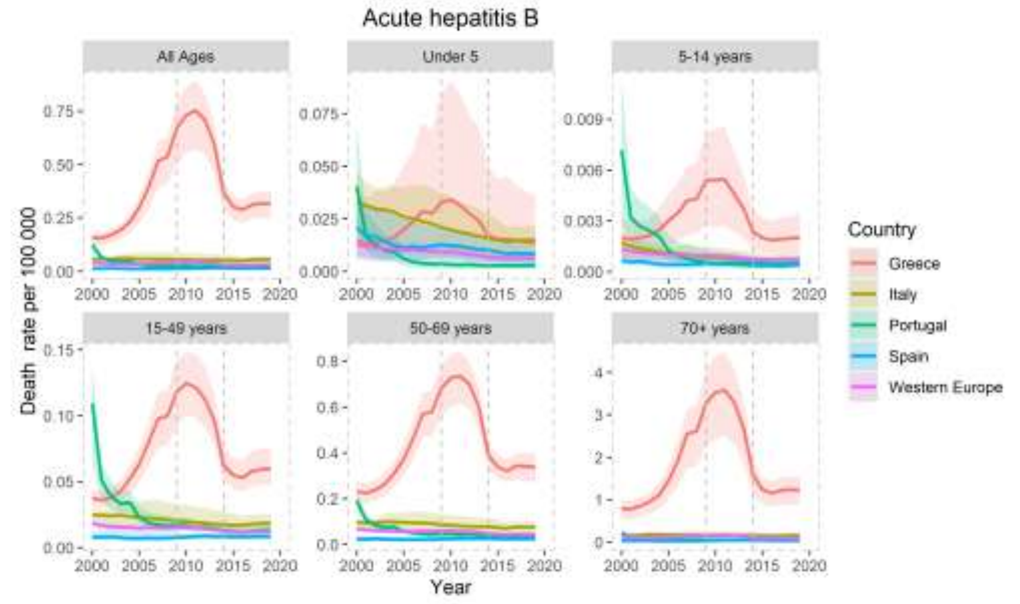
C	D

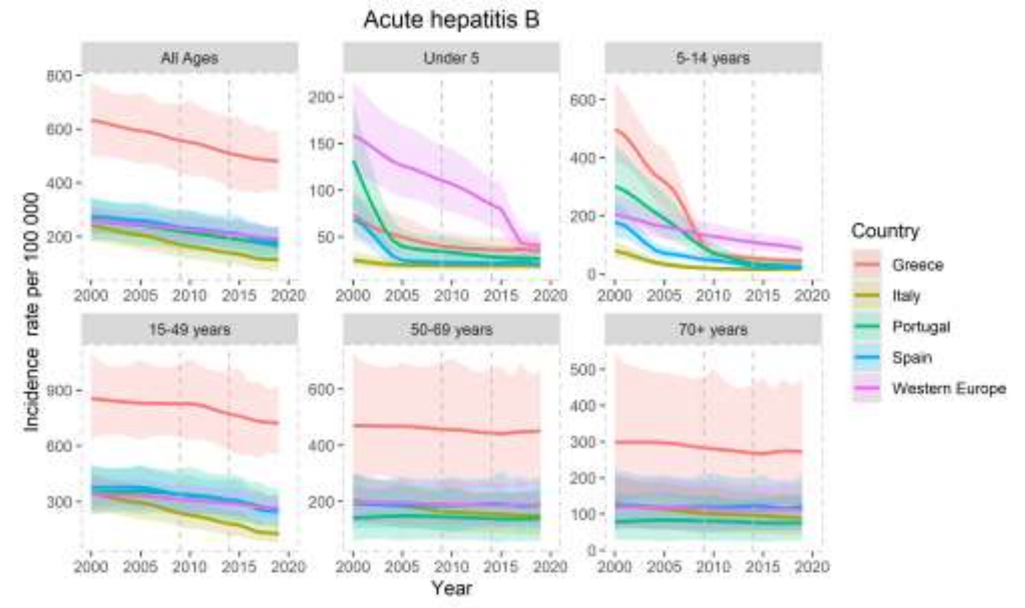
Acute hepatitis B
All Ages
Under 5
5-14 years
15-49 years
50-69 years
70+ years
Incidence rate per 100 000
Year
Country
Greece
Italy
Portugal
Spain
Western Europe

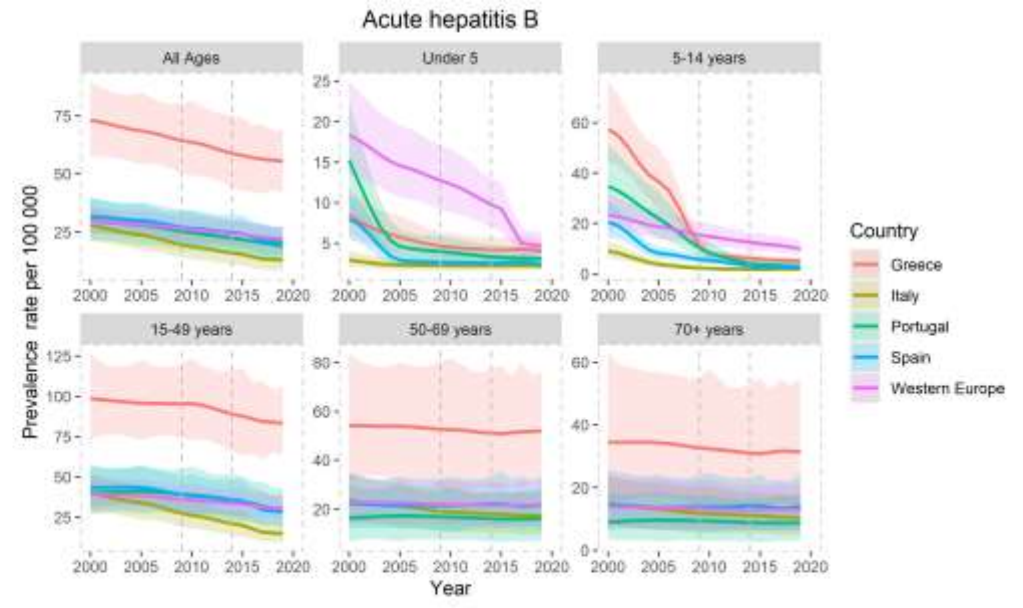
Acute hepatitis B
All Ages
Under 5
5-14 years
15-49 years
50-69 years
70+ years
Prevalence rate per 100 000
Year
Country
Greece
Italy
Portugal
Spain
Western Europe

E

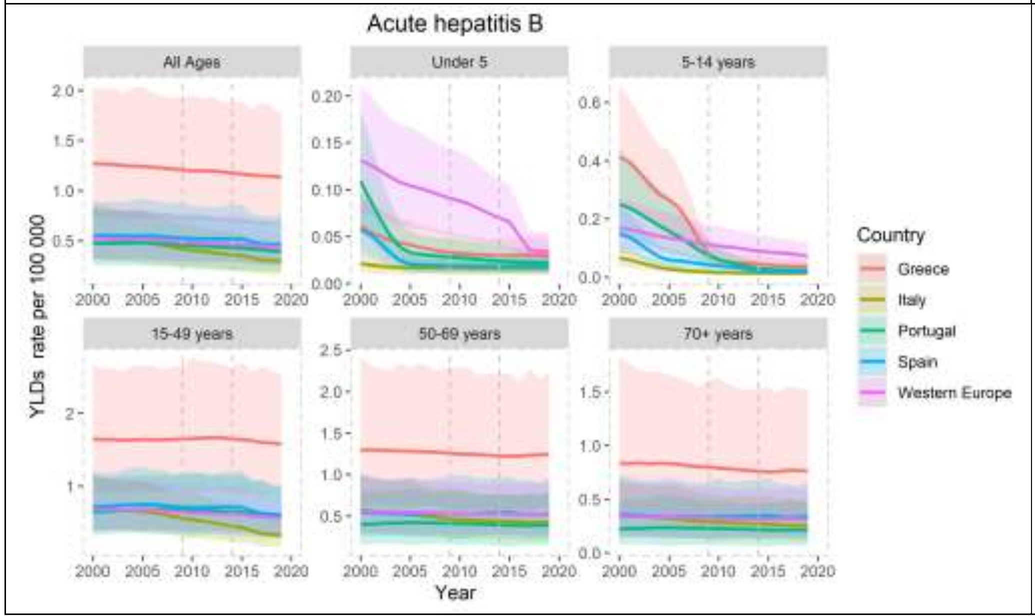
Acute hepatitis B
All Ages
Under 5
5-14 years
15-49 years
50-69 years
70+ years
YLDs rate per 100 000
Year
Country
Greece
Italy
Portugal
Spain
Western Europe

F

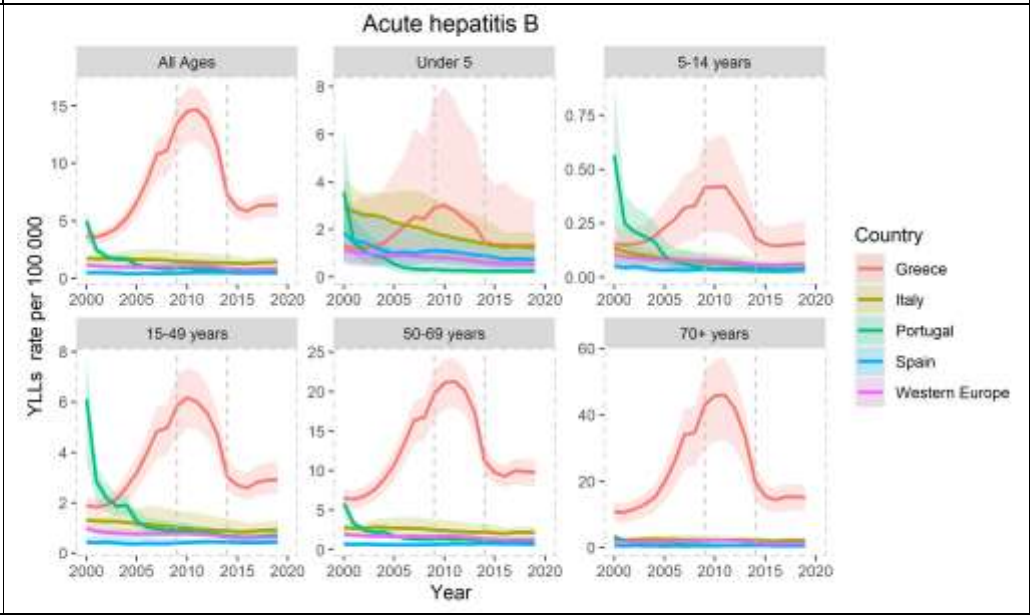
Acute hepatitis B
All Ages
Under 5
5-14 years
15-49 years
50-69 years
70+ years
YLLs rate per 100 000
Year
Country
Greece
Italy
Portugal
Spain
Western Europe

Figure S3 - Trends of **acute hepatitis C age-standardized rates** of prevalence, incidence, mortality, years lived with disability (YLDs), years of life lost (YLLs) and disability-adjusted life years (DALYs) per 100,000 population by sex in Greece, Italy, Portugal, Spain, Western Europe from 2000 to 2019 (Global Burden of Disease Study 2019).

A

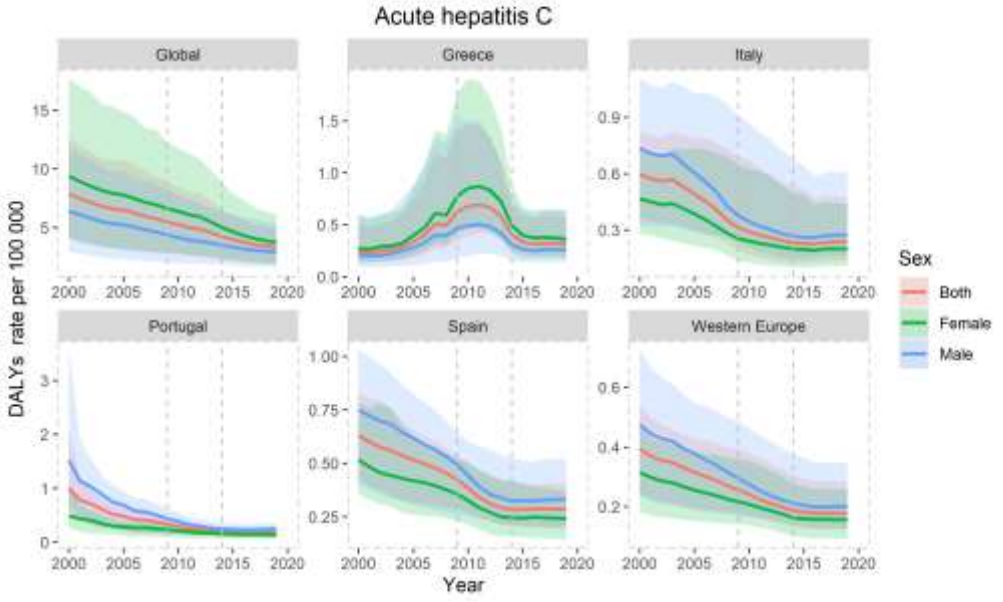

B

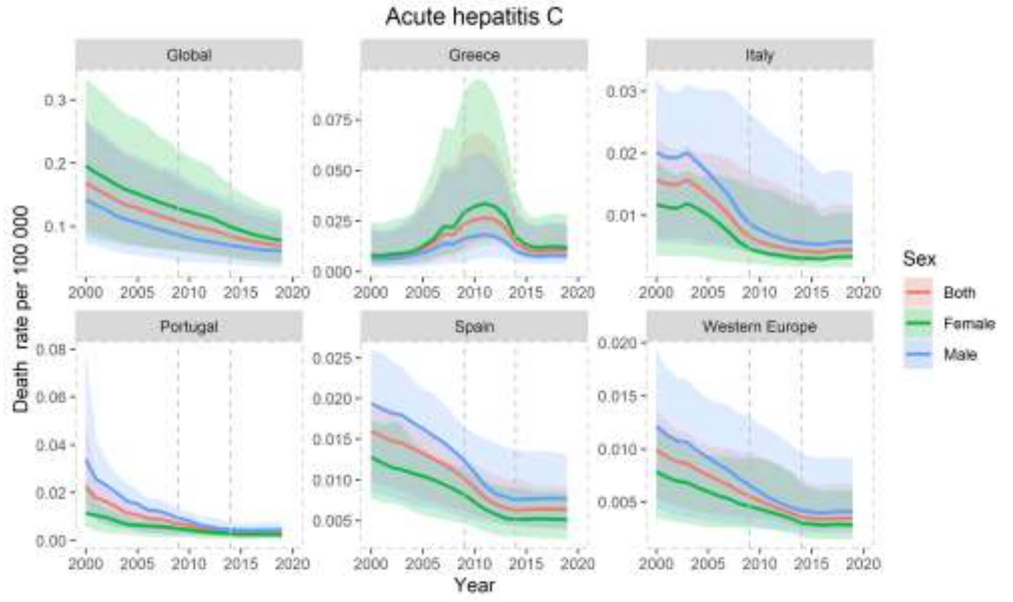

C

D

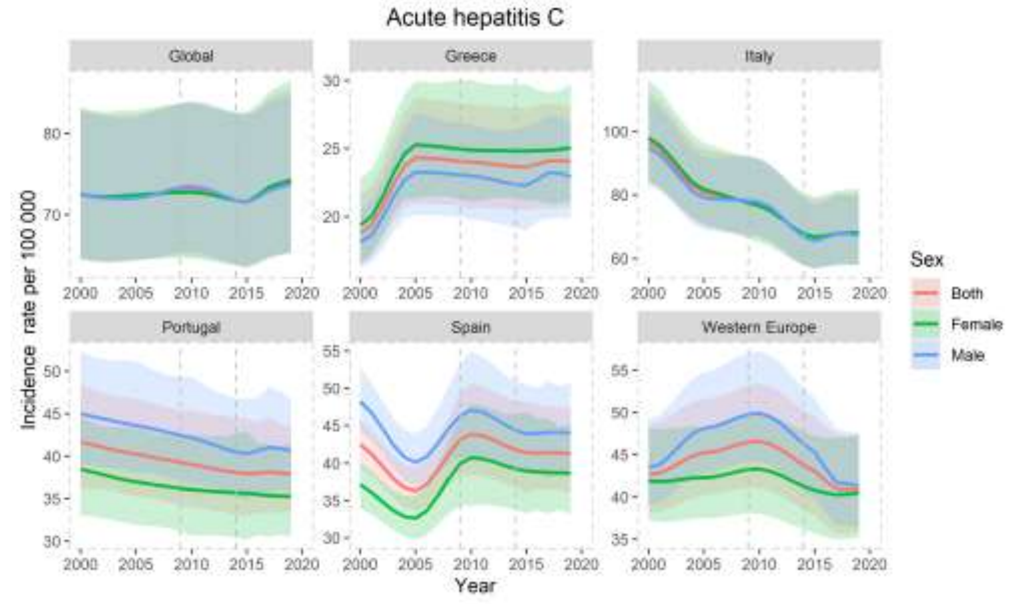
Acute hepatitis C
Global
Greece
Italy
Portugal
Spain
Western Europe
Incidence rate per 100 000
Year
Sex
Both
Female
Male

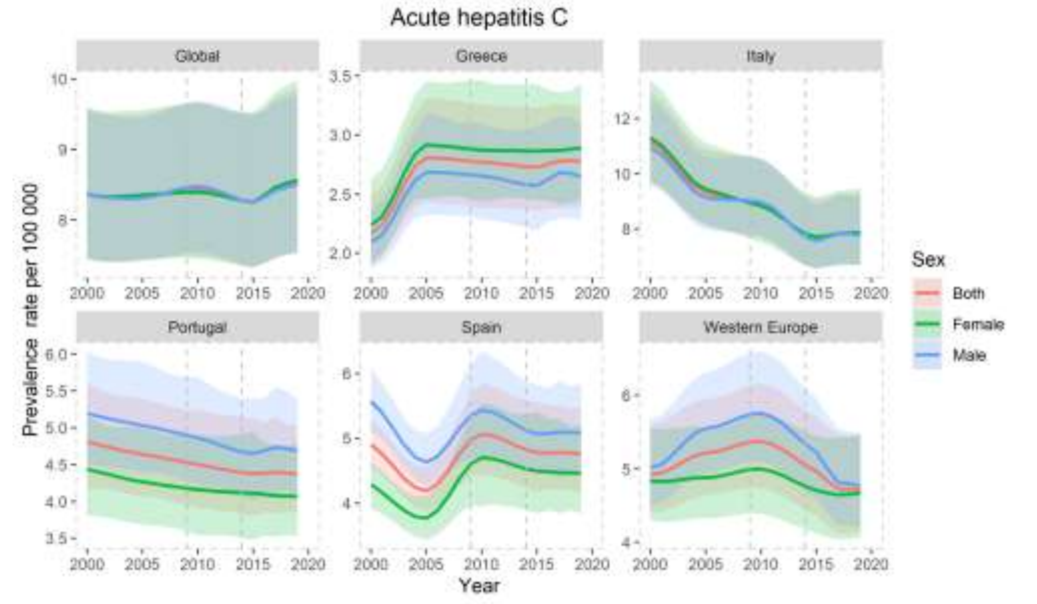
Acute hepatitis C
Global
Greece
Italy
Portugal
Spain
Western Europe
Prevalence rate per 100 000
Year
Sex
Both
Female
Male

E

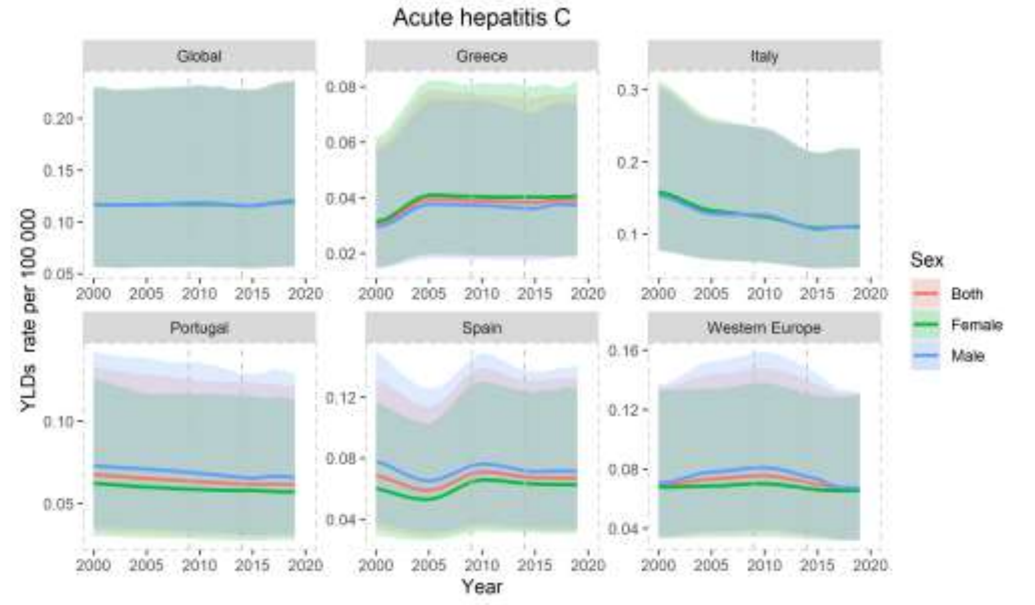
Acute hepatitis C
Global
Greece
Italy
Portugal
Spain
Western Europe
YLDs rate per 100 000
Year
Sex
Both
Female
Male

F

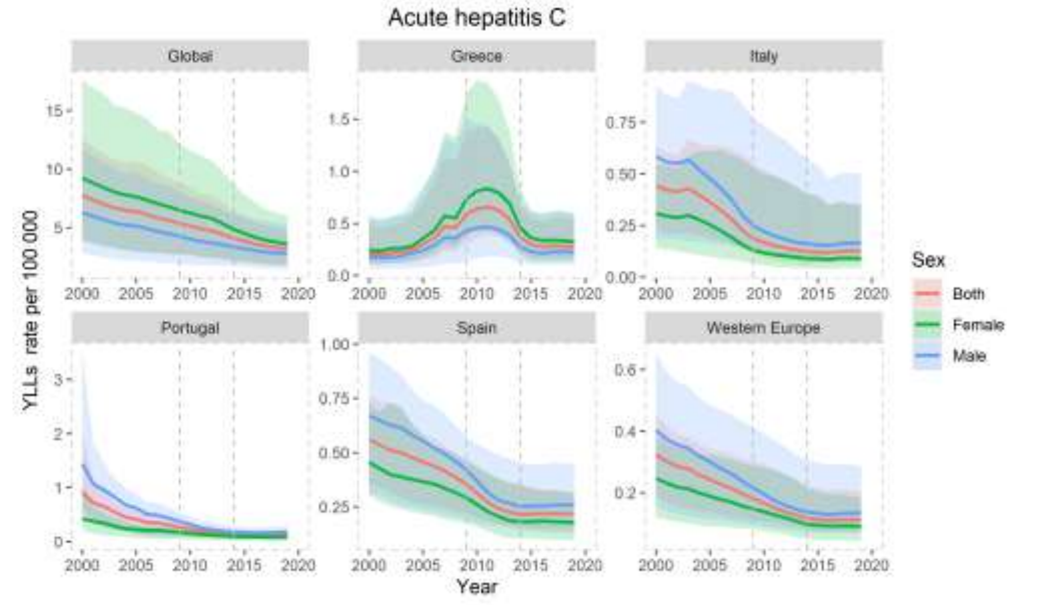
Acute hepatitis C
Global
Greece
Italy
Portugal
Spain
Western Europe
YLLs rate per 100 000
Year
Sex
Both
Female
Male

Figure S4 - Trends of **acute hepatitis C rates** of prevalence, incidence, mortality, years lived with disability (YLDs), years of life lost (YLLs) and disability-adjusted life years (DALYs) per 100,000 population by age group in Greece, Italy, Portugal, Spain and Western Europe from 2000 to 2019 (Global Burden of Disease Study 2019).

A

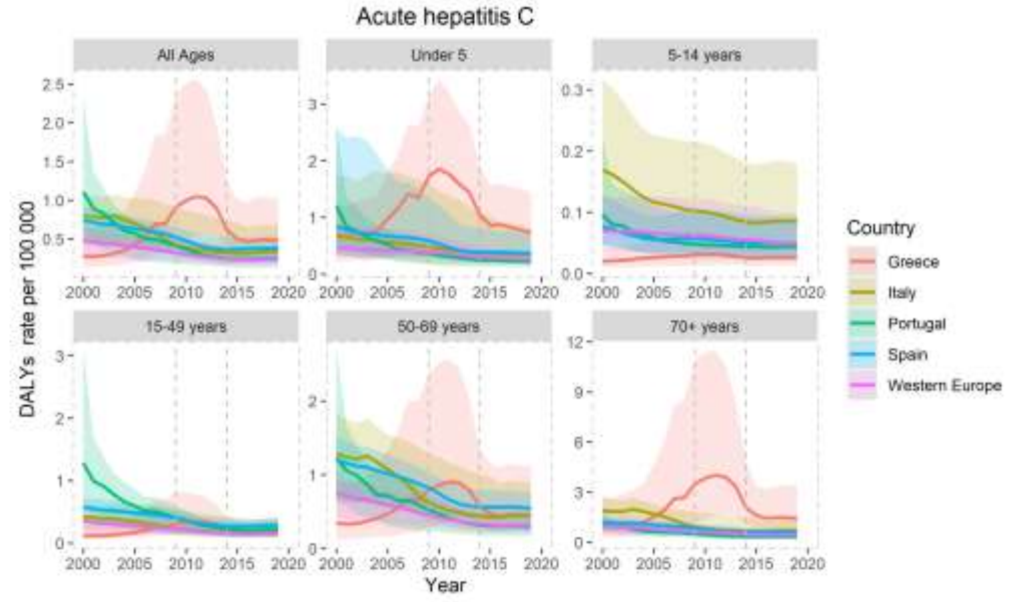

B

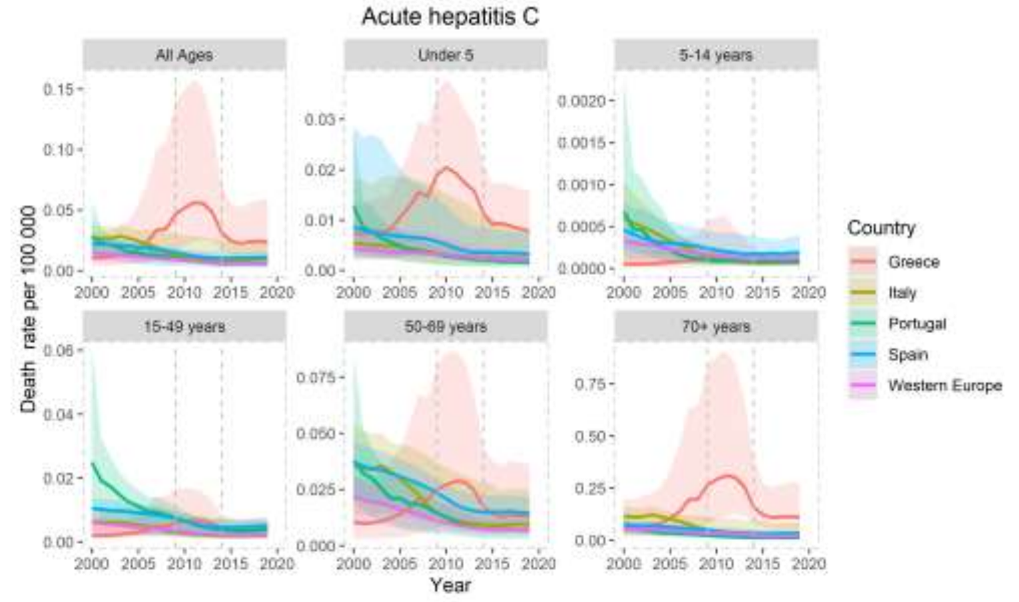

C

D

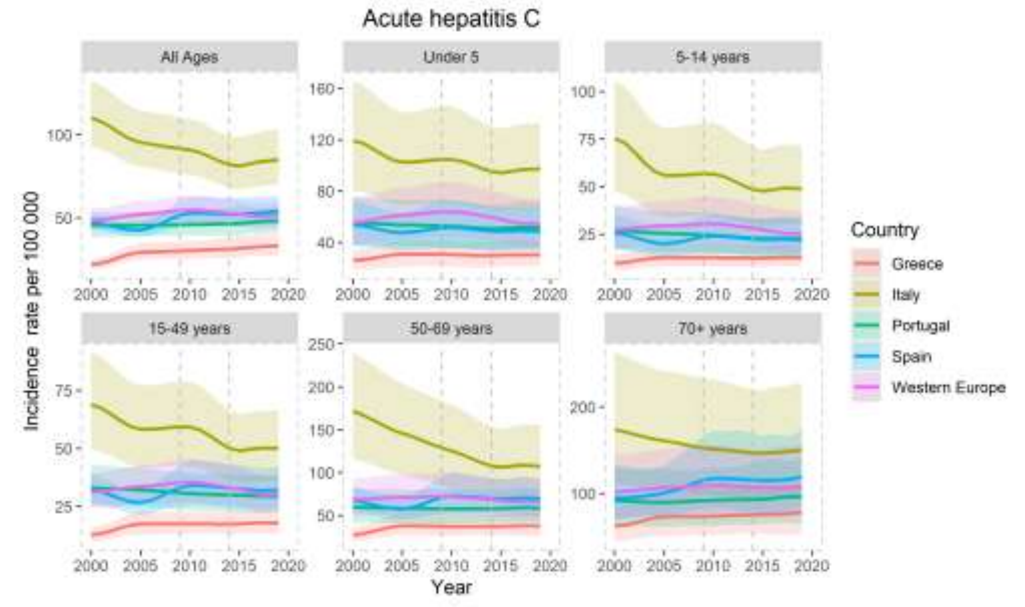
Acute hepatitis C
All Ages
Under 5
5-14 years
15-49 years
50-69 years
70+ years
Incidence rate per 100 000
Year
Country
Greece
Italy
Portugal
Spain
Western Europe

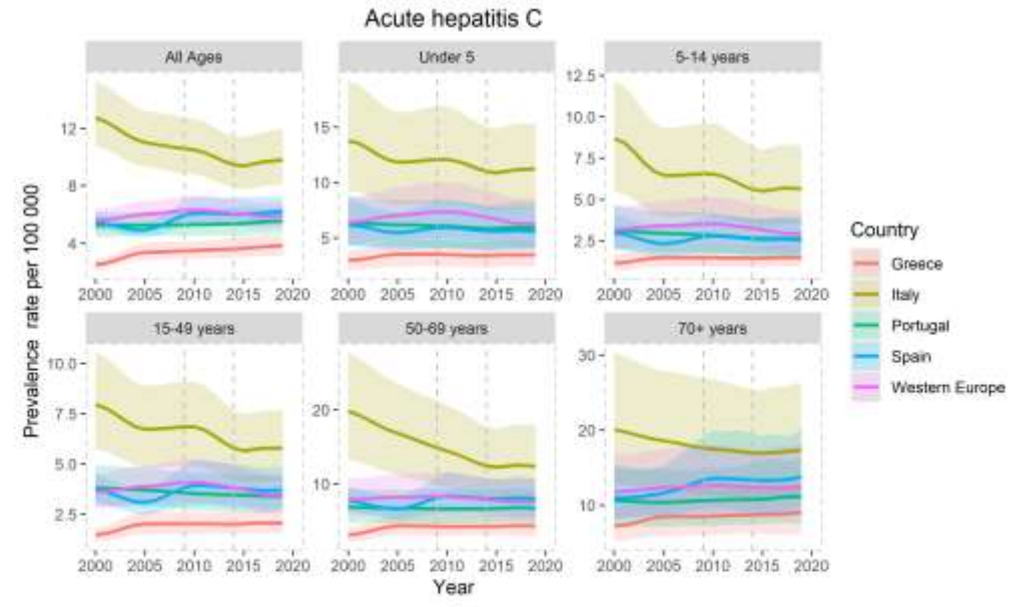
Acute hepatitis C
All Ages
Under 5
5-14 years
15-49 years
50-69 years
70+ years
Prevalence rate per 100 000
Year
Country
Greece
Italy
Portugal
Spain
Western Europe

E

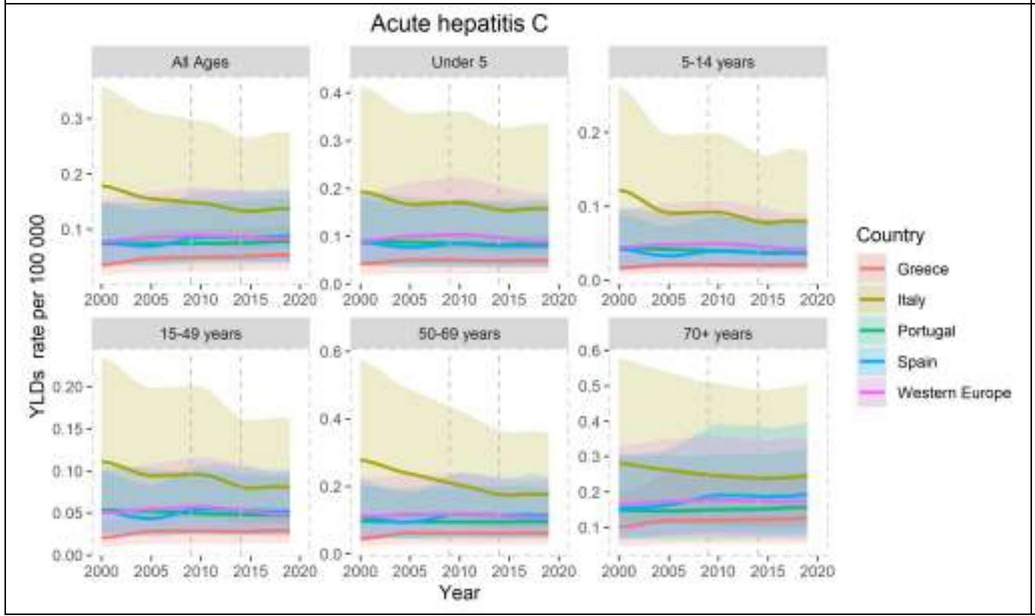
Acute hepatitis C
All Ages
Under 5
5-14 years
15-49 years
50-69 years
70+ years
YLDs rate per 100 000
Year
Country
Greece
Italy
Portugal
Spain
Western Europe

F

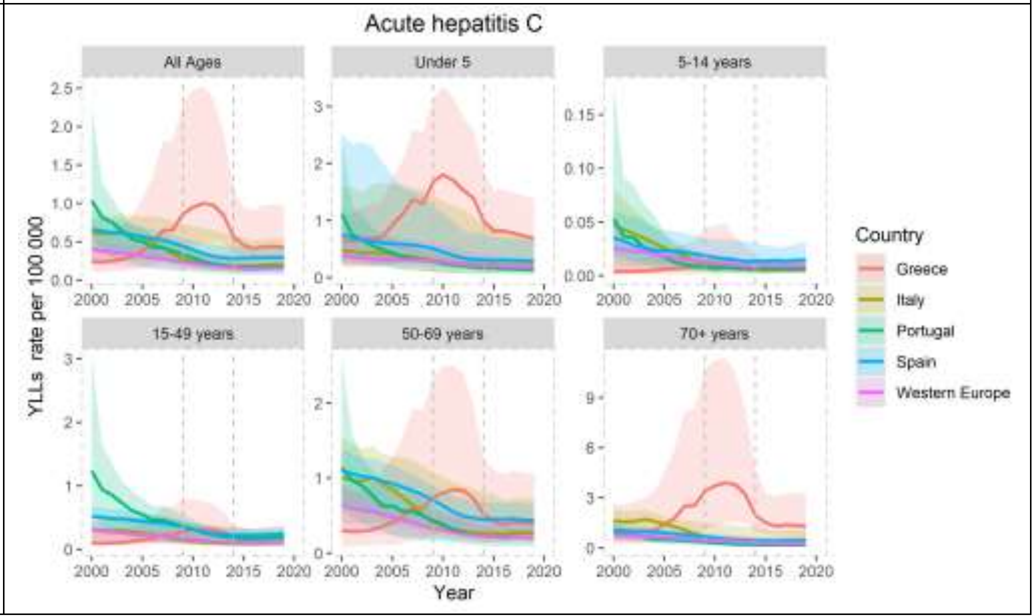
Acute hepatitis C
All Ages
Under 5
5-14 years
15-49 years
50-69 years
70+ years
YLLs rate per 100 000
Year
Country
Greece
Italy
Portugal
Spain
Western Europe

Figure S5 - Trends of **age-standardized rates** of **cirrhosis and other chronic liver diseases due to hepatitis B** prevalence, incidence, mortality, years lived with disability (YLDs), years of life lost (YLLs) and disability-adjusted life years (DALYs) per 100,000 population by sex in Greece, Italy, Portugal, Spain, Western Europe from 2000 to 2019 (Global Burden of Disease Study 2019).

A	B
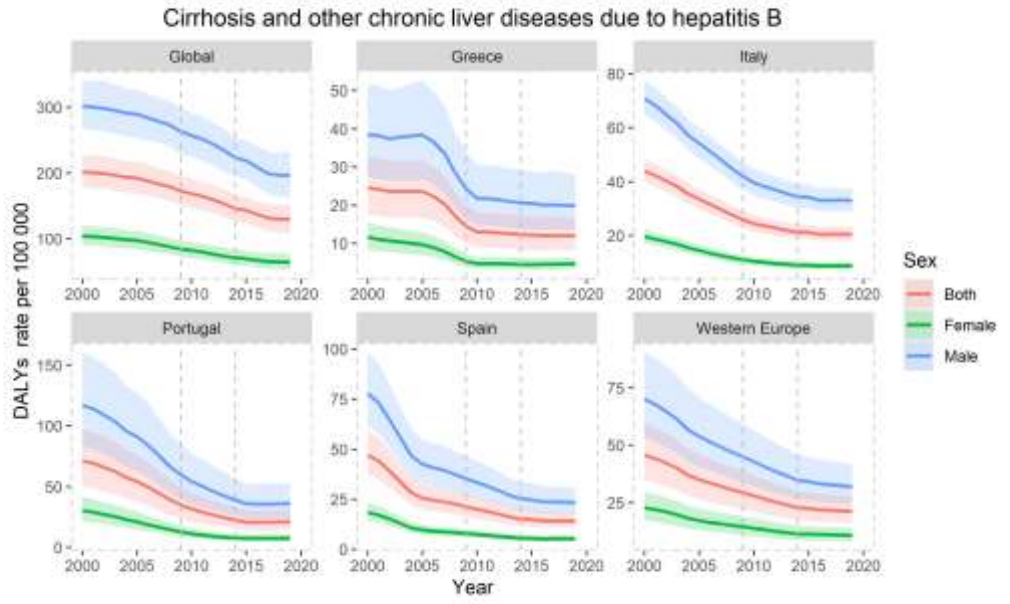	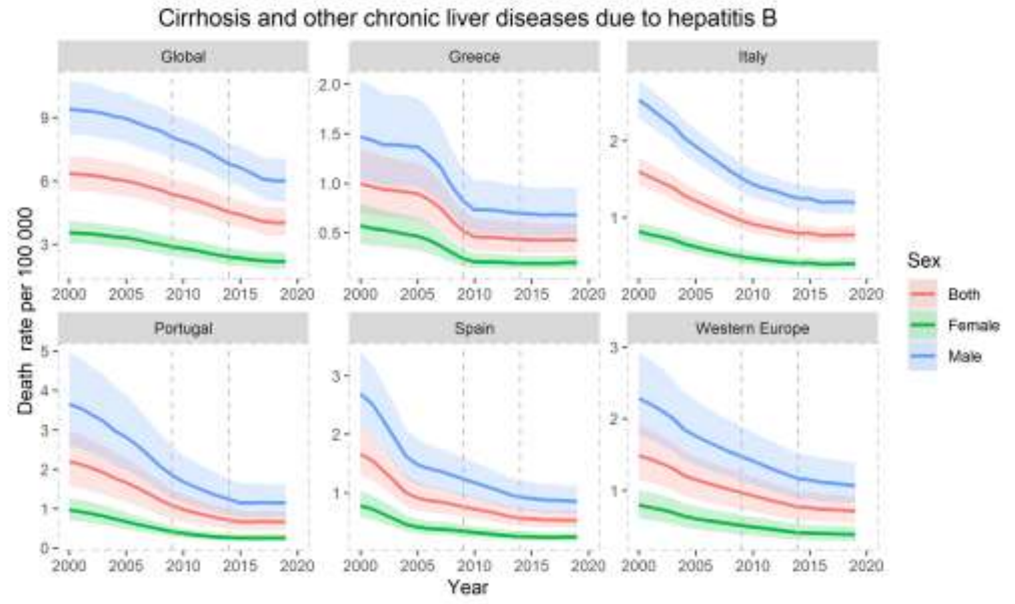
C	D

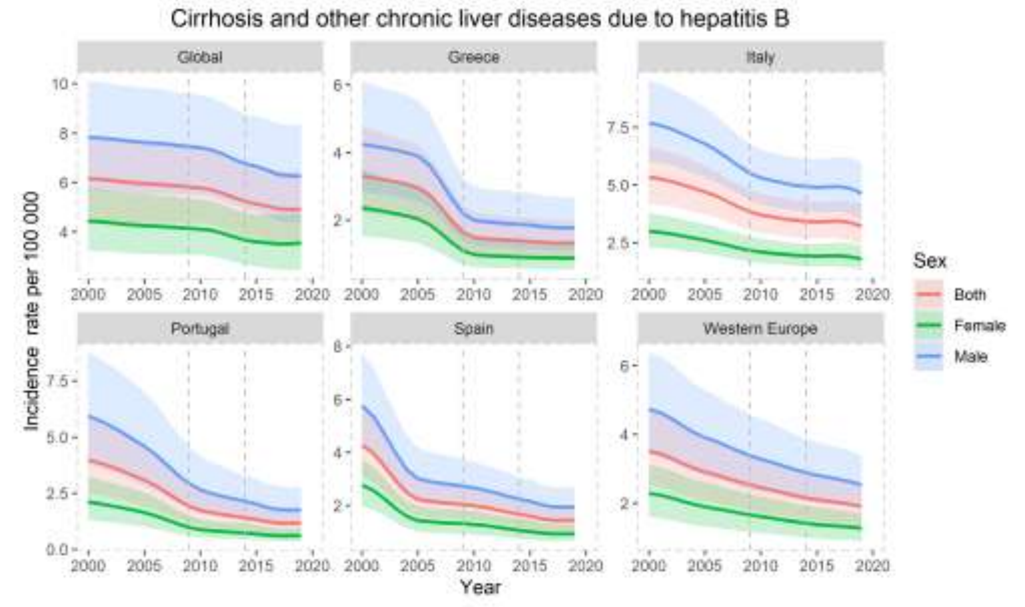
Cirrhosis and other chronic liver diseases due to hepatitis B
Global
Greece
Italy
Portugal
Spain
Western Europe
Incidence rate per 100 000
Year
Sex
Both
Female
Male

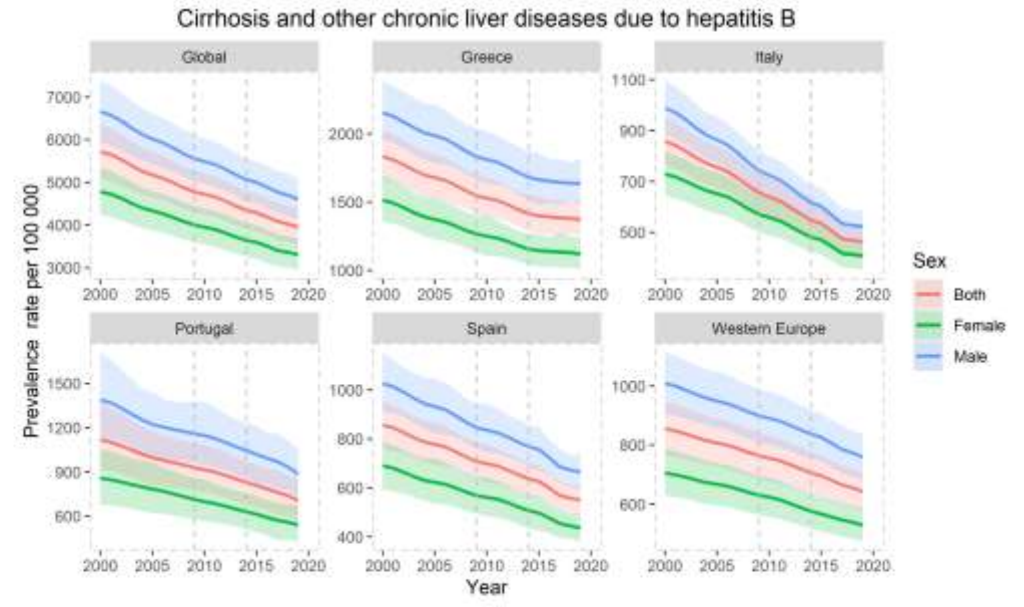
Cirrhosis and other chronic liver diseases due to hepatitis B
Global
Greece
Italy
Portugal
Spain
Western Europe
Prevalence rate per 100 000
Year
Sex
Both
Female
Male

E

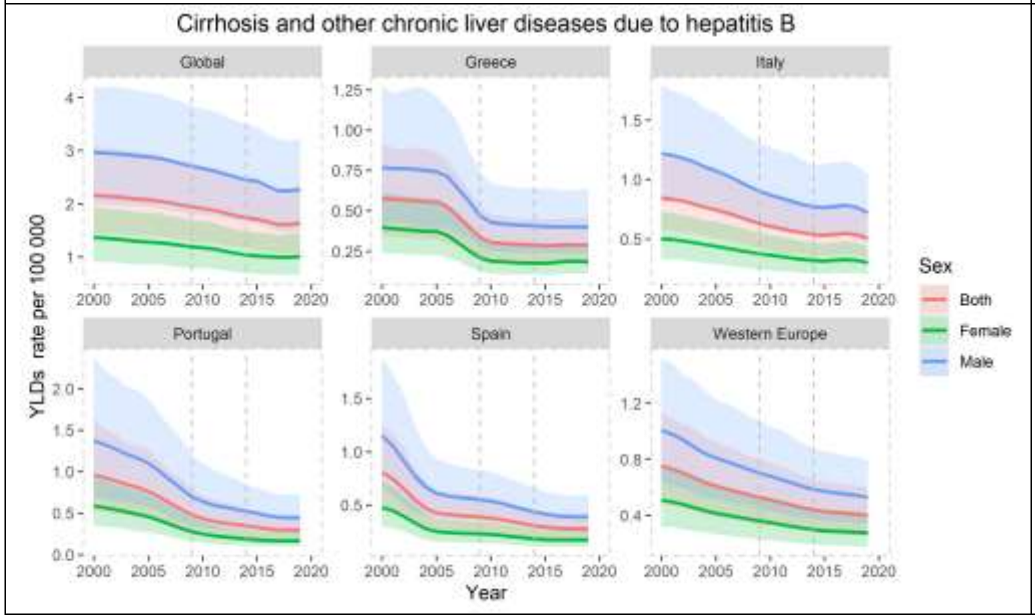
Cirrhosis and other chronic liver diseases due to hepatitis B
Global
Greece
Italy
Portugal
Spain
Western Europe
YLDs rate per 100 000
Year
Sex
Both
Female
Male

F

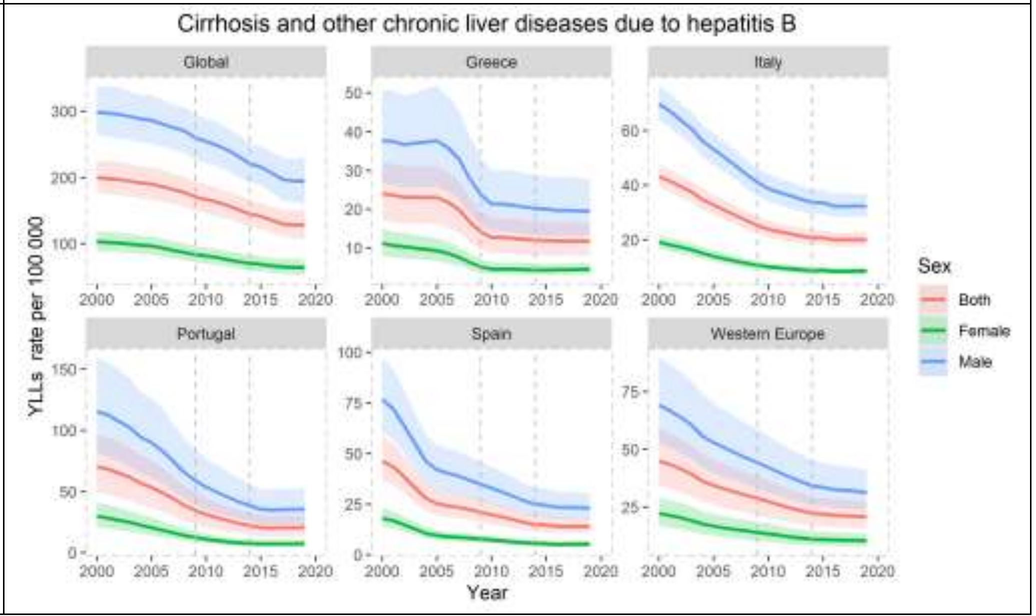
Cirrhosis and other chronic liver diseases due to hepatitis B
Global
Greece
Italy
Portugal
Spain
Western Europe
YLLs rate per 100 000
Year
Sex
Both
Female
Male

Figure S6 - Trends of **rates** of **cirrhosis and other chronic liver diseases due to hepatitis B** prevalence, incidence, mortality, years lived with disability (YLDs), years of life lost (YLLs) and disability-adjusted life years (DALYs) per 100,000 population by age group in Greece, Italy, Portugal, Spain and Western Europe from 2000 to 2019 (Global Burden of Disease Study 2019).

**A**	**B**
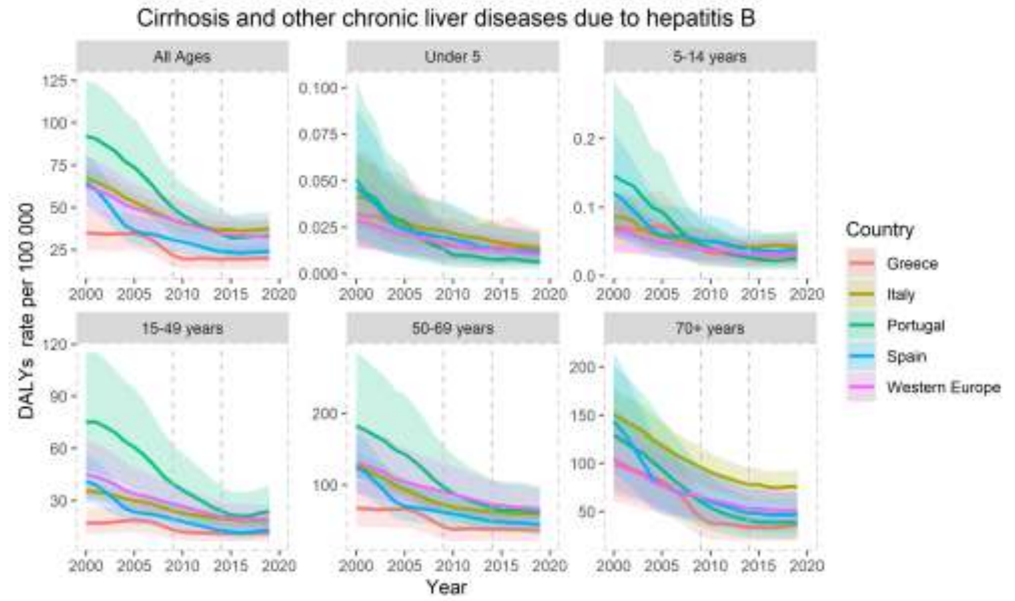	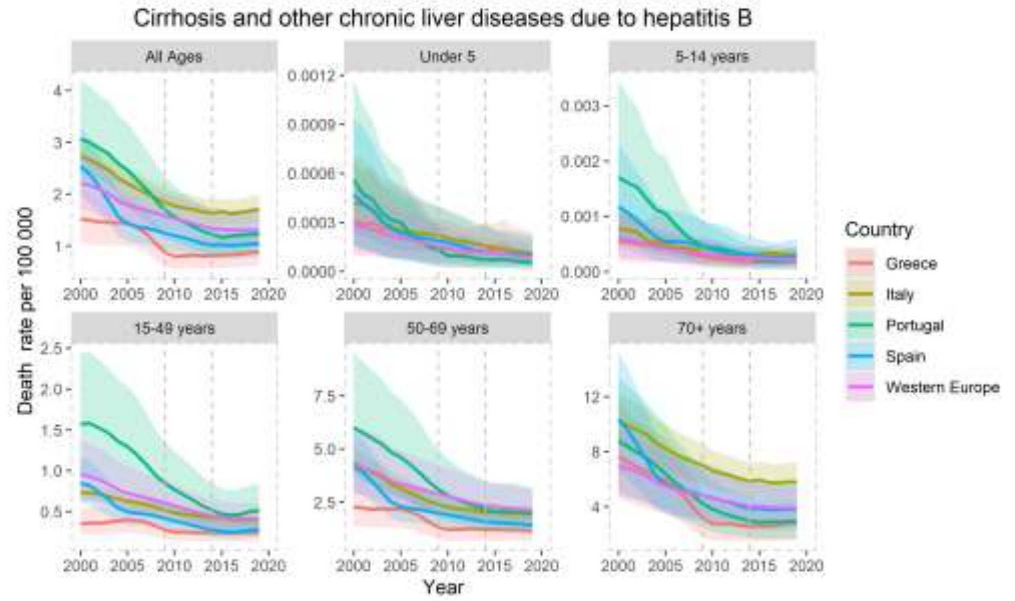
**C**	**D**

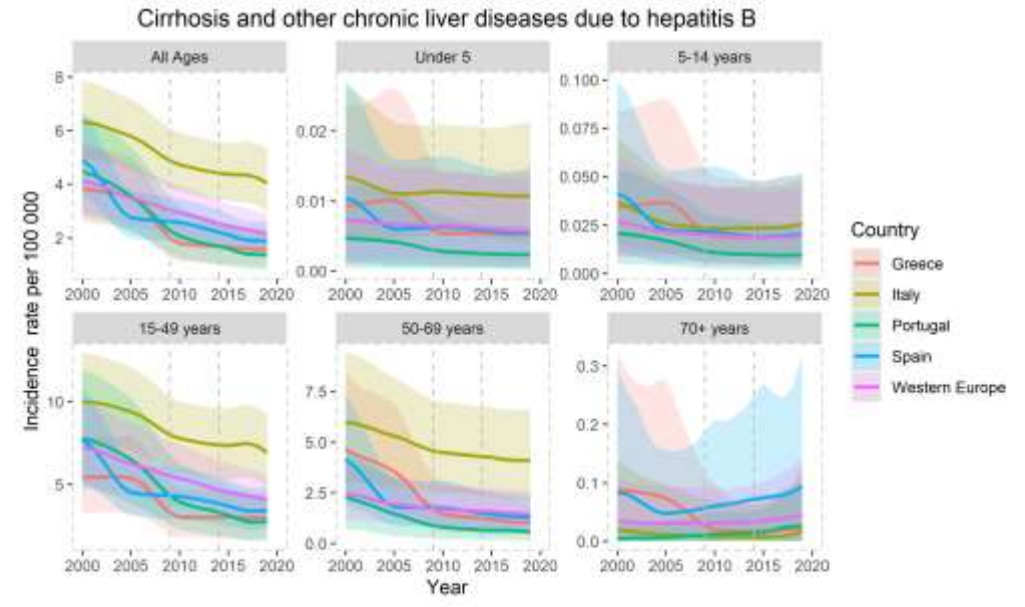
Cirrhosis and other chronic liver diseases due to hepatitis B
All Ages
Under 5
5-14 years
15-49 years
50-69 years
70+ years
Incidence rate per 100 000
Year
Country
Greece
Italy
Portugal
Spain
Western Europe

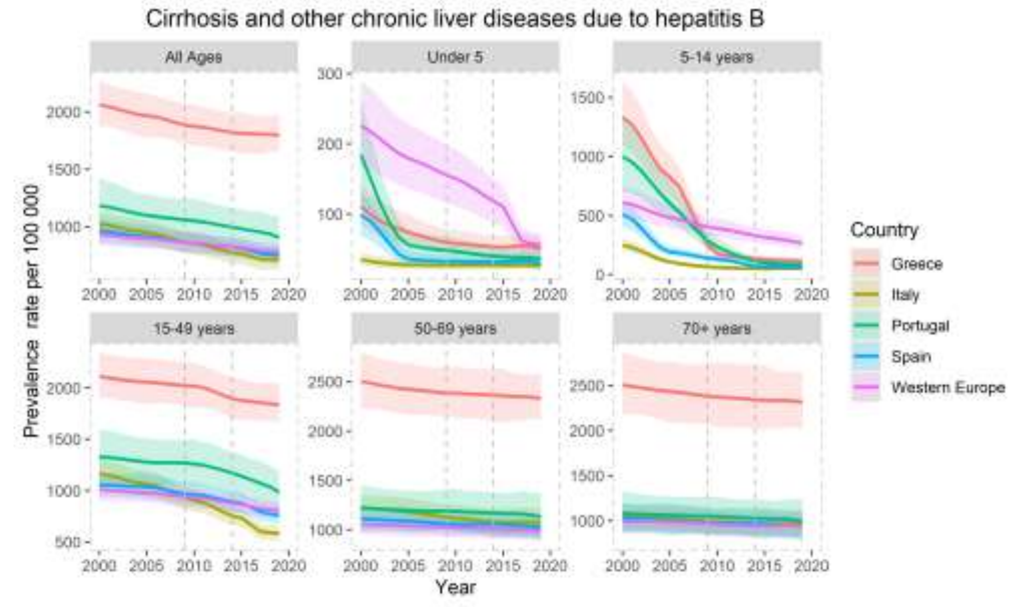
Cirrhosis and other chronic liver diseases due to hepatitis B
All Ages
Under 5
5-14 years
15-49 years
50-69 years
70+ years
Prevalence rate per 100 000
Year
Country
Greece
Italy
Portugal
Spain
Western Europe

E

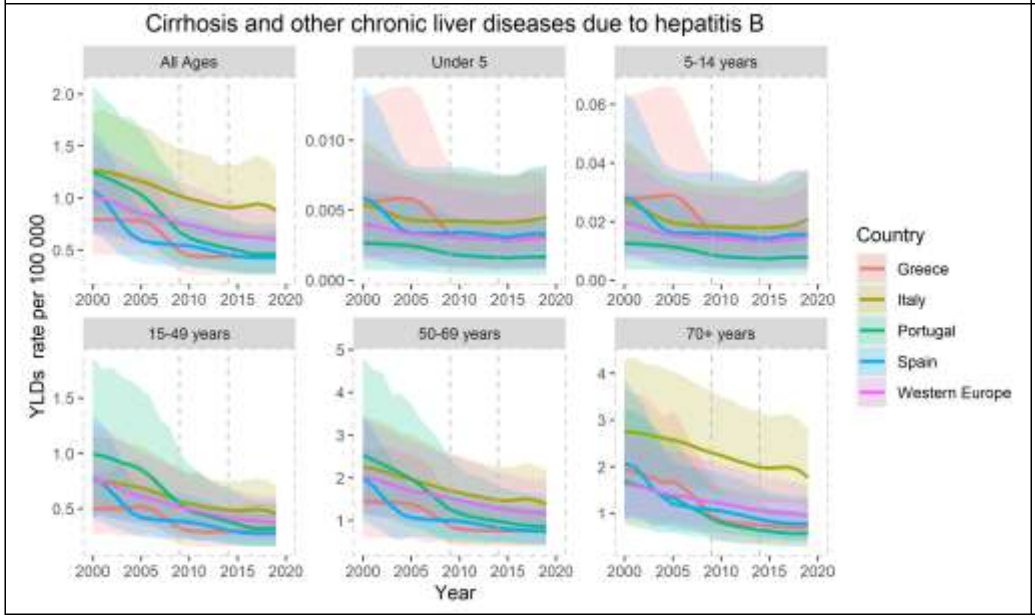
Cirrhosis and other chronic liver diseases due to hepatitis B
All Ages
Under 5
5-14 years
15-49 years
50-69 years
70+ years
YLDs rate per 100 000
Year
Country
Greece
Italy
Portugal
Spain
Western Europe

F

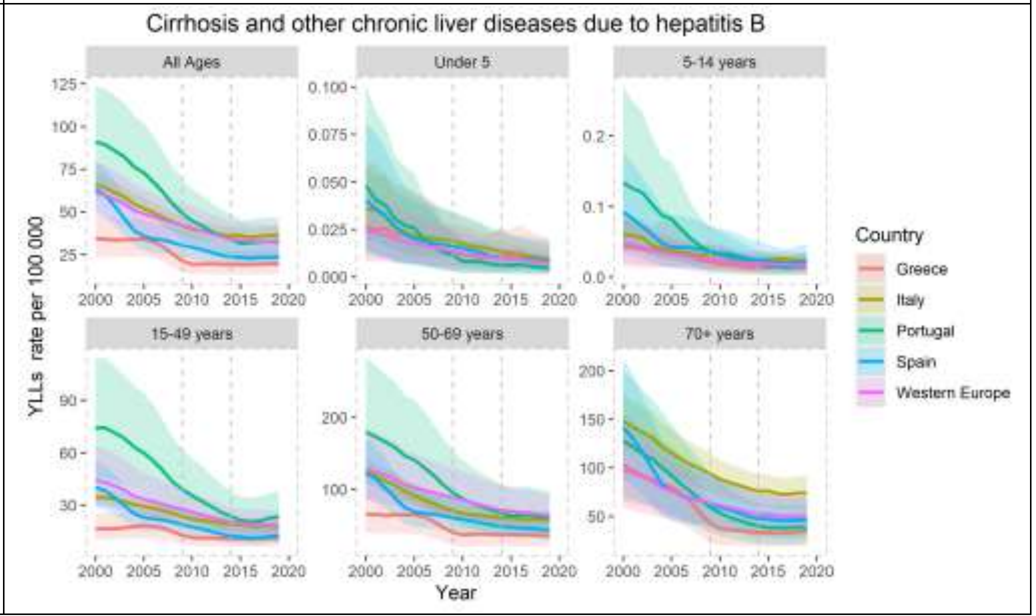
Cirrhosis and other chronic liver diseases due to hepatitis B
All Ages
Under 5
5-14 years
15-49 years
50-69 years
70+ years
YLLs rate per 100 000
Year
Country
Greece
Italy
Portugal
Spain
Western Europe

Figure S7 - Trends of **age-standardized rates** of **cirrhosis and other chronic liver diseases due to hepatitis C** prevalence, incidence, mortality, years lived with disability (YLDs), years of life lost (YLLs) and disability-adjusted life years (DALYs) per 100,000 population by sex in Greece, Italy, Portugal, Spain, Western Europe from 2000 to 2019 (Global Burden of Disease Study 2019).

A	B
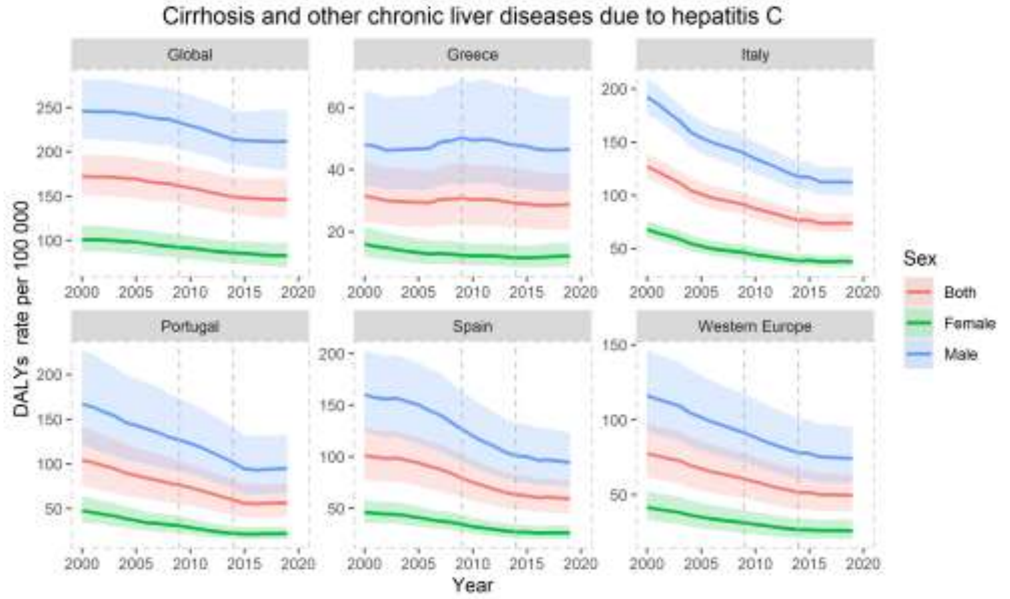	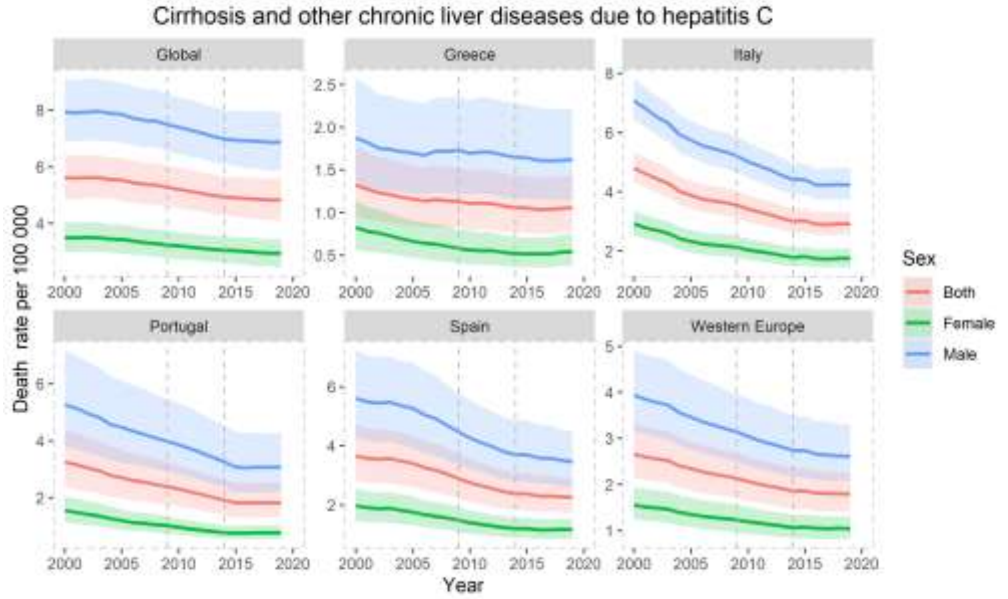
C	D

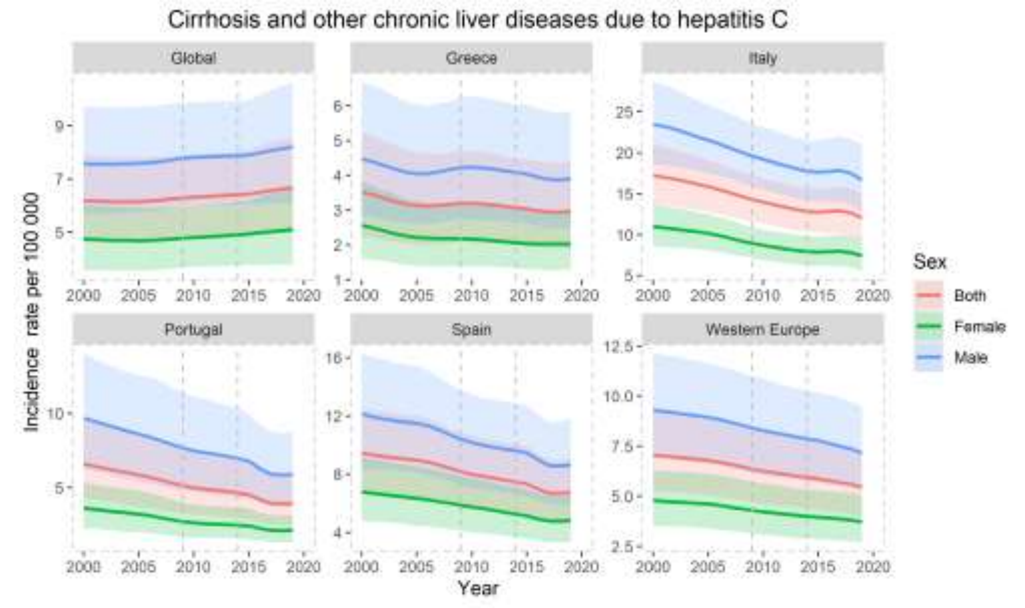
Cirrhosis and other chronic liver diseases due to hepatitis C
Global
Greece
Italy
Portugal
Spain
Western Europe
Incidence rate per 100 000
Year
Sex
Both
Female
Male

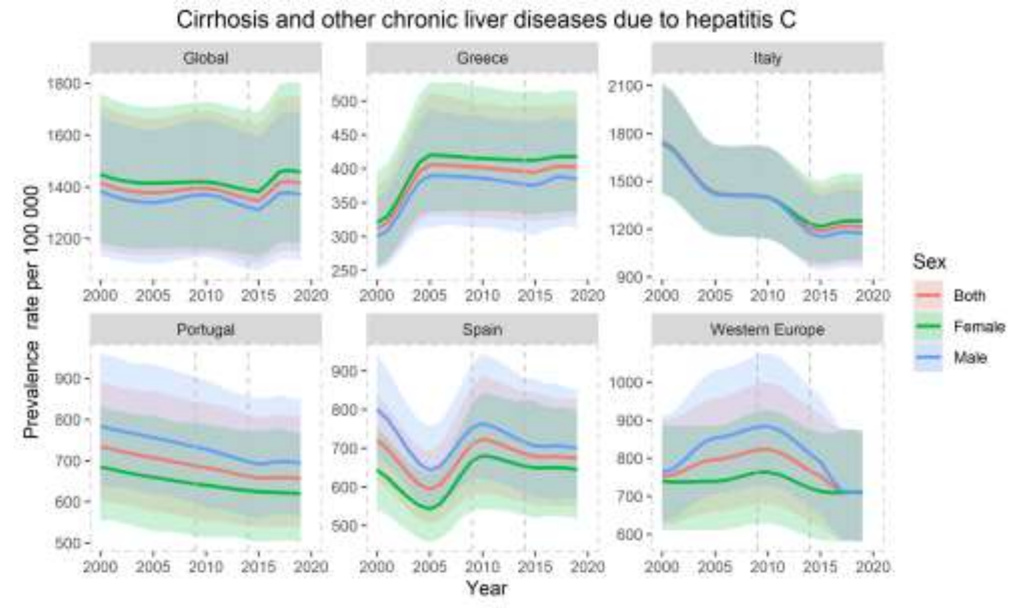
Cirrhosis and other chronic liver diseases due to hepatitis C
Global
Greece
Italy
Portugal
Spain
Western Europe
Prevalence rate per 100 000
Year
Sex
Both
Female
Male

E

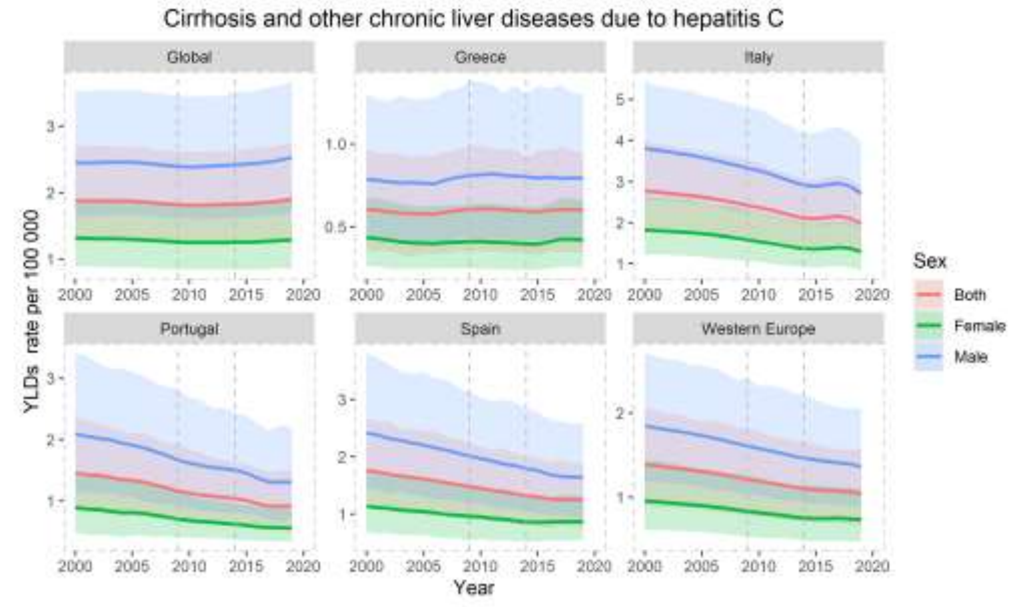
Cirrhosis and other chronic liver diseases due to hepatitis C
Global
Greece
Italy
Portugal
Spain
Western Europe
YLDs rate per 100 000
Year
Sex
Both
Female
Male

F

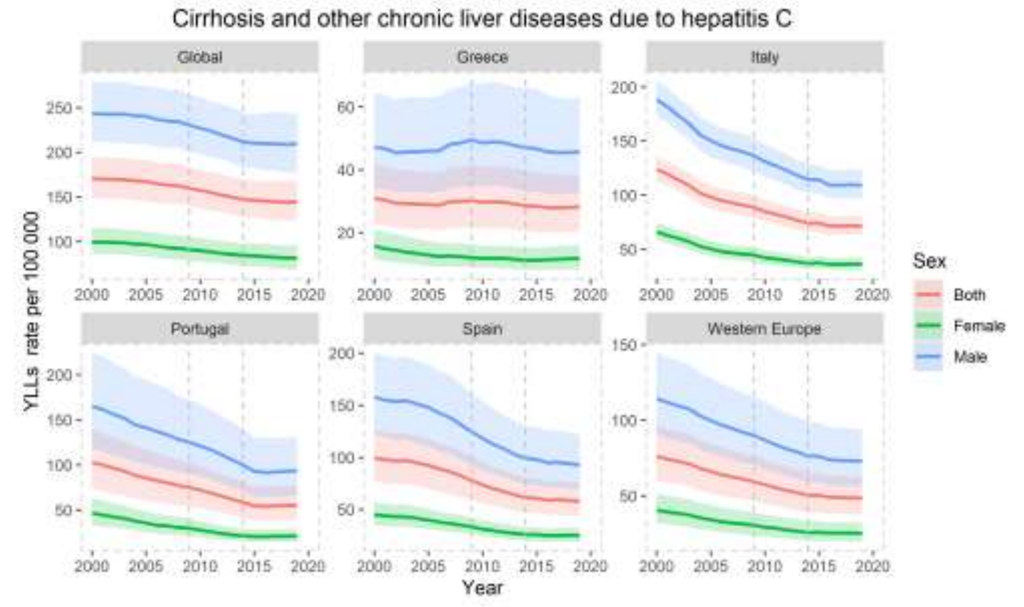
Cirrhosis and other chronic liver diseases due to hepatitis C
Global
Greece
Italy
Portugal
Spain
Western Europe
YLLs rate per 100 000
Year
Sex
Both
Female
Male

Figure S8 - Trends of **rates** of **cirrhosis and other chronic liver diseases due to hepatitis C** prevalence, incidence, mortality, years lived with disability (YLDs), years of life lost (YLLs) and disability-adjusted life years (DALYs) per 100,000 population by age group in Greece, Italy, Portugal, Spain and Western Europe from 2000 to 2019 (Global Burden of Disease Study 2019).

A

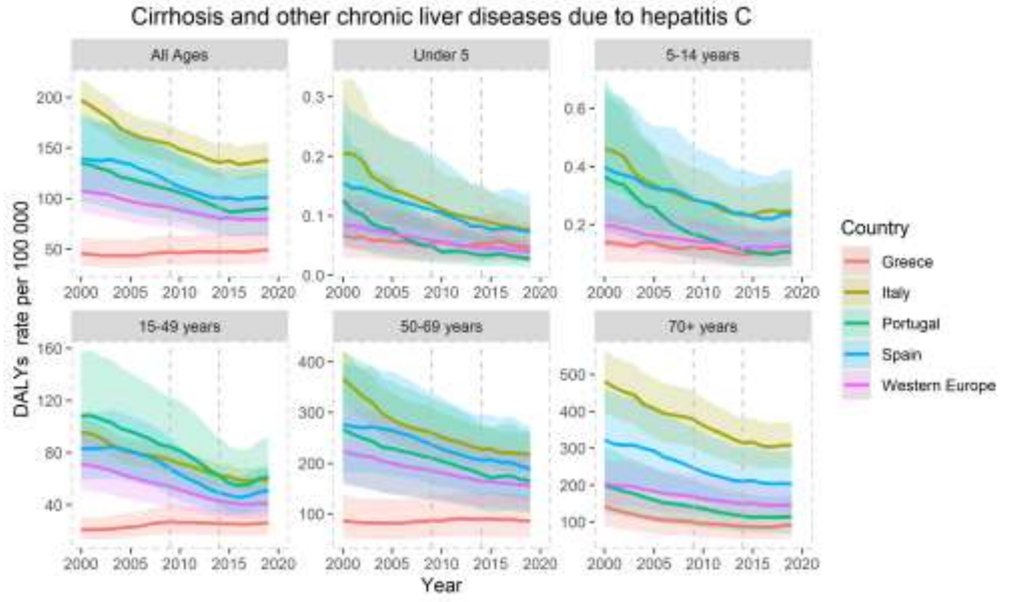

B

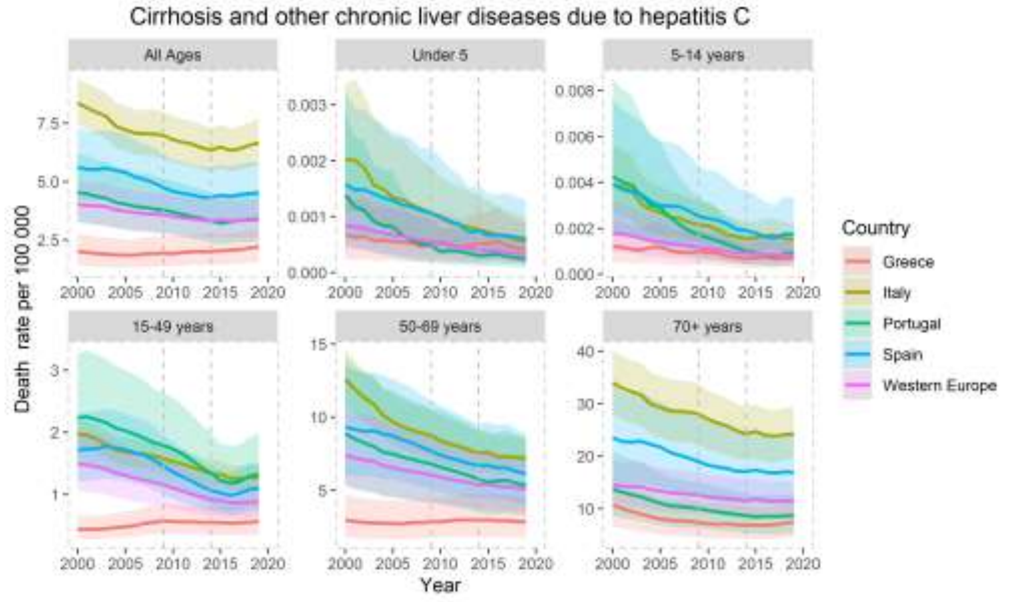

C

D

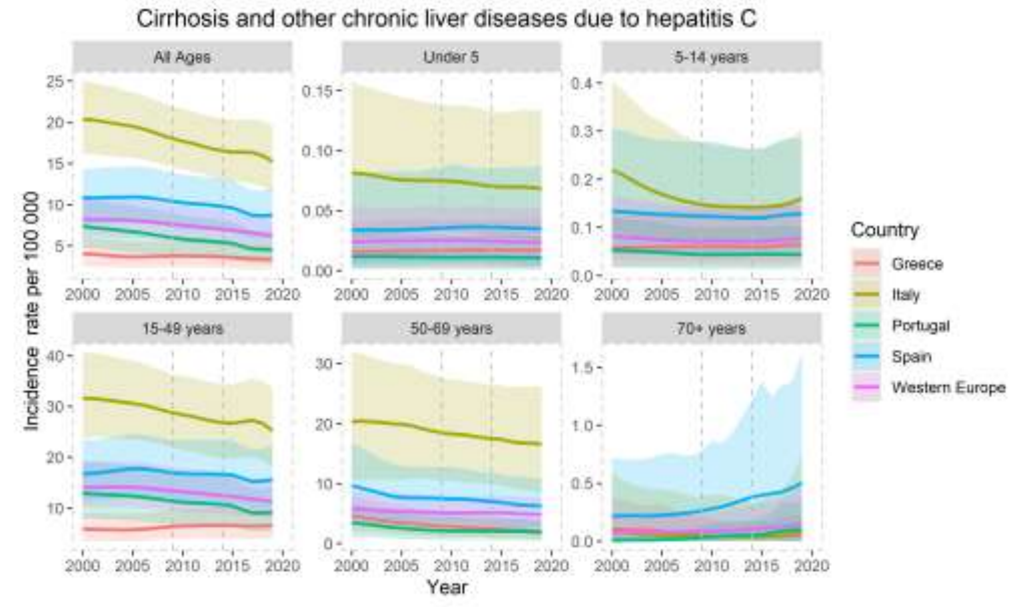
Cirrhosis and other chronic liver diseases due to hepatitis C
All Ages
Under 5
5-14 years
15-49 years
50-69 years
70+ years
Incidence rate per 100 000
Year
Country
Greece
Italy
Portugal
Spain
Western Europe

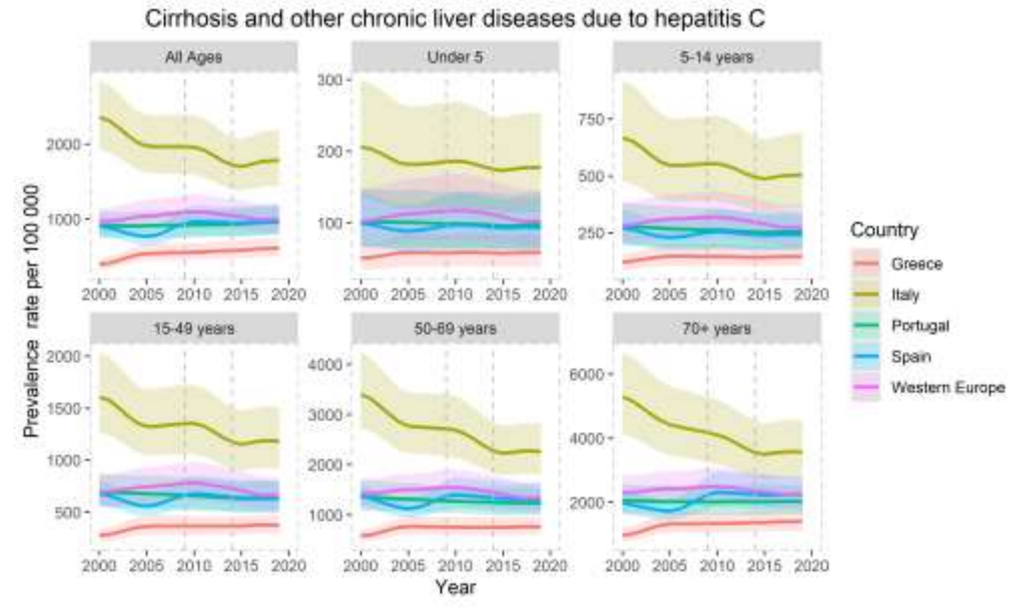
Cirrhosis and other chronic liver diseases due to hepatitis C
All Ages
Under 5
5-14 years
15-49 years
50-69 years
70+ years
Prevalence rate per 100 000
Year
Country
Greece
Italy
Portugal
Spain
Western Europe

E

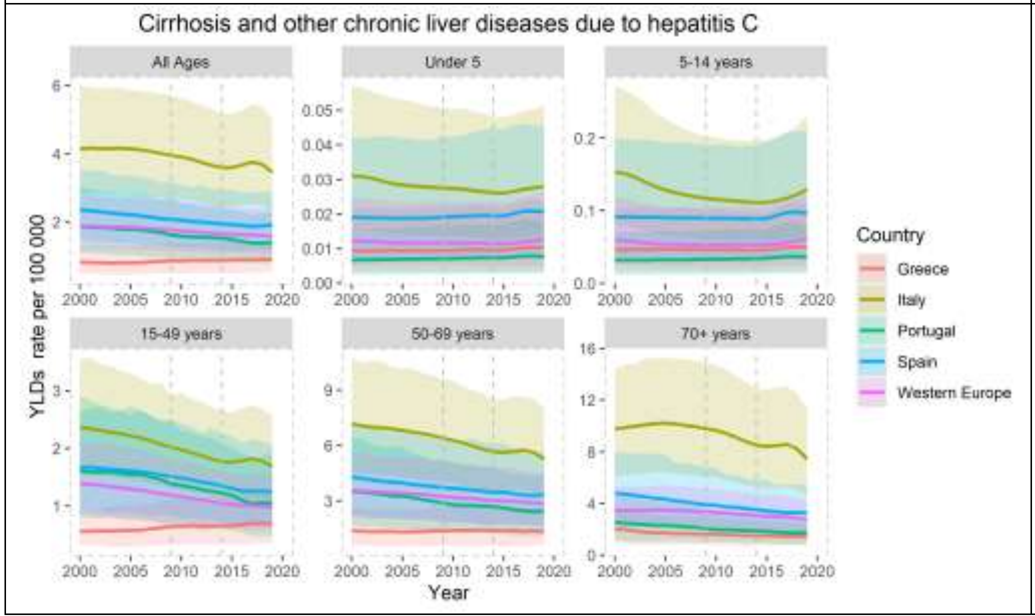
Cirrhosis and other chronic liver diseases due to hepatitis C
All Ages
Under 5
5-14 years
15-49 years
50-69 years
70+ years
YLDs rate per 100 000
Year
Country
Greece
Italy
Portugal
Spain
Western Europe

F

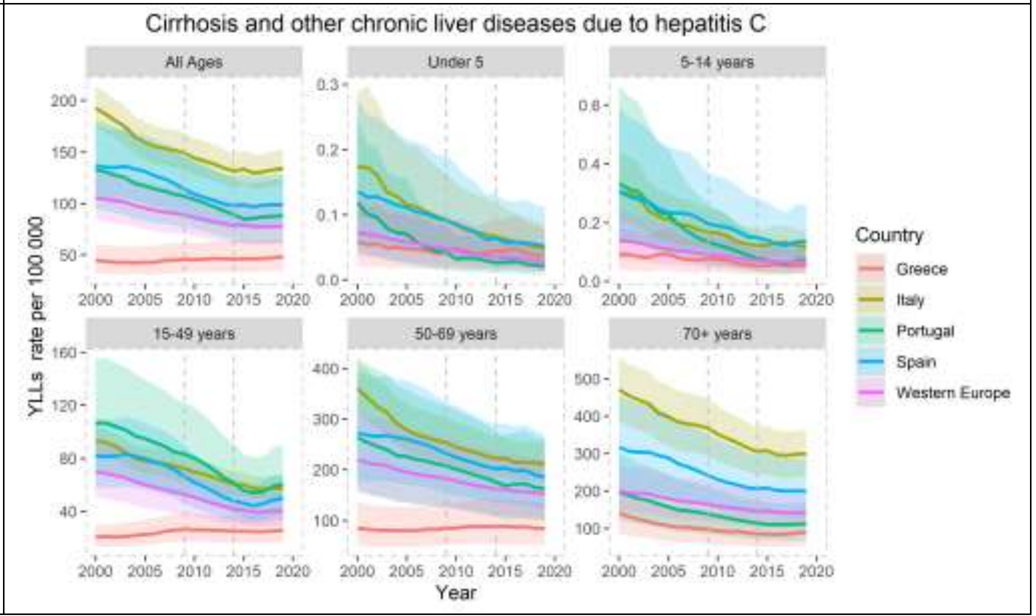
Cirrhosis and other chronic liver diseases due to hepatitis C
All Ages
Under 5
5-14 years
15-49 years
50-69 years
70+ years
YLLs rate per 100 000
Year
Country
Greece
Italy
Portugal
Spain
Western Europe

Figure S9 - Trends of **age-standardized rates** of **liver cancer due to hepatitis B** prevalence, incidence, mortality, years lived with disability (YLDs), years of life lost (YLLs) and disability-adjusted life years (DALYs) per 100,000 population by sex in Greece, Italy, Portugal, Spain, Western Europe from 2000 to 2019 (Global Burden of Disease Study 2019).

A

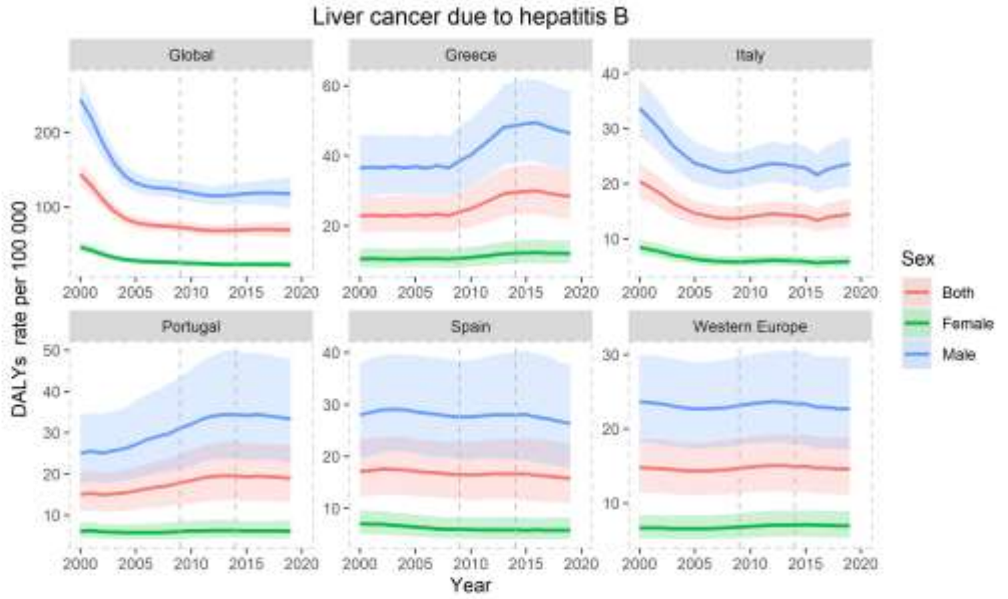

B

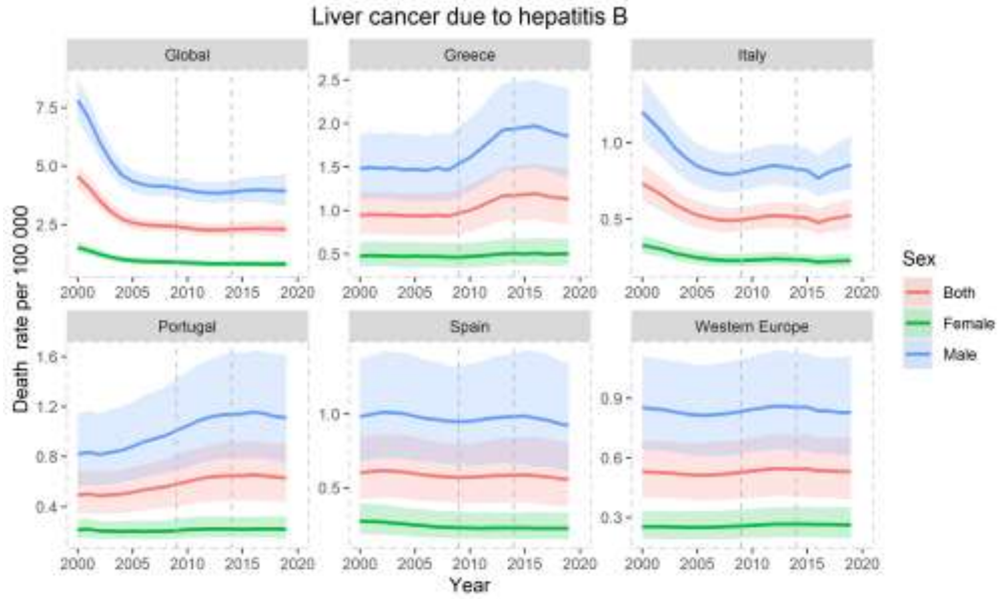

C

D

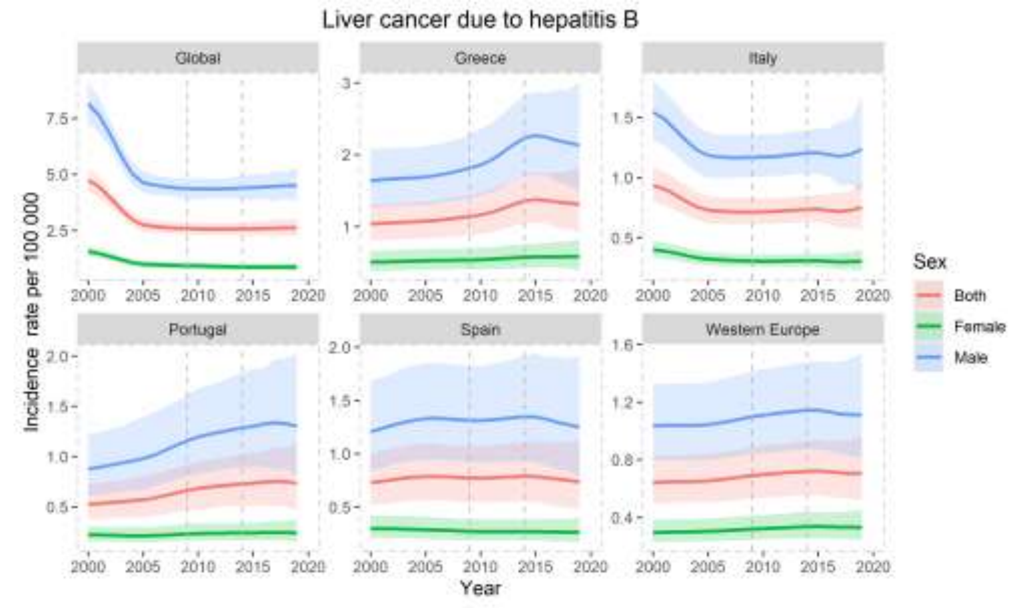
Liver cancer due to hepatitis B
Global
Greece
Italy
Portugal
Spain
Western Europe
Incidence rate per 100 000
Year
Sex
Both
Female
Male

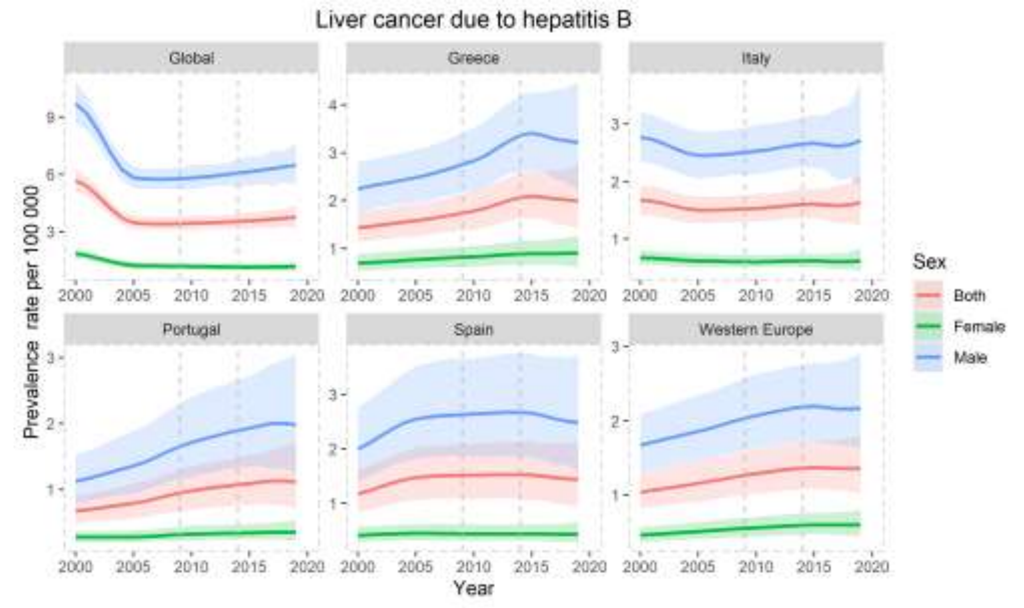
Liver cancer due to hepatitis B
Global
Greece
Italy
Portugal
Spain
Western Europe
Prevalence rate per 100 000
Year
Sex
Both
Female
Male

E

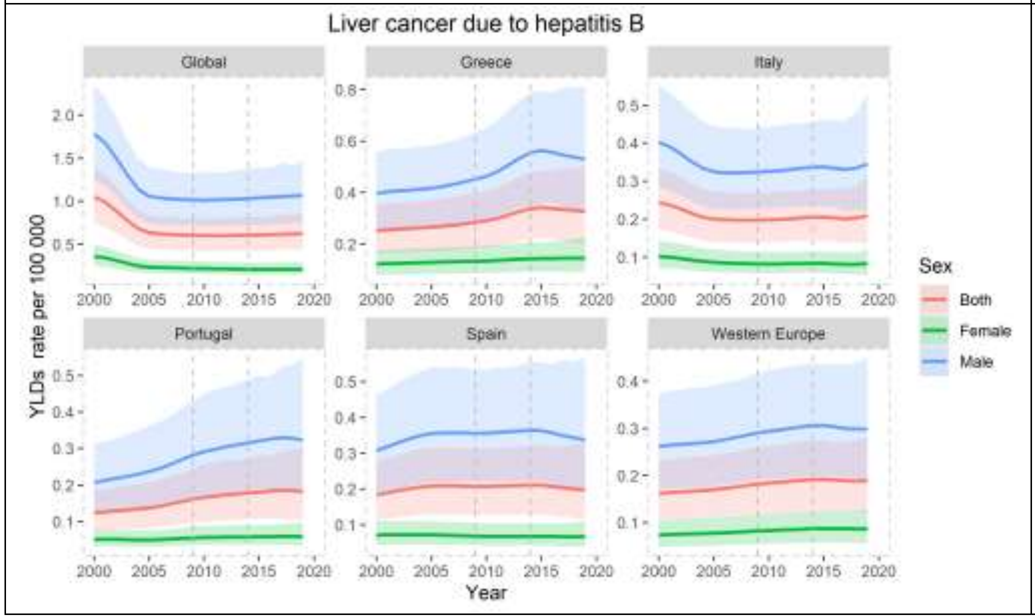
Liver cancer due to hepatitis B
Global
Greece
Italy
Portugal
Spain
Western Europe
YLDs rate per 100 000
Year
Sex
Both
Female
Male

F

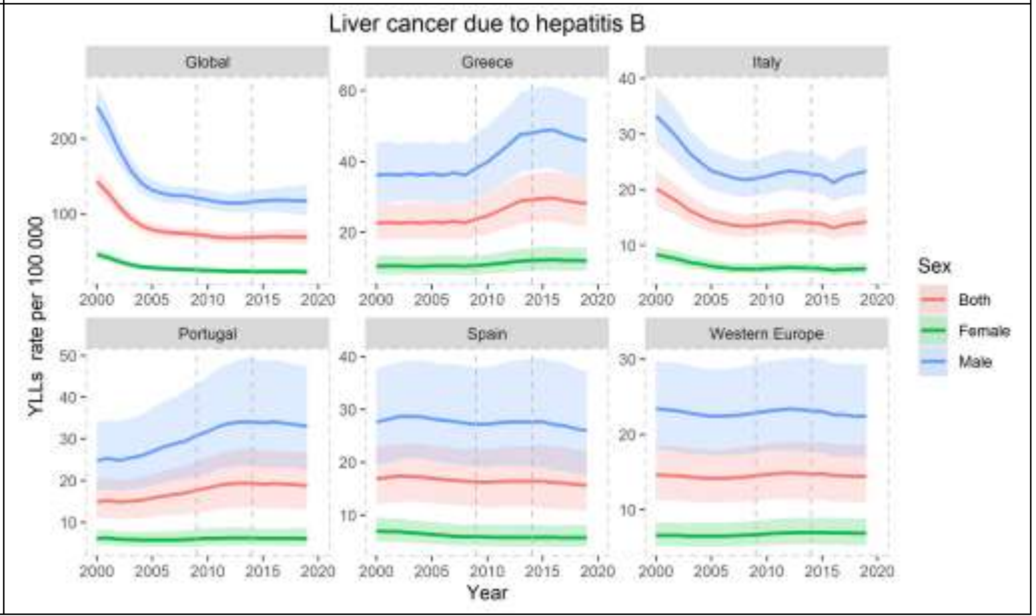
Liver cancer due to hepatitis B
Global
Greece
Italy
Portugal
Spain
Western Europe
YLLs rate per 100 000
Year
Sex
Both
Female
Male

Figure S10 - Trends of **rates** of **liver cancer due to hepatitis B** prevalence, incidence, mortality, years lived with disability (YLDs), years of life lost (YLLs) and disability-adjusted life years (DALYs) per 100,000 population by age group in Greece, Italy, Portugal, Spain and Western Europe from 2000 to 2019 (Global Burden of Disease Study 2019).

**A**	**B**
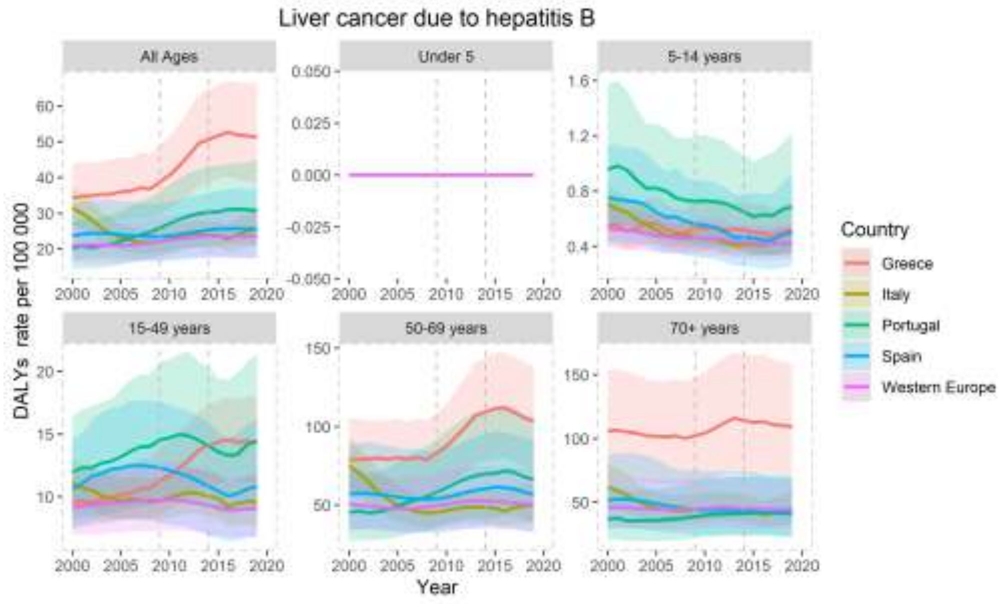	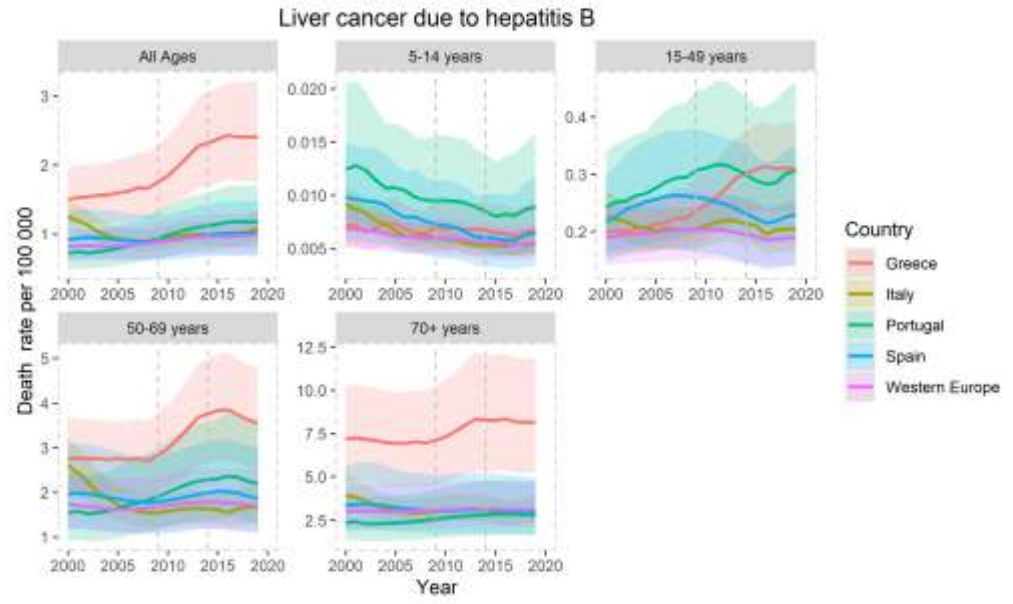
**C**	**D**

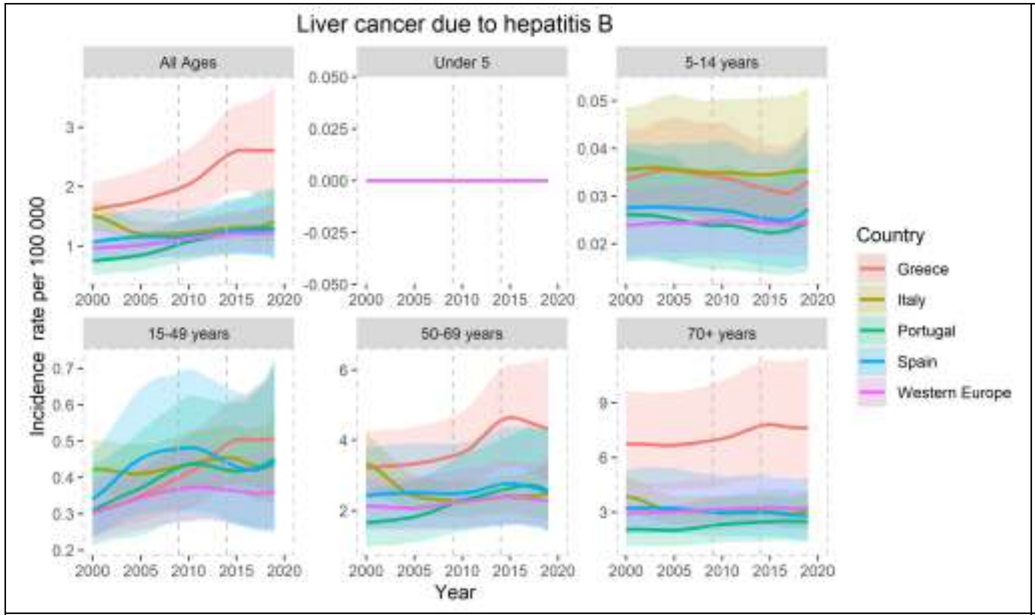
Liver cancer due to hepatitis B
All Ages
Under 5
5-14 years
15-49 years
50-69 years
70+ years
Incidence rate per 100 000
Year
Country
Greece
Italy
Portugal
Spain
Western Europe

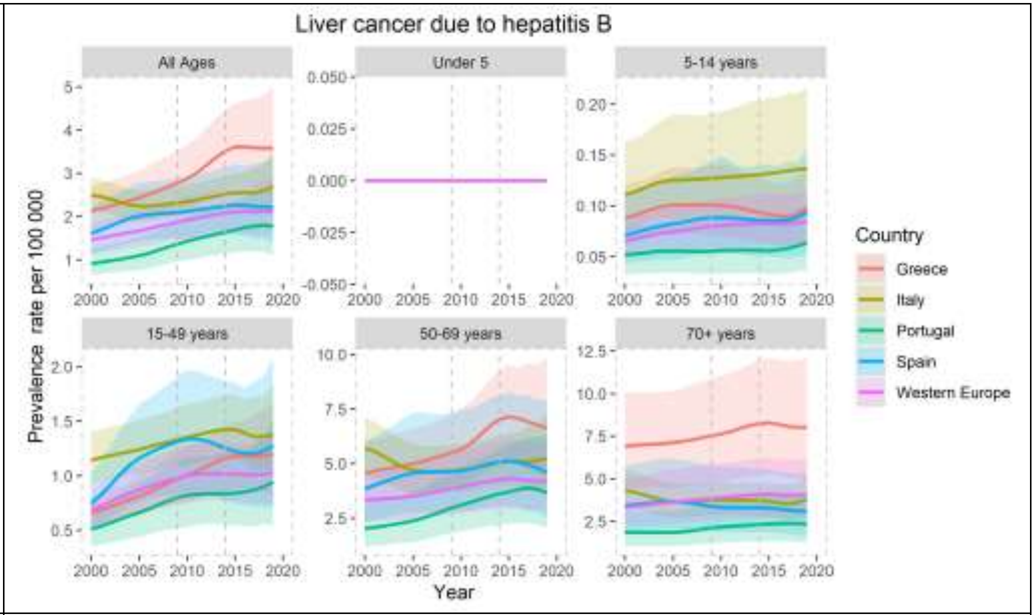
Liver cancer due to hepatitis B
All Ages
Under 5
5-14 years
15-49 years
50-69 years
70+ years
Prevalence rate per 100 000
Year
Country
Greece
Italy
Portugal
Spain
Western Europe

E

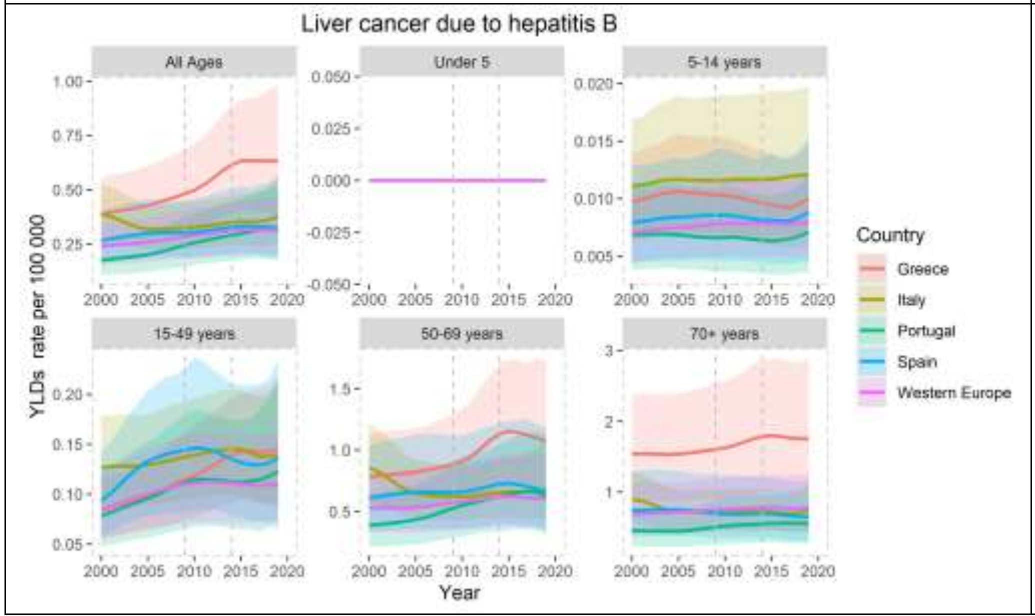
Liver cancer due to hepatitis B
All Ages
Under 5
5-14 years
15-49 years
50-69 years
70+ years
YLDs rate per 100 000
Year
Country
Greece
Italy
Portugal
Spain
Western Europe

F

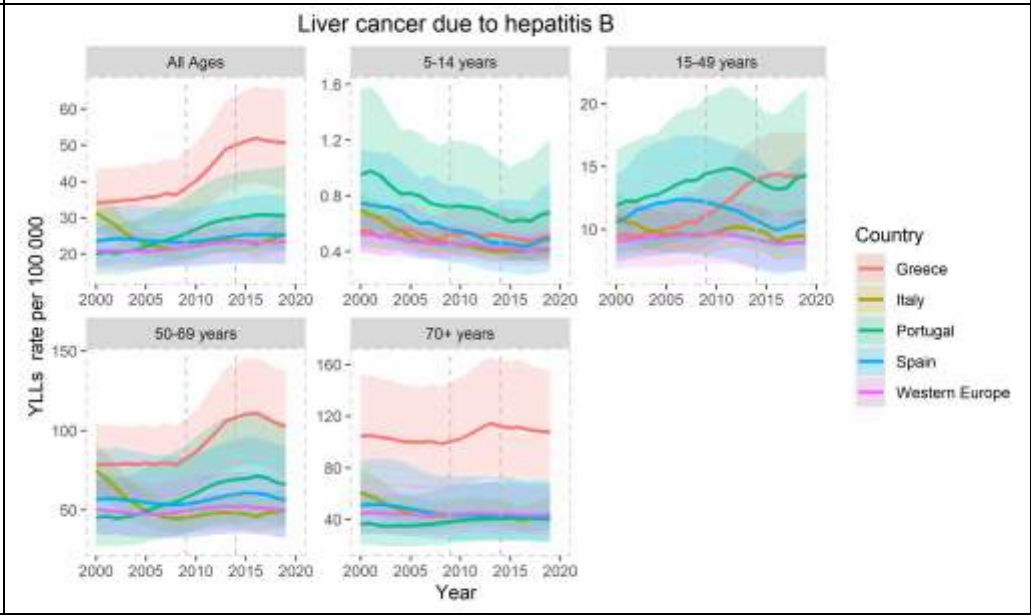
Liver cancer due to hepatitis B
All Ages
5-14 years
15-49 years
50-69 years
70+ years
YLLs rate per 100 000
Year
Country
Greece
Italy
Portugal
Spain
Western Europe

Figure S11 - Trends of **age-standardized rates** of **liver cancer due to hepatitis C** prevalence, incidence, mortality, years lived with disability (YLDs), years of life lost (YLLs) and disability-adjusted life years (DALYs) per 100,000 population by sex in Greece, Italy, Portugal, Spain, Western Europe from 2000 to 2019 (Global Burden of Disease Study 2019).

A

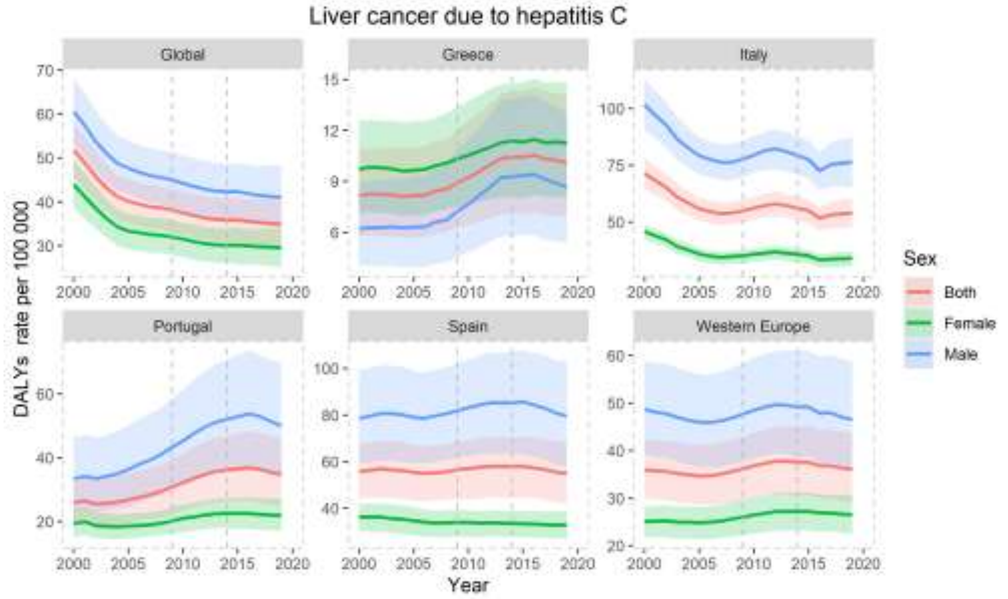

B

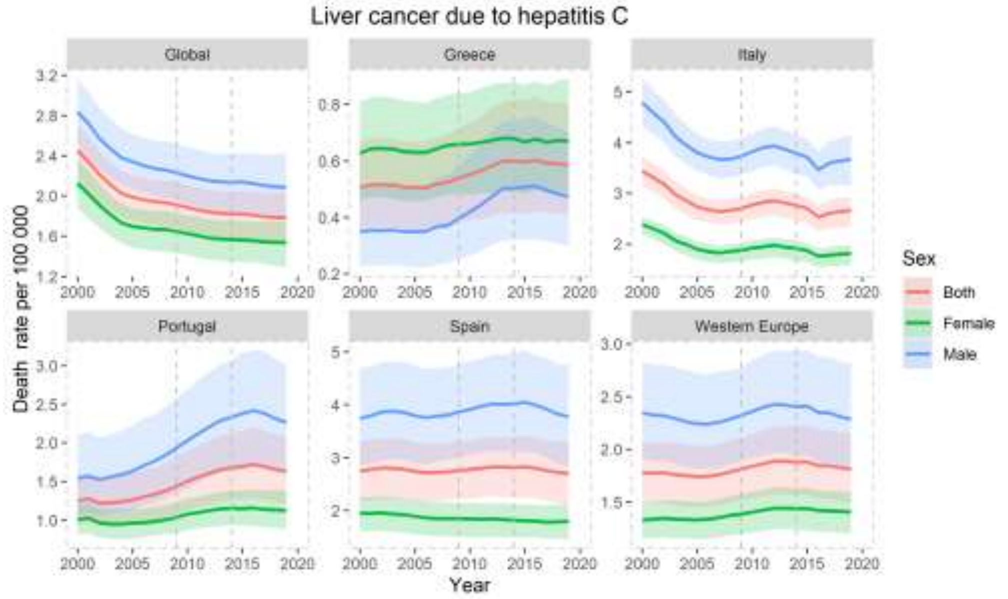

C

D

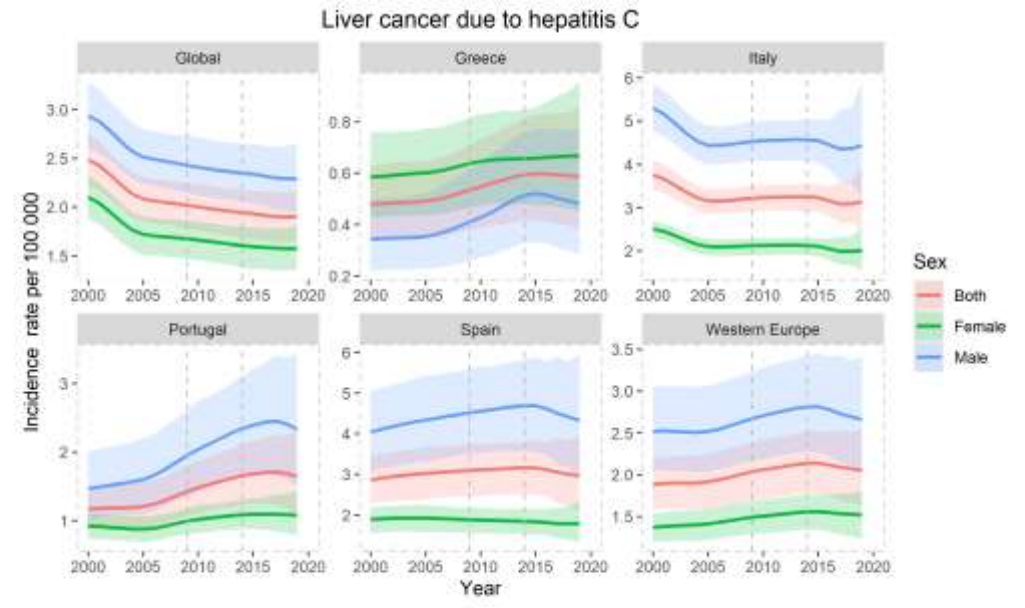
Liver cancer due to hepatitis C
Global
Greece
Italy
Portugal
Spain
Western Europe
Incidence rate per 100 000
Year
Sex
Both
Female
Male

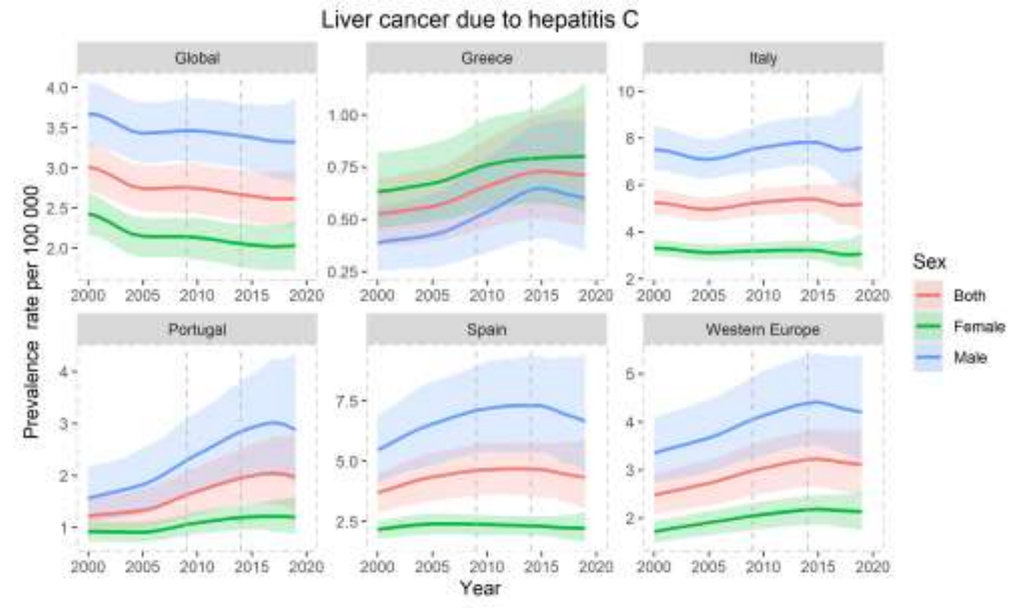
Liver cancer due to hepatitis C
Global
Greece
Italy
Portugal
Spain
Western Europe
Prevalence rate per 100 000
Year
Sex
Both
Female
Male

E

F

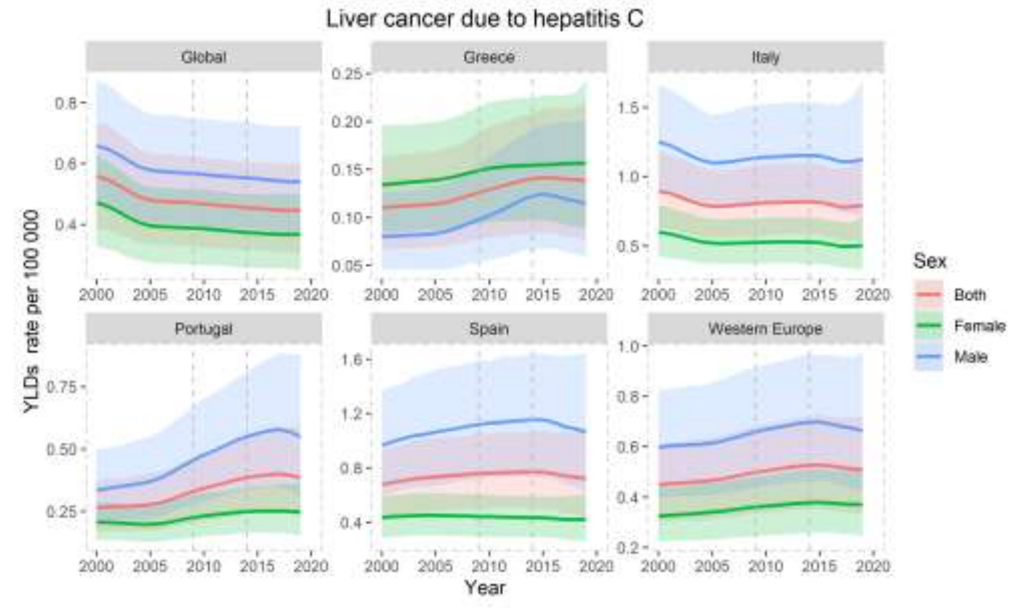
Liver cancer due to hepatitis C
Global
Greece
Italy
Portugal
Spain
Western Europe
YLDs rate per 100 000
Year
Sex
Both
Female
Male

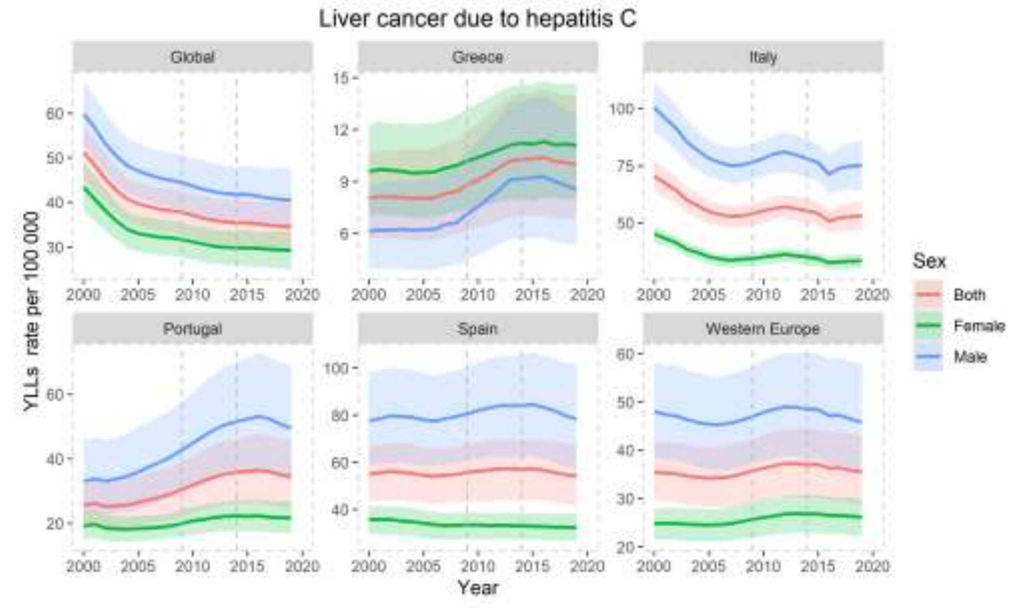
Liver cancer due to hepatitis C
Global
Greece
Italy
Portugal
Spain
Western Europe
YLLs rate per 100 000
Year
Sex
Both
Female
Male

Figure S12 - Trends of **rates** of **liver cancer due to hepatitis C** prevalence, incidence, mortality, years lived with disability (YLDs), years of life lost (YLLs) and disability-adjusted life years (DALYs) per 100,000 population by age group in Greece, Italy, Portugal, Spain and Western Europe from 2000 to 2019 (Global Burden of Disease Study 2019).

A

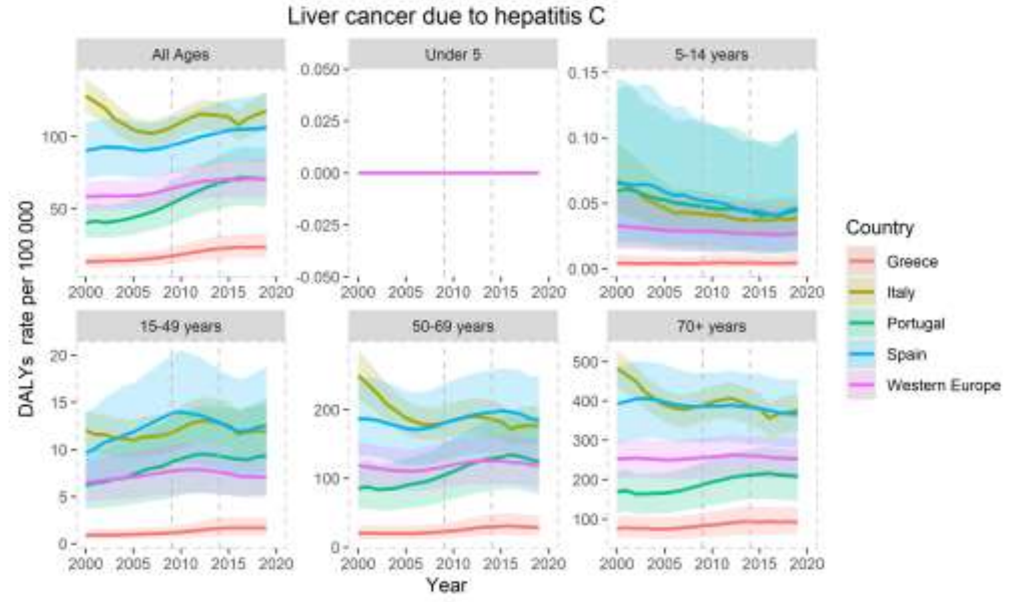

B

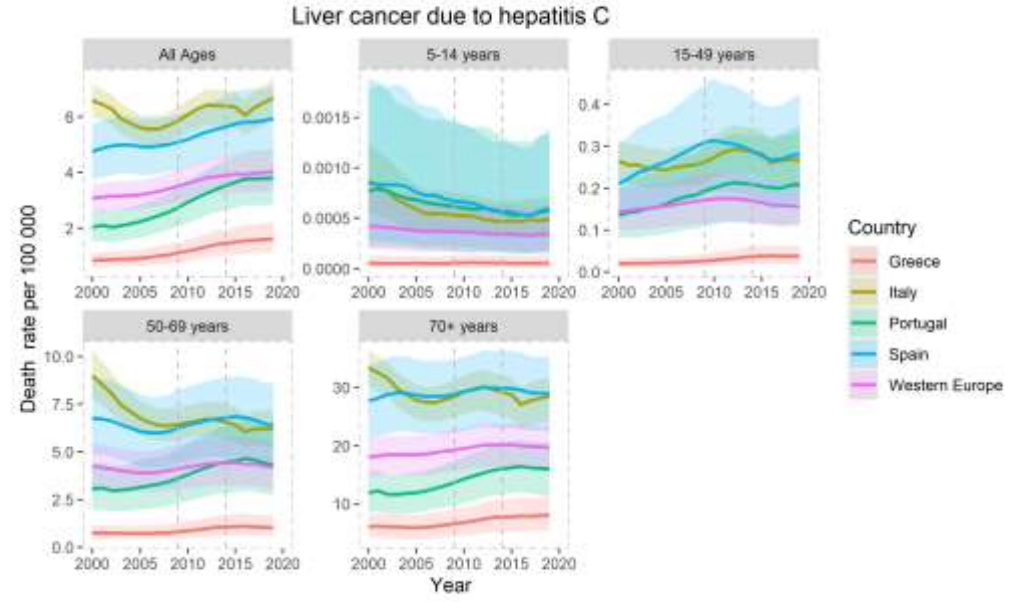

C

D

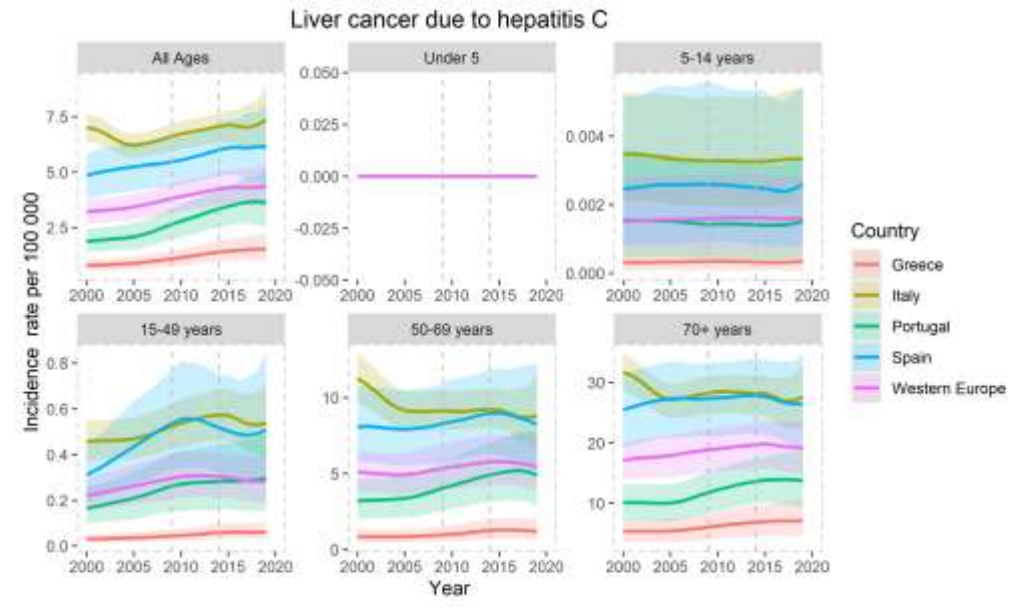
Liver cancer due to hepatitis C
All Ages
Under 5
5-14 years
15-49 years
50-69 years
70+ years
Incidence rate per 100 000
Year
Country
Greece
Italy
Portugal
Spain
Western Europe

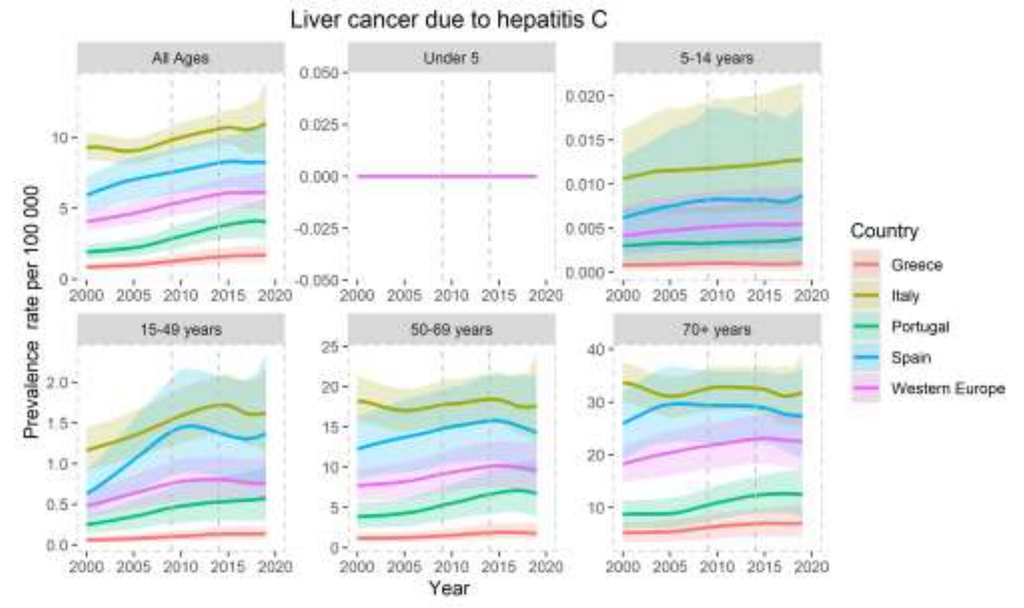
Liver cancer due to hepatitis C
All Ages
Under 5
5-14 years
15-49 years
50-69 years
70+ years
Prevalence rate per 100 000
Year
Country
Greece
Italy
Portugal
Spain
Western Europe

E

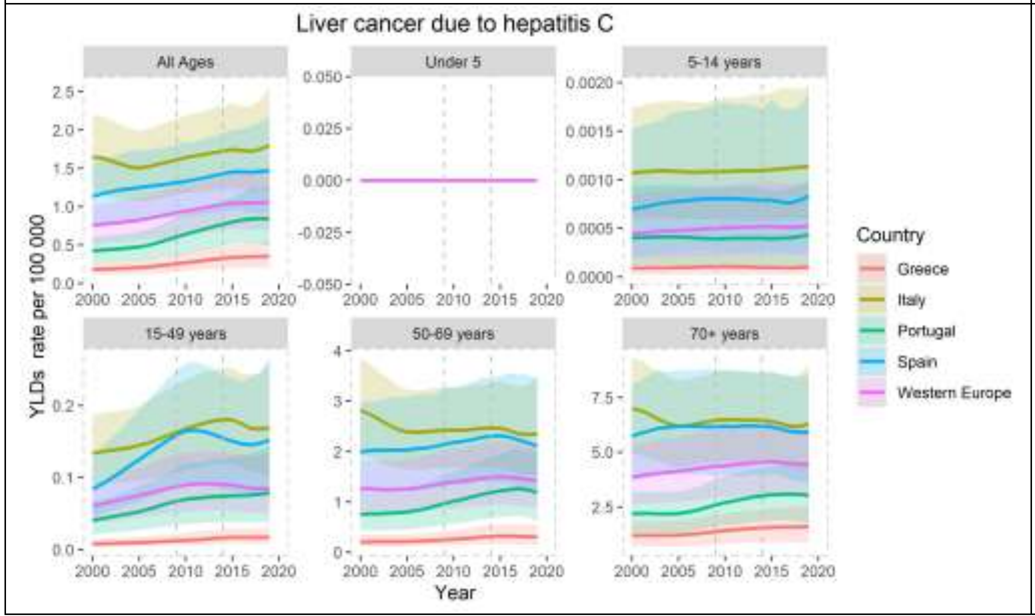
Liver cancer due to hepatitis C
All Ages
Under 5
5-14 years
15-49 years
50-69 years
70+ years
YLDs rate per 100 000
Year
Country
Greece
Italy
Portugal
Spain
Western Europe

F

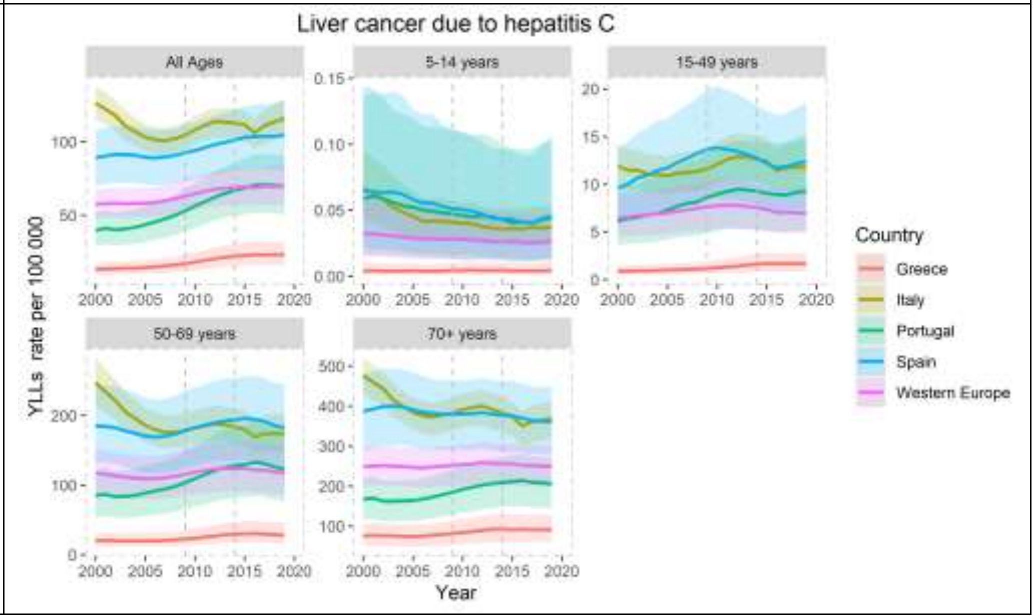
Liver cancer due to hepatitis C
All Ages
5-14 years
15-49 years
50-69 years
70+ years
YLLs rate per 100 000
Year
Country
Greece
Italy
Portugal
Spain
Western Europe

Section 4. Tables

Table S1- Age-standardized rates and 95% uncertainty levels (UL) of **acute HBV prevalence**, per 100,000 population in Greece, Italy, Portugal and Spain from 2000 to 2019 by sex classes (Global Burden of Disease Study 2019).

Year	Country	Sex	Prevalence (95% UL)	95% UL (upper)	95% UL (lower)
2000	Greece	Both	71.1	85.7	57.8
2000	Greece	Female	58.0	76.0	43.4
2000	Greece	Male	83.7	104.	65.5
2000	Italy	Both	25.7	32.7	19.6
2000	Italy	Female	20.3	27.1	14.7
2000	Italy	Male	31.2	40.7	23.7
2000	Portugal	Both	31.3	41.3	23.4
2000	Portugal	Female	22.1	30.5	14.6
2000	Portugal	Male	40.7	55.3	29.1
2000	Spain	Both	30.3	37.6	23.7
2000	Spain	Female	23.8	31.4	18.0
2000	Spain	Male	36.7	48.2	27.0
2001	Greece	Both	70.2	83.2	57.6
2001	Greece	Female	57.4	72.6	44.2
2001	Greece	Male	82.6	100.	66.3
2001	Italy	Both	24.8	31.3	19.0
2001	Italy	Female	19.7	25.9	14.5
2001	Italy	Male	30.0	38.7	23.1
2001	Portugal	Both	30.8	39.7	23.2
2001	Portugal	Female	21.6	29.3	14.6
2001	Portugal	Male	40.0	53.3	29.6
2001	Spain	Both	30.0	36.5	23.8
2001	Spain	Female	23.6	30.0	18.1
2001	Spain	Male	36.3	46.2	27.6
2002	Greece	Both	68.7	81.5	56.8
2002	Greece	Female	56.3	69.6	44.4
2002	Greece	Male	80.8	97.3	65.0
2002	Italy	Both	23.7	29.5	18.3
2002	Italy	Female	18.9	24.7	14.0
2002	Italy	Male	28.7	36.4	22.0
2002	Portugal	Both	30.2	38.4	22.8
2002	Portugal	Female	21.1	28.3	14.5
2002	Portugal	Male	39.4	51.9	29.5
2002	Spain	Both	29.3	35.7	23.3
2002	Spain	Female	23.1	28.7	17.8
2002	Spain	Male	35.5	44.7	27.7
2003	Greece	Both	67.1	80.1	55.1
2003	Greece	Female	55.1	67.7	44.2
2003	Greece	Male	78.8	96.0	62.9
2003	Italy	Both	22.6	28.2	17.4
2003	Italy	Female	18.1	23.4	13.4
2003	Italy	Male	27.2	34.6	20.9
2003	Portugal	Both	29.6	37.3	22.1
2003	Portugal	Female	20.5	27.6	14.2

2003	Portugal	Male	38.8	50.8	28.1
2003	Spain	Both	28.6	34.8	22.8
2003	Spain	Female	22.5	28.0	17.5
2003	Spain	Male	34.5	43.7	26.8
2004	Greece	Both	65.8	79.3	53.4
2004	Greece	Female	54.1	66.5	43.5
2004	Greece	Male	77.1	95.8	60.4
2004	Italy	Both	21.6	27.1	16.4
2004	Italy	Female	17.3	22.9	12.7
2004	Italy	Male	26.0	33.4	19.5
2004	Portugal	Both	29.0	36.9	21.3
2004	Portugal	Female	20.0	27.3	13.6
2004	Portugal	Male	38.2	50.7	27.0
2004	Spain	Both	27.9	34.7	22.0
2004	Spain	Female	22.0	27.9	16.8
2004	Spain	Male	33.7	43.6	25.3
2005	Greece	Both	65.1	79.8	52.0
2005	Greece	Female	53.5	67.3	42.0
2005	Greece	Male	76.3	96.6	57.3
2005	Italy	Both	20.9	26.6	15.6
2005	Italy	Female	16.9	22.8	12.1
2005	Italy	Male	25.1	32.9	18.2
2005	Portugal	Both	28.5	36.9	20.4
2005	Portugal	Female	19.6	27.4	12.9
2005	Portugal	Male	37.6	51.4	25.7
2005	Spain	Both	27.6	34.8	21.4
2005	Spain	Female	21.7	28.3	16.1
2005	Spain	Male	33.3	44.1	24.5
2006	Greece	Both	64.3	77.9	52.5
2006	Greece	Female	52.7	66.1	42.2
2006	Greece	Male	75.6	93.2	58.3
2006	Italy	Both	20.2	25.6	15.2
2006	Italy	Female	16.4	22.1	11.8
2006	Italy	Male	24.1	30.8	18.1
2006	Portugal	Both	28.0	36.0	20.2
2006	Portugal	Female	19.2	26.3	12.9
2006	Portugal	Male	36.9	49.6	25.6
2006	Spain	Both	27.2	33.8	21.5
2006	Spain	Female	21.3	27.4	16.2
2006	Spain	Male	32.7	42.7	25.0
2007	Greece	Both	63.0	75.2	52.0
2007	Greece	Female	51.3	64.0	41.2
2007	Greece	Male	74.3	90.8	58.5
2007	Italy	Both	19.1	24.2	14.4
2007	Italy	Female	15.5	20.8	11.2
2007	Italy	Male	22.7	28.5	17.1
2007	Portugal	Both	27.2	35.1	19.8
2007	Portugal	Female	18.7	25.4	12.7
2007	Portugal	Male	35.9	47.6	25.3
2007	Spain	Both	26.2	32.7	21.0
2007	Spain	Female	20.6	26.4	15.7
2007	Spain	Male	31.7	41.0	24.4
2008	Greece	Both	61.4	73.6	50.6

2008	Greece	Female	49.7	62.7	39.4
2008	Greece	Male	72.9	89.3	57.0
2008	Italy	Both	17.8	22.8	13.4
2008	Italy	Female	14.6	19.6	10.3
2008	Italy	Male	21.1	26.6	15.8
2008	Portugal	Both	26.3	34.4	19.1
2008	Portugal	Female	18.1	24.8	12.1
2008	Portugal	Male	34.7	46.3	24.1
2008	Spain	Both	25.1	31.4	20.0
2008	Spain	Female	19.7	25.4	14.9
2008	Spain	Male	30.4	38.8	23.1
2009	Greece	Both	60.1	73.7	48.4
2009	Greece	Female	48.3	61.7	37.0
2009	Greece	Male	71.6	89.9	55.2
2009	Italy	Both	16.7	21.5	12.3
2009	Italy	Female	13.8	18.9	9.6
2009	Italy	Male	19.7	25.5	14.6
2009	Portugal	Both	25.5	33.9	18.3
2009	Portugal	Female	17.5	24.6	11.6
2009	Portugal	Male	33.6	46.4	23.2
2009	Spain	Both	24.2	30.5	18.9
2009	Spain	Female	18.9	25.0	14.1
2009	Spain	Male	29.3	38.4	21.7
2010	Greece	Both	59.4	74.4	46.7
2010	Greece	Female	47.5	62.8	34.6
2010	Greece	Male	71.0	91.8	53.5
2010	Italy	Both	16.0	21.0	11.6
2010	Italy	Female	13.2	18.5	9.0
2010	Italy	Male	18.8	25.3	13.6
2010	Portugal	Both	24.8	34.0	17.2
2010	Portugal	Female	17.1	24.8	10.9
2010	Portugal	Male	32.9	46.7	22.1
2010	Spain	Both	23.7	30.6	18.1
2010	Spain	Female	18.6	25.1	13.5
2010	Spain	Male	28.8	38.7	20.8
2011	Greece	Both	58.7	72.5	47.0
2011	Greece	Female	46.9	60.4	35.7
2011	Greece	Male	70.3	88.0	54.0
2011	Italy	Both	15.4	19.9	11.1
2011	Italy	Female	12.8	17.6	8.81
2011	Italy	Male	18.1	23.6	13.2
2011	Portugal	Both	24.3	32.9	16.9
2011	Portugal	Female	16.6	23.8	10.8
2011	Portugal	Male	32.2	44.6	22.0
2011	Spain	Both	23.4	29.6	18.3
2011	Spain	Female	18.3	23.9	13.5
2011	Spain	Male	28.4	37.2	20.8
2012	Greece	Both	57.3	70.4	46.3
2012	Greece	Female	45.9	58.4	36.1
2012	Greece	Male	68.6	86.4	53.9
2012	Italy	Both	14.6	18.8	10.6
2012	Italy	Female	12.1	16.4	8.3
2012	Italy	Male	17.1	22.3	12.5

2012	Portugal	Both	23.6	31.7	16.4
2012	Portugal	Female	16.1	22.9	10.7
2012	Portugal	Male	31.5	42.8	21.5
2012	Spain	Both	22.8	28.9	17.8
2012	Spain	Female	17.8	23.1	13.5
2012	Spain	Male	27.8	35.8	20.7
2013	Greece	Both	55.7	68.8	44.8
2013	Greece	Female	44.7	56.7	35.4
2013	Greece	Male	66.6	84.2	52.0
2013	Italy	Both	13.7	17.7	9.9
2013	Italy	Female	11.3	15.3	7.8
2013	Italy	Male	16.1	21.1	11.7
2013	Portugal	Both	22.9	30.6	16.1
2013	Portugal	Female	15.4	21.9	10.2
2013	Portugal	Male	30.7	41.4	20.6
2013	Spain	Both	22.1	28.4	17.3
2013	Spain	Female	17.2	22.3	12.8
2013	Spain	Male	27.0	35.4	19.9
2014	Greece	Both	54.3	68.0	43.0
2014	Greece	Female	43.8	56.7	33.8
2014	Greece	Male	64.8	83.7	49.0
2014	Italy	Both	12.9	16.9	9.2
2014	Italy	Female	10.6	14.6	7.2
2014	Italy	Male	15.2	20.1	11.0
2014	Portugal	Both	22.2	30.2	15.3
2014	Portugal	Female	14.8	21.6	9.6
2014	Portugal	Male	29.8	41.0	19.6
2014	Spain	Both	21.5	28.0	16.6
2014	Spain	Female	16.7	22.3	12.0
2014	Spain	Male	26.3	35.5	19.3
2015	Greece	Both	53.5	68.3	41.0
2015	Greece	Female	43.2	58.1	31.8
2015	Greece	Male	63.8	86.2	46.1
2015	Italy	Both	12.4	16.6	8.8
2015	Italy	Female	10.2	14.3	6.8
2015	Italy	Male	14.7	19.9	10.5
2015	Portugal	Both	21.5	30.2	14.5
2015	Portugal	Female	14.3	20.8	8.9
2015	Portugal	Male	29.0	41.5	18.6
2015	Spain	Both	21.2	27.8	15.9
2015	Spain	Female	16.4	22.3	11.2
2015	Spain	Male	25.9	36.1	18.7
2016	Greece	Both	52.7	64.8	41.0
2016	Greece	Female	42.8	54.1	32.8
2016	Greece	Male	62.7	80.2	47.8
2016	Italy	Both	11.4	14.9	8.0
2016	Italy	Female	9.42	12.7	6.3
2016	Italy	Male	13.4	18.0	9.5
2016	Portugal	Both	20.6	27.8	14.1
2016	Portugal	Female	13.9	19.5	9.4
2016	Portugal	Male	27.6	38.1	18.1
2016	Spain	Both	19.6	25.1	14.9
2016	Spain	Female	15.3	20.0	11.3

2016	Spain	Male	23.8	31.6	17.6
2017	Greece	Both	52.1	65.2	39.7
2017	Greece	Female	42.5	56.6	31.2
2017	Greece	Male	61.8	83.2	44.5
2017	Italy	Both	10.3	13.9	7.0
2017	Italy	Female	8.5	12.0	5.5
2017	Italy	Male	12.1	17.0	8.4
2017	Portugal	Both	19.7	27.0	12.9
2017	Portugal	Female	13.6	19.3	8.8
2017	Portugal	Male	26.0	38.4	16.4
2017	Spain	Both	17.8	23.3	13.1
2017	Spain	Female	14.1	18.9	9.9
2017	Spain	Male	21.5	29.7	14.7
2018	Greece	Both	51.8	62.2	41.5
2018	Greece	Female	42.2	52.2	33.3
2018	Greece	Male	61.6	77.6	47.2
2018	Italy	Both	10.0	13.4	6.9
2018	Italy	Female	8.3	11.4	5.5
2018	Italy	Male	11.9	16.1	8.3
2018	Portugal	Both	18.9	25.5	13.1
2018	Portugal	Female	13.0	17.9	8.4
2018	Portugal	Male	25.1	35.3	16.8
2018	Spain	Both	17.5	22.1	13.4
2018	Spain	Female	13.8	17.8	10.2
2018	Spain	Male	21.2	27.9	15.7
2019	Greece	Both	51.7	64.0	40.8
2019	Greece	Female	42.0	54.1	31.7
2019	Greece	Male	61.5	80.7	44.2
2019	Italy	Both	9.9	13.4	6.8
2019	Italy	Female	8.1	11.5	5.3
2019	Italy	Male	11.7	16.4	8.0
2019	Portugal	Both	17.9	24.9	12.0
2019	Portugal	Female	12.0	18.0	6.9
2019	Portugal	Male	24.2	34.8	15.8
2019	Spain	Both	17.5	22.4	13.1
2019	Spain	Female	13.6	18.4	9.5
2019	Spain	Male	21.4	29.1	15.0

Table S2- Age-standardized rates and 95% uncertainty levels (UL) of **acute HBV incidence**, per 100,000 population in Greece, Italy, Portugal and Spain from 2000 to 2019 by sex classes (Global Burden of Disease Study 2019).

Year	Country	Sex	Incidence (95% UL)	95% UL (upper)	95% UL (lower)
2000	Greece	Both	616.2	743.1	501.3
2000	Greece	Female	503.3	659.1	376.7
2000	Greece	Male	725.8	902.2	568.4
2000	Italy	Both	223.0	283.5	170.4
2000	Italy	Female	176.4	234.9	127.9
2000	Italy	Male	270.6	353.0	205.5
2000	Portugal	Both	271.9	358.5	203.0
2000	Portugal	Female	191.9	264.8	126.9
2000	Portugal	Male	352.8	479.6	252.6
2000	Spain	Both	263.2	326.3	206.2
2000	Spain	Female	206.9	272.7	156.3
2000	Spain	Male	318.5	418.5	234.1
2001	Greece	Both	608.6	721.4	499.8
2001	Greece	Female	497.7	629.4	383.9
2001	Greece	Male	716.2	872.2	575.1
2001	Italy	Both	215.5	272.0	165.0
2001	Italy	Female	171.2	224.9	125.7
2001	Italy	Male	260.7	335.9	200.7
2001	Portugal	Both	267.0	344.1	201.2
2001	Portugal	Female	187.6	254.4	127.1
2001	Portugal	Male	347.4	462.1	257.0
2001	Spain	Both	260.3	317.1	207.0
2001	Spain	Female	204.6	260.1	157.1
2001	Spain	Male	314.9	400.6	239.7
2002	Greece	Both	596.1	706.4	492.5
2002	Greece	Female	488.4	604.0	384.9
2002	Greece	Male	700.5	843.6	563.5
2002	Italy	Both	206.1	256.3	158.9
2002	Italy	Female	164.3	214.7	121.7
2002	Italy	Male	248.8	315.8	191.1
2002	Portugal	Both	261.9	332.8	198.4
2002	Portugal	Female	182.9	245.6	125.8
2002	Portugal	Male	341.9	449.9	256.2
2002	Spain	Both	254.7	309.5	202.2
2002	Spain	Female	200.2	248.9	154.9
2002	Spain	Male	307.7	387.4	240.5
2003	Greece	Both	582.2	694.6	477.7
2003	Greece	Female	478.0	587.3	383.1
2003	Greece	Male	683.2	832.4	545.1
2003	Italy	Both	196.4	244.7	151.4
2003	Italy	Female	157.1	203.6	116.7
2003	Italy	Male	236.5	300.5	181.1
2003	Portugal	Both	256.7	323.9	191.6
2003	Portugal	Female	178.2	239.2	123.3
2003	Portugal	Male	336.4	440.6	244.2
2003	Spain	Both	248.1	302.1	197.9

2003	Spain	Female	195.1	242.7	151.8
2003	Spain	Male	299.4	379.2	232.7
2004	Greece	Both	570.4	687.6	462.8
2004	Greece	Female	469.0	577.1	377.3
2004	Greece	Male	668.7	830.7	523.9
2004	Italy	Both	187.8	235.7	142.9
2004	Italy	Female	150.7	198.5	110.5
2004	Italy	Male	225.6	290.3	169.3
2004	Portugal	Both	251.9	319.9	184.7
2004	Portugal	Female	173.9	237.1	118.6
2004	Portugal	Male	331.3	439.7	234.2
2004	Spain	Both	242.5	300.9	191.3
2004	Spain	Female	190.7	242.5	145.8
2004	Spain	Male	292.2	378.3	220.1
2005	Greece	Both	564.2	692.0	450.8
2005	Greece	Female	463.8	583.4	364.2
2005	Greece	Male	661.5	838.0	496.6
2005	Italy	Both	181.9	231.2	136.0
2005	Italy	Female	146.7	197.6	105.0
2005	Italy	Male	217.8	285.2	158.5
2005	Portugal	Both	247.7	320.1	177.5
2005	Portugal	Female	170.5	238.2	112.5
2005	Portugal	Male	326.6	445.8	222.8
2005	Spain	Both	239.6	302.2	185.8
2005	Spain	Female	188.4	246.0	140.3
2005	Spain	Male	288.6	383.0	212.6
2006	Greece	Both	558.0	675.1	455.6
2006	Greece	Female	457.3	573.0	366.0
2006	Greece	Male	655.6	808.4	505.7
2006	Italy	Both	175.4	221.9	132.4
2006	Italy	Female	142.2	191.6	102.8
2006	Italy	Male	209.2	267.7	156.9
2006	Portugal	Both	242.7	312.7	175.4
2006	Portugal	Female	166.9	228.4	112.1
2006	Portugal	Male	320.3	430.0	222.1
2006	Spain	Both	235.8	293.7	186.4
2006	Spain	Female	185.1	237.5	140.4
2006	Spain	Male	284.1	370.1	216.9
2007	Greece	Both	546.3	652.0	451.2
2007	Greece	Female	445.1	555.1	357.8
2007	Greece	Male	644.6	787.6	507.6
2007	Italy	Both	165.6	210.0	125.2
2007	Italy	Female	135.0	180.5	97.50
2007	Italy	Male	196.7	247.5	148.9
2007	Portugal	Both	235.8	304.3	172.3
2007	Portugal	Female	162.2	220.3	110.8
2007	Portugal	Male	311.3	412.7	219.7
2007	Spain	Both	227.7	283.7	182.0
2007	Spain	Female	178.6	229.2	136.8
2007	Spain	Male	274.8	355.4	211.5
2008	Greece	Both	532.9	638.6	438.5
2008	Greece	Female	430.9	543.5	341.4
2008	Greece	Male	632.0	774.7	494.1

2008	Italy	Both	154.7	197.9	116.4
2008	Italy	Female	126.8	170.0	89.55
2008	Italy	Male	183.0	231.2	137.5
2008	Portugal	Both	228.1	298.3	165.6
2008	Portugal	Female	157.0	215.0	105.2
2008	Portugal	Male	301.3	401.2	209.3
2008	Spain	Both	218.3	272.7	173.3
2008	Spain	Female	170.9	220.3	129.4
2008	Spain	Male	263.9	337.0	200.7
2009	Greece	Both	521.4	639.4	419.6
2009	Greece	Female	418.6	535.0	320.9
2009	Greece	Male	621.3	779.4	478.8
2009	Italy	Both	145.0	186.7	107.2
2009	Italy	Female	119.6	164.5	83.32
2009	Italy	Male	171.0	221.5	126.6
2009	Portugal	Both	221.0	294.1	158.6
2009	Portugal	Female	152.2	213.4	101.3
2009	Portugal	Male	292.0	402.6	201.1
2009	Spain	Both	210.2	264.7	163.8
2009	Spain	Female	164.4	216.9	122.3
2009	Spain	Male	254.5	333.4	188.9
2010	Greece	Both	515.4	644.9	405.3
2010	Greece	Female	412.0	544.8	300.0
2010	Greece	Male	616.1	796.3	463.9
2010	Italy	Both	139.0	182.4	101.2
2010	Italy	Female	115.2	160.6	78.62
2010	Italy	Male	163.3	219.3	118.2
2010	Portugal	Both	215.6	295.0	149.8
2010	Portugal	Female	148.4	215.2	94.77
2010	Portugal	Male	285.2	404.8	191.9
2010	Spain	Both	206.1	265.7	157.6
2010	Spain	Female	161.2	218.1	117.1
2010	Spain	Male	249.8	335.9	180.4
2011	Greece	Both	509.2	629.0	407.8
2011	Greece	Female	406.8	524.1	309.4
2011	Greece	Male	609.4	763.3	468.3
2011	Italy	Both	133.8	172.8	96.69
2011	Italy	Female	111.1	152.5	76.36
2011	Italy	Male	157.1	204.6	114.8
2011	Portugal	Both	210.8	285.3	146.9
2011	Portugal	Female	144.5	206.5	94.28
2011	Portugal	Male	279.7	386.6	191.2
2011	Spain	Both	203.4	257.3	158.6
2011	Spain	Female	158.9	207.1	117.7
2011	Spain	Male	246.7	322.5	180.7
2012	Greece	Both	497.3	610.5	401.4
2012	Greece	Female	398.1	506.6	313.0
2012	Greece	Male	594.9	748.9	467.4
2012	Italy	Both	126.6	163.1	92.07
2012	Italy	Female	105.0	142.5	72.53
2012	Italy	Male	148.8	193.2	108.9
2012	Portugal	Both	205.1	275.5	142.9
2012	Portugal	Female	139.6	198.8	93.38

2012	Portugal	Male	273.2	371.1	186.8
2012	Spain	Both	198.3	251.1	155.0
2012	Spain	Female	154.5	200.2	117.0
2012	Spain	Male	241.0	310.4	179.5
2013	Greece	Both	483.3	596.4	388.8
2013	Greece	Female	388.2	491.8	307.2
2013	Greece	Male	577.4	730.4	450.8
2013	Italy	Both	118.9	153.9	86.43
2013	Italy	Female	98.3	133.0	67.8
2013	Italy	Male	140.1	183.0	92.5
2013	Portugal	Both	198.8	265.9	89.1
2013	Portugal	Female	134.2	190.1	98.8
2013	Portugal	Male	266.2	359.4	81.6
2013	Spain	Both	192.3	246.4	89.6
2013	Spain	Female	149.4	193.2	74.2
2013	Spain	Male	234.4	307.3	87.5
2014	Greece	Both	471.0	590.1	72.3
2014	Greece	Female	379.7	491.9	86.2
2014	Greece	Male	561.9	725.4	425.1
2014	Italy	Both	112.2	147.2	80.40
2014	Italy	Female	92.5	127.0	62.8
2014	Italy	Male	132.5	174.2	95.81
2014	Portugal	Both	192.6	261.8	133.1
2014	Portugal	Female	129.0	187.3	83.92
2014	Portugal	Male	259.0	355.7	170.3
2014	Spain	Both	187.0	243.0	144.1
2014	Spain	Female	144.9	193.8	104.1
2014	Spain	Male	228.5	308.1	167.4
2015	Greece	Both	463.9	592.3	356.1
2015	Greece	Female	375.0	503.6	276.1
2015	Greece	Male	553.0	747.7	399.5
2015	Italy	Both	108.1	144.6	77.02
2015	Italy	Female	89.1	123.9	59.3
2015	Italy	Male	127.9	173.3	91.03
2015	Portugal	Both	186.9	262.0	126.1
2015	Portugal	Female	124.6	180.8	77.87
2015	Portugal	Male	252.1	360.1	161.2
2015	Spain	Both	183.9	241.7	138.5
2015	Spain	Female	142.3	194.1	97.88
2015	Spain	Male	224.9	313.1	162.6
2016	Greece	Both	457.5	562.0	355.9
2016	Greece	Female	371.7	469.7	284.4
2016	Greece	Male	544.0	695.6	414.3
2016	Italy	Both	98.8	129.6	69.9
2016	Italy	Female	81.6	110.8	54.7
2016	Italy	Male	116.7	156.3	82.83
2016	Portugal	Both	178.9	241.6	122.8
2016	Portugal	Female	121.3	169.5	81.57
2016	Portugal	Male	239.2	330.8	156.8
2016	Spain	Both	170.0	218.2	129.8
2016	Spain	Female	133.1	173.5	98.55
2016	Spain	Male	206.5	274.2	153.0
2017	Greece	Both	451.6	565.5	344.5

2017	Greece	Female	368.3	491.1	271.2
2017	Greece	Male	535.9	721.4	385.8
2017	Italy	Both	89.6	120.7	61.2
2017	Italy	Female	74.2	104.6	47.6
2017	Italy	Male	105.5	147.4	73.00
2017	Portugal	Both	170.8	234.3	112.3
2017	Portugal	Female	118.3	167.7	76.50
2017	Portugal	Male	225.9	333.1	142.2
2017	Spain	Both	155.0	202.2	113.7
2017	Spain	Female	122.8	163.9	86.01
2017	Spain	Male	186.8	258.0	128.1
2018	Greece	Both	449.7	539.0	360.1
2018	Greece	Female	366.4	452.6	289.4
2018	Greece	Male	533.9	672.7	409.5
2018	Italy	Both	87.5	116.9	60.2
2018	Italy	Female	72.3	98.9	48.0
2018	Italy	Male	103.2	139.6	72.19
2018	Portugal	Both	164.0	221.6	114.0
2018	Portugal	Female	112.8	155.7	73.65
2018	Portugal	Male	217.9	306.1	145.9
2018	Spain	Both	151.9	192.0	116.8
2018	Spain	Female	119.8	154.4	88.40
2018	Spain	Male	183.9	242.5	136.0
2019	Greece	Both	448.9	555.2	354.3
2019	Greece	Female	364.7	468.9	275.2
2019	Greece	Male	533.8	699.7	383.3
2019	Italy	Both	86.2	116.8	59.0
2019	Italy	Female	70.99	99.95	46.77
2019	Italy	Male	102.0	142.9	69.43
2019	Portugal	Both	155.6	216.3	104.3
2019	Portugal	Female	104.1	156.0	60.34
2019	Portugal	Male	210.2	301.6	137.1
2019	Spain	Both	151.7	194.8	114.1
2019	Spain	Female	118.0	160.1	82.44
2019	Spain	Male	185.4	252.3	130.0

Table S3- Age-standardized rates and 95% uncertainty levels (UL) of **acute HBV deaths** per 100,000 population in Greece, Italy, Portugal and Spain from 2000 to 2019 by sex classes (Global Burden of Disease Study 2019).

Year	Country	Sex	Deaths (95% UL)	95% UL (upper)	95% UL (lower)
2000	Greece	Both	0.10	0.11	0.08
2000	Greece	Female	0.05	0.07	0.03
2000	Greece	Male	0.15	0.17	0.12
2000	Italy	Both	0.03	0.04	0.02
2000	Italy	Female	0.02	0.03	0.01
2000	Italy	Male	0.05	0.06	0.03
2000	Portugal	Both	0.10	0.12	0.07
2000	Portugal	Female	0.04	0.05	0.03
2000	Portugal	Male	0.16	0.19	0.10
2000	Spain	Both	0.01	0.01	0.00
2000	Spain	Female	0.00	0.01	0.00
2000	Spain	Male	0.01	0.02	0.01
2001	Greece	Both	0.10	0.11	0.08
2001	Greece	Female	0.05	0.06	0.03
2001	Greece	Male	0.15	0.17	0.12
2001	Italy	Both	0.03	0.04	0.02
2001	Italy	Female	0.02	0.02	0.01
2001	Italy	Male	0.05	0.06	0.03
2001	Portugal	Both	0.05	0.05	0.04
2001	Portugal	Female	0.02	0.03	0.02
2001	Portugal	Male	0.07	0.09	0.05
2001	Spain	Both	0.01	0.01	0.00
2001	Spain	Female	0.00	0.01	0.00
2001	Spain	Male	0.01	0.02	0.01
2002	Greece	Both	0.10	0.12	0.08
2002	Greece	Female	0.06	0.07	0.03
2002	Greece	Male	0.16	0.18	0.13
2002	Italy	Both	0.03	0.04	0.02
2002	Italy	Female	0.02	0.02	0.01
2002	Italy	Male	0.05	0.06	0.03
2002	Portugal	Both	0.03	0.04	0.03
2002	Portugal	Female	0.02	0.02	0.01
2002	Portugal	Male	0.05	0.07	0.04
2002	Spain	Both	0.01	0.01	0.00
2002	Spain	Female	0.00	0.01	0.00
2002	Spain	Male	0.01	0.02	0.01
2003	Greece	Both	0.12	0.13	0.09
2003	Greece	Female	0.06	0.07	0.03
2003	Greece	Male	0.18	0.20	0.15
2003	Italy	Both	0.04	0.05	0.02
2003	Italy	Female	0.02	0.03	0.01
2003	Italy	Male	0.05	0.08	0.03
2003	Portugal	Both	0.03	0.04	0.02
2003	Portugal	Female	0.02	0.02	0.01
2003	Portugal	Male	0.04	0.06	0.04
2003	Spain	Both	0.01	0.01	0.00
2003	Spain	Female	0.00	0.01	0.00

2003	Spain	Male	0.01	0.02	0.01
2004	Greece	Both	0.14	0.16	0.11
2004	Greece	Female	0.06	0.08	0.04
2004	Greece	Male	0.22	0.25	0.18
2004	Italy	Both	0.03	0.05	0.02
2004	Italy	Female	0.02	0.03	0.01
2004	Italy	Male	0.05	0.08	0.03
2004	Portugal	Both	0.03	0.04	0.02
2004	Portugal	Female	0.01	0.02	0.01
2004	Portugal	Male	0.05	0.06	0.04
2004	Spain	Both	0.00	0.01	0.00
2004	Spain	Female	0.00	0.01	0.00
2004	Spain	Male	0.01	0.02	0.00
2005	Greece	Both	0.17	0.20	0.13
2005	Greece	Female	0.09	0.12	0.05
2005	Greece	Male	0.26	0.30	0.22
2005	Italy	Both	0.03	0.05	0.02
2005	Italy	Female	0.02	0.03	0.01
2005	Italy	Male	0.05	0.08	0.03
2005	Portugal	Both	0.02	0.03	0.02
2005	Portugal	Female	0.01	0.01	0.01
2005	Portugal	Male	0.03	0.04	0.03
2005	Spain	Both	0.00	0.01	0.00
2005	Spain	Female	0.00	0.01	0.00
2005	Spain	Male	0.01	0.02	0.00
2006	Greece	Both	0.22	0.26	0.17
2006	Greece	Female	0.12	0.16	0.07
2006	Greece	Male	0.33	0.38	0.27
2006	Italy	Both	0.03	0.05	0.02
2006	Italy	Female	0.02	0.03	0.01
2006	Italy	Male	0.05	0.08	0.03
2006	Portugal	Both	0.02	0.02	0.01
2006	Portugal	Female	0.01	0.01	0.00
2006	Portugal	Male	0.03	0.04	0.02
2006	Spain	Both	0.00	0.01	0.00
2006	Spain	Female	0.00	0.01	0.00
2006	Spain	Male	0.01	0.02	0.00
2007	Greece	Both	0.29	0.33	0.22
2007	Greece	Female	0.17	0.21	0.10
2007	Greece	Male	0.41	0.47	0.34
2007	Italy	Both	0.03	0.05	0.02
2007	Italy	Female	0.02	0.03	0.01
2007	Italy	Male	0.05	0.08	0.02
2007	Portugal	Both	0.01	0.02	0.01
2007	Portugal	Female	0.01	0.01	0.00
2007	Portugal	Male	0.02	0.03	0.02
2007	Spain	Both	0.00	0.01	0.00
2007	Spain	Female	0.00	0.01	0.00
2007	Spain	Male	0.01	0.02	0.00
2008	Greece	Both	0.29	0.34	0.23
2008	Greece	Female	0.17	0.21	0.10
2008	Greece	Male	0.42	0.48	0.35
2008	Italy	Both	0.03	0.05	0.02

2008	Italy	Female	0.02	0.03	0.01
2008	Italy	Male	0.05	0.08	0.02
2008	Portugal	Both	0.01	0.02	0.01
2008	Portugal	Female	0.01	0.01	0.00
2008	Portugal	Male	0.02	0.03	0.02
2008	Spain	Both	0.00	0.01	0.00
2008	Spain	Female	0.00	0.01	0.00
2008	Spain	Male	0.01	0.02	0.00
2009	Greece	Both	0.35	0.41	0.27
2009	Greece	Female	0.22	0.28	0.14
2009	Greece	Male	0.49	0.55	0.41
2009	Italy	Both	0.03	0.05	0.02
2009	Italy	Female	0.02	0.03	0.01
2009	Italy	Male	0.04	0.08	0.02
2009	Portugal	Both	0.01	0.02	0.01
2009	Portugal	Female	0.00	0.01	0.00
2009	Portugal	Male	0.02	0.03	0.02
2009	Spain	Both	0.01	0.01	0.00
2009	Spain	Female	0.00	0.01	0.00
2009	Spain	Male	0.01	0.02	0.01
2010	Greece	Both	0.37	0.43	0.29
2010	Greece	Female	0.24	0.30	0.15
2010	Greece	Male	0.52	0.59	0.44
2010	Italy	Both	0.03	0.05	0.02
2010	Italy	Female	0.01	0.03	0.01
2010	Italy	Male	0.04	0.07	0.02
2010	Portugal	Both	0.01	0.02	0.01
2010	Portugal	Female	0.00	0.01	0.00
2010	Portugal	Male	0.02	0.03	0.02
2010	Spain	Both	0.01	0.01	0.00
2010	Spain	Female	0.00	0.01	0.00
2010	Spain	Male	0.01	0.01	0.01
2011	Greece	Both	0.37	0.43	0.29
2011	Greece	Female	0.23	0.29	0.15
2011	Greece	Male	0.52	0.59	0.44
2011	Italy	Both	0.03	0.05	0.01
2011	Italy	Female	0.01	0.03	0.01
2011	Italy	Male	0.04	0.07	0.02
2011	Portugal	Both	0.01	0.02	0.01
2011	Portugal	Female	0.00	0.01	0.00
2011	Portugal	Male	0.02	0.03	0.02
2011	Spain	Both	0.01	0.01	0.00
2011	Spain	Female	0.00	0.01	0.00
2011	Spain	Male	0.01	0.01	0.00
2012	Greece	Both	0.34	0.39	0.27
2012	Greece	Female	0.21	0.26	0.14
2012	Greece	Male	0.48	0.54	0.41
2012	Italy	Both	0.03	0.04	0.01
2012	Italy	Female	0.01	0.03	0.01
2012	Italy	Male	0.04	0.07	0.02
2012	Portugal	Both	0.01	0.02	0.01
2012	Portugal	Female	0.00	0.01	0.00
2012	Portugal	Male	0.02	0.03	0.02

2012	Spain	Both	0.01	0.01	0.00
2012	Spain	Female	0.00	0.01	0.00
2012	Spain	Male	0.01	0.01	0.00
2013	Greece	Both	0.28	0.32	0.23
2013	Greece	Female	0.17	0.20	0.11
2013	Greece	Male	0.40	0.45	0.34
2013	Italy	Both	0.03	0.04	0.01
2013	Italy	Female	0.01	0.03	0.01
2013	Italy	Male	0.04	0.07	0.02
2013	Portugal	Both	0.01	0.01	0.01
2013	Portugal	Female	0.00	0.01	0.00
2013	Portugal	Male	0.02	0.02	0.02
2013	Spain	Both	0.01	0.01	0.00
2013	Spain	Female	0.00	0.01	0.00
2013	Spain	Male	0.01	0.01	0.00
2014	Greece	Both	0.17	0.19	0.14
2014	Greece	Female	0.10	0.12	0.06
2014	Greece	Male	0.25	0.28	0.21
2014	Italy	Both	0.03	0.04	0.01
2014	Italy	Female	0.01	0.02	0.01
2014	Italy	Male	0.04	0.06	0.02
2014	Portugal	Both	0.01	0.01	0.01
2014	Portugal	Female	0.00	0.01	0.00
2014	Portugal	Male	0.02	0.02	0.01
2014	Spain	Both	0.01	0.01	0.00
2014	Spain	Female	0.00	0.01	0.00
2014	Spain	Male	0.01	0.01	0.00
2015	Greece	Both	0.14	0.16	0.12
2015	Greece	Female	0.07	0.08	0.05
2015	Greece	Male	0.22	0.25	0.19
2015	Italy	Both	0.02	0.04	0.01
2015	Italy	Female	0.01	0.02	0.01
2015	Italy	Male	0.04	0.06	0.02
2015	Portugal	Both	0.01	0.01	0.01
2015	Portugal	Female	0.00	0.01	0.00
2015	Portugal	Male	0.02	0.02	0.01
2015	Spain	Both	0.01	0.01	0.00
2015	Spain	Female	0.00	0.00	0.00
2015	Spain	Male	0.01	0.01	0.00
2016	Greece	Both	0.13	0.15	0.11
2016	Greece	Female	0.06	0.08	0.04
2016	Greece	Male	0.21	0.23	0.17
2016	Italy	Both	0.02	0.03	0.02
2016	Italy	Female	0.01	0.02	0.01
2016	Italy	Male	0.04	0.05	0.02
2016	Portugal	Both	0.01	0.01	0.01
2016	Portugal	Female	0.00	0.01	0.00
2016	Portugal	Male	0.02	0.02	0.01
2016	Spain	Both	0.01	0.01	0.00
2016	Spain	Female	0.00	0.00	0.00
2016	Spain	Male	0.01	0.01	0.00
2017	Greece	Both	0.14	0.16	0.12
2017	Greece	Female	0.07	0.08	0.05

2017	Greece	Male	0.22	0.25	0.18
2017	Italy	Both	0.02	0.04	0.02
2017	Italy	Female	0.01	0.02	0.01
2017	Italy	Male	0.04	0.06	0.02
2017	Portugal	Both	0.01	0.01	0.01
2017	Portugal	Female	0.00	0.01	0.00
2017	Portugal	Male	0.02	0.02	0.01
2017	Spain	Both	0.01	0.01	0.00
2017	Spain	Female	0.00	0.00	0.00
2017	Spain	Male	0.01	0.01	0.00
2018	Greece	Both	0.14	0.16	0.12
2018	Greece	Female	0.07	0.09	0.05
2018	Greece	Male	0.22	0.25	0.18
2018	Italy	Both	0.03	0.04	0.02
2018	Italy	Female	0.01	0.02	0.01
2018	Italy	Male	0.04	0.06	0.02
2018	Portugal	Both	0.01	0.01	0.01
2018	Portugal	Female	0.00	0.01	0.00
2018	Portugal	Male	0.02	0.02	0.01
2018	Spain	Both	0.01	0.01	0.00
2018	Spain	Female	0.00	0.00	0.00
2018	Spain	Male	0.01	0.01	0.00
2019	Greece	Both	0.14	0.16	0.11
2019	Greece	Female	0.07	0.09	0.05
2019	Greece	Male	0.22	0.25	0.18
2019	Italy	Both	0.03	0.04	0.02
2019	Italy	Female	0.01	0.02	0.01
2019	Italy	Male	0.04	0.05	0.02
2019	Portugal	Both	0.01	0.01	0.01
2019	Portugal	Female	0.00	0.01	0.00
2019	Portugal	Male	0.02	0.02	0.01
2019	Spain	Both	0.01	0.01	0.00
2019	Spain	Female	0.00	0.00	0.00
2019	Spain	Male	0.01	0.01	0.00

Table S4- Age-standardized rates and 95% uncertainty levels (UL) of **acute HBV years of life lost** (YLLs) per 100,000 population in Greece, Italy, Portugal and Spain from 2000 to 2019 by sex classes (Global Burden of Disease Study 2019).

Year	Country	Sex	YLLs (95% UL)	95% UL (upper)	95% UL (lower)
2000	Greece	Both	2.70	3.01	2.32
2000	Greece	Female	1.22	1.43	0.86
2000	Greece	Male	4.27	4.78	3.69
2000	Italy	Both	1.51	1.80	1.03
2000	Italy	Female	1.00	1.21	0.53
2000	Italy	Male	2.05	2.49	1.37
2000	Portugal	Both	4.60	5.62	3.06
2000	Portugal	Female	2.13	2.58	1.37
2000	Portugal	Male	7.17	8.93	4.19
2000	Spain	Both	0.53	0.77	0.37
2000	Spain	Female	0.40	0.58	0.23
2000	Spain	Male	0.66	1.13	0.42
2001	Greece	Both	2.60	2.88	2.23
2001	Greece	Female	1.17	1.38	0.84
2001	Greece	Male	4.12	4.62	3.54
2001	Italy	Both	1.47	1.75	1.00
2001	Italy	Female	0.97	1.15	0.53
2001	Italy	Male	2.00	2.43	1.35
2001	Portugal	Both	2.23	2.60	1.70
2001	Portugal	Female	1.21	1.43	0.88
2001	Portugal	Male	3.30	4.02	2.32
2001	Spain	Both	0.49	0.77	0.35
2001	Spain	Female	0.34	0.55	0.22
2001	Spain	Male	0.63	1.14	0.42
2002	Greece	Both	2.76	3.07	2.37
2002	Greece	Female	1.29	1.52	0.93
2002	Greece	Male	4.33	4.84	3.73
2002	Italy	Both	1.45	1.72	0.98
2002	Italy	Female	0.92	1.07	0.53
2002	Italy	Male	2.02	2.53	1.31
2002	Portugal	Both	1.71	2.10	1.40
2002	Portugal	Female	0.99	1.21	0.78
2002	Portugal	Male	2.48	3.22	1.90
2002	Spain	Both	0.49	0.77	0.35
2002	Spain	Female	0.35	0.56	0.23
2002	Spain	Male	0.64	1.13	0.41
2003	Greece	Both	3.07	3.42	2.61
2003	Greece	Female	1.33	1.56	0.95
2003	Greece	Male	4.93	5.52	4.19
2003	Italy	Both	1.47	1.94	0.95
2003	Italy	Female	0.92	1.19	0.52
2003	Italy	Male	2.05	2.93	1.25
2003	Portugal	Both	1.47	1.80	1.23

2003	Portugal	Female	0.92	1.12	0.75
2003	Portugal	Male	2.05	2.68	1.66
2003	Spain	Both	0.45	0.76	0.33
2003	Spain	Female	0.33	0.56	0.22
2003	Spain	Male	0.58	1.08	0.38
2004	Greece	Both	3.66	4.08	3.05
2004	Greece	Female	1.45	1.70	1.06
2004	Greece	Male	6.01	6.78	4.98
2004	Italy	Both	1.44	2.02	0.91
2004	Italy	Female	0.91	1.26	0.50
2004	Italy	Male	2.00	3.02	1.20
2004	Portugal	Both	1.48	1.75	1.25
2004	Portugal	Female	0.84	1.01	0.70
2004	Portugal	Male	2.16	2.64	1.73
2004	Spain	Both	0.41	0.71	0.29
2004	Spain	Female	0.27	0.51	0.18
2004	Spain	Male	0.55	1.03	0.37
2005	Greece	Both	4.49	5.03	3.73
2005	Greece	Female	1.97	2.30	1.39
2005	Greece	Male	7.17	8.09	5.98
2005	Italy	Both	1.36	2.05	0.85
2005	Italy	Female	0.86	1.29	0.46
2005	Italy	Male	1.89	3.08	1.12
2005	Portugal	Both	1.02	1.32	0.86
2005	Portugal	Female	0.60	0.79	0.50
2005	Portugal	Male	1.47	1.97	1.21
2005	Spain	Both	0.39	0.69	0.28
2005	Spain	Female	0.27	0.49	0.17
2005	Spain	Male	0.52	0.96	0.35
2006	Greece	Both	5.65	6.31	4.68
2006	Greece	Female	2.50	2.93	1.72
2006	Greece	Male	8.99	10.0	7.61
2006	Italy	Both	1.34	2.11	0.81
2006	Italy	Female	0.83	1.34	0.44
2006	Italy	Male	1.87	3.15	1.08
2006	Portugal	Both	0.84	1.10	0.70
2006	Portugal	Female	0.51	0.69	0.41
2006	Portugal	Male	1.21	1.63	0.98
2006	Spain	Both	0.41	0.67	0.30
2006	Spain	Female	0.28	0.47	0.19
2006	Spain	Male	0.54	0.95	0.38
2007	Greece	Both	7.02	7.82	5.83
2007	Greece	Female	3.35	3.89	2.34
2007	Greece	Male	10.9	12.2	9.15
2007	Italy	Both	1.30	2.11	0.77
2007	Italy	Female	0.80	1.33	0.42
2007	Italy	Male	1.82	3.14	1.03
2007	Portugal	Both	0.77	1.05	0.62
2007	Portugal	Female	0.46	0.65	0.36
2007	Portugal	Male	1.11	1.55	0.88

2007	Spain	Both	0.39	0.64	0.29
2007	Spain	Female	0.28	0.47	0.19
2007	Spain	Male	0.51	0.91	0.35
2008	Greece	Both	7.15	7.96	5.95
2008	Greece	Female	3.33	3.88	2.34
2008	Greece	Male	11.1	12.5	9.43
2008	Italy	Both	1.26	2.05	0.74
2008	Italy	Female	0.76	1.31	0.39
2008	Italy	Male	1.79	3.01	1.02
2008	Portugal	Both	0.73	1.00	0.58
2008	Portugal	Female	0.42	0.61	0.33
2008	Portugal	Male	1.07	1.48	0.84
2008	Spain	Both	0.41	0.62	0.31
2008	Spain	Female	0.30	0.46	0.20
2008	Spain	Male	0.52	0.87	0.38
2009	Greece	Both	8.52	9.45	7.17
2009	Greece	Female	4.33	5.05	3.10
2009	Greece	Male	12.9	14.4	10.9
2009	Italy	Both	1.18	1.94	0.71
2009	Italy	Female	0.71	1.25	0.37
2009	Italy	Male	1.68	2.84	0.98
2009	Portugal	Both	0.73	0.98	0.60
2009	Portugal	Female	0.41	0.60	0.32
2009	Portugal	Male	1.08	1.43	0.88
2009	Spain	Both	0.43	0.60	0.34
2009	Spain	Female	0.33	0.46	0.21
2009	Spain	Male	0.53	0.81	0.39
2010	Greece	Both	9.03	9.99	7.71
2010	Greece	Female	4.60	5.38	3.37
2010	Greece	Male	13.7	15.2	11.7
2010	Italy	Both	1.16	1.89	0.70
2010	Italy	Female	0.69	1.23	0.37
2010	Italy	Male	1.66	2.77	0.97
2010	Portugal	Both	0.73	0.95	0.60
2010	Portugal	Female	0.40	0.59	0.31
2010	Portugal	Male	1.08	1.40	0.90
2010	Spain	Both	0.44	0.57	0.34
2010	Spain	Female	0.33	0.45	0.20
2010	Spain	Male	0.55	0.77	0.40
2011	Greece	Both	8.95	9.89	7.67
2011	Greece	Female	4.60	5.35	3.44
2011	Greece	Male	13.5	15.1	11.7
2011	Italy	Both	1.11	1.81	0.68
2011	Italy	Female	0.67	1.23	0.36
2011	Italy	Male	1.57	2.60	0.95
2011	Portugal	Both	0.66	0.85	0.56
2011	Portugal	Female	0.38	0.54	0.30
2011	Portugal	Male	0.97	1.23	0.81
2011	Spain	Both	0.46	0.57	0.34
2011	Spain	Female	0.33	0.43	0.20

2011	Spain	Male	0.60	0.77	0.40
2012	Greece	Both	8.25	9.12	7.13
2012	Greece	Female	4.18	4.89	3.23
2012	Greece	Male	12.5	14.0	10.8
2012	Italy	Both	1.08	1.75	0.67
2012	Italy	Female	0.66	1.17	0.36
2012	Italy	Male	1.52	2.52	0.93
2012	Portugal	Both	0.67	0.84	0.58
2012	Portugal	Female	0.37	0.51	0.29
2012	Portugal	Male	1.01	1.25	0.85
2012	Spain	Both	0.46	0.57	0.33
2012	Spain	Female	0.32	0.42	0.19
2012	Spain	Male	0.61	0.78	0.39
2013	Greece	Both	6.90	7.64	6.04
2013	Greece	Female	3.36	3.96	2.68
2013	Greece	Male	10.6	11.8	9.33
2013	Italy	Both	1.05	1.67	0.66
2013	Italy	Female	0.65	1.11	0.36
2013	Italy	Male	1.47	2.40	0.92
2013	Portugal	Both	0.62	0.76	0.54
2013	Portugal	Female	0.35	0.48	0.28
2013	Portugal	Male	0.91	1.11	0.77
2013	Spain	Both	0.45	0.56	0.31
2013	Spain	Female	0.32	0.42	0.18
2013	Spain	Male	0.59	0.75	0.38
2014	Greece	Both	4.35	4.83	3.83
2014	Greece	Female	2.07	2.47	1.71
2014	Greece	Male	6.78	7.59	5.96
2014	Italy	Both	1.03	1.59	0.67
2014	Italy	Female	0.60	1.03	0.34
2014	Italy	Male	1.48	2.31	0.95
2014	Portugal	Both	0.57	0.71	0.49
2014	Portugal	Female	0.35	0.46	0.28
2014	Portugal	Male	0.82	1.02	0.68
2014	Spain	Both	0.45	0.55	0.31
2014	Spain	Female	0.33	0.43	0.18
2014	Spain	Male	0.58	0.72	0.36
2015	Greece	Both	3.69	4.15	3.26
2015	Greece	Female	1.57	1.92	1.32
2015	Greece	Male	5.97	6.70	5.19
2015	Italy	Both	0.98	1.51	0.66
2015	Italy	Female	0.59	1.02	0.35
2015	Italy	Male	1.38	2.14	0.92
2015	Portugal	Both	0.56	0.68	0.47
2015	Portugal	Female	0.34	0.45	0.28
2015	Portugal	Male	0.79	0.99	0.64
2015	Spain	Both	0.43	0.53	0.30
2015	Spain	Female	0.31	0.40	0.18
2015	Spain	Male	0.55	0.69	0.35
2016	Greece	Both	3.52	3.94	3.08

2016	Greece	Female	1.51	1.85	1.26
2016	Greece	Male	5.67	6.39	4.90
2016	Italy	Both	0.96	1.34	0.68
2016	Italy	Female	0.54	0.89	0.34
2016	Italy	Male	1.39	1.92	0.97
2016	Portugal	Both	0.53	0.67	0.44
2016	Portugal	Female	0.33	0.43	0.27
2016	Portugal	Male	0.75	0.95	0.59
2016	Spain	Both	0.41	0.51	0.30
2016	Spain	Female	0.29	0.39	0.17
2016	Spain	Male	0.54	0.68	0.35
2017	Greece	Both	3.77	4.23	3.29
2017	Greece	Female	1.62	1.96	1.32
2017	Greece	Male	6.07	6.88	5.19
2017	Italy	Both	1.02	1.41	0.70
2017	Italy	Female	0.60	0.94	0.35
2017	Italy	Male	1.46	2.03	0.98
2017	Portugal	Both	0.55	0.68	0.45
2017	Portugal	Female	0.33	0.42	0.26
2017	Portugal	Male	0.79	1.00	0.62
2017	Spain	Both	0.42	0.52	0.30
2017	Spain	Female	0.30	0.39	0.18
2017	Spain	Male	0.55	0.70	0.35
2018	Greece	Both	3.80	4.29	3.30
2018	Greece	Female	1.65	1.99	1.31
2018	Greece	Male	6.10	6.92	5.19
2018	Italy	Both	1.03	1.42	0.71
2018	Italy	Female	0.61	0.94	0.36
2018	Italy	Male	1.48	2.05	0.98
2018	Portugal	Both	0.57	0.71	0.47
2018	Portugal	Female	0.34	0.43	0.27
2018	Portugal	Male	0.82	1.03	0.65
2018	Spain	Both	0.42	0.53	0.30
2018	Spain	Female	0.30	0.39	0.18
2018	Spain	Male	0.55	0.71	0.36
2019	Greece	Both	3.78	4.29	3.27
2019	Greece	Female	1.64	1.98	1.29
2019	Greece	Male	6.07	6.92	5.11
2019	Italy	Both	1.04	1.40	0.72
2019	Italy	Female	0.61	0.92	0.37
2019	Italy	Male	1.49	2.03	0.99
2019	Portugal	Both	0.58	0.72	0.48
2019	Portugal	Female	0.34	0.43	0.27
2019	Portugal	Male	0.85	1.05	0.66
2019	Spain	Both	0.42	0.53	0.30
2019	Spain	Female	0.29	0.40	0.18
2019	Spain	Male	0.55	0.71	0.36

Table S5- Age-standardized rates and 95% uncertainty levels (UL) of **acute HBV years lived with disability** (YLDs) per 100,000 population in Greece, Italy, Portugal and Spain from 2000 to 2019 by sex classes (Global Burden of Disease Study 2019).

Year	Country	Sex	YLDs (95% UL)	95% UL (upper)	95% UL (lower)
2000	Greece	Both	1.12	1.79	0.65
2000	Greece	Female	0.92	1.56	0.49
2000	Greece	Male	1.32	2.17	0.74
2000	Italy	Both	0.44	0.72	0.26
2000	Italy	Female	0.35	0.58	0.19
2000	Italy	Male	0.54	0.87	0.31
2000	Portugal	Both	0.44	0.76	0.24
2000	Portugal	Female	0.31	0.54	0.15
2000	Portugal	Male	0.58	1.03	0.31
2000	Spain	Both	0.48	0.79	0.27
2000	Spain	Female	0.38	0.62	0.22
2000	Spain	Male	0.58	0.97	0.31
2001	Greece	Both	1.11	1.76	0.65
2001	Greece	Female	0.91	1.49	0.51
2001	Greece	Male	1.31	2.09	0.75
2001	Italy	Both	0.43	0.69	0.25
2001	Italy	Female	0.34	0.56	0.19
2001	Italy	Male	0.53	0.85	0.30
2001	Portugal	Both	0.44	0.73	0.24
2001	Portugal	Female	0.30	0.53	0.15
2001	Portugal	Male	0.58	1.00	0.31
2001	Spain	Both	0.48	0.77	0.27
2001	Spain	Female	0.38	0.61	0.22
2001	Spain	Male	0.57	0.95	0.32
2002	Greece	Both	1.10	1.76	0.65
2002	Greece	Female	0.91	1.47	0.53
2002	Greece	Male	1.29	2.08	0.73
2002	Italy	Both	0.42	0.68	0.24
2002	Italy	Female	0.33	0.53	0.18
2002	Italy	Male	0.51	0.82	0.29
2002	Portugal	Both	0.44	0.72	0.24
2002	Portugal	Female	0.30	0.51	0.15
2002	Portugal	Male	0.58	1.00	0.31
2002	Spain	Both	0.47	0.75	0.27
2002	Spain	Female	0.37	0.59	0.22
2002	Spain	Male	0.57	0.94	0.32
2003	Greece	Both	1.08	1.69	0.63
2003	Greece	Female	0.89	1.45	0.50
2003	Greece	Male	1.27	2.03	0.74
2003	Italy	Both	0.41	0.65	0.23
2003	Italy	Female	0.32	0.52	0.18
2003	Italy	Male	0.50	0.79	0.28
2003	Portugal	Both	0.44	0.73	0.24
2003	Portugal	Female	0.29	0.50	0.15
2003	Portugal	Male	0.58	1.01	0.31
2003	Spain	Both	0.47	0.75	0.27

2003	Spain	Female	0.37	0.60	0.21
2003	Spain	Male	0.56	0.92	0.31
2004	Greece	Both	1.07	1.73	0.63
2004	Greece	Female	0.88	1.44	0.51
2004	Greece	Male	1.26	2.11	0.71
2004	Italy	Both	0.40	0.64	0.23
2004	Italy	Female	0.31	0.52	0.17
2004	Italy	Male	0.48	0.77	0.27
2004	Portugal	Both	0.43	0.72	0.24
2004	Portugal	Female	0.29	0.50	0.15
2004	Portugal	Male	0.58	1.01	0.31
2004	Spain	Both	0.46	0.76	0.26
2004	Spain	Female	0.37	0.60	0.21
2004	Spain	Male	0.55	0.94	0.29
2005	Greece	Both	1.06	1.73	0.61
2005	Greece	Female	0.88	1.42	0.50
2005	Greece	Male	1.25	2.10	0.69
2005	Italy	Both	0.39	0.63	0.22
2005	Italy	Female	0.31	0.51	0.17
2005	Italy	Male	0.47	0.77	0.26
2005	Portugal	Both	0.43	0.71	0.23
2005	Portugal	Female	0.29	0.50	0.15
2005	Portugal	Male	0.58	1.03	0.29
2005	Spain	Both	0.46	0.75	0.26
2005	Spain	Female	0.37	0.61	0.20
2005	Spain	Male	0.55	0.94	0.29
2006	Greece	Both	1.05	1.68	0.61
2006	Greece	Female	0.86	1.39	0.50
2006	Greece	Male	1.24	2.07	0.70
2006	Italy	Both	0.38	0.61	0.21
2006	Italy	Female	0.30	0.50	0.17
2006	Italy	Male	0.45	0.74	0.25
2006	Portugal	Both	0.43	0.69	0.23
2006	Portugal	Female	0.28	0.49	0.14
2006	Portugal	Male	0.57	0.97	0.29
2006	Spain	Both	0.45	0.74	0.26
2006	Spain	Female	0.36	0.59	0.20
2006	Spain	Male	0.54	0.92	0.30
2007	Greece	Both	1.04	1.64	0.62
2007	Greece	Female	0.85	1.35	0.49
2007	Greece	Male	1.23	1.99	0.72
2007	Italy	Both	0.36	0.57	0.20
2007	Italy	Female	0.29	0.48	0.16
2007	Italy	Male	0.43	0.69	0.24
2007	Portugal	Both	0.42	0.69	0.23
2007	Portugal	Female	0.28	0.48	0.14
2007	Portugal	Male	0.57	0.94	0.29
2007	Spain	Both	0.44	0.72	0.25
2007	Spain	Female	0.35	0.57	0.20
2007	Spain	Male	0.53	0.89	0.29
2008	Greece	Both	1.03	1.64	0.60
2008	Greece	Female	0.83	1.35	0.48
2008	Greece	Male	1.22	2.00	0.69

2008	Italy	Both	0.34	0.55	0.19
2008	Italy	Female	0.28	0.47	0.15
2008	Italy	Male	0.41	0.65	0.23
2008	Portugal	Both	0.41	0.68	0.22
2008	Portugal	Female	0.28	0.47	0.14
2008	Portugal	Male	0.55	0.93	0.29
2008	Spain	Both	0.43	0.70	0.25
2008	Spain	Female	0.34	0.55	0.19
2008	Spain	Male	0.52	0.86	0.28
2009	Greece	Both	1.02	1.61	0.57
2009	Greece	Female	0.82	1.33	0.44
2009	Greece	Male	1.21	1.96	0.66
2009	Italy	Both	0.33	0.52	0.18
2009	Italy	Female	0.27	0.45	0.14
2009	Italy	Male	0.39	0.62	0.22
2009	Portugal	Both	0.40	0.66	0.21
2009	Portugal	Female	0.27	0.48	0.14
2009	Portugal	Male	0.54	0.90	0.28
2009	Spain	Both	0.42	0.70	0.24
2009	Spain	Female	0.33	0.55	0.19
2009	Spain	Male	0.51	0.86	0.27
2010	Greece	Both	1.01	1.65	0.59
2010	Greece	Female	0.81	1.36	0.42
2010	Greece	Male	1.20	1.98	0.67
2010	Italy	Both	0.32	0.51	0.18
2010	Italy	Female	0.26	0.45	0.13
2010	Italy	Male	0.37	0.61	0.20
2010	Portugal	Both	0.40	0.67	0.20
2010	Portugal	Female	0.27	0.48	0.13
2010	Portugal	Male	0.53	0.91	0.26
2010	Spain	Both	0.42	0.69	0.23
2010	Spain	Female	0.33	0.55	0.18
2010	Spain	Male	0.51	0.86	0.25
2011	Greece	Both	1.01	1.61	0.59
2011	Greece	Female	0.82	1.35	0.45
2011	Greece	Male	1.20	1.99	0.66
2011	Italy	Both	0.31	0.49	0.17
2011	Italy	Female	0.25	0.43	0.13
2011	Italy	Male	0.36	0.59	0.20
2011	Portugal	Both	0.39	0.67	0.20
2011	Portugal	Female	0.26	0.47	0.13
2011	Portugal	Male	0.53	0.92	0.27
2011	Spain	Both	0.42	0.67	0.23
2011	Spain	Female	0.33	0.54	0.19
2011	Spain	Male	0.50	0.83	0.26
2012	Greece	Both	1.00	1.60	0.58
2012	Greece	Female	0.81	1.32	0.45
2012	Greece	Male	1.19	1.93	0.66
2012	Italy	Both	0.29	0.47	0.16
2012	Italy	Female	0.24	0.40	0.13
2012	Italy	Male	0.35	0.56	0.19
2012	Portugal	Both	0.39	0.65	0.21
2012	Portugal	Female	0.26	0.45	0.13

2012	Portugal	Male	0.52	0.91	0.27
2012	Spain	Both	0.41	0.65	0.24
2012	Spain	Female	0.32	0.53	0.18
2012	Spain	Male	0.50	0.82	0.27
2013	Greece	Both	0.99	1.55	0.58
2013	Greece	Female	0.80	1.26	0.45
2013	Greece	Male	1.18	1.91	0.66
2013	Italy	Both	0.28	0.45	0.15
2013	Italy	Female	0.23	0.39	0.12
2013	Italy	Male	0.33	0.54	0.18
2013	Portugal	Both	0.38	0.64	0.20
2013	Portugal	Female	0.25	0.45	0.13
2013	Portugal	Male	0.52	0.87	0.26
2013	Spain	Both	0.41	0.67	0.24
2013	Spain	Female	0.32	0.53	0.18
2013	Spain	Male	0.50	0.84	0.27
2014	Greece	Both	0.97	1.57	0.57
2014	Greece	Female	0.79	1.26	0.45
2014	Greece	Male	1.16	1.94	0.63
2014	Italy	Both	0.27	0.44	0.15
2014	Italy	Female	0.22	0.37	0.11
2014	Italy	Male	0.32	0.52	0.17
2014	Portugal	Both	0.38	0.63	0.19
2014	Portugal	Female	0.25	0.45	0.13
2014	Portugal	Male	0.51	0.88	0.25
2014	Spain	Both	0.41	0.68	0.24
2014	Spain	Female	0.32	0.52	0.17
2014	Spain	Male	0.50	0.84	0.27
2015	Greece	Both	0.97	1.57	0.55
2015	Greece	Female	0.78	1.28	0.44
2015	Greece	Male	1.15	1.92	0.61
2015	Italy	Both	0.26	0.43	0.14
2015	Italy	Female	0.21	0.36	0.11
2015	Italy	Male	0.31	0.51	0.17
2015	Portugal	Both	0.37	0.63	0.18
2015	Portugal	Female	0.24	0.45	0.12
2015	Portugal	Male	0.50	0.87	0.23
2015	Spain	Both	0.41	0.67	0.23
2015	Spain	Female	0.32	0.54	0.17
2015	Spain	Male	0.49	0.84	0.26
2016	Greece	Both	0.96	1.50	0.57
2016	Greece	Female	0.78	1.23	0.45
2016	Greece	Male	1.14	1.85	0.64
2016	Italy	Both	0.24	0.39	0.13
2016	Italy	Female	0.19	0.32	0.10
2016	Italy	Male	0.28	0.48	0.15
2016	Portugal	Both	0.36	0.60	0.18
2016	Portugal	Female	0.24	0.41	0.13
2016	Portugal	Male	0.49	0.83	0.23
2016	Spain	Both	0.38	0.60	0.21
2016	Spain	Female	0.30	0.48	0.17
2016	Spain	Male	0.46	0.75	0.25
2017	Greece	Both	0.95	1.53	0.54

2017	Greece	Female	0.78	1.28	0.43
2017	Greece	Male	1.13	1.93	0.60
2017	Italy	Both	0.22	0.36	0.12
2017	Italy	Female	0.18	0.30	0.09
2017	Italy	Male	0.26	0.43	0.14
2017	Portugal	Both	0.35	0.60	0.17
2017	Portugal	Female	0.24	0.42	0.12
2017	Portugal	Male	0.47	0.87	0.22
2017	Spain	Both	0.36	0.58	0.20
2017	Spain	Female	0.29	0.46	0.16
2017	Spain	Male	0.43	0.72	0.22
2018	Greece	Both	0.95	1.52	0.55
2018	Greece	Female	0.78	1.25	0.44
2018	Greece	Male	1.12	1.90	0.62
2018	Italy	Both	0.21	0.35	0.11
2018	Italy	Female	0.17	0.29	0.09
2018	Italy	Male	0.25	0.41	0.14
2018	Portugal	Both	0.34	0.58	0.17
2018	Portugal	Female	0.24	0.39	0.12
2018	Portugal	Male	0.46	0.82	0.22
2018	Spain	Both	0.35	0.56	0.20
2018	Spain	Female	0.28	0.46	0.16
2018	Spain	Male	0.43	0.71	0.22
2019	Greece	Both	0.94	1.48	0.56
2019	Greece	Female	0.77	1.29	0.43
2019	Greece	Male	1.12	1.83	0.61
2019	Italy	Both	0.21	0.35	0.11
2019	Italy	Female	0.17	0.31	0.09
2019	Italy	Male	0.25	0.42	0.13
2019	Portugal	Both	0.33	0.58	0.17
2019	Portugal	Female	0.22	0.41	0.10
2019	Portugal	Male	0.46	0.82	0.21
2019	Spain	Both	0.35	0.58	0.20
2019	Spain	Female	0.28	0.48	0.14
2019	Spain	Male	0.43	0.74	0.22

Table S6- Age-standardized rates and 95% uncertainty levels (UL) of **acute HBV disability-adjusted life years** (DALYs) per 100,000 population in Greece, Italy, Portugal and Spain from 2000 to 2019 by sex classes (Global Burden of Disease Study 2019).

Year	Country	Sex	DALYs (95% UL)	95% UL (upper)	95% UL (lower)
2000	Greece	Both	3.83	4.53	3.24
2000	Greece	Female	2.14	2.82	1.62
2000	Greece	Male	5.60	6.55	4.78
2000	Italy	Both	1.96	2.40	1.46
2000	Italy	Female	1.36	1.70	0.86
2000	Italy	Male	2.60	3.21	1.85
2000	Portugal	Both	5.05	6.12	3.49
2000	Portugal	Female	2.45	3.00	1.69
2000	Portugal	Male	7.76	9.60	4.79
2000	Spain	Both	1.01	1.35	0.76
2000	Spain	Female	0.78	1.07	0.57
2000	Spain	Male	1.24	1.78	0.86
2001	Greece	Both	3.72	4.44	3.15
2001	Greece	Female	2.09	2.71	1.61
2001	Greece	Male	5.43	6.34	4.63
2001	Italy	Both	1.91	2.33	1.42
2001	Italy	Female	1.31	1.62	0.86
2001	Italy	Male	2.54	3.14	1.82
2001	Portugal	Both	2.67	3.15	2.11
2001	Portugal	Female	1.51	1.83	1.17
2001	Portugal	Male	3.89	4.68	2.88
2001	Spain	Both	0.97	1.34	0.70
2001	Spain	Female	0.72	1.01	0.52
2001	Spain	Male	1.21	1.75	0.85
2002	Greece	Both	3.87	4.57	3.29
2002	Greece	Female	2.20	2.85	1.70
2002	Greece	Male	5.63	6.54	4.79
2002	Italy	Both	1.88	2.30	1.38
2002	Italy	Female	1.25	1.54	0.83
2002	Italy	Male	2.54	3.14	1.75
2002	Portugal	Both	2.16	2.63	1.78
2002	Portugal	Female	1.29	1.58	1.05
2002	Portugal	Male	3.07	3.86	2.42
2002	Spain	Both	0.97	1.34	0.72
2002	Spain	Female	0.73	1.02	0.52
2002	Spain	Male	1.21	1.74	0.85
2003	Greece	Both	4.16	4.86	3.51
2003	Greece	Female	2.23	2.80	1.74
2003	Greece	Male	6.20	7.16	5.25
2003	Italy	Both	1.88	2.39	1.34
2003	Italy	Female	1.25	1.58	0.83
2003	Italy	Male	2.56	3.44	1.70
2003	Portugal	Both	1.91	2.34	1.59
2003	Portugal	Female	1.22	1.48	1.00
2003	Portugal	Male	2.64	3.38	2.15
2003	Spain	Both	0.92	1.31	0.66
2003	Spain	Female	0.70	1.00	0.50

2003	Spain	Male	1.14	1.70	0.78
2004	Greece	Both	4.73	5.49	3.98
2004	Greece	Female	2.33	2.92	1.84
2004	Greece	Male	7.27	8.41	6.05
2004	Italy	Both	1.84	2.45	1.28
2004	Italy	Female	1.23	1.59	0.80
2004	Italy	Male	2.48	3.54	1.64
2004	Portugal	Both	1.92	2.32	1.62
2004	Portugal	Female	1.13	1.37	0.94
2004	Portugal	Male	2.74	3.38	2.24
2004	Spain	Both	0.87	1.26	0.61
2004	Spain	Female	0.64	0.94	0.44
2004	Spain	Male	1.10	1.67	0.75
2005	Greece	Both	5.56	6.41	4.68
2005	Greece	Female	2.85	3.52	2.18
2005	Greece	Male	8.43	9.66	7.06
2005	Italy	Both	1.75	2.49	1.20
2005	Italy	Female	1.17	1.60	0.75
2005	Italy	Male	2.37	3.57	1.54
2005	Portugal	Both	1.46	1.87	1.17
2005	Portugal	Female	0.90	1.14	0.70
2005	Portugal	Male	2.05	2.68	1.61
2005	Spain	Both	0.85	1.26	0.58
2005	Spain	Female	0.64	0.93	0.43
2005	Spain	Male	1.07	1.65	0.71
2006	Greece	Both	6.71	7.67	5.66
2006	Greece	Female	3.37	4.08	2.50
2006	Greece	Male	10.2	11.6	8.65
2006	Italy	Both	1.72	2.52	1.16
2006	Italy	Female	1.14	1.62	0.73
2006	Italy	Male	2.33	3.61	1.49
2006	Portugal	Both	1.27	1.66	1.00
2006	Portugal	Female	0.80	1.04	0.61
2006	Portugal	Male	1.78	2.34	1.38
2006	Spain	Both	0.87	1.23	0.61
2006	Spain	Female	0.64	0.92	0.44
2006	Spain	Male	1.09	1.60	0.76
2007	Greece	Both	8.07	9.09	6.78
2007	Greece	Female	4.21	4.95	3.11
2007	Greece	Male	12.1	13.6	10.2
2007	Italy	Both	1.66	2.50	1.11
2007	Italy	Female	1.09	1.63	0.70
2007	Italy	Male	2.26	3.57	1.41
2007	Portugal	Both	1.20	1.60	0.91
2007	Portugal	Female	0.74	1.01	0.55
2007	Portugal	Male	1.68	2.27	1.27
2007	Spain	Both	0.84	1.19	0.59
2007	Spain	Female	0.63	0.89	0.44
2007	Spain	Male	1.04	1.56	0.71
2008	Greece	Both	8.18	9.22	6.92
2008	Greece	Female	4.17	4.92	3.12
2008	Greece	Male	12.4	13.8	10.5
2008	Italy	Both	1.61	2.44	1.07

2008	Italy	Female	1.04	1.61	0.68
2008	Italy	Male	2.21	3.44	1.38
2008	Portugal	Both	1.15	1.54	0.87
2008	Portugal	Female	0.70	0.95	0.51
2008	Portugal	Male	1.62	2.20	1.23
2008	Spain	Both	0.84	1.17	0.62
2008	Spain	Female	0.64	0.88	0.46
2008	Spain	Male	1.05	1.52	0.74
2009	Greece	Both	9.54	10.7	8.07
2009	Greece	Female	5.16	6.03	3.90
2009	Greece	Male	14.1	15.8	12.0
2009	Italy	Both	1.51	2.32	1.02
2009	Italy	Female	0.98	1.53	0.64
2009	Italy	Male	2.08	3.26	1.32
2009	Portugal	Both	1.14	1.49	0.89
2009	Portugal	Female	0.69	0.94	0.49
2009	Portugal	Male	1.63	2.12	1.28
2009	Spain	Both	0.86	1.17	0.65
2009	Spain	Female	0.67	0.89	0.48
2009	Spain	Male	1.05	1.47	0.76
2010	Greece	Both	10.0	11.2	8.56
2010	Greece	Female	5.42	6.32	4.12
2010	Greece	Male	14.9	16.6	12.8
2010	Italy	Both	1.48	2.26	1.00
2010	Italy	Female	0.95	1.51	0.61
2010	Italy	Male	2.04	3.18	1.31
2010	Portugal	Both	1.13	1.50	0.88
2010	Portugal	Female	0.67	0.95	0.47
2010	Portugal	Male	1.62	2.11	1.28
2010	Spain	Both	0.86	1.16	0.65
2010	Spain	Female	0.66	0.90	0.48
2010	Spain	Male	1.07	1.49	0.78
2011	Greece	Both	9.96	11.1	8.54
2011	Greece	Female	5.42	6.31	4.21
2011	Greece	Male	14.7	16.5	12.8
2011	Italy	Both	1.42	2.16	0.97
2011	Italy	Female	0.93	1.48	0.61
2011	Italy	Male	1.94	2.99	1.27
2011	Portugal	Both	1.06	1.39	0.83
2011	Portugal	Female	0.65	0.90	0.47
2011	Portugal	Male	1.50	1.94	1.17
2011	Spain	Both	0.88	1.16	0.67
2011	Spain	Female	0.66	0.89	0.48
2011	Spain	Male	1.11	1.48	0.82
2012	Greece	Both	9.25	10.3	8.01
2012	Greece	Female	4.99	5.84	3.94
2012	Greece	Male	13.7	15.4	11.9
2012	Italy	Both	1.38	2.08	0.94
2012	Italy	Female	0.91	1.41	0.59
2012	Italy	Male	1.87	2.89	1.23
2012	Portugal	Both	1.07	1.39	0.84
2012	Portugal	Female	0.63	0.87	0.46
2012	Portugal	Male	1.54	1.97	1.21

2012	Spain	Both	0.88	1.16	0.67
2012	Spain	Female	0.65	0.88	0.48
2012	Spain	Male	1.12	1.46	0.81
2013	Greece	Both	7.89	8.82	6.90
2013	Greece	Female	4.16	4.87	3.37
2013	Greece	Male	11.8	13.2	10.3
2013	Italy	Both	1.33	1.99	0.92
2013	Italy	Female	0.88	1.34	0.58
2013	Italy	Male	1.81	2.79	1.20
2013	Portugal	Both	1.01	1.29	0.79
2013	Portugal	Female	0.61	0.83	0.45
2013	Portugal	Male	1.44	1.82	1.12
2013	Spain	Both	0.87	1.14	0.65
2013	Spain	Female	0.65	0.87	0.47
2013	Spain	Male	1.10	1.46	0.80
2014	Greece	Both	5.33	6.08	4.69
2014	Greece	Female	2.86	3.46	2.34
2014	Greece	Male	7.95	8.99	7.00
2014	Italy	Both	1.30	1.89	0.91
2014	Italy	Female	0.82	1.27	0.55
2014	Italy	Male	1.80	2.66	1.23
2014	Portugal	Both	0.95	1.24	0.73
2014	Portugal	Female	0.60	0.81	0.44
2014	Portugal	Male	1.33	1.75	1.02
2014	Spain	Both	0.87	1.13	0.65
2014	Spain	Female	0.66	0.89	0.46
2014	Spain	Male	1.08	1.43	0.78
2015	Greece	Both	4.66	5.43	4.06
2015	Greece	Female	2.36	2.92	1.91
2015	Greece	Male	7.13	8.17	6.19
2015	Italy	Both	1.24	1.79	0.90
2015	Italy	Female	0.81	1.26	0.55
2015	Italy	Male	1.70	2.47	1.18
2015	Portugal	Both	0.93	1.20	0.72
2015	Portugal	Female	0.59	0.80	0.43
2015	Portugal	Male	1.29	1.69	0.97
2015	Spain	Both	0.84	1.12	0.62
2015	Spain	Female	0.63	0.88	0.45
2015	Spain	Male	1.05	1.42	0.75
2016	Greece	Both	4.48	5.14	3.90
2016	Greece	Female	2.30	2.84	1.86
2016	Greece	Male	6.81	7.86	5.90
2016	Italy	Both	1.20	1.61	0.91
2016	Italy	Female	0.74	1.10	0.52
2016	Italy	Male	1.68	2.22	1.23
2016	Portugal	Both	0.90	1.16	0.69
2016	Portugal	Female	0.58	0.77	0.44
2016	Portugal	Male	1.24	1.63	0.93
2016	Spain	Both	0.80	1.03	0.60
2016	Spain	Female	0.60	0.81	0.43
2016	Spain	Male	1.01	1.31	0.74
2017	Greece	Both	4.73	5.41	4.12
2017	Greece	Female	2.40	2.96	1.93

2017	Greece	Male	7.21	8.23	6.17
2017	Italy	Both	1.24	1.66	0.91
2017	Italy	Female	0.78	1.11	0.52
2017	Italy	Male	1.72	2.32	1.23
2017	Portugal	Both	0.91	1.17	0.69
2017	Portugal	Female	0.58	0.78	0.43
2017	Portugal	Male	1.27	1.69	0.95
2017	Spain	Both	0.78	1.03	0.59
2017	Spain	Female	0.59	0.80	0.42
2017	Spain	Male	0.98	1.34	0.71
2018	Greece	Both	4.75	5.47	4.13
2018	Greece	Female	2.43	2.95	1.95
2018	Greece	Male	7.23	8.34	6.17
2018	Italy	Both	1.25	1.66	0.92
2018	Italy	Female	0.79	1.09	0.52
2018	Italy	Male	1.73	2.32	1.23
2018	Portugal	Both	0.92	1.19	0.72
2018	Portugal	Female	0.58	0.76	0.44
2018	Portugal	Male	1.29	1.68	0.99
2018	Spain	Both	0.78	1.02	0.58
2018	Spain	Female	0.58	0.77	0.42
2018	Spain	Male	0.98	1.31	0.71
2019	Greece	Both	4.73	5.42	4.08
2019	Greece	Female	2.42	3.00	1.95
2019	Greece	Male	7.19	8.31	6.08
2019	Italy	Both	1.26	1.64	0.92
2019	Italy	Female	0.79	1.09	0.52
2019	Italy	Male	1.74	2.30	1.24
2019	Portugal	Both	0.92	1.18	0.72
2019	Portugal	Female	0.56	0.77	0.41
2019	Portugal	Male	1.31	1.71	0.99
2019	Spain	Both	0.78	1.02	0.57
2019	Spain	Female	0.58	0.79	0.41
2019	Spain	Male	0.99	1.34	0.69

Table S7- Age-standardized rates and 95% uncertainty levels (UL) of **acute HCV prevalence**, per 100,000 population in Greece, Italy, Portugal and Spain from 2000 to 2019 by sex classes (Global Burden of Disease Study 2019).

Year	Country	Sex	Prevalence (95% UL)	95% UL (upper)	95% UL (lower)
2000	Greece	Both	2.1	2.4	1.9
2000	Greece	Female	2.2	2.6	1.9
2000	Greece	Male	2.0	2.3	1.8
2000	Italy	Both	11.1	13.0	9.65
2000	Italy	Female	11.3	13.4	9.73
2000	Italy	Male	10.9	12.7	9.56
2000	Portugal	Both	4.8	5.6	4.1
2000	Portugal	Female	4.4	5.1	3.8
2000	Portugal	Male	5.1	6.0	4.4
2000	Spain	Both	4.9	5.2	4.5
2000	Spain	Female	4.2	4.6	3.9
2000	Spain	Male	5.5	6.0	5.0
2001	Greece	Both	2.2	2.5	1.9
2001	Greece	Female	2.3	2.7	1.9
2001	Greece	Male	2.1	2.4	1.9
2001	Italy	Both	10.8	12.7	9.45
2001	Italy	Female	11.0	13.0	9.51
2001	Italy	Male	10.6	12.4	9.38
2001	Portugal	Both	4.7	5.5	4.1
2001	Portugal	Female	4.4	5.0	3.7
2001	Portugal	Male	5.1	5.9	4.4
2001	Spain	Both	4.7	5.1	4.4
2001	Spain	Female	4.1	4.4	3.8
2001	Spain	Male	5.3	5.8	4.9
2002	Greece	Both	2.3	2.7	2.1
2002	Greece	Female	2.4	2.8	2.1
2002	Greece	Male	2.3	2.6	2.0
2002	Italy	Both	10.4	12.1	9.10
2002	Italy	Female	10.6	12.5	9.14
2002	Italy	Male	10.2	11.9	9.01
2002	Portugal	Both	4.7	5.4	4.1
2002	Portugal	Female	4.3	5.0	3.7
2002	Portugal	Male	5.1	5.9	4.4
2002	Spain	Both	4.5	4.9	4.2
2002	Spain	Female	4.0	4.3	3.7
2002	Spain	Male	5.1	5.5	4.7
2003	Greece	Both	2.5	2.9	2.2
2003	Greece	Female	2.6	3.1	2.3
2003	Greece	Male	2.4	2.8	2.1
2003	Italy	Both	9.9	11.	8.6
2003	Italy	Female	10.1	11.9	8.73
2003	Italy	Male	9.7	11.	8.5
2003	Portugal	Both	4.6	5.4	4.1
2003	Portugal	Female	4.3	5.0	3.7
2003	Portugal	Male	5.0	5.9	4.4
2003	Spain	Both	4.3	4.7	4.0

2003	Spain	Female	3.8	4.1	3.5
2003	Spain	Male	4.8	5.3	4.5
2004	Greece	Both	2.7	3.1	2.3
2004	Greece	Female	2.8	3.3	2.4
2004	Greece	Male	2.6	3.0	2.2
2004	Italy	Both	9.5	11.	8.3
2004	Italy	Female	9.6	11.	8.3
2004	Italy	Male	9.3	10.	8.1
2004	Portugal	Both	4.6	5.4	4.0
2004	Portugal	Female	4.2	5.0	3.7
2004	Portugal	Male	5.0	5.9	4.4
2004	Spain	Both	4.2	4.5	3.9
2004	Spain	Female	3.7	4.1	3.4
2004	Spain	Male	4.7	5.1	4.3
2005	Greece	Both	2.8	3.3	2.4
2005	Greece	Female	2.9	3.4	2.4
2005	Greece	Male	2.6	3.1	2.3
2005	Italy	Both	9.3	10.	8.1
2005	Italy	Female	9.4	11.	8.1
2005	Italy	Male	9.1	10.	7.9
2005	Portugal	Both	4.6	5.3	4.0
2005	Portugal	Female	4.2	4.9	3.6
2005	Portugal	Male	5.0	5.8	4.3
2005	Spain	Both	4.1	4.5	3.8
2005	Spain	Female	3.7	4.1	3.4
2005	Spain	Male	4.6	5.0	4.2
2006	Greece	Both	2.8	3.3	2.4
2006	Greece	Female	2.9	3.4	2.4
2006	Greece	Male	2.6	3.1	2.3
2006	Italy	Both	9.2	10.	8.0
2006	Italy	Female	9.3	10.	8.0
2006	Italy	Male	9.0	10.	7.9
2006	Portugal	Both	4.6	5.3	4.0
2006	Portugal	Female	4.2	4.9	3.6
2006	Portugal	Male	5.0	5.8	4.3
2006	Spain	Both	4.2	4.6	3.9
2006	Spain	Female	3.8	4.2	3.5
2006	Spain	Male	4.7	5.1	4.3
2007	Greece	Both	2.7	3.2	2.4
2007	Greece	Female	2.9	3.4	2.4
2007	Greece	Male	2.6	3.1	2.3
2007	Italy	Both	9.1	10.	7.9
2007	Italy	Female	9.1	10.	7.9
2007	Italy	Male	9.0	10.	7.8
2007	Portugal	Both	4.5	5.3	4.0
2007	Portugal	Female	4.2	4.9	3.6
2007	Portugal	Male	4.9	5.7	4.3
2007	Spain	Both	4.5	4.9	4.1
2007	Spain	Female	4.1	4.5	3.6
2007	Spain	Male	4.9	5.4	4.4
2008	Greece	Both	2.7	3.2	2.4
2008	Greece	Female	2.8	3.4	2.4
2008	Greece	Male	2.6	3.1	2.3

2008	Italy	Both	9.0	10.	7.8
2008	Italy	Female	9.0	10.	7.7
2008	Italy	Male	9.0	10.	7.8
2008	Portugal	Both	4.5	5.2	3.9
2008	Portugal	Female	4.1	4.9	3.6
2008	Portugal	Male	4.9	5.7	4.3
2008	Spain	Both	4.7	5.3	4.2
2008	Spain	Female	4.3	4.9	3.8
2008	Spain	Male	5.1	5.8	4.6
2009	Greece	Both	2.7	3.2	2.3
2009	Greece	Female	2.8	3.4	2.4
2009	Greece	Male	2.6	3.1	2.3
2009	Italy	Both	8.9	10.	7.7
2009	Italy	Female	8.9	10.	7.6
2009	Italy	Male	9.0	10.	7.8
2009	Portugal	Both	4.5	5.2	3.9
2009	Portugal	Female	4.1	4.8	3.6
2009	Portugal	Male	4.8	5.6	4.2
2009	Spain	Both	4.9	5.6	4.4
2009	Spain	Female	4.6	5.3	3.9
2009	Spain	Male	5.3	6.1	4.7
2010	Greece	Both	2.7	3.2	2.3
2010	Greece	Female	2.8	3.4	2.4
2010	Greece	Male	2.6	3.0	2.2
2010	Italy	Both	8.9	10.	7.6
2010	Italy	Female	8.8	10.	7.5
2010	Italy	Male	8.9	10.	7.7
2010	Portugal	Both	4.5	5.2	3.9
2010	Portugal	Female	4.1	4.9	3.5
2010	Portugal	Male	4.8	5.6	4.2
2010	Spain	Both	5.0	5.8	4.4
2010	Spain	Female	4.6	5.5	3.9
2010	Spain	Male	5.4	6.3	4.7
2011	Greece	Both	2.7	3.2	2.3
2011	Greece	Female	2.8	3.4	2.4
2011	Greece	Male	2.6	3.0	2.2
2011	Italy	Both	8.7	10.	7.4
2011	Italy	Female	8.6	10.	7.3
2011	Italy	Male	8.7	10.	7.6
2011	Portugal	Both	4.4	5.2	3.9
2011	Portugal	Female	4.1	4.8	3.5
2011	Portugal	Male	4.8	5.5	4.2
2011	Spain	Both	5.0	5.7	4.4
2011	Spain	Female	4.6	5.5	3.9
2011	Spain	Male	5.4	6.2	4.7
2012	Greece	Both	2.7	3.2	2.3
2012	Greece	Female	2.8	3.4	2.4
2012	Greece	Male	2.6	3.0	2.2
2012	Italy	Both	8.4	9.9	7.2
2012	Italy	Female	8.3	9.9	7.1
2012	Italy	Male	8.4	9.9	7.3
2012	Portugal	Both	4.4	5.1	3.8
2012	Portugal	Female	4.1	4.8	3.5

2012	Portugal	Male	4.7	5.5	4.1
2012	Spain	Both	4.9	5.7	4.3
2012	Spain	Female	4.6	5.4	3.9
2012	Spain	Male	5.3	6.1	4.6
2013	Greece	Both	2.7	3.2	2.3
2013	Greece	Female	2.8	3.4	2.4
2013	Greece	Male	2.5	3.0	2.2
2013	Italy	Both	8.0	9.5	6.9
2013	Italy	Female	8.0	9.6	6.8
2013	Italy	Male	8.0	9.4	6.9
2013	Portugal	Both	4.4	5.1	3.8
2013	Portugal	Female	4.1	4.8	3.5
2013	Portugal	Male	4.7	5.4	4.1
2013	Spain	Both	4.8	5.6	4.3
2013	Spain	Female	4.5	5.3	3.9
2013	Spain	Male	5.2	5.9	4.6
2014	Greece	Both	2.7	3.2	2.3
2014	Greece	Female	2.8	3.4	2.4
2014	Greece	Male	2.5	3.0	2.2
2014	Italy	Both	7.7	9.1	6.6
2014	Italy	Female	7.8	9.3	6.6
2014	Italy	Male	7.7	9.0	6.6
2014	Portugal	Both	4.3	5.1	3.8
2014	Portugal	Female	4.1	4.9	3.5
2014	Portugal	Male	4.6	5.3	4.0
2014	Spain	Both	4.8	5.5	4.2
2014	Spain	Female	4.5	5.3	3.8
2014	Spain	Male	5.1	5.8	4.5
2015	Greece	Both	2.7	3.2	2.3
2015	Greece	Female	2.8	3.4	2.4
2015	Greece	Male	2.5	3.0	2.1
2015	Italy	Both	7.6	9.0	6.5
2015	Italy	Female	7.7	9.1	6.5
2015	Italy	Male	7.5	8.8	6.5
2015	Portugal	Both	4.3	5.1	3.8
2015	Portugal	Female	4.1	4.9	3.4
2015	Portugal	Male	4.6	5.3	4.0
2015	Spain	Both	4.7	5.5	4.1
2015	Spain	Female	4.4	5.4	3.8
2015	Spain	Male	5.0	5.8	4.4
2016	Greece	Both	2.7	3.2	2.3
2016	Greece	Female	2.8	3.3	2.4
2016	Greece	Male	2.6	3.1	2.2
2016	Italy	Both	7.7	9.1	6.6
2016	Italy	Female	7.7	9.2	6.6
2016	Italy	Male	7.6	9.0	6.6
2016	Portugal	Both	4.3	5.0	3.8
2016	Portugal	Female	4.0	4.7	3.5
2016	Portugal	Male	4.6	5.4	4.0
2016	Spain	Both	4.7	5.5	4.2
2016	Spain	Female	4.4	5.2	3.8
2016	Spain	Male	5.0	5.7	4.4
2017	Greece	Both	2.7	3.2	2.3

2017	Greece	Female	2.8	3.3	2.4
2017	Greece	Male	2.6	3.1	2.2
2017	Italy	Both	7.8	9.2	6.7
2017	Italy	Female	7.8	9.3	6.6
2017	Italy	Male	7.8	9.2	6.7
2017	Portugal	Both	4.3	5.1	3.8
2017	Portugal	Female	4.0	4.7	3.5
2017	Portugal	Male	4.7	5.5	4.1
2017	Spain	Both	4.7	5.4	4.2
2017	Spain	Female	4.4	5.2	3.8
2017	Spain	Male	5.0	5.8	4.3
2018	Greece	Both	2.7	3.2	2.3
2018	Greece	Female	2.8	3.3	2.4
2018	Greece	Male	2.6	3.1	2.3
2018	Italy	Both	7.8	9.2	6.7
2018	Italy	Female	7.8	9.4	6.6
2018	Italy	Male	7.8	9.2	6.7
2018	Portugal	Both	4.3	5.0	3.8
2018	Portugal	Female	4.0	4.7	3.5
2018	Portugal	Male	4.7	5.4	4.1
2018	Spain	Both	4.7	5.4	4.2
2018	Spain	Female	4.4	5.1	3.8
2018	Spain	Male	5.0	5.8	4.4
2019	Greece	Both	2.7	3.2	2.3
2019	Greece	Female	2.8	3.4	2.4
2019	Greece	Male	2.6	3.1	2.2
2019	Italy	Both	7.8	9.2	6.6
2019	Italy	Female	7.8	9.4	6.6
2019	Italy	Male	7.7	9.1	6.6
2019	Portugal	Both	4.3	5.0	3.8
2019	Portugal	Female	4.0	4.7	3.5
2019	Portugal	Male	4.6	5.3	4.0
2019	Spain	Both	4.7	5.4	4.1
2019	Spain	Female	4.4	5.2	3.8
2019	Spain	Male	5.0	5.8	4.4

Table S8- Age-standardized rates and 95% uncertainty levels (UL) of **acute HCV incidence**, per 100,000 population in Greece, Italy, Portugal and Spain from 2000 to 2019 by sex classes (Global Burden of Disease Study 2019).

Year	Country	Sex	Incidence (95% UL)	95% UL (upper)	95% UL (lower)
2000	Greece	Both	18.8	21.5	16.4
2000	Greece	Female	19.3	22.8	16.5
2000	Greece	Male	18.1	20.5	16.2
2000	Italy	Both	96.5	112.6	83.7
2000	Italy	Female	97.9	116.1	84.4
2000	Italy	Male	94.7	110.1	82.9
2000	Portugal	Both	41.6	48.5	36.1
2000	Portugal	Female	38.4	44.6	32.9
2000	Portugal	Male	44.9	52.3	38.9
2000	Spain	Both	42.4	45.8	39.4
2000	Spain	Female	37.1	40.1	33.9
2000	Spain	Male	48.2	52.6	44.1
2001	Greece	Both	19.3	22.2	17.0
2001	Greece	Female	20.0	23.4	17.2
2001	Greece	Male	18.6	21.2	16.6
2001	Italy	Both	94.3	110.1	81.9
2001	Italy	Female	95.6	113.0	82.4
2001	Italy	Male	92.7	107.6	81.2
2001	Portugal	Both	41.3	47.9	36.1
2001	Portugal	Female	38.1	43.9	32.9
2001	Portugal	Male	44.6	51.7	38.8
2001	Spain	Both	41.2	44.4	38.4
2001	Spain	Female	36.0	38.9	33.1
2001	Spain	Male	46.7	50.7	43.1
2002	Greece	Both	20.7	23.8	18.2
2002	Greece	Female	21.4	25.0	18.6
2002	Greece	Male	19.9	22.6	17.6
2002	Italy	Both	90.6	105.5	78.9
2002	Italy	Female	91.8	108.3	79.2
2002	Italy	Male	88.9	103.2	78.1
2002	Portugal	Both	41.0	47.3	36.0
2002	Portugal	Female	37.8	43.4	32.7
2002	Portugal	Male	44.3	51.3	38.6
2002	Spain	Both	39.5	42.5	36.8
2002	Spain	Female	34.8	37.4	32.1
2002	Spain	Male	44.6	48.3	41.3
2003	Greece	Both	22.3	25.9	19.6
2003	Greece	Female	23.1	27.1	20.2
2003	Greece	Male	21.4	24.6	18.9
2003	Italy	Both	86.3	100.7	75.2
2003	Italy	Female	87.6	103.3	75.6
2003	Italy	Male	84.6	98.3	74.1
2003	Portugal	Both	40.7	47.1	35.8
2003	Portugal	Female	37.5	43.4	32.4
2003	Portugal	Male	44.1	51.2	38.5
2003	Spain	Both	37.9	40.9	35.3
2003	Spain	Female	33.6	36.3	31.0

2003	Spain	Male	42.4	45.9	39.3
2004	Greece	Both	23.7	27.6	20.7
2004	Greece	Female	24.6	29.0	21.2
2004	Greece	Male	22.7	26.4	19.9
2004	Italy	Both	82.6	96.4	71.9
2004	Italy	Female	83.9	99.0	72.5
2004	Italy	Male	80.9	94.0	70.7
2004	Portugal	Both	40.4	46.9	35.4
2004	Portugal	Female	37.2	43.4	32.1
2004	Portugal	Male	43.8	51.2	38.3
2004	Spain	Both	36.7	39.7	34.1
2004	Spain	Female	32.8	35.6	30.1
2004	Spain	Male	40.7	44.3	37.7
2005	Greece	Both	24.3	28.6	21.0
2005	Greece	Female	25.2	29.9	21.3
2005	Greece	Male	23.2	27.6	20.1
2005	Italy	Both	80.6	94.3	70.2
2005	Italy	Female	81.7	96.3	70.6
2005	Italy	Male	79.1	91.9	69.1
2005	Portugal	Both	40.2	46.7	35.2
2005	Portugal	Female	36.9	43.2	31.9
2005	Portugal	Male	43.6	51.1	38.0
2005	Spain	Both	36.3	39.4	33.5
2005	Spain	Female	32.6	35.6	29.7
2005	Spain	Male	40.1	44.0	37.0
2006	Greece	Both	24.3	28.6	20.9
2006	Greece	Female	25.2	29.8	21.4
2006	Greece	Male	23.2	27.4	20.1
2006	Italy	Both	79.8	93.4	69.4
2006	Italy	Female	80.7	95.2	69.6
2006	Italy	Male	78.6	91.6	68.6
2006	Portugal	Both	39.9	46.4	35.1
2006	Portugal	Female	36.7	43.0	31.8
2006	Portugal	Male	43.3	50.5	38.0
2006	Spain	Both	37.1	40.6	34.2
2006	Spain	Female	33.6	36.8	30.4
2006	Spain	Male	40.8	44.8	37.5
2007	Greece	Both	24.2	28.5	20.8
2007	Greece	Female	25.1	29.8	21.4
2007	Greece	Male	23.2	27.2	20.1
2007	Italy	Both	79.2	92.9	68.5
2007	Italy	Female	79.6	93.9	68.5
2007	Italy	Male	78.5	91.8	68.3
2007	Portugal	Both	39.7	46.0	34.8
2007	Portugal	Female	36.5	42.4	31.7
2007	Portugal	Male	43.0	49.9	37.8
2007	Spain	Both	39.0	43.2	35.5
2007	Spain	Female	35.6	39.6	31.7
2007	Spain	Male	42.5	47.3	38.7
2008	Greece	Both	24.1	28.5	20.8
2008	Greece	Female	25.0	29.8	21.4
2008	Greece	Male	23.1	26.9	20.0
2008	Italy	Both	78.5	92.5	67.8

2008	Italy	Female	78.5	92.9	67.3
2008	Italy	Male	78.4	92.0	68.0
2008	Portugal	Both	39.4	45.7	34.5
2008	Portugal	Female	36.3	42.5	31.4
2008	Portugal	Male	42.7	49.4	37.4
2008	Spain	Both	41.2	46.2	37.0
2008	Spain	Female	37.9	43.0	33.2
2008	Spain	Male	44.6	50.3	39.9
2009	Greece	Both	24.0	28.3	20.7
2009	Greece	Female	24.9	29.9	21.2
2009	Greece	Male	23.0	26.8	20.0
2009	Italy	Both	77.8	91.8	67.0
2009	Italy	Female	77.4	92.0	66.2
2009	Italy	Male	78.2	92.0	67.8
2009	Portugal	Both	39.2	45.4	34.2
2009	Portugal	Female	36.2	42.3	31.2
2009	Portugal	Male	42.4	49.2	37.1
2009	Spain	Both	43.0	49.2	38.2
2009	Spain	Female	39.9	46.1	34.3
2009	Spain	Male	46.3	53.2	40.8
2010	Greece	Both	24.0	28.2	20.6
2010	Greece	Female	24.8	30.0	21.1
2010	Greece	Male	23.0	26.8	19.8
2010	Italy	Both	77.1	91.2	66.2
2010	Italy	Female	76.5	91.1	65.0
2010	Italy	Male	77.8	91.6	67.2
2010	Portugal	Both	39.0	45.4	33.9
2010	Portugal	Female	36.0	42.5	30.6
2010	Portugal	Male	42.1	49.2	36.6
2010	Spain	Both	43.8	50.7	38.3
2010	Spain	Female	40.7	47.9	34.2
2010	Spain	Male	47.0	54.9	40.7
2011	Greece	Both	23.9	28.1	20.6
2011	Greece	Female	24.8	29.7	21.2
2011	Greece	Male	22.8	26.5	19.7
2011	Italy	Both	75.5	89.3	64.9
2011	Italy	Female	75.0	89.3	63.7
2011	Italy	Male	76.1	89.6	65.9
2011	Portugal	Both	38.7	45.1	33.8
2011	Portugal	Female	35.9	42.3	30.6
2011	Portugal	Male	41.7	48.4	36.6
2011	Spain	Both	43.6	50.2	38.2
2011	Spain	Female	40.5	47.7	34.5
2011	Spain	Male	46.8	54.1	40.9
2012	Greece	Both	23.8	28.1	20.6
2012	Greece	Female	24.8	29.7	21.3
2012	Greece	Male	22.7	26.4	19.5
2012	Italy	Both	72.8	86.1	62.5
2012	Italy	Female	72.5	86.4	61.7
2012	Italy	Male	73.1	85.8	63.2
2012	Portugal	Both	38.5	44.8	33.5
2012	Portugal	Female	35.8	42.3	30.6
2012	Portugal	Male	41.3	47.7	36.3

2012	Spain	Both	43.1	49.5	37.8
2012	Spain	Female	40.1	47.1	34.2
2012	Spain	Male	46.0	53.1	40.5
2013	Greece	Both	23.7	28.0	20.6
2013	Greece	Female	24.8	29.6	21.3
2013	Greece	Male	22.5	26.3	19.4
2013	Italy	Both	69.7	82.5	59.9
2013	Italy	Female	69.9	83.2	59.6
2013	Italy	Male	69.6	81.7	60.3
2013	Portugal	Both	38.2	44.7	33.4
2013	Portugal	Female	35.7	42.3	30.5
2013	Portugal	Male	40.8	47.1	35.7
2013	Spain	Both	42.4	48.8	37.3
2013	Spain	Female	39.6	46.5	34.1
2013	Spain	Male	45.1	51.8	39.9
2014	Greece	Both	23.6	27.9	20.5
2014	Greece	Female	24.8	29.6	21.3
2014	Greece	Male	22.3	26.4	19.1
2014	Italy	Both	67.2	79.5	57.7
2014	Italy	Female	67.7	80.6	57.7
2014	Italy	Male	66.8	78.4	57.8
2014	Portugal	Both	38.0	44.4	33.1
2014	Portugal	Female	35.6	42.6	30.3
2014	Portugal	Male	40.4	46.7	35.2
2014	Spain	Both	41.7	48.3	36.6
2014	Spain	Female	39.2	46.5	33.7
2014	Spain	Male	44.3	50.9	39.0
2015	Greece	Both	23.6	27.9	20.4
2015	Greece	Female	24.8	29.7	21.2
2015	Greece	Male	22.2	26.5	19.0
2015	Italy	Both	66.1	78.3	56.8
2015	Italy	Female	66.7	79.3	56.9
2015	Italy	Male	65.6	77.1	56.9
2015	Portugal	Both	37.9	44.4	32.9
2015	Portugal	Female	35.6	42.8	30.1
2015	Portugal	Male	40.3	46.7	34.7
2015	Spain	Both	41.4	48.1	36.1
2015	Spain	Female	38.9	46.9	33.2
2015	Spain	Male	43.9	50.4	38.6
2016	Greece	Both	23.8	28.1	20.5
2016	Greece	Female	24.8	29.2	21.1
2016	Greece	Male	22.7	26.9	19.6
2016	Italy	Both	66.9	78.9	57.4
2016	Italy	Female	67.2	80.1	57.4
2016	Italy	Male	66.6	78.0	57.5
2016	Portugal	Both	37.9	43.9	33.3
2016	Portugal	Female	35.4	41.5	30.6
2016	Portugal	Male	40.6	46.8	35.4
2016	Spain	Both	41.3	47.8	36.4
2016	Spain	Female	38.8	45.9	33.7
2016	Spain	Male	43.9	49.9	38.8
2017	Greece	Both	24.0	28.2	20.6
2017	Greece	Female	24.8	29.1	20.9

2017	Greece	Male	23.2	27.4	19.8
2017	Italy	Both	67.7	80.3	58.0
2017	Italy	Female	67.8	81.3	57.8
2017	Italy	Male	67.7	79.8	58.3
2017	Portugal	Both	38.0	44.6	33.3
2017	Portugal	Female	35.3	41.3	30.5
2017	Portugal	Male	41.0	48.0	35.6
2017	Spain	Both	41.3	47.6	36.5
2017	Spain	Female	38.7	45.9	33.2
2017	Spain	Male	44.1	50.9	38.0
2018	Greece	Both	24.1	28.1	20.6
2018	Greece	Female	24.9	29.2	21.0
2018	Greece	Male	23.1	27.3	19.9
2018	Italy	Both	67.8	80.5	58.1
2018	Italy	Female	68.0	81.6	57.9
2018	Italy	Male	67.7	79.9	58.3
2018	Portugal	Both	38.0	43.7	33.4
2018	Portugal	Female	35.3	41.1	30.7
2018	Portugal	Male	40.9	47.6	36.0
2018	Spain	Both	41.3	47.4	36.4
2018	Spain	Female	38.6	44.9	33.7
2018	Spain	Male	44.1	50.2	38.7
2019	Greece	Both	24.0	28.1	20.6
2019	Greece	Female	25.0	29.7	21.1
2019	Greece	Male	22.9	26.8	19.8
2019	Italy	Both	67.7	80.4	57.9
2019	Italy	Female	68.2	82.1	58.0
2019	Italy	Male	67.3	79.3	57.9
2019	Portugal	Both	37.8	43.5	33.3
2019	Portugal	Female	35.2	41.5	30.5
2019	Portugal	Male	40.6	46.7	35.5
2019	Spain	Both	41.2	47.3	35.9
2019	Spain	Female	38.6	45.2	33.1
2019	Spain	Male	44.0	50.6	38.3

Table S9- Age-standardized rates and 95% uncertainty levels (UL) of **acute HCV deaths** per 100,000 population in Greece, Italy, Portugal and Spain from 2000 to 2019 by sex classes (Global Burden of Disease Study 2019).

Year	Country	Sex	Deaths (95% UL)	95% UL (upper)	95% UL (lower)
2000	Greece	Both	0.00	0.02	0.00
2000	Greece	Female	0.00	0.02	0.00
2000	Greece	Male	0.00	0.02	0.00
2000	Italy	Both	0.01	0.02	0.00
2000	Italy	Female	0.01	0.01	0.00
2000	Italy	Male	0.02	0.03	0.00
2000	Portugal	Both	0.02	0.04	0.01
2000	Portugal	Female	0.01	0.02	0.00
2000	Portugal	Male	0.03	0.07	0.01
2000	Spain	Both	0.01	0.01	0.00
2000	Spain	Female	0.01	0.01	0.00
2000	Spain	Male	0.01	0.02	0.00
2001	Greece	Both	0.00	0.01	0.00
2001	Greece	Female	0.00	0.02	0.00
2001	Greece	Male	0.00	0.02	0.00
2001	Italy	Both	0.01	0.02	0.00
2001	Italy	Female	0.01	0.01	0.00
2001	Italy	Male	0.01	0.02	0.00
2001	Portugal	Both	0.01	0.02	0.00
2001	Portugal	Female	0.01	0.01	0.00
2001	Portugal	Male	0.02	0.04	0.00
2001	Spain	Both	0.01	0.01	0.00
2001	Spain	Female	0.01	0.01	0.00
2001	Spain	Male	0.01	0.02	0.00
2002	Greece	Both	0.00	0.02	0.00
2002	Greece	Female	0.00	0.02	0.00
2002	Greece	Male	0.00	0.02	0.00
2002	Italy	Both	0.01	0.02	0.00
2002	Italy	Female	0.01	0.01	0.00
2002	Italy	Male	0.01	0.02	0.00
2002	Portugal	Both	0.01	0.02	0.00
2002	Portugal	Female	0.00	0.01	0.00
2002	Portugal	Male	0.02	0.03	0.00
2002	Spain	Both	0.01	0.01	0.00
2002	Spain	Female	0.01	0.01	0.00
2002	Spain	Male	0.01	0.02	0.00
2003	Greece	Both	0.00	0.02	0.00
2003	Greece	Female	0.00	0.02	0.00
2003	Greece	Male	0.00	0.02	0.00
2003	Italy	Both	0.01	0.02	0.00
2003	Italy	Female	0.01	0.01	0.00
2003	Italy	Male	0.02	0.03	0.00
2003	Portugal	Both	0.01	0.01	0.00

2003	Portugal	Female	0.00	0.01	0.00
2003	Portugal	Male	0.01	0.02	0.00
2003	Spain	Both	0.01	0.01	0.00
2003	Spain	Female	0.01	0.01	0.00
2003	Spain	Male	0.01	0.02	0.00
2004	Greece	Both	0.00	0.02	0.00
2004	Greece	Female	0.00	0.02	0.00
2004	Greece	Male	0.00	0.02	0.00
2004	Italy	Both	0.01	0.02	0.00
2004	Italy	Female	0.01	0.01	0.00
2004	Italy	Male	0.01	0.03	0.00
2004	Portugal	Both	0.01	0.01	0.00
2004	Portugal	Female	0.00	0.01	0.00
2004	Portugal	Male	0.01	0.02	0.00
2004	Spain	Both	0.01	0.01	0.00
2004	Spain	Female	0.01	0.01	0.00
2004	Spain	Male	0.01	0.02	0.00
2005	Greece	Both	0.01	0.03	0.00
2005	Greece	Female	0.01	0.03	0.00
2005	Greece	Male	0.00	0.03	0.00
2005	Italy	Both	0.01	0.02	0.00
2005	Italy	Female	0.01	0.01	0.00
2005	Italy	Male	0.01	0.03	0.00
2005	Portugal	Both	0.01	0.01	0.00
2005	Portugal	Female	0.00	0.00	0.00
2005	Portugal	Male	0.01	0.02	0.00
2005	Spain	Both	0.01	0.01	0.00
2005	Spain	Female	0.01	0.01	0.00
2005	Spain	Male	0.01	0.02	0.00
2006	Greece	Both	0.01	0.03	0.00
2006	Greece	Female	0.01	0.05	0.00
2006	Greece	Male	0.01	0.03	0.00
2006	Italy	Both	0.01	0.01	0.00
2006	Italy	Female	0.00	0.01	0.00
2006	Italy	Male	0.01	0.03	0.00
2006	Portugal	Both	0.00	0.01	0.00
2006	Portugal	Female	0.00	0.00	0.00
2006	Portugal	Male	0.01	0.01	0.00
2006	Spain	Both	0.01	0.01	0.00
2006	Spain	Female	0.01	0.01	0.00
2006	Spain	Male	0.01	0.02	0.00
2007	Greece	Both	0.01	0.05	0.00
2007	Greece	Female	0.02	0.07	0.00
2007	Greece	Male	0.01	0.05	0.00
2007	Italy	Both	0.01	0.01	0.00
2007	Italy	Female	0.00	0.01	0.00
2007	Italy	Male	0.01	0.02	0.00
2007	Portugal	Both	0.00	0.01	0.00
2007	Portugal	Female	0.00	0.00	0.00
2007	Portugal	Male	0.01	0.01	0.00

2007	Spain	Both	0.01	0.01	0.00
2007	Spain	Female	0.00	0.01	0.00
2007	Spain	Male	0.01	0.01	0.00
2008	Greece	Both	0.01	0.05	0.00
2008	Greece	Female	0.02	0.07	0.00
2008	Greece	Male	0.01	0.04	0.00
2008	Italy	Both	0.00	0.01	0.00
2008	Italy	Female	0.00	0.01	0.00
2008	Italy	Male	0.01	0.02	0.00
2008	Portugal	Both	0.00	0.01	0.00
2008	Portugal	Female	0.00	0.00	0.00
2008	Portugal	Male	0.01	0.01	0.00
2008	Spain	Both	0.01	0.01	0.00
2008	Spain	Female	0.00	0.01	0.00
2008	Spain	Male	0.01	0.01	0.00
2009	Greece	Both	0.02	0.06	0.00
2009	Greece	Female	0.02	0.08	0.01
2009	Greece	Male	0.01	0.05	0.00
2009	Italy	Both	0.00	0.01	0.00
2009	Italy	Female	0.00	0.01	0.00
2009	Italy	Male	0.00	0.02	0.00
2009	Portugal	Both	0.00	0.00	0.00
2009	Portugal	Female	0.00	0.00	0.00
2009	Portugal	Male	0.00	0.01	0.00
2009	Spain	Both	0.01	0.01	0.00
2009	Spain	Female	0.00	0.01	0.00
2009	Spain	Male	0.01	0.01	0.00
2010	Greece	Both	0.02	0.06	0.01
2010	Greece	Female	0.03	0.09	0.01
2010	Greece	Male	0.01	0.05	0.00
2010	Italy	Both	0.00	0.01	0.00
2010	Italy	Female	0.00	0.01	0.00
2010	Italy	Male	0.00	0.02	0.00
2010	Portugal	Both	0.00	0.00	0.00
2010	Portugal	Female	0.00	0.00	0.00
2010	Portugal	Male	0.00	0.01	0.00
2010	Spain	Both	0.00	0.01	0.00
2010	Spain	Female	0.00	0.01	0.00
2010	Spain	Male	0.01	0.01	0.00
2011	Greece	Both	0.02	0.06	0.01
2011	Greece	Female	0.03	0.09	0.01
2011	Greece	Male	0.01	0.05	0.00
2011	Italy	Both	0.00	0.01	0.00
2011	Italy	Female	0.00	0.01	0.00
2011	Italy	Male	0.00	0.02	0.00
2011	Portugal	Both	0.00	0.00	0.00
2011	Portugal	Female	0.00	0.00	0.00
2011	Portugal	Male	0.00	0.01	0.00
2011	Spain	Both	0.00	0.01	0.00
2011	Spain	Female	0.00	0.00	0.00

2011	Spain	Male	0.00	0.01	0.00
2012	Greece	Both	0.02	0.06	0.01
2012	Greece	Female	0.03	0.08	0.01
2012	Greece	Male	0.01	0.05	0.00
2012	Italy	Both	0.00	0.01	0.00
2012	Italy	Female	0.00	0.01	0.00
2012	Italy	Male	0.00	0.02	0.00
2012	Portugal	Both	0.00	0.00	0.00
2012	Portugal	Female	0.00	0.00	0.00
2012	Portugal	Male	0.00	0.00	0.00
2012	Spain	Both	0.00	0.01	0.00
2012	Spain	Female	0.00	0.00	0.00
2012	Spain	Male	0.00	0.01	0.00
2013	Greece	Both	0.02	0.05	0.01
2013	Greece	Female	0.02	0.06	0.01
2013	Greece	Male	0.01	0.04	0.00
2013	Italy	Both	0.00	0.01	0.00
2013	Italy	Female	0.00	0.01	0.00
2013	Italy	Male	0.00	0.02	0.00
2013	Portugal	Both	0.00	0.00	0.00
2013	Portugal	Female	0.00	0.00	0.00
2013	Portugal	Male	0.00	0.00	0.00
2013	Spain	Both	0.00	0.01	0.00
2013	Spain	Female	0.00	0.00	0.00
2013	Spain	Male	0.00	0.01	0.00
2014	Greece	Both	0.01	0.03	0.00
2014	Greece	Female	0.01	0.04	0.00
2014	Greece	Male	0.01	0.02	0.00
2014	Italy	Both	0.00	0.01	0.00
2014	Italy	Female	0.00	0.01	0.00
2014	Italy	Male	0.00	0.01	0.00
2014	Portugal	Both	0.00	0.00	0.00
2014	Portugal	Female	0.00	0.00	0.00
2014	Portugal	Male	0.00	0.00	0.00
2014	Spain	Both	0.00	0.01	0.00
2014	Spain	Female	0.00	0.00	0.00
2014	Spain	Male	0.00	0.01	0.00
2015	Greece	Both	0.01	0.02	0.00
2015	Greece	Female	0.01	0.02	0.00
2015	Greece	Male	0.00	0.02	0.00
2015	Italy	Both	0.00	0.01	0.00
2015	Italy	Female	0.00	0.01	0.00
2015	Italy	Male	0.00	0.01	0.00
2015	Portugal	Both	0.00	0.00	0.00
2015	Portugal	Female	0.00	0.00	0.00
2015	Portugal	Male	0.00	0.00	0.00
2015	Spain	Both	0.00	0.00	0.00
2015	Spain	Female	0.00	0.00	0.00
2015	Spain	Male	0.00	0.01	0.00
2016	Greece	Both	0.00	0.02	0.00

2016	Greece	Female	0.01	0.02	0.00
2016	Greece	Male	0.00	0.02	0.00
2016	Italy	Both	0.00	0.01	0.00
2016	Italy	Female	0.00	0.00	0.00
2016	Italy	Male	0.00	0.01	0.00
2016	Portugal	Both	0.00	0.00	0.00
2016	Portugal	Female	0.00	0.00	0.00
2016	Portugal	Male	0.00	0.00	0.00
2016	Spain	Both	0.00	0.00	0.00
2016	Spain	Female	0.00	0.00	0.00
2016	Spain	Male	0.00	0.01	0.00
2017	Greece	Both	0.01	0.02	0.00
2017	Greece	Female	0.01	0.02	0.00
2017	Greece	Male	0.00	0.02	0.00
2017	Italy	Both	0.00	0.01	0.00
2017	Italy	Female	0.00	0.01	0.00
2017	Italy	Male	0.00	0.01	0.00
2017	Portugal	Both	0.00	0.00	0.00
2017	Portugal	Female	0.00	0.00	0.00
2017	Portugal	Male	0.00	0.00	0.00
2017	Spain	Both	0.00	0.00	0.00
2017	Spain	Female	0.00	0.00	0.00
2017	Spain	Male	0.00	0.01	0.00
2018	Greece	Both	0.01	0.02	0.00
2018	Greece	Female	0.01	0.02	0.00
2018	Greece	Male	0.00	0.02	0.00
2018	Italy	Both	0.00	0.01	0.00
2018	Italy	Female	0.00	0.01	0.00
2018	Italy	Male	0.00	0.01	0.00
2018	Portugal	Both	0.00	0.00	0.00
2018	Portugal	Female	0.00	0.00	0.00
2018	Portugal	Male	0.00	0.00	0.00
2018	Spain	Both	0.00	0.00	0.00
2018	Spain	Female	0.00	0.00	0.00
2018	Spain	Male	0.00	0.01	0.00
2019	Greece	Both	0.01	0.02	0.00
2019	Greece	Female	0.01	0.02	0.00
2019	Greece	Male	0.00	0.02	0.00
2019	Italy	Both	0.00	0.01	0.00
2019	Italy	Female	0.00	0.01	0.00
2019	Italy	Male	0.00	0.01	0.00
2019	Portugal	Both	0.00	0.00	0.00
2019	Portugal	Female	0.00	0.00	0.00
2019	Portugal	Male	0.00	0.00	0.00
2019	Spain	Both	0.00	0.00	0.00
2019	Spain	Female	0.00	0.00	0.00
2019	Spain	Male	0.00	0.01	0.00

Table S10- Age-standardized rates and 95% uncertainty levels (UL) of **acute HCV years of life lost** (YLLs) per 100,000 population in Greece, Italy, Portugal and Spain from 2000 to 2019 by sex classes (Global Burden of Disease Study 2019).

Year	Country	Sex	YLLs (95% UL)	95% UL (upper)	95% UL (lower)
2000	Greece	Both	0.20	0.48	0.10
2000	Greece	Female	0.24	0.55	0.11
2000	Greece	Male	0.17	0.57	0.06
2000	Italy	Both	0.44	0.63	0.23
2000	Italy	Female	0.30	0.54	0.14
2000	Italy	Male	0.58	0.93	0.18
2000	Portugal	Both	0.92	2.02	0.51
2000	Portugal	Female	0.42	1.03	0.21
2000	Portugal	Male	1.44	3.50	0.63
2000	Spain	Both	0.56	0.76	0.35
2000	Spain	Female	0.45	0.72	0.30
2000	Spain	Male	0.67	0.96	0.30
2001	Greece	Both	0.20	0.45	0.10
2001	Greece	Female	0.23	0.51	0.11
2001	Greece	Male	0.16	0.54	0.06
2001	Italy	Both	0.42	0.59	0.21
2001	Italy	Female	0.29	0.54	0.13
2001	Italy	Male	0.55	0.88	0.18
2001	Portugal	Both	0.71	1.13	0.33
2001	Portugal	Female	0.37	0.63	0.14
2001	Portugal	Male	1.06	1.83	0.34
2001	Spain	Both	0.53	0.73	0.33
2001	Spain	Female	0.42	0.68	0.27
2001	Spain	Male	0.64	0.92	0.30
2002	Greece	Both	0.21	0.48	0.11
2002	Greece	Female	0.25	0.56	0.12
2002	Greece	Male	0.17	0.55	0.06
2002	Italy	Both	0.41	0.59	0.21
2002	Italy	Female	0.28	0.52	0.12
2002	Italy	Male	0.54	0.86	0.17
2002	Portugal	Both	0.64	0.92	0.26
2002	Portugal	Female	0.34	0.52	0.11
2002	Portugal	Male	0.96	1.45	0.25
2002	Spain	Both	0.50	0.72	0.31
2002	Spain	Female	0.39	0.73	0.25
2002	Spain	Male	0.62	0.89	0.27
2003	Greece	Both	0.22	0.50	0.11
2003	Greece	Female	0.26	0.57	0.12
2003	Greece	Male	0.18	0.60	0.06
2003	Italy	Both	0.42	0.66	0.21
2003	Italy	Female	0.29	0.59	0.11
2003	Italy	Male	0.56	0.95	0.17
2003	Portugal	Both	0.54	0.74	0.21
2003	Portugal	Female	0.28	0.43	0.10

2003	Portugal	Male	0.81	1.16	0.20
2003	Spain	Both	0.49	0.69	0.29
2003	Spain	Female	0.38	0.71	0.24
2003	Spain	Male	0.61	0.85	0.25
2004	Greece	Both	0.24	0.58	0.12
2004	Greece	Female	0.28	0.62	0.13
2004	Greece	Male	0.21	0.73	0.07
2004	Italy	Both	0.39	0.64	0.20
2004	Italy	Female	0.27	0.61	0.10
2004	Italy	Male	0.52	0.93	0.17
2004	Portugal	Both	0.45	0.62	0.19
2004	Portugal	Female	0.23	0.36	0.08
2004	Portugal	Male	0.67	0.99	0.20
2004	Spain	Both	0.47	0.63	0.27
2004	Spain	Female	0.37	0.63	0.22
2004	Spain	Male	0.57	0.79	0.24
2005	Greece	Both	0.29	0.71	0.15
2005	Greece	Female	0.35	0.83	0.17
2005	Greece	Male	0.24	0.84	0.07
2005	Italy	Both	0.36	0.63	0.18
2005	Italy	Female	0.25	0.60	0.09
2005	Italy	Male	0.47	0.90	0.16
2005	Portugal	Both	0.41	0.56	0.14
2005	Portugal	Female	0.22	0.30	0.06
2005	Portugal	Male	0.62	0.88	0.14
2005	Spain	Both	0.45	0.58	0.26
2005	Spain	Female	0.36	0.57	0.21
2005	Spain	Male	0.55	0.73	0.23
2006	Greece	Both	0.36	0.87	0.17
2006	Greece	Female	0.43	1.02	0.21
2006	Greece	Male	0.28	1.00	0.08
2006	Italy	Both	0.32	0.63	0.17
2006	Italy	Female	0.22	0.61	0.09
2006	Italy	Male	0.42	0.91	0.16
2006	Portugal	Both	0.35	0.48	0.11
2006	Portugal	Female	0.20	0.28	0.05
2006	Portugal	Male	0.50	0.72	0.12
2006	Spain	Both	0.43	0.56	0.25
2006	Spain	Female	0.35	0.53	0.20
2006	Spain	Male	0.52	0.71	0.22
2007	Greece	Both	0.46	1.14	0.23
2007	Greece	Female	0.56	1.37	0.28
2007	Greece	Male	0.36	1.30	0.11
2007	Italy	Both	0.28	0.62	0.15
2007	Italy	Female	0.19	0.59	0.08
2007	Italy	Male	0.37	0.89	0.15
2007	Portugal	Both	0.34	0.47	0.10
2007	Portugal	Female	0.20	0.28	0.05
2007	Portugal	Male	0.49	0.71	0.11
2007	Spain	Both	0.41	0.53	0.23

2007	Spain	Female	0.33	0.51	0.19
2007	Spain	Male	0.49	0.67	0.21
2008	Greece	Both	0.45	1.10	0.22
2008	Greece	Female	0.55	1.32	0.27
2008	Greece	Male	0.35	1.26	0.10
2008	Italy	Both	0.22	0.59	0.13
2008	Italy	Female	0.15	0.56	0.07
2008	Italy	Male	0.30	0.84	0.14
2008	Portugal	Both	0.30	0.42	0.09
2008	Portugal	Female	0.19	0.26	0.04
2008	Portugal	Male	0.43	0.63	0.10
2008	Spain	Both	0.38	0.49	0.22
2008	Spain	Female	0.31	0.49	0.18
2008	Spain	Male	0.45	0.62	0.20
2009	Greece	Both	0.58	1.37	0.30
2009	Greece	Female	0.73	1.74	0.38
2009	Greece	Male	0.43	1.49	0.14
2009	Italy	Both	0.18	0.54	0.11
2009	Italy	Female	0.13	0.53	0.06
2009	Italy	Male	0.24	0.77	0.13
2009	Portugal	Both	0.26	0.36	0.09
2009	Portugal	Female	0.17	0.24	0.04
2009	Portugal	Male	0.36	0.53	0.09
2009	Spain	Both	0.35	0.45	0.21
2009	Spain	Female	0.29	0.46	0.17
2009	Spain	Male	0.41	0.57	0.19
2010	Greece	Both	0.63	1.41	0.35
2010	Greece	Female	0.81	1.87	0.42
2010	Greece	Male	0.45	1.46	0.16
2010	Italy	Both	0.16	0.52	0.09
2010	Italy	Female	0.11	0.49	0.05
2010	Italy	Male	0.22	0.73	0.12
2010	Portugal	Both	0.23	0.32	0.08
2010	Portugal	Female	0.15	0.22	0.04
2010	Portugal	Male	0.32	0.45	0.09
2010	Spain	Both	0.31	0.42	0.20
2010	Spain	Female	0.26	0.44	0.15
2010	Spain	Male	0.36	0.53	0.17
2011	Greece	Both	0.65	1.42	0.38
2011	Greece	Female	0.83	1.84	0.42
2011	Greece	Male	0.46	1.43	0.18
2011	Italy	Both	0.15	0.49	0.08
2011	Italy	Female	0.10	0.48	0.05
2011	Italy	Male	0.19	0.69	0.11
2011	Portugal	Both	0.19	0.26	0.07
2011	Portugal	Female	0.13	0.19	0.03
2011	Portugal	Male	0.25	0.37	0.08
2011	Spain	Both	0.27	0.40	0.18
2011	Spain	Female	0.22	0.40	0.13
2011	Spain	Male	0.31	0.51	0.16

2012	Greece	Both	0.62	1.30	0.37
2012	Greece	Female	0.79	1.69	0.38
2012	Greece	Male	0.44	1.31	0.18
2012	Italy	Both	0.14	0.46	0.07
2012	Italy	Female	0.10	0.46	0.04
2012	Italy	Male	0.18	0.66	0.09
2012	Portugal	Both	0.17	0.23	0.06
2012	Portugal	Female	0.11	0.16	0.02
2012	Portugal	Male	0.22	0.35	0.07
2012	Spain	Both	0.24	0.38	0.17
2012	Spain	Female	0.20	0.38	0.11
2012	Spain	Male	0.27	0.48	0.16
2013	Greece	Both	0.54	1.07	0.33
2013	Greece	Female	0.68	1.38	0.32
2013	Greece	Male	0.38	1.08	0.17
2013	Italy	Both	0.13	0.43	0.06
2013	Italy	Female	0.09	0.43	0.04
2013	Italy	Male	0.16	0.61	0.08
2013	Portugal	Both	0.15	0.20	0.06
2013	Portugal	Female	0.11	0.16	0.02
2013	Portugal	Male	0.19	0.30	0.06
2013	Spain	Both	0.22	0.35	0.16
2013	Spain	Female	0.18	0.36	0.10
2013	Spain	Male	0.26	0.47	0.14
2014	Greece	Both	0.36	0.67	0.21
2014	Greece	Female	0.45	0.83	0.19
2014	Greece	Male	0.26	0.68	0.12
2014	Italy	Both	0.12	0.41	0.06
2014	Italy	Female	0.08	0.40	0.04
2014	Italy	Male	0.16	0.57	0.08
2014	Portugal	Both	0.13	0.18	0.05
2014	Portugal	Female	0.09	0.13	0.02
2014	Portugal	Male	0.17	0.28	0.06
2014	Spain	Both	0.21	0.35	0.15
2014	Spain	Female	0.18	0.35	0.10
2014	Spain	Male	0.25	0.45	0.14
2015	Greece	Both	0.29	0.55	0.17
2015	Greece	Female	0.35	0.61	0.13
2015	Greece	Male	0.22	0.61	0.10
2015	Italy	Both	0.12	0.38	0.06
2015	Italy	Female	0.08	0.39	0.04
2015	Italy	Male	0.15	0.53	0.08
2015	Portugal	Both	0.12	0.18	0.05
2015	Portugal	Female	0.09	0.13	0.01
2015	Portugal	Male	0.17	0.26	0.05
2015	Spain	Both	0.21	0.33	0.15
2015	Spain	Female	0.18	0.33	0.10
2015	Spain	Male	0.25	0.44	0.13
2016	Greece	Both	0.27	0.51	0.16
2016	Greece	Female	0.33	0.59	0.13

2016	Greece	Male	0.21	0.57	0.10
2016	Italy	Both	0.11	0.34	0.06
2016	Italy	Female	0.08	0.34	0.03
2016	Italy	Male	0.15	0.47	0.08
2016	Portugal	Both	0.12	0.17	0.05
2016	Portugal	Female	0.08	0.13	0.01
2016	Portugal	Male	0.16	0.25	0.05
2016	Spain	Both	0.21	0.32	0.14
2016	Spain	Female	0.18	0.32	0.10
2016	Spain	Male	0.25	0.44	0.13
2017	Greece	Both	0.28	0.54	0.17
2017	Greece	Female	0.33	0.61	0.13
2017	Greece	Male	0.22	0.61	0.10
2017	Italy	Both	0.12	0.36	0.07
2017	Italy	Female	0.09	0.36	0.04
2017	Italy	Male	0.16	0.49	0.09
2017	Portugal	Both	0.12	0.17	0.04
2017	Portugal	Female	0.08	0.13	0.01
2017	Portugal	Male	0.16	0.26	0.05
2017	Spain	Both	0.21	0.32	0.14
2017	Spain	Female	0.18	0.32	0.09
2017	Spain	Male	0.25	0.45	0.13
2018	Greece	Both	0.28	0.55	0.17
2018	Greece	Female	0.33	0.61	0.13
2018	Greece	Male	0.22	0.61	0.10
2018	Italy	Both	0.12	0.36	0.07
2018	Italy	Female	0.09	0.35	0.04
2018	Italy	Male	0.16	0.50	0.09
2018	Portugal	Both	0.12	0.18	0.04
2018	Portugal	Female	0.08	0.12	0.01
2018	Portugal	Male	0.17	0.28	0.05
2018	Spain	Both	0.21	0.32	0.14
2018	Spain	Female	0.18	0.32	0.09
2018	Spain	Male	0.25	0.44	0.13
2019	Greece	Both	0.27	0.53	0.16
2019	Greece	Female	0.32	0.59	0.13
2019	Greece	Male	0.22	0.60	0.10
2019	Italy	Both	0.12	0.35	0.07
2019	Italy	Female	0.08	0.34	0.04
2019	Italy	Male	0.16	0.49	0.09
2019	Portugal	Both	0.12	0.18	0.04
2019	Portugal	Female	0.08	0.12	0.01
2019	Portugal	Male	0.17	0.28	0.05
2019	Spain	Both	0.21	0.31	0.14
2019	Spain	Female	0.17	0.31	0.09
2019	Spain	Male	0.25	0.44	0.12

Table S11- Age-standardized rates and 95% uncertainty levels (UL) of **acute HCV years lived with disability** (YLDs) per 100,000 population in Greece, Italy, Portugal and Spain from 2000 to 2019 by sex classes (Global Burden of Disease Study 2019).

Year	Country	Sex	YLDs (95% UL)	95% UL (upper)	95% UL (lower)
2000	Greece	Both	0.03	0.05	0.01
2000	Greece	Female	0.03	0.06	0.01
2000	Greece	Male	0.02	0.05	0.01
2000	Italy	Both	0.15	0.30	0.07
2000	Italy	Female	0.15	0.31	0.07
2000	Italy	Male	0.15	0.30	0.07
2000	Portugal	Both	0.06	0.13	0.03
2000	Portugal	Female	0.06	0.12	0.03
2000	Portugal	Male	0.07	0.14	0.03
2000	Spain	Both	0.06	0.13	0.03
2000	Spain	Female	0.06	0.11	0.02
2000	Spain	Male	0.07	0.14	0.03
2001	Greece	Both	0.03	0.06	0.01
2001	Greece	Female	0.03	0.06	0.01
2001	Greece	Male	0.03	0.05	0.01
2001	Italy	Both	0.15	0.30	0.07
2001	Italy	Female	0.15	0.30	0.07
2001	Italy	Male	0.15	0.29	0.07
2001	Portugal	Both	0.06	0.13	0.03
2001	Portugal	Female	0.06	0.12	0.03
2001	Portugal	Male	0.07	0.14	0.03
2001	Spain	Both	0.06	0.12	0.03
2001	Spain	Female	0.05	0.11	0.02
2001	Spain	Male	0.07	0.14	0.03
2002	Greece	Both	0.03	0.06	0.01
2002	Greece	Female	0.03	0.06	0.01
2002	Greece	Male	0.03	0.06	0.01
2002	Italy	Both	0.14	0.28	0.07
2002	Italy	Female	0.14	0.29	0.07
2002	Italy	Male	0.14	0.28	0.07
2002	Portugal	Both	0.06	0.13	0.03
2002	Portugal	Female	0.06	0.12	0.03
2002	Portugal	Male	0.07	0.13	0.03
2002	Spain	Both	0.06	0.12	0.03
2002	Spain	Female	0.05	0.10	0.02
2002	Spain	Male	0.07	0.13	0.03
2003	Greece	Both	0.03	0.07	0.01
2003	Greece	Female	0.03	0.07	0.01
2003	Greece	Male	0.03	0.06	0.01
2003	Italy	Both	0.14	0.27	0.06
2003	Italy	Female	0.14	0.27	0.07
2003	Italy	Male	0.13	0.26	0.06
2003	Portugal	Both	0.06	0.12	0.03
2003	Portugal	Female	0.06	0.12	0.02
2003	Portugal	Male	0.07	0.13	0.03
2003	Spain	Both	0.06	0.11	0.03
2003	Spain	Female	0.05	0.10	0.02

2003	Spain	Male	0.06	0.13	0.03
2004	Greece	Both	0.03	0.07	0.01
2004	Greece	Female	0.03	0.08	0.01
2004	Greece	Male	0.03	0.07	0.01
2004	Italy	Both	0.13	0.26	0.06
2004	Italy	Female	0.13	0.26	0.06
2004	Italy	Male	0.13	0.25	0.06
2004	Portugal	Both	0.06	0.12	0.03
2004	Portugal	Female	0.06	0.11	0.02
2004	Portugal	Male	0.07	0.13	0.03
2004	Spain	Both	0.05	0.11	0.02
2004	Spain	Female	0.05	0.10	0.02
2004	Spain	Male	0.06	0.12	0.03
2005	Greece	Both	0.03	0.07	0.01
2005	Greece	Female	0.04	0.08	0.01
2005	Greece	Male	0.03	0.07	0.01
2005	Italy	Both	0.13	0.25	0.06
2005	Italy	Female	0.13	0.26	0.06
2005	Italy	Male	0.12	0.25	0.06
2005	Portugal	Both	0.06	0.12	0.03
2005	Portugal	Female	0.05	0.11	0.02
2005	Portugal	Male	0.07	0.13	0.03
2005	Spain	Both	0.05	0.11	0.02
2005	Spain	Female	0.05	0.10	0.02
2005	Spain	Male	0.06	0.12	0.03
2006	Greece	Both	0.03	0.07	0.01
2006	Greece	Female	0.04	0.08	0.01
2006	Greece	Male	0.03	0.07	0.01
2006	Italy	Both	0.12	0.25	0.06
2006	Italy	Female	0.13	0.25	0.06
2006	Italy	Male	0.12	0.25	0.06
2006	Portugal	Both	0.06	0.12	0.03
2006	Portugal	Female	0.05	0.11	0.02
2006	Portugal	Male	0.07	0.13	0.03
2006	Spain	Both	0.06	0.11	0.02
2006	Spain	Female	0.05	0.10	0.02
2006	Spain	Male	0.06	0.12	0.03
2007	Greece	Both	0.03	0.07	0.01
2007	Greece	Female	0.04	0.08	0.01
2007	Greece	Male	0.03	0.07	0.01
2007	Italy	Both	0.12	0.25	0.06
2007	Italy	Female	0.12	0.25	0.06
2007	Italy	Male	0.12	0.25	0.06
2007	Portugal	Both	0.06	0.12	0.03
2007	Portugal	Female	0.05	0.11	0.02
2007	Portugal	Male	0.06	0.13	0.03
2007	Spain	Both	0.06	0.12	0.03
2007	Spain	Female	0.05	0.11	0.02
2007	Spain	Male	0.06	0.13	0.03
2008	Greece	Both	0.03	0.07	0.01
2008	Greece	Female	0.04	0.08	0.01
2008	Greece	Male	0.03	0.07	0.01
2008	Italy	Both	0.12	0.25	0.06

2008	Italy	Female	0.12	0.25	0.06
2008	Italy	Male	0.12	0.25	0.06
2008	Portugal	Both	0.06	0.12	0.03
2008	Portugal	Female	0.05	0.11	0.02
2008	Portugal	Male	0.06	0.13	0.03
2008	Spain	Both	0.06	0.13	0.03
2008	Spain	Female	0.06	0.12	0.02
2008	Spain	Male	0.07	0.13	0.03
2009	Greece	Both	0.03	0.07	0.01
2009	Greece	Female	0.04	0.08	0.01
2009	Greece	Male	0.03	0.07	0.01
2009	Italy	Both	0.12	0.24	0.06
2009	Italy	Female	0.12	0.24	0.06
2009	Italy	Male	0.12	0.24	0.06
2009	Portugal	Both	0.06	0.12	0.03
2009	Portugal	Female	0.05	0.11	0.02
2009	Portugal	Male	0.06	0.13	0.03
2009	Spain	Both	0.06	0.13	0.03
2009	Spain	Female	0.06	0.12	0.03
2009	Spain	Male	0.07	0.14	0.03
2010	Greece	Both	0.03	0.07	0.01
2010	Greece	Female	0.04	0.08	0.01
2010	Greece	Male	0.03	0.07	0.01
2010	Italy	Both	0.12	0.24	0.06
2010	Italy	Female	0.12	0.24	0.06
2010	Italy	Male	0.12	0.24	0.06
2010	Portugal	Both	0.06	0.12	0.03
2010	Portugal	Female	0.05	0.11	0.02
2010	Portugal	Male	0.06	0.13	0.03
2010	Spain	Both	0.07	0.13	0.03
2010	Spain	Female	0.06	0.13	0.03
2010	Spain	Male	0.07	0.14	0.03
2011	Greece	Both	0.03	0.07	0.01
2011	Greece	Female	0.04	0.08	0.01
2011	Greece	Male	0.03	0.07	0.01
2011	Italy	Both	0.12	0.24	0.05
2011	Italy	Female	0.12	0.23	0.05
2011	Italy	Male	0.12	0.24	0.06
2011	Portugal	Both	0.06	0.12	0.03
2011	Portugal	Female	0.05	0.11	0.02
2011	Portugal	Male	0.06	0.13	0.03
2011	Spain	Both	0.07	0.13	0.03
2011	Spain	Female	0.06	0.12	0.03
2011	Spain	Male	0.07	0.14	0.03
2012	Greece	Both	0.03	0.07	0.01
2012	Greece	Female	0.04	0.08	0.01
2012	Greece	Male	0.03	0.07	0.01
2012	Italy	Both	0.11	0.23	0.05
2012	Italy	Female	0.11	0.23	0.05
2012	Italy	Male	0.11	0.23	0.05
2012	Portugal	Both	0.06	0.12	0.03
2012	Portugal	Female	0.05	0.11	0.02
2012	Portugal	Male	0.06	0.13	0.03

2012	Spain	Both	0.06	0.13	0.03
2012	Spain	Female	0.06	0.12	0.03
2012	Spain	Male	0.07	0.14	0.03
2013	Greece	Both	0.03	0.07	0.01
2013	Greece	Female	0.04	0.08	0.01
2013	Greece	Male	0.03	0.07	0.01
2013	Italy	Both	0.11	0.22	0.05
2013	Italy	Female	0.11	0.22	0.05
2013	Italy	Male	0.11	0.22	0.05
2013	Portugal	Both	0.06	0.12	0.02
2013	Portugal	Female	0.05	0.11	0.02
2013	Portugal	Male	0.06	0.13	0.03
2013	Spain	Both	0.06	0.13	0.03
2013	Spain	Female	0.06	0.12	0.03
2013	Spain	Male	0.07	0.14	0.03
2014	Greece	Both	0.03	0.07	0.01
2014	Greece	Female	0.04	0.08	0.01
2014	Greece	Male	0.03	0.07	0.01
2014	Italy	Both	0.10	0.21	0.05
2014	Italy	Female	0.11	0.21	0.05
2014	Italy	Male	0.10	0.21	0.05
2014	Portugal	Both	0.06	0.12	0.02
2014	Portugal	Female	0.05	0.11	0.02
2014	Portugal	Male	0.06	0.12	0.03
2014	Spain	Both	0.06	0.13	0.03
2014	Spain	Female	0.06	0.12	0.03
2014	Spain	Male	0.07	0.13	0.03
2015	Greece	Both	0.03	0.07	0.01
2015	Greece	Female	0.04	0.08	0.01
2015	Greece	Male	0.03	0.07	0.01
2015	Italy	Both	0.10	0.21	0.05
2015	Italy	Female	0.10	0.21	0.05
2015	Italy	Male	0.10	0.21	0.05
2015	Portugal	Both	0.06	0.12	0.02
2015	Portugal	Female	0.05	0.11	0.02
2015	Portugal	Male	0.06	0.12	0.03
2015	Spain	Both	0.06	0.13	0.03
2015	Spain	Female	0.06	0.12	0.03
2015	Spain	Male	0.07	0.13	0.03
2016	Greece	Both	0.03	0.07	0.01
2016	Greece	Female	0.04	0.08	0.01
2016	Greece	Male	0.03	0.07	0.01
2016	Italy	Both	0.10	0.21	0.05
2016	Italy	Female	0.10	0.21	0.05
2016	Italy	Male	0.10	0.21	0.05
2016	Portugal	Both	0.06	0.12	0.02
2016	Portugal	Female	0.05	0.11	0.02
2016	Portugal	Male	0.06	0.12	0.03
2016	Spain	Both	0.06	0.13	0.03
2016	Spain	Female	0.06	0.12	0.03
2016	Spain	Male	0.07	0.13	0.03
2017	Greece	Both	0.03	0.07	0.01
2017	Greece	Female	0.04	0.07	0.01

2017	Greece	Male	0.03	0.07	0.01
2017	Italy	Both	0.10	0.21	0.05
2017	Italy	Female	0.11	0.21	0.05
2017	Italy	Male	0.11	0.21	0.05
2017	Portugal	Both	0.06	0.12	0.03
2017	Portugal	Female	0.05	0.11	0.02
2017	Portugal	Male	0.06	0.13	0.03
2017	Spain	Both	0.06	0.13	0.03
2017	Spain	Female	0.06	0.12	0.03
2017	Spain	Male	0.07	0.13	0.03
2018	Greece	Both	0.03	0.07	0.01
2018	Greece	Female	0.04	0.08	0.01
2018	Greece	Male	0.03	0.07	0.01
2018	Italy	Both	0.11	0.21	0.05
2018	Italy	Female	0.11	0.21	0.05
2018	Italy	Male	0.11	0.21	0.05
2018	Portugal	Both	0.06	0.12	0.02
2018	Portugal	Female	0.05	0.11	0.02
2018	Portugal	Male	0.06	0.13	0.03
2018	Spain	Both	0.06	0.13	0.03
2018	Spain	Female	0.06	0.12	0.03
2018	Spain	Male	0.07	0.13	0.03
2019	Greece	Both	0.03	0.07	0.01
2019	Greece	Female	0.04	0.08	0.01
2019	Greece	Male	0.03	0.07	0.01
2019	Italy	Both	0.10	0.21	0.05
2019	Italy	Female	0.11	0.21	0.05
2019	Italy	Male	0.10	0.21	0.05
2019	Portugal	Both	0.06	0.12	0.02
2019	Portugal	Female	0.05	0.11	0.02
2019	Portugal	Male	0.06	0.12	0.03
2019	Spain	Both	0.06	0.13	0.03
2019	Spain	Female	0.06	0.12	0.03
2019	Spain	Male	0.07	0.14	0.03

Table S12- Age-standardized rates and 95% uncertainty levels (UL) of **acute HCV disability-adjusted life years** (DALYs) per 100,000 population in Greece, Italy, Portugal and Spain from 2000 to 2019 by sex classes (Global Burden of Disease Study 2019).

Year	Country	Sex	DALYs (95% UL)	95% UL (upper)	95% UL (lower)
2000	Greece	Both	0.23	0.51	0.13
2000	Greece	Female	0.27	0.59	0.14
2000	Greece	Male	0.20	0.59	0.08
2000	Italy	Both	0.59	0.82	0.36
2000	Italy	Female	0.46	0.73	0.27
2000	Italy	Male	0.73	1.10	0.32
2000	Portugal	Both	0.99	2.09	0.57
2000	Portugal	Female	0.48	1.10	0.27
2000	Portugal	Male	1.51	3.56	0.70
2000	Spain	Both	0.62	0.82	0.42
2000	Spain	Female	0.51	0.78	0.35
2000	Spain	Male	0.74	1.02	0.39
2001	Greece	Both	0.23	0.48	0.13
2001	Greece	Female	0.26	0.55	0.13
2001	Greece	Male	0.19	0.57	0.08
2001	Italy	Both	0.57	0.80	0.34
2001	Italy	Female	0.45	0.71	0.27
2001	Italy	Male	0.70	1.07	0.31
2001	Portugal	Both	0.77	1.20	0.39
2001	Portugal	Female	0.43	0.69	0.19
2001	Portugal	Male	1.13	1.92	0.41
2001	Spain	Both	0.60	0.80	0.38
2001	Spain	Female	0.48	0.75	0.33
2001	Spain	Male	0.72	0.99	0.38
2002	Greece	Both	0.24	0.51	0.14
2002	Greece	Female	0.29	0.60	0.15
2002	Greece	Male	0.20	0.57	0.09
2002	Italy	Both	0.56	0.78	0.33
2002	Italy	Female	0.43	0.68	0.25
2002	Italy	Male	0.69	1.05	0.31
2002	Portugal	Both	0.71	1.00	0.32
2002	Portugal	Female	0.40	0.58	0.16
2002	Portugal	Male	1.03	1.52	0.34
2002	Spain	Both	0.57	0.79	0.37
2002	Spain	Female	0.45	0.78	0.30
2002	Spain	Male	0.69	0.96	0.35
2003	Greece	Both	0.25	0.54	0.14
2003	Greece	Female	0.29	0.61	0.15
2003	Greece	Male	0.22	0.64	0.09
2003	Italy	Both	0.56	0.81	0.33
2003	Italy	Female	0.44	0.73	0.24
2003	Italy	Male	0.70	1.08	0.32
2003	Portugal	Both	0.61	0.81	0.26

2003	Portugal	Female	0.34	0.51	0.15
2003	Portugal	Male	0.89	1.24	0.28
2003	Spain	Both	0.56	0.75	0.34
2003	Spain	Female	0.44	0.75	0.29
2003	Spain	Male	0.68	0.92	0.33
2004	Greece	Both	0.28	0.61	0.16
2004	Greece	Female	0.32	0.66	0.17
2004	Greece	Male	0.24	0.76	0.10
2004	Italy	Both	0.53	0.80	0.31
2004	Italy	Female	0.41	0.73	0.23
2004	Italy	Male	0.65	1.06	0.30
2004	Portugal	Both	0.51	0.69	0.24
2004	Portugal	Female	0.30	0.43	0.14
2004	Portugal	Male	0.74	1.06	0.27
2004	Spain	Both	0.53	0.69	0.32
2004	Spain	Female	0.42	0.67	0.26
2004	Spain	Male	0.64	0.86	0.32
2005	Greece	Both	0.33	0.75	0.18
2005	Greece	Female	0.39	0.87	0.20
2005	Greece	Male	0.28	0.87	0.11
2005	Italy	Both	0.49	0.78	0.30
2005	Italy	Female	0.38	0.73	0.21
2005	Italy	Male	0.60	1.03	0.28
2005	Portugal	Both	0.48	0.64	0.19
2005	Portugal	Female	0.28	0.38	0.12
2005	Portugal	Male	0.69	0.95	0.21
2005	Spain	Both	0.51	0.65	0.31
2005	Spain	Female	0.41	0.62	0.26
2005	Spain	Male	0.61	0.82	0.30
2006	Greece	Both	0.39	0.91	0.21
2006	Greece	Female	0.47	1.07	0.24
2006	Greece	Male	0.32	1.04	0.12
2006	Italy	Both	0.45	0.78	0.27
2006	Italy	Female	0.35	0.73	0.20
2006	Italy	Male	0.55	1.02	0.28
2006	Portugal	Both	0.41	0.56	0.17
2006	Portugal	Female	0.26	0.36	0.10
2006	Portugal	Male	0.57	0.80	0.19
2006	Spain	Both	0.49	0.62	0.30
2006	Spain	Female	0.40	0.57	0.25
2006	Spain	Male	0.58	0.78	0.29
2007	Greece	Both	0.50	1.17	0.26
2007	Greece	Female	0.61	1.41	0.31
2007	Greece	Male	0.40	1.33	0.14
2007	Italy	Both	0.40	0.76	0.26
2007	Italy	Female	0.32	0.71	0.19
2007	Italy	Male	0.50	1.01	0.27
2007	Portugal	Both	0.41	0.56	0.16
2007	Portugal	Female	0.26	0.36	0.10
2007	Portugal	Male	0.56	0.80	0.17

2007	Spain	Both	0.47	0.60	0.30
2007	Spain	Female	0.39	0.56	0.25
2007	Spain	Male	0.56	0.74	0.28
2008	Greece	Both	0.49	1.14	0.26
2008	Greece	Female	0.59	1.36	0.30
2008	Greece	Male	0.39	1.29	0.14
2008	Italy	Both	0.35	0.73	0.23
2008	Italy	Female	0.28	0.69	0.16
2008	Italy	Male	0.42	0.96	0.26
2008	Portugal	Both	0.37	0.51	0.15
2008	Portugal	Female	0.24	0.34	0.09
2008	Portugal	Male	0.50	0.71	0.16
2008	Spain	Both	0.45	0.56	0.29
2008	Spain	Female	0.37	0.54	0.24
2008	Spain	Male	0.53	0.69	0.27
2009	Greece	Both	0.62	1.40	0.34
2009	Greece	Female	0.77	1.77	0.41
2009	Greece	Male	0.46	1.51	0.17
2009	Italy	Both	0.31	0.69	0.19
2009	Italy	Female	0.25	0.65	0.14
2009	Italy	Male	0.37	0.91	0.23
2009	Portugal	Both	0.33	0.45	0.14
2009	Portugal	Female	0.23	0.32	0.09
2009	Portugal	Male	0.43	0.61	0.16
2009	Spain	Both	0.42	0.53	0.28
2009	Spain	Female	0.35	0.52	0.23
2009	Spain	Male	0.49	0.64	0.25
2010	Greece	Both	0.67	1.46	0.38
2010	Greece	Female	0.85	1.90	0.45
2010	Greece	Male	0.49	1.49	0.20
2010	Italy	Both	0.29	0.66	0.17
2010	Italy	Female	0.24	0.62	0.12
2010	Italy	Male	0.34	0.87	0.20
2010	Portugal	Both	0.30	0.40	0.14
2010	Portugal	Female	0.21	0.30	0.09
2010	Portugal	Male	0.38	0.55	0.15
2010	Spain	Both	0.38	0.50	0.26
2010	Spain	Female	0.32	0.50	0.21
2010	Spain	Male	0.44	0.60	0.24
2011	Greece	Both	0.69	1.45	0.41
2011	Greece	Female	0.87	1.88	0.46
2011	Greece	Male	0.50	1.49	0.21
2011	Italy	Both	0.27	0.62	0.15
2011	Italy	Female	0.23	0.61	0.11
2011	Italy	Male	0.32	0.81	0.18
2011	Portugal	Both	0.25	0.35	0.12
2011	Portugal	Female	0.19	0.28	0.07
2011	Portugal	Male	0.32	0.46	0.14
2011	Spain	Both	0.34	0.46	0.25
2011	Spain	Female	0.29	0.47	0.19

2011	Spain	Male	0.39	0.58	0.23
2012	Greece	Both	0.66	1.34	0.40
2012	Greece	Female	0.83	1.72	0.43
2012	Greece	Male	0.48	1.35	0.21
2012	Italy	Both	0.25	0.59	0.14
2012	Italy	Female	0.22	0.58	0.11
2012	Italy	Male	0.30	0.78	0.17
2012	Portugal	Both	0.23	0.32	0.12
2012	Portugal	Female	0.17	0.25	0.07
2012	Portugal	Male	0.29	0.42	0.14
2012	Spain	Both	0.31	0.44	0.22
2012	Spain	Female	0.27	0.44	0.16
2012	Spain	Male	0.35	0.56	0.23
2013	Greece	Both	0.58	1.11	0.36
2013	Greece	Female	0.72	1.43	0.36
2013	Greece	Male	0.42	1.10	0.20
2013	Italy	Both	0.24	0.55	0.13
2013	Italy	Female	0.20	0.53	0.10
2013	Italy	Male	0.28	0.72	0.15
2013	Portugal	Both	0.21	0.29	0.11
2013	Portugal	Female	0.17	0.25	0.07
2013	Portugal	Male	0.26	0.38	0.13
2013	Spain	Both	0.29	0.42	0.21
2013	Spain	Female	0.24	0.41	0.15
2013	Spain	Male	0.33	0.53	0.21
2014	Greece	Both	0.40	0.71	0.26
2014	Greece	Female	0.49	0.88	0.22
2014	Greece	Male	0.30	0.71	0.15
2014	Italy	Both	0.23	0.53	0.12
2014	Italy	Female	0.19	0.49	0.10
2014	Italy	Male	0.27	0.68	0.15
2014	Portugal	Both	0.19	0.27	0.10
2014	Portugal	Female	0.15	0.23	0.06
2014	Portugal	Male	0.24	0.35	0.12
2014	Spain	Both	0.28	0.41	0.20
2014	Spain	Female	0.24	0.41	0.15
2014	Spain	Male	0.32	0.52	0.20
2015	Greece	Both	0.33	0.59	0.21
2015	Greece	Female	0.39	0.66	0.17
2015	Greece	Male	0.26	0.64	0.14
2015	Italy	Both	0.22	0.52	0.12
2015	Italy	Female	0.19	0.49	0.10
2015	Italy	Male	0.26	0.63	0.14
2015	Portugal	Both	0.19	0.27	0.10
2015	Portugal	Female	0.14	0.22	0.06
2015	Portugal	Male	0.23	0.35	0.11
2015	Spain	Both	0.28	0.40	0.20
2015	Spain	Female	0.24	0.40	0.15
2015	Spain	Male	0.32	0.51	0.20
2016	Greece	Both	0.31	0.56	0.20

2016	Greece	Female	0.37	0.63	0.17
2016	Greece	Male	0.25	0.59	0.13
2016	Italy	Both	0.22	0.46	0.13
2016	Italy	Female	0.19	0.43	0.10
2016	Italy	Male	0.26	0.58	0.15
2016	Portugal	Both	0.18	0.26	0.10
2016	Portugal	Female	0.14	0.22	0.06
2016	Portugal	Male	0.22	0.33	0.11
2016	Spain	Both	0.28	0.40	0.20
2016	Spain	Female	0.24	0.39	0.15
2016	Spain	Male	0.32	0.50	0.19
2017	Greece	Both	0.32	0.59	0.20
2017	Greece	Female	0.37	0.64	0.18
2017	Greece	Male	0.26	0.63	0.14
2017	Italy	Both	0.23	0.48	0.13
2017	Italy	Female	0.20	0.45	0.10
2017	Italy	Male	0.27	0.60	0.15
2017	Portugal	Both	0.18	0.26	0.09
2017	Portugal	Female	0.14	0.21	0.06
2017	Portugal	Male	0.23	0.34	0.11
2017	Spain	Both	0.28	0.40	0.20
2017	Spain	Female	0.24	0.38	0.14
2017	Spain	Male	0.32	0.51	0.19
2018	Greece	Both	0.31	0.59	0.20
2018	Greece	Female	0.37	0.65	0.17
2018	Greece	Male	0.26	0.63	0.13
2018	Italy	Both	0.23	0.48	0.13
2018	Italy	Female	0.20	0.44	0.11
2018	Italy	Male	0.27	0.60	0.16
2018	Portugal	Both	0.18	0.27	0.09
2018	Portugal	Female	0.14	0.22	0.05
2018	Portugal	Male	0.24	0.35	0.11
2018	Spain	Both	0.28	0.40	0.19
2018	Spain	Female	0.24	0.37	0.14
2018	Spain	Male	0.33	0.52	0.19
2019	Greece	Both	0.31	0.56	0.20
2019	Greece	Female	0.36	0.63	0.17
2019	Greece	Male	0.26	0.63	0.13
2019	Italy	Both	0.23	0.47	0.14
2019	Italy	Female	0.20	0.44	0.11
2019	Italy	Male	0.27	0.60	0.16
2019	Portugal	Both	0.18	0.27	0.09
2019	Portugal	Female	0.13	0.21	0.05
2019	Portugal	Male	0.24	0.36	0.11
2019	Spain	Both	0.28	0.40	0.19
2019	Spain	Female	0.24	0.37	0.14
2019	Spain	Male	0.33	0.51	0.19

Table S13- Age-standardized rates and 95% uncertainty levels (UL) of **prevalence of cirrhosis and other chronic liver diseases due to hepatitis B** per 100,000 population in Greece, Italy, Portugal and Spain from 2000 to 2019 by sex classes (Global Burden of Disease Study 2019).

Year	Country	Sex	Prevalence (95% UL)	95% UL (upper)	95% UL (lower)
2000	Greece	Both	1835.8	2029.1	1668.0
2000	Greece	Female	1516.1	1696.9	1357.8
2000	Greece	Male	2157.5	2387.2	1954.1
2000	Italy	Both	856.7	951.1	765.7
2000	Italy	Female	729.5	817.9	642.7
2000	Italy	Male	988.5	1094.	884.5
2000	Portugal	Both	1118.0	1367.3	907.74
2000	Portugal	Female	858.4	1059.	677.8
2000	Portugal	Male	1388.6	1707.5	1127.0
2000	Spain	Both	857.1	954.4	757.7
2000	Spain	Female	690.3	789.1	593.0
2000	Spain	Male	1025.9	1152.2	907.40
2001	Greece	Both	1810.9	1998.5	1649.5
2001	Greece	Female	1493.7	1660.7	1343.6
2001	Greece	Male	2129.6	2353.2	1937.3
2001	Italy	Both	842.2	932.9	751.9
2001	Italy	Female	718.1	804.5	634.0
2001	Italy	Male	970.6	1075.	869.1
2001	Portugal	Both	1102.1	1328.2	905.62
2001	Portugal	Female	847.0	1039.	672.4
2001	Portugal	Male	1368.0	1661.1	1126.8
2001	Spain	Both	845.5	938.4	749.7
2001	Spain	Female	679.8	773.6	587.2
2001	Spain	Male	1012.5	1122.6	903.82
2002	Greece	Both	1774.0	1950.8	1610.6
2002	Greece	Female	1460.3	1615.1	1319.5
2002	Greece	Male	2089.0	2307.2	1894.1
2002	Italy	Both	819.2	906.8	733.4
2002	Italy	Female	700.4	786.5	618.9
2002	Italy	Male	942.2	1041.	844.6
2002	Portugal	Both	1076.9	1297.4	888.72
2002	Portugal	Female	831.9	1017.	663.3
2002	Portugal	Male	1332.9	1593.4	1104.2
2002	Spain	Both	826.6	916.4	739.0
2002	Spain	Female	663.5	754.9	575.5
2002	Spain	Male	990.4	1091.	892.6
2003	Greece	Both	1733.9	1905.6	1575.8
2003	Greece	Female	1423.7	1569.0	1284.3
2003	Greece	Male	2045.3	2265.1	1852.8
2003	Italy	Both	793.5	879.6	707.5
2003	Italy	Female	680.7	763.2	602.0
2003	Italy	Male	910.1	1004.	816.3
2003	Portugal	Both	1047.7	1248.7	863.27
2003	Portugal	Female	815.1	992.9	652.0
2003	Portugal	Male	1291.5	1532.4	1060.0
2003	Spain	Both	805.5	890.5	724.6

2003	Spain	Female	645.6	732.1	561.0
2003	Spain	Male	965.6	1068.	865.4
2004	Greece	Both	1699.5	1874.3	1544.5
2004	Greece	Female	1392.1	1537.9	1252.6
2004	Greece	Male	2008.0	2222.5	1814.4
2004	Italy	Both	770.6	853.7	688.0
2004	Italy	Female	663.3	746.6	584.5
2004	Italy	Male	881.5	971.5	791.0
2004	Portugal	Both	1019.7	1206.1	836.97
2004	Portugal	Female	798.7	976.3	636.6
2004	Portugal	Male	1252.1	1461.9	1023.0
2004	Spain	Both	787.2	873.5	707.3
2004	Spain	Female	630.3	720.6	547.1
2004	Spain	Male	943.8	1050.	839.6
2005	Greece	Both	1679.6	1852.9	1524.8
2005	Greece	Female	1373.5	1528.8	1229.2
2005	Greece	Male	1986.6	2215.4	1782.3
2005	Italy	Both	755.9	837.5	673.5
2005	Italy	Female	652.2	736.0	572.7
2005	Italy	Male	863.0	953.5	774.9
2005	Portugal	Both	998.1	1176.	813.6
2005	Portugal	Female	784.7	962.4	623.1
2005	Portugal	Male	1223.1	1441.8	994.49
2005	Spain	Both	777.0	865.4	692.5
2005	Spain	Female	621.9	718.9	534.7
2005	Spain	Male	931.2	1047.	821.9
2006	Greece	Both	1658.8	1826.5	1506.1
2006	Greece	Female	1355.6	1502.6	1215.5
2006	Greece	Male	1963.1	2175.8	1779.4
2006	Italy	Both	739.7	819.9	660.6
2006	Italy	Female	639.3	721.5	562.4
2006	Italy	Male	843.1	927.6	753.9
2006	Portugal	Both	981.1	1150.	807.8
2006	Portugal	Female	770.3	932.7	618.1
2006	Portugal	Male	1203.9	1395.3	983.06
2006	Spain	Both	766.4	851.0	689.2
2006	Spain	Female	613.7	703.3	532.7
2006	Spain	Male	917.9	1027.	817.5
2007	Greece	Both	1623.1	1783.8	1481.1
2007	Greece	Female	1326.5	1467.2	1194.9
2007	Greece	Male	1921.4	2120.1	1751.4
2007	Italy	Both	712.9	790.9	635.5
2007	Italy	Female	617.5	696.5	543.1
2007	Italy	Male	811.2	893.6	725.2
2007	Portugal	Both	963.7	1125.	798.0
2007	Portugal	Female	752.3	906.0	608.5
2007	Portugal	Male	1187.6	1374.3	979.42
2007	Spain	Both	747.5	827.7	675.5
2007	Spain	Female	599.2	680.0	522.5
2007	Spain	Male	894.8	1002.	806.3
2008	Greece	Both	1582.8	1735.3	1452.3
2008	Greece	Female	1294.0	1430.2	1165.0
2008	Greece	Male	1874.0	2072.5	1710.7

2008	Italy	Both	682.6	758.5	607.1
2008	Italy	Female	592.7	667.7	520.0
2008	Italy	Male	775.4	854.2	690.1
2008	Portugal	Both	946.7	1105.	786.5
2008	Portugal	Female	733.0	880.9	593.3
2008	Portugal	Male	1173.1	1370.8	972.45
2008	Spain	Both	726.0	802.9	653.4
2008	Spain	Female	582.6	658.5	505.6
2008	Spain	Male	868.7	964.3	782.7
2009	Greece	Both	1548.4	1700.2	1418.1
2009	Greece	Female	1266.5	1403.5	1130.1
2009	Greece	Male	1833.3	2038.0	1668.8
2009	Italy	Both	655.9	730.1	581.6
2009	Italy	Female	570.9	646.1	500.8
2009	Italy	Male	743.9	820.8	660.8
2009	Portugal	Both	930.4	1096.	775.3
2009	Portugal	Female	714.8	861.3	579.5
2009	Portugal	Male	1159.4	1375.1	964.61
2009	Spain	Both	707.6	783.5	637.0
2009	Spain	Female	568.3	644.8	495.0
2009	Spain	Male	846.5	942.5	761.9
2010	Greece	Both	1530.1	1679.9	1387.8
2010	Greece	Female	1251.8	1397.7	1106.7
2010	Greece	Male	1812.0	2021.9	1636.2
2010	Italy	Both	639.7	713.3	565.2
2010	Italy	Female	557.9	633.4	487.8
2010	Italy	Male	724.5	802.0	642.1
2010	Portugal	Both	915.5	1083.	765.3
2010	Portugal	Female	699.6	847.5	568.5
2010	Portugal	Male	1145.2	1374.5	947.35
2010	Spain	Both	697.8	776.3	622.2
2010	Spain	Female	560.6	640.2	482.5
2010	Spain	Male	834.9	943.1	743.6
2011	Greece	Both	1513.2	1657.4	1383.9
2011	Greece	Female	1237.8	1369.1	1104.5
2011	Greece	Male	1792.9	1987.0	1629.5
2011	Italy	Both	624.2	696.3	553.1
2011	Italy	Female	545.2	617.4	478.6
2011	Italy	Male	706.1	782.4	627.9
2011	Portugal	Both	898.6	1060.	750.3
2011	Portugal	Female	685.0	817.9	560.2
2011	Portugal	Male	1126.2	1333.2	931.49
2011	Spain	Both	689.0	761.5	615.2
2011	Spain	Female	553.0	627.2	479.0
2011	Spain	Male	825.1	925.5	739.7
2012	Greece	Both	1482.3	1619.0	1360.5
2012	Greece	Female	1212.1	1328.9	1091.6
2012	Greece	Male	1758.0	1949.2	1608.7
2012	Italy	Both	599.3	668.2	531.2
2012	Italy	Female	524.4	594.9	460.6
2012	Italy	Male	677.2	750.3	603.8
2012	Portugal	Both	877.1	1031.	727.9
2012	Portugal	Female	667.7	792.2	545.0

2012	Portugal	Male	1101.0	1296.2	914.37
2012	Spain	Both	672.6	741.3	602.0
2012	Spain	Female	538.7	608.8	468.4
2012	Spain	Male	807.1	897.5	726.2
2013	Greece	Both	1446.9	1581.7	1323.4
2013	Greece	Female	1182.6	1291.0	1067.4
2013	Greece	Male	1717.8	1903.5	1563.8
2013	Italy	Both	571.7	636.5	506.7
2013	Italy	Female	501.2	567.8	440.1
2013	Italy	Male	645.4	715.4	573.6
2013	Portugal	Both	853.5	999.7	706.2
2013	Portugal	Female	649.1	770.6	531.1
2013	Portugal	Male	1072.7	1258.7	886.18
2013	Spain	Both	653.5	719.4	587.0
2013	Spain	Female	521.9	589.6	450.5
2013	Spain	Male	786.2	876.9	707.6
2014	Greece	Both	1416.3	1549.5	1288.9
2014	Greece	Female	1157.1	1269.5	1046.6
2014	Greece	Male	1683.2	1870.6	1518.2
2014	Italy	Both	548.1	611.9	485.9
2014	Italy	Female	481.3	544.0	422.2
2014	Italy	Male	618.1	685.1	546.5
2014	Portugal	Both	830.1	973.8	683.6
2014	Portugal	Female	630.6	756.1	512.3
2014	Portugal	Male	1044.6	1231.1	854.68
2014	Spain	Both	636.5	700.7	571.0
2014	Spain	Female	506.8	572.7	434.0
2014	Spain	Male	767.6	858.3	686.1
2015	Greece	Both	1399.8	1544.5	1265.9
2015	Greece	Female	1143.5	1262.7	1026.3
2015	Greece	Male	1664.6	1858.1	1478.3
2015	Italy	Both	535.1	598.0	472.4
2015	Italy	Female	470.5	531.7	412.4
2015	Italy	Male	602.9	672.3	530.6
2015	Portugal	Both	809.0	950.2	660.0
2015	Portugal	Female	613.5	741.5	492.5
2015	Portugal	Male	1019.8	1210.5	824.06
2015	Spain	Both	626.2	692.8	560.3
2015	Spain	Female	497.6	565.2	424.9
2015	Spain	Male	756.6	851.3	671.7
2016	Greece	Both	1392.1	1517.8	1270.7
2016	Greece	Female	1139.4	1249.3	1029.6
2016	Greece	Male	1653.8	1818.6	1500.7
2016	Italy	Both	504.9	563.8	445.1
2016	Italy	Female	444.7	504.4	388.5
2016	Italy	Male	568.1	632.3	500.6
2016	Portugal	Both	785.1	924.2	643.5
2016	Portugal	Female	592.4	707.1	470.4
2016	Portugal	Male	993.2	1166.	818.2
2016	Spain	Both	598.6	657.7	539.0
2016	Spain	Female	476.4	535.4	414.5
2016	Spain	Male	722.7	793.7	649.8
2017	Greece	Both	1385.4	1515.9	1254.3

2017	Greece	Female	1136.0	1269.4	1019.4
2017	Greece	Male	1644.4	1815.0	1480.6
2017	Italy	Both	474.7	532.6	416.8
2017	Italy	Female	418.4	474.1	363.6
2017	Italy	Male	534.0	596.7	467.6
2017	Portugal	Both	763.2	908.9	611.9
2017	Portugal	Female	573.7	691.2	438.7
2017	Portugal	Male	968.3	1161.	786.2
2017	Spain	Both	568.1	626.7	512.2
2017	Spain	Female	453.1	513.5	395.0
2017	Spain	Male	685.1	757.1	614.9
2018	Greece	Both	1380.2	1507.9	1269.1
2018	Greece	Female	1129.8	1252.7	1021.8
2018	Greece	Male	1640.4	1787.8	1509.8
2018	Italy	Both	468.8	524.6	411.8
2018	Italy	Female	412.7	468.3	358.0
2018	Italy	Male	527.8	588.2	462.7
2018	Portugal	Both	739.8	877.6	608.1
2018	Portugal	Female	559.6	678.4	441.3
2018	Portugal	Male	935.2	1114.	777.5
2018	Spain	Both	557.0	608.6	502.0
2018	Spain	Female	443.7	497.5	391.9
2018	Spain	Male	672.7	733.3	604.1
2019	Greece	Both	1374.5	1505.6	1261.0
2019	Greece	Female	1120.9	1239.4	1004.9
2019	Greece	Male	1638.2	1819.3	1480.4
2019	Italy	Both	463.9	520.1	408.0
2019	Italy	Female	407.9	463.8	352.9
2019	Italy	Male	522.7	585.8	459.0
2019	Portugal	Both	706.4	845.9	580.5
2019	Portugal	Female	543.1	676.6	430.6
2019	Portugal	Male	884.2	1052.	727.6
2019	Spain	Both	548.4	605.0	489.1
2019	Spain	Female	435.8	495.0	381.0
2019	Spain	Male	663.8	740.0	587.9

Table S14- Age-standardized rates and 95% uncertainty levels (UL) of **incidence of cirrhosis and other chronic liver diseases due to hepatitis B** per 100,000 population in Greece, Italy, Portugal and Spain from 2000 to 2019 by sex classes (Global Burden of Disease Study 2019).

Year	Country	Sex	Incidence (95% UL)	95% UL (upper)	95% UL (lower)
2000	Greece	Both	3.30	4.76	2.21
2000	Greece	Female	2.36	3.51	1.53
2000	Greece	Male	4.24	6.10	2.83
2000	Italy	Both	5.33	6.65	4.15
2000	Italy	Female	3.00	3.79	2.29
2000	Italy	Male	7.67	9.50	6.01
2000	Portugal	Both	3.98	5.91	2.50
2000	Portugal	Female	2.11	3.24	1.29
2000	Portugal	Male	5.93	8.76	3.73
2000	Spain	Both	4.27	5.73	3.11
2000	Spain	Female	2.77	3.75	1.98
2000	Spain	Male	5.76	7.76	4.24
2001	Greece	Both	3.24	4.66	2.17
2001	Greece	Female	2.31	3.37	1.50
2001	Greece	Male	4.18	5.99	2.77
2001	Italy	Both	5.26	6.54	4.13
2001	Italy	Female	2.94	3.69	2.25
2001	Italy	Male	7.58	9.41	5.96
2001	Portugal	Both	3.85	5.70	2.42
2001	Portugal	Female	2.03	3.11	1.24
2001	Portugal	Male	5.74	8.48	3.62
2001	Spain	Both	3.97	5.36	2.89
2001	Spain	Female	2.57	3.46	1.84
2001	Spain	Male	5.35	7.21	3.90
2002	Greece	Both	3.19	4.54	2.13
2002	Greece	Female	2.25	3.26	1.46
2002	Greece	Male	4.12	5.86	2.75
2002	Italy	Both	5.15	6.40	4.07
2002	Italy	Female	2.86	3.62	2.20
2002	Italy	Male	7.42	9.18	5.84
2002	Portugal	Both	3.68	5.52	2.32
2002	Portugal	Female	1.94	2.96	1.18
2002	Portugal	Male	5.49	8.17	3.46
2002	Spain	Both	3.48	4.66	2.52
2002	Spain	Female	2.25	3.01	1.62
2002	Spain	Male	4.70	6.32	3.38
2003	Greece	Both	3.12	4.43	2.08
2003	Greece	Female	2.19	3.16	1.42
2003	Greece	Male	4.06	5.80	2.68
2003	Italy	Both	5.00	6.23	3.96
2003	Italy	Female	2.78	3.52	2.14
2003	Italy	Male	7.22	8.90	5.67
2003	Portugal	Both	3.49	5.28	2.20
2003	Portugal	Female	1.84	2.83	1.12
2003	Portugal	Male	5.20	7.83	3.28
2003	Spain	Both	2.94	3.96	2.13
2003	Spain	Female	1.89	2.53	1.36

2003	Spain	Male	3.96	5.41	2.84
2004	Greece	Both	3.05	4.34	2.02
2004	Greece	Female	2.12	3.07	1.38
2004	Greece	Male	3.98	5.69	2.64
2004	Italy	Both	4.85	6.02	3.83
2004	Italy	Female	2.69	3.40	2.09
2004	Italy	Male	6.99	8.66	5.50
2004	Portugal	Both	3.29	4.96	2.05
2004	Portugal	Female	1.74	2.69	1.05
2004	Portugal	Male	4.89	7.43	3.04
2004	Spain	Both	2.48	3.36	1.79
2004	Spain	Female	1.59	2.16	1.13
2004	Spain	Male	3.34	4.62	2.38
2005	Greece	Both	2.96	4.21	1.95
2005	Greece	Female	2.03	2.96	1.32
2005	Greece	Male	3.88	5.52	2.55
2005	Italy	Both	4.70	5.82	3.70
2005	Italy	Female	2.60	3.30	2.02
2005	Italy	Male	6.77	8.37	5.33
2005	Portugal	Both	3.08	4.69	1.91
2005	Portugal	Female	1.63	2.52	0.99
2005	Portugal	Male	4.58	7.01	2.81
2005	Spain	Both	2.23	3.05	1.60
2005	Spain	Female	1.43	1.97	1.01
2005	Spain	Male	3.01	4.18	2.13
2006	Greece	Both	2.74	3.92	1.81
2006	Greece	Female	1.87	2.74	1.19
2006	Greece	Male	3.62	5.16	2.37
2006	Italy	Both	4.51	5.56	3.57
2006	Italy	Female	2.49	3.17	1.94
2006	Italy	Male	6.51	8.00	5.12
2006	Portugal	Both	2.83	4.31	1.74
2006	Portugal	Female	1.49	2.31	0.90
2006	Portugal	Male	4.22	6.49	2.60
2006	Spain	Both	2.16	2.95	1.56
2006	Spain	Female	1.39	1.89	0.98
2006	Spain	Male	2.91	4.01	2.08
2007	Greece	Both	2.38	3.43	1.55
2007	Greece	Female	1.61	2.38	1.02
2007	Greece	Male	3.16	4.53	2.04
2007	Italy	Both	4.28	5.27	3.40
2007	Italy	Female	2.37	3.03	1.85
2007	Italy	Male	6.17	7.57	4.86
2007	Portugal	Both	2.52	3.88	1.56
2007	Portugal	Female	1.32	2.03	0.79
2007	Portugal	Male	3.77	5.85	2.34
2007	Spain	Both	2.11	2.88	1.53
2007	Spain	Female	1.36	1.87	0.95
2007	Spain	Male	2.84	3.93	2.03
2008	Greece	Both	1.98	2.90	1.28
2008	Greece	Female	1.33	1.99	0.84
2008	Greece	Male	2.64	3.84	1.68
2008	Italy	Both	4.04	5.01	3.21

2008	Italy	Female	2.25	2.89	1.75
2008	Italy	Male	5.81	7.13	4.58
2008	Portugal	Both	2.20	3.44	1.36
2008	Portugal	Female	1.13	1.75	0.68
2008	Portugal	Male	3.32	5.15	2.05
2008	Spain	Both	2.08	2.80	1.51
2008	Spain	Female	1.34	1.85	0.94
2008	Spain	Male	2.78	3.84	1.99
2009	Greece	Both	1.64	2.43	1.05
2009	Greece	Female	1.10	1.66	0.68
2009	Greece	Male	2.20	3.24	1.38
2009	Italy	Both	3.83	4.76	3.04
2009	Italy	Female	2.15	2.76	1.67
2009	Italy	Male	5.50	6.75	4.33
2009	Portugal	Both	1.92	3.00	1.16
2009	Portugal	Female	0.97	1.50	0.58
2009	Portugal	Male	2.92	4.55	1.78
2009	Spain	Both	2.04	2.74	1.49
2009	Spain	Female	1.32	1.82	0.93
2009	Spain	Male	2.73	3.74	1.97
2010	Greece	Both	1.48	2.23	0.94
2010	Greece	Female	0.98	1.49	0.60
2010	Greece	Male	1.99	3.02	1.24
2010	Italy	Both	3.70	4.60	2.93
2010	Italy	Female	2.08	2.67	1.61
2010	Italy	Male	5.30	6.53	4.14
2010	Portugal	Both	1.74	2.70	1.04
2010	Portugal	Female	0.88	1.35	0.52
2010	Portugal	Male	2.65	4.13	1.58
2010	Spain	Both	1.99	2.69	1.45
2010	Spain	Female	1.29	1.80	0.91
2010	Spain	Male	2.66	3.66	1.93
2011	Greece	Both	1.45	2.18	0.91
2011	Greece	Female	0.95	1.45	0.59
2011	Greece	Male	1.94	2.93	1.21
2011	Italy	Both	3.61	4.50	2.85
2011	Italy	Female	2.03	2.60	1.57
2011	Italy	Male	5.18	6.39	4.05
2011	Portugal	Both	1.63	2.50	0.98
2011	Portugal	Female	0.82	1.26	0.49
2011	Portugal	Male	2.48	3.86	1.50
2011	Spain	Both	1.92	2.61	1.39
2011	Spain	Female	1.24	1.73	0.88
2011	Spain	Male	2.57	3.51	1.85
2012	Greece	Both	1.42	2.13	0.89
2012	Greece	Female	0.93	1.42	0.58
2012	Greece	Male	1.92	2.88	1.20
2012	Italy	Both	3.53	4.41	2.79
2012	Italy	Female	1.98	2.54	1.53
2012	Italy	Male	5.07	6.27	3.97
2012	Portugal	Both	1.55	2.38	0.93
2012	Portugal	Female	0.79	1.21	0.47
2012	Portugal	Male	2.35	3.67	1.43

2012	Spain	Both	1.84	2.48	1.32
2012	Spain	Female	1.19	1.65	0.85
2012	Spain	Male	2.47	3.38	1.75
2013	Greece	Both	1.40	2.09	0.89
2013	Greece	Female	0.92	1.38	0.57
2013	Greece	Male	1.89	2.87	1.17
2013	Italy	Both	3.46	4.33	2.73
2013	Italy	Female	1.94	2.48	1.50
2013	Italy	Male	4.98	6.19	3.90
2013	Portugal	Both	1.48	2.28	0.89
2013	Portugal	Female	0.75	1.18	0.45
2013	Portugal	Male	2.24	3.46	1.35
2013	Spain	Both	1.75	2.39	1.26
2013	Spain	Female	1.13	1.58	0.80
2013	Spain	Male	2.36	3.25	1.67
2014	Greece	Both	1.39	2.07	0.87
2014	Greece	Female	0.90	1.36	0.56
2014	Greece	Male	1.87	2.83	1.16
2014	Italy	Both	3.41	4.28	2.69
2014	Italy	Female	1.91	2.45	1.47
2014	Italy	Male	4.92	6.11	3.86
2014	Portugal	Both	1.41	2.19	0.85
2014	Portugal	Female	0.72	1.14	0.44
2014	Portugal	Male	2.14	3.28	1.28
2014	Spain	Both	1.67	2.29	1.19
2014	Spain	Female	1.07	1.51	0.76
2014	Spain	Male	2.25	3.08	1.60
2015	Greece	Both	1.37	2.05	0.86
2015	Greece	Female	0.89	1.34	0.55
2015	Greece	Male	1.85	2.80	1.12
2015	Italy	Both	3.39	4.25	2.67
2015	Italy	Female	1.90	2.43	1.46
2015	Italy	Male	4.89	6.11	3.81
2015	Portugal	Both	1.33	2.06	0.81
2015	Portugal	Female	0.69	1.10	0.41
2015	Portugal	Male	2.02	3.14	1.21
2015	Spain	Both	1.59	2.22	1.13
2015	Spain	Female	1.02	1.46	0.72
2015	Spain	Male	2.15	2.97	1.52
2016	Greece	Both	1.34	2.01	0.84
2016	Greece	Female	0.88	1.32	0.54
2016	Greece	Male	1.81	2.72	1.11
2016	Italy	Both	3.41	4.27	2.68
2016	Italy	Female	1.91	2.47	1.46
2016	Italy	Male	4.90	6.14	3.81
2016	Portugal	Both	1.23	1.91	0.74
2016	Portugal	Female	0.63	0.99	0.38
2016	Portugal	Male	1.87	2.89	1.12
2016	Spain	Both	1.51	2.06	1.09
2016	Spain	Female	0.97	1.33	0.68
2016	Spain	Male	2.03	2.77	1.45
2017	Greece	Both	1.32	1.98	0.81
2017	Greece	Female	0.88	1.34	0.53

2017	Greece	Male	1.77	2.69	1.08
2017	Italy	Both	3.42	4.31	2.67
2017	Italy	Female	1.92	2.51	1.45
2017	Italy	Male	4.92	6.23	3.83
2017	Portugal	Both	1.16	1.81	0.70
2017	Portugal	Female	0.60	0.93	0.35
2017	Portugal	Male	1.76	2.82	1.06
2017	Spain	Both	1.44	1.96	1.04
2017	Spain	Female	0.93	1.31	0.65
2017	Spain	Male	1.95	2.70	1.38
2018	Greece	Both	1.32	1.99	0.81
2018	Greece	Female	0.88	1.32	0.54
2018	Greece	Male	1.76	2.67	1.09
2018	Italy	Both	3.36	4.28	2.58
2018	Italy	Female	1.88	2.46	1.41
2018	Italy	Male	4.84	6.15	3.71
2018	Portugal	Both	1.15	1.79	0.69
2018	Portugal	Female	0.59	0.92	0.36
2018	Portugal	Male	1.75	2.75	1.04
2018	Spain	Both	1.44	1.95	1.03
2018	Spain	Female	0.93	1.29	0.66
2018	Spain	Male	1.93	2.68	1.37
2019	Greece	Both	1.32	1.98	0.82
2019	Greece	Female	0.88	1.33	0.54
2019	Greece	Male	1.76	2.68	1.09
2019	Italy	Both	3.20	4.15	2.42
2019	Italy	Female	1.77	2.35	1.31
2019	Italy	Male	4.62	5.97	3.47
2019	Portugal	Both	1.15	1.80	0.68
2019	Portugal	Female	0.60	0.95	0.36
2019	Portugal	Male	1.75	2.77	1.03
2019	Spain	Both	1.44	1.98	1.04
2019	Spain	Female	0.93	1.31	0.67
2019	Spain	Male	1.94	2.70	1.38

Table S15- Age-standardized rates and 95% uncertainty levels (UL) of **deaths due to cirrhosis and other chronic liver diseases due to hepatitis B** per 100,000 population in Greece, Italy, Portugal and Spain from 2000 to 2019 by sex classes (Global Burden of Disease Study 2019).

Year	Country	Sex	Deaths (95% UL)	95% UL (upper)	95% UL (lower)
2000	Greece	Both	0.99	1.34	0.70
2000	Greece	Female	0.57	0.79	0.39
2000	Greece	Male	1.46	2.03	1.03
2000	Italy	Both	1.59	1.77	1.42
2000	Italy	Female	0.81	0.93	0.70
2000	Italy	Male	2.52	2.78	2.28
2000	Portugal	Both	2.19	2.97	1.56
2000	Portugal	Female	0.97	1.28	0.70
2000	Portugal	Male	3.64	4.96	2.57
2000	Spain	Both	1.66	2.12	1.30
2000	Spain	Female	0.77	1.03	0.58
2000	Spain	Male	2.69	3.40	2.12
2001	Greece	Both	0.96	1.31	0.68
2001	Greece	Female	0.54	0.75	0.36
2001	Greece	Male	1.43	1.98	1.01
2001	Italy	Both	1.52	1.70	1.36
2001	Italy	Female	0.77	0.89	0.66
2001	Italy	Male	2.42	2.68	2.18
2001	Portugal	Both	2.12	2.88	1.52
2001	Portugal	Female	0.92	1.22	0.67
2001	Portugal	Male	3.54	4.81	2.51
2001	Spain	Both	1.55	1.98	1.21
2001	Spain	Female	0.71	0.95	0.53
2001	Spain	Male	2.51	3.19	1.97
2002	Greece	Both	0.93	1.27	0.66
2002	Greece	Female	0.52	0.72	0.36
2002	Greece	Male	1.39	1.90	0.96
2002	Italy	Both	1.45	1.62	1.30
2002	Italy	Female	0.74	0.85	0.63
2002	Italy	Male	2.30	2.55	2.07
2002	Portugal	Both	2.02	2.73	1.44
2002	Portugal	Female	0.86	1.16	0.62
2002	Portugal	Male	3.38	4.58	2.40
2002	Spain	Both	1.37	1.75	1.07
2002	Spain	Female	0.63	0.84	0.46
2002	Spain	Male	2.23	2.85	1.75
2003	Greece	Both	0.92	1.25	0.66
2003	Greece	Female	0.50	0.69	0.35
2003	Greece	Male	1.38	1.90	0.97
2003	Italy	Both	1.39	1.55	1.23
2003	Italy	Female	0.71	0.82	0.60
2003	Italy	Male	2.19	2.43	1.97
2003	Portugal	Both	1.91	2.60	1.36
2003	Portugal	Female	0.81	1.08	0.58
2003	Portugal	Male	3.21	4.41	2.28
2003	Spain	Both	1.18	1.52	0.92
2003	Spain	Female	0.54	0.73	0.40

2003	Spain	Male	1.92	2.49	1.50
2004	Greece	Both	0.90	1.22	0.64
2004	Greece	Female	0.48	0.67	0.33
2004	Greece	Male	1.37	1.88	0.96
2004	Italy	Both	1.28	1.44	1.14
2004	Italy	Female	0.65	0.76	0.55
2004	Italy	Male	2.03	2.26	1.81
2004	Portugal	Both	1.76	2.41	1.25
2004	Portugal	Female	0.73	0.98	0.53
2004	Portugal	Male	2.96	4.09	2.09
2004	Spain	Both	0.99	1.29	0.76
2004	Spain	Female	0.45	0.61	0.33
2004	Spain	Male	1.62	2.07	1.24
2005	Greece	Both	0.89	1.21	0.63
2005	Greece	Female	0.46	0.65	0.32
2005	Greece	Male	1.36	1.86	0.95
2005	Italy	Both	1.21	1.37	1.07
2005	Italy	Female	0.61	0.71	0.52
2005	Italy	Male	1.92	2.15	1.71
2005	Portugal	Both	1.66	2.27	1.17
2005	Portugal	Female	0.67	0.90	0.48
2005	Portugal	Male	2.81	3.90	1.97
2005	Spain	Both	0.90	1.17	0.69
2005	Spain	Female	0.41	0.55	0.29
2005	Spain	Male	1.47	1.91	1.13
2006	Greece	Both	0.83	1.13	0.59
2006	Greece	Female	0.42	0.60	0.29
2006	Greece	Male	1.28	1.76	0.89
2006	Italy	Both	1.14	1.29	1.01
2006	Italy	Female	0.57	0.67	0.48
2006	Italy	Male	1.81	2.03	1.61
2006	Portugal	Both	1.52	2.08	1.06
2006	Portugal	Female	0.59	0.80	0.42
2006	Portugal	Male	2.59	3.56	1.81
2006	Spain	Both	0.86	1.11	0.66
2006	Spain	Female	0.38	0.51	0.28
2006	Spain	Male	1.41	1.82	1.08
2007	Greece	Both	0.74	1.02	0.53
2007	Greece	Female	0.36	0.52	0.25
2007	Greece	Male	1.16	1.59	0.80
2007	Italy	Both	1.08	1.22	0.96
2007	Italy	Female	0.54	0.64	0.46
2007	Italy	Male	1.71	1.91	1.52
2007	Portugal	Both	1.37	1.88	0.96
2007	Portugal	Female	0.54	0.72	0.38
2007	Portugal	Male	2.33	3.23	1.64
2007	Spain	Both	0.84	1.07	0.64
2007	Spain	Female	0.37	0.50	0.27
2007	Spain	Male	1.36	1.75	1.05
2008	Greece	Both	0.62	0.85	0.43
2008	Greece	Female	0.29	0.42	0.20
2008	Greece	Male	0.97	1.34	0.66
2008	Italy	Both	1.02	1.15	0.89

2008	Italy	Female	0.51	0.60	0.43
2008	Italy	Male	1.60	1.80	1.42
2008	Portugal	Both	1.21	1.67	0.84
2008	Portugal	Female	0.47	0.64	0.33
2008	Portugal	Male	2.06	2.88	1.43
2008	Spain	Both	0.80	1.02	0.61
2008	Spain	Female	0.36	0.48	0.26
2008	Spain	Male	1.30	1.68	0.99
2009	Greece	Both	0.51	0.71	0.35
2009	Greece	Female	0.23	0.33	0.16
2009	Greece	Male	0.81	1.14	0.56
2009	Italy	Both	0.96	1.09	0.84
2009	Italy	Female	0.49	0.58	0.40
2009	Italy	Male	1.51	1.70	1.33
2009	Portugal	Both	1.08	1.50	0.74
2009	Portugal	Female	0.42	0.56	0.29
2009	Portugal	Male	1.85	2.57	1.28
2009	Spain	Both	0.76	0.97	0.58
2009	Spain	Female	0.33	0.44	0.24
2009	Spain	Male	1.23	1.60	0.94
2010	Greece	Both	0.45	0.64	0.31
2010	Greece	Female	0.20	0.29	0.14
2010	Greece	Male	0.73	1.03	0.49
2010	Italy	Both	0.91	1.03	0.79
2010	Italy	Female	0.46	0.54	0.38
2010	Italy	Male	1.43	1.61	1.25
2010	Portugal	Both	0.97	1.35	0.66
2010	Portugal	Female	0.37	0.50	0.25
2010	Portugal	Male	1.68	2.35	1.15
2010	Spain	Both	0.72	0.92	0.55
2010	Spain	Female	0.31	0.42	0.23
2010	Spain	Male	1.17	1.52	0.89
2011	Greece	Both	0.45	0.63	0.31
2011	Greece	Female	0.20	0.28	0.14
2011	Greece	Male	0.73	1.02	0.49
2011	Italy	Both	0.87	0.99	0.76
2011	Italy	Female	0.44	0.53	0.37
2011	Italy	Male	1.38	1.56	1.21
2011	Portugal	Both	0.90	1.26	0.61
2011	Portugal	Female	0.33	0.46	0.23
2011	Portugal	Male	1.55	2.18	1.06
2011	Spain	Both	0.68	0.88	0.52
2011	Spain	Female	0.30	0.40	0.22
2011	Spain	Male	1.11	1.44	0.84
2012	Greece	Both	0.45	0.63	0.30
2012	Greece	Female	0.20	0.28	0.14
2012	Greece	Male	0.72	1.01	0.48
2012	Italy	Both	0.85	0.96	0.74
2012	Italy	Female	0.43	0.51	0.36
2012	Italy	Male	1.34	1.52	1.17
2012	Portugal	Both	0.83	1.15	0.56
2012	Portugal	Female	0.30	0.42	0.21
2012	Portugal	Male	1.43	2.03	0.97

2012	Spain	Both	0.64	0.83	0.48
2012	Spain	Female	0.28	0.37	0.20
2012	Spain	Male	1.04	1.36	0.79
2013	Greece	Both	0.44	0.61	0.30
2013	Greece	Female	0.19	0.27	0.13
2013	Greece	Male	0.70	0.99	0.48
2013	Italy	Both	0.82	0.93	0.71
2013	Italy	Female	0.41	0.49	0.34
2013	Italy	Male	1.29	1.46	1.13
2013	Portugal	Both	0.76	1.08	0.52
2013	Portugal	Female	0.28	0.38	0.19
2013	Portugal	Male	1.33	1.90	0.90
2013	Spain	Both	0.59	0.77	0.45
2013	Spain	Female	0.26	0.35	0.19
2013	Spain	Male	0.96	1.26	0.73
2014	Greece	Both	0.43	0.60	0.29
2014	Greece	Female	0.19	0.26	0.13
2014	Greece	Male	0.69	0.96	0.46
2014	Italy	Both	0.79	0.90	0.69
2014	Italy	Female	0.40	0.47	0.33
2014	Italy	Male	1.24	1.41	1.09
2014	Portugal	Both	0.71	1.00	0.48
2014	Portugal	Female	0.26	0.35	0.18
2014	Portugal	Male	1.23	1.75	0.84
2014	Spain	Both	0.56	0.73	0.42
2014	Spain	Female	0.24	0.33	0.18
2014	Spain	Male	0.91	1.20	0.69
2015	Greece	Both	0.42	0.59	0.29
2015	Greece	Female	0.19	0.26	0.13
2015	Greece	Male	0.69	0.95	0.46
2015	Italy	Both	0.79	0.90	0.69
2015	Italy	Female	0.40	0.48	0.34
2015	Italy	Male	1.24	1.41	1.09
2015	Portugal	Both	0.66	0.94	0.45
2015	Portugal	Female	0.25	0.35	0.17
2015	Portugal	Male	1.15	1.64	0.77
2015	Spain	Both	0.55	0.72	0.41
2015	Spain	Female	0.24	0.33	0.17
2015	Spain	Male	0.90	1.18	0.68
2016	Greece	Both	0.42	0.58	0.29
2016	Greece	Female	0.19	0.26	0.13
2016	Greece	Male	0.68	0.95	0.46
2016	Italy	Both	0.76	0.87	0.66
2016	Italy	Female	0.39	0.46	0.32
2016	Italy	Male	1.19	1.36	1.04
2016	Portugal	Both	0.66	0.93	0.45
2016	Portugal	Female	0.25	0.35	0.17
2016	Portugal	Male	1.14	1.64	0.78
2016	Spain	Both	0.53	0.70	0.40
2016	Spain	Female	0.23	0.31	0.16
2016	Spain	Male	0.87	1.14	0.66
2017	Greece	Both	0.42	0.59	0.29
2017	Greece	Female	0.19	0.26	0.12

2017	Greece	Male	0.68	0.96	0.46
2017	Italy	Both	0.76	0.87	0.66
2017	Italy	Female	0.38	0.46	0.32
2017	Italy	Male	1.19	1.37	1.04
2017	Portugal	Both	0.67	0.94	0.45
2017	Portugal	Female	0.25	0.35	0.17
2017	Portugal	Male	1.15	1.63	0.78
2017	Spain	Both	0.53	0.69	0.40
2017	Spain	Female	0.23	0.32	0.16
2017	Spain	Male	0.87	1.14	0.65
2018	Greece	Both	0.42	0.59	0.29
2018	Greece	Female	0.19	0.27	0.13
2018	Greece	Male	0.68	0.95	0.46
2018	Italy	Both	0.77	0.88	0.66
2018	Italy	Female	0.39	0.47	0.32
2018	Italy	Male	1.20	1.37	1.05
2018	Portugal	Both	0.66	0.93	0.44
2018	Portugal	Female	0.25	0.35	0.17
2018	Portugal	Male	1.14	1.60	0.78
2018	Spain	Both	0.53	0.69	0.40
2018	Spain	Female	0.23	0.32	0.17
2018	Spain	Male	0.85	1.11	0.64
2019	Greece	Both	0.42	0.59	0.29
2019	Greece	Female	0.19	0.27	0.13
2019	Greece	Male	0.67	0.95	0.46
2019	Italy	Both	0.77	0.88	0.66
2019	Italy	Female	0.39	0.47	0.32
2019	Italy	Male	1.19	1.36	1.04
2019	Portugal	Both	0.66	0.94	0.45
2019	Portugal	Female	0.25	0.35	0.17
2019	Portugal	Male	1.14	1.61	0.77
2019	Spain	Both	0.52	0.69	0.39
2019	Spain	Female	0.23	0.32	0.17
2019	Spain	Male	0.84	1.11	0.63

Table S16- Age-standardized rates and 95% uncertainty levels (UL) of **years of life lost** (YLLs) **due to cirrhosis and other chronic liver diseases due to hepatitis B** per 100,000 population in Greece, Italy, Portugal and Spain from 2000 to 2019 by sex classes (Global Burden of Disease Study 2019).

Year	Country	Sex	YLLs (95% UL)	95% UL (upper)	95% UL (lower)
2000	Greece	Both	23.9	32.0	17.0
2000	Greece	Female	11.1	15.0	7.93
2000	Greece	Male	37.6	50.7	26.5
2000	Italy	Both	43.1	47.3	39.1
2000	Italy	Female	19.1	21.7	16.6
2000	Italy	Male	69.6	76.1	63.4
2000	Portugal	Both	70.2	96.6	49.3
2000	Portugal	Female	29.6	40.9	20.5
2000	Portugal	Male	115.4	159.2	81.4
2000	Spain	Both	46.2	58.6	36.7
2000	Spain	Female	17.9	22.8	13.9
2000	Spain	Male	76.7	97.1	60.7
2001	Greece	Both	23.5	31.6	16.6
2001	Greece	Female	10.6	14.2	7.52
2001	Greece	Male	37.4	50.5	26.0
2001	Italy	Both	41.4	45.5	37.5
2001	Italy	Female	18.1	20.5	15.7
2001	Italy	Male	67.0	73.6	60.9
2001	Portugal	Both	68.2	94.1	47.7
2001	Portugal	Female	28.3	39.2	19.7
2001	Portugal	Male	112.6	155.6	78.7
2001	Spain	Both	43.4	55.0	34.3
2001	Spain	Female	16.7	21.2	12.9
2001	Spain	Male	72.0	91.7	56.6
2002	Greece	Both	23.0	31.0	16.2
2002	Greece	Female	10.3	13.9	7.33
2002	Greece	Male	36.6	49.3	25.5
2002	Italy	Both	39.3	43.4	35.5
2002	Italy	Female	17.2	19.6	15.0
2002	Italy	Male	63.6	70.0	57.7
2002	Portugal	Both	64.9	90.3	45.2
2002	Portugal	Female	26.5	36.7	18.4
2002	Portugal	Male	107.5	149.4	74.8
2002	Spain	Both	38.4	48.8	30.1
2002	Spain	Female	14.7	18.6	11.4
2002	Spain	Male	63.7	81.2	50.1
2003	Greece	Both	23.0	31.1	16.2
2003	Greece	Female	9.9	13.3	7.0
2003	Greece	Male	37.0	50.0	25.6
2003	Italy	Both	37.4	41.4	33.5
2003	Italy	Female	16.3	18.7	14.1
2003	Italy	Male	60.4	66.7	54.6
2003	Portugal	Both	61.5	85.8	42.8
2003	Portugal	Female	24.7	34.3	17.2
2003	Portugal	Male	102.3	142.6	70.7
2003	Spain	Both	33.1	41.9	25.7
2003	Spain	Female	12.6	16.1	9.69
2003	Spain	Male	54.8	70.0	42.5
2004	Greece	Both	23.0	31.1	16.1

2004	Greece	Female	9.6	12.9	6.9
2004	Greece	Male	37.3	51.1	25.6
2004	Italy	Both	34.6	38.5	30.9
2004	Italy	Female	14.9	17.2	12.9
2004	Italy	Male	56.0	62.0	50.3
2004	Portugal	Both	56.6	79.4	39.3
2004	Portugal	Female	22.3	31.1	15.5
2004	Portugal	Male	94.6	131.5	65.1
2004	Spain	Both	27.7	35.5	21.4
2004	Spain	Female	10.4	13.4	7.93
2004	Spain	Male	46.1	59.0	35.2
2005	Greece	Both	23.0	31.1	16.1
2005	Greece	Female	9.2	12.5	6.6
2005	Greece	Male	37.6	51.7	26.0
2005	Italy	Both	32.7	36.5	29.1
2005	Italy	Female	14.0	16.1	12.0
2005	Italy	Male	53.0	58.9	47.3
2005	Portugal	Both	53.4	75.4	36.9
2005	Portugal	Female	20.5	28.9	14.1
2005	Portugal	Male	89.9	125.9	61.6
2005	Spain	Both	25.2	32.1	19.5
2005	Spain	Female	9.4	12.0	7.1
2005	Spain	Male	41.9	53.9	32.1
2006	Greece	Both	21.8	29.6	15.2
2006	Greece	Female	8.5	11.6	6.1
2006	Greece	Male	35.8	49.2	24.6
2006	Italy	Both	30.8	34.4	27.3
2006	Italy	Female	12.9	15.0	11.1
2006	Italy	Male	50.1	55.7	44.6
2006	Portugal	Both	48.9	69.5	33.4
2006	Portugal	Female	18.1	25.5	12.5
2006	Portugal	Male	83.0	117.3	56.6
2006	Spain	Both	24.1	30.9	18.5
2006	Spain	Female	8.8	11.4	6.6
2006	Spain	Male	40.1	52.3	30.6
2007	Greece	Both	19.9	27.2	13.9
2007	Greece	Female	7.5	10.2	5.3
2007	Greece	Male	33.0	45.7	22.5
2007	Italy	Both	28.9	32.4	25.7
2007	Italy	Female	12.2	14.1	10.4
2007	Italy	Male	47.1	52.5	41.9
2007	Portugal	Both	44.0	62.2	29.9
2007	Portugal	Female	16.2	23.0	11.3
2007	Portugal	Male	74.7	105.	50.6
2007	Spain	Both	23.3	30.2	18.1
2007	Spain	Female	8.5	10.8	6.4
2007	Spain	Male	38.8	51.0	29.8
2008	Greece	Both	16.7	23.0	11.6
2008	Greece	Female	6.2	8.5	4.4
2008	Greece	Male	27.9	38.8	18.7
2008	Italy	Both	27.0	30.3	23.7
2008	Italy	Female	11.3	13.1	9.7
2008	Italy	Male	43.9	49.2	38.8
2008	Portugal	Both	38.6	54.6	26.1
2008	Portugal	Female	14.1	19.8	9.87
2008	Portugal	Male	65.7	93.0	44.3
2008	Spain	Both	22.1	28.9	17.0

2008	Spain	Female	8.2	10.4	6.2
2008	Spain	Male	36.7	48.4	28.2
2009	Greece	Both	14.1	19.6	9.61
2009	Greece	Female	5.0	6.9	3.5
2009	Greece	Male	23.7	33.3	16.0
2009	Italy	Both	25.3	28.4	22.2
2009	Italy	Female	10.6	12.4	9.12
2009	Italy	Male	41.0	46.1	36.1
2009	Portugal	Both	34.5	49.2	23.2
2009	Portugal	Female	12.5	17.5	8.60
2009	Portugal	Male	58.9	84.1	39.0
2009	Spain	Both	20.8	27.2	16.1
2009	Spain	Female	7.6	9.79	5.8
2009	Spain	Male	34.7	45.6	26.5
2010	Greece	Both	12.6	17.7	8.64
2010	Greece	Female	4.5	6.3	3.1
2010	Greece	Male	21.3	30.0	14.4
2010	Italy	Both	23.8	26.9	20.8
2010	Italy	Female	10.0	11.6	8.57
2010	Italy	Male	38.7	43.8	33.9
2010	Portugal	Both	31.1	44.7	20.7
2010	Portugal	Female	11.0	15.4	7.49
2010	Portugal	Male	53.5	77.0	35.4
2010	Spain	Both	19.6	25.6	15.2
2010	Spain	Female	7.1	9.1	5.4
2010	Spain	Male	32.8	43.3	25.0
2011	Greece	Both	12.6	17.7	8.56
2011	Greece	Female	4.4	6.2	3.1
2011	Greece	Male	21.2	30.1	14.2
2011	Italy	Both	22.9	26.0	20.0
2011	Italy	Female	9.6	11.2	8.2
2011	Italy	Male	37.3	42.4	32.7
2011	Portugal	Both	28.6	41.3	19.0
2011	Portugal	Female	9.8	13.8	6.7
2011	Portugal	Male	49.4	71.7	32.7
2011	Spain	Both	18.5	24.3	14.2
2011	Spain	Female	6.7	8.6	5.1
2011	Spain	Male	30.8	40.9	23.7
2012	Greece	Both	12.5	17.6	8.49
2012	Greece	Female	4.5	6.2	3.1
2012	Greece	Male	21.0	29.8	13.9
2012	Italy	Both	22.2	25.2	19.4
2012	Italy	Female	9.3	10.8	7.9
2012	Italy	Male	36.1	41.1	31.6
2012	Portugal	Both	26.1	37.8	17.4
2012	Portugal	Female	8.8	12.4	5.9
2012	Portugal	Male	45.3	66.0	30.2
2012	Spain	Both	17.2	22.7	13.2
2012	Spain	Female	6.2	7.9	4.7
2012	Spain	Male	28.8	38.3	22.1
2013	Greece	Both	12.2	17.1	8.28
2013	Greece	Female	4.3	6.1	3.0
2013	Greece	Male	20.5	29.2	13.5
2013	Italy	Both	21.4	24.4	18.7
2013	Italy	Female	9.0	10.5	7.6
2013	Italy	Male	34.7	39.5	30.4
2013	Portugal	Both	23.9	34.7	15.9

2013	Portugal	Female	8.0	11.3	5.4
2013	Portugal	Male	41.5	60.7	27.7
2013	Spain	Both	15.8	20.8	12.2
2013	Spain	Female	5.8	7.4	4.4
2013	Spain	Male	26.5	35.0	20.3
2014	Greece	Both	11.9	16.8	8.01
2014	Greece	Female	4.3	5.9	3.0
2014	Greece	Male	20.1	28.7	13.3
2014	Italy	Both	20.6	23.4	18.1
2014	Italy	Female	8.6	10.0	7.3
2014	Italy	Male	33.6	38.2	29.3
2014	Portugal	Both	22.1	32.4	14.6
2014	Portugal	Female	7.4	10.5	5.0
2014	Portugal	Male	38.5	56.5	25.4
2014	Spain	Both	14.9	19.7	11.4
2014	Spain	Female	5.4	6.9	4.1
2014	Spain	Male	24.9	32.9	19.0
2015	Greece	Both	11.9	16.6	7.91
2015	Greece	Female	4.2	5.9	3.0
2015	Greece	Male	20.0	28.4	13.0
2015	Italy	Both	20.6	23.5	18.1
2015	Italy	Female	8.7	10.1	7.4
2015	Italy	Male	33.4	38.2	29.4
2015	Portugal	Both	20.4	29.9	13.5
2015	Portugal	Female	7.1	10.2	4.8
2015	Portugal	Male	35.3	51.8	23.1
2015	Spain	Both	14.5	19.2	11.0
2015	Spain	Female	5.3	6.9	4.0
2015	Spain	Male	24.2	32.4	18.5
2016	Greece	Both	11.7	16.7	7.91
2016	Greece	Female	4.3	6.0	3.0
2016	Greece	Male	19.6	28.0	13.0
2016	Italy	Both	19.8	22.6	17.5
2016	Italy	Female	8.4	9.7	7.1
2016	Italy	Male	32.2	36.5	28.2
2016	Portugal	Both	20.2	29.6	13.3
2016	Portugal	Female	7.1	10.3	4.8
2016	Portugal	Male	34.9	51.4	22.8
2016	Spain	Both	13.9	18.4	10.7
2016	Spain	Female	5.1	6.6	3.8
2016	Spain	Male	23.3	31.0	17.7
2017	Greece	Both	11.7	16.8	8.01
2017	Greece	Female	4.3	6.0	3.0
2017	Greece	Male	19.6	28.4	13.2
2017	Italy	Both	19.9	22.7	17.4
2017	Italy	Female	8.4	9.8	7.1
2017	Italy	Male	32.2	37.0	28.2
2017	Portugal	Both	20.3	30.1	13.3
2017	Portugal	Female	7.2	10.3	4.8
2017	Portugal	Male	35.1	52.2	22.8
2017	Spain	Both	13.9	18.4	10.6
2017	Spain	Female	5.1	6.6	3.8
2017	Spain	Male	23.3	31.0	17.6
2018	Greece	Both	11.7	16.4	8.02
2018	Greece	Female	4.4	6.0	3.0
2018	Greece	Male	19.5	28.0	13.1
2018	Italy	Both	19.9	22.7	17.5

2018	Italy	Female	8.4	9.9	7.1
2018	Italy	Male	32.3	36.7	28.4
2018	Portugal	Both	20.5	30.1	13.6
2018	Portugal	Female	7.2	10.3	4.8
2018	Portugal	Male	35.4	51.9	23.4
2018	Spain	Both	13.9	18.2	10.6
2018	Spain	Female	5.2	6.7	3.9
2018	Spain	Male	23.1	30.6	17.6
2019	Greece	Both	11.6	16.2	8.02
2019	Greece	Female	4.4	6.1	3.1
2019	Greece	Male	19.4	27.5	13.1
2019	Italy	Both	19.9	22.7	17.5
2019	Italy	Female	8.5	10.0	7.1
2019	Italy	Male	32.2	36.8	28.1
2019	Portugal	Both	20.5	29.9	13.7
2019	Portugal	Female	7.2	10.2	4.8
2019	Portugal	Male	35.5	51.9	23.5
2019	Spain	Both	13.8	18.2	10.6
2019	Spain	Female	5.2	6.7	3.9
2019	Spain	Male	22.9	30.3	17.5

Table S17- Age-standardized rates and 95% uncertainty levels (UL) of **years lived with disability** (YLDs) **due to cirrhosis and other chronic liver diseases due to hepatitis B** per 100,000 population in Greece, Italy, Portugal and Spain from 2000 to 2019 by sex classes (Global Burden of Disease Study 2019).

Year	Country	Sex	YLDs (95% UL)	95% UL (upper)	95% UL (lower)
2000	Greece	Both	0.57	0.91	0.34
2000	Greece	Female	0.39	0.61	0.24
2000	Greece	Male	0.76	1.27	0.42
2000	Italy	Both	0.84	1.22	0.57
2000	Italy	Female	0.50	0.73	0.33
2000	Italy	Male	1.22	1.78	0.81
2000	Portugal	Both	0.96	1.62	0.52
2000	Portugal	Female	0.59	0.92	0.35
2000	Portugal	Male	1.37	2.37	0.69
2000	Spain	Both	0.80	1.24	0.49
2000	Spain	Female	0.47	0.72	0.29
2000	Spain	Male	1.15	1.88	0.68
2001	Greece	Both	0.57	0.88	0.34
2001	Greece	Female	0.39	0.60	0.23
2001	Greece	Male	0.76	1.22	0.42
2001	Italy	Both	0.83	1.20	0.56
2001	Italy	Female	0.49	0.71	0.33
2001	Italy	Male	1.20	1.74	0.81
2001	Portugal	Both	0.92	1.51	0.51
2001	Portugal	Female	0.56	0.89	0.33
2001	Portugal	Male	1.32	2.21	0.67
2001	Spain	Both	0.74	1.17	0.46
2001	Spain	Female	0.44	0.66	0.28
2001	Spain	Male	1.05	1.73	0.59
2002	Greece	Both	0.56	0.88	0.33
2002	Greece	Female	0.38	0.59	0.23
2002	Greece	Male	0.76	1.24	0.42
2002	Italy	Both	0.81	1.17	0.55
2002	Italy	Female	0.48	0.70	0.32
2002	Italy	Male	1.18	1.71	0.80
2002	Portugal	Both	0.89	1.45	0.49
2002	Portugal	Female	0.54	0.84	0.32
2002	Portugal	Male	1.27	2.13	0.64
2002	Spain	Both	0.64	1.03	0.38
2002	Spain	Female	0.39	0.58	0.25
2002	Spain	Male	0.91	1.53	0.50
2003	Greece	Both	0.56	0.89	0.33
2003	Greece	Female	0.37	0.58	0.22
2003	Greece	Male	0.75	1.26	0.40
2003	Italy	Both	0.79	1.15	0.54
2003	Italy	Female	0.46	0.68	0.31
2003	Italy	Male	1.14	1.67	0.76
2003	Portugal	Both	0.84	1.34	0.47

2003	Portugal	Female	0.51	0.80	0.30
2003	Portugal	Male	1.21	2.02	0.63
2003	Spain	Both	0.55	0.84	0.32
2003	Spain	Female	0.33	0.49	0.21
2003	Spain	Male	0.77	1.24	0.42
2004	Greece	Both	0.55	0.88	0.32
2004	Greece	Female	0.37	0.57	0.22
2004	Greece	Male	0.75	1.25	0.39
2004	Italy	Both	0.76	1.09	0.52
2004	Italy	Female	0.45	0.65	0.30
2004	Italy	Male	1.10	1.59	0.75
2004	Portugal	Both	0.80	1.32	0.44
2004	Portugal	Female	0.48	0.77	0.28
2004	Portugal	Male	1.16	1.98	0.60
2004	Spain	Both	0.47	0.71	0.29
2004	Spain	Female	0.27	0.41	0.17
2004	Spain	Male	0.67	1.04	0.40
2005	Greece	Both	0.55	0.86	0.32
2005	Greece	Female	0.37	0.58	0.22
2005	Greece	Male	0.74	1.20	0.39
2005	Italy	Both	0.74	1.08	0.50
2005	Italy	Female	0.44	0.65	0.29
2005	Italy	Male	1.07	1.56	0.72
2005	Portugal	Both	0.76	1.25	0.43
2005	Portugal	Female	0.45	0.71	0.26
2005	Portugal	Male	1.10	1.87	0.57
2005	Spain	Both	0.42	0.64	0.27
2005	Spain	Female	0.24	0.37	0.15
2005	Spain	Male	0.60	0.93	0.38
2006	Greece	Both	0.52	0.81	0.30
2006	Greece	Female	0.34	0.54	0.21
2006	Greece	Male	0.71	1.14	0.39
2006	Italy	Both	0.72	1.04	0.48
2006	Italy	Female	0.42	0.62	0.28
2006	Italy	Male	1.03	1.49	0.69
2006	Portugal	Both	0.69	1.13	0.39
2006	Portugal	Female	0.41	0.65	0.24
2006	Portugal	Male	1.01	1.70	0.53
2006	Spain	Both	0.41	0.62	0.26
2006	Spain	Female	0.24	0.36	0.15
2006	Spain	Male	0.58	0.89	0.37
2007	Greece	Both	0.46	0.72	0.27
2007	Greece	Female	0.30	0.47	0.18
2007	Greece	Male	0.63	1.04	0.35
2007	Italy	Both	0.69	0.99	0.46
2007	Italy	Female	0.40	0.60	0.27
2007	Italy	Male	0.99	1.41	0.67
2007	Portugal	Both	0.62	1.01	0.33
2007	Portugal	Female	0.36	0.58	0.21
2007	Portugal	Male	0.90	1.54	0.44

2007	Spain	Both	0.40	0.61	0.25
2007	Spain	Female	0.23	0.35	0.14
2007	Spain	Male	0.57	0.86	0.36
2008	Greece	Both	0.40	0.62	0.24
2008	Greece	Female	0.25	0.39	0.15
2008	Greece	Male	0.55	0.87	0.33
2008	Italy	Both	0.66	0.94	0.44
2008	Italy	Female	0.39	0.57	0.25
2008	Italy	Male	0.94	1.34	0.63
2008	Portugal	Both	0.54	0.92	0.30
2008	Portugal	Female	0.32	0.50	0.18
2008	Portugal	Male	0.79	1.40	0.39
2008	Spain	Both	0.39	0.59	0.25
2008	Spain	Female	0.23	0.35	0.14
2008	Spain	Male	0.56	0.84	0.35
2009	Greece	Both	0.33	0.52	0.20
2009	Greece	Female	0.20	0.33	0.12
2009	Greece	Male	0.47	0.74	0.27
2009	Italy	Both	0.63	0.92	0.42
2009	Italy	Female	0.37	0.56	0.25
2009	Italy	Male	0.90	1.31	0.61
2009	Portugal	Both	0.47	0.78	0.26
2009	Portugal	Female	0.27	0.44	0.16
2009	Portugal	Male	0.70	1.19	0.35
2009	Spain	Both	0.38	0.58	0.24
2009	Spain	Female	0.22	0.34	0.14
2009	Spain	Male	0.55	0.82	0.35
2010	Greece	Both	0.30	0.47	0.18
2010	Greece	Female	0.18	0.29	0.11
2010	Greece	Male	0.42	0.66	0.25
2010	Italy	Both	0.61	0.88	0.40
2010	Italy	Female	0.36	0.53	0.24
2010	Italy	Male	0.87	1.27	0.58
2010	Portugal	Both	0.43	0.70	0.23
2010	Portugal	Female	0.24	0.39	0.14
2010	Portugal	Male	0.64	1.10	0.33
2010	Spain	Both	0.37	0.57	0.24
2010	Spain	Female	0.22	0.33	0.13
2010	Spain	Male	0.53	0.81	0.34
2011	Greece	Both	0.29	0.46	0.17
2011	Greece	Female	0.18	0.29	0.10
2011	Greece	Male	0.42	0.65	0.24
2011	Italy	Both	0.59	0.85	0.39
2011	Italy	Female	0.35	0.51	0.23
2011	Italy	Male	0.84	1.23	0.55
2011	Portugal	Both	0.40	0.66	0.22
2011	Portugal	Female	0.22	0.36	0.13
2011	Portugal	Male	0.59	1.03	0.31
2011	Spain	Both	0.36	0.54	0.23
2011	Spain	Female	0.21	0.32	0.13

2011	Spain	Male	0.51	0.77	0.33
2012	Greece	Both	0.29	0.45	0.17
2012	Greece	Female	0.18	0.28	0.10
2012	Greece	Male	0.41	0.65	0.24
2012	Italy	Both	0.57	0.84	0.38
2012	Italy	Female	0.34	0.49	0.22
2012	Italy	Male	0.81	1.22	0.54
2012	Portugal	Both	0.38	0.63	0.21
2012	Portugal	Female	0.21	0.33	0.12
2012	Portugal	Male	0.57	0.96	0.32
2012	Spain	Both	0.34	0.51	0.22
2012	Spain	Female	0.20	0.31	0.12
2012	Spain	Male	0.48	0.74	0.31
2013	Greece	Both	0.29	0.45	0.17
2013	Greece	Female	0.17	0.28	0.10
2013	Greece	Male	0.41	0.64	0.23
2013	Italy	Both	0.55	0.80	0.37
2013	Italy	Female	0.33	0.48	0.22
2013	Italy	Male	0.79	1.16	0.54
2013	Portugal	Both	0.36	0.60	0.20
2013	Portugal	Female	0.20	0.31	0.11
2013	Portugal	Male	0.54	0.92	0.30
2013	Spain	Both	0.32	0.49	0.20
2013	Spain	Female	0.19	0.29	0.12
2013	Spain	Male	0.46	0.69	0.29
2014	Greece	Both	0.28	0.44	0.17
2014	Greece	Female	0.17	0.27	0.10
2014	Greece	Male	0.40	0.64	0.24
2014	Italy	Both	0.53	0.77	0.36
2014	Italy	Female	0.32	0.47	0.21
2014	Italy	Male	0.77	1.11	0.51
2014	Portugal	Both	0.34	0.55	0.20
2014	Portugal	Female	0.18	0.30	0.11
2014	Portugal	Male	0.52	0.84	0.31
2014	Spain	Both	0.30	0.46	0.19
2014	Spain	Female	0.18	0.27	0.11
2014	Spain	Male	0.43	0.65	0.27
2015	Greece	Both	0.28	0.44	0.17
2015	Greece	Female	0.17	0.27	0.10
2015	Greece	Male	0.40	0.63	0.23
2015	Italy	Both	0.53	0.77	0.36
2015	Italy	Female	0.31	0.46	0.20
2015	Italy	Male	0.76	1.13	0.51
2015	Portugal	Both	0.33	0.52	0.19
2015	Portugal	Female	0.18	0.28	0.10
2015	Portugal	Male	0.49	0.81	0.29
2015	Spain	Both	0.29	0.44	0.18
2015	Spain	Female	0.17	0.26	0.11
2015	Spain	Male	0.41	0.62	0.26
2016	Greece	Both	0.28	0.44	0.17

2016	Greece	Female	0.18	0.28	0.10
2016	Greece	Male	0.40	0.63	0.23
2016	Italy	Both	0.54	0.80	0.36
2016	Italy	Female	0.32	0.47	0.21
2016	Italy	Male	0.77	1.14	0.51
2016	Portugal	Both	0.31	0.49	0.18
2016	Portugal	Female	0.17	0.27	0.10
2016	Portugal	Male	0.46	0.75	0.27
2016	Spain	Both	0.28	0.42	0.18
2016	Spain	Female	0.17	0.26	0.10
2016	Spain	Male	0.39	0.60	0.25
2017	Greece	Both	0.29	0.45	0.17
2017	Greece	Female	0.18	0.29	0.11
2017	Greece	Male	0.40	0.62	0.23
2017	Italy	Both	0.54	0.79	0.37
2017	Italy	Female	0.32	0.48	0.21
2017	Italy	Male	0.78	1.15	0.53
2017	Portugal	Both	0.29	0.48	0.17
2017	Portugal	Female	0.16	0.26	0.09
2017	Portugal	Male	0.44	0.72	0.25
2017	Spain	Both	0.27	0.42	0.17
2017	Spain	Female	0.17	0.26	0.10
2017	Spain	Male	0.38	0.58	0.24
2018	Greece	Both	0.28	0.45	0.17
2018	Greece	Female	0.18	0.29	0.11
2018	Greece	Male	0.40	0.63	0.23
2018	Italy	Both	0.53	0.78	0.36
2018	Italy	Female	0.32	0.46	0.21
2018	Italy	Male	0.76	1.11	0.50
2018	Portugal	Both	0.29	0.47	0.17
2018	Portugal	Female	0.16	0.26	0.09
2018	Portugal	Male	0.44	0.71	0.25
2018	Spain	Both	0.27	0.42	0.17
2018	Spain	Female	0.17	0.26	0.10
2018	Spain	Male	0.38	0.58	0.24
2019	Greece	Both	0.28	0.45	0.17
2019	Greece	Female	0.18	0.29	0.11
2019	Greece	Male	0.39	0.63	0.23
2019	Italy	Both	0.50	0.73	0.33
2019	Italy	Female	0.30	0.44	0.19
2019	Italy	Male	0.72	1.04	0.47
2019	Portugal	Both	0.29	0.47	0.17
2019	Portugal	Female	0.16	0.26	0.09
2019	Portugal	Male	0.44	0.72	0.25
2019	Spain	Both	0.27	0.42	0.17
2019	Spain	Female	0.17	0.26	0.10
2019	Spain	Male	0.38	0.59	0.24

Table S18- Age-standardized rates and 95% uncertainty levels (UL) of **disability-adjusted life years** (DALYs) **due to cirrhosis and other chronic liver diseases due to hepatitis B** per 100,000 population in Greece, Italy, Portugal and Spain from 2000 to 2019 by sex classes (Global Burden of Disease Study 2019).

Year	Country	Sex	DALYs (95% UL)	95% UL (upper)	95% UL (lower)
2000	Greece	Both	24.5	32.7	17.6
2000	Greece	Female	11.5	15.4	8.32
2000	Greece	Male	38.4	51.5	27.4
2000	Italy	Both	43.9	48.2	39.9
2000	Italy	Female	19.6	22.1	17.1
2000	Italy	Male	70.8	77.4	64.6
2000	Portugal	Both	71.1	97.4	50.2
2000	Portugal	Female	30.2	41.5	21.1
2000	Portugal	Male	116.8	160.5	82.9
2000	Spain	Both	47.0	59.4	37.5
2000	Spain	Female	18.4	23.2	14.3
2000	Spain	Male	77.8	98.3	61.9
2001	Greece	Both	24.1	32.3	17.2
2001	Greece	Female	11.0	14.6	7.91
2001	Greece	Male	38.1	51.3	27.0
2001	Italy	Both	42.2	46.4	38.3
2001	Italy	Female	18.6	21.1	16.3
2001	Italy	Male	68.2	74.8	62.3
2001	Portugal	Both	69.2	94.8	48.6
2001	Portugal	Female	28.9	39.7	20.3
2001	Portugal	Male	113.9	156.6	80.4
2001	Spain	Both	44.1	55.8	35.1
2001	Spain	Female	17.1	21.6	13.3
2001	Spain	Male	73.0	92.6	57.6
2002	Greece	Both	23.6	31.6	16.7
2002	Greece	Female	10.7	14.3	7.7
2002	Greece	Male	37.3	50.0	26.3
2002	Italy	Both	40.2	44.2	36.3
2002	Italy	Female	17.7	20.1	15.5
2002	Italy	Male	64.8	71.1	59.0
2002	Portugal	Both	65.8	91.2	46.1
2002	Portugal	Female	27.1	37.2	19.0
2002	Portugal	Male	108.8	150.3	76.1
2002	Spain	Both	39.0	49.4	30.8
2002	Spain	Female	15.1	19.0	11.7
2002	Spain	Male	64.6	82.2	50.9
2003	Greece	Both	23.6	31.7	16.7
2003	Greece	Female	10.3	13.7	7.44
2003	Greece	Male	37.8	50.8	26.4
2003	Italy	Both	38.2	42.3	34.4
2003	Italy	Female	16.8	19.1	14.6
2003	Italy	Male	61.6	67.8	55.8
2003	Portugal	Both	62.4	86.4	43.6
2003	Portugal	Female	25.2	34.8	17.7
2003	Portugal	Male	103.5	143.7	71.7
2003	Spain	Both	33.6	42.6	26.2
2003	Spain	Female	12.9	16.4	10.0

2003	Spain	Male	55.6	70.9	43.4
2004	Greece	Both	23.5	31.6	16.6
2004	Greece	Female	9.9	13.2	7.23
2004	Greece	Male	38.0	51.8	26.3
2004	Italy	Both	35.3	39.2	31.6
2004	Italy	Female	15.4	17.7	13.4
2004	Italy	Male	57.1	63.1	51.5
2004	Portugal	Both	57.4	80.2	40.1
2004	Portugal	Female	22.8	31.7	16.0
2004	Portugal	Male	95.7	132.9	66.4
2004	Spain	Both	28.2	35.9	22.0
2004	Spain	Female	10.7	13.6	8.21
2004	Spain	Male	46.7	59.9	36.0
2005	Greece	Both	23.5	31.7	16.6
2005	Greece	Female	9.6	12.9	6.9
2005	Greece	Male	38.3	52.5	26.6
2005	Italy	Both	33.4	37.3	29.8
2005	Italy	Female	14.4	16.6	12.5
2005	Italy	Male	54.1	60.0	48.4
2005	Portugal	Both	54.2	76.3	37.7
2005	Portugal	Female	20.9	29.4	14.6
2005	Portugal	Male	91.0	127.0	62.6
2005	Spain	Both	25.6	32.6	19.8
2005	Spain	Female	9.6	12.3	7.3
2005	Spain	Male	42.5	54.6	32.7
2006	Greece	Both	22.3	30.1	15.8
2006	Greece	Female	8.9	11.9	6.4
2006	Greece	Male	36.5	50.0	25.4
2006	Italy	Both	31.5	35.2	28.1
2006	Italy	Female	13.4	15.4	11.5
2006	Italy	Male	51.2	56.7	45.7
2006	Portugal	Both	49.6	70.2	34.2
2006	Portugal	Female	18.5	26.0	12.9
2006	Portugal	Male	84.0	118.	57.8
2006	Spain	Both	24.5	31.3	19.0
2006	Spain	Female	9.1	11.6	6.9
2006	Spain	Male	40.7	52.9	31.3
2007	Greece	Both	20.3	27.7	14.4
2007	Greece	Female	7.8	10.5	5.6
2007	Greece	Male	33.6	46.2	23.1
2007	Italy	Both	29.6	33.1	26.4
2007	Italy	Female	12.6	14.4	10.8
2007	Italy	Male	48.1	53.5	42.7
2007	Portugal	Both	44.6	62.7	30.5
2007	Portugal	Female	16.6	23.3	11.6
2007	Portugal	Male	75.6	106.7	51.7
2007	Spain	Both	23.7	30.6	18.4
2007	Spain	Female	8.7	11.1	6.7
2007	Spain	Male	39.4	51.6	30.4
2008	Greece	Both	17.1	23.4	12.0
2008	Greece	Female	6.4	8.80	4.5
2008	Greece	Male	28.4	39.3	19.4
2008	Italy	Both	27.6	30.9	24.4

2008	Italy	Female	11.7	13.4	10.0
2008	Italy	Male	44.8	50.1	39.7
2008	Portugal	Both	39.2	55.4	26.7
2008	Portugal	Female	14.5	20.1	10.1
2008	Portugal	Male	66.5	94.0	45.1
2008	Spain	Both	22.5	29.3	17.4
2008	Spain	Female	8.4	10.6	6.4
2008	Spain	Male	37.3	48.8	28.7
2009	Greece	Both	14.4	20.0	10.0
2009	Greece	Female	5.2	7.16	3.7
2009	Greece	Male	24.2	33.7	16.5
2009	Italy	Both	25.9	29.0	22.8
2009	Italy	Female	11.0	12.7	9.4
2009	Italy	Male	41.9	47.1	36.9
2009	Portugal	Both	35.0	49.7	23.6
2009	Portugal	Female	12.8	17.7	8.9
2009	Portugal	Male	59.6	84.8	39.5
2009	Spain	Both	21.2	27.5	16.5
2009	Spain	Female	7.9	10.0	6.0
2009	Spain	Male	35.2	46.1	27.1
2010	Greece	Both	13.0	18.1	8.9
2010	Greece	Female	4.7	6.4	3.3
2010	Greece	Male	21.7	30.4	14.8
2010	Italy	Both	24.4	27.5	21.4
2010	Italy	Female	10.3	11.9	8.8
2010	Italy	Male	39.6	44.7	34.8
2010	Portugal	Both	31.5	45.1	21.0
2010	Portugal	Female	11.2	15.7	7.7
2010	Portugal	Male	54.1	77.6	35.9
2010	Spain	Both	20.0	26.0	15.6
2010	Spain	Female	7.4	9.41	5.6
2010	Spain	Male	33.3	43.9	25.6
2011	Greece	Both	12.9	18.0	8.9
2011	Greece	Female	4.6	6.40	3.3
2011	Greece	Male	21.7	30.6	14.7
2011	Italy	Both	23.5	26.6	20.6
2011	Italy	Female	10.0	11.5	8.5
2011	Italy	Male	38.1	43.2	33.5
2011	Portugal	Both	29.0	41.8	19.4
2011	Portugal	Female	10.0	14.0	6.9
2011	Portugal	Male	50.0	72.3	33.2
2011	Spain	Both	18.8	24.6	14.6
2011	Spain	Female	7.0	8.84	5.4
2011	Spain	Male	31.3	41.5	24.2
2012	Greece	Both	12.8	17.8	8.8
2012	Greece	Female	4.6	6.42	3.3
2012	Greece	Male	21.4	30.2	14.4
2012	Italy	Both	22.8	25.9	20.0
2012	Italy	Female	9.6	11.1	8.2
2012	Italy	Male	37.0	41.8	32.6
2012	Portugal	Both	26.4	38.1	17.7
2012	Portugal	Female	9.0	12.6	6.1
2012	Portugal	Male	45.9	66.6	30.6

2012	Spain	Both	17.5	23.0	13.6
2012	Spain	Female	6.4	8.21	4.9
2012	Spain	Male	29.3	38.8	22.4
2013	Greece	Both	12.5	17.4	8.55
2013	Greece	Female	4.5	6.29	3.2
2013	Greece	Male	20.9	29.6	13.9
2013	Italy	Both	21.9	25.0	19.3
2013	Italy	Female	9.3	10.8	7.9
2013	Italy	Male	35.5	40.3	31.4
2013	Portugal	Both	24.2	35.1	16.3
2013	Portugal	Female	8.2	11.6	5.6
2013	Portugal	Male	42.1	61.4	28.1
2013	Spain	Both	16.2	21.2	12.4
2013	Spain	Female	6.0	7.65	4.6
2013	Spain	Male	26.9	35.4	20.7
2014	Greece	Both	12.2	17.1	8.31
2014	Greece	Female	4.4	6.17	3.2
2014	Greece	Male	20.5	29.1	13.6
2014	Italy	Both	21.2	24.0	18.6
2014	Italy	Female	8.9	10.3	7.6
2014	Italy	Male	34.4	38.9	30.2
2014	Portugal	Both	22.4	32.7	15.0
2014	Portugal	Female	7.6	10.8	5.2
2014	Portugal	Male	39.0	57.1	25.9
2014	Spain	Both	15.2	19.9	11.6
2014	Spain	Female	5.6	7.21	4.2
2014	Spain	Male	25.3	33.5	19.5
2015	Greece	Both	12.1	17.0	8.17
2015	Greece	Female	4.4	6.10	3.1
2015	Greece	Male	20.4	28.8	13.4
2015	Italy	Both	21.1	24.0	18.7
2015	Italy	Female	9.0	10.4	7.6
2015	Italy	Male	34.2	38.9	30.1
2015	Portugal	Both	20.7	30.3	13.8
2015	Portugal	Female	7.3	10.4	5.0
2015	Portugal	Male	35.8	52.2	23.6
2015	Spain	Both	14.8	19.5	11.3
2015	Spain	Female	5.5	7.07	4.2
2015	Spain	Male	24.6	32.8	18.9
2016	Greece	Both	12.0	17.0	8.19
2016	Greece	Female	4.5	6.11	3.1
2016	Greece	Male	20.0	28.5	13.5
2016	Italy	Both	20.4	23.1	18.0
2016	Italy	Female	8.7	10.0	7.4
2016	Italy	Male	32.9	37.4	29.1
2016	Portugal	Both	20.5	30.0	13.6
2016	Portugal	Female	7.3	10.4	4.9
2016	Portugal	Male	35.4	52.0	23.4
2016	Spain	Both	14.2	18.7	10.9
2016	Spain	Female	5.2	6.78	4.0
2016	Spain	Male	23.7	31.4	18.1
2017	Greece	Both	12.0	17.0	8.28
2017	Greece	Female	4.5	6.19	3.1

2017	Greece	Male	20.0	28.8	13.7
2017	Italy	Both	20.4	23.3	17.9
2017	Italy	Female	8.7	10.1	7.4
2017	Italy	Male	33.0	37.8	28.9
2017	Portugal	Both	20.6	30.4	13.6
2017	Portugal	Female	7.3	10.5	4.9
2017	Portugal	Male	35.6	52.7	23.3
2017	Spain	Both	14.2	18.7	10.8
2017	Spain	Female	5.3	6.80	4.0
2017	Spain	Male	23.7	31.4	17.9
2018	Greece	Both	12.0	16.7	8.28
2018	Greece	Female	4.5	6.25	3.2
2018	Greece	Male	19.9	28.3	13.5
2018	Italy	Both	20.5	23.2	18.0
2018	Italy	Female	8.80	10.2	7.52
2018	Italy	Male	33.0	37.5	29.2
2018	Portugal	Both	20.8	30.3	13.9
2018	Portugal	Female	7.4	10.4	5.0
2018	Portugal	Male	35.8	52.2	23.8
2018	Spain	Both	14.2	18.5	10.9
2018	Spain	Female	5.3	6.89	4.1
2018	Spain	Male	23.5	31.0	18.0
2019	Greece	Both	11.9	16.5	8.29
2019	Greece	Female	4.5	6.31	3.2
2019	Greece	Male	19.8	28.0	13.4
2019	Italy	Both	20.4	23.2	17.9
2019	Italy	Female	8.8	10.3	7.4
2019	Italy	Male	32.9	37.5	28.8
2019	Portugal	Both	20.8	30.0	14.0
2019	Portugal	Female	7.4	10.4	4.9
2019	Portugal	Male	35.9	52.3	24.0
2019	Spain	Both	14.1	18.5	10.8
2019	Spain	Female	5.4	6.89	4.1
2019	Spain	Male	23.3	30.7	17.9

Table S19- Age-standardized rates and 95% uncertainty levels (UL) of **prevalence of cirrhosis and other chronic liver diseases due to hepatitis C** per 100,000 population in Greece, Italy, Portugal and Spain from 2000 to 2019 by sex classes (Global Burden of Disease Study 2019).

Year	Country	Sex	Prevalence (95% UL)	95% UL (upper)	95% UL (lower)
2000	Greece	Both	310.8	375.6	253.6
2000	Greece	Female	320.2	395.7	254.6
2000	Greece	Male	299.7	356.6	249.3
2000	Italy	Both	1750.3	2122.2	1432.0
2000	Italy	Female	1739.6	2107.7	1421.7
2000	Italy	Male	1740.2	2108.1	1419.9
2000	Portugal	Both	734.3	890.7	601.2
2000	Portugal	Female	685.2	833.1	553.2
2000	Portugal	Male	783.9	960.4	640.2
2000	Spain	Both	721.2	838.3	614.3
2000	Spain	Female	644.0	751.5	541.2
2000	Spain	Male	801.2	942.2	677.1
2001	Greece	Both	320.1	386.8	262.8
2001	Greece	Female	330.0	407.1	265.0
2001	Greece	Male	308.4	367.4	256.5
2001	Italy	Both	1710.2	2071.0	1399.8
2001	Italy	Female	1697.0	2057.7	1387.9
2001	Italy	Male	1703.4	2070.2	1392.6
2001	Portugal	Both	728.4	885.7	598.5
2001	Portugal	Female	679.8	825.1	556.4
2001	Portugal	Male	777.6	954.5	637.1
2001	Spain	Both	698.0	807.5	597.0
2001	Spain	Female	622.8	725.5	528.0
2001	Spain	Male	775.1	901.8	658.8
2002	Greece	Both	343.7	417.7	281.6
2002	Greece	Female	354.7	437.2	287.7
2002	Greece	Male	330.7	398.4	274.0
2002	Italy	Both	1633.4	1977.6	1339.5
2002	Italy	Female	1621.4	1963.6	1325.5
2002	Italy	Male	1625.9	1976.8	1333.4
2002	Portugal	Both	722.6	878.4	593.6
2002	Portugal	Female	674.1	820.7	549.6
2002	Portugal	Male	771.9	947.2	634.8
2002	Spain	Both	665.8	769.3	567.9
2002	Spain	Female	596.8	693.6	504.5
2002	Spain	Male	735.6	855.4	629.3
2003	Greece	Both	372.1	457.0	304.8
2003	Greece	Female	384.5	474.4	312.2
2003	Greece	Male	357.6	436.0	295.5
2003	Italy	Both	1544.5	1869.9	1270.1
2003	Italy	Female	1535.1	1856.6	1254.1
2003	Italy	Male	1534.6	1865.7	1261.1
2003	Portugal	Both	717.0	872.6	590.3
2003	Portugal	Female	668.6	812.6	542.5
2003	Portugal	Male	766.5	936.8	631.1
2003	Spain	Both	632.4	731.8	538.2

2003	Spain	Female	571.2	664.9	484.8
2003	Spain	Male	693.3	806.4	592.0
2004	Greece	Both	396.0	490.5	322.7
2004	Greece	Female	409.5	509.8	332.1
2004	Greece	Male	380.3	470.1	311.1
2004	Italy	Both	1467.9	1778.4	1206.3
2004	Italy	Female	1460.9	1770.2	1190.7
2004	Italy	Male	1456.2	1771.5	1198.1
2004	Portugal	Both	711.8	870.9	583.8
2004	Portugal	Female	663.7	811.3	535.7
2004	Portugal	Male	761.3	936.1	623.8
2004	Spain	Both	605.6	703.3	514.5
2004	Spain	Female	551.3	643.4	466.0
2004	Spain	Male	658.9	771.0	559.9
2005	Greece	Both	406.0	511.9	330.4
2005	Greece	Female	420.0	527.9	336.7
2005	Greece	Male	389.8	488.8	313.3
2005	Italy	Both	1428.0	1731.7	1174.5
2005	Italy	Female	1421.1	1725.9	1158.4
2005	Italy	Male	1417.7	1725.9	1164.6
2005	Portugal	Both	707.1	869.6	575.9
2005	Portugal	Female	659.6	810.1	528.8
2005	Portugal	Male	756.1	934.6	618.3
2005	Spain	Both	593.2	691.9	503.2
2005	Spain	Female	542.3	634.5	456.3
2005	Spain	Male	642.9	757.4	544.0
2006	Greece	Both	405.6	509.8	330.1
2006	Greece	Female	419.5	526.3	337.1
2006	Greece	Male	389.5	484.0	315.8
2006	Italy	Both	1418.7	1723.6	1162.9
2006	Italy	Female	1411.2	1719.7	1150.4
2006	Italy	Male	1411.2	1720.4	1156.9
2006	Portugal	Both	702.5	864.3	574.6
2006	Portugal	Female	655.7	803.1	527.2
2006	Portugal	Male	750.7	927.1	616.4
2006	Spain	Both	605.7	708.6	512.0
2006	Spain	Female	555.8	652.5	464.5
2006	Spain	Male	654.1	768.6	551.0
2007	Greece	Both	404.8	506.2	330.2
2007	Greece	Female	418.5	527.9	338.4
2007	Greece	Male	389.0	482.5	316.9
2007	Italy	Both	1415.3	1722.8	1154.6
2007	Italy	Female	1408.8	1718.2	1146.1
2007	Italy	Male	1409.0	1723.3	1152.8
2007	Portugal	Both	697.5	856.7	569.8
2007	Portugal	Female	651.5	795.3	526.2
2007	Portugal	Male	745.0	919.4	610.9
2007	Spain	Both	638.8	755.1	536.3
2007	Spain	Female	590.7	700.8	488.4
2007	Spain	Male	685.2	811.1	574.5
2008	Greece	Both	403.8	502.8	329.1
2008	Greece	Female	417.2	526.7	337.8
2008	Greece	Male	388.1	479.7	315.1

2008	Italy	Both	1413.9	1726.3	1152.2
2008	Italy	Female	1409.1	1721.4	1146.2
2008	Italy	Male	1408.1	1726.1	1151.9
2008	Portugal	Both	692.4	849.2	563.5
2008	Portugal	Female	647.3	792.7	521.4
2008	Portugal	Male	739.2	911.7	603.6
2008	Spain	Both	678.6	810.0	562.1
2008	Spain	Female	632.6	758.4	518.9
2008	Spain	Male	722.7	865.8	601.0
2009	Greece	Both	402.6	499.5	327.0
2009	Greece	Female	416.0	521.9	337.0
2009	Greece	Male	387.1	479.8	314.4
2009	Italy	Both	1410.7	1727.8	1148.1
2009	Italy	Female	1407.3	1723.3	1145.4
2009	Italy	Male	1405.5	1724.5	1147.6
2009	Portugal	Both	687.5	844.0	559.1
2009	Portugal	Female	643.3	792.4	517.1
2009	Portugal	Male	733.4	904.1	593.6
2009	Spain	Both	711.4	861.1	581.9
2009	Spain	Female	667.3	815.3	540.8
2009	Spain	Male	753.3	917.6	621.8
2010	Greece	Both	401.6	495.5	326.8
2010	Greece	Female	415.1	522.0	337.3
2010	Greece	Male	385.9	477.1	313.7
2010	Italy	Both	1401.9	1720.4	1140.9
2010	Italy	Female	1398.8	1718.3	1136.4
2010	Italy	Male	1398.0	1718.0	1142.0
2010	Portugal	Both	683.1	840.2	553.4
2010	Portugal	Female	640.0	790.1	513.0
2010	Portugal	Male	727.7	894.9	584.9
2010	Spain	Both	723.1	889.1	583.6
2010	Spain	Female	680.6	843.2	543.0
2010	Spain	Male	763.6	944.9	621.2
2011	Greece	Both	400.3	496.3	326.0
2011	Greece	Female	414.3	519.2	337.2
2011	Greece	Male	384.2	472.9	312.5
2011	Italy	Both	1371.4	1684.4	1114.4
2011	Italy	Female	1371.3	1684.3	1111.4
2011	Italy	Male	1365.4	1677.8	1115.0
2011	Portugal	Both	678.3	835.1	552.2
2011	Portugal	Female	636.9	783.2	515.5
2011	Portugal	Male	721.2	886.2	584.2
2011	Spain	Both	717.0	878.6	580.4
2011	Spain	Female	676.3	839.6	541.1
2011	Spain	Male	755.7	932.4	616.6
2012	Greece	Both	398.8	491.5	325.0
2012	Greece	Female	413.5	517.2	336.7
2012	Greece	Male	381.7	472.2	309.9
2012	Italy	Both	1317.3	1618.0	1070.7
2012	Italy	Female	1325.1	1628.1	1071.5
2012	Italy	Male	1303.3	1600.8	1065.7
2012	Portugal	Both	672.7	831.5	549.0
2012	Portugal	Female	633.3	781.0	514.4

2012	Portugal	Male	713.3	871.9	578.5
2012	Spain	Both	706.9	869.0	574.2
2012	Spain	Female	669.1	831.2	539.5
2012	Spain	Male	742.7	918.1	606.5
2013	Greece	Both	397.2	491.8	324.1
2013	Greece	Female	412.9	513.2	335.3
2013	Greece	Male	379.0	471.4	307.5
2013	Italy	Both	1256.8	1544.2	1022.5
2013	Italy	Female	1274.3	1572.7	1028.8
2013	Italy	Male	1232.8	1517.7	1007.5
2013	Portugal	Both	667.0	821.4	545.8
2013	Portugal	Female	629.8	774.6	513.2
2013	Portugal	Male	705.2	863.3	570.9
2013	Spain	Both	695.4	853.8	567.8
2013	Spain	Female	660.9	823.3	532.8
2013	Spain	Male	728.0	899.4	595.1
2014	Greece	Both	395.9	492.4	323.4
2014	Greece	Female	412.5	514.3	333.6
2014	Greece	Male	376.6	469.0	304.4
2014	Italy	Both	1207.4	1483.6	982.6
2014	Italy	Female	1233.2	1521.8	997.3
2014	Italy	Male	1174.6	1442.1	959.3
2014	Portugal	Both	661.9	812.4	541.0
2014	Portugal	Female	626.6	772.6	507.1
2014	Portugal	Male	697.7	856.1	564.1
2014	Spain	Both	685.1	846.8	557.9
2014	Spain	Female	653.5	824.0	526.4
2014	Spain	Male	714.7	880.5	581.6
2015	Greece	Both	395.1	493.9	322.1
2015	Greece	Female	412.4	515.1	332.2
2015	Greece	Male	375.2	469.5	301.7
2015	Italy	Both	1186.4	1458.3	964.2
2015	Italy	Female	1215.9	1499.7	982.4
2015	Italy	Male	1149.8	1408.7	937.9
2015	Portugal	Both	657.9	805.4	538.1
2015	Portugal	Female	624.3	773.2	502.2
2015	Portugal	Male	691.9	852.8	556.9
2015	Spain	Both	678.4	843.8	550.2
2015	Spain	Female	648.9	822.8	519.2
2015	Spain	Male	706.2	881.4	572.8
2016	Greece	Both	399.2	493.1	324.5
2016	Greece	Female	414.9	511.6	336.2
2016	Greece	Male	381.2	472.8	309.5
2016	Italy	Both	1199.9	1472.7	974.0
2016	Italy	Female	1230.6	1524.7	994.9
2016	Italy	Male	1163.2	1430.0	947.3
2016	Portugal	Both	657.9	809.1	540.1
2016	Portugal	Female	622.8	774.0	504.6
2016	Portugal	Male	694.1	851.7	565.5
2016	Spain	Both	677.5	836.7	551.7
2016	Spain	Female	648.7	806.6	524.1
2016	Spain	Male	704.9	865.8	573.1
2017	Greece	Both	403.7	494.3	325.0

2017	Greece	Female	417.6	514.2	335.0
2017	Greece	Male	387.7	476.5	312.6
2017	Italy	Both	1216.5	1493.3	985.9
2017	Italy	Female	1248.0	1550.5	1008.5
2017	Italy	Male	1180.0	1448.5	960.4
2017	Portugal	Both	659.4	811.7	538.3
2017	Portugal	Female	622.1	779.8	505.2
2017	Portugal	Male	698.4	860.7	568.9
2017	Spain	Both	678.4	829.5	548.6
2017	Spain	Female	649.7	804.0	524.9
2017	Spain	Male	706.1	869.3	565.9
2018	Greece	Both	403.7	493.7	329.8
2018	Greece	Female	417.9	515.8	337.7
2018	Greece	Male	387.3	471.6	315.5
2018	Italy	Both	1218.3	1497.4	986.5
2018	Italy	Female	1251.2	1549.7	1007.0
2018	Italy	Male	1180.3	1451.1	960.1
2018	Portugal	Both	658.7	808.5	540.2
2018	Portugal	Female	621.6	770.7	506.5
2018	Portugal	Male	697.4	851.7	569.8
2018	Spain	Both	676.9	826.6	552.5
2018	Spain	Female	648.5	805.0	523.4
2018	Spain	Male	704.5	855.5	572.5
2019	Greece	Both	402.2	494.7	328.8
2019	Greece	Female	417.7	516.4	335.4
2019	Greece	Male	384.2	472.6	312.4
2019	Italy	Both	1214.1	1497.7	981.9
2019	Italy	Female	1250.5	1547.7	1008.5
2019	Italy	Male	1172.0	1444.0	951.64
2019	Portugal	Both	656.2	806.9	536.2
2019	Portugal	Female	620.0	766.8	501.4
2019	Portugal	Male	693.8	850.9	564.0
2019	Spain	Both	672.9	825.9	547.2
2019	Spain	Female	645.3	795.6	517.1
2019	Spain	Male	699.7	851.3	569.1

Table S20- Age-standardized rates and 95% uncertainty levels (UL) of **incidence of cirrhosis and other chronic liver diseases due to hepatitis C** per 100,000 population in Greece, Italy, Portugal and Spain from 2000 to 2019 by sex classes (Global Burden of Disease Study 2019).

Year	Country	Sex	Incidence (95% UL)	95% UL (upper)	95% UL (lower)
2000	Greece	Both	3.5	5.2	2.2
2000	Greece	Female	2.5	3.8	1.5
2000	Greece	Male	4.4	6.6	2.8
2000	Italy	Both	17.2	20.9	13.6
2000	Italy	Female	10.9	13.6	8.62
2000	Italy	Male	23.4	28.6	18.5
2000	Portugal	Both	6.5	9.5	4.2
2000	Portugal	Female	3.6	5.3	2.2
2000	Portugal	Male	9.6	14.0	6.2
2000	Spain	Both	9.4	12.4	6.6
2000	Spain	Female	6.7	9.1	4.8
2000	Spain	Male	12.1	16.2	8.42
2001	Greece	Both	3.4	5.1	2.1
2001	Greece	Female	2.4	3.7	1.5
2001	Greece	Male	4.3	6.5	2.7
2001	Italy	Both	17.0	20.6	13.5
2001	Italy	Female	10.8	13.4	8.51
2001	Italy	Male	23.1	28.2	18.4
2001	Portugal	Both	6.4	9.2	4.1
2001	Portugal	Female	3.5	5.1	2.2
2001	Portugal	Male	9.4	13.6	6.1
2001	Spain	Both	9.3	12.3	6.6
2001	Spain	Female	6.6	9.0	4.7
2001	Spain	Male	11.9	16.0	8.31
2002	Greece	Both	3.3	4.9	2.1
2002	Greece	Female	2.4	3.5	1.5
2002	Greece	Male	4.2	6.4	2.7
2002	Italy	Both	16.7	20.3	13.4
2002	Italy	Female	10.6	13.2	8.43
2002	Italy	Male	22.8	27.6	18.3
2002	Portugal	Both	6.2	9.0	4.0
2002	Portugal	Female	3.4	5.0	2.1
2002	Portugal	Male	9.2	13.3	5.9
2002	Spain	Both	9.2	12.2	6.5
2002	Spain	Female	6.6	8.9	4.7
2002	Spain	Male	11.7	15.8	8.31
2003	Greece	Both	3.2	4.8	2.0
2003	Greece	Female	2.3	3.4	1.4
2003	Greece	Male	4.1	6.2	2.6
2003	Italy	Both	16.4	19.9	13.2
2003	Italy	Female	10.5	13.0	8.37
2003	Italy	Male	22.4	27.0	18.1
2003	Portugal	Both	6.1	8.8	3.9
2003	Portugal	Female	3.3	4.9	2.0
2003	Portugal	Male	9.0	13.0	5.7
2003	Spain	Both	9.1	12.2	6.4

2003	Spain	Female	6.5	8.7	4.6
2003	Spain	Male	11.6	15.6	8.30
2004	Greece	Both	3.1	4.7	2.0
2004	Greece	Female	2.2	3.3	1.4
2004	Greece	Male	4.0	6.1	2.6
2004	Italy	Both	16.1	19.5	13.0
2004	Italy	Female	10.3	12.7	8.27
2004	Italy	Male	21.9	26.3	17.8
2004	Portugal	Both	5.9	8.7	3.7
2004	Portugal	Female	3.2	4.8	2.0
2004	Portugal	Male	8.8	12.7	5.5
2004	Spain	Both	9.0	12.0	6.4
2004	Spain	Female	6.4	8.6	4.5
2004	Spain	Male	11.5	15.5	8.22
2005	Greece	Both	3.1	4.6	2.0
2005	Greece	Female	2.2	3.2	1.3
2005	Greece	Male	4.0	6.0	2.5
2005	Italy	Both	15.8	19.1	12.7
2005	Italy	Female	10.1	12.4	8.11
2005	Italy	Male	21.5	25.8	17.5
2005	Portugal	Both	5.8	8.5	3.6
2005	Portugal	Female	3.2	4.7	1.9
2005	Portugal	Male	8.6	12.5	5.3
2005	Spain	Both	8.9	12.0	6.3
2005	Spain	Female	6.3	8.5	4.4
2005	Spain	Male	11.4	15.4	8.15
2006	Greece	Both	3.1	4.6	2.0
2006	Greece	Female	2.1	3.2	1.3
2006	Greece	Male	4.0	5.9	2.5
2006	Italy	Both	15.5	18.6	12.5
2006	Italy	Female	9.8	12.1	7.8
2006	Italy	Male	21.0	25.3	17.2
2006	Portugal	Both	5.6	8.3	3.5
2006	Portugal	Female	3.0	4.6	1.8
2006	Portugal	Male	8.4	12.4	5.2
2006	Spain	Both	8.8	11.8	6.2
2006	Spain	Female	6.2	8.3	4.4
2006	Spain	Male	11.3	15.1	7.94
2007	Greece	Both	3.1	4.6	2.0
2007	Greece	Female	2.1	3.1	1.3
2007	Greece	Male	4.0	6.0	2.6
2007	Italy	Both	15.1	18.1	12.2
2007	Italy	Female	9.5	11.7	7.6
2007	Italy	Male	20.5	24.7	16.8
2007	Portugal	Both	5.5	8.1	3.4
2007	Portugal	Female	2.9	4.4	1.8
2007	Portugal	Male	8.1	12.1	5.1
2007	Spain	Both	8.6	11.5	6.0
2007	Spain	Female	6.1	8.2	4.3
2007	Spain	Male	11.0	14.6	7.77
2008	Greece	Both	3.1	4.6	2.0
2008	Greece	Female	2.1	3.1	1.3
2008	Greece	Male	4.1	6.1	2.6

2008	Italy	Both	14.6	17.6	11.8
2008	Italy	Female	9.2	11.3	7.3
2008	Italy	Male	20.0	24.1	16.3
2008	Portugal	Both	5.3	7.8	3.3
2008	Portugal	Female	2.8	4.2	1.7
2008	Portugal	Male	7.9	11.7	4.9
2008	Spain	Both	8.4	11.1	5.9
2008	Spain	Female	6.0	8.0	4.2
2008	Spain	Male	10.7	14.1	7.53
2009	Greece	Both	3.1	4.7	2.0
2009	Greece	Female	2.1	3.2	1.3
2009	Greece	Male	4.2	6.2	2.7
2009	Italy	Both	14.2	17.1	11.5
2009	Italy	Female	8.9	10.9	7.1
2009	Italy	Male	19.5	23.4	15.9
2009	Portugal	Both	5.1	7.6	3.2
2009	Portugal	Female	2.6	3.9	1.6
2009	Portugal	Male	7.6	11.4	4.7
2009	Spain	Both	8.1	10.9	5.7
2009	Spain	Female	5.8	7.8	4.1
2009	Spain	Male	10.3	13.7	7.24
2010	Greece	Both	3.1	4.6	2.0
2010	Greece	Female	2.1	3.2	1.3
2010	Greece	Male	4.2	6.2	2.7
2010	Italy	Both	13.9	16.8	11.3
2010	Italy	Female	8.6	10.6	6.9
2010	Italy	Male	19.1	23.1	15.5
2010	Portugal	Both	4.9	7.3	3.1
2010	Portugal	Female	2.6	3.8	1.6
2010	Portugal	Male	7.4	11.1	4.6
2010	Spain	Both	8.0	10.6	5.5
2010	Spain	Female	5.7	7.7	4.0
2010	Spain	Male	10.1	13.4	7.03
2011	Greece	Both	3.1	4.6	2.0
2011	Greece	Female	2.1	3.1	1.3
2011	Greece	Male	4.2	6.2	2.7
2011	Italy	Both	13.6	16.4	11.0
2011	Italy	Female	8.4	10.4	6.7
2011	Italy	Male	18.8	22.7	15.1
2011	Portugal	Both	4.8	7.2	3.1
2011	Portugal	Female	2.5	3.7	1.6
2011	Portugal	Male	7.3	10.9	4.5
2011	Spain	Both	7.8	10.4	5.5
2011	Spain	Female	5.6	7.5	3.9
2011	Spain	Male	9.9	13.2	7.0
2012	Greece	Both	3.1	4.6	2.0
2012	Greece	Female	2.1	3.1	1.3
2012	Greece	Male	4.1	6.1	2.6
2012	Italy	Both	13.3	16.1	10.7
2012	Italy	Female	8.2	10.1	6.5
2012	Italy	Male	18.4	22.2	14.7
2012	Portugal	Both	4.8	7.0	3.0
2012	Portugal	Female	2.5	3.7	1.6

2012	Portugal	Male	7.2	10.7	4.4
2012	Spain	Both	7.7	10.2	5.4
2012	Spain	Female	5.5	7.4	3.8
2012	Spain	Male	9.8	13.0	6.9
2013	Greece	Both	3.1	4.5	2.0
2013	Greece	Female	2.0	3.0	1.3
2013	Greece	Male	4.1	6.1	2.6
2013	Italy	Both	13.0	15.8	10.5
2013	Italy	Female	8.0	9.9	6.3
2013	Italy	Male	18.0	21.8	14.4
2013	Portugal	Both	4.7	6.9	2.9
2013	Portugal	Female	2.4	3.7	1.5
2013	Portugal	Male	7.1	10.5	4.4
2013	Spain	Both	7.6	10.1	5.3
2013	Spain	Female	5.3	7.2	3.8
2013	Spain	Male	9.7	12.9	6.7
2014	Greece	Both	3.0	4.4	1.9
2014	Greece	Female	2.0	3.0	1.3
2014	Greece	Male	4.0	6.0	2.6
2014	Italy	Both	12.8	15.6	10.3
2014	Italy	Female	7.9	9.8	6.2
2014	Italy	Male	17.7	21.5	14.1
2014	Portugal	Both	4.6	6.8	2.9
2014	Portugal	Female	2.4	3.6	1.5
2014	Portugal	Male	6.9	10.3	4.3
2014	Spain	Both	7.4	10.0	5.2
2014	Spain	Female	5.2	7.0	3.7
2014	Spain	Male	9.6	12.8	6.6
2015	Greece	Both	3.0	4.4	1.9
2015	Greece	Female	2.0	3.0	1.2
2015	Greece	Male	4.0	6.0	2.6
2015	Italy	Both	12.7	15.4	10.2
2015	Italy	Female	7.8	9.7	6.1
2015	Italy	Male	17.6	21.4	14.0
2015	Portugal	Both	4.5	6.6	2.8
2015	Portugal	Female	2.4	3.5	1.5
2015	Portugal	Male	6.7	9.9	4.2
2015	Spain	Both	7.3	9.8	5.1
2015	Spain	Female	5.1	6.9	3.6
2015	Spain	Male	9.4	12.7	6.5
2016	Greece	Both	2.9	4.4	1.9
2016	Greece	Female	2.0	2.9	1.2
2016	Greece	Male	3.9	5.9	2.5
2016	Italy	Both	12.8	15.6	10.2
2016	Italy	Female	7.9	9.8	6.1
2016	Italy	Male	17.7	21.6	14.0
2016	Portugal	Both	4.2	6.2	2.6
2016	Portugal	Female	2.2	3.3	1.4
2016	Portugal	Male	6.2	9.2	3.9
2016	Spain	Both	7.0	9.3	4.8
2016	Spain	Female	4.9	6.6	3.4
2016	Spain	Male	9.0	12.1	6.2
2017	Greece	Both	2.9	4.3	1.9

2017	Greece	Female	2.0	2.9	1.2
2017	Greece	Male	3.8	5.8	2.4
2017	Italy	Both	12.9	15.8	10.1
2017	Italy	Female	7.9	10.0	6.1
2017	Italy	Male	17.8	21.9	14.0
2017	Portugal	Both	3.9	5.8	2.4
2017	Portugal	Female	2.0	3.1	1.3
2017	Portugal	Male	5.8	8.7	3.6
2017	Spain	Both	6.7	9.0	4.6
2017	Spain	Female	4.7	6.5	3.3
2017	Spain	Male	8.5	11.5	5.9
2018	Greece	Both	2.9	4.3	1.8
2018	Greece	Female	2.0	3.0	1.2
2018	Greece	Male	3.8	5.7	2.4
2018	Italy	Both	12.6	15.6	9.81
2018	Italy	Female	7.7	9.8	5.9
2018	Italy	Male	17.4	21.6	13.6
2018	Portugal	Both	3.9	5.8	2.4
2018	Portugal	Female	2.0	3.1	1.3
2018	Portugal	Male	5.8	8.6	3.6
2018	Spain	Both	6.7	8.9	4.6
2018	Spain	Female	4.7	6.5	3.3
2018	Spain	Male	8.5	11.5	5.8
2019	Greece	Both	2.9	4.3	1.8
2019	Greece	Female	2.0	3.0	1.2
2019	Greece	Male	3.8	5.8	2.4
2019	Italy	Both	12.0	15.0	9.30
2019	Italy	Female	7.3	9.5	5.5
2019	Italy	Male	16.6	21.0	12.6
2019	Portugal	Both	3.9	5.8	2.4
2019	Portugal	Female	2.1	3.1	1.3
2019	Portugal	Male	5.8	8.7	3.6
2019	Spain	Both	6.7	9.1	4.6
2019	Spain	Female	4.8	6.5	3.3
2019	Spain	Male	8.6	11.8	5.9

Table S21- Age-standardized rates and 95% uncertainty levels (UL) of **deaths due to cirrhosis and other chronic liver diseases due to hepatitis C** per 100,000 population in Greece, Italy, Portugal and Spain from 2000 to 2019 by sex classes (Global Burden of Disease Study 2019).

Year	Country	Sex	Deaths (95% UL)	95% UL (upper)	95% UL (lower)
2000	Greece	Both	1.32	1.78	0.95
2000	Greece	Female	0.82	1.13	0.56
2000	Greece	Male	1.87	2.56	1.33
2000	Italy	Both	4.79	5.34	4.31
2000	Italy	Female	2.91	3.33	2.51
2000	Italy	Male	7.07	7.82	6.41
2000	Portugal	Both	3.24	4.36	2.37
2000	Portugal	Female	1.55	2.06	1.13
2000	Portugal	Male	5.26	7.17	3.81
2000	Spain	Both	3.65	4.71	2.81
2000	Spain	Female	1.97	2.55	1.46
2000	Spain	Male	5.60	7.22	4.28
2001	Greece	Both	1.27	1.71	0.92
2001	Greece	Female	0.78	1.07	0.54
2001	Greece	Male	1.82	2.49	1.30
2001	Italy	Both	4.61	5.15	4.14
2001	Italy	Female	2.78	3.20	2.39
2001	Italy	Male	6.82	7.54	6.19
2001	Portugal	Both	3.15	4.27	2.30
2001	Portugal	Female	1.48	1.96	1.08
2001	Portugal	Male	5.13	6.97	3.71
2001	Spain	Both	3.57	4.58	2.76
2001	Spain	Female	1.90	2.46	1.41
2001	Spain	Male	5.48	7.05	4.17
2002	Greece	Both	1.23	1.65	0.89
2002	Greece	Female	0.75	1.03	0.53
2002	Greece	Male	1.75	2.38	1.26
2002	Italy	Both	4.43	4.97	3.98
2002	Italy	Female	2.69	3.09	2.31
2002	Italy	Male	6.53	7.21	5.90
2002	Portugal	Both	3.04	4.10	2.22
2002	Portugal	Female	1.41	1.88	1.04
2002	Portugal	Male	4.96	6.77	3.58
2002	Spain	Both	3.54	4.54	2.75
2002	Spain	Female	1.88	2.42	1.40
2002	Spain	Male	5.44	6.99	4.11
2003	Greece	Both	1.21	1.62	0.88
2003	Greece	Female	0.72	0.99	0.51
2003	Greece	Male	1.74	2.37	1.25
2003	Italy	Both	4.28	4.81	3.81
2003	Italy	Female	2.59	2.99	2.21
2003	Italy	Male	6.31	7.02	5.70
2003	Portugal	Both	2.94	3.99	2.15
2003	Portugal	Female	1.36	1.82	1.00
2003	Portugal	Male	4.81	6.57	3.47
2003	Spain	Both	3.56	4.54	2.73

2003	Spain	Female	1.88	2.44	1.39
2003	Spain	Male	5.47	7.00	4.14
2004	Greece	Both	1.18	1.58	0.85
2004	Greece	Female	0.69	0.93	0.49
2004	Greece	Male	1.71	2.32	1.24
2004	Italy	Both	4.01	4.52	3.55
2004	Italy	Female	2.41	2.81	2.03
2004	Italy	Male	5.93	6.59	5.31
2004	Portugal	Both	2.79	3.81	2.03
2004	Portugal	Female	1.26	1.69	0.93
2004	Portugal	Male	4.57	6.26	3.32
2004	Spain	Both	3.47	4.43	2.66
2004	Spain	Female	1.81	2.35	1.35
2004	Spain	Male	5.35	6.87	4.02
2005	Greece	Both	1.15	1.55	0.83
2005	Greece	Female	0.66	0.90	0.47
2005	Greece	Male	1.69	2.30	1.23
2005	Italy	Both	3.88	4.38	3.43
2005	Italy	Female	2.32	2.71	1.95
2005	Italy	Male	5.74	6.41	5.17
2005	Portugal	Both	2.70	3.69	1.98
2005	Portugal	Female	1.20	1.61	0.87
2005	Portugal	Male	4.46	6.12	3.23
2005	Spain	Both	3.40	4.35	2.59
2005	Spain	Female	1.75	2.30	1.31
2005	Spain	Male	5.26	6.80	3.90
2006	Greece	Both	1.13	1.52	0.82
2006	Greece	Female	0.64	0.88	0.45
2006	Greece	Male	1.67	2.27	1.21
2006	Italy	Both	3.75	4.25	3.31
2006	Italy	Female	2.23	2.62	1.87
2006	Italy	Male	5.56	6.21	4.98
2006	Portugal	Both	2.60	3.54	1.91
2006	Portugal	Female	1.12	1.49	0.82
2006	Portugal	Male	4.33	5.94	3.17
2006	Spain	Both	3.26	4.16	2.49
2006	Spain	Female	1.66	2.16	1.24
2006	Spain	Male	5.05	6.54	3.75
2007	Greece	Both	1.15	1.55	0.83
2007	Greece	Female	0.62	0.87	0.44
2007	Greece	Male	1.72	2.35	1.24
2007	Italy	Both	3.68	4.18	3.24
2007	Italy	Female	2.19	2.58	1.84
2007	Italy	Male	5.44	6.07	4.87
2007	Portugal	Both	2.54	3.47	1.88
2007	Portugal	Female	1.10	1.46	0.81
2007	Portugal	Male	4.22	5.82	3.09
2007	Spain	Both	3.17	4.07	2.42
2007	Spain	Female	1.61	2.09	1.20
2007	Spain	Male	4.91	6.38	3.65
2008	Greece	Both	1.13	1.53	0.83
2008	Greece	Female	0.60	0.84	0.42
2008	Greece	Male	1.71	2.34	1.22

2008	Italy	Both	3.62	4.14	3.19
2008	Italy	Female	2.16	2.56	1.80
2008	Italy	Male	5.34	5.99	4.77
2008	Portugal	Both	2.45	3.35	1.81
2008	Portugal	Female	1.05	1.41	0.77
2008	Portugal	Male	4.08	5.60	3.00
2008	Spain	Both	3.04	3.92	2.33
2008	Spain	Female	1.56	2.03	1.15
2008	Spain	Male	4.68	6.05	3.49
2009	Greece	Both	1.13	1.52	0.82
2009	Greece	Female	0.58	0.80	0.41
2009	Greece	Male	1.73	2.36	1.23
2009	Italy	Both	3.55	4.04	3.12
2009	Italy	Female	2.12	2.50	1.76
2009	Italy	Male	5.22	5.88	4.65
2009	Portugal	Both	2.39	3.26	1.76
2009	Portugal	Female	1.02	1.36	0.75
2009	Portugal	Male	3.98	5.47	2.91
2009	Spain	Both	2.89	3.73	2.21
2009	Spain	Female	1.48	1.91	1.09
2009	Spain	Male	4.47	5.79	3.34
2010	Greece	Both	1.10	1.49	0.80
2010	Greece	Female	0.56	0.78	0.39
2010	Greece	Male	1.69	2.31	1.21
2010	Italy	Both	3.40	3.88	2.98
2010	Italy	Female	2.02	2.41	1.69
2010	Italy	Male	5.01	5.66	4.45
2010	Portugal	Both	2.30	3.12	1.69
2010	Portugal	Female	0.96	1.27	0.71
2010	Portugal	Male	3.86	5.31	2.83
2010	Spain	Both	2.75	3.56	2.10
2010	Spain	Female	1.39	1.81	1.02
2010	Spain	Male	4.26	5.55	3.22
2011	Greece	Both	1.10	1.49	0.79
2011	Greece	Female	0.55	0.77	0.39
2011	Greece	Male	1.70	2.34	1.22
2011	Italy	Both	3.31	3.77	2.89
2011	Italy	Female	1.97	2.34	1.64
2011	Italy	Male	4.87	5.48	4.34
2011	Portugal	Both	2.21	3.00	1.61
2011	Portugal	Female	0.91	1.20	0.66
2011	Portugal	Male	3.72	5.14	2.72
2011	Spain	Both	2.64	3.42	2.02
2011	Spain	Female	1.34	1.74	0.99
2011	Spain	Male	4.09	5.32	3.11
2012	Greece	Both	1.10	1.50	0.79
2012	Greece	Female	0.55	0.76	0.38
2012	Greece	Male	1.70	2.33	1.21
2012	Italy	Both	3.22	3.66	2.81
2012	Italy	Female	1.92	2.27	1.59
2012	Italy	Male	4.73	5.30	4.19
2012	Portugal	Both	2.11	2.87	1.54
2012	Portugal	Female	0.85	1.14	0.63

2012	Portugal	Male	3.57	4.92	2.61
2012	Spain	Both	2.55	3.28	1.94
2012	Spain	Female	1.28	1.66	0.94
2012	Spain	Male	3.96	5.15	2.99
2013	Greece	Both	1.07	1.45	0.77
2013	Greece	Female	0.53	0.73	0.37
2013	Greece	Male	1.66	2.30	1.18
2013	Italy	Both	3.10	3.52	2.71
2013	Italy	Female	1.84	2.18	1.52
2013	Italy	Male	4.54	5.13	4.02
2013	Portugal	Both	2.01	2.75	1.46
2013	Portugal	Female	0.81	1.09	0.60
2013	Portugal	Male	3.41	4.72	2.48
2013	Spain	Both	2.44	3.14	1.86
2013	Spain	Female	1.23	1.61	0.90
2013	Spain	Male	3.79	4.94	2.86
2014	Greece	Both	1.05	1.43	0.76
2014	Greece	Female	0.51	0.71	0.36
2014	Greece	Male	1.64	2.26	1.17
2014	Italy	Both	3.00	3.42	2.61
2014	Italy	Female	1.78	2.11	1.47
2014	Italy	Male	4.41	4.96	3.91
2014	Portugal	Both	1.91	2.62	1.40
2014	Portugal	Female	0.77	1.03	0.56
2014	Portugal	Male	3.25	4.53	2.35
2014	Spain	Both	2.38	3.08	1.81
2014	Spain	Female	1.20	1.56	0.87
2014	Spain	Male	3.70	4.86	2.80
2015	Greece	Both	1.05	1.43	0.76
2015	Greece	Female	0.51	0.70	0.36
2015	Greece	Male	1.64	2.26	1.16
2015	Italy	Both	3.01	3.43	2.63
2015	Italy	Female	1.80	2.13	1.49
2015	Italy	Male	4.40	4.97	3.89
2015	Portugal	Both	1.82	2.50	1.30
2015	Portugal	Female	0.75	1.01	0.54
2015	Portugal	Male	3.07	4.30	2.20
2015	Spain	Both	2.37	3.09	1.82
2015	Spain	Female	1.20	1.57	0.87
2015	Spain	Male	3.69	4.86	2.77
2016	Greece	Both	1.03	1.40	0.75
2016	Greece	Female	0.51	0.70	0.36
2016	Greece	Male	1.61	2.22	1.15
2016	Italy	Both	2.90	3.30	2.54
2016	Italy	Female	1.73	2.06	1.44
2016	Italy	Male	4.23	4.76	3.75
2016	Portugal	Both	1.82	2.51	1.30
2016	Portugal	Female	0.76	1.02	0.55
2016	Portugal	Male	3.06	4.27	2.18
2016	Spain	Both	2.30	2.98	1.77
2016	Spain	Female	1.15	1.51	0.84
2016	Spain	Male	3.58	4.70	2.70
2017	Greece	Both	1.03	1.41	0.75

2017	Greece	Female	0.51	0.70	0.35
2017	Greece	Male	1.60	2.21	1.14
2017	Italy	Both	2.89	3.30	2.53
2017	Italy	Female	1.72	2.05	1.43
2017	Italy	Male	4.23	4.79	3.75
2017	Portugal	Both	1.82	2.51	1.29
2017	Portugal	Female	0.77	1.03	0.54
2017	Portugal	Male	3.07	4.27	2.17
2017	Spain	Both	2.30	2.98	1.76
2017	Spain	Female	1.16	1.51	0.84
2017	Spain	Male	3.58	4.69	2.70
2018	Greece	Both	1.04	1.42	0.76
2018	Greece	Female	0.53	0.73	0.37
2018	Greece	Male	1.60	2.20	1.15
2018	Italy	Both	2.91	3.30	2.55
2018	Italy	Female	1.74	2.08	1.46
2018	Italy	Male	4.23	4.80	3.74
2018	Portugal	Both	1.82	2.52	1.32
2018	Portugal	Female	0.77	1.01	0.55
2018	Portugal	Male	3.06	4.24	2.20
2018	Spain	Both	2.27	2.92	1.75
2018	Spain	Female	1.17	1.54	0.85
2018	Spain	Male	3.51	4.56	2.65
2019	Greece	Both	1.05	1.43	0.77
2019	Greece	Female	0.54	0.75	0.37
2019	Greece	Male	1.61	2.20	1.15
2019	Italy	Both	2.91	3.31	2.53
2019	Italy	Female	1.75	2.07	1.45
2019	Italy	Male	4.22	4.78	3.73
2019	Portugal	Both	1.82	2.52	1.31
2019	Portugal	Female	0.76	1.02	0.54
2019	Portugal	Male	3.06	4.27	2.20
2019	Spain	Both	2.25	2.89	1.72
2019	Spain	Female	1.16	1.53	0.84
2019	Spain	Male	3.46	4.48	2.62

Table S22- Age-standardized rates and 95% uncertainty levels (UL) of **years of life lost** (YLLs) **due to cirrhosis and other chronic liver diseases due to hepatitis C** per 100,000 population in Greece, Italy, Portugal and Spain from 2000 to 2019 by sex classes (Global Burden of Disease Study 2019).

Year	Country	Sex	YLLs (95% UL)	95% UL (upper)	95% UL (lower)
2002	Greece	Male	45.3	62.1	32.8
2002	Greece	Female	14.3	19.3	10.5
2002	Greece	Both	29.3	39.9	21.6
2002	Italy	Male	173.2	189.8	158.2
2002	Italy	Female	59.9	68.0	52.6
2002	Italy	Both	113.9	125.5	103.7
2003	Greece	Male	45.6	63.0	32.9
2003	Greece	Female	13.7	18.5	10.1
2003	Greece	Both	29.1	39.9	21.3
2001	Italy	Male	181.7	198.6	166.7
2001	Italy	Female	62.7	70.9	55.3
2001	Italy	Both	119.3	131.3	108.6
2000	Greece	Male	47.2	64.4	34.0
2000	Greece	Female	15.6	21.1	11.4
2000	Greece	Both	30.9	41.6	22.7
2004	Greece	Male	45.6	62.8	32.7
2004	Greece	Female	13.3	17.9	9.73
2004	Greece	Both	28.9	39.5	21.1
2001	Greece	Male	46.6	63.8	33.8
2001	Greece	Female	14.8	20.0	10.9
2001	Greece	Both	30.2	40.8	22.3
2000	Portugal	Male	164.8	224.0	118.5
2000	Portugal	Female	46.4	62.6	33.1
2000	Portugal	Both	102.4	138.3	73.76
2002	Spain	Male	153.6	195.5	116.6
2002	Spain	Female	43.2	54.9	33.8
2002	Spain	Both	96.7	123.	74.3
2000	Spain	Male	157.7	200.2	122.1
2000	Spain	Female	45.2	57.5	34.9
2000	Spain	Both	99.5	126.	76.9
2001	Portugal	Male	161.4	220.4	114.6
2001	Portugal	Female	44.7	60.8	31.9
2001	Portugal	Both	99.9	135.	71.1
2002	Portugal	Male	156.0	212.2	110.4
2002	Portugal	Female	42.5	57.5	30.6
2002	Portugal	Both	96.3	130.	68.2
2000	Italy	Male	188.0	205.4	172.9
2000	Italy	Female	65.9	74.8	58.3
2000	Italy	Both	123.9	136.3	113.0
2003	Spain	Male	154.4	196.5	117.5
2003	Spain	Female	43.0	55.0	33.7
2003	Spain	Both	97.1	122.	74.6
2001	Spain	Male	155.0	197.3	118.9
2001	Spain	Female	43.9	55.5	34.1
2001	Spain	Both	97.7	124.	75.2
2003	Italy	Male	166.1	182.7	151.3

2003	Italy	Female	57.0	64.8	49.8
2003	Italy	Both	109.0	120.6	98.6
2004	Portugal	Male	144.2	197.7	103.0
2004	Portugal	Female	37.7	51.1	27.2
2004	Portugal	Both	88.2	120.	62.8
2003	Portugal	Male	151.7	206.5	107.5
2003	Portugal	Female	40.6	55.0	29.2
2003	Portugal	Both	93.2	126.	66.1
2005	Greece	Male	45.9	63.2	32.7
2005	Greece	Female	12.8	17.2	9.36
2005	Greece	Both	28.8	39.3	20.9
2006	Italy	Male	145.9	162.5	131.2
2006	Italy	Female	47.8	55.3	41.2
2006	Italy	Both	94.8	106.	84.9
2006	Greece	Male	46.0	63.0	32.7
2006	Greece	Female	12.4	16.7	9.10
2006	Greece	Both	28.7	39.0	20.8
2004	Italy	Male	155.6	172.4	141.0
2004	Italy	Female	52.6	60.4	45.8
2004	Italy	Both	101.8	113.2	91.7
2006	Portugal	Male	137.1	188.9	97.6
2006	Portugal	Female	33.2	45.4	24.0
2006	Portugal	Both	82.5	112.	59.3
2007	Spain	Male	138.3	178.9	103.3
2007	Spain	Female	36.6	46.4	28.8
2007	Spain	Both	86.3	110.	65.1
2005	Italy	Male	150.	166.	135.
2005	Italy	Female	50.2	57.7	43.5
2005	Italy	Both	98.0	109.	87.8
2005	Portugal	Male	141.2	194.7	100.5
2005	Portugal	Female	35.6	48.5	25.6
2005	Portugal	Both	85.8	117.	61.2
2009	Portugal	Male	125.2	171.7	89.9
2009	Portugal	Female	29.8	40.7	21.3
2009	Portugal	Both	75.0	102.	54.0
2008	Italy	Male	139.9	156.9	125.9
2008	Italy	Female	45.4	52.6	39.1
2008	Italy	Both	90.8	102.	80.8
2007	Greece	Male	48.1	65.7	34.1
2007	Greece	Female	12.5	16.6	9.19
2007	Greece	Both	29.8	40.7	21.3
2008	Portugal	Male	128.2	176.5	92.3
2008	Portugal	Female	30.8	42.2	22.1
2008	Portugal	Both	77.0	105.	55.5
2005	Spain	Male	148.0	189.0	110.6
2005	Spain	Female	39.9	50.7	31.5
2005	Spain	Both	92.5	117.	70.6
2008	Spain	Male	131.3	170.3	97.7
2008	Spain	Female	35.4	45.1	27.9
2008	Spain	Both	82.2	105.	62.1
2007	Portugal	Male	133.3	184.7	95.4
2007	Portugal	Female	32.2	43.9	23.1
2007	Portugal	Both	80.2	110.	57.8

2009	Greece	Male	49.4	67.9	35.1
2009	Greece	Female	12.0	16.0	8.91
2009	Greece	Both	30.2	41.4	21.6
2009	Spain	Male	124.5	160.8	93.1
2009	Spain	Female	33.3	42.5	26.1
2009	Spain	Both	77.8	100.	59.4
2009	Italy	Male	136.2	152.5	121.9
2009	Italy	Female	44.3	51.5	38.0
2009	Italy	Both	88.5	99.6	78.7
2007	Italy	Male	142.7	158.7	128.3
2007	Italy	Female	46.7	54.1	40.3
2007	Italy	Both	92.8	104.	82.8
2010	Portugal	Male	121.1	166.9	87.3
2010	Portugal	Female	27.9	37.8	19.9
2010	Portugal	Both	72.1	98.8	52.1
2008	Greece	Male	48.5	66.2	34.6
2008	Greece	Female	12.3	16.5	9.08
2008	Greece	Both	29.9	40.9	21.5
2012	Greece	Male	48.5	67.3	34.7
2012	Greece	Female	11.8	15.8	8.57
2012	Greece	Both	29.6	40.8	21.4
2010	Greece	Male	48.5	67.2	34.6
2010	Greece	Female	11.8	15.7	8.78
2010	Greece	Both	29.6	40.7	21.3
2013	Spain	Male	102.6	134.8	77.1
2013	Spain	Female	27.1	34.6	21.0
2013	Spain	Both	63.8	83.0	48.4
2006	Spain	Male	142.4	182.8	105.7
2006	Spain	Female	37.9	48.0	29.9
2006	Spain	Both	88.9	113.	67.2
2010	Spain	Male	118.1	153.9	87.9
2010	Spain	Female	31.2	40.0	24.4
2010	Spain	Both	73.7	95.7	55.9
2004	Spain	Male	151.0	192.4	113.6
2004	Spain	Female	41.3	52.4	32.4
2004	Spain	Both	94.7	120.	72.4
2010	Italy	Male	130.8	147.1	117.0
2010	Italy	Female	42.1	48.8	36.0
2010	Italy	Both	84.8	95.6	75.1
2011	Portugal	Male	116.7	160.6	83.8
2011	Portugal	Female	25.9	35.2	18.5
2011	Portugal	Both	68.9	94.6	49.6
2011	Greece	Male	48.8	68.1	35.1
2011	Greece	Female	11.7	15.6	8.65
2011	Greece	Both	29.7	41.1	21.5
2012	Italy	Male	122.7	137.7	109.5
2012	Italy	Female	39.7	46.2	33.8
2012	Italy	Both	79.6	89.8	70.5
2011	Italy	Male	126.9	142.2	113.4
2011	Italy	Female	41.0	47.6	35.0
2011	Italy	Both	82.3	92.8	73.1
2015	Spain	Male	98.1	129.	73.7
2015	Spain	Female	25.9	33.0	19.8

2015	Spain	Both	61.0	79.8	46.9
2012	Portugal	Male	111.1	153.4	79.2
2012	Portugal	Female	24.0	32.5	17.2
2012	Portugal	Both	65.2	89.6	46.7
2015	Greece	Male	46.7	65.6	33.0
2015	Greece	Female	11.1	15.1	8.08
2015	Greece	Both	28.3	39.4	20.2
2018	Greece	Male	45.4	62.4	32.2
2018	Greece	Female	11.6	15.9	8.28
2018	Greece	Both	28.0	38.2	20.2
2013	Greece	Male	47.6	66.2	33.9
2013	Greece	Female	11.4	15.4	8.34
2013	Greece	Both	29.0	40.3	20.9
2014	Greece	Male	46.9	65.9	33.1
2014	Greece	Female	11.2	15.1	8.08
2014	Greece	Both	28.5	39.4	20.4
2015	Portugal	Male	92.6	130.	65.0
2015	Portugal	Female	20.6	28.5	14.9
2015	Portugal	Both	54.6	76.5	38.8
2016	Italy	Male	109.3	122.8	97.4
2016	Italy	Female	35.9	41.8	30.7
2016	Italy	Both	71.3	80.5	63.2
2014	Spain	Male	99.2	130.	74.1
2014	Spain	Female	26.0	33.2	20.1
2014	Spain	Both	61.6	80.2	46.7
2014	Portugal	Male	99.8	139.	70.5
2014	Portugal	Female	21.3	29.2	15.5
2014	Portugal	Both	58.3	81.3	41.4
2016	Portugal	Male	91.8	129.	64.2
2016	Portugal	Female	20.7	28.8	14.9
2016	Portugal	Both	54.2	76.1	38.1
2013	Italy	Male	118.1	132.5	104.6
2013	Italy	Female	38.2	44.4	32.7
2013	Italy	Both	76.6	86.5	67.7
2014	Italy	Male	114.5	128.6	101.8
2014	Italy	Female	36.6	42.4	31.2
2014	Italy	Both	74.1	83.6	65.5
2017	Italy	Male	109.2	123.0	97.0
2017	Italy	Female	35.9	41.8	30.8
2017	Italy	Both	71.2	80.6	63.1
2011	Spain	Male	112.7	146.8	84.2
2011	Spain	Female	30.0	38.4	23.3
2011	Spain	Both	70.3	91.3	53.5
2013	Portugal	Male	105.3	146.9	75.1
2013	Portugal	Female	22.6	30.5	16.2
2013	Portugal	Both	61.7	85.3	44.1
2018	Portugal	Male	93.0	129.	65.8
2018	Portugal	Female	21.1	29.2	14.9
2018	Portugal	Both	55.0	76.3	39.2
2017	Spain	Male	95.2	125.	71.0
2017	Spain	Female	25.2	32.2	19.4
2017	Spain	Both	59.2	77.6	44.8
2017	Greece	Male	45.4	63.0	32.1

2017	Greece	Female	11.4	15.6	8.14
2017	Greece	Both	27.8	38.3	20.0
2019	Italy	Male	109.1	122.9	96.9
2019	Italy	Female	36.2	42.3	30.8
2019	Italy	Both	71.4	80.8	63.3
2017	Portugal	Male	92.1	130.	64.3
2017	Portugal	Female	21.0	29.0	14.9
2017	Portugal	Both	54.5	76.0	38.3
2015	Italy	Male	113.8	127.8	101.4
2015	Italy	Female	37.2	43.2	31.7
2015	Italy	Both	74.1	83.7	65.6
2019	Greece	Male	45.6	62.8	32.5
2019	Greece	Female	11.6	15.9	8.19
2019	Greece	Both	28.1	38.3	20.5
2012	Spain	Male	108.1	140.4	81.2
2012	Spain	Female	28.3	36.0	22.0
2012	Spain	Both	67.2	86.9	51.2
2016	Greece	Male	45.6	63.6	32.3
2016	Greece	Female	11.3	15.4	8.15
2016	Greece	Both	27.9	38.5	20.1
2018	Italy	Male	109.4	123.6	97.5
2018	Italy	Female	36.2	42.3	31.0
2018	Italy	Both	71.5	81.0	63.6
2016	Spain	Male	95.0	125.	71.1
2016	Spain	Female	24.9	31.7	19.1
2016	Spain	Both	59.0	77.3	45.0
2019	Portugal	Male	93.5	131.	66.2
2019	Portugal	Female	21.0	29.2	15.0
2019	Portugal	Both	55.1	77.5	39.5
2018	Spain	Male	94.0	123.	70.6
2018	Spain	Female	25.4	32.7	19.3
2018	Spain	Both	58.8	76.2	44.4
2019	Spain	Male	92.8	121.	70.0
2019	Spain	Female	25.2	32.4	19.1
2019	Spain	Both	58.2	75.5	43.9

Table S23- Age-standardized rates and 95% uncertainty levels (UL) of **years lived with disability** (YLDs) **due to cirrhosis and other chronic liver diseases due to hepatitis C** per 100,000 population in Greece, Italy, Portugal and Spain from 2000 to 2019 by sex classes (Global Burden of Disease Study 2019).

Year	Country	Sex	YLDs (95% UL)	95% UL (upper)	95% UL (lower)
2000	Greece	Both	0.60	0.96	0.35
2000	Greece	Female	0.43	0.67	0.26
2000	Greece	Male	0.78	1.29	0.42
2000	Italy	Both	2.77	3.99	1.88
2000	Italy	Female	1.81	2.65	1.21
2000	Italy	Male	3.80	5.44	2.57
2000	Portugal	Both	1.45	2.35	0.84
2000	Portugal	Female	0.89	1.47	0.47
2000	Portugal	Male	2.08	3.41	1.15
2000	Spain	Both	1.75	2.67	1.11
2000	Spain	Female	1.13	1.78	0.67
2000	Spain	Male	2.41	3.81	1.42
2001	Greece	Both	0.59	0.93	0.35
2001	Greece	Female	0.42	0.66	0.25
2001	Greece	Male	0.77	1.26	0.44
2001	Italy	Both	2.74	3.90	1.85
2001	Italy	Female	1.80	2.59	1.23
2001	Italy	Male	3.76	5.33	2.54
2001	Portugal	Both	1.43	2.31	0.83
2001	Portugal	Female	0.86	1.44	0.44
2001	Portugal	Male	2.05	3.37	1.13
2001	Spain	Both	1.73	2.61	1.07
2001	Spain	Female	1.10	1.71	0.65
2001	Spain	Male	2.38	3.72	1.42
2002	Greece	Both	0.58	0.94	0.35
2002	Greece	Female	0.41	0.66	0.24
2002	Greece	Male	0.77	1.25	0.42
2002	Italy	Both	2.71	3.89	1.86
2002	Italy	Female	1.78	2.59	1.21
2002	Italy	Male	3.72	5.29	2.53
2002	Portugal	Both	1.41	2.25	0.81
2002	Portugal	Female	0.85	1.46	0.44
2002	Portugal	Male	2.02	3.24	1.10
2002	Spain	Both	1.69	2.61	1.05
2002	Spain	Female	1.09	1.72	0.66
2002	Spain	Male	2.32	3.60	1.33
2003	Greece	Both	0.58	0.93	0.33
2003	Greece	Female	0.40	0.63	0.24
2003	Greece	Male	0.76	1.28	0.40
2003	Italy	Both	2.68	3.80	1.82
2003	Italy	Female	1.77	2.56	1.20
2003	Italy	Male	3.67	5.22	2.50
2003	Portugal	Both	1.38	2.21	0.79
2003	Portugal	Female	0.83	1.39	0.43
2003	Portugal	Male	2.00	3.16	1.13
2003	Spain	Both	1.66	2.48	1.02

2003	Spain	Female	1.06	1.69	0.62
2003	Spain	Male	2.28	3.48	1.39
2004	Greece	Both	0.58	0.92	0.32
2004	Greece	Female	0.40	0.62	0.24
2004	Greece	Male	0.76	1.26	0.38
2004	Italy	Both	2.66	3.78	1.83
2004	Italy	Female	1.75	2.57	1.19
2004	Italy	Male	3.63	5.21	2.49
2004	Portugal	Both	1.34	2.13	0.77
2004	Portugal	Female	0.80	1.33	0.40
2004	Portugal	Male	1.94	3.16	1.06
2004	Spain	Both	1.63	2.48	1.00
2004	Spain	Female	1.05	1.65	0.62
2004	Spain	Male	2.23	3.45	1.32
2005	Greece	Both	0.57	0.93	0.33
2005	Greece	Female	0.40	0.62	0.24
2005	Greece	Male	0.76	1.27	0.39
2005	Italy	Both	2.62	3.74	1.80
2005	Italy	Female	1.73	2.47	1.16
2005	Italy	Male	3.58	5.08	2.45
2005	Portugal	Both	1.33	2.10	0.77
2005	Portugal	Female	0.80	1.36	0.43
2005	Portugal	Male	1.91	3.10	1.07
2005	Spain	Both	1.61	2.45	0.99
2005	Spain	Female	1.03	1.64	0.63
2005	Spain	Male	2.20	3.48	1.31
2006	Greece	Both	0.57	0.92	0.33
2006	Greece	Female	0.39	0.62	0.24
2006	Greece	Male	0.76	1.27	0.41
2006	Italy	Both	2.58	3.68	1.76
2006	Italy	Female	1.70	2.42	1.13
2006	Italy	Male	3.53	5.03	2.41
2006	Portugal	Both	1.30	2.10	0.73
2006	Portugal	Female	0.79	1.31	0.44
2006	Portugal	Male	1.86	3.04	0.99
2006	Spain	Both	1.57	2.43	0.95
2006	Spain	Female	1.01	1.61	0.59
2006	Spain	Male	2.15	3.37	1.24
2007	Greece	Both	0.59	0.94	0.33
2007	Greece	Female	0.40	0.62	0.24
2007	Greece	Male	0.78	1.32	0.40
2007	Italy	Both	2.53	3.63	1.69
2007	Italy	Female	1.66	2.41	1.11
2007	Italy	Male	3.46	4.94	2.33
2007	Portugal	Both	1.26	2.02	0.70
2007	Portugal	Female	0.76	1.27	0.41
2007	Portugal	Male	1.80	2.92	0.96
2007	Spain	Both	1.54	2.35	0.96
2007	Spain	Female	0.98	1.55	0.57
2007	Spain	Male	2.11	3.34	1.25
2008	Greece	Both	0.59	0.96	0.35
2008	Greece	Female	0.40	0.63	0.24
2008	Greece	Male	0.79	1.32	0.43

2008	Italy	Both	2.47	3.59	1.68
2008	Italy	Female	1.62	2.37	1.09
2008	Italy	Male	3.39	4.89	2.28
2008	Portugal	Both	1.21	1.92	0.69
2008	Portugal	Female	0.73	1.19	0.42
2008	Portugal	Male	1.74	2.85	0.94
2008	Spain	Both	1.51	2.29	0.93
2008	Spain	Female	0.97	1.54	0.56
2008	Spain	Male	2.06	3.20	1.22
2009	Greece	Both	0.60	0.98	0.35
2009	Greece	Female	0.41	0.63	0.24
2009	Greece	Male	0.80	1.37	0.44
2009	Italy	Both	2.42	3.49	1.62
2009	Italy	Female	1.58	2.29	1.06
2009	Italy	Male	3.32	4.75	2.23
2009	Portugal	Both	1.16	1.94	0.67
2009	Portugal	Female	0.70	1.17	0.40
2009	Portugal	Male	1.66	2.82	0.90
2009	Spain	Both	1.47	2.24	0.89
2009	Spain	Female	0.95	1.48	0.53
2009	Spain	Male	2.01	3.13	1.18
2010	Greece	Both	0.60	0.99	0.35
2010	Greece	Female	0.41	0.63	0.24
2010	Greece	Male	0.81	1.37	0.42
2010	Italy	Both	2.36	3.45	1.61
2010	Italy	Female	1.53	2.23	1.05
2010	Italy	Male	3.26	4.75	2.22
2010	Portugal	Both	1.12	1.83	0.65
2010	Portugal	Female	0.67	1.09	0.40
2010	Portugal	Male	1.61	2.68	0.88
2010	Spain	Both	1.45	2.17	0.91
2010	Spain	Female	0.94	1.47	0.55
2010	Spain	Male	1.97	3.02	1.16
2011	Greece	Both	0.60	0.97	0.35
2011	Greece	Female	0.40	0.63	0.24
2011	Greece	Male	0.81	1.35	0.42
2011	Italy	Both	2.31	3.30	1.57
2011	Italy	Female	1.50	2.18	1.01
2011	Italy	Male	3.18	4.61	2.13
2011	Portugal	Both	1.09	1.78	0.62
2011	Portugal	Female	0.66	1.03	0.38
2011	Portugal	Male	1.58	2.65	0.82
2011	Spain	Both	1.41	2.12	0.86
2011	Spain	Female	0.91	1.42	0.52
2011	Spain	Male	1.92	2.97	1.11
2012	Greece	Both	0.60	0.96	0.34
2012	Greece	Female	0.40	0.63	0.24
2012	Greece	Male	0.81	1.30	0.43
2012	Italy	Both	2.23	3.22	1.53
2012	Italy	Female	1.45	2.12	0.96
2012	Italy	Male	3.08	4.44	2.09
2012	Portugal	Both	1.07	1.70	0.59
2012	Portugal	Female	0.65	1.02	0.38

2012	Portugal	Male	1.54	2.50	0.82
2012	Spain	Both	1.38	2.15	0.85
2012	Spain	Female	0.90	1.44	0.54
2012	Spain	Male	1.87	3.00	1.09
2013	Greece	Both	0.59	0.96	0.34
2013	Greece	Female	0.40	0.62	0.24
2013	Greece	Male	0.80	1.34	0.41
2013	Italy	Both	2.16	3.13	1.46
2013	Italy	Female	1.40	2.04	0.94
2013	Italy	Male	2.98	4.32	2.00
2013	Portugal	Both	1.05	1.67	0.60
2013	Portugal	Female	0.63	0.99	0.38
2013	Portugal	Male	1.52	2.52	0.80
2013	Spain	Both	1.35	2.05	0.82
2013	Spain	Female	0.87	1.37	0.52
2013	Spain	Male	1.84	2.95	1.08
2014	Greece	Both	0.59	0.91	0.33
2014	Greece	Female	0.39	0.62	0.24
2014	Greece	Male	0.80	1.29	0.43
2014	Italy	Both	2.11	3.03	1.43
2014	Italy	Female	1.36	1.98	0.92
2014	Italy	Male	2.90	4.17	1.94
2014	Portugal	Both	1.03	1.62	0.59
2014	Portugal	Female	0.61	0.96	0.36
2014	Portugal	Male	1.50	2.44	0.80
2014	Spain	Both	1.31	1.99	0.78
2014	Spain	Female	0.85	1.34	0.51
2014	Spain	Male	1.79	2.83	1.04
2015	Greece	Both	0.58	0.95	0.32
2015	Greece	Female	0.39	0.61	0.23
2015	Greece	Male	0.79	1.34	0.41
2015	Italy	Both	2.09	3.03	1.42
2015	Italy	Female	1.35	1.97	0.89
2015	Italy	Male	2.88	4.17	1.96
2015	Portugal	Both	1.00	1.58	0.58
2015	Portugal	Female	0.59	0.93	0.35
2015	Portugal	Male	1.45	2.38	0.78
2015	Spain	Both	1.29	1.97	0.78
2015	Spain	Female	0.84	1.32	0.51
2015	Spain	Male	1.75	2.77	1.02
2016	Greece	Both	0.59	0.95	0.34
2016	Greece	Female	0.40	0.63	0.24
2016	Greece	Male	0.79	1.33	0.43
2016	Italy	Both	2.12	3.09	1.41
2016	Italy	Female	1.37	2.01	0.92
2016	Italy	Male	2.92	4.27	1.92
2016	Portugal	Both	0.95	1.53	0.56
2016	Portugal	Female	0.57	0.90	0.34
2016	Portugal	Male	1.37	2.26	0.75
2016	Spain	Both	1.26	1.89	0.75
2016	Spain	Female	0.85	1.28	0.53
2016	Spain	Male	1.68	2.64	0.96
2017	Greece	Both	0.60	0.97	0.34

2017	Greece	Female	0.42	0.67	0.25
2017	Greece	Male	0.79	1.35	0.42
2017	Italy	Both	2.15	3.16	1.45
2017	Italy	Female	1.39	2.01	0.93
2017	Italy	Male	2.96	4.32	1.98
2017	Portugal	Both	0.91	1.45	0.53
2017	Portugal	Female	0.56	0.89	0.33
2017	Portugal	Male	1.30	2.15	0.68
2017	Spain	Both	1.25	1.93	0.78
2017	Spain	Female	0.86	1.33	0.52
2017	Spain	Male	1.65	2.61	0.98
2018	Greece	Both	0.60	0.96	0.34
2018	Greece	Female	0.42	0.67	0.25
2018	Greece	Male	0.79	1.30	0.42
2018	Italy	Both	2.10	3.07	1.41
2018	Italy	Female	1.36	2.00	0.91
2018	Italy	Male	2.88	4.21	1.94
2018	Portugal	Both	0.91	1.50	0.51
2018	Portugal	Female	0.55	0.88	0.33
2018	Portugal	Male	1.30	2.22	0.67
2018	Spain	Both	1.24	1.88	0.76
2018	Spain	Female	0.86	1.33	0.53
2018	Spain	Male	1.64	2.58	0.94
2019	Greece	Both	0.60	0.94	0.34
2019	Greece	Female	0.42	0.66	0.25
2019	Greece	Male	0.79	1.29	0.42
2019	Italy	Both	1.97	2.87	1.31
2019	Italy	Female	1.28	1.87	0.84
2019	Italy	Male	2.70	3.95	1.78
2019	Portugal	Both	0.91	1.49	0.53
2019	Portugal	Female	0.55	0.86	0.33
2019	Portugal	Male	1.30	2.20	0.72
2019	Spain	Both	1.24	1.86	0.76
2019	Spain	Female	0.86	1.30	0.53
2019	Spain	Male	1.64	2.58	0.93

Table S24- Age-standardized rates and 95% uncertainty levels (UL) of **disability-adjusted life years** (DALYs) **due to cirrhosis and other chronic liver diseases due to hepatitis C** per 100,000 population in Greece, Italy, Portugal and Spain from 2000 to 2019 by sex classes (Global Burden of Disease Study 2019).

Year	Country	Sex	DALYs (95% UL)	95% UL (upper)	95% UL (lower)
2000	Greece	Both	31.5	42.4	23.2
2000	Greece	Female	16.1	21.5	11.9
2000	Greece	Male	48.0	65.3	34.6
2000	Italy	Both	126.7	138.8	115.9
2000	Italy	Female	67.7	76.4	60.1
2000	Italy	Male	191.9	209.4	176.2
2000	Portugal	Both	103.8	140.7	75.12
2000	Portugal	Female	47.3	63.6	34.0
2000	Portugal	Male	166.9	227.1	120.9
2000	Spain	Both	101.2	128.5	78.26
2000	Spain	Female	46.3	58.4	35.9
2000	Spain	Male	160.1	203.1	124.1
2001	Greece	Both	30.8	41.4	22.9
2001	Greece	Female	15.2	20.4	11.3
2001	Greece	Male	47.4	64.5	34.4
2001	Italy	Both	122.0	133.9	111.5
2001	Italy	Female	64.5	72.6	56.9
2001	Italy	Male	185.4	202.4	170.5
2001	Portugal	Both	101.3	137.0	72.87
2001	Portugal	Female	45.5	61.4	32.7
2001	Portugal	Male	163.4	222.8	117.3
2001	Spain	Both	99.4	126.	76.5
2001	Spain	Female	45.0	56.5	35.2
2001	Spain	Male	157.4	199.8	121.0
2002	Greece	Both	29.9	40.6	22.1
2002	Greece	Female	14.7	19.8	11.0
2002	Greece	Male	46.1	63.1	33.4
2002	Italy	Both	116.6	127.9	106.4
2002	Italy	Female	61.7	69.6	54.3
2002	Italy	Male	177.0	193.4	162.3
2002	Portugal	Both	97.7	131.	69.8
2002	Portugal	Female	43.3	58.3	31.6
2002	Portugal	Male	158.0	215.3	112.0
2002	Spain	Both	98.4	124.	75.5
2002	Spain	Female	44.3	55.9	34.8
2002	Spain	Male	155.9	197.7	119.0
2003	Greece	Both	29.7	40.6	21.9
2003	Greece	Female	14.2	18.9	10.5
2003	Greece	Male	46.3	63.6	33.6
2003	Italy	Both	111.7	123.1	101.1
2003	Italy	Female	58.7	66.6	51.5
2003	Italy	Male	169.8	185.6	155.1
2003	Portugal	Both	94.6	127.	67.5
2003	Portugal	Female	41.4	55.9	30.0
2003	Portugal	Male	153.7	208.4	108.9
2003	Spain	Both	98.8	124.	76.0

2003	Spain	Female	44.1	55.8	34.9
2003	Spain	Male	156.7	199.0	119.2
2004	Greece	Both	29.5	40.2	21.5
2004	Greece	Female	13.7	18.3	10.1
2004	Greece	Male	46.4	63.5	33.5
2004	Italy	Both	104.4	116.1	94.40
2004	Italy	Female	54.4	62.3	47.4
2004	Italy	Male	159.3	175.8	144.8
2004	Portugal	Both	89.6	121.	64.2
2004	Portugal	Female	38.5	52.0	28.0
2004	Portugal	Male	146.1	199.7	104.2
2004	Spain	Both	96.3	121.	73.7
2004	Spain	Female	42.4	53.6	33.4
2004	Spain	Male	153.2	194.3	115.1
2005	Greece	Both	29.4	39.9	21.6
2005	Greece	Female	13.2	17.6	9.80
2005	Greece	Male	46.7	63.9	33.4
2005	Italy	Both	100.6	112.1	90.53
2005	Italy	Female	52.0	59.5	45.2
2005	Italy	Male	153.8	169.9	138.7
2005	Portugal	Both	87.1	117.	62.3
2005	Portugal	Female	36.4	49.3	26.3
2005	Portugal	Male	143.2	196.3	102.1
2005	Spain	Both	94.2	119.	72.2
2005	Spain	Female	40.9	51.8	32.4
2005	Spain	Male	150.2	191.2	113.0
2006	Greece	Both	29.3	39.6	21.4
2006	Greece	Female	12.8	17.1	9.50
2006	Greece	Male	46.7	63.8	33.5
2006	Italy	Both	97.4	108.	87.7
2006	Italy	Female	49.5	57.1	42.9
2006	Italy	Male	149.5	165.2	135.3
2006	Portugal	Both	83.8	114.	60.6
2006	Portugal	Female	34.0	46.3	24.7
2006	Portugal	Male	138.9	190.3	99.59
2006	Spain	Both	90.5	114.	68.7
2006	Spain	Female	38.9	49.0	30.8
2006	Spain	Male	144.6	185.2	107.7
2007	Greece	Both	30.4	41.2	22.0
2007	Greece	Female	12.9	17.1	9.63
2007	Greece	Male	48.8	66.5	34.9
2007	Italy	Both	95.3	107.	85.4
2007	Italy	Female	48.4	55.8	42.0
2007	Italy	Male	146.2	162.2	131.8
2007	Portugal	Both	81.5	111.	59.0
2007	Portugal	Female	33.0	44.9	23.8
2007	Portugal	Male	135.1	186.7	97.07
2007	Spain	Both	87.8	112.	66.8
2007	Spain	Female	37.6	47.6	29.9
2007	Spain	Male	140.4	181.4	105.1
2008	Greece	Both	30.5	41.6	22.1
2008	Greece	Female	12.7	16.9	9.53
2008	Greece	Male	49.3	67.1	35.3

2008	Italy	Both	93.3	104.	83.5
2008	Italy	Female	47.0	54.3	40.5
2008	Italy	Male	143.3	160.3	128.9
2008	Portugal	Both	78.2	106.	56.5
2008	Portugal	Female	31.5	43.1	22.7
2008	Portugal	Male	129.9	178.4	94.11
2008	Spain	Both	83.7	107.	63.7
2008	Spain	Female	36.4	46.2	28.7
2008	Spain	Male	133.4	172.1	99.98
2009	Greece	Both	30.8	42.1	22.3
2009	Greece	Female	12.4	16.4	9.30
2009	Greece	Male	50.2	68.7	35.7
2009	Italy	Both	90.9	101.	81.1
2009	Italy	Female	45.9	53.2	39.4
2009	Italy	Male	139.5	155.6	124.8
2009	Portugal	Both	76.2	103.	55.0
2009	Portugal	Female	30.5	41.3	22.1
2009	Portugal	Male	126.9	173.2	91.70
2009	Spain	Both	79.3	102.	60.4
2009	Spain	Female	34.3	43.7	27.0
2009	Spain	Male	126.5	163.0	95.40
2010	Greece	Both	30.2	41.2	21.8
2010	Greece	Female	12.2	16.1	9.10
2010	Greece	Male	49.3	68.2	35.4
2010	Italy	Both	87.1	98.3	77.4
2010	Italy	Female	43.6	50.5	37.4
2010	Italy	Male	134.1	150.0	119.9
2010	Portugal	Both	73.2	99.8	52.9
2010	Portugal	Female	28.6	38.8	20.6
2010	Portugal	Male	122.7	168.0	88.80
2010	Spain	Both	75.1	97.2	57.1
2010	Spain	Female	32.2	41.0	25.2
2010	Spain	Male	120.1	156.4	89.98
2011	Greece	Both	30.3	41.6	22.0
2011	Greece	Female	12.1	16.0	9.03
2011	Greece	Male	49.6	69.2	35.8
2011	Italy	Both	84.6	95.3	75.0
2011	Italy	Female	42.5	49.2	36.4
2011	Italy	Male	130.1	145.2	116.1
2011	Portugal	Both	70.0	95.6	50.6
2011	Portugal	Female	26.6	35.8	19.2
2011	Portugal	Male	118.3	162.0	85.27
2011	Spain	Both	71.7	92.5	54.7
2011	Spain	Female	30.9	39.2	24.0
2011	Spain	Male	114.6	149.3	86.01
2012	Greece	Both	30.2	41.4	21.9
2012	Greece	Female	12.2	16.2	9.03
2012	Greece	Male	49.3	68.0	35.5
2012	Italy	Both	81.9	92.1	72.6
2012	Italy	Female	41.1	47.7	35.3
2012	Italy	Male	125.8	141.0	112.3
2012	Portugal	Both	66.3	90.6	47.7
2012	Portugal	Female	24.7	33.4	17.8

2012	Portugal	Male	112.7	155.0	80.69
2012	Spain	Both	68.5	88.5	52.5
2012	Spain	Female	29.2	37.0	22.8
2012	Spain	Male	109.9	142.8	82.55
2013	Greece	Both	29.6	40.9	21.3
2013	Greece	Female	11.8	15.9	8.76
2013	Greece	Male	48.4	67.1	34.5
2013	Italy	Both	78.8	88.6	69.6
2013	Italy	Female	39.6	45.9	33.9
2013	Italy	Male	121.1	135.6	107.8
2013	Portugal	Both	62.7	86.2	45.1
2013	Portugal	Female	23.2	31.2	16.9
2013	Portugal	Male	106.8	148.5	76.40
2013	Spain	Both	65.2	84.3	49.8
2013	Spain	Female	28.0	35.6	21.8
2013	Spain	Male	104.4	136.6	78.71
2014	Greece	Both	29.1	40.2	21.0
2014	Greece	Female	11.6	15.5	8.47
2014	Greece	Male	47.7	66.6	33.8
2014	Italy	Both	76.2	85.7	67.4
2014	Italy	Female	38.0	44.0	32.4
2014	Italy	Male	117.4	131.5	104.5
2014	Portugal	Both	59.4	82.2	42.5
2014	Portugal	Female	21.9	29.7	16.0
2014	Portugal	Male	101.3	141.3	71.89
2014	Spain	Both	63.0	81.9	48.3
2014	Spain	Female	26.9	34.3	20.7
2014	Spain	Male	101.0	132.7	76.22
2015	Greece	Both	28.9	40.0	20.9
2015	Greece	Female	11.5	15.5	8.46
2015	Greece	Male	47.5	66.3	33.7
2015	Italy	Both	76.2	85.8	67.5
2015	Italy	Female	38.5	44.6	33.0
2015	Italy	Male	116.7	131.3	104.0
2015	Portugal	Both	55.6	77.5	39.6
2015	Portugal	Female	21.2	29.1	15.5
2015	Portugal	Male	94.1	131.	66.3
2015	Spain	Both	62.3	81.4	48.0
2015	Spain	Female	26.8	34.0	20.5
2015	Spain	Male	99.9	132.	75.3
2016	Greece	Both	28.5	39.1	20.7
2016	Greece	Female	11.7	15.8	8.48
2016	Greece	Male	46.4	64.4	33.1
2016	Italy	Both	73.4	82.7	65.0
2016	Italy	Female	37.3	43.1	31.9
2016	Italy	Male	112.2	126.0	99.83
2016	Portugal	Both	55.1	77.0	39.3
2016	Portugal	Female	21.3	29.3	15.4
2016	Portugal	Male	93.1	131.	65.4
2016	Spain	Both	60.2	78.6	46.3
2016	Spain	Female	25.8	32.5	19.9
2016	Spain	Male	96.7	127.	72.6
2017	Greece	Both	28.4	38.9	20.6

2017	Greece	Female	11.8	16.1	8.51
2017	Greece	Male	46.2	63.7	32.7
2017	Italy	Both	73.4	82.8	65.0
2017	Italy	Female	37.3	43.3	32.1
2017	Italy	Male	112.1	125.9	99.96
2017	Portugal	Both	55.4	76.9	39.1
2017	Portugal	Female	21.6	29.6	15.4
2017	Portugal	Male	93.4	131.	65.6
2017	Spain	Both	60.5	79.0	46.1
2017	Spain	Female	26.0	33.0	20.3
2017	Spain	Male	96.9	127.	72.5
2018	Greece	Both	28.6	38.8	20.8
2018	Greece	Female	12.0	16.3	8.71
2018	Greece	Male	46.2	63.3	33.0
2018	Italy	Both	73.6	83.1	65.4
2018	Italy	Female	37.6	43.7	32.3
2018	Italy	Male	112.3	126.6	99.96
2018	Portugal	Both	55.9	77.1	40.1
2018	Portugal	Female	21.7	29.6	15.4
2018	Portugal	Male	94.3	131.	67.1
2018	Spain	Both	60.0	77.5	45.7
2018	Spain	Female	26.2	33.6	20.3
2018	Spain	Male	95.7	125.	72.3
2019	Greece	Both	28.7	39.1	21.0
2019	Greece	Female	12.1	16.2	8.72
2019	Greece	Male	46.4	63.6	33.1
2019	Italy	Both	73.4	82.7	65.1
2019	Italy	Female	37.5	43.7	32.1
2019	Italy	Male	111.8	125.7	99.33
2019	Portugal	Both	56.0	78.5	40.4
2019	Portugal	Female	21.6	29.8	15.7
2019	Portugal	Male	94.8	133.	67.5
2019	Spain	Both	59.4	76.8	45.2
2019	Spain	Female	26.1	33.4	19.9
2019	Spain	Male	94.4	123.	71.4

Table S25- Age-standardized rates and 95% uncertainty levels (UL) of **prevalence of liver cancer due to hepatitis B** per 100,000 population in Greece, Italy, Portugal and Spain from 2000 to 2019 by sex classes (Global Burden of Disease Study 2019).

Year	Country	Sex	Prevalence (95% UL)	95% UL (upper)	95% UL (lower)
2000	Greece	Both	1.42	1.77	1.13
2000	Greece	Female	0.69	0.88	0.53
2000	Greece	Male	2.24	2.80	1.76
2000	Italy	Both	1.67	1.93	1.41
2000	Italy	Female	0.66	0.79	0.54
2000	Italy	Male	2.76	3.20	2.34
2000	Portugal	Both	0.67	0.90	0.48
2000	Portugal	Female	0.27	0.37	0.19
2000	Portugal	Male	1.12	1.53	0.78
2000	Spain	Both	1.16	1.60	0.83
2000	Spain	Female	0.40	0.56	0.29
2000	Spain	Male	1.98	2.76	1.40
2001	Greece	Both	1.46	1.80	1.16
2001	Greece	Female	0.70	0.89	0.54
2001	Greece	Male	2.30	2.84	1.81
2001	Italy	Both	1.65	1.92	1.39
2001	Italy	Female	0.66	0.79	0.54
2001	Italy	Male	2.73	3.16	2.31
2001	Portugal	Both	0.69	0.93	0.50
2001	Portugal	Female	0.27	0.36	0.20
2001	Portugal	Male	1.16	1.58	0.82
2001	Spain	Both	1.23	1.68	0.88
2001	Spain	Female	0.41	0.57	0.30
2001	Spain	Male	2.10	2.90	1.49
2002	Greece	Both	1.49	1.83	1.18
2002	Greece	Female	0.71	0.91	0.55
2002	Greece	Male	2.34	2.89	1.86
2002	Italy	Both	1.61	1.87	1.36
2002	Italy	Female	0.65	0.77	0.53
2002	Italy	Male	2.65	3.08	2.24
2002	Portugal	Both	0.71	0.96	0.52
2002	Portugal	Female	0.27	0.36	0.20
2002	Portugal	Male	1.20	1.65	0.86
2002	Spain	Both	1.30	1.77	0.93
2002	Spain	Female	0.42	0.59	0.30
2002	Spain	Male	2.23	3.05	1.59
2003	Greece	Both	1.51	1.87	1.21
2003	Greece	Female	0.73	0.93	0.56
2003	Greece	Male	2.38	2.95	1.89
2003	Italy	Both	1.56	1.81	1.32
2003	Italy	Female	0.63	0.76	0.52
2003	Italy	Male	2.56	2.99	2.16
2003	Portugal	Both	0.73	1.00	0.53
2003	Portugal	Female	0.27	0.37	0.20
2003	Portugal	Male	1.25	1.73	0.89
2003	Spain	Both	1.37	1.86	0.98

2003	Spain	Female	0.43	0.59	0.31
2003	Spain	Male	2.36	3.22	1.69
2004	Greece	Both	1.54	1.89	1.23
2004	Greece	Female	0.74	0.95	0.57
2004	Greece	Male	2.43	2.99	1.92
2004	Italy	Both	1.52	1.76	1.28
2004	Italy	Female	0.62	0.75	0.51
2004	Italy	Male	2.49	2.91	2.08
2004	Portugal	Both	0.76	1.04	0.54
2004	Portugal	Female	0.27	0.37	0.19
2004	Portugal	Male	1.30	1.81	0.91
2004	Spain	Both	1.43	1.94	1.03
2004	Spain	Female	0.44	0.60	0.31
2004	Spain	Male	2.47	3.38	1.77
2005	Greece	Both	1.57	1.92	1.25
2005	Greece	Female	0.76	0.97	0.58
2005	Greece	Male	2.47	3.06	1.94
2005	Italy	Both	1.50	1.74	1.27
2005	Italy	Female	0.61	0.74	0.50
2005	Italy	Male	2.45	2.87	2.05
2005	Portugal	Both	0.78	1.09	0.55
2005	Portugal	Female	0.27	0.38	0.19
2005	Portugal	Male	1.35	1.88	0.93
2005	Spain	Both	1.46	2.02	1.05
2005	Spain	Female	0.44	0.61	0.31
2005	Spain	Male	2.54	3.51	1.81
2006	Greece	Both	1.61	1.97	1.28
2006	Greece	Female	0.77	0.98	0.59
2006	Greece	Male	2.53	3.12	1.99
2006	Italy	Both	1.50	1.73	1.27
2006	Italy	Female	0.61	0.73	0.50
2006	Italy	Male	2.45	2.86	2.06
2006	Portugal	Both	0.82	1.12	0.58
2006	Portugal	Female	0.28	0.38	0.20
2006	Portugal	Male	1.41	1.96	0.99
2006	Spain	Both	1.48	2.04	1.07
2006	Spain	Female	0.44	0.61	0.32
2006	Spain	Male	2.57	3.56	1.84
2007	Greece	Both	1.65	2.02	1.31
2007	Greece	Female	0.78	1.00	0.60
2007	Greece	Male	2.60	3.20	2.06
2007	Italy	Both	1.50	1.74	1.27
2007	Italy	Female	0.60	0.72	0.50
2007	Italy	Male	2.46	2.88	2.08
2007	Portugal	Both	0.86	1.17	0.61
2007	Portugal	Female	0.29	0.40	0.21
2007	Portugal	Male	1.49	2.06	1.04
2007	Spain	Both	1.49	2.05	1.08
2007	Spain	Female	0.44	0.61	0.31
2007	Spain	Male	2.60	3.61	1.85
2008	Greece	Both	1.69	2.07	1.34
2008	Greece	Female	0.80	1.01	0.60
2008	Greece	Male	2.67	3.30	2.11

2008	Italy	Both	1.51	1.74	1.28
2008	Italy	Female	0.60	0.71	0.50
2008	Italy	Male	2.48	2.89	2.10
2008	Portugal	Both	0.90	1.23	0.64
2008	Portugal	Female	0.30	0.41	0.21
2008	Portugal	Male	1.57	2.18	1.10
2008	Spain	Both	1.50	2.06	1.08
2008	Spain	Female	0.44	0.61	0.31
2008	Spain	Male	2.61	3.65	1.87
2009	Greece	Both	1.73	2.13	1.37
2009	Greece	Female	0.81	1.03	0.61
2009	Greece	Male	2.75	3.40	2.16
2009	Italy	Both	1.52	1.75	1.29
2009	Italy	Female	0.59	0.71	0.49
2009	Italy	Male	2.50	2.93	2.12
2009	Portugal	Both	0.94	1.30	0.67
2009	Portugal	Female	0.31	0.43	0.22
2009	Portugal	Male	1.65	2.30	1.15
2009	Spain	Both	1.50	2.08	1.08
2009	Spain	Female	0.43	0.61	0.31
2009	Spain	Male	2.62	3.64	1.86
2010	Greece	Both	1.78	2.21	1.39
2010	Greece	Female	0.82	1.05	0.61
2010	Greece	Male	2.83	3.52	2.19
2010	Italy	Both	1.52	1.78	1.30
2010	Italy	Female	0.59	0.72	0.49
2010	Italy	Male	2.52	2.97	2.13
2010	Portugal	Both	0.97	1.35	0.69
2010	Portugal	Female	0.32	0.45	0.23
2010	Portugal	Male	1.71	2.40	1.18
2010	Spain	Both	1.51	2.08	1.07
2010	Spain	Female	0.43	0.61	0.30
2010	Spain	Male	2.63	3.64	1.85
2011	Greece	Both	1.84	2.28	1.44
2011	Greece	Female	0.83	1.07	0.62
2011	Greece	Male	2.94	3.65	2.29
2011	Italy	Both	1.54	1.79	1.31
2011	Italy	Female	0.60	0.72	0.50
2011	Italy	Male	2.54	3.00	2.15
2011	Portugal	Both	1.00	1.38	0.71
2011	Portugal	Female	0.32	0.45	0.23
2011	Portugal	Male	1.76	2.47	1.23
2011	Spain	Both	1.52	2.10	1.08
2011	Spain	Female	0.43	0.60	0.30
2011	Spain	Male	2.65	3.68	1.85
2012	Greece	Both	1.92	2.37	1.51
2012	Greece	Female	0.85	1.09	0.63
2012	Greece	Male	3.09	3.84	2.41
2012	Italy	Both	1.56	1.81	1.33
2012	Italy	Female	0.60	0.72	0.50
2012	Italy	Male	2.58	3.02	2.19
2012	Portugal	Both	1.02	1.41	0.73
2012	Portugal	Female	0.33	0.45	0.23

2012	Portugal	Male	1.80	2.54	1.26
2012	Spain	Both	1.52	2.11	1.08
2012	Spain	Female	0.43	0.60	0.31
2012	Spain	Male	2.66	3.71	1.85
2013	Greece	Both	1.99	2.47	1.57
2013	Greece	Female	0.87	1.12	0.65
2013	Greece	Male	3.24	4.04	2.54
2013	Italy	Both	1.58	1.83	1.34
2013	Italy	Female	0.61	0.72	0.51
2013	Italy	Male	2.62	3.06	2.22
2013	Portugal	Both	1.05	1.44	0.74
2013	Portugal	Female	0.33	0.46	0.24
2013	Portugal	Male	1.85	2.59	1.30
2013	Spain	Both	1.52	2.11	1.08
2013	Spain	Female	0.43	0.60	0.31
2013	Spain	Male	2.66	3.74	1.86
2014	Greece	Both	2.06	2.56	1.62
2014	Greece	Female	0.88	1.14	0.65
2014	Greece	Male	3.36	4.18	2.60
2014	Italy	Both	1.60	1.85	1.35
2014	Italy	Female	0.61	0.73	0.51
2014	Italy	Male	2.65	3.11	2.24
2014	Portugal	Both	1.07	1.47	0.76
2014	Portugal	Female	0.33	0.46	0.24
2014	Portugal	Male	1.89	2.66	1.33
2014	Spain	Both	1.52	2.12	1.07
2014	Spain	Female	0.43	0.60	0.31
2014	Spain	Male	2.66	3.74	1.85
2015	Greece	Both	2.08	2.61	1.63
2015	Greece	Female	0.89	1.16	0.66
2015	Greece	Male	3.40	4.26	2.64
2015	Italy	Both	1.60	1.87	1.36
2015	Italy	Female	0.61	0.74	0.50
2015	Italy	Male	2.66	3.15	2.23
2015	Portugal	Both	1.08	1.52	0.77
2015	Portugal	Female	0.34	0.47	0.24
2015	Portugal	Male	1.92	2.72	1.35
2015	Spain	Both	1.51	2.12	1.05
2015	Spain	Female	0.43	0.60	0.30
2015	Spain	Male	2.65	3.74	1.85
2016	Greece	Both	2.06	2.62	1.60
2016	Greece	Female	0.89	1.17	0.66
2016	Greece	Male	3.36	4.26	2.61
2016	Italy	Both	1.59	1.88	1.34
2016	Italy	Female	0.60	0.73	0.49
2016	Italy	Male	2.63	3.14	2.19
2016	Portugal	Both	1.11	1.54	0.78
2016	Portugal	Female	0.34	0.48	0.24
2016	Portugal	Male	1.96	2.79	1.34
2016	Spain	Both	1.49	2.08	1.04
2016	Spain	Female	0.43	0.60	0.30
2016	Spain	Male	2.60	3.67	1.79
2017	Greece	Both	2.03	2.64	1.52

2017	Greece	Female	0.89	1.19	0.65
2017	Greece	Male	3.29	4.30	2.44
2017	Italy	Both	1.57	1.92	1.26
2017	Italy	Female	0.60	0.75	0.46
2017	Italy	Male	2.60	3.26	2.02
2017	Portugal	Both	1.12	1.60	0.75
2017	Portugal	Female	0.34	0.49	0.24
2017	Portugal	Male	1.99	2.90	1.31
2017	Spain	Both	1.46	2.11	0.98
2017	Spain	Female	0.43	0.61	0.28
2017	Spain	Male	2.54	3.68	1.71
2018	Greece	Both	2.01	2.64	1.49
2018	Greece	Female	0.89	1.22	0.64
2018	Greece	Male	3.24	4.34	2.37
2018	Italy	Both	1.59	1.95	1.27
2018	Italy	Female	0.60	0.76	0.47
2018	Italy	Male	2.63	3.31	2.05
2018	Portugal	Both	1.12	1.65	0.75
2018	Portugal	Female	0.35	0.51	0.24
2018	Portugal	Male	2.00	2.95	1.32
2018	Spain	Both	1.44	2.09	0.98
2018	Spain	Female	0.43	0.63	0.29
2018	Spain	Male	2.50	3.65	1.70
2019	Greece	Both	1.99	2.77	1.41
2019	Greece	Female	0.89	1.27	0.61
2019	Greece	Male	3.21	4.47	2.22
2019	Italy	Both	1.62	2.13	1.21
2019	Italy	Female	0.61	0.83	0.44
2019	Italy	Male	2.70	3.72	1.91
2019	Portugal	Both	1.11	1.72	0.71
2019	Portugal	Female	0.35	0.53	0.22
2019	Portugal	Male	1.98	3.05	1.23
2019	Spain	Both	1.43	2.13	0.92
2019	Spain	Female	0.43	0.66	0.28
2019	Spain	Male	2.47	3.73	1.56

Table S26- Age-standardized rates and 95% uncertainty levels (UL) of **incidence of liver cancer due to hepatitis B** per 100,000 population in Greece, Italy, Portugal and Spain from 2000 to 2019 by sex classes (Global Burden of Disease Study 2019).

Year	Country	Sex	Incidence (95% UL)	95% UL (upper)	95% UL (lower)
2000	Greece	Both	1.03	1.30	0.80
2000	Greece	Female	0.50	0.66	0.38
2000	Greece	Male	1.63	2.07	1.25
2000	Italy	Both	0.93	1.08	0.79
2000	Italy	Female	0.40	0.47	0.33
2000	Italy	Male	1.54	1.79	1.31
2000	Portugal	Both	0.52	0.72	0.37
2000	Portugal	Female	0.22	0.30	0.15
2000	Portugal	Male	0.87	1.22	0.60
2000	Spain	Both	0.72	1.00	0.51
2000	Spain	Female	0.29	0.41	0.20
2000	Spain	Male	1.20	1.69	0.84
2001	Greece	Both	1.04	1.31	0.81
2001	Greece	Female	0.50	0.66	0.38
2001	Greece	Male	1.65	2.08	1.27
2001	Italy	Both	0.90	1.05	0.77
2001	Italy	Female	0.39	0.46	0.32
2001	Italy	Male	1.48	1.72	1.26
2001	Portugal	Both	0.53	0.73	0.37
2001	Portugal	Female	0.22	0.30	0.15
2001	Portugal	Male	0.89	1.24	0.62
2001	Spain	Both	0.74	1.02	0.52
2001	Spain	Female	0.29	0.41	0.20
2001	Spain	Male	1.23	1.71	0.86
2002	Greece	Both	1.05	1.31	0.81
2002	Greece	Female	0.51	0.66	0.38
2002	Greece	Male	1.66	2.08	1.29
2002	Italy	Both	0.85	0.99	0.72
2002	Italy	Female	0.37	0.44	0.31
2002	Italy	Male	1.40	1.62	1.19
2002	Portugal	Both	0.54	0.74	0.38
2002	Portugal	Female	0.21	0.29	0.15
2002	Portugal	Male	0.91	1.27	0.63
2002	Spain	Both	0.75	1.04	0.53
2002	Spain	Female	0.29	0.42	0.20
2002	Spain	Male	1.26	1.75	0.89
2003	Greece	Both	1.05	1.32	0.82
2003	Greece	Female	0.51	0.67	0.39
2003	Greece	Male	1.66	2.09	1.29
2003	Italy	Both	0.80	0.93	0.68
2003	Italy	Female	0.35	0.41	0.29
2003	Italy	Male	1.30	1.51	1.11
2003	Portugal	Both	0.55	0.76	0.39

2003	Portugal	Female	0.21	0.29	0.15
2003	Portugal	Male	0.93	1.30	0.65
2003	Spain	Both	0.77	1.06	0.55
2003	Spain	Female	0.29	0.41	0.20
2003	Spain	Male	1.29	1.80	0.92
2004	Greece	Both	1.06	1.33	0.82
2004	Greece	Female	0.52	0.67	0.39
2004	Greece	Male	1.67	2.10	1.30
2004	Italy	Both	0.75	0.87	0.64
2004	Italy	Female	0.33	0.39	0.28
2004	Italy	Male	1.22	1.42	1.04
2004	Portugal	Both	0.56	0.78	0.39
2004	Portugal	Female	0.21	0.29	0.15
2004	Portugal	Male	0.95	1.34	0.66
2004	Spain	Both	0.78	1.08	0.56
2004	Spain	Female	0.29	0.41	0.20
2004	Spain	Male	1.31	1.83	0.93
2005	Greece	Both	1.07	1.34	0.83
2005	Greece	Female	0.52	0.69	0.39
2005	Greece	Male	1.68	2.12	1.30
2005	Italy	Both	0.73	0.84	0.62
2005	Italy	Female	0.32	0.38	0.27
2005	Italy	Male	1.18	1.37	1.00
2005	Portugal	Both	0.57	0.80	0.39
2005	Portugal	Female	0.21	0.29	0.14
2005	Portugal	Male	0.98	1.39	0.66
2005	Spain	Both	0.78	1.09	0.56
2005	Spain	Female	0.28	0.41	0.20
2005	Spain	Male	1.33	1.84	0.93
2006	Greece	Both	1.08	1.36	0.84
2006	Greece	Female	0.52	0.69	0.39
2006	Greece	Male	1.71	2.13	1.32
2006	Italy	Both	0.72	0.83	0.61
2006	Italy	Female	0.31	0.37	0.26
2006	Italy	Male	1.17	1.35	0.99
2006	Portugal	Both	0.58	0.82	0.41
2006	Portugal	Female	0.21	0.30	0.15
2006	Portugal	Male	1.01	1.43	0.70
2006	Spain	Both	0.78	1.09	0.56
2006	Spain	Female	0.28	0.40	0.19
2006	Spain	Male	1.33	1.85	0.94
2007	Greece	Both	1.10	1.38	0.85
2007	Greece	Female	0.52	0.69	0.39
2007	Greece	Male	1.73	2.17	1.35
2007	Italy	Both	0.71	0.82	0.61
2007	Italy	Female	0.31	0.36	0.26
2007	Italy	Male	1.16	1.35	0.99
2007	Portugal	Both	0.61	0.85	0.43
2007	Portugal	Female	0.21	0.31	0.15
2007	Portugal	Male	1.06	1.49	0.73

2007	Spain	Both	0.78	1.08	0.56
2007	Spain	Female	0.28	0.39	0.19
2007	Spain	Male	1.32	1.84	0.94
2008	Greece	Both	1.11	1.41	0.86
2008	Greece	Female	0.53	0.69	0.39
2008	Greece	Male	1.77	2.21	1.37
2008	Italy	Both	0.71	0.82	0.61
2008	Italy	Female	0.31	0.36	0.26
2008	Italy	Male	1.16	1.35	1.00
2008	Portugal	Both	0.64	0.88	0.45
2008	Portugal	Female	0.22	0.32	0.15
2008	Portugal	Male	1.11	1.55	0.77
2008	Spain	Both	0.77	1.08	0.55
2008	Spain	Female	0.27	0.39	0.19
2008	Spain	Male	1.32	1.83	0.93
2009	Greece	Both	1.13	1.43	0.88
2009	Greece	Female	0.53	0.69	0.40
2009	Greece	Male	1.81	2.28	1.40
2009	Italy	Both	0.71	0.82	0.61
2009	Italy	Female	0.30	0.36	0.25
2009	Italy	Male	1.16	1.35	1.00
2009	Portugal	Both	0.66	0.92	0.46
2009	Portugal	Female	0.23	0.33	0.16
2009	Portugal	Male	1.15	1.61	0.80
2009	Spain	Both	0.77	1.08	0.54
2009	Spain	Female	0.27	0.38	0.18
2009	Spain	Male	1.31	1.81	0.92
2010	Greece	Both	1.15	1.46	0.89
2010	Greece	Female	0.53	0.70	0.39
2010	Greece	Male	1.85	2.35	1.42
2010	Italy	Both	0.71	0.82	0.61
2010	Italy	Female	0.30	0.36	0.25
2010	Italy	Male	1.17	1.36	1.00
2010	Portugal	Both	0.68	0.95	0.47
2010	Portugal	Female	0.23	0.33	0.16
2010	Portugal	Male	1.19	1.68	0.82
2010	Spain	Both	0.77	1.07	0.54
2010	Spain	Female	0.27	0.38	0.18
2010	Spain	Male	1.31	1.82	0.91
2011	Greece	Both	1.19	1.51	0.91
2011	Greece	Female	0.54	0.71	0.40
2011	Greece	Male	1.92	2.44	1.47
2011	Italy	Both	0.71	0.83	0.61
2011	Italy	Female	0.30	0.36	0.25
2011	Italy	Male	1.17	1.36	1.01
2011	Portugal	Both	0.69	0.96	0.48
2011	Portugal	Female	0.23	0.33	0.16
2011	Portugal	Male	1.22	1.70	0.84
2011	Spain	Both	0.77	1.07	0.54
2011	Spain	Female	0.26	0.38	0.18

2011	Spain	Male	1.31	1.83	0.91
2012	Greece	Both	1.25	1.58	0.95
2012	Greece	Female	0.55	0.72	0.40
2012	Greece	Male	2.03	2.56	1.55
2012	Italy	Both	0.72	0.83	0.62
2012	Italy	Female	0.30	0.36	0.26
2012	Italy	Male	1.18	1.36	1.01
2012	Portugal	Both	0.70	0.98	0.50
2012	Portugal	Female	0.23	0.33	0.16
2012	Portugal	Male	1.24	1.74	0.87
2012	Spain	Both	0.77	1.08	0.55
2012	Spain	Female	0.26	0.38	0.18
2012	Spain	Male	1.32	1.85	0.92
2013	Greece	Both	1.30	1.65	1.00
2013	Greece	Female	0.56	0.74	0.41
2013	Greece	Male	2.14	2.70	1.64
2013	Italy	Both	0.73	0.84	0.62
2013	Italy	Female	0.30	0.36	0.26
2013	Italy	Male	1.19	1.38	1.02
2013	Portugal	Both	0.71	1.00	0.50
2013	Portugal	Female	0.24	0.33	0.16
2013	Portugal	Male	1.26	1.79	0.88
2013	Spain	Both	0.78	1.10	0.55
2013	Spain	Female	0.27	0.38	0.18
2013	Spain	Male	1.34	1.88	0.93
2014	Greece	Both	1.35	1.71	1.04
2014	Greece	Female	0.57	0.75	0.42
2014	Greece	Male	2.23	2.82	1.70
2014	Italy	Both	0.73	0.84	0.63
2014	Italy	Female	0.31	0.36	0.26
2014	Italy	Male	1.20	1.39	1.03
2014	Portugal	Both	0.72	1.02	0.51
2014	Portugal	Female	0.24	0.34	0.16
2014	Portugal	Male	1.28	1.83	0.89
2014	Spain	Both	0.78	1.11	0.55
2014	Spain	Female	0.27	0.38	0.18
2014	Spain	Male	1.34	1.92	0.93
2015	Greece	Both	1.37	1.75	1.05
2015	Greece	Female	0.57	0.76	0.42
2015	Greece	Male	2.26	2.87	1.72
2015	Italy	Both	0.73	0.85	0.63
2015	Italy	Female	0.31	0.36	0.26
2015	Italy	Male	1.20	1.40	1.03
2015	Portugal	Both	0.73	1.04	0.51
2015	Portugal	Female	0.24	0.34	0.16
2015	Portugal	Male	1.30	1.87	0.89
2015	Spain	Both	0.78	1.12	0.55
2015	Spain	Female	0.26	0.39	0.18
2015	Spain	Male	1.34	1.93	0.92
2016	Greece	Both	1.36	1.74	1.03

2016	Greece	Female	0.57	0.77	0.42
2016	Greece	Male	2.24	2.86	1.71
2016	Italy	Both	0.72	0.86	0.61
2016	Italy	Female	0.30	0.36	0.25
2016	Italy	Male	1.19	1.41	1.00
2016	Portugal	Both	0.74	1.04	0.51
2016	Portugal	Female	0.24	0.34	0.17
2016	Portugal	Male	1.32	1.88	0.89
2016	Spain	Both	0.77	1.10	0.52
2016	Spain	Female	0.26	0.38	0.18
2016	Spain	Male	1.32	1.89	0.89
2017	Greece	Both	1.34	1.75	0.97
2017	Greece	Female	0.57	0.78	0.41
2017	Greece	Male	2.19	2.89	1.61
2017	Italy	Both	0.72	0.88	0.58
2017	Italy	Female	0.30	0.37	0.23
2017	Italy	Male	1.17	1.46	0.93
2017	Portugal	Both	0.75	1.09	0.50
2017	Portugal	Female	0.24	0.35	0.16
2017	Portugal	Male	1.33	1.96	0.87
2017	Spain	Both	0.76	1.11	0.50
2017	Spain	Female	0.26	0.38	0.17
2017	Spain	Male	1.29	1.91	0.85
2018	Greece	Both	1.32	1.77	0.97
2018	Greece	Female	0.57	0.78	0.41
2018	Greece	Male	2.16	2.91	1.56
2018	Italy	Both	0.72	0.88	0.58
2018	Italy	Female	0.30	0.38	0.24
2018	Italy	Male	1.19	1.49	0.94
2018	Portugal	Both	0.74	1.09	0.50
2018	Portugal	Female	0.24	0.36	0.16
2018	Portugal	Male	1.32	1.97	0.87
2018	Spain	Both	0.75	1.10	0.50
2018	Spain	Female	0.26	0.39	0.17
2018	Spain	Male	1.27	1.88	0.83
2019	Greece	Both	1.31	1.84	0.91
2019	Greece	Female	0.58	0.82	0.39
2019	Greece	Male	2.12	3.01	1.46
2019	Italy	Both	0.75	0.98	0.56
2019	Italy	Female	0.30	0.40	0.22
2019	Italy	Male	1.23	1.68	0.88
2019	Portugal	Both	0.73	1.13	0.46
2019	Portugal	Female	0.24	0.37	0.15
2019	Portugal	Male	1.30	2.02	0.80
2019	Spain	Both	0.73	1.13	0.46
2019	Spain	Female	0.26	0.40	0.17
2019	Spain	Male	1.24	1.91	0.78

Table S27- Age-standardized rates and 95% uncertainty levels (UL) of **deaths due to liver cancer due to hepatitis B** per 100,000 population in Greece, Italy, Portugal and Spain from 2000 to 2019 by sex classes (Global Burden of Disease Study 2019).

Year	Country	Sex	Deaths (95% UL)	95% UL (upper)	95% UL (lower)
2000	Greece	Both	0.94	1.20	0.72
2000	Greece	Female	0.47	0.63	0.35
2000	Greece	Male	1.48	1.87	1.11
2000	Italy	Both	0.73	0.85	0.61
2000	Italy	Female	0.32	0.39	0.27
2000	Italy	Male	1.20	1.41	1.01
2000	Portugal	Both	0.49	0.67	0.34
2000	Portugal	Female	0.21	0.30	0.14
2000	Portugal	Male	0.81	1.14	0.56
2000	Spain	Both	0.60	0.84	0.42
2000	Spain	Female	0.27	0.39	0.18
2000	Spain	Male	0.98	1.37	0.68
2001	Greece	Both	0.95	1.21	0.72
2001	Greece	Female	0.47	0.63	0.35
2001	Greece	Male	1.49	1.89	1.13
2001	Italy	Both	0.69	0.81	0.58
2001	Italy	Female	0.31	0.37	0.26
2001	Italy	Male	1.13	1.33	0.95
2001	Portugal	Both	0.50	0.69	0.35
2001	Portugal	Female	0.21	0.30	0.15
2001	Portugal	Male	0.83	1.17	0.57
2001	Spain	Both	0.60	0.85	0.42
2001	Spain	Female	0.27	0.38	0.18
2001	Spain	Male	0.99	1.38	0.69
2002	Greece	Both	0.94	1.20	0.72
2002	Greece	Female	0.47	0.64	0.35
2002	Greece	Male	1.48	1.87	1.12
2002	Italy	Both	0.64	0.76	0.54
2002	Italy	Female	0.29	0.35	0.24
2002	Italy	Male	1.05	1.23	0.89
2002	Portugal	Both	0.48	0.67	0.33
2002	Portugal	Female	0.20	0.28	0.14
2002	Portugal	Male	0.81	1.14	0.56
2002	Spain	Both	0.61	0.86	0.43
2002	Spain	Female	0.26	0.38	0.18
2002	Spain	Male	1.01	1.42	0.70
2003	Greece	Both	0.94	1.20	0.72
2003	Greece	Female	0.47	0.63	0.34
2003	Greece	Male	1.48	1.89	1.13
2003	Italy	Both	0.59	0.69	0.50
2003	Italy	Female	0.27	0.32	0.22
2003	Italy	Male	0.96	1.12	0.81
2003	Portugal	Both	0.49	0.68	0.34
2003	Portugal	Female	0.20	0.28	0.14
2003	Portugal	Male	0.83	1.17	0.57
2003	Spain	Both	0.61	0.85	0.43

2003	Spain	Female	0.26	0.37	0.17
2003	Spain	Male	1.00	1.42	0.70
2004	Greece	Both	0.93	1.19	0.71
2004	Greece	Female	0.46	0.63	0.34
2004	Greece	Male	1.46	1.87	1.11
2004	Italy	Both	0.55	0.65	0.47
2004	Italy	Female	0.26	0.31	0.21
2004	Italy	Male	0.89	1.05	0.75
2004	Portugal	Both	0.50	0.69	0.34
2004	Portugal	Female	0.20	0.28	0.13
2004	Portugal	Male	0.85	1.19	0.59
2004	Spain	Both	0.60	0.85	0.42
2004	Spain	Female	0.25	0.36	0.17
2004	Spain	Male	1.00	1.41	0.69
2005	Greece	Both	0.94	1.20	0.71
2005	Greece	Female	0.47	0.63	0.34
2005	Greece	Male	1.47	1.88	1.10
2005	Italy	Both	0.52	0.61	0.44
2005	Italy	Female	0.24	0.29	0.20
2005	Italy	Male	0.84	0.99	0.71
2005	Portugal	Both	0.51	0.71	0.35
2005	Portugal	Female	0.20	0.28	0.13
2005	Portugal	Male	0.88	1.23	0.61
2005	Spain	Both	0.59	0.84	0.41
2005	Spain	Female	0.24	0.35	0.17
2005	Spain	Male	0.98	1.39	0.68
2006	Greece	Both	0.93	1.18	0.70
2006	Greece	Female	0.47	0.62	0.34
2006	Greece	Male	1.45	1.85	1.10
2006	Italy	Both	0.50	0.59	0.43
2006	Italy	Female	0.23	0.28	0.19
2006	Italy	Male	0.82	0.96	0.69
2006	Portugal	Both	0.53	0.73	0.37
2006	Portugal	Female	0.20	0.28	0.13
2006	Portugal	Male	0.91	1.28	0.63
2006	Spain	Both	0.58	0.82	0.41
2006	Spain	Female	0.24	0.35	0.16
2006	Spain	Male	0.96	1.36	0.67
2007	Greece	Both	0.94	1.20	0.71
2007	Greece	Female	0.46	0.62	0.34
2007	Greece	Male	1.48	1.89	1.11
2007	Italy	Both	0.49	0.58	0.41
2007	Italy	Female	0.23	0.27	0.19
2007	Italy	Male	0.79	0.94	0.67
2007	Portugal	Both	0.54	0.75	0.38
2007	Portugal	Female	0.20	0.28	0.14
2007	Portugal	Male	0.94	1.31	0.65
2007	Spain	Both	0.57	0.81	0.40
2007	Spain	Female	0.23	0.34	0.16
2007	Spain	Male	0.96	1.35	0.67
2008	Greece	Both	0.93	1.19	0.70
2008	Greece	Female	0.46	0.62	0.33
2008	Greece	Male	1.46	1.86	1.10

2008	Italy	Both	0.49	0.57	0.41
2008	Italy	Female	0.22	0.27	0.19
2008	Italy	Male	0.79	0.92	0.67
2008	Portugal	Both	0.55	0.77	0.38
2008	Portugal	Female	0.20	0.28	0.13
2008	Portugal	Male	0.96	1.35	0.67
2008	Spain	Both	0.57	0.80	0.40
2008	Spain	Female	0.23	0.34	0.15
2008	Spain	Male	0.94	1.32	0.66
2009	Greece	Both	0.96	1.24	0.72
2009	Greece	Female	0.46	0.62	0.34
2009	Greece	Male	1.53	1.96	1.16
2009	Italy	Both	0.49	0.58	0.42
2009	Italy	Female	0.22	0.27	0.18
2009	Italy	Male	0.80	0.94	0.68
2009	Portugal	Both	0.58	0.81	0.40
2009	Portugal	Female	0.20	0.30	0.14
2009	Portugal	Male	1.01	1.42	0.69
2009	Spain	Both	0.56	0.79	0.39
2009	Spain	Female	0.23	0.34	0.15
2009	Spain	Male	0.94	1.32	0.65
2010	Greece	Both	1.00	1.29	0.75
2010	Greece	Female	0.46	0.62	0.34
2010	Greece	Male	1.60	2.05	1.21
2010	Italy	Both	0.50	0.59	0.43
2010	Italy	Female	0.23	0.27	0.19
2010	Italy	Male	0.82	0.96	0.70
2010	Portugal	Both	0.59	0.84	0.41
2010	Portugal	Female	0.21	0.31	0.14
2010	Portugal	Male	1.04	1.47	0.71
2010	Spain	Both	0.56	0.79	0.39
2010	Spain	Female	0.22	0.33	0.15
2010	Spain	Male	0.94	1.33	0.65
2011	Greece	Both	1.05	1.35	0.79
2011	Greece	Female	0.47	0.63	0.34
2011	Greece	Male	1.70	2.18	1.29
2011	Italy	Both	0.51	0.60	0.44
2011	Italy	Female	0.23	0.28	0.19
2011	Italy	Male	0.84	0.97	0.71
2011	Portugal	Both	0.62	0.87	0.42
2011	Portugal	Female	0.21	0.31	0.14
2011	Portugal	Male	1.08	1.53	0.74
2011	Spain	Both	0.57	0.80	0.39
2011	Spain	Female	0.22	0.33	0.15
2011	Spain	Male	0.95	1.35	0.65
2012	Greece	Both	1.10	1.42	0.83
2012	Greece	Female	0.48	0.65	0.35
2012	Greece	Male	1.81	2.32	1.36
2012	Italy	Both	0.52	0.61	0.44
2012	Italy	Female	0.23	0.28	0.19
2012	Italy	Male	0.85	0.99	0.72
2012	Portugal	Both	0.63	0.89	0.43
2012	Portugal	Female	0.22	0.32	0.15

2012	Portugal	Male	1.11	1.57	0.76
2012	Spain	Both	0.58	0.82	0.39
2012	Spain	Female	0.22	0.33	0.15
2012	Spain	Male	0.97	1.38	0.66
2013	Greece	Both	1.16	1.49	0.88
2013	Greece	Female	0.49	0.66	0.36
2013	Greece	Male	1.92	2.46	1.45
2013	Italy	Both	0.51	0.61	0.44
2013	Italy	Female	0.23	0.28	0.19
2013	Italy	Male	0.84	0.99	0.72
2013	Portugal	Both	0.64	0.90	0.44
2013	Portugal	Female	0.22	0.32	0.15
2013	Portugal	Male	1.13	1.61	0.78
2013	Spain	Both	0.58	0.83	0.40
2013	Spain	Female	0.22	0.33	0.15
2013	Spain	Male	0.97	1.39	0.66
2014	Greece	Both	1.17	1.50	0.88
2014	Greece	Female	0.50	0.67	0.36
2014	Greece	Male	1.93	2.46	1.46
2014	Italy	Both	0.51	0.60	0.43
2014	Italy	Female	0.23	0.27	0.19
2014	Italy	Male	0.82	0.98	0.70
2014	Portugal	Both	0.64	0.91	0.44
2014	Portugal	Female	0.22	0.31	0.15
2014	Portugal	Male	1.13	1.63	0.77
2014	Spain	Both	0.58	0.83	0.40
2014	Spain	Female	0.22	0.33	0.15
2014	Spain	Male	0.98	1.40	0.66
2015	Greece	Both	1.18	1.51	0.88
2015	Greece	Female	0.50	0.67	0.36
2015	Greece	Male	1.95	2.48	1.47
2015	Italy	Both	0.50	0.59	0.42
2015	Italy	Female	0.22	0.27	0.19
2015	Italy	Male	0.81	0.96	0.69
2015	Portugal	Both	0.64	0.91	0.44
2015	Portugal	Female	0.21	0.31	0.14
2015	Portugal	Male	1.14	1.62	0.78
2015	Spain	Both	0.58	0.84	0.40
2015	Spain	Female	0.22	0.33	0.15
2015	Spain	Male	0.98	1.42	0.66
2016	Greece	Both	1.19	1.53	0.89
2016	Greece	Female	0.50	0.67	0.36
2016	Greece	Male	1.96	2.50	1.48
2016	Italy	Both	0.47	0.56	0.40
2016	Italy	Female	0.21	0.26	0.18
2016	Italy	Male	0.76	0.91	0.64
2016	Portugal	Both	0.65	0.92	0.45
2016	Portugal	Female	0.22	0.31	0.15
2016	Portugal	Male	1.15	1.64	0.79
2016	Spain	Both	0.57	0.84	0.39
2016	Spain	Female	0.22	0.33	0.15
2016	Spain	Male	0.97	1.42	0.64
2017	Greece	Both	1.16	1.50	0.87

2017	Greece	Female	0.49	0.67	0.36
2017	Greece	Male	1.92	2.46	1.45
2017	Italy	Both	0.49	0.59	0.41
2017	Italy	Female	0.22	0.27	0.18
2017	Italy	Male	0.80	0.96	0.66
2017	Portugal	Both	0.64	0.91	0.44
2017	Portugal	Female	0.22	0.31	0.15
2017	Portugal	Male	1.14	1.63	0.78
2017	Spain	Both	0.57	0.83	0.38
2017	Spain	Female	0.22	0.33	0.15
2017	Spain	Male	0.95	1.40	0.63
2018	Greece	Both	1.14	1.49	0.85
2018	Greece	Female	0.49	0.67	0.36
2018	Greece	Male	1.88	2.42	1.40
2018	Italy	Both	0.51	0.61	0.42
2018	Italy	Female	0.22	0.27	0.18
2018	Italy	Male	0.83	1.00	0.68
2018	Portugal	Both	0.63	0.90	0.44
2018	Portugal	Female	0.22	0.31	0.15
2018	Portugal	Male	1.12	1.62	0.76
2018	Spain	Both	0.56	0.81	0.37
2018	Spain	Female	0.22	0.33	0.15
2018	Spain	Male	0.93	1.36	0.62
2019	Greece	Both	1.13	1.48	0.84
2019	Greece	Female	0.49	0.67	0.36
2019	Greece	Male	1.84	2.41	1.37
2019	Italy	Both	0.52	0.63	0.43
2019	Italy	Female	0.22	0.27	0.18
2019	Italy	Male	0.85	1.04	0.70
2019	Portugal	Both	0.62	0.90	0.43
2019	Portugal	Female	0.21	0.32	0.14
2019	Portugal	Male	1.10	1.61	0.75
2019	Spain	Both	0.55	0.80	0.37
2019	Spain	Female	0.22	0.33	0.15
2019	Spain	Male	0.92	1.34	0.61

Table S28- Age-standardized rates and 95% uncertainty levels (UL) of **years of life lost** (YLLs) **due to liver cancer due to hepatitis B** per 100,000 population in Greece, Italy, Portugal and Spain from 2000 to 2019 by sex classes (Global Burden of Disease Study 2019).

Year	Country	Sex	YLLs (95% UL)	95% UL (upper)	95% UL (lower)
2000	Greece	Both	22.5	28.2	17.9
2000	Greece	Female	10.3	13.3	8.00
2000	Greece	Male	36.1	45.3	28.3
2000	Italy	Both	20.1	23.4	17.1
2000	Italy	Female	8.35	9.79	7.05
2000	Italy	Male	33.2	38.5	28.2
2000	Portugal	Both	14.8	20.2	10.7
2000	Portugal	Female	6.05	8.08	4.42
2000	Portugal	Male	24.7	33.9	17.5
2000	Spain	Both	16.9	23.0	12.0
2000	Spain	Female	6.99	9.65	4.97
2000	Spain	Male	27.6	37.6	19.6
2001	Greece	Both	22.7	28.2	18.1
2001	Greece	Female	10.4	13.4	8.10
2001	Greece	Male	36.3	45.5	28.4
2001	Italy	Both	18.9	22.0	16.2
2001	Italy	Female	7.91	9.26	6.69
2001	Italy	Male	31.2	36.4	26.7
2001	Portugal	Both	15.1	20.6	10.9
2001	Portugal	Female	6.19	8.25	4.50
2001	Portugal	Male	25.2	34.4	17.8
2001	Spain	Both	17.1	23.2	12.2
2001	Spain	Female	6.93	9.48	4.98
2001	Spain	Male	28.1	38.3	19.9
2002	Greece	Both	22.5	27.8	18.0
2002	Greece	Female	10.4	13.3	8.02
2002	Greece	Male	36.0	44.8	28.6
2002	Italy	Both	17.7	20.6	15.1
2002	Italy	Female	7.53	8.85	6.36
2002	Italy	Male	29.1	33.9	24.7
2002	Portugal	Both	14.8	20.0	10.6
2002	Portugal	Female	5.85	7.79	4.27
2002	Portugal	Male	24.8	34.2	17.5
2002	Spain	Both	17.3	23.4	12.4
2002	Spain	Female	6.90	9.43	4.95
2002	Spain	Male	28.6	38.9	20.5
2003	Greece	Both	22.7	28.3	18.1
2003	Greece	Female	10.3	13.2	8.01
2003	Greece	Male	36.4	45.5	28.8
2003	Italy	Both	16.2	18.8	13.8
2003	Italy	Female	6.93	8.14	5.85
2003	Italy	Male	26.5	30.8	22.7
2003	Portugal	Both	15.0	20.3	10.7
2003	Portugal	Female	5.74	7.71	4.19
2003	Portugal	Male	25.4	35.0	17.8
2003	Spain	Both	17.3	23.5	12.5

2003	Spain	Female	6.67	9.08	4.78
2003	Spain	Male	28.6	39.2	20.7
2004	Greece	Both	22.5	28.1	17.9
2004	Greece	Female	10.2	13.1	7.99
2004	Greece	Male	36.1	45.0	28.4
2004	Italy	Both	15.3	17.7	13.0
2004	Italy	Female	6.62	7.79	5.61
2004	Italy	Male	24.8	28.9	21.1
2004	Portugal	Both	15.2	20.8	10.8
2004	Portugal	Female	5.68	7.61	4.10
2004	Portugal	Male	25.8	35.6	18.1
2004	Spain	Both	17.2	23.5	12.5
2004	Spain	Female	6.55	8.98	4.66
2004	Spain	Male	28.6	39.2	20.6
2005	Greece	Both	22.8	28.3	18.0
2005	Greece	Female	10.4	13.3	8.09
2005	Greece	Male	36.4	45.5	28.5
2005	Italy	Both	14.4	16.7	12.2
2005	Italy	Female	6.25	7.38	5.30
2005	Italy	Male	23.3	27.2	19.8
2005	Portugal	Both	15.7	21.5	11.1
2005	Portugal	Female	5.72	7.72	4.13
2005	Portugal	Male	26.9	37.4	18.7
2005	Spain	Both	16.9	23.1	12.2
2005	Spain	Female	6.36	8.78	4.50
2005	Spain	Male	28.2	38.9	20.2
2006	Greece	Both	22.6	28.0	17.7
2006	Greece	Female	10.4	13.4	8.04
2006	Greece	Male	36.0	44.8	28.1
2006	Italy	Both	14.0	16.2	11.9
2006	Italy	Female	6.02	7.08	5.10
2006	Italy	Male	22.7	26.5	19.4
2006	Portugal	Both	16.2	22.2	11.5
2006	Portugal	Female	5.70	7.79	4.06
2006	Portugal	Male	28.0	38.8	19.5
2006	Spain	Both	16.7	22.9	12.1
2006	Spain	Female	6.21	8.53	4.41
2006	Spain	Male	27.9	38.4	19.9
2007	Greece	Both	22.9	28.4	18.1
2007	Greece	Female	10.4	13.4	8.03
2007	Greece	Male	36.7	45.8	28.8
2007	Italy	Both	13.6	15.7	11.6
2007	Italy	Female	5.80	6.81	4.93
2007	Italy	Male	22.0	25.6	18.8
2007	Portugal	Both	16.6	22.9	11.8
2007	Portugal	Female	5.74	7.86	4.11
2007	Portugal	Male	28.8	40.0	20.0
2007	Spain	Both	16.6	22.7	11.8
2007	Spain	Female	6.02	8.30	4.26
2007	Spain	Male	27.7	38.6	19.7
2008	Greece	Both	22.6	28.1	17.7
2008	Greece	Female	10.3	13.2	7.87
2008	Greece	Male	36.1	45.1	28.3

2008	Italy	Both	13.4	15.5	11.4
2008	Italy	Female	5.76	6.78	4.89
2008	Italy	Male	21.7	25.1	18.6
2008	Portugal	Both	16.9	23.4	11.9
2008	Portugal	Female	5.76	8.06	4.10
2008	Portugal	Male	29.5	41.4	20.3
2008	Spain	Both	16.3	22.4	11.7
2008	Spain	Female	5.93	8.20	4.20
2008	Spain	Male	27.4	38.2	19.4
2009	Greece	Both	23.6	29.5	18.5
2009	Greece	Female	10.5	13.3	7.96
2009	Greece	Male	38.0	47.9	29.6
2009	Italy	Both	13.5	15.6	11.5
2009	Italy	Female	5.72	6.70	4.87
2009	Italy	Male	22.0	25.5	18.9
2009	Portugal	Both	17.6	24.5	12.3
2009	Portugal	Female	5.90	8.26	4.17
2009	Portugal	Male	30.7	43.0	21.1
2009	Spain	Both	16.3	22.4	11.6
2009	Spain	Female	5.92	8.21	4.19
2009	Spain	Male	27.2	37.9	19.2
2010	Greece	Both	24.5	30.6	19.2
2010	Greece	Female	10.7	13.6	8.15
2010	Greece	Male	39.7	49.9	30.8
2010	Italy	Both	13.7	15.9	11.8
2010	Italy	Female	5.82	6.80	4.94
2010	Italy	Male	22.4	25.9	19.2
2010	Portugal	Both	18.2	25.5	12.6
2010	Portugal	Female	6.10	8.59	4.31
2010	Portugal	Male	31.7	45.0	21.5
2010	Spain	Both	16.2	22.4	11.4
2010	Spain	Female	5.86	8.28	4.13
2010	Spain	Male	27.2	37.9	18.9
2011	Greece	Both	25.9	32.4	20.3
2011	Greece	Female	11.1	14.2	8.36
2011	Greece	Male	42.2	52.7	32.8
2011	Italy	Both	14.0	16.3	12.0
2011	Italy	Female	5.89	6.89	5.01
2011	Italy	Male	22.9	26.6	19.6
2011	Portugal	Both	18.8	26.4	13.1
2011	Portugal	Female	6.11	8.66	4.31
2011	Portugal	Male	33.0	47.0	22.7
2011	Spain	Both	16.3	22.7	11.5
2011	Spain	Female	5.79	8.16	4.08
2011	Spain	Male	27.4	38.4	19.3
2012	Greece	Both	27.3	34.2	21.2
2012	Greece	Female	11.4	14.7	8.60
2012	Greece	Male	44.8	56.0	34.7
2012	Italy	Both	14.3	16.5	12.2
2012	Italy	Female	5.99	6.99	5.08
2012	Italy	Male	23.3	27.2	19.9
2012	Portugal	Both	19.1	27.0	13.3
2012	Portugal	Female	6.17	8.74	4.33

2012	Portugal	Male	33.7	48.1	23.2
2012	Spain	Both	16.4	23.0	11.5
2012	Spain	Female	5.83	8.24	4.09
2012	Spain	Male	27.6	39.0	19.4
2013	Greece	Both	28.8	36.0	22.4
2013	Greece	Female	11.8	15.2	8.91
2013	Greece	Male	47.5	59.5	37.0
2013	Italy	Both	14.2	16.4	12.1
2013	Italy	Female	5.94	6.94	5.04
2013	Italy	Male	23.1	26.8	19.8
2013	Portugal	Both	19.3	27.4	13.4
2013	Portugal	Female	6.19	8.76	4.38
2013	Portugal	Male	34.0	49.2	23.5
2013	Spain	Both	16.4	22.9	11.5
2013	Spain	Female	5.80	8.20	4.05
2013	Spain	Male	27.6	39.4	19.3
2014	Greece	Both	29.1	36.6	22.7
2014	Greece	Female	12.0	15.6	9.04
2014	Greece	Male	48.0	60.3	37.3
2014	Italy	Both	14.0	16.3	11.9
2014	Italy	Female	5.88	6.91	4.97
2014	Italy	Male	22.8	26.6	19.4
2014	Portugal	Both	19.3	27.5	13.4
2014	Portugal	Female	6.14	8.56	4.35
2014	Portugal	Male	34.0	49.4	23.5
2014	Spain	Both	16.4	23.1	11.4
2014	Spain	Female	5.80	8.18	4.09
2014	Spain	Male	27.6	39.5	19.1
2015	Greece	Both	29.4	37.0	23.0
2015	Greece	Female	12.0	15.7	9.09
2015	Greece	Male	48.5	61.2	38.0
2015	Italy	Both	13.8	16.1	11.8
2015	Italy	Female	5.78	6.77	4.86
2015	Italy	Male	22.5	26.2	19.2
2015	Portugal	Both	19.1	27.1	13.2
2015	Portugal	Female	6.06	8.36	4.27
2015	Portugal	Male	33.8	48.7	23.1
2015	Spain	Both	16.4	23.2	11.4
2015	Spain	Female	5.77	8.13	4.09
2015	Spain	Male	27.7	39.7	19.0
2016	Greece	Both	29.6	37.1	23.1
2016	Greece	Female	12.1	15.8	9.16
2016	Greece	Male	48.8	61.1	37.9
2016	Italy	Both	13.0	15.3	11.0
2016	Italy	Female	5.54	6.55	4.67
2016	Italy	Male	21.2	25.0	17.9
2016	Portugal	Both	19.2	27.1	13.2
2016	Portugal	Female	6.12	8.44	4.32
2016	Portugal	Male	34.0	49.0	23.1
2016	Spain	Both	16.1	23.0	11.1
2016	Spain	Female	5.79	8.07	4.07
2016	Spain	Male	27.1	39.1	18.5
2017	Greece	Both	28.9	36.2	22.4

2017	Greece	Female	11.9	15.5	8.99
2017	Greece	Male	47.6	59.9	36.6
2017	Italy	Both	13.7	16.2	11.4
2017	Italy	Female	5.69	6.77	4.76
2017	Italy	Male	22.3	26.6	18.6
2017	Portugal	Both	19.0	27.0	13.2
2017	Portugal	Female	6.08	8.45	4.26
2017	Portugal	Male	33.7	48.5	23.2
2017	Spain	Both	16.0	22.9	11.0
2017	Spain	Female	5.77	8.21	4.04
2017	Spain	Male	26.8	38.8	18.3
2018	Greece	Both	28.4	35.6	22.0
2018	Greece	Female	11.9	15.5	9.10
2018	Greece	Male	46.6	58.6	35.8
2018	Italy	Both	13.9	16.6	11.6
2018	Italy	Female	5.75	6.83	4.81
2018	Italy	Male	22.8	27.4	18.9
2018	Portugal	Both	18.9	26.7	13.1
2018	Portugal	Female	6.10	8.62	4.30
2018	Portugal	Male	33.3	47.7	22.9
2018	Spain	Both	15.7	22.4	11.0
2018	Spain	Female	5.77	8.21	4.03
2018	Spain	Male	26.3	37.7	17.8
2019	Greece	Both	28.0	35.4	21.7
2019	Greece	Female	11.9	15.4	9.10
2019	Greece	Male	45.8	57.8	35.3
2019	Italy	Both	14.2	16.9	11.8
2019	Italy	Female	5.80	6.94	4.79
2019	Italy	Male	23.3	28.1	19.1
2019	Portugal	Both	18.7	26.8	12.9
2019	Portugal	Female	6.04	8.58	4.23
2019	Portugal	Male	33.0	47.4	22.5
2019	Spain	Both	15.6	22.1	10.7
2019	Spain	Female	5.76	8.22	4.03
2019	Spain	Male	25.9	37.3	17.4

Table S29- Age-standardized rates and 95% uncertainty levels (UL) of **years lived with disability** (YLDs) **due to liver cancer due to hepatitis B** per 100,000 population in Greece, Italy, Portugal and Spain from 2000 to 2019 by sex classes (Global Burden of Disease Study 2019).

Year	Country	Sex	YLDs (95% UL)	95% UL (upper)	95% UL (lower)
2000	Greece	Both	0.25	0.35	0.16
2000	Greece	Female	0.12	0.17	0.07
2000	Greece	Male	0.39	0.55	0.25
2000	Italy	Both	0.24	0.33	0.17
2000	Italy	Female	0.10	0.14	0.07
2000	Italy	Male	0.40	0.55	0.28
2000	Portugal	Both	0.12	0.18	0.07
2000	Portugal	Female	0.05	0.07	0.03
2000	Portugal	Male	0.20	0.31	0.12
2000	Spain	Both	0.18	0.27	0.11
2000	Spain	Female	0.07	0.11	0.04
2000	Spain	Male	0.30	0.46	0.18
2001	Greece	Both	0.25	0.35	0.16
2001	Greece	Female	0.12	0.17	0.07
2001	Greece	Male	0.40	0.56	0.26
2001	Italy	Both	0.23	0.32	0.16
2001	Italy	Female	0.09	0.13	0.07
2001	Italy	Male	0.39	0.53	0.27
2001	Portugal	Both	0.12	0.18	0.07
2001	Portugal	Female	0.05	0.07	0.03
2001	Portugal	Male	0.21	0.31	0.12
2001	Spain	Both	0.18	0.28	0.11
2001	Spain	Female	0.07	0.11	0.04
2001	Spain	Male	0.31	0.47	0.19
2002	Greece	Both	0.25	0.35	0.16
2002	Greece	Female	0.12	0.17	0.07
2002	Greece	Male	0.40	0.57	0.26
2002	Italy	Both	0.22	0.30	0.16
2002	Italy	Female	0.09	0.13	0.06
2002	Italy	Male	0.37	0.50	0.26
2002	Portugal	Both	0.12	0.19	0.07
2002	Portugal	Female	0.05	0.07	0.03
2002	Portugal	Male	0.21	0.32	0.13
2002	Spain	Both	0.19	0.29	0.12
2002	Spain	Female	0.07	0.11	0.04
2002	Spain	Male	0.32	0.49	0.19
2003	Greece	Both	0.25	0.36	0.16
2003	Greece	Female	0.12	0.18	0.08
2003	Greece	Male	0.40	0.57	0.26
2003	Italy	Both	0.21	0.29	0.15
2003	Italy	Female	0.09	0.12	0.06
2003	Italy	Male	0.35	0.48	0.24
2003	Portugal	Both	0.13	0.19	0.07
2003	Portugal	Female	0.05	0.07	0.03
2003	Portugal	Male	0.22	0.33	0.13
2003	Spain	Both	0.20	0.30	0.12

2003	Spain	Female	0.07	0.11	0.04
2003	Spain	Male	0.34	0.51	0.20
2004	Greece	Both	0.26	0.36	0.16
2004	Greece	Female	0.12	0.18	0.08
2004	Greece	Male	0.41	0.57	0.26
2004	Italy	Both	0.20	0.27	0.14
2004	Italy	Female	0.08	0.12	0.06
2004	Italy	Male	0.33	0.45	0.23
2004	Portugal	Both	0.13	0.20	0.08
2004	Portugal	Female	0.05	0.07	0.03
2004	Portugal	Male	0.23	0.34	0.13
2004	Spain	Both	0.20	0.31	0.12
2004	Spain	Female	0.07	0.11	0.04
2004	Spain	Male	0.34	0.52	0.21
2005	Greece	Both	0.26	0.37	0.17
2005	Greece	Female	0.12	0.18	0.08
2005	Greece	Male	0.41	0.58	0.27
2005	Italy	Both	0.19	0.27	0.13
2005	Italy	Female	0.08	0.11	0.06
2005	Italy	Male	0.32	0.44	0.22
2005	Portugal	Both	0.13	0.20	0.08
2005	Portugal	Female	0.05	0.07	0.03
2005	Portugal	Male	0.23	0.36	0.14
2005	Spain	Both	0.20	0.31	0.13
2005	Spain	Female	0.07	0.10	0.04
2005	Spain	Male	0.35	0.53	0.21
2006	Greece	Both	0.26	0.37	0.17
2006	Greece	Female	0.12	0.18	0.08
2006	Greece	Male	0.42	0.59	0.27
2006	Italy	Both	0.19	0.27	0.13
2006	Italy	Female	0.08	0.11	0.05
2006	Italy	Male	0.32	0.44	0.22
2006	Portugal	Both	0.14	0.21	0.08
2006	Portugal	Female	0.05	0.07	0.03
2006	Portugal	Male	0.24	0.37	0.14
2006	Spain	Both	0.20	0.31	0.12
2006	Spain	Female	0.07	0.10	0.04
2006	Spain	Male	0.35	0.53	0.21
2007	Greece	Both	0.27	0.38	0.17
2007	Greece	Female	0.13	0.18	0.08
2007	Greece	Male	0.43	0.60	0.28
2007	Italy	Both	0.19	0.26	0.13
2007	Italy	Female	0.08	0.11	0.05
2007	Italy	Male	0.32	0.44	0.22
2007	Portugal	Both	0.14	0.22	0.08
2007	Portugal	Female	0.05	0.07	0.03
2007	Portugal	Male	0.25	0.38	0.15
2007	Spain	Both	0.20	0.31	0.12
2007	Spain	Female	0.07	0.10	0.04
2007	Spain	Male	0.35	0.53	0.21
2008	Greece	Both	0.27	0.39	0.17
2008	Greece	Female	0.13	0.18	0.08
2008	Greece	Male	0.44	0.61	0.28

2008	Italy	Both	0.19	0.26	0.13
2008	Italy	Female	0.08	0.11	0.05
2008	Italy	Male	0.32	0.43	0.22
2008	Portugal	Both	0.15	0.23	0.09
2008	Portugal	Female	0.05	0.08	0.03
2008	Portugal	Male	0.27	0.41	0.15
2008	Spain	Both	0.20	0.31	0.12
2008	Spain	Female	0.06	0.10	0.04
2008	Spain	Male	0.35	0.53	0.22
2009	Greece	Both	0.28	0.39	0.18
2009	Greece	Female	0.13	0.19	0.08
2009	Greece	Male	0.45	0.63	0.28
2009	Italy	Both	0.19	0.26	0.13
2009	Italy	Female	0.08	0.11	0.05
2009	Italy	Male	0.32	0.44	0.22
2009	Portugal	Both	0.16	0.24	0.09
2009	Portugal	Female	0.05	0.08	0.03
2009	Portugal	Male	0.28	0.43	0.16
2009	Spain	Both	0.20	0.31	0.12
2009	Spain	Female	0.06	0.10	0.04
2009	Spain	Male	0.35	0.53	0.22
2010	Greece	Both	0.28	0.40	0.18
2010	Greece	Female	0.13	0.19	0.08
2010	Greece	Male	0.46	0.65	0.29
2010	Italy	Both	0.19	0.27	0.13
2010	Italy	Female	0.08	0.11	0.05
2010	Italy	Male	0.32	0.44	0.22
2010	Portugal	Both	0.16	0.25	0.09
2010	Portugal	Female	0.05	0.08	0.03
2010	Portugal	Male	0.29	0.44	0.17
2010	Spain	Both	0.20	0.31	0.12
2010	Spain	Female	0.06	0.10	0.04
2010	Spain	Male	0.35	0.53	0.22
2011	Greece	Both	0.29	0.42	0.19
2011	Greece	Female	0.13	0.19	0.08
2011	Greece	Male	0.48	0.67	0.31
2011	Italy	Both	0.19	0.27	0.14
2011	Italy	Female	0.08	0.11	0.05
2011	Italy	Male	0.32	0.44	0.23
2011	Portugal	Both	0.16	0.26	0.10
2011	Portugal	Female	0.05	0.08	0.03
2011	Portugal	Male	0.29	0.46	0.17
2011	Spain	Both	0.20	0.31	0.12
2011	Spain	Female	0.06	0.10	0.04
2011	Spain	Male	0.35	0.53	0.22
2012	Greece	Both	0.31	0.44	0.20
2012	Greece	Female	0.13	0.19	0.08
2012	Greece	Male	0.50	0.72	0.32
2012	Italy	Both	0.20	0.27	0.14
2012	Italy	Female	0.08	0.11	0.05
2012	Italy	Male	0.33	0.45	0.23
2012	Portugal	Both	0.17	0.26	0.10
2012	Portugal	Female	0.05	0.09	0.03

2012	Portugal	Male	0.30	0.46	0.18
2012	Spain	Both	0.20	0.31	0.12
2012	Spain	Female	0.06	0.10	0.04
2012	Spain	Male	0.35	0.53	0.21
2013	Greece	Both	0.32	0.45	0.21
2013	Greece	Female	0.14	0.20	0.09
2013	Greece	Male	0.53	0.75	0.34
2013	Italy	Both	0.20	0.27	0.14
2013	Italy	Female	0.08	0.11	0.05
2013	Italy	Male	0.33	0.45	0.23
2013	Portugal	Both	0.17	0.26	0.10
2013	Portugal	Female	0.05	0.08	0.03
2013	Portugal	Male	0.31	0.47	0.18
2013	Spain	Both	0.21	0.31	0.12
2013	Spain	Female	0.06	0.10	0.04
2013	Spain	Male	0.36	0.54	0.22
2014	Greece	Both	0.33	0.47	0.21
2014	Greece	Female	0.14	0.20	0.09
2014	Greece	Male	0.55	0.78	0.34
2014	Italy	Both	0.20	0.27	0.14
2014	Italy	Female	0.08	0.11	0.05
2014	Italy	Male	0.33	0.45	0.23
2014	Portugal	Both	0.17	0.27	0.10
2014	Portugal	Female	0.05	0.08	0.03
2014	Portugal	Male	0.31	0.48	0.18
2014	Spain	Both	0.21	0.32	0.12
2014	Spain	Female	0.06	0.10	0.04
2014	Spain	Male	0.36	0.55	0.22
2015	Greece	Both	0.34	0.48	0.22
2015	Greece	Female	0.14	0.20	0.09
2015	Greece	Male	0.56	0.79	0.35
2015	Italy	Both	0.20	0.27	0.14
2015	Italy	Female	0.08	0.11	0.05
2015	Italy	Male	0.33	0.46	0.23
2015	Portugal	Both	0.18	0.28	0.10
2015	Portugal	Female	0.05	0.09	0.03
2015	Portugal	Male	0.32	0.49	0.18
2015	Spain	Both	0.21	0.32	0.12
2015	Spain	Female	0.06	0.10	0.04
2015	Spain	Male	0.36	0.55	0.22
2016	Greece	Both	0.33	0.48	0.22
2016	Greece	Female	0.14	0.20	0.09
2016	Greece	Male	0.55	0.78	0.36
2016	Italy	Both	0.20	0.27	0.14
2016	Italy	Female	0.08	0.11	0.05
2016	Italy	Male	0.33	0.45	0.23
2016	Portugal	Both	0.18	0.28	0.10
2016	Portugal	Female	0.05	0.09	0.03
2016	Portugal	Male	0.32	0.49	0.19
2016	Spain	Both	0.20	0.31	0.12
2016	Spain	Female	0.06	0.10	0.04
2016	Spain	Male	0.35	0.54	0.21
2017	Greece	Both	0.33	0.49	0.21

2017	Greece	Female	0.14	0.21	0.09
2017	Greece	Male	0.54	0.80	0.34
2017	Italy	Both	0.20	0.28	0.13
2017	Italy	Female	0.08	0.11	0.05
2017	Italy	Male	0.33	0.46	0.22
2017	Portugal	Both	0.18	0.29	0.10
2017	Portugal	Female	0.05	0.09	0.03
2017	Portugal	Male	0.32	0.52	0.18
2017	Spain	Both	0.20	0.32	0.12
2017	Spain	Female	0.06	0.10	0.03
2017	Spain	Male	0.34	0.55	0.20
2018	Greece	Both	0.32	0.49	0.20
2018	Greece	Female	0.14	0.21	0.09
2018	Greece	Male	0.53	0.80	0.33
2018	Italy	Both	0.20	0.28	0.13
2018	Italy	Female	0.08	0.11	0.05
2018	Italy	Male	0.33	0.48	0.22
2018	Portugal	Both	0.18	0.29	0.10
2018	Portugal	Female	0.05	0.09	0.03
2018	Portugal	Male	0.32	0.52	0.18
2018	Spain	Both	0.20	0.32	0.11
2018	Spain	Female	0.06	0.10	0.03
2018	Spain	Male	0.34	0.55	0.20
2019	Greece	Both	0.32	0.50	0.19
2019	Greece	Female	0.14	0.22	0.08
2019	Greece	Male	0.52	0.81	0.31
2019	Italy	Both	0.20	0.31	0.13
2019	Italy	Female	0.08	0.12	0.05
2019	Italy	Male	0.34	0.53	0.21
2019	Portugal	Both	0.18	0.30	0.10
2019	Portugal	Female	0.05	0.10	0.03
2019	Portugal	Male	0.32	0.54	0.18
2019	Spain	Both	0.19	0.33	0.11
2019	Spain	Female	0.06	0.11	0.03
2019	Spain	Male	0.33	0.56	0.19

Table S30- Age-standardized rates and 95% uncertainty levels (UL) of **disability-adjusted life years** (DALYs) **due to liver cancer due to hepatitis B** per 100,000 population in Greece, Italy, Portugal and Spain from 2000 to 2019 by sex classes (Global Burden of Disease Study 2019).

Year	Country	Sex	DALYs (95% UL)	95% UL (upper)	95% UL (lower)
2000	Greece	Both	22.8	28.4	18.1
2000	Greece	Female	10.5	13.5	8.1
2000	Greece	Male	36.5	45.8	28.6
2000	Italy	Both	20.3	23.7	17.3
2000	Italy	Female	8.4	9.9	7.1
2000	Italy	Male	33.6	38.9	28.5
2000	Portugal	Both	14.9	20.4	10.8
2000	Portugal	Female	6.1	8.1	4.4
2000	Portugal	Male	24.9	34.2	17.7
2000	Spain	Both	17.0	23.2	12.1
2000	Spain	Female	7.0	9.7	5.0
2000	Spain	Male	27.9	38.0	19.8
2001	Greece	Both	22.9	28.5	18.3
2001	Greece	Female	10.5	13.5	8.2
2001	Greece	Male	36.7	46.0	28.7
2001	Italy	Both	19.2	22.3	16.4
2001	Italy	Female	8.0	9.3	6.7
2001	Italy	Male	31.6	36.8	27.0
2001	Portugal	Both	15.3	20.7	11.0
2001	Portugal	Female	6.2	8.3	4.5
2001	Portugal	Male	25.4	34.7	18.0
2001	Spain	Both	17.3	23.5	12.3
2001	Spain	Female	7.0	9.5	5.0
2001	Spain	Male	28.4	38.8	20.1
2002	Greece	Both	22.8	28.1	18.1
2002	Greece	Female	10.5	13.4	8.1
2002	Greece	Male	36.4	45.4	28.9
2002	Italy	Both	18.0	20.9	15.3
2002	Italy	Female	7.6	8.9	6.4
2002	Italy	Male	29.4	34.3	25.1
2002	Portugal	Both	14.9	20.2	10.7
2002	Portugal	Female	5.9	7.8	4.3
2002	Portugal	Male	25.0	34.5	17.7
2002	Spain	Both	17.5	23.7	12.6
2002	Spain	Female	6.9	9.5	5.0
2002	Spain	Male	29.0	39.4	20.8
2003	Greece	Both	23.0	28.6	18.3
2003	Greece	Female	10.4	13.4	8.1
2003	Greece	Male	36.8	45.9	29.2
2003	Italy	Both	16.4	19.0	14.0
2003	Italy	Female	7.0	8.2	5.9
2003	Italy	Male	26.9	31.2	22.9
2003	Portugal	Both	15.1	20.5	10.8
2003	Portugal	Female	5.8	7.7	4.2
2003	Portugal	Male	25.6	35.2	18.0
2003	Spain	Both	17.5	23.8	12.6

2003	Spain	Female	6.7	9.2	4.8
2003	Spain	Male	29.0	39.6	20.9
2004	Greece	Both	22.8	28.4	18.1
2004	Greece	Female	10.4	13.2	8.0
2004	Greece	Male	36.5	45.5	28.7
2004	Italy	Both	15.5	18.0	13.1
2004	Italy	Female	6.7	7.9	5.6
2004	Italy	Male	25.2	29.2	21.3
2004	Portugal	Both	15.3	20.9	11.0
2004	Portugal	Female	5.7	7.7	4.1
2004	Portugal	Male	26.1	35.9	18.3
2004	Spain	Both	17.4	23.7	12.6
2004	Spain	Female	6.6	9.0	4.7
2004	Spain	Male	28.9	39.6	20.9
2005	Greece	Both	23.0	28.7	18.3
2005	Greece	Female	10.5	13.5	8.1
2005	Greece	Male	36.8	46.0	28.8
2005	Italy	Both	14.6	16.9	12.4
2005	Italy	Female	6.3	7.4	5.3
2005	Italy	Male	23.6	27.5	20.2
2005	Portugal	Both	15.8	21.7	11.2
2005	Portugal	Female	5.7	7.8	4.1
2005	Portugal	Male	27.1	37.7	18.8
2005	Spain	Both	17.1	23.4	12.4
2005	Spain	Female	6.4	8.8	4.5
2005	Spain	Male	28.5	39.5	20.5
2006	Greece	Both	22.9	28.3	18.0
2006	Greece	Female	10.5	13.6	8.1
2006	Greece	Male	36.4	45.2	28.5
2006	Italy	Both	14.2	16.5	12.1
2006	Italy	Female	6.1	7.1	5.1
2006	Italy	Male	23.0	26.8	19.7
2006	Portugal	Both	16.4	22.4	11.7
2006	Portugal	Female	5.7	7.8	4.1
2006	Portugal	Male	28.2	39.2	19.7
2006	Spain	Both	16.9	23.1	12.2
2006	Spain	Female	6.2	8.6	4.4
2006	Spain	Male	28.2	39.0	20.1
2007	Greece	Both	23.2	28.7	18.3
2007	Greece	Female	10.6	13.6	8.1
2007	Greece	Male	37.1	46.2	29.1
2007	Italy	Both	13.7	16.0	11.8
2007	Italy	Female	5.8	6.9	5.0
2007	Italy	Male	22.4	26.0	19.1
2007	Portugal	Both	16.8	23.1	11.9
2007	Portugal	Female	5.7	7.9	4.1
2007	Portugal	Male	29.0	40.4	20.1
2007	Spain	Both	16.8	23.1	12.0
2007	Spain	Female	6.0	8.4	4.3
2007	Spain	Male	28.1	39.0	20.0
2008	Greece	Both	22.8	28.4	17.9
2008	Greece	Female	10.4	13.4	7.9
2008	Greece	Male	36.5	45.6	28.6

2008	Italy	Both	13.6	15.7	11.6
2008	Italy	Female	5.8	6.8	4.9
2008	Italy	Male	22.0	25.6	18.8
2008	Portugal	Both	17.1	23.6	12.0
2008	Portugal	Female	5.8	8.1	4.1
2008	Portugal	Male	29.7	41.8	20.5
2008	Spain	Both	16.5	22.7	11.9
2008	Spain	Female	6.0	8.3	4.2
2008	Spain	Male	27.7	38.7	19.7
2009	Greece	Both	23.9	29.8	18.7
2009	Greece	Female	10.6	13.5	8.0
2009	Greece	Male	38.5	48.5	30.0
2009	Italy	Both	13.7	15.8	11.7
2009	Italy	Female	5.8	6.8	4.9
2009	Italy	Male	22.3	25.9	19.1
2009	Portugal	Both	17.8	24.7	12.5
2009	Portugal	Female	5.9	8.3	4.2
2009	Portugal	Male	31.0	43.4	21.3
2009	Spain	Both	16.5	22.7	11.7
2009	Spain	Female	5.9	8.3	4.2
2009	Spain	Male	27.6	38.4	19.5
2010	Greece	Both	24.8	31.0	19.4
2010	Greece	Female	10.9	13.8	8.2
2010	Greece	Male	40.2	50.5	31.1
2010	Italy	Both	13.9	16.2	12.0
2010	Italy	Female	5.9	6.9	5.0
2010	Italy	Male	22.7	26.3	19.5
2010	Portugal	Both	18.3	25.7	12.8
2010	Portugal	Female	6.1	8.6	4.3
2010	Portugal	Male	32.0	45.3	21.6
2010	Spain	Both	16.4	22.7	11.5
2010	Spain	Female	5.9	8.3	4.1
2010	Spain	Male	27.5	38.4	19.2
2011	Greece	Both	26.2	32.7	20.5
2011	Greece	Female	11.2	14.3	8.4
2011	Greece	Male	42.7	53.3	33.1
2011	Italy	Both	14.2	16.5	12.2
2011	Italy	Female	5.9	6.9	5.0
2011	Italy	Male	23.3	27.0	19.9
2011	Portugal	Both	18.9	26.7	13.2
2011	Portugal	Female	6.1	8.7	4.3
2011	Portugal	Male	33.3	47.5	22.9
2011	Spain	Both	16.5	23.0	11.6
2011	Spain	Female	5.8	8.2	4.1
2011	Spain	Male	27.8	38.8	19.6
2012	Greece	Both	27.6	34.6	21.4
2012	Greece	Female	11.5	14.8	8.7
2012	Greece	Male	45.3	56.5	35.0
2012	Italy	Both	14.5	16.8	12.4
2012	Italy	Female	6.0	7.0	5.1
2012	Italy	Male	23.6	27.6	20.2
2012	Portugal	Both	19.3	27.2	13.4
2012	Portugal	Female	6.2	8.8	4.3

2012	Portugal	Male	34.0	48.6	23.4
2012	Spain	Both	16.6	23.3	11.7
2012	Spain	Female	5.9	8.3	4.1
2012	Spain	Male	27.9	39.5	19.7
2013	Greece	Both	29.1	36.4	22.7
2013	Greece	Female	11.9	15.3	9.0
2013	Greece	Male	48.1	60.2	37.4
2013	Italy	Both	14.4	16.6	12.3
2013	Italy	Female	6.0	7.0	5.1
2013	Italy	Male	23.4	27.3	20.1
2013	Portugal	Both	19.5	27.7	13.5
2013	Portugal	Female	6.2	8.8	4.4
2013	Portugal	Male	34.3	49.8	23.7
2013	Spain	Both	16.6	23.3	11.6
2013	Spain	Female	5.8	8.3	4.1
2013	Spain	Male	28.0	39.8	19.5
2014	Greece	Both	29.4	37.0	22.9
2014	Greece	Female	12.1	15.8	9.1
2014	Greece	Male	48.5	61.1	37.7
2014	Italy	Both	14.2	16.5	12.1
2014	Italy	Female	5.9	7.0	5.0
2014	Italy	Male	23.1	27.0	19.7
2014	Portugal	Both	19.4	27.8	13.5
2014	Portugal	Female	6.2	8.6	4.3
2014	Portugal	Male	34.4	49.9	23.7
2014	Spain	Both	16.6	23.3	11.6
2014	Spain	Female	5.8	8.2	4.1
2014	Spain	Male	28.0	39.9	19.4
2015	Greece	Both	29.7	37.5	23.2
2015	Greece	Female	12.1	15.9	9.1
2015	Greece	Male	49.1	61.9	38.4
2015	Italy	Both	14.0	16.4	12.0
2015	Italy	Female	5.8	6.8	4.9
2015	Italy	Male	22.8	26.8	19.5
2015	Portugal	Both	19.3	27.4	13.3
2015	Portugal	Female	6.1	8.4	4.3
2015	Portugal	Male	34.1	49.2	23.3
2015	Spain	Both	16.6	23.6	11.5
2015	Spain	Female	5.8	8.2	4.1
2015	Spain	Male	28.0	40.3	19.3
2016	Greece	Both	29.9	37.5	23.3
2016	Greece	Female	12.3	16.1	9.2
2016	Greece	Male	49.4	61.8	38.3
2016	Italy	Both	13.2	15.5	11.2
2016	Italy	Female	5.6	6.6	4.7
2016	Italy	Male	21.5	25.4	18.2
2016	Portugal	Both	19.4	27.3	13.4
2016	Portugal	Female	6.1	8.5	4.3
2016	Portugal	Male	34.4	49.4	23.4
2016	Spain	Both	16.3	23.3	11.3
2016	Spain	Female	5.8	8.1	4.1
2016	Spain	Male	27.5	39.6	18.8
2017	Greece	Both	29.2	36.7	22.6

2017	Greece	Female	12.0	15.7	9.1
2017	Greece	Male	48.1	60.5	37.0
2017	Italy	Both	13.9	16.4	11.6
2017	Italy	Female	5.7	6.8	4.8
2017	Italy	Male	22.6	27.0	18.9
2017	Portugal	Both	19.2	27.2	13.3
2017	Portugal	Female	6.1	8.5	4.3
2017	Portugal	Male	34.0	48.9	23.4
2017	Spain	Both	16.2	23.1	11.1
2017	Spain	Female	5.8	8.3	4.0
2017	Spain	Male	27.2	39.3	18.5
2018	Greece	Both	28.7	36.0	22.3
2018	Greece	Female	12.0	15.7	9.2
2018	Greece	Male	47.1	59.2	36.3
2018	Italy	Both	14.1	16.9	11.8
2018	Italy	Female	5.8	6.9	4.8
2018	Italy	Male	23.1	27.7	19.2
2018	Portugal	Both	19.1	27.0	13.2
2018	Portugal	Female	6.1	8.6	4.3
2018	Portugal	Male	33.6	48.2	23.1
2018	Spain	Both	15.9	22.7	11.1
2018	Spain	Female	5.8	8.3	4.0
2018	Spain	Male	26.7	38.1	18.1
2019	Greece	Both	28.3	35.8	21.9
2019	Greece	Female	12.0	15.6	9.2
2019	Greece	Male	46.3	58.7	35.6
2019	Italy	Both	14.4	17.2	11.9
2019	Italy	Female	5.8	7.0	4.8
2019	Italy	Male	23.6	28.5	19.4
2019	Portugal	Both	18.9	27.0	13.0
2019	Portugal	Female	6.1	8.6	4.2
2019	Portugal	Male	33.3	47.9	22.7
2019	Spain	Both	15.8	22.5	10.9
2019	Spain	Female	5.8	8.3	4.0
2019	Spain	Male	26.3	37.7	17.7

Table S31- Age-standardized rates and 95% uncertainty levels (UL) of **prevalence of liver cancer due to hepatitis C** per 100,000 population in Greece, Italy, Portugal and Spain from 2000 to 2019 by sex classes (Global Burden of Disease Study 2019).

Year	Country	Sex	Prevalence (95% UL)	95% UL (upper)	95% UL (lower)
2000	Greece	Both	0.52	0.69	0.37
2000	Greece	Female	0.63	0.82	0.45
2000	Greece	Male	0.38	0.56	0.25
2000	Italy	Both	5.24	5.81	4.71
2000	Italy	Female	3.30	3.66	2.97
2000	Italy	Male	7.51	8.50	6.62
2000	Portugal	Both	1.21	1.57	0.90
2000	Portugal	Female	0.91	1.10	0.72
2000	Portugal	Male	1.56	2.16	1.08
2000	Spain	Both	3.67	4.50	2.87
2000	Spain	Female	2.14	2.50	1.77
2000	Spain	Male	5.44	6.88	4.06
2001	Greece	Both	0.53	0.69	0.37
2001	Greece	Female	0.63	0.82	0.46
2001	Greece	Male	0.39	0.57	0.25
2001	Italy	Both	5.21	5.77	4.69
2001	Italy	Female	3.29	3.63	2.95
2001	Italy	Male	7.46	8.42	6.60
2001	Portugal	Both	1.23	1.59	0.92
2001	Portugal	Female	0.91	1.10	0.72
2001	Portugal	Male	1.61	2.21	1.12
2001	Spain	Both	3.82	4.66	2.97
2001	Spain	Female	2.20	2.57	1.81
2001	Spain	Male	5.68	7.14	4.25
2002	Greece	Both	0.53	0.71	0.37
2002	Greece	Female	0.64	0.82	0.46
2002	Greece	Male	0.40	0.57	0.26
2002	Italy	Both	5.14	5.68	4.62
2002	Italy	Female	3.24	3.58	2.91
2002	Italy	Male	7.35	8.26	6.49
2002	Portugal	Both	1.25	1.61	0.93
2002	Portugal	Female	0.91	1.10	0.72
2002	Portugal	Male	1.66	2.26	1.14
2002	Spain	Both	3.97	4.84	3.09
2002	Spain	Female	2.26	2.64	1.87
2002	Spain	Male	5.92	7.44	4.44
2003	Greece	Both	0.54	0.72	0.37
2003	Greece	Female	0.65	0.83	0.47
2003	Greece	Male	0.40	0.59	0.26
2003	Italy	Both	5.05	5.57	4.52
2003	Italy	Female	3.18	3.52	2.84
2003	Italy	Male	7.21	8.11	6.37
2003	Portugal	Both	1.27	1.64	0.94
2003	Portugal	Female	0.90	1.10	0.71
2003	Portugal	Male	1.71	2.31	1.19
2003	Spain	Both	4.11	5.03	3.18
2003	Spain	Female	2.32	2.71	1.90

2003	Spain	Male	6.15	7.77	4.61
2004	Greece	Both	0.55	0.73	0.38
2004	Greece	Female	0.66	0.85	0.48
2004	Greece	Male	0.41	0.60	0.26
2004	Italy	Both	4.97	5.49	4.45
2004	Italy	Female	3.13	3.49	2.77
2004	Italy	Male	7.11	7.97	6.28
2004	Portugal	Both	1.30	1.69	0.95
2004	Portugal	Female	0.90	1.10	0.71
2004	Portugal	Male	1.76	2.42	1.21
2004	Spain	Both	4.23	5.21	3.27
2004	Spain	Female	2.36	2.77	1.93
2004	Spain	Male	6.36	8.09	4.69
2005	Greece	Both	0.56	0.74	0.39
2005	Greece	Female	0.67	0.86	0.48
2005	Greece	Male	0.42	0.62	0.27
2005	Italy	Both	4.95	5.48	4.43
2005	Italy	Female	3.11	3.49	2.74
2005	Italy	Male	7.08	7.92	6.25
2005	Portugal	Both	1.32	1.75	0.96
2005	Portugal	Female	0.90	1.11	0.71
2005	Portugal	Male	1.82	2.53	1.24
2005	Spain	Both	4.33	5.32	3.33
2005	Spain	Female	2.38	2.80	1.94
2005	Spain	Male	6.53	8.30	4.81
2006	Greece	Both	0.57	0.76	0.40
2006	Greece	Female	0.68	0.87	0.49
2006	Greece	Male	0.43	0.64	0.27
2006	Italy	Both	4.99	5.50	4.47
2006	Italy	Female	3.11	3.47	2.76
2006	Italy	Male	7.15	7.99	6.31
2006	Portugal	Both	1.37	1.79	1.00
2006	Portugal	Female	0.92	1.12	0.73
2006	Portugal	Male	1.91	2.62	1.30
2006	Spain	Both	4.41	5.42	3.40
2006	Spain	Female	2.39	2.80	1.95
2006	Spain	Male	6.68	8.42	4.94
2007	Greece	Both	0.59	0.78	0.41
2007	Greece	Female	0.70	0.89	0.50
2007	Greece	Male	0.46	0.68	0.29
2007	Italy	Both	5.06	5.58	4.52
2007	Italy	Female	3.13	3.47	2.78
2007	Italy	Male	7.26	8.14	6.40
2007	Portugal	Both	1.45	1.87	1.06
2007	Portugal	Female	0.96	1.15	0.77
2007	Portugal	Male	2.02	2.75	1.40
2007	Spain	Both	4.48	5.48	3.47
2007	Spain	Female	2.38	2.79	1.95
2007	Spain	Male	6.83	8.57	5.11
2008	Greece	Both	0.61	0.81	0.43
2008	Greece	Female	0.72	0.92	0.52
2008	Greece	Male	0.48	0.71	0.30
2008	Italy	Both	5.14	5.68	4.59

2008	Italy	Female	3.16	3.50	2.79
2008	Italy	Male	7.39	8.30	6.50
2008	Portugal	Both	1.53	1.98	1.12
2008	Portugal	Female	1.00	1.20	0.80
2008	Portugal	Male	2.15	2.92	1.50
2008	Spain	Both	4.55	5.57	3.52
2008	Spain	Female	2.38	2.77	1.95
2008	Spain	Male	6.97	8.73	5.19
2009	Greece	Both	0.63	0.84	0.44
2009	Greece	Female	0.74	0.95	0.54
2009	Greece	Male	0.51	0.75	0.32
2009	Italy	Both	5.21	5.78	4.64
2009	Italy	Female	3.18	3.54	2.80
2009	Italy	Male	7.51	8.45	6.58
2009	Portugal	Both	1.62	2.08	1.19
2009	Portugal	Female	1.05	1.25	0.85
2009	Portugal	Male	2.28	3.08	1.59
2009	Spain	Both	4.60	5.68	3.56
2009	Spain	Female	2.37	2.75	1.94
2009	Spain	Male	7.09	8.92	5.29
2010	Greece	Both	0.65	0.88	0.45
2010	Greece	Female	0.75	0.98	0.55
2010	Greece	Male	0.53	0.79	0.33
2010	Italy	Both	5.26	5.87	4.68
2010	Italy	Female	3.19	3.56	2.81
2010	Italy	Male	7.60	8.61	6.64
2010	Portugal	Both	1.69	2.17	1.23
2010	Portugal	Female	1.08	1.30	0.87
2010	Portugal	Male	2.40	3.23	1.66
2010	Spain	Both	4.63	5.74	3.60
2010	Spain	Female	2.35	2.74	1.92
2010	Spain	Male	7.18	9.13	5.37
2011	Greece	Both	0.67	0.91	0.47
2011	Greece	Female	0.77	0.99	0.55
2011	Greece	Male	0.56	0.82	0.35
2011	Italy	Both	5.30	5.90	4.71
2011	Italy	Female	3.21	3.58	2.83
2011	Italy	Male	7.67	8.72	6.70
2011	Portugal	Both	1.75	2.26	1.29
2011	Portugal	Female	1.11	1.33	0.89
2011	Portugal	Male	2.51	3.36	1.75
2011	Spain	Both	4.65	5.73	3.61
2011	Spain	Female	2.34	2.72	1.92
2011	Spain	Male	7.23	9.17	5.36
2012	Greece	Both	0.69	0.94	0.48
2012	Greece	Female	0.77	1.00	0.56
2012	Greece	Male	0.59	0.86	0.37
2012	Italy	Both	5.34	5.94	4.74
2012	Italy	Female	3.22	3.59	2.84
2012	Italy	Male	7.73	8.79	6.77
2012	Portugal	Both	1.82	2.34	1.35
2012	Portugal	Female	1.14	1.36	0.91
2012	Portugal	Male	2.63	3.49	1.84

2012	Spain	Both	4.67	5.75	3.61
2012	Spain	Female	2.33	2.71	1.92
2012	Spain	Male	7.27	9.22	5.36
2013	Greece	Both	0.71	0.96	0.49
2013	Greece	Female	0.78	1.01	0.57
2013	Greece	Male	0.61	0.90	0.39
2013	Italy	Both	5.37	5.98	4.77
2013	Italy	Female	3.22	3.60	2.84
2013	Italy	Male	7.79	8.85	6.84
2013	Portugal	Both	1.89	2.42	1.40
2013	Portugal	Female	1.17	1.40	0.93
2013	Portugal	Male	2.74	3.67	1.92
2013	Spain	Both	4.67	5.75	3.58
2013	Spain	Female	2.31	2.70	1.92
2013	Spain	Male	7.30	9.27	5.35
2014	Greece	Both	0.72	0.98	0.50
2014	Greece	Female	0.79	1.02	0.57
2014	Greece	Male	0.64	0.94	0.40
2014	Italy	Both	5.38	6.00	4.78
2014	Italy	Female	3.22	3.62	2.83
2014	Italy	Male	7.82	8.91	6.81
2014	Portugal	Both	1.95	2.51	1.44
2014	Portugal	Female	1.19	1.43	0.95
2014	Portugal	Male	2.84	3.81	1.96
2014	Spain	Both	4.67	5.77	3.54
2014	Spain	Female	2.30	2.70	1.91
2014	Spain	Male	7.31	9.34	5.34
2015	Greece	Both	0.72	1.00	0.50
2015	Greece	Female	0.79	1.03	0.57
2015	Greece	Male	0.65	0.95	0.40
2015	Italy	Both	5.37	6.03	4.77
2015	Italy	Female	3.20	3.62	2.80
2015	Italy	Male	7.81	8.94	6.78
2015	Portugal	Both	1.99	2.59	1.45
2015	Portugal	Female	1.20	1.45	0.96
2015	Portugal	Male	2.92	3.95	1.97
2015	Spain	Both	4.65	5.75	3.49
2015	Spain	Female	2.28	2.69	1.88
2015	Spain	Male	7.29	9.33	5.32
2016	Greece	Both	0.72	1.00	0.49
2016	Greece	Female	0.79	1.06	0.57
2016	Greece	Male	0.64	0.96	0.40
2016	Italy	Both	5.26	5.97	4.60
2016	Italy	Female	3.11	3.58	2.69
2016	Italy	Male	7.65	8.90	6.47
2016	Portugal	Both	2.02	2.65	1.46
2016	Portugal	Female	1.21	1.47	0.95
2016	Portugal	Male	2.98	4.09	2.04
2016	Spain	Both	4.56	5.71	3.48
2016	Spain	Female	2.25	2.69	1.86
2016	Spain	Male	7.15	9.22	5.23
2017	Greece	Both	0.72	1.02	0.48
2017	Greece	Female	0.79	1.08	0.56

2017	Greece	Male	0.62	0.97	0.38
2017	Italy	Both	5.14	6.06	4.28
2017	Italy	Female	3.03	3.65	2.49
2017	Italy	Male	7.49	9.23	5.99
2017	Portugal	Both	2.04	2.75	1.44
2017	Portugal	Female	1.21	1.53	0.91
2017	Portugal	Male	3.02	4.25	2.00
2017	Spain	Both	4.46	5.82	3.27
2017	Spain	Female	2.22	2.76	1.76
2017	Spain	Male	6.95	9.32	4.87
2018	Greece	Both	0.71	1.02	0.47
2018	Greece	Female	0.80	1.11	0.55
2018	Greece	Male	0.61	0.96	0.37
2018	Italy	Both	5.14	6.12	4.32
2018	Italy	Female	3.03	3.68	2.53
2018	Italy	Male	7.49	9.33	5.96
2018	Portugal	Both	2.02	2.75	1.41
2018	Portugal	Female	1.20	1.54	0.91
2018	Portugal	Male	2.98	4.23	1.97
2018	Spain	Both	4.38	5.78	3.21
2018	Spain	Female	2.20	2.75	1.71
2018	Spain	Male	6.80	9.27	4.82
2019	Greece	Both	0.71	1.05	0.46
2019	Greece	Female	0.80	1.15	0.52
2019	Greece	Male	0.60	0.96	0.35
2019	Italy	Both	5.20	6.64	4.05
2019	Italy	Female	3.05	3.98	2.33
2019	Italy	Male	7.59	10.3	5.40
2019	Portugal	Both	1.97	2.79	1.31
2019	Portugal	Female	1.19	1.59	0.86
2019	Portugal	Male	2.87	4.34	1.83
2019	Spain	Both	4.31	5.91	2.99
2019	Spain	Female	2.20	2.92	1.62
2019	Spain	Male	6.65	9.45	4.47

Table S32- Age-standardized rates and 95% uncertainty levels (UL) of **incidence of liver cancer due to hepatitis C** per 100,000 population in Greece, Italy, Portugal and Spain from 2000 to 2019 by sex classes (Global Burden of Disease Study 2019).

Year	Country	Sex	Incidence (95% UL)	95% UL (upper)	95% UL (lower)
2000	Greece	Both	0.47	0.63	0.33
2000	Greece	Female	0.58	0.75	0.42
2000	Greece	Male	0.34	0.49	0.22
2000	Italy	Both	3.75	4.09	3.42
2000	Italy	Female	2.50	2.69	2.30
2000	Italy	Male	5.28	5.85	4.76
2000	Portugal	Both	1.16	1.49	0.87
2000	Portugal	Female	0.91	1.10	0.72
2000	Portugal	Male	1.46	2.01	1.02
2000	Spain	Both	2.86	3.45	2.26
2000	Spain	Female	1.89	2.18	1.55
2000	Spain	Male	4.03	5.05	3.07
2001	Greece	Both	0.48	0.63	0.34
2001	Greece	Female	0.58	0.75	0.43
2001	Greece	Male	0.34	0.50	0.22
2001	Italy	Both	3.66	3.99	3.35
2001	Italy	Female	2.45	2.63	2.25
2001	Italy	Male	5.15	5.69	4.65
2001	Portugal	Both	1.17	1.50	0.88
2001	Portugal	Female	0.91	1.09	0.72
2001	Portugal	Male	1.49	2.04	1.05
2001	Spain	Both	2.90	3.50	2.31
2001	Spain	Female	1.90	2.20	1.57
2001	Spain	Male	4.10	5.12	3.15
2002	Greece	Both	0.48	0.64	0.34
2002	Greece	Female	0.59	0.76	0.43
2002	Greece	Male	0.34	0.50	0.22
2002	Italy	Both	3.52	3.82	3.20
2002	Italy	Female	2.35	2.53	2.15
2002	Italy	Male	4.94	5.46	4.46
2002	Portugal	Both	1.18	1.50	0.88
2002	Portugal	Female	0.90	1.07	0.72
2002	Portugal	Male	1.51	2.07	1.06
2002	Spain	Both	2.94	3.54	2.33
2002	Spain	Female	1.91	2.21	1.58
2002	Spain	Male	4.17	5.20	3.18
2003	Greece	Both	0.48	0.64	0.34
2003	Greece	Female	0.59	0.76	0.43
2003	Greece	Male	0.34	0.50	0.22
2003	Italy	Both	3.35	3.65	3.05
2003	Italy	Female	2.25	2.41	2.04
2003	Italy	Male	4.72	5.20	4.25
2003	Portugal	Both	1.18	1.51	0.88
2003	Portugal	Female	0.89	1.07	0.70
2003	Portugal	Male	1.53	2.10	1.07
2003	Spain	Both	2.97	3.58	2.36
2003	Spain	Female	1.92	2.23	1.58

2003	Spain	Male	4.23	5.26	3.24
2004	Greece	Both	0.48	0.64	0.34
2004	Greece	Female	0.59	0.76	0.43
2004	Greece	Male	0.34	0.51	0.22
2004	Italy	Both	3.22	3.50	2.92
2004	Italy	Female	2.15	2.32	1.94
2004	Italy	Male	4.53	4.98	4.08
2004	Portugal	Both	1.19	1.54	0.87
2004	Portugal	Female	0.87	1.06	0.70
2004	Portugal	Male	1.56	2.14	1.09
2004	Spain	Both	3.00	3.62	2.37
2004	Spain	Female	1.92	2.24	1.58
2004	Spain	Male	4.29	5.34	3.28
2005	Greece	Both	0.49	0.64	0.34
2005	Greece	Female	0.60	0.77	0.43
2005	Greece	Male	0.35	0.52	0.22
2005	Italy	Both	3.15	3.43	2.86
2005	Italy	Female	2.10	2.28	1.89
2005	Italy	Male	4.44	4.89	4.00
2005	Portugal	Both	1.20	1.57	0.88
2005	Portugal	Female	0.87	1.06	0.68
2005	Portugal	Male	1.60	2.20	1.10
2005	Spain	Both	3.02	3.67	2.38
2005	Spain	Female	1.91	2.24	1.57
2005	Spain	Male	4.33	5.43	3.31
2006	Greece	Both	0.49	0.65	0.35
2006	Greece	Female	0.60	0.77	0.44
2006	Greece	Male	0.36	0.53	0.23
2006	Italy	Both	3.15	3.42	2.85
2006	Italy	Female	2.10	2.27	1.88
2006	Italy	Male	4.43	4.86	4.00
2006	Portugal	Both	1.23	1.59	0.91
2006	Portugal	Female	0.88	1.07	0.70
2006	Portugal	Male	1.65	2.26	1.14
2006	Spain	Both	3.04	3.68	2.42
2006	Spain	Female	1.91	2.22	1.57
2006	Spain	Male	4.37	5.44	3.36
2007	Greece	Both	0.50	0.66	0.35
2007	Greece	Female	0.61	0.78	0.44
2007	Greece	Male	0.37	0.56	0.23
2007	Italy	Both	3.17	3.44	2.87
2007	Italy	Female	2.10	2.27	1.89
2007	Italy	Male	4.45	4.88	4.03
2007	Portugal	Both	1.29	1.64	0.96
2007	Portugal	Female	0.91	1.10	0.73
2007	Portugal	Male	1.74	2.36	1.20
2007	Spain	Both	3.06	3.69	2.44
2007	Spain	Female	1.90	2.21	1.56
2007	Spain	Male	4.42	5.48	3.41
2008	Greece	Both	0.52	0.68	0.36
2008	Greece	Female	0.62	0.80	0.45
2008	Greece	Male	0.39	0.58	0.24
2008	Italy	Both	3.19	3.47	2.89

2008	Italy	Female	2.11	2.28	1.89
2008	Italy	Male	4.49	4.94	4.05
2008	Portugal	Both	1.36	1.73	1.02
2008	Portugal	Female	0.95	1.13	0.77
2008	Portugal	Male	1.84	2.48	1.29
2008	Spain	Both	3.08	3.69	2.45
2008	Spain	Female	1.89	2.18	1.56
2008	Spain	Male	4.47	5.53	3.46
2009	Greece	Both	0.53	0.71	0.37
2009	Greece	Female	0.63	0.81	0.46
2009	Greece	Male	0.41	0.61	0.25
2009	Italy	Both	3.22	3.50	2.91
2009	Italy	Female	2.12	2.29	1.90
2009	Italy	Male	4.52	4.98	4.08
2009	Portugal	Both	1.42	1.80	1.06
2009	Portugal	Female	0.99	1.17	0.80
2009	Portugal	Male	1.95	2.60	1.35
2009	Spain	Both	3.09	3.71	2.46
2009	Spain	Female	1.88	2.17	1.55
2009	Spain	Male	4.51	5.58	3.49
2010	Greece	Both	0.54	0.73	0.38
2010	Greece	Female	0.64	0.83	0.47
2010	Greece	Male	0.42	0.63	0.27
2010	Italy	Both	3.23	3.51	2.93
2010	Italy	Female	2.13	2.30	1.90
2010	Italy	Male	4.55	5.00	4.08
2010	Portugal	Both	1.48	1.88	1.09
2010	Portugal	Female	1.01	1.22	0.82
2010	Portugal	Male	2.03	2.73	1.40
2010	Spain	Both	3.10	3.74	2.46
2010	Spain	Female	1.87	2.17	1.54
2010	Spain	Male	4.55	5.65	3.51
2011	Greece	Both	0.56	0.75	0.39
2011	Greece	Female	0.65	0.83	0.47
2011	Greece	Male	0.44	0.67	0.28
2011	Italy	Both	3.24	3.52	2.93
2011	Italy	Female	2.13	2.30	1.91
2011	Italy	Male	4.55	5.00	4.08
2011	Portugal	Both	1.52	1.92	1.14
2011	Portugal	Female	1.03	1.23	0.83
2011	Portugal	Male	2.11	2.80	1.47
2011	Spain	Both	3.12	3.75	2.49
2011	Spain	Female	1.86	2.16	1.53
2011	Spain	Male	4.59	5.66	3.54
2012	Greece	Both	0.57	0.77	0.40
2012	Greece	Female	0.65	0.84	0.47
2012	Greece	Male	0.47	0.70	0.29
2012	Italy	Both	3.24	3.53	2.93
2012	Italy	Female	2.13	2.30	1.91
2012	Italy	Male	4.56	5.01	4.09
2012	Portugal	Both	1.57	1.99	1.17
2012	Portugal	Female	1.05	1.25	0.85
2012	Portugal	Male	2.19	2.90	1.54

2012	Spain	Both	3.14	3.76	2.50
2012	Spain	Female	1.85	2.14	1.53
2012	Spain	Male	4.63	5.70	3.58
2013	Greece	Both	0.58	0.79	0.40
2013	Greece	Female	0.65	0.83	0.47
2013	Greece	Male	0.49	0.73	0.31
2013	Italy	Both	3.25	3.54	2.93
2013	Italy	Female	2.13	2.30	1.91
2013	Italy	Male	4.56	5.02	4.09
2013	Portugal	Both	1.61	2.04	1.20
2013	Portugal	Female	1.07	1.27	0.86
2013	Portugal	Male	2.27	3.00	1.60
2013	Spain	Both	3.15	3.78	2.50
2013	Spain	Female	1.85	2.13	1.53
2013	Spain	Male	4.66	5.76	3.56
2014	Greece	Both	0.59	0.81	0.41
2014	Greece	Female	0.65	0.84	0.47
2014	Greece	Male	0.51	0.76	0.32
2014	Italy	Both	3.24	3.54	2.92
2014	Italy	Female	2.12	2.30	1.90
2014	Italy	Male	4.56	5.03	4.07
2014	Portugal	Both	1.65	2.11	1.23
2014	Portugal	Female	1.08	1.29	0.87
2014	Portugal	Male	2.34	3.10	1.62
2014	Spain	Both	3.16	3.79	2.51
2014	Spain	Female	1.84	2.13	1.52
2014	Spain	Male	4.69	5.84	3.57
2015	Greece	Both	0.59	0.82	0.41
2015	Greece	Female	0.65	0.85	0.47
2015	Greece	Male	0.52	0.77	0.33
2015	Italy	Both	3.23	3.53	2.90
2015	Italy	Female	2.11	2.29	1.88
2015	Italy	Male	4.54	5.04	4.04
2015	Portugal	Both	1.67	2.16	1.24
2015	Portugal	Female	1.09	1.32	0.87
2015	Portugal	Male	2.39	3.20	1.64
2015	Spain	Both	3.15	3.83	2.49
2015	Spain	Female	1.83	2.15	1.52
2015	Spain	Male	4.69	5.87	3.54
2016	Greece	Both	0.59	0.83	0.40
2016	Greece	Female	0.66	0.87	0.47
2016	Greece	Male	0.51	0.77	0.32
2016	Italy	Both	3.16	3.49	2.81
2016	Italy	Female	2.05	2.28	1.79
2016	Italy	Male	4.45	5.01	3.89
2016	Portugal	Both	1.69	2.19	1.24
2016	Portugal	Female	1.09	1.33	0.87
2016	Portugal	Male	2.43	3.28	1.68
2016	Spain	Both	3.10	3.79	2.45
2016	Spain	Female	1.81	2.13	1.50
2016	Spain	Male	4.61	5.76	3.47
2017	Greece	Both	0.59	0.83	0.40
2017	Greece	Female	0.66	0.90	0.46

2017	Greece	Male	0.50	0.77	0.30
2017	Italy	Both	3.08	3.57	2.63
2017	Italy	Female	1.99	2.34	1.67
2017	Italy	Male	4.35	5.25	3.60
2017	Portugal	Both	1.70	2.24	1.22
2017	Portugal	Female	1.09	1.38	0.83
2017	Portugal	Male	2.44	3.40	1.65
2017	Spain	Both	3.04	3.89	2.28
2017	Spain	Female	1.79	2.18	1.42
2017	Spain	Male	4.49	5.90	3.26
2018	Greece	Both	0.59	0.83	0.39
2018	Greece	Female	0.66	0.91	0.46
2018	Greece	Male	0.49	0.76	0.29
2018	Italy	Both	3.09	3.59	2.64
2018	Italy	Female	1.99	2.33	1.68
2018	Italy	Male	4.36	5.29	3.56
2018	Portugal	Both	1.68	2.24	1.19
2018	Portugal	Female	1.08	1.38	0.82
2018	Portugal	Male	2.41	3.37	1.61
2018	Spain	Both	3.00	3.82	2.21
2018	Spain	Female	1.78	2.21	1.39
2018	Spain	Male	4.41	5.77	3.16
2019	Greece	Both	0.58	0.86	0.38
2019	Greece	Female	0.66	0.95	0.45
2019	Greece	Male	0.48	0.77	0.28
2019	Italy	Both	3.14	3.91	2.50
2019	Italy	Female	2.01	2.55	1.54
2019	Italy	Male	4.43	5.89	3.22
2019	Portugal	Both	1.64	2.29	1.10
2019	Portugal	Female	1.07	1.44	0.77
2019	Portugal	Male	2.32	3.43	1.49
2019	Spain	Both	2.96	3.98	2.07
2019	Spain	Female	1.78	2.32	1.32
2019	Spain	Male	4.32	5.97	2.96

Table S33- Age-standardized rates and 95% uncertainty levels (UL) of **deaths due to liver cancer due to hepatitis C** per 100,000 population in Greece, Italy, Portugal and Spain from 2000 to 2019 by sex classes (Global Burden of Disease Study 2019).

Year	Country	Sex	Deaths (95% UL)	95% UL (upper)	95% UL (lower)
2000	Greece	Both	0.50	0.66	0.35
2000	Greece	Female	0.62	0.81	0.45
2000	Greece	Male	0.35	0.50	0.22
2000	Italy	Both	3.43	3.73	3.14
2000	Italy	Female	2.38	2.53	2.17
2000	Italy	Male	4.78	5.26	4.32
2000	Portugal	Both	1.24	1.58	0.94
2000	Portugal	Female	1.00	1.20	0.80
2000	Portugal	Male	1.53	2.10	1.07
2000	Spain	Both	2.74	3.30	2.19
2000	Spain	Female	1.94	2.25	1.60
2000	Spain	Male	3.74	4.69	2.86
2001	Greece	Both	0.51	0.67	0.36
2001	Greece	Female	0.64	0.82	0.47
2001	Greece	Male	0.35	0.51	0.23
2001	Italy	Both	3.30	3.59	3.02
2001	Italy	Female	2.30	2.45	2.09
2001	Italy	Male	4.58	5.03	4.15
2001	Portugal	Both	1.27	1.61	0.95
2001	Portugal	Female	1.02	1.22	0.82
2001	Portugal	Male	1.56	2.14	1.08
2001	Spain	Both	2.77	3.34	2.20
2001	Spain	Female	1.95	2.25	1.60
2001	Spain	Male	3.79	4.72	2.88
2002	Greece	Both	0.51	0.67	0.36
2002	Greece	Female	0.64	0.82	0.46
2002	Greece	Male	0.35	0.51	0.23
2002	Italy	Both	3.17	3.44	2.88
2002	Italy	Female	2.21	2.36	2.00
2002	Italy	Male	4.40	4.83	3.99
2002	Portugal	Both	1.21	1.54	0.91
2002	Portugal	Female	0.96	1.15	0.77
2002	Portugal	Male	1.52	2.07	1.05
2002	Spain	Both	2.80	3.36	2.23
2002	Spain	Female	1.95	2.27	1.61
2002	Spain	Male	3.86	4.80	2.95
2003	Greece	Both	0.51	0.67	0.36
2003	Greece	Female	0.64	0.82	0.46
2003	Greece	Male	0.35	0.52	0.23
2003	Italy	Both	2.97	3.24	2.69
2003	Italy	Female	2.06	2.22	1.85
2003	Italy	Male	4.12	4.55	3.72
2003	Portugal	Both	1.22	1.55	0.90
2003	Portugal	Female	0.94	1.14	0.75
2003	Portugal	Male	1.55	2.10	1.08

2003	Spain	Both	2.80	3.37	2.22
2003	Spain	Female	1.93	2.25	1.59
2003	Spain	Male	3.87	4.80	2.97
2004	Greece	Both	0.50	0.67	0.36
2004	Greece	Female	0.63	0.81	0.45
2004	Greece	Male	0.34	0.51	0.22
2004	Italy	Both	2.85	3.09	2.57
2004	Italy	Female	1.99	2.14	1.77
2004	Italy	Male	3.95	4.34	3.57
2004	Portugal	Both	1.23	1.57	0.91
2004	Portugal	Female	0.95	1.13	0.75
2004	Portugal	Male	1.59	2.16	1.09
2004	Spain	Both	2.78	3.34	2.21
2004	Spain	Female	1.92	2.22	1.58
2004	Spain	Male	3.85	4.80	2.95
2005	Greece	Both	0.50	0.66	0.35
2005	Greece	Female	0.63	0.80	0.45
2005	Greece	Male	0.35	0.51	0.22
2005	Italy	Both	2.73	2.96	2.47
2005	Italy	Female	1.89	2.04	1.69
2005	Italy	Male	3.79	4.16	3.44
2005	Portugal	Both	1.26	1.60	0.93
2005	Portugal	Female	0.96	1.14	0.76
2005	Portugal	Male	1.63	2.23	1.13
2005	Spain	Both	2.74	3.30	2.17
2005	Spain	Female	1.89	2.19	1.55
2005	Spain	Male	3.79	4.74	2.89
2006	Greece	Both	0.50	0.66	0.35
2006	Greece	Female	0.62	0.80	0.45
2006	Greece	Male	0.35	0.51	0.22
2006	Italy	Both	2.67	2.89	2.42
2006	Italy	Female	1.85	2.00	1.64
2006	Italy	Male	3.71	4.06	3.36
2006	Portugal	Both	1.29	1.64	0.97
2006	Portugal	Female	0.96	1.14	0.77
2006	Portugal	Male	1.70	2.31	1.19
2006	Spain	Both	2.71	3.26	2.16
2006	Spain	Female	1.86	2.16	1.53
2006	Spain	Male	3.75	4.68	2.87
2007	Greece	Both	0.51	0.68	0.36
2007	Greece	Female	0.64	0.82	0.47
2007	Greece	Male	0.36	0.54	0.23
2007	Italy	Both	2.64	2.86	2.38
2007	Italy	Female	1.82	1.97	1.62
2007	Italy	Male	3.66	4.02	3.29
2007	Portugal	Both	1.33	1.67	0.99
2007	Portugal	Female	0.98	1.17	0.79
2007	Portugal	Male	1.76	2.37	1.21
2007	Spain	Both	2.72	3.26	2.17
2007	Spain	Female	1.84	2.16	1.51
2007	Spain	Male	3.78	4.69	2.93
2008	Greece	Both	0.52	0.69	0.37
2008	Greece	Female	0.65	0.83	0.48

2008	Greece	Male	0.37	0.55	0.23
2008	Italy	Both	2.66	2.88	2.40
2008	Italy	Female	1.85	1.99	1.64
2008	Italy	Male	3.67	4.03	3.31
2008	Portugal	Both	1.38	1.73	1.03
2008	Portugal	Female	1.00	1.19	0.81
2008	Portugal	Male	1.84	2.46	1.28
2008	Spain	Both	2.73	3.27	2.20
2008	Spain	Female	1.84	2.16	1.51
2008	Spain	Male	3.80	4.70	2.96
2009	Greece	Both	0.54	0.71	0.38
2009	Greece	Female	0.65	0.83	0.48
2009	Greece	Male	0.39	0.58	0.25
2009	Italy	Both	2.70	2.92	2.43
2009	Italy	Female	1.87	2.01	1.65
2009	Italy	Male	3.73	4.08	3.36
2009	Portugal	Both	1.43	1.81	1.07
2009	Portugal	Female	1.03	1.22	0.83
2009	Portugal	Male	1.93	2.58	1.35
2009	Spain	Both	2.75	3.29	2.21
2009	Spain	Female	1.84	2.15	1.51
2009	Spain	Male	3.86	4.77	3.01
2010	Greece	Both	0.55	0.73	0.39
2010	Greece	Female	0.66	0.84	0.47
2010	Greece	Male	0.41	0.62	0.26
2010	Italy	Both	2.77	2.99	2.50
2010	Italy	Female	1.92	2.06	1.69
2010	Italy	Male	3.82	4.17	3.43
2010	Portugal	Both	1.50	1.89	1.12
2010	Portugal	Female	1.07	1.28	0.87
2010	Portugal	Male	2.03	2.69	1.41
2010	Spain	Both	2.77	3.32	2.23
2010	Spain	Female	1.84	2.14	1.49
2010	Spain	Male	3.91	4.82	3.03
2011	Greece	Both	0.56	0.75	0.39
2011	Greece	Female	0.66	0.85	0.48
2011	Greece	Male	0.44	0.66	0.28
2011	Italy	Both	2.82	3.05	2.53
2011	Italy	Female	1.94	2.09	1.71
2011	Italy	Male	3.90	4.27	3.51
2011	Portugal	Both	1.55	1.96	1.16
2011	Portugal	Female	1.09	1.30	0.89
2011	Portugal	Male	2.12	2.82	1.48
2011	Spain	Both	2.80	3.36	2.25
2011	Spain	Female	1.83	2.13	1.49
2011	Spain	Male	3.96	4.87	3.10
2012	Greece	Both	0.58	0.78	0.40
2012	Greece	Female	0.67	0.87	0.48
2012	Greece	Male	0.47	0.70	0.30
2012	Italy	Both	2.85	3.09	2.55
2012	Italy	Female	1.97	2.12	1.73
2012	Italy	Male	3.93	4.31	3.53
2012	Portugal	Both	1.60	2.03	1.21

2012	Portugal	Female	1.12	1.33	0.90
2012	Portugal	Male	2.20	2.92	1.55
2012	Spain	Both	2.82	3.39	2.27
2012	Spain	Female	1.84	2.13	1.50
2012	Spain	Male	4.01	4.94	3.12
2013	Greece	Both	0.60	0.81	0.41
2013	Greece	Female	0.68	0.87	0.49
2013	Greece	Male	0.50	0.74	0.31
2013	Italy	Both	2.80	3.04	2.52
2013	Italy	Female	1.94	2.09	1.71
2013	Italy	Male	3.86	4.23	3.47
2013	Portugal	Both	1.65	2.09	1.24
2013	Portugal	Female	1.14	1.36	0.92
2013	Portugal	Male	2.28	3.02	1.62
2013	Spain	Both	2.82	3.38	2.26
2013	Spain	Female	1.83	2.14	1.50
2013	Spain	Male	4.00	4.94	3.12
2014	Greece	Both	0.60	0.81	0.41
2014	Greece	Female	0.67	0.87	0.49
2014	Greece	Male	0.50	0.74	0.31
2014	Italy	Both	2.75	2.99	2.46
2014	Italy	Female	1.91	2.06	1.66
2014	Italy	Male	3.78	4.15	3.37
2014	Portugal	Both	1.67	2.12	1.25
2014	Portugal	Female	1.15	1.38	0.93
2014	Portugal	Male	2.33	3.07	1.62
2014	Spain	Both	2.81	3.39	2.24
2014	Spain	Female	1.81	2.12	1.49
2014	Spain	Male	4.01	4.97	3.11
2015	Greece	Both	0.59	0.81	0.41
2015	Greece	Female	0.66	0.86	0.48
2015	Greece	Male	0.50	0.75	0.32
2015	Italy	Both	2.70	2.93	2.41
2015	Italy	Female	1.86	2.01	1.64
2015	Italy	Male	3.70	4.07	3.33
2015	Portugal	Both	1.69	2.15	1.26
2015	Portugal	Female	1.14	1.37	0.92
2015	Portugal	Male	2.38	3.16	1.65
2015	Spain	Both	2.82	3.41	2.24
2015	Spain	Female	1.80	2.12	1.49
2015	Spain	Male	4.04	5.02	3.11
2016	Greece	Both	0.60	0.82	0.42
2016	Greece	Female	0.67	0.88	0.49
2016	Greece	Male	0.51	0.75	0.32
2016	Italy	Both	2.53	2.76	2.25
2016	Italy	Female	1.75	1.90	1.53
2016	Italy	Male	3.46	3.82	3.07
2016	Portugal	Both	1.72	2.19	1.28
2016	Portugal	Female	1.15	1.40	0.92
2016	Portugal	Male	2.41	3.19	1.69
2016	Spain	Both	2.79	3.37	2.22
2016	Spain	Female	1.80	2.10	1.49
2016	Spain	Male	3.99	4.93	3.08

2017	Greece	Both	0.59	0.80	0.41
2017	Greece	Female	0.66	0.86	0.48
2017	Greece	Male	0.49	0.73	0.31
2017	Italy	Both	2.60	2.87	2.30
2017	Italy	Female	1.78	1.93	1.55
2017	Italy	Male	3.59	4.02	3.16
2017	Portugal	Both	1.69	2.16	1.26
2017	Portugal	Female	1.13	1.38	0.90
2017	Portugal	Male	2.38	3.18	1.66
2017	Spain	Both	2.75	3.33	2.19
2017	Spain	Female	1.78	2.10	1.46
2017	Spain	Male	3.91	4.87	2.98
2018	Greece	Both	0.59	0.80	0.41
2018	Greece	Female	0.67	0.88	0.49
2018	Greece	Male	0.48	0.71	0.30
2018	Italy	Both	2.63	2.91	2.33
2018	Italy	Female	1.79	1.96	1.57
2018	Italy	Male	3.63	4.08	3.15
2018	Portugal	Both	1.66	2.11	1.22
2018	Portugal	Female	1.13	1.38	0.90
2018	Portugal	Male	2.31	3.08	1.61
2018	Spain	Both	2.72	3.30	2.14
2018	Spain	Female	1.79	2.10	1.46
2018	Spain	Male	3.82	4.76	2.88
2019	Greece	Both	0.58	0.80	0.40
2019	Greece	Female	0.67	0.89	0.48
2019	Greece	Male	0.47	0.70	0.30
2019	Italy	Both	2.66	2.93	2.33
2019	Italy	Female	1.81	1.97	1.57
2019	Italy	Male	3.67	4.16	3.16
2019	Portugal	Both	1.63	2.08	1.20
2019	Portugal	Female	1.12	1.38	0.88
2019	Portugal	Male	2.26	3.01	1.57
2019	Spain	Both	2.70	3.30	2.10
2019	Spain	Female	1.79	2.11	1.45
2019	Spain	Male	3.77	4.75	2.82

Table S34- Age-standardized rates and 95% uncertainty levels (UL) of **years of life lost** (YLLs) **due to liver cancer due to hepatitis C** per 100,000 population in Greece, Italy, Portugal and Spain from 2000 to 2019 by sex classes (Global Burden of Disease Study 2019).

Year	Country	Sex	YLLs (95% UL)	95% UL (upper)	95% UL (lower)
2000	Greece	Both	8.0	10.6	5.7
2000	Greece	Female	9.5	12.3	6.9
2000	Greece	Male	6.13	8.8	3.9
2000	Italy	Both	70.4	77.1	63.8
2000	Italy	Female	45.0	48.2	41.6
2000	Italy	Male	100.5	111.8	89.9
2000	Portugal	Both	25.5	33.1	18.8
2000	Portugal	Female	19.2	23.3	15.1
2000	Portugal	Male	33.0	45.8	22.7
2000	Spain	Both	55.1	67.0	43.5
2000	Spain	Female	35.8	41.5	29.9
2000	Spain	Male	77.5	97.7	58.6
2001	Greece	Both	8.1	10.7	5.7
2001	Greece	Female	9.7	12.4	7.1
2001	Greece	Male	6.1	8.9	3.9
2001	Italy	Both	67.2	73.5	60.9
2001	Italy	Female	43.3	46.2	39.8
2001	Italy	Male	95.7	106.3	85.6
2001	Portugal	Both	26.1	34.0	19.3
2001	Portugal	Female	19.6	23.8	15.4
2001	Portugal	Male	33.8	46.7	23.4
2001	Spain	Both	55.6	67.3	43.8
2001	Spain	Female	35.8	41.4	29.6
2001	Spain	Male	78.5	98.9	59.2
2002	Greece	Both	8.1	10.7	5.7
2002	Greece	Female	9.6	12.3	7.0
2002	Greece	Male	6.19	8.9	3.9
2002	Italy	Both	64.3	70.2	58.3
2002	Italy	Female	41.5	44.3	38.2
2002	Italy	Male	91.3	101.3	81.5
2002	Portugal	Both	25.2	32.8	18.5
2002	Portugal	Female	18.5	22.5	14.6
2002	Portugal	Male	33.0	45.8	22.6
2002	Spain	Both	56.1	67.9	44.0
2002	Spain	Female	35.7	41.5	29.5
2002	Spain	Male	79.6	100.1	59.7
2003	Greece	Both	8.1	10.8	5.6
2003	Greece	Female	9.5	12.3	7.0
2003	Greece	Male	6.2	9.0	3.9
2003	Italy	Both	59.9	65.8	54.2
2003	Italy	Female	38.5	41.4	35.3
2003	Italy	Male	85.3	94.9	76.2
2003	Portugal	Both	25.4	33.2	18.7
2003	Portugal	Female	18.2	22.3	14.3
2003	Portugal	Male	33.8	46.6	23.1
2003	Spain	Both	55.7	67.9	43.7
2003	Spain	Female	35.1	41.0	29.0

2003	Spain	Male	79.5	99.7	59.6
2004	Greece	Both	8.0	10.7	5.5
2004	Greece	Female	9.4	12.2	6.8
2004	Greece	Male	6.18	9.0	3.9
2004	Italy	Both	57.4	62.8	52.1
2004	Italy	Female	37.1	39.8	33.8
2004	Italy	Male	81.5	90.1	73.0
2004	Portugal	Both	25.7	33.6	19.0
2004	Portugal	Female	18.1	22.0	14.2
2004	Portugal	Male	34.5	47.8	23.8
2004	Spain	Both	55.3	67.5	43.3
2004	Spain	Female	34.7	40.6	28.7
2004	Spain	Male	79.0	99.2	59.2
2005	Greece	Both	8.0	10.8	5.5
2005	Greece	Female	9.5	12.3	6.8
2005	Greece	Male	6.2	9.1	3.8
2005	Italy	Both	54.9	59.7	50.0
2005	Italy	Female	35.2	37.8	32.2
2005	Italy	Male	78.1	86.1	70.2
2005	Portugal	Both	26.4	34.5	19.5
2005	Portugal	Female	18.3	22.2	14.5
2005	Portugal	Male	35.9	49.7	24.2
2005	Spain	Both	54.5	66.4	42.8
2005	Spain	Female	34.1	39.7	28.0
2005	Spain	Male	77.9	97.7	58.5
2006	Greece	Both	8.0	10.8	5.6
2006	Greece	Female	9.5	12.3	6.9
2006	Greece	Male	6.23	9.2	3.9
2006	Italy	Both	53.6	58.4	48.6
2006	Italy	Female	34.3	36.8	31.2
2006	Italy	Male	76.3	84.0	68.7
2006	Portugal	Both	27.3	35.7	20.1
2006	Portugal	Female	18.4	22.3	14.6
2006	Portugal	Male	37.6	51.5	25.6
2006	Spain	Both	54.1	65.8	42.5
2006	Spain	Female	33.5	38.9	27.4
2006	Spain	Male	77.5	96.9	58.5
2007	Greece	Both	8.3	11.2	5.8
2007	Greece	Female	9.7	12.5	7.1
2007	Greece	Male	6.52	9.7	4.0
2007	Italy	Both	52.8	57.5	47.8
2007	Italy	Female	33.7	36.2	30.5
2007	Italy	Male	75.1	82.9	67.4
2007	Portugal	Both	28.1	36.4	20.6
2007	Portugal	Female	18.7	22.6	15.1
2007	Portugal	Male	38.9	53.2	26.8
2007	Spain	Both	54.4	66.0	42.6
2007	Spain	Female	33.2	38.5	27.1
2007	Spain	Male	78.5	98.0	59.1
2008	Greece	Both	8.4	11.3	5.9
2008	Greece	Female	9.9	12.8	7.2
2008	Greece	Male	6.62	9.9	4.1
2008	Italy	Both	53.1	57.8	48.2

2008	Italy	Female	34.1	36.5	31.0
2008	Italy	Male	75.1	82.9	67.5
2008	Portugal	Both	29.0	37.5	21.1
2008	Portugal	Female	19.2	23.0	15.4
2008	Portugal	Male	40.6	54.8	28.1
2008	Spain	Both	54.9	66.2	43.0
2008	Spain	Female	33.3	38.5	27.2
2008	Spain	Male	79.3	98.7	59.9
2009	Greece	Both	8.8	11.8	6.1
2009	Greece	Female	10.1	13.1	7.4
2009	Greece	Male	7.1	10.7	4.4
2009	Italy	Both	53.9	58.6	48.9
2009	Italy	Female	34.4	36.9	31.3
2009	Italy	Male	76.4	84.4	68.5
2009	Portugal	Both	30.4	39.1	22.2
2009	Portugal	Female	19.9	24.0	15.9
2009	Portugal	Male	42.8	57.8	29.6
2009	Spain	Both	55.7	67.6	43.1
2009	Spain	Female	33.5	38.8	27.6
2009	Spain	Male	80.8	100.6	60.7
2010	Greece	Both	9.1	12.3	6.3
2010	Greece	Female	10.3	13.5	7.5
2010	Greece	Male	7.5	11.3	4.7
2010	Italy	Both	55.1	60.0	50.0
2010	Italy	Female	35.1	37.7	31.9
2010	Italy	Male	78.2	86.3	70.4
2010	Portugal	Both	31.8	41.0	23.3
2010	Portugal	Female	20.7	25.0	16.5
2010	Portugal	Male	44.8	60.4	30.8
2010	Spain	Both	56.3	68.2	43.4
2010	Spain	Female	33.4	38.6	27.4
2010	Spain	Male	81.9	102.3	61.4
2011	Greece	Both	9.4	12.9	6.5
2011	Greece	Female	10.6	13.8	7.6
2011	Greece	Male	8.0	12.0	5.0
2011	Italy	Both	56.3	61.4	51.2
2011	Italy	Female	35.6	38.1	32.4
2011	Italy	Male	80.1	88.2	71.8
2011	Portugal	Both	33.1	42.8	24.2
2011	Portugal	Female	21.2	25.5	16.8
2011	Portugal	Male	47.0	63.7	32.4
2011	Spain	Both	56.7	68.9	43.6
2011	Spain	Female	33.1	38.4	27.1
2011	Spain	Male	83.1	103.8	62.4
2012	Greece	Both	9.8	13.4	6.7
2012	Greece	Female	10.8	14.2	7.8
2012	Greece	Male	8.5	12.7	5.3
2012	Italy	Both	57.0	62.3	51.8
2012	Italy	Female	36.2	38.7	32.9
2012	Italy	Male	80.9	89.5	72.3
2012	Portugal	Both	34.3	44.5	25.0
2012	Portugal	Female	21.7	26.3	17.3
2012	Portugal	Male	49.0	66.3	33.6

2012	Spain	Both	57.2	69.4	44.1
2012	Spain	Female	33.3	38.5	27.5
2012	Spain	Male	84.0	105.1	62.4
2013	Greece	Both	10.2	13.9	6.9
2013	Greece	Female	11.1	14.5	7.9
2013	Greece	Male	9.1	13.6	5.7
2013	Italy	Both	56.2	61.5	51.0
2013	Italy	Female	35.6	38.2	32.4
2013	Italy	Male	79.7	88.1	71.3
2013	Portugal	Both	35.2	45.9	25.6
2013	Portugal	Female	22.1	26.8	17.6
2013	Portugal	Male	50.6	68.5	35.0
2013	Spain	Both	57.2	69.6	44.1
2013	Spain	Female	33.2	38.5	27.4
2013	Spain	Male	84.0	105.0	62.1
2014	Greece	Both	10.2	14.0	6.9
2014	Greece	Female	11.2	14.5	8.0
2014	Greece	Male	9.1	13.6	5.6
2014	Italy	Both	55.2	60.3	49.8
2014	Italy	Female	35.1	37.8	31.6
2014	Italy	Male	78.0	86.6	69.5
2014	Portugal	Both	35.7	46.3	25.9
2014	Portugal	Female	22.2	26.9	17.6
2014	Portugal	Male	51.5	69.9	35.1
2014	Spain	Both	57.1	69.5	43.9
2014	Spain	Female	33.0	38.3	27.3
2014	Spain	Male	84.0	105.4	62.5
2015	Greece	Both	10.2	14.1	6.9
2015	Greece	Female	11.1	14.5	7.8
2015	Greece	Male	9.2	13.8	5.7
2015	Italy	Both	54.1	59.1	48.7
2015	Italy	Female	34.4	37.1	31.2
2015	Italy	Male	76.3	84.4	68.0
2015	Portugal	Both	36.0	47.2	26.2
2015	Portugal	Female	22.2	27.1	17.5
2015	Portugal	Male	52.2	71.4	35.0
2015	Spain	Both	57.2	69.8	44.1
2015	Spain	Female	32.9	38.3	27.3
2015	Spain	Male	84.5	106.4	62.6
2016	Greece	Both	10.3	14.3	7.0
2016	Greece	Female	11.3	14.7	8.0
2016	Greece	Male	9.2	13.9	5.7
2016	Italy	Both	50.7	55.6	45.6
2016	Italy	Female	32.6	35.1	29.4
2016	Italy	Male	71.3	79.5	62.8
2016	Portugal	Both	36.5	48.0	26.5
2016	Portugal	Female	22.3	27.3	17.5
2016	Portugal	Male	53.1	72.8	36.1
2016	Spain	Both	56.5	69.3	43.7
2016	Spain	Female	32.7	38.1	27.1
2016	Spain	Male	83.1	105.1	61.6
2017	Greece	Both	10.1	14.1	6.9
2017	Greece	Female	11.1	14.6	7.9

2017	Greece	Male	9.0	13.5	5.5
2017	Italy	Both	52.3	58.0	46.5
2017	Italy	Female	33.0	35.8	29.6
2017	Italy	Male	74.1	83.6	64.4
2017	Portugal	Both	35.9	47.2	25.5
2017	Portugal	Female	21.9	26.9	17.1
2017	Portugal	Male	52.3	71.5	35.3
2017	Spain	Both	55.7	68.6	43.0
2017	Spain	Female	32.4	38.1	26.8
2017	Spain	Male	81.7	104.2	60.0
2018	Greece	Both	10.1	13.9	6.8
2018	Greece	Female	11.1	14.6	8.0
2018	Greece	Male	8.7	13.2	5.4
2018	Italy	Both	52.7	58.6	46.6
2018	Italy	Female	33.3	36.2	29.7
2018	Italy	Male	74.7	84.9	64.6
2018	Portugal	Both	35.1	46.2	25.0
2018	Portugal	Female	21.7	26.8	17.0
2018	Portugal	Male	50.7	69.7	33.9
2018	Spain	Both	54.8	67.7	42.0
2018	Spain	Female	32.4	38.0	26.7
2018	Spain	Male	79.6	101.7	57.8
2019	Greece	Both	9.9	13.9	6.8
2019	Greece	Female	11.0	14.6	7.9
2019	Greece	Male	8.5	12.8	5.3
2019	Italy	Both	53.1	59.3	46.6
2019	Italy	Female	33.4	36.4	30.0
2019	Italy	Male	75.2	86.0	64.2
2019	Portugal	Both	34.4	45.8	24.4
2019	Portugal	Female	21.5	26.7	16.7
2019	Portugal	Male	49.4	69.0	33.3
2019	Spain	Both	54.2	67.2	41.4
2019	Spain	Female	32.4	38.3	26.3
2019	Spain	Male	78.4	101.8	57.4

Table S35- Age-standardized rates and 95% uncertainty levels (UL) of **years lived with disability** (YLDs) **due to liver cancer due to hepatitis C** per 100,000 population in Greece, Italy, Portugal and Spain from 2000 to 2019 by sex classes (Global Burden of Disease Study 2019).

Year	Country	Sex	YLDs (95% UL)	95% UL (upper)	95% UL (lower)
2000	Greece	Both	0.11	0.16	0.06
2000	Greece	Female	0.13	0.19	0.08
2000	Greece	Male	0.07	0.12	0.04
2000	Italy	Both	0.89	1.18	0.63
2000	Italy	Female	0.59	0.78	0.41
2000	Italy	Male	1.25	1.65	0.88
2000	Portugal	Both	0.26	0.37	0.16
2000	Portugal	Female	0.20	0.28	0.13
2000	Portugal	Male	0.33	0.50	0.20
2000	Spain	Both	0.67	0.93	0.43
2000	Spain	Female	0.43	0.59	0.28
2000	Spain	Male	0.96	1.36	0.60
2001	Greece	Both	0.11	0.16	0.06
2001	Greece	Female	0.13	0.19	0.08
2001	Greece	Male	0.08	0.12	0.04
2001	Italy	Both	0.87	1.16	0.62
2001	Italy	Female	0.58	0.77	0.41
2001	Italy	Male	1.22	1.63	0.86
2001	Portugal	Both	0.26	0.38	0.16
2001	Portugal	Female	0.20	0.28	0.13
2001	Portugal	Male	0.34	0.50	0.20
2001	Spain	Both	0.69	0.95	0.44
2001	Spain	Female	0.44	0.59	0.29
2001	Spain	Male	0.99	1.39	0.61
2002	Greece	Both	0.11	0.16	0.06
2002	Greece	Female	0.13	0.19	0.08
2002	Greece	Male	0.08	0.12	0.04
2002	Italy	Both	0.85	1.13	0.59
2002	Italy	Female	0.56	0.75	0.39
2002	Italy	Male	1.19	1.58	0.83
2002	Portugal	Both	0.26	0.38	0.16
2002	Portugal	Female	0.20	0.28	0.13
2002	Portugal	Male	0.34	0.51	0.21
2002	Spain	Both	0.70	0.97	0.46
2002	Spain	Female	0.44	0.60	0.29
2002	Spain	Male	1.01	1.42	0.64
2003	Greece	Both	0.11	0.16	0.06
2003	Greece	Female	0.13	0.19	0.08
2003	Greece	Male	0.08	0.12	0.04
2003	Italy	Both	0.82	1.09	0.58
2003	Italy	Female	0.54	0.73	0.38
2003	Italy	Male	1.15	1.53	0.80
2003	Portugal	Both	0.27	0.38	0.17
2003	Portugal	Female	0.20	0.27	0.13
2003	Portugal	Male	0.35	0.52	0.21
2003	Spain	Both	0.71	1.00	0.46
2003	Spain	Female	0.44	0.61	0.30

2003	Spain	Male	1.03	1.46	0.65
2004	Greece	Both	0.11	0.16	0.06
2004	Greece	Female	0.13	0.19	0.08
2004	Greece	Male	0.08	0.13	0.04
2004	Italy	Both	0.79	1.06	0.56
2004	Italy	Female	0.52	0.70	0.37
2004	Italy	Male	1.11	1.47	0.78
2004	Portugal	Both	0.27	0.39	0.17
2004	Portugal	Female	0.19	0.27	0.12
2004	Portugal	Male	0.36	0.53	0.21
2004	Spain	Both	0.72	1.00	0.47
2004	Spain	Female	0.45	0.61	0.30
2004	Spain	Male	1.04	1.49	0.66
2005	Greece	Both	0.11	0.16	0.06
2005	Greece	Female	0.13	0.19	0.08
2005	Greece	Male	0.08	0.13	0.04
2005	Italy	Both	0.78	1.04	0.55
2005	Italy	Female	0.51	0.69	0.37
2005	Italy	Male	1.09	1.44	0.76
2005	Portugal	Both	0.27	0.40	0.17
2005	Portugal	Female	0.19	0.27	0.12
2005	Portugal	Male	0.37	0.55	0.22
2005	Spain	Both	0.73	1.02	0.47
2005	Spain	Female	0.45	0.61	0.30
2005	Spain	Male	1.06	1.52	0.67
2006	Greece	Both	0.11	0.17	0.07
2006	Greece	Female	0.14	0.20	0.08
2006	Greece	Male	0.08	0.13	0.04
2006	Italy	Both	0.78	1.04	0.55
2006	Italy	Female	0.51	0.69	0.36
2006	Italy	Male	1.10	1.45	0.77
2006	Portugal	Both	0.28	0.40	0.17
2006	Portugal	Female	0.20	0.28	0.13
2006	Portugal	Male	0.38	0.57	0.23
2006	Spain	Both	0.74	1.02	0.48
2006	Spain	Female	0.44	0.61	0.30
2006	Spain	Male	1.07	1.54	0.68
2007	Greece	Both	0.11	0.17	0.07
2007	Greece	Female	0.14	0.20	0.09
2007	Greece	Male	0.08	0.13	0.04
2007	Italy	Both	0.79	1.05	0.56
2007	Italy	Female	0.51	0.69	0.36
2007	Italy	Male	1.11	1.47	0.78
2007	Portugal	Both	0.29	0.42	0.18
2007	Portugal	Female	0.20	0.28	0.13
2007	Portugal	Male	0.40	0.60	0.24
2007	Spain	Both	0.74	1.03	0.49
2007	Spain	Female	0.44	0.60	0.30
2007	Spain	Male	1.09	1.54	0.70
2008	Greece	Both	0.12	0.18	0.07
2008	Greece	Female	0.14	0.21	0.09
2008	Greece	Male	0.09	0.14	0.05
2008	Italy	Both	0.79	1.06	0.56

2008	Italy	Female	0.52	0.70	0.36
2008	Italy	Male	1.12	1.50	0.79
2008	Portugal	Both	0.31	0.44	0.19
2008	Portugal	Female	0.21	0.30	0.13
2008	Portugal	Male	0.43	0.63	0.26
2008	Spain	Both	0.75	1.03	0.49
2008	Spain	Female	0.44	0.60	0.29
2008	Spain	Male	1.10	1.56	0.71
2009	Greece	Both	0.12	0.18	0.07
2009	Greece	Female	0.14	0.21	0.09
2009	Greece	Male	0.09	0.15	0.05
2009	Italy	Both	0.80	1.08	0.57
2009	Italy	Female	0.52	0.69	0.37
2009	Italy	Male	1.13	1.51	0.80
2009	Portugal	Both	0.33	0.46	0.20
2009	Portugal	Female	0.22	0.31	0.14
2009	Portugal	Male	0.45	0.67	0.27
2009	Spain	Both	0.75	1.05	0.49
2009	Spain	Female	0.44	0.60	0.29
2009	Spain	Male	1.11	1.59	0.71
2010	Greece	Both	0.12	0.19	0.07
2010	Greece	Female	0.15	0.22	0.09
2010	Greece	Male	0.10	0.16	0.05
2010	Italy	Both	0.80	1.08	0.57
2010	Italy	Female	0.52	0.70	0.37
2010	Italy	Male	1.14	1.52	0.80
2010	Portugal	Both	0.34	0.48	0.21
2010	Portugal	Female	0.23	0.32	0.14
2010	Portugal	Male	0.47	0.70	0.28
2010	Spain	Both	0.76	1.04	0.49
2010	Spain	Female	0.44	0.60	0.29
2010	Spain	Male	1.12	1.57	0.73
2011	Greece	Both	0.13	0.19	0.07
2011	Greece	Female	0.15	0.22	0.09
2011	Greece	Male	0.10	0.17	0.05
2011	Italy	Both	0.81	1.09	0.57
2011	Italy	Female	0.52	0.70	0.37
2011	Italy	Male	1.14	1.52	0.81
2011	Portugal	Both	0.35	0.49	0.21
2011	Portugal	Female	0.23	0.32	0.15
2011	Portugal	Male	0.49	0.72	0.30
2011	Spain	Both	0.76	1.06	0.50
2011	Spain	Female	0.43	0.59	0.28
2011	Spain	Male	1.13	1.61	0.73
2012	Greece	Both	0.13	0.20	0.08
2012	Greece	Female	0.15	0.22	0.09
2012	Greece	Male	0.11	0.17	0.06
2012	Italy	Both	0.81	1.09	0.58
2012	Italy	Female	0.52	0.70	0.37
2012	Italy	Male	1.14	1.53	0.81
2012	Portugal	Both	0.36	0.51	0.22
2012	Portugal	Female	0.24	0.33	0.15
2012	Portugal	Male	0.51	0.75	0.31

2012	Spain	Both	0.76	1.06	0.50
2012	Spain	Female	0.43	0.59	0.29
2012	Spain	Male	1.14	1.61	0.73
2013	Greece	Both	0.13	0.20	0.08
2013	Greece	Female	0.15	0.22	0.09
2013	Greece	Male	0.11	0.18	0.06
2013	Italy	Both	0.81	1.09	0.58
2013	Italy	Female	0.52	0.70	0.37
2013	Italy	Male	1.15	1.53	0.81
2013	Portugal	Both	0.37	0.53	0.23
2013	Portugal	Female	0.24	0.33	0.16
2013	Portugal	Male	0.53	0.78	0.32
2013	Spain	Both	0.76	1.05	0.50
2013	Spain	Female	0.43	0.59	0.29
2013	Spain	Male	1.14	1.62	0.74
2014	Greece	Both	0.14	0.20	0.08
2014	Greece	Female	0.15	0.22	0.09
2014	Greece	Male	0.12	0.19	0.06
2014	Italy	Both	0.81	1.09	0.58
2014	Italy	Female	0.52	0.70	0.36
2014	Italy	Male	1.15	1.53	0.81
2014	Portugal	Both	0.38	0.54	0.24
2014	Portugal	Female	0.24	0.34	0.16
2014	Portugal	Male	0.55	0.80	0.33
2014	Spain	Both	0.77	1.06	0.50
2014	Spain	Female	0.43	0.58	0.29
2014	Spain	Male	1.15	1.63	0.73
2015	Greece	Both	0.14	0.21	0.08
2015	Greece	Female	0.15	0.22	0.09
2015	Greece	Male	0.12	0.19	0.06
2015	Italy	Both	0.81	1.09	0.57
2015	Italy	Female	0.52	0.69	0.36
2015	Italy	Male	1.14	1.53	0.80
2015	Portugal	Both	0.39	0.56	0.24
2015	Portugal	Female	0.25	0.35	0.16
2015	Portugal	Male	0.56	0.83	0.33
2015	Spain	Both	0.76	1.07	0.49
2015	Spain	Female	0.43	0.59	0.29
2015	Spain	Male	1.15	1.63	0.72
2016	Greece	Both	0.14	0.21	0.08
2016	Greece	Female	0.15	0.22	0.09
2016	Greece	Male	0.12	0.19	0.06
2016	Italy	Both	0.79	1.06	0.56
2016	Italy	Female	0.50	0.68	0.35
2016	Italy	Male	1.12	1.52	0.77
2016	Portugal	Both	0.39	0.57	0.24
2016	Portugal	Female	0.25	0.35	0.16
2016	Portugal	Male	0.57	0.86	0.34
2016	Spain	Both	0.75	1.06	0.49
2016	Spain	Female	0.42	0.58	0.28
2016	Spain	Male	1.13	1.62	0.72
2017	Greece	Both	0.14	0.21	0.08
2017	Greece	Female	0.15	0.22	0.09

2017	Greece	Male	0.11	0.19	0.06
2017	Italy	Both	0.78	1.05	0.53
2017	Italy	Female	0.49	0.67	0.34
2017	Italy	Male	1.10	1.52	0.73
2017	Portugal	Both	0.39	0.58	0.24
2017	Portugal	Female	0.25	0.35	0.16
2017	Portugal	Male	0.57	0.88	0.34
2017	Spain	Both	0.74	1.05	0.46
2017	Spain	Female	0.42	0.59	0.27
2017	Spain	Male	1.10	1.61	0.67
2018	Greece	Both	0.13	0.21	0.07
2018	Greece	Female	0.15	0.22	0.09
2018	Greece	Male	0.11	0.19	0.06
2018	Italy	Both	0.78	1.07	0.53
2018	Italy	Female	0.49	0.66	0.33
2018	Italy	Male	1.10	1.56	0.73
2018	Portugal	Both	0.39	0.58	0.24
2018	Portugal	Female	0.24	0.35	0.16
2018	Portugal	Male	0.56	0.87	0.33
2018	Spain	Both	0.73	1.06	0.46
2018	Spain	Female	0.41	0.59	0.26
2018	Spain	Male	1.08	1.63	0.66
2019	Greece	Both	0.13	0.21	0.07
2019	Greece	Female	0.15	0.24	0.08
2019	Greece	Male	0.11	0.20	0.05
2019	Italy	Both	0.79	1.14	0.52
2019	Italy	Female	0.49	0.70	0.32
2019	Italy	Male	1.12	1.69	0.70
2019	Portugal	Both	0.38	0.59	0.22
2019	Portugal	Female	0.24	0.36	0.15
2019	Portugal	Male	0.54	0.87	0.30
2019	Spain	Both	0.72	1.10	0.43
2019	Spain	Female	0.42	0.60	0.25
2019	Spain	Male	1.06	1.64	0.61

Table S36- Age-standardized rates and 95% uncertainty levels (UL) of **disability-adjusted life years** (DALYs) **due to liver cancer due to hepatitis C** per 100,000 population in Greece, Italy, Portugal and Spain from 2000 to 2019 by sex classes (Global Burden of Disease Study 2019).

Year	Country	Sex	DALYs (95% UL)	95% UL (upper)	95% UL (lower)
2000	Greece	Both	8.1	10.8	5.7
2000	Greece	Female	9.7	12.5	7.0
2000	Greece	Male	6.2	8.93	3.9
2000	Italy	Both	71.3	78.1	64.6
2000	Italy	Female	45.6	48.8	42.1
2000	Italy	Male	101.8	113.0	90.8
2000	Portugal	Both	25.8	33.4	19.0
2000	Portugal	Female	19.4	23.5	15.2
2000	Portugal	Male	33.4	46.2	23.0
2000	Spain	Both	55.7	67.7	43.9
2000	Spain	Female	36.2	42.0	30.3
2000	Spain	Male	78.5	99.1	59.3
2001	Greece	Both	8.2	10.9	5.8
2001	Greece	Female	9.8	12.6	7.1
2001	Greece	Male	6.2	9.05	4.0
2001	Italy	Both	68.1	74.5	61.7
2001	Italy	Female	43.8	46.8	40.4
2001	Italy	Male	97.0	107.	86.6
2001	Portugal	Both	26.4	34.4	19.5
2001	Portugal	Female	19.8	24.1	15.6
2001	Portugal	Male	34.1	47.2	23.6
2001	Spain	Both	56.2	68.2	44.4
2001	Spain	Female	36.2	41.9	30.0
2001	Spain	Male	79.5	100.	59.8
2002	Greece	Both	8.2	10.9	5.7
2002	Greece	Female	9.7	12.5	7.1
2002	Greece	Male	6.2	9.12	4.0
2002	Italy	Both	65.1	71.3	58.9
2002	Italy	Female	42.0	44.9	38.8
2002	Italy	Male	92.5	102.	82.7
2002	Portugal	Both	25.4	33.1	18.7
2002	Portugal	Female	18.7	22.8	14.7
2002	Portugal	Male	33.3	46.3	22.8
2002	Spain	Both	56.8	68.8	44.5
2002	Spain	Female	36.2	42.0	29.8
2002	Spain	Male	80.6	101.	60.3
2003	Greece	Both	8.2	11.0	5.7
2003	Greece	Female	9.7	12.5	7.1
2003	Greece	Male	6.3	9.18	4.0
2003	Italy	Both	60.8	66.8	55.0
2003	Italy	Female	39.1	42.0	35.8
2003	Italy	Male	86.5	96.2	77.0
2003	Portugal	Both	25.6	33.5	18.8
2003	Portugal	Female	18.4	22.5	14.4
2003	Portugal	Male	34.1	47.1	23.4
2003	Spain	Both	56.4	68.9	44.2

2003	Spain	Female	35.5	41.5	29.4
2003	Spain	Male	80.6	101.	60.3
2004	Greece	Both	8.1	10.9	5.6
2004	Greece	Female	9.6	12.4	6.9
2004	Greece	Male	6.2	9.15	3.9
2004	Italy	Both	58.2	63.7	52.7
2004	Italy	Female	37.6	40.3	34.4
2004	Italy	Male	82.6	91.5	73.9
2004	Portugal	Both	26.0	34.0	19.1
2004	Portugal	Female	18.3	22.2	14.4
2004	Portugal	Male	34.9	48.3	24.0
2004	Spain	Both	56.1	68.4	43.8
2004	Spain	Female	35.2	41.2	28.9
2004	Spain	Male	80.1	100.	59.8
2005	Greece	Both	8.1	10.9	5.6
2005	Greece	Female	9.6	12.5	6.9
2005	Greece	Male	6.3	9.32	3.9
2005	Italy	Both	55.7	60.5	50.6
2005	Italy	Female	35.8	38.3	32.6
2005	Italy	Male	79.2	87.2	70.9
2005	Portugal	Both	26.7	34.9	19.7
2005	Portugal	Female	18.5	22.5	14.6
2005	Portugal	Male	36.2	50.1	24.5
2005	Spain	Both	55.3	67.3	43.3
2005	Spain	Female	34.5	40.3	28.3
2005	Spain	Male	79.0	98.9	59.4
2006	Greece	Both	8.1	11.0	5.7
2006	Greece	Female	9.7	12.5	7.0
2006	Greece	Male	6.3	9.34	3.9
2006	Italy	Both	54.4	59.3	49.3
2006	Italy	Female	34.8	37.4	31.6
2006	Italy	Male	77.4	85.5	69.6
2006	Portugal	Both	27.5	36.0	20.3
2006	Portugal	Female	18.6	22.5	14.7
2006	Portugal	Male	38.0	52.2	25.9
2006	Spain	Both	54.8	66.7	43.0
2006	Spain	Female	34.0	39.4	27.8
2006	Spain	Male	78.5	98.3	59.4
2007	Greece	Both	8.4	11.3	5.9
2007	Greece	Female	9.9	12.7	7.2
2007	Greece	Male	6.6	9.88	4.1
2007	Italy	Both	53.6	58.4	48.5
2007	Italy	Female	34.2	36.7	30.9
2007	Italy	Male	76.2	84.1	68.4
2007	Portugal	Both	28.4	36.8	20.8
2007	Portugal	Female	18.9	22.8	15.3
2007	Portugal	Male	39.3	53.9	27.1
2007	Spain	Both	55.2	66.9	43.2
2007	Spain	Female	33.6	39.0	27.5
2007	Spain	Male	79.6	99.3	60.0
2008	Greece	Both	8.5	11.4	6.0
2008	Greece	Female	10.0	12.9	7.3
2008	Greece	Male	6.7	10.0	4.2

2008	Italy	Both	53.9	58.6	49.0
2008	Italy	Female	34.7	37.1	31.4
2008	Italy	Male	76.2	84.2	68.5
2008	Portugal	Both	29.4	37.8	21.3
2008	Portugal	Female	19.4	23.2	15.5
2008	Portugal	Male	41.0	55.4	28.3
2008	Spain	Both	55.6	67.2	43.6
2008	Spain	Female	33.7	39.1	27.5
2008	Spain	Male	80.4	100.	60.7
2009	Greece	Both	8.9	12.0	6.2
2009	Greece	Female	10.3	13.3	7.5
2009	Greece	Male	7.2	10.8	4.5
2009	Italy	Both	54.7	59.5	49.6
2009	Italy	Female	34.9	37.5	31.7
2009	Italy	Male	77.5	85.6	69.5
2009	Portugal	Both	30.8	39.5	22.4
2009	Portugal	Female	20.1	24.2	16.1
2009	Portugal	Male	43.2	58.5	29.9
2009	Spain	Both	56.5	68.5	43.7
2009	Spain	Female	33.9	39.4	28.0
2009	Spain	Male	81.9	102.	61.5
2010	Greece	Both	9.2	12.5	6.4
2010	Greece	Female	10.5	13.7	7.6
2010	Greece	Male	7.6	11.4	4.8
2010	Italy	Both	56.0	60.9	50.8
2010	Italy	Female	35.7	38.3	32.4
2010	Italy	Male	79.3	87.5	71.5
2010	Portugal	Both	32.2	41.4	23.5
2010	Portugal	Female	21.0	25.3	16.6
2010	Portugal	Male	45.3	61.1	31.1
2010	Spain	Both	57.0	69.1	43.8
2010	Spain	Female	33.8	39.1	27.7
2010	Spain	Male	83.1	103.	62.2
2011	Greece	Both	9.5	13.0	6.6
2011	Greece	Female	10.7	14.0	7.8
2011	Greece	Male	8.1	12.1	5.1
2011	Italy	Both	57.1	62.2	52.0
2011	Italy	Female	36.2	38.6	32.8
2011	Italy	Male	81.2	89.5	72.8
2011	Portugal	Both	33.5	43.3	24.4
2011	Portugal	Female	21.4	25.7	17.0
2011	Portugal	Male	47.5	64.3	32.7
2011	Spain	Both	57.5	69.9	44.1
2011	Spain	Female	33.6	38.8	27.4
2011	Spain	Male	84.2	105.	63.2
2012	Greece	Both	9.9	13.6	6.8
2012	Greece	Female	11.0	14.4	7.9
2012	Greece	Male	8.6	12.9	5.4
2012	Italy	Both	57.9	63.2	52.5
2012	Italy	Female	36.7	39.3	33.4
2012	Italy	Male	82.1	90.8	73.2
2012	Portugal	Both	34.7	44.9	25.3
2012	Portugal	Female	21.9	26.6	17.5

2012	Portugal	Male	49.5	67.0	34.0
2012	Spain	Both	58.0	70.5	44.7
2012	Spain	Female	33.8	39.0	27.9
2012	Spain	Male	85.1	106.	63.3
2013	Greece	Both	10.3	14.1	7.0
2013	Greece	Female	11.2	14.6	8.0
2013	Greece	Male	9.2	13.7	5.7
2013	Italy	Both	57.1	62.2	51.7
2013	Italy	Female	36.2	38.8	32.8
2013	Italy	Male	80.9	89.4	72.3
2013	Portugal	Both	35.6	46.3	25.9
2013	Portugal	Female	22.4	27.1	17.8
2013	Portugal	Male	51.1	69.2	35.3
2013	Spain	Both	57.9	70.4	44.6
2013	Spain	Female	33.6	38.9	27.8
2013	Spain	Male	85.1	106.	63.1
2014	Greece	Both	10.4	14.2	7.0
2014	Greece	Female	11.3	14.7	8.1
2014	Greece	Male	9.2	13.8	5.7
2014	Italy	Both	56.0	61.2	50.6
2014	Italy	Female	35.7	38.4	32.1
2014	Italy	Male	79.2	87.9	70.6
2014	Portugal	Both	36.1	46.8	26.2
2014	Portugal	Female	22.5	27.2	17.8
2014	Portugal	Male	52.0	70.6	35.4
2014	Spain	Both	57.9	70.5	44.6
2014	Spain	Female	33.4	38.7	27.7
2014	Spain	Male	85.2	106.	63.3
2015	Greece	Both	10.4	14.3	7.0
2015	Greece	Female	11.3	14.8	8.0
2015	Greece	Male	9.3	14.0	5.8
2015	Italy	Both	54.9	59.9	49.5
2015	Italy	Female	34.9	37.7	31.6
2015	Italy	Male	77.5	85.8	69.0
2015	Portugal	Both	36.4	47.7	26.5
2015	Portugal	Female	22.4	27.3	17.6
2015	Portugal	Male	52.8	72.1	35.4
2015	Spain	Both	58.0	70.7	44.7
2015	Spain	Female	33.3	38.8	27.7
2015	Spain	Male	85.6	107.	63.3
2016	Greece	Both	10.5	14.5	7.1
2016	Greece	Female	11.4	15.0	8.1
2016	Greece	Male	9.3	14.0	5.8
2016	Italy	Both	51.5	56.5	46.2
2016	Italy	Female	33.1	35.7	29.8
2016	Italy	Male	72.4	80.7	63.8
2016	Portugal	Both	36.9	48.4	26.7
2016	Portugal	Female	22.5	27.6	17.7
2016	Portugal	Male	53.7	73.6	36.6
2016	Spain	Both	57.3	70.1	44.3
2016	Spain	Female	33.1	38.6	27.4
2016	Spain	Male	84.3	106.	62.3
2017	Greece	Both	10.3	14.3	7.0

2017	Greece	Female	11.2	14.7	8.0
2017	Greece	Male	9.1	13.7	5.5
2017	Italy	Both	53.1	58.8	47.2
2017	Italy	Female	33.5	36.3	30.1
2017	Italy	Male	75.2	85.0	65.5
2017	Portugal	Both	36.3	47.7	25.8
2017	Portugal	Female	22.1	27.2	17.3
2017	Portugal	Male	52.9	72.2	35.8
2017	Spain	Both	56.5	69.6	43.5
2017	Spain	Female	32.9	38.6	27.1
2017	Spain	Male	82.8	105.	60.6
2018	Greece	Both	10.2	14.1	6.95
2018	Greece	Female	11.3	14.8	8.0
2018	Greece	Male	8.8	13.3	5.4
2018	Italy	Both	53.5	59.5	47.3
2018	Italy	Female	33.7	36.7	30.2
2018	Italy	Male	75.8	86.3	65.5
2018	Portugal	Both	35.5	46.7	25.3
2018	Portugal	Female	22.0	27.1	17.1
2018	Portugal	Male	51.2	70.5	34.4
2018	Spain	Both	55.5	68.5	42.4
2018	Spain	Female	32.8	38.6	27.1
2018	Spain	Male	80.7	103.	58.5
2019	Greece	Both	10.1	14.1	6.92
2019	Greece	Female	11.2	14.8	8.0
2019	Greece	Male	8.6	12.9	5.4
2019	Italy	Both	53.9	60.2	47.2
2019	Italy	Female	33.9	37.0	30.4
2019	Italy	Male	76.3	87.5	65.0
2019	Portugal	Both	34.8	46.3	24.7
2019	Portugal	Female	21.8	27.0	16.9
2019	Portugal	Male	50.0	69.7	33.7
2019	Spain	Both	54.9	68.2	41.9
2019	Spain	Female	32.8	38.9	26.7
2019	Spain	Male	79.5	103.	58.1

Table S37- Rates and 95% uncertainty levels (UL) of **acute HBV prevalence** per 100,000 population in Greece, Italy, Portugal and Spain from 2000 to 2019 by age group (Global Burden of Disease Study 2019).

Year	Country	Age	Prevalence (95% UL)	95% UL (upper)	95% UL (lower)
2000	Greece	Under 5	8.61	11.9	6.18
2000	Greece	5-14 years	57.4	76.1	44.3
2000	Greece	15-49 years	98.5	126.	73.0
2000	Greece	50-69 years	54.1	83.6	31.7
2000	Greece	70+ years	34.3	62.6	14.2
2000	Greece	All Ages	72.9	89.4	57.5
2005	Greece	Under 5	5.74	8.41	3.80
2005	Greece	5-14 years	36.7	49.9	25.7
2005	Greece	15-49 years	95.8	122.	72.8
2005	Greece	50-69 years	53.8	79.8	31.4
2005	Greece	70+ years	34.2	56.8	16.9
2005	Greece	All Ages	68.4	84.9	54.1
2010	Greece	Under 5	4.50	6.34	3.20
2010	Greece	5-14 years	7.89	10.3	5.95
2010	Greece	15-49 years	95.3	123.	72.4
2010	Greece	50-69 years	52.5	81.6	27.5
2010	Greece	70+ years	32.2	57.7	13.0
2010	Greece	All Ages	63.5	81.4	49.2
2015	Greece	Under 5	4.15	5.76	2.99
2015	Greece	5-14 years	5.87	7.68	4.37
2015	Greece	15-49 years	87.7	117.	63.1
2015	Greece	50-69 years	50.8	78.6	27.6
2015	Greece	70+ years	30.7	54.8	13.5
2015	Greece	All Ages	57.8	74.4	42.4
2019	Greece	Under 5	4.01	5.41	2.95
2019	Greece	5-14 years	5.14	6.88	3.84
2019	Greece	15-49 years	83.4	106.	63.5
2019	Greece	50-69 years	52.0	76.3	29.4
2019	Greece	70+ years	31.2	54.4	12.7
2019	Greece	All Ages	55.4	69.1	42.2
2000	Italy	Under 5	2.93	4.02	2.13
2000	Italy	5-14 years	9.04	12.7	6.27
2000	Italy	15-49 years	39.7	53.0	28.4
2000	Italy	50-69 years	23.5	34.8	14.5
2000	Italy	70+ years	14.7	24.4	7.71
2000	Italy	All Ages	27.9	35.6	21.2
2005	Italy	Under 5	2.31	3.13	1.68
2005	Italy	5-14 years	3.80	5.36	2.57
2005	Italy	15-49 years	33.9	45.1	23.7
2005	Italy	50-69 years	21.2	31.3	12.4
2005	Italy	70+ years	13.0	21.5	6.50
2005	Italy	All Ages	23.6	30.3	17.4
2010	Italy	Under 5	2.26	3.06	1.60
2010	Italy	5-14 years	2.14	3.07	1.48
2010	Italy	15-49 years	26.2	36.5	17.7
2010	Italy	50-69 years	18.7	29.1	10.5
2010	Italy	70+ years	11.5	20.4	5.37
2010	Italy	All Ages	18.8	24.9	13.3

2015	Italy	Under 5	2.24	3.03	1.60
2015	Italy	5-14 years	1.77	2.52	1.21
2015	Italy	15-49 years	20.0	28.9	13.1
2015	Italy	50-69 years	17.9	27.3	10.1
2015	Italy	70+ years	10.9	18.4	5.43
2015	Italy	All Ages	15.5	21.3	10.7
2019	Italy	Under 5	2.24	2.97	1.67
2019	Italy	5-14 years	1.83	2.61	1.21
2019	Italy	15-49 years	14.9	21.4	9.47
2019	Italy	50-69 years	17.4	27.2	9.53
2019	Italy	70+ years	10.3	17.9	4.96
2019	Italy	All Ages	13.1	18.4	8.37
2000	Portugal	Under 5	15.2	22.4	9.23
2000	Portugal	5-14 years	34.7	51.2	22.8
2000	Portugal	15-49 years	40.3	58.0	26.6
2000	Portugal	50-69 years	16.3	29.9	6.97
2000	Portugal	70+ years	9.10	18.1	3.23
2000	Portugal	All Ages	29.8	39.9	21.4
2005	Portugal	Under 5	4.58	6.68	3.01
2005	Portugal	5-14 years	21.9	32.6	14.3
2005	Portugal	15-49 years	41.1	56.7	27.0
2005	Portugal	50-69 years	17.1	30.0	6.94
2005	Portugal	70+ years	9.58	18.4	2.77
2005	Portugal	All Ages	28.0	37.0	19.1
2010	Portugal	Under 5	3.86	5.74	2.50
2010	Portugal	5-14 years	8.33	12.5	5.32
2010	Portugal	15-49 years	38.7	56.3	24.5
2010	Portugal	50-69 years	16.6	28.2	7.17
2010	Portugal	70+ years	9.26	17.5	2.96
2010	Portugal	All Ages	24.7	33.9	16.9
2015	Portugal	Under 5	3.25	4.75	2.12
2015	Portugal	5-14 years	3.69	5.42	2.45
2015	Portugal	15-49 years	35.0	50.3	21.8
2015	Portugal	50-69 years	15.9	27.1	6.36
2015	Portugal	70+ years	8.70	16.6	2.50
2015	Portugal	All Ages	21.8	30.4	14.1
2019	Portugal	Under 5	3.06	4.45	1.90
2019	Portugal	5-14 years	3.07	4.58	2.04
2019	Portugal	15-49 years	29.0	42.6	18.2
2019	Portugal	50-69 years	16.0	29.0	6.70
2019	Portugal	70+ years	8.73	17.6	2.81
2019	Portugal	All Ages	18.8	26.9	11.8
2000	Spain	Under 5	7.97	11.0	5.45
2000	Spain	5-14 years	20.6	29.0	14.3
2000	Spain	15-49 years	43.4	56.4	31.2
2000	Spain	50-69 years	22.1	34.4	11.8
2000	Spain	70+ years	14.4	25.5	6.40
2000	Spain	All Ages	31.5	39.7	24.5
2005	Spain	Under 5	2.86	3.87	2.01
2005	Spain	5-14 years	8.14	10.9	5.88
2005	Spain	15-49 years	43.5	57.3	31.6
2005	Spain	50-69 years	21.4	33.0	11.7
2005	Spain	70+ years	13.7	24.1	6.43

2005	Spain	All Ages	29.9	38.0	22.6
2010	Spain	Under 5	2.61	3.68	1.86
2010	Spain	5-14 years	5.37	7.50	3.82
2010	Spain	15-49 years	38.3	51.6	27.1
2010	Spain	50-69 years	21.4	33.0	10.6
2010	Spain	70+ years	13.7	24.6	5.78
2010	Spain	All Ages	26.5	34.5	19.6
2015	Spain	Under 5	2.57	3.58	1.84
2015	Spain	5-14 years	2.73	3.68	2.00
2015	Spain	15-49 years	35.2	48.5	24.7
2015	Spain	50-69 years	22.1	35.2	12.3
2015	Spain	70+ years	14.1	23.9	6.35
2015	Spain	All Ages	24.3	32.3	17.7
2019	Spain	Under 5	2.37	3.27	1.73
2019	Spain	5-14 years	2.62	3.61	1.90
2019	Spain	15-49 years	28.6	38.8	19.7
2019	Spain	50-69 years	21.6	33.3	11.7
2019	Spain	70+ years	13.9	24.4	6.22
2019	Spain	All Ages	20.8	27.5	15.0

Table S38- Rates and 95% uncertainty levels (UL) of **acute HBV incidence** per 100,000 population in Greece, Italy, Portugal and Spain from 2000 to 2019 by age group (Global Burden of Disease Study 2019).

Year	Country	Age	incidence (95% UL)	95% UL (upper)	95% UL (lower)
2000	Greece	Under 5	74.6	103.2	53.5
2000	Greece	5-14 years	497.6	660.1	384.0
2000	Greece	15-49 years	854.4	1096.	632.7
2000	Greece	50-69 years	469.7	725.1	275.4
2000	Greece	70+ years	297.8	543.3	123.5
2000	Greece	All Ages	631.9	775.1	499.1
2005	Greece	Under 5	49.8	72.9	32.9
2005	Greece	5-14 years	318.2	432.8	223.2
2005	Greece	15-49 years	830.2	1064.	631.4
2005	Greece	50-69 years	466.8	691.8	272.3
2005	Greece	70+ years	296.8	492.9	146.8
2005	Greece	All Ages	593.5	736.2	468.9
2010	Greece	Under 5	39.0	54.9	27.7
2010	Greece	5-14 years	68.4	89.4	51.5
2010	Greece	15-49 years	826.3	1069.	627.6
2010	Greece	50-69 years	455.3	707.4	238.3
2010	Greece	70+ years	279.8	500.5	112.9
2010	Greece	All Ages	550.8	706.0	426.6
2015	Greece	Under 5	35.9	50.0	25.9
2015	Greece	5-14 years	50.9	66.6	37.9
2015	Greece	15-49 years	760.4	1021.	546.9
2015	Greece	50-69 years	441.1	681.7	239.6
2015	Greece	70+ years	266.9	475.2	117.1
2015	Greece	All Ages	501.6	645.3	368.0
2019	Greece	Under 5	34.7	46.9	25.6
2019	Greece	5-14 years	44.5	59.6	33.3
2019	Greece	15-49 years	723.2	924.1	550.4
2019	Greece	50-69 years	450.6	661.2	255.0
2019	Greece	70+ years	270.7	472.3	110.5
2019	Greece	All Ages	480.8	599.0	365.9
2000	Italy	Under 5	25.4	34.8	18.5
2000	Italy	5-14 years	78.3	110.	54.4
2000	Italy	15-49 years	344.2	459.3	246.8
2000	Italy	50-69 years	203.8	302.0	126.0
2000	Italy	70+ years	127.8	211.7	66.87
2000	Italy	All Ages	242.1	308.9	183.9
2005	Italy	Under 5	20.0	27.1	14.5
2005	Italy	5-14 years	32.9	46.4	22.3
2005	Italy	15-49 years	294.6	391.3	206.1
2005	Italy	50-69 years	184.3	271.2	107.5
2005	Italy	70+ years	113.3	186.3	56.37
2005	Italy	All Ages	205.3	262.7	151.2
2010	Italy	Under 5	19.6	26.5	13.9
2010	Italy	5-14 years	18.6	26.6	12.8
2010	Italy	15-49 years	227.9	316.3	153.9
2010	Italy	50-69 years	162.7	252.4	91.25
2010	Italy	70+ years	100.2	177.5	46.55
2010	Italy	All Ages	163.6	216.4	115.8

2015	Italy	Under 5	19.4	26.2	13.8
2015	Italy	5-14 years	15.4	21.9	10.5
2015	Italy	15-49 years	174.0	250.9	114.1
2015	Italy	50-69 years	155.2	237.1	87.98
2015	Italy	70+ years	95.3	160.	47.0
2015	Italy	All Ages	134.9	184.8	93.19
2019	Italy	Under 5	19.4	25.7	14.5
2019	Italy	5-14 years	15.9	22.6	10.5
2019	Italy	15-49 years	129.1	186.2	82.13
2019	Italy	50-69 years	150.8	236.5	82.65
2019	Italy	70+ years	89.9	155.	43.0
2019	Italy	All Ages	113.6	160.2	72.62
2000	Portugal	Under 5	132.0	194.8	80.07
2000	Portugal	5-14 years	301.0	444.4	198.1
2000	Portugal	15-49 years	349.8	503.1	230.6
2000	Portugal	50-69 years	142.0	259.8	60.44
2000	Portugal	70+ years	78.9	157.	28.0
2000	Portugal	All Ages	258.4	346.4	185.8
2005	Portugal	Under 5	39.6	57.9	26.1
2005	Portugal	5-14 years	190.1	282.9	124.2
2005	Portugal	15-49 years	356.7	492.1	234.3
2005	Portugal	50-69 years	148.8	260.0	60.22
2005	Portugal	70+ years	83.0	160.1	24.0
2005	Portugal	All Ages	242.7	321.3	166.3
2010	Portugal	Under 5	33.4	49.8	21.6
2010	Portugal	5-14 years	72.2	108.	46.1
2010	Portugal	15-49 years	336.1	488.6	212.8
2010	Portugal	50-69 years	144.6	245.1	62.14
2010	Portugal	70+ years	80.2	152.	25.6
2010	Portugal	All Ages	214.1	294.5	146.5
2015	Portugal	Under 5	28.1	41.1	18.3
2015	Portugal	5-14 years	32.0	47.0	21.2
2015	Portugal	15-49 years	303.6	436.7	189.5
2015	Portugal	50-69 years	138.0	235.3	55.13
2015	Portugal	70+ years	75.4	144.	21.7
2015	Portugal	All Ages	189.1	263.8	122.3
2019	Portugal	Under 5	26.5	38.6	16.5
2019	Portugal	5-14 years	26.6	39.7	17.6
2019	Portugal	15-49 years	251.6	369.9	158.1
2019	Portugal	50-69 years	138.9	251.5	58.11
2019	Portugal	70+ years	75.6	153.	24.4
2019	Portugal	All Ages	163.7	233.9	102.6
2000	Spain	Under 5	69.1	95.6	47.3
2000	Spain	5-14 years	178.5	252.1	124.2
2000	Spain	15-49 years	376.2	489.0	271.1
2000	Spain	50-69 years	192.1	298.6	103.1
2000	Spain	70+ years	125.0	221.1	55.54
2000	Spain	All Ages	273.6	344.5	212.3
2005	Spain	Under 5	24.8	33.5	17.4
2005	Spain	5-14 years	70.5	94.8	51.0
2005	Spain	15-49 years	377.6	496.8	274.4
2005	Spain	50-69 years	185.5	286.4	101.9
2005	Spain	70+ years	118.8	208.9	55.76

2005	Spain	All Ages	259.4	330.0	196.4
2010	Spain	Under 5	22.6	31.8	16.1
2010	Spain	5-14 years	46.5	65.0	33.1
2010	Spain	15-49 years	332.1	447.7	235.4
2010	Spain	50-69 years	186.1	286.2	92.62
2010	Spain	70+ years	119.1	214.0	50.15
2010	Spain	All Ages	230.3	299.8	170.0
2015	Spain	Under 5	22.3	31.0	16.0
2015	Spain	5-14 years	23.7	31.9	17.3
2015	Spain	15-49 years	305.1	420.4	214.4
2015	Spain	50-69 years	191.6	305.3	106.9
2015	Spain	70+ years	122.4	207.5	55.10
2015	Spain	All Ages	210.9	280.4	153.6
2019	Spain	Under 5	20.5	28.3	15.0
2019	Spain	5-14 years	22.7	31.3	16.5
2019	Spain	15-49 years	248.2	337.0	170.7
2019	Spain	50-69 years	187.5	289.0	101.5
2019	Spain	70+ years	121.1	211.4	53.94
2019	Spain	All Ages	180.8	238.4	130.4

Table S39- Rates and 95% uncertainty levels (UL) of **acute HBV deaths** per 100,000 population in Greece, Italy, Portugal and Spain from 2000 to 2019 by age group (Global Burden of Disease Study 2019).

Year	Country	Age	Deaths (95% UL)	95% UL (upper)	95% UL (lower)
2000	Greece	Under 5	0.014	0.036	0.005
2000	Greece	5-14 years	0.001	0.003	0.000
2000	Greece	15-49 years	0.037	0.044	0.031
2000	Greece	50-69 years	0.232	0.274	0.185
2000	Greece	70+ years	0.790	0.982	0.544
2000	Greece	All Ages	0.157	0.184	0.122
2005	Greece	Under 5	0.019	0.050	0.007
2005	Greece	5-14 years	0.002	0.004	0.001
2005	Greece	15-49 years	0.063	0.075	0.049
2005	Greece	50-69 years	0.373	0.438	0.298
2005	Greece	70+ years	1.452	1.800	1.004
2005	Greece	All Ages	0.298	0.349	0.226
2010	Greece	Under 5	0.034	0.090	0.010
2010	Greece	5-14 years	0.005	0.008	0.002
2010	Greece	15-49 years	0.124	0.147	0.100
2010	Greece	50-69 years	0.727	0.833	0.602
2010	Greece	70+ years	3.521	4.379	2.414
2010	Greece	All Ages	0.730	0.863	0.553
2015	Greece	Under 5	0.015	0.043	0.004
2015	Greece	5-14 years	0.001	0.003	0.001
2015	Greece	15-49 years	0.054	0.068	0.044
2015	Greece	50-69 years	0.337	0.387	0.285
2015	Greece	70+ years	1.232	1.509	0.902
2015	Greece	All Ages	0.301	0.348	0.242
2019	Greece	Under 5	0.013	0.035	0.004
2019	Greece	5-14 years	0.001	0.003	0.001
2019	Greece	15-49 years	0.059	0.075	0.047
2019	Greece	50-69 years	0.336	0.394	0.270
2019	Greece	70+ years	1.222	1.533	0.883
2019	Greece	All Ages	0.317	0.375	0.248
2000	Italy	Under 5	0.032	0.046	0.015
2000	Italy	5-14 years	0.001	0.002	0.000
2000	Italy	15-49 years	0.024	0.029	0.017
2000	Italy	50-69 years	0.094	0.110	0.069
2000	Italy	70+ years	0.164	0.202	0.093
2000	Italy	All Ages	0.057	0.068	0.039
2005	Italy	Under 5	0.025	0.040	0.009
2005	Italy	5-14 years	0.001	0.001	0.000
2005	Italy	15-49 years	0.022	0.034	0.015
2005	Italy	50-69 years	0.094	0.140	0.064
2005	Italy	70+ years	0.167	0.255	0.099
2005	Italy	All Ages	0.058	0.087	0.037
2010	Italy	Under 5	0.019	0.035	0.007
2010	Italy	5-14 years	0.000	0.001	0.000
2010	Italy	15-49 years	0.019	0.032	0.013
2010	Italy	50-69 years	0.084	0.129	0.054
2010	Italy	70+ years	0.161	0.263	0.087
2010	Italy	All Ages	0.054	0.087	0.033

2015	Italy	Under 5	0.014	0.024	0.006
2015	Italy	5-14 years	0.000	0.001	0.000
2015	Italy	15-49 years	0.016	0.025	0.012
2015	Italy	50-69 years	0.073	0.107	0.051
2015	Italy	70+ years	0.148	0.225	0.088
2015	Italy	All Ages	0.050	0.075	0.034
2019	Italy	Under 5	0.014	0.021	0.006
2019	Italy	5-14 years	0.000	0.001	0.000
2019	Italy	15-49 years	0.018	0.025	0.013
2019	Italy	50-69 years	0.076	0.099	0.054
2019	Italy	70+ years	0.159	0.210	0.097
2019	Italy	All Ages	0.056	0.073	0.038
2000	Portugal	Under 5	0.040	0.069	0.021
2000	Portugal	5-14 years	0.007	0.011	0.002
2000	Portugal	15-49 years	0.109	0.137	0.064
2000	Portugal	50-69 years	0.191	0.232	0.134
2000	Portugal	70+ years	0.218	0.282	0.130
2000	Portugal	All Ages	0.124	0.148	0.086
2005	Portugal	Under 5	0.005	0.012	0.002
2005	Portugal	5-14 years	0.001	0.002	0.000
2005	Portugal	15-49 years	0.022	0.031	0.018
2005	Portugal	50-69 years	0.055	0.069	0.045
2005	Portugal	70+ years	0.067	0.093	0.050
2005	Portugal	All Ages	0.032	0.042	0.027
2010	Portugal	Under 5	0.003	0.008	0.001
2010	Portugal	5-14 years	0.000	0.001	0.000
2010	Portugal	15-49 years	0.016	0.022	0.012
2010	Portugal	50-69 years	0.043	0.052	0.036
2010	Portugal	70+ years	0.049	0.072	0.037
2010	Portugal	All Ages	0.025	0.032	0.021
2015	Portugal	Under 5	0.002	0.006	0.000
2015	Portugal	5-14 years	0.000	0.000	0.000
2015	Portugal	15-49 years	0.012	0.015	0.009
2015	Portugal	50-69 years	0.038	0.045	0.031
2015	Portugal	70+ years	0.047	0.065	0.035
2015	Portugal	All Ages	0.022	0.027	0.019
2019	Portugal	Under 5	0.002	0.005	0.000
2019	Portugal	5-14 years	0.000	0.000	0.000
2019	Portugal	15-49 years	0.013	0.017	0.010
2019	Portugal	50-69 years	0.037	0.044	0.029
2019	Portugal	70+ years	0.047	0.064	0.033
2019	Portugal	All Ages	0.023	0.028	0.019
2000	Spain	Under 5	0.020	0.036	0.006
2000	Spain	5-14 years	0.000	0.001	0.000
2000	Spain	15-49 years	0.007	0.012	0.005
2000	Spain	50-69 years	0.023	0.039	0.016
2000	Spain	70+ years	0.038	0.058	0.026
2000	Spain	All Ages	0.014	0.022	0.011
2005	Spain	Under 5	0.011	0.024	0.004
2005	Spain	5-14 years	0.000	0.000	0.000
2005	Spain	15-49 years	0.006	0.012	0.004
2005	Spain	50-69 years	0.020	0.035	0.013
2005	Spain	70+ years	0.030	0.054	0.020

2005	Spain	All Ages	0.012	0.020	0.008
2010	Spain	Under 5	0.012	0.020	0.006
2010	Spain	5-14 years	0.000	0.000	0.000
2010	Spain	15-49 years	0.007	0.011	0.006
2010	Spain	50-69 years	0.022	0.031	0.017
2010	Spain	70+ years	0.037	0.050	0.024
2010	Spain	All Ages	0.014	0.019	0.010
2015	Spain	Under 5	0.009	0.016	0.004
2015	Spain	5-14 years	0.000	0.000	0.000
2015	Spain	15-49 years	0.008	0.010	0.006
2015	Spain	50-69 years	0.024	0.030	0.016
2015	Spain	70+ years	0.040	0.052	0.023
2015	Spain	All Ages	0.015	0.018	0.010
2019	Spain	Under 5	0.008	0.016	0.003
2019	Spain	5-14 years	0.000	0.000	0.000
2019	Spain	15-49 years	0.008	0.010	0.006
2019	Spain	50-69 years	0.023	0.029	0.016
2019	Spain	70+ years	0.039	0.053	0.023
2019	Spain	All Ages	0.015	0.019	0.010

Table S40- Rates and 95% uncertainty levels (UL) of **acute HBV years of life lost** (YLLs) per 100,000 population in Greece, Italy, Portugal and Spain from 2000 to 2019 by age group (Global Burden of Disease Study 2019).

Year	Country	Age	YLLs (95% UL)	95% UL (upper)	95% UL (lower)
2000	Greece	Under 5	1.29	3.24	0.51
2000	Greece	5-14 years	0.15	0.23	0.07
2000	Greece	15-49 years	1.90	2.23	1.58
2000	Greece	50-69 years	6.56	7.71	5.28
2000	Greece	70+ years	10.7	13.4	7.52
2000	Greece	All Ages	3.69	4.19	3.09
2005	Greece	Under 5	1.75	4.45	0.68
2005	Greece	5-14 years	0.23	0.35	0.11
2005	Greece	15-49 years	3.17	3.78	2.48
2005	Greece	50-69 years	10.7	12.4	8.64
2005	Greece	70+ years	19.7	24.5	13.9
2005	Greece	All Ages	6.58	7.46	5.30
2010	Greece	Under 5	2.99	8.01	0.91
2010	Greece	5-14 years	0.41	0.64	0.20
2010	Greece	15-49 years	6.16	7.33	4.98
2010	Greece	50-69 years	21.0	24.0	17.6
2010	Greece	70+ years	45.7	56.7	31.8
2010	Greece	All Ages	14.5	16.4	11.7
2015	Greece	Under 5	1.34	3.86	0.37
2015	Greece	5-14 years	0.15	0.24	0.08
2015	Greece	15-49 years	2.68	3.30	2.20
2015	Greece	50-69 years	9.76	11.1	8.33
2015	Greece	70+ years	15.4	18.9	11.3
2015	Greece	All Ages	6.12	6.84	5.22
2019	Greece	Under 5	1.20	3.16	0.35
2019	Greece	5-14 years	0.15	0.26	0.08
2019	Greece	15-49 years	2.91	3.65	2.33
2019	Greece	50-69 years	9.77	11.4	7.94
2019	Greece	70+ years	15.0	18.7	10.9
2019	Greece	All Ages	6.37	7.33	5.32
2000	Italy	Under 5	2.88	4.10	1.35
2000	Italy	5-14 years	0.13	0.18	0.04
2000	Italy	15-49 years	1.30	1.53	0.92
2000	Italy	50-69 years	2.75	3.21	2.04
2000	Italy	70+ years	2.39	2.93	1.41
2000	Italy	All Ages	1.75	2.06	1.22
2005	Italy	Under 5	2.28	3.55	0.86
2005	Italy	5-14 years	0.08	0.11	0.04
2005	Italy	15-49 years	1.16	1.79	0.77
2005	Italy	50-69 years	2.75	4.06	1.87
2005	Italy	70+ years	2.40	3.69	1.47
2005	Italy	All Ages	1.66	2.48	1.10
2010	Italy	Under 5	1.74	3.15	0.64
2010	Italy	5-14 years	0.06	0.11	0.04
2010	Italy	15-49 years	0.99	1.64	0.66
2010	Italy	50-69 years	2.44	3.77	1.59
2010	Italy	70+ years	2.28	3.67	1.26
2010	Italy	All Ages	1.48	2.34	0.94

2015	Italy	Under 5	1.31	2.18	0.58
2015	Italy	5-14 years	0.05	0.08	0.03
2015	Italy	15-49 years	0.84	1.30	0.62
2015	Italy	50-69 years	2.11	3.13	1.49
2015	Italy	70+ years	2.05	3.05	1.24
2015	Italy	All Ages	1.31	1.97	0.91
2019	Italy	Under 5	1.30	1.87	0.61
2019	Italy	5-14 years	0.05	0.08	0.03
2019	Italy	15-49 years	0.92	1.27	0.66
2019	Italy	50-69 years	2.22	2.91	1.59
2019	Italy	70+ years	2.18	2.87	1.36
2019	Italy	All Ages	1.43	1.88	1.01
2000	Portugal	Under 5	3.57	6.12	1.86
2000	Portugal	5-14 years	0.56	0.88	0.22
2000	Portugal	15-49 years	6.12	7.75	3.62
2000	Portugal	50-69 years	5.83	7.14	4.10
2000	Portugal	70+ years	3.29	4.31	1.97
2000	Portugal	All Ages	5.00	6.06	3.37
2005	Portugal	Under 5	0.52	1.08	0.22
2005	Portugal	5-14 years	0.09	0.17	0.05
2005	Portugal	15-49 years	1.27	1.70	1.01
2005	Portugal	50-69 years	1.70	2.10	1.36
2005	Portugal	70+ years	1.00	1.37	0.74
2005	Portugal	All Ages	1.17	1.52	0.99
2010	Portugal	Under 5	0.28	0.71	0.08
2010	Portugal	5-14 years	0.03	0.09	0.01
2010	Portugal	15-49 years	0.89	1.20	0.70
2010	Portugal	50-69 years	1.33	1.60	1.11
2010	Portugal	70+ years	0.72	1.03	0.54
2010	Portugal	All Ages	0.86	1.10	0.72
2015	Portugal	Under 5	0.24	0.58	0.08
2015	Portugal	5-14 years	0.02	0.06	0.01
2015	Portugal	15-49 years	0.64	0.81	0.50
2015	Portugal	50-69 years	1.14	1.36	0.93
2015	Portugal	70+ years	0.67	0.91	0.49
2015	Portugal	All Ages	0.70	0.85	0.59
2019	Portugal	Under 5	0.23	0.51	0.08
2019	Portugal	5-14 years	0.03	0.06	0.01
2019	Portugal	15-49 years	0.70	0.90	0.54
2019	Portugal	50-69 years	1.11	1.34	0.88
2019	Portugal	70+ years	0.66	0.90	0.48
2019	Portugal	All Ages	0.72	0.89	0.60
2000	Spain	Under 5	1.83	3.25	0.61
2000	Spain	5-14 years	0.05	0.08	0.03
2000	Spain	15-49 years	0.42	0.67	0.30
2000	Spain	50-69 years	0.68	1.15	0.47
2000	Spain	70+ years	0.55	0.87	0.38
2000	Spain	All Ages	0.52	0.78	0.39
2005	Spain	Under 5	0.98	2.15	0.40
2005	Spain	5-14 years	0.03	0.06	0.01
2005	Spain	15-49 years	0.36	0.63	0.25
2005	Spain	50-69 years	0.60	1.06	0.40
2005	Spain	70+ years	0.43	0.78	0.28

2005	Spain	All Ages	0.42	0.73	0.31
2010	Spain	Under 5	1.07	1.76	0.54
2010	Spain	5-14 years	0.03	0.06	0.02
2010	Spain	15-49 years	0.41	0.56	0.32
2010	Spain	50-69 years	0.68	0.94	0.51
2010	Spain	70+ years	0.50	0.69	0.34
2010	Spain	All Ages	0.48	0.64	0.37
2015	Spain	Under 5	0.81	1.42	0.35
2015	Spain	5-14 years	0.03	0.05	0.02
2015	Spain	15-49 years	0.41	0.53	0.31
2015	Spain	50-69 years	0.72	0.91	0.50
2015	Spain	70+ years	0.54	0.70	0.31
2015	Spain	All Ages	0.49	0.59	0.34
2019	Spain	Under 5	0.73	1.41	0.28
2019	Spain	5-14 years	0.03	0.06	0.01
2019	Spain	15-49 years	0.42	0.54	0.31
2019	Spain	50-69 years	0.70	0.90	0.49
2019	Spain	70+ years	0.53	0.70	0.31
2019	Spain	All Ages	0.48	0.59	0.33

Table S41- Rates and 95% uncertainty levels (UL) of **acute HBV years lived with disability** (YLDs) per 100,000 population in Greece, Italy, Portugal and Spain from 2000 to 2019 by age group (Global Burden of Disease Study 2019).

Year	Country	Age	YLDs (95% UL)	95% UL (upper)	95% UL (lower)
2000	Greece	Under 5	0.06	0.09	0.03
2000	Greece	5-14 years	0.41	0.65	0.24
2000	Greece	15-49 years	1.64	2.67	0.89
2000	Greece	50-69 years	1.29	2.40	0.62
2000	Greece	70+ years	0.83	1.81	0.29
2000	Greece	All Ages	1.27	2.03	0.72
2005	Greece	Under 5	0.04	0.06	0.02
2005	Greece	5-14 years	0.26	0.43	0.14
2005	Greece	15-49 years	1.63	2.65	0.92
2005	Greece	50-69 years	1.27	2.28	0.61
2005	Greece	70+ years	0.83	1.65	0.34
2005	Greece	All Ages	1.24	2.04	0.71
2010	Greece	Under 5	0.03	0.05	0.01
2010	Greece	5-14 years	0.05	0.08	0.03
2010	Greece	15-49 years	1.64	2.75	0.91
2010	Greece	50-69 years	1.24	2.28	0.56
2010	Greece	70+ years	0.79	1.62	0.29
2010	Greece	All Ages	1.19	1.95	0.70
2015	Greece	Under 5	0.02	0.04	0.01
2015	Greece	5-14 years	0.04	0.06	0.02
2015	Greece	15-49 years	1.63	2.69	0.87
2015	Greece	50-69 years	1.22	2.21	0.54
2015	Greece	70+ years	0.75	1.53	0.28
2015	Greece	All Ages	1.16	1.88	0.66
2019	Greece	Under 5	0.02	0.04	0.01
2019	Greece	5-14 years	0.03	0.05	0.02
2019	Greece	15-49 years	1.57	2.54	0.89
2019	Greece	50-69 years	1.24	2.22	0.57
2019	Greece	70+ years	0.76	1.51	0.29
2019	Greece	All Ages	1.13	1.75	0.67
2000	Italy	Under 5	0.02	0.03	0.01
2000	Italy	5-14 years	0.06	0.10	0.03
2000	Italy	15-49 years	0.70	1.17	0.39
2000	Italy	50-69 years	0.57	1.00	0.28
2000	Italy	70+ years	0.35	0.68	0.16
2000	Italy	All Ages	0.53	0.87	0.31
2005	Italy	Under 5	0.01	0.02	0.00
2005	Italy	5-14 years	0.02	0.04	0.01
2005	Italy	15-49 years	0.65	1.07	0.37
2005	Italy	50-69 years	0.51	0.93	0.26
2005	Italy	70+ years	0.31	0.62	0.14
2005	Italy	All Ages	0.48	0.77	0.28
2010	Italy	Under 5	0.01	0.02	0.00
2010	Italy	5-14 years	0.01	0.02	0.00
2010	Italy	15-49 years	0.54	0.90	0.29

2010	Italy	50-69 years	0.45	0.82	0.21
2010	Italy	70+ years	0.28	0.53	0.11
2010	Italy	All Ages	0.40	0.66	0.23
2015	Italy	Under 5	0.01	0.02	0.00
2015	Italy	5-14 years	0.01	0.02	0.00
2015	Italy	15-49 years	0.43	0.75	0.22
2015	Italy	50-69 years	0.43	0.77	0.21
2015	Italy	70+ years	0.26	0.53	0.11
2015	Italy	All Ages	0.35	0.57	0.19
2019	Italy	Under 5	0.01	0.02	0.00
2019	Italy	5-14 years	0.01	0.02	0.00
2019	Italy	15-49 years	0.33	0.55	0.17
2019	Italy	50-69 years	0.42	0.79	0.20
2019	Italy	70+ years	0.25	0.51	0.10
2019	Italy	All Ages	0.30	0.50	0.15
2000	Portugal	Under 5	0.10	0.18	0.05
2000	Portugal	5-14 years	0.24	0.43	0.13
2000	Portugal	15-49 years	0.64	1.15	0.33
2000	Portugal	50-69 years	0.40	0.80	0.15
2000	Portugal	70+ years	0.22	0.49	0.07
2000	Portugal	All Ages	0.47	0.82	0.25
2005	Portugal	Under 5	0.03	0.05	0.01
2005	Portugal	5-14 years	0.15	0.28	0.08
2005	Portugal	15-49 years	0.68	1.16	0.35
2005	Portugal	50-69 years	0.42	0.83	0.15
2005	Portugal	70+ years	0.23	0.54	0.06
2005	Portugal	All Ages	0.48	0.80	0.25
2010	Portugal	Under 5	0.02	0.04	0.01
2010	Portugal	5-14 years	0.06	0.10	0.03
2010	Portugal	15-49 years	0.66	1.15	0.31
2010	Portugal	50-69 years	0.40	0.79	0.15
2010	Portugal	70+ years	0.22	0.48	0.06
2010	Portugal	All Ages	0.45	0.76	0.22
2015	Portugal	Under 5	0.02	0.03	0.01
2015	Portugal	5-14 years	0.02	0.04	0.01
2015	Portugal	15-49 years	0.63	1.16	0.30
2015	Portugal	50-69 years	0.39	0.75	0.13
2015	Portugal	70+ years	0.21	0.46	0.05
2015	Portugal	All Ages	0.42	0.72	0.20
2019	Portugal	Under 5	0.02	0.03	0.01
2019	Portugal	5-14 years	0.02	0.03	0.01
2019	Portugal	15-49 years	0.56	1.00	0.27
2019	Portugal	50-69 years	0.39	0.79	0.14
2019	Portugal	70+ years	0.21	0.47	0.06
2019	Portugal	All Ages	0.39	0.68	0.19
2000	Spain	Under 5	0.05	0.09	0.03
2000	Spain	5-14 years	0.14	0.24	0.08
2000	Spain	15-49 years	0.71	1.20	0.38
2000	Spain	50-69 years	0.54	1.00	0.25
2000	Spain	70+ years	0.35	0.72	0.13
2000	Spain	All Ages	0.54	0.90	0.31
2005	Spain	Under 5	0.02	0.03	0.01
2005	Spain	5-14 years	0.05	0.09	0.03

2005	Spain	15-49 years	0.74	1.25	0.40
2005	Spain	50-69 years	0.52	0.96	0.24
2005	Spain	70+ years	0.33	0.69	0.13
2005	Spain	All Ages	0.54	0.91	0.30
2010	Spain	Under 5	0.01	0.03	0.01
2010	Spain	5-14 years	0.03	0.06	0.02
2010	Spain	15-49 years	0.70	1.20	0.38
2010	Spain	50-69 years	0.52	0.99	0.22
2010	Spain	70+ years	0.33	0.69	0.12
2010	Spain	All Ages	0.52	0.85	0.28
2015	Spain	Under 5	0.01	0.02	0.01
2015	Spain	5-14 years	0.01	0.03	0.01
2015	Spain	15-49 years	0.70	1.20	0.37
2015	Spain	50-69 years	0.54	0.97	0.26
2015	Spain	70+ years	0.34	0.69	0.13
2015	Spain	All Ages	0.51	0.86	0.29
2019	Spain	Under 5	0.01	0.02	0.00
2019	Spain	5-14 years	0.01	0.03	0.01
2019	Spain	15-49 years	0.60	1.01	0.31
2019	Spain	50-69 years	0.52	0.95	0.23
2019	Spain	70+ years	0.34	0.66	0.13
2019	Spain	All Ages	0.46	0.76	0.26

Table S42- Rates and 95% uncertainty levels (UL) of **acute HBV disability-adjusted life years** (DALYs) per 100,000 population in Greece, Italy, Portugal and Spain from 2000 to 2019 by age group (Global Burden of Disease Study 2019).

Year	Country	Age	DALYs (95% UL)	95% UL (upper)	95% UL (lower)
2000	Greece	Under 5	1.35	3.31	0.57
2000	Greece	5-14 years	0.56	0.82	0.37
2000	Greece	15-49 years	3.54	4.57	2.73
2000	Greece	50-69 years	7.86	9.40	6.38
2000	Greece	70+ years	11.6	14.3	8.28
2000	Greece	All Ages	4.96	5.88	4.16
2005	Greece	Under 5	1.79	4.49	0.72
2005	Greece	5-14 years	0.49	0.70	0.32
2005	Greece	15-49 years	4.81	6.01	3.84
2005	Greece	50-69 years	11.9	13.9	9.85
2005	Greece	70+ years	20.5	25.5	14.7
2005	Greece	All Ages	7.82	9.04	6.48
2010	Greece	Under 5	3.03	8.03	0.95
2010	Greece	5-14 years	0.47	0.70	0.26
2010	Greece	15-49 years	7.81	9.38	6.34
2010	Greece	50-69 years	22.3	25.4	18.8
2010	Greece	70+ years	46.5	57.8	32.6
2010	Greece	All Ages	15.7	17.9	12.8
2015	Greece	Under 5	1.37	3.90	0.40
2015	Greece	5-14 years	0.19	0.28	0.12
2015	Greece	15-49 years	4.32	5.48	3.40
2015	Greece	50-69 years	10.9	12.7	9.36
2015	Greece	70+ years	16.2	19.6	11.9
2015	Greece	All Ages	7.28	8.29	6.25
2019	Greece	Under 5	1.23	3.19	0.38
2019	Greece	5-14 years	0.19	0.31	0.11
2019	Greece	15-49 years	4.49	5.73	3.51
2019	Greece	50-69 years	11.0	12.9	9.06
2019	Greece	70+ years	15.8	19.5	11.5
2019	Greece	All Ages	7.50	8.57	6.29
2000	Italy	Under 5	2.90	4.13	1.37
2000	Italy	5-14 years	0.19	0.26	0.09
2000	Italy	15-49 years	2.01	2.57	1.54
2000	Italy	50-69 years	3.33	4.02	2.53
2000	Italy	70+ years	2.75	3.40	1.77
2000	Italy	All Ages	2.29	2.76	1.72
2005	Italy	Under 5	2.30	3.56	0.87
2005	Italy	5-14 years	0.11	0.14	0.07
2005	Italy	15-49 years	1.81	2.53	1.30
2005	Italy	50-69 years	3.26	4.63	2.31
2005	Italy	70+ years	2.72	4.00	1.78
2005	Italy	All Ages	2.15	3.03	1.54
2010	Italy	Under 5	1.76	3.16	0.66
2010	Italy	5-14 years	0.08	0.12	0.05
2010	Italy	15-49 years	1.53	2.27	1.11
2010	Italy	50-69 years	2.90	4.27	2.01
2010	Italy	70+ years	2.56	3.95	1.55

2010	Italy	All Ages	1.89	2.84	1.31
2015	Italy	Under 5	1.32	2.20	0.59
2015	Italy	5-14 years	0.06	0.09	0.04
2015	Italy	15-49 years	1.28	1.80	0.96
2015	Italy	50-69 years	2.55	3.65	1.91
2015	Italy	70+ years	2.31	3.34	1.53
2015	Italy	All Ages	1.66	2.36	1.23
2019	Italy	Under 5	1.32	1.88	0.62
2019	Italy	5-14 years	0.07	0.09	0.04
2019	Italy	15-49 years	1.25	1.66	0.95
2019	Italy	50-69 years	2.64	3.39	1.98
2019	Italy	70+ years	2.44	3.11	1.62
2019	Italy	All Ages	1.73	2.24	1.28
2000	Portugal	Under 5	3.68	6.24	1.96
2000	Portugal	5-14 years	0.81	1.17	0.45
2000	Portugal	15-49 years	6.76	8.46	4.26
2000	Portugal	50-69 years	6.23	7.67	4.47
2000	Portugal	70+ years	3.51	4.59	2.16
2000	Portugal	All Ages	5.48	6.61	3.81
2005	Portugal	Under 5	0.55	1.11	0.25
2005	Portugal	5-14 years	0.25	0.39	0.16
2005	Portugal	15-49 years	1.95	2.57	1.49
2005	Portugal	50-69 years	2.12	2.70	1.66
2005	Portugal	70+ years	1.23	1.70	0.90
2005	Portugal	All Ages	1.65	2.09	1.34
2010	Portugal	Under 5	0.31	0.74	0.11
2010	Portugal	5-14 years	0.09	0.16	0.05
2010	Portugal	15-49 years	1.56	2.15	1.13
2010	Portugal	50-69 years	1.74	2.23	1.38
2010	Portugal	70+ years	0.95	1.32	0.68
2010	Portugal	All Ages	1.31	1.71	1.02
2015	Portugal	Under 5	0.26	0.60	0.10
2015	Portugal	5-14 years	0.05	0.09	0.02
2015	Portugal	15-49 years	1.27	1.79	0.90
2015	Portugal	50-69 years	1.53	1.98	1.18
2015	Portugal	70+ years	0.89	1.21	0.64
2015	Portugal	All Ages	1.12	1.43	0.87
2019	Portugal	Under 5	0.25	0.53	0.10
2019	Portugal	5-14 years	0.05	0.09	0.02
2019	Portugal	15-49 years	1.27	1.74	0.91
2019	Portugal	50-69 years	1.50	1.97	1.14
2019	Portugal	70+ years	0.88	1.20	0.63
2019	Portugal	All Ages	1.12	1.45	0.87
2000	Spain	Under 5	1.89	3.31	0.68
2000	Spain	5-14 years	0.20	0.29	0.13
2000	Spain	15-49 years	1.13	1.63	0.77
2000	Spain	50-69 years	1.22	1.84	0.83
2000	Spain	70+ years	0.90	1.31	0.62
2000	Spain	All Ages	1.06	1.45	0.78
2005	Spain	Under 5	1.00	2.17	0.41
2005	Spain	5-14 years	0.09	0.14	0.05
2005	Spain	15-49 years	1.11	1.67	0.71
2005	Spain	50-69 years	1.13	1.78	0.74

2005	Spain	70+ years	0.77	1.23	0.48
2005	Spain	All Ages	0.97	1.44	0.66
2010	Spain	Under 5	1.09	1.79	0.56
2010	Spain	5-14 years	0.07	0.10	0.05
2010	Spain	15-49 years	1.11	1.60	0.76
2010	Spain	50-69 years	1.21	1.74	0.86
2010	Spain	70+ years	0.84	1.24	0.57
2010	Spain	All Ages	1.00	1.36	0.74
2015	Spain	Under 5	0.83	1.44	0.37
2015	Spain	5-14 years	0.05	0.08	0.03
2015	Spain	15-49 years	1.12	1.63	0.78
2015	Spain	50-69 years	1.27	1.73	0.91
2015	Spain	70+ years	0.89	1.27	0.57
2015	Spain	All Ages	1.00	1.34	0.75
2019	Spain	Under 5	0.74	1.43	0.30
2019	Spain	5-14 years	0.05	0.08	0.03
2019	Spain	15-49 years	1.03	1.45	0.72
2019	Spain	50-69 years	1.23	1.70	0.86
2019	Spain	70+ years	0.87	1.23	0.57
2019	Spain	All Ages	0.94	1.25	0.69

Table S43- Rates and 95% uncertainty levels (UL) of **acute HCV prevalence** per 100,000 population in Greece, Italy, Portugal and Spain from 2000 to 2019 by age group (Global Burden of Disease Study 2019).

Year	Country	Age	Prevalence (95% UL)	95% UL (upper)	95% UL (lower)
2000	Greece	Under 5	2.99	3.94	2.14
2000	Greece	5-14 years	1.13	1.69	0.73
2000	Greece	15-49 years	1.47	1.83	1.14
2000	Greece	50-69 years	3.18	4.21	2.30
2000	Greece	70+ years	7.20	9.68	5.16
2000	Greece	All Ages	2.50	2.90	2.15
2005	Greece	Under 5	3.53	4.79	2.42
2005	Greece	5-14 years	1.48	2.19	0.90
2005	Greece	15-49 years	2.00	2.64	1.48
2005	Greece	50-69 years	4.39	6.04	3.09
2005	Greece	70+ years	8.44	12.5	5.84
2005	Greece	All Ages	3.37	4.03	2.83
2010	Greece	Under 5	3.51	4.80	2.42
2010	Greece	5-14 years	1.47	2.23	0.91
2010	Greece	15-49 years	2.01	2.59	1.49
2010	Greece	50-69 years	4.28	5.84	3.04
2010	Greece	70+ years	8.52	12.0	5.97
2010	Greece	All Ages	3.48	4.16	2.91
2015	Greece	Under 5	3.44	4.70	2.40
2015	Greece	5-14 years	1.44	2.18	0.89
2015	Greece	15-49 years	2.00	2.62	1.50
2015	Greece	50-69 years	4.30	5.97	3.07
2015	Greece	70+ years	8.73	12.6	6.11
2015	Greece	All Ages	3.63	4.49	3.00
2019	Greece	Under 5	3.48	4.70	2.44
2019	Greece	5-14 years	1.46	2.21	0.89
2019	Greece	15-49 years	2.06	2.67	1.53
2019	Greece	50-69 years	4.33	6.06	2.99
2019	Greece	70+ years	9.05	12.7	6.09
2019	Greece	All Ages	3.81	4.60	3.12
2000	Italy	Under 5	13.7	19.1	9.25
2000	Italy	5-14 years	8.67	12.1	5.49
2000	Italy	15-49 years	7.95	10.5	5.72
2000	Italy	50-69 years	19.8	27.6	13.3
2000	Italy	70+ years	20.0	30.3	12.8
2000	Italy	All Ages	12.7	15.2	10.7
2005	Italy	Under 5	11.8	16.3	7.98
2005	Italy	5-14 years	6.46	9.33	3.96
2005	Italy	15-49 years	6.72	8.90	4.82
2005	Italy	50-69 years	16.8	23.5	11.2
2005	Italy	70+ years	18.5	27.8	12.0
2005	Italy	All Ages	11.0	13.2	9.33
2010	Italy	Under 5	12.0	16.8	8.17
2010	Italy	5-14 years	6.53	9.64	4.01
2010	Italy	15-49 years	6.83	9.07	4.88
2010	Italy	50-69 years	14.4	20.6	9.52
2010	Italy	70+ years	17.4	26.7	11.2
2010	Italy	All Ages	10.4	12.6	8.76

2015	Italy	Under 5	10.8	14.8	7.48
2015	Italy	5-14 years	5.49	7.97	3.40
2015	Italy	15-49 years	5.66	7.49	4.10
2015	Italy	50-69 years	12.2	17.3	8.24
2015	Italy	70+ years	16.9	25.3	10.8
2015	Italy	All Ages	9.38	11.3	7.75
2019	Italy	Under 5	11.2	15.3	7.64
2019	Italy	5-14 years	5.62	8.25	3.48
2019	Italy	15-49 years	5.77	7.63	4.12
2019	Italy	50-69 years	12.4	17.9	8.27
2019	Italy	70+ years	17.3	26.2	11.2
2019	Italy	All Ages	9.79	11.9	8.08
2000	Portugal	Under 5	6.39	8.74	4.29
2000	Portugal	5-14 years	3.12	4.64	1.92
2000	Portugal	15-49 years	3.80	4.93	2.90
2000	Portugal	50-69 years	6.88	9.49	4.75
2000	Portugal	70+ years	10.5	15.3	7.04
2000	Portugal	All Ages	5.26	6.18	4.42
2005	Portugal	Under 5	6.19	8.44	4.23
2005	Portugal	5-14 years	2.93	4.31	1.79
2005	Portugal	15-49 years	3.70	4.83	2.84
2005	Portugal	50-69 years	6.73	9.27	4.71
2005	Portugal	70+ years	10.3	14.9	7.12
2005	Portugal	All Ages	5.24	6.16	4.48
2010	Portugal	Under 5	6.07	8.37	4.13
2010	Portugal	5-14 years	2.82	4.19	1.74
2010	Portugal	15-49 years	3.52	4.52	2.69
2010	Portugal	50-69 years	6.69	9.30	4.71
2010	Portugal	70+ years	10.6	14.9	7.21
2010	Portugal	All Ages	5.28	6.29	4.50
2015	Portugal	Under 5	5.86	7.98	3.97
2015	Portugal	5-14 years	2.64	3.82	1.66
2015	Portugal	15-49 years	3.44	4.47	2.58
2015	Portugal	50-69 years	6.71	9.29	4.81
2015	Portugal	70+ years	10.7	15.9	7.38
2015	Portugal	All Ages	5.37	6.57	4.53
2019	Portugal	Under 5	5.94	8.27	4.09
2019	Portugal	5-14 years	2.62	3.97	1.58
2019	Portugal	15-49 years	3.39	4.35	2.56
2019	Portugal	50-69 years	6.71	9.24	4.73
2019	Portugal	70+ years	11.0	16.5	7.55
2019	Portugal	All Ages	5.54	6.62	4.70
2000	Spain	Under 5	6.22	8.41	4.26
2000	Spain	5-14 years	2.96	4.10	1.99
2000	Spain	15-49 years	3.75	4.51	3.06
2000	Spain	50-69 years	7.83	9.63	6.26
2000	Spain	70+ years	10.9	14.9	8.11
2000	Spain	All Ages	5.48	6.02	4.98
2005	Spain	Under 5	5.48	7.29	3.86
2005	Spain	5-14 years	2.31	3.27	1.53
2005	Spain	15-49 years	3.08	3.70	2.45
2005	Spain	50-69 years	6.65	8.43	5.19
2005	Spain	70+ years	11.5	15.2	8.59

2005	Spain	All Ages	4.91	5.41	4.47
2010	Spain	Under 5	5.95	8.42	4.00
2010	Spain	5-14 years	2.78	4.10	1.72
2010	Spain	15-49 years	3.91	5.18	2.92
2010	Spain	50-69 years	8.40	11.6	5.93
2010	Spain	70+ years	13.5	19.9	9.52
2010	Spain	All Ages	6.08	7.24	5.20
2015	Spain	Under 5	5.59	7.74	3.83
2015	Spain	5-14 years	2.57	3.86	1.54
2015	Spain	15-49 years	3.76	4.97	2.76
2015	Spain	50-69 years	8.00	10.6	5.98
2015	Spain	70+ years	13.2	19.2	8.79
2015	Spain	All Ages	6.01	7.22	5.18
2019	Spain	Under 5	5.58	7.65	3.88
2019	Spain	5-14 years	2.52	3.75	1.59
2019	Spain	15-49 years	3.69	4.90	2.74
2019	Spain	50-69 years	8.01	10.8	5.74
2019	Spain	70+ years	13.7	20.2	9.64
2019	Spain	All Ages	6.23	7.30	5.28

Table S44- Rates and 95% uncertainty levels (UL) of **acute HCV incidence** per 100,000 population in Greece, Italy, Portugal and Spain from 2000 to 2019 by age group (Global Burden of Disease Study 2019).

Year	Country	Age	incidence (95% UL)	95% UL (upper)	95% UL (lower)
2000	Greece	Under 5	25.9	34.1	18.6
2000	Greece	5-14 years	9.83	14.6	6.37
2000	Greece	15-49 years	12.7	15.9	9.93
2000	Greece	50-69 years	27.6	36.5	19.9
2000	Greece	70+ years	62.4	83.9	44.7
2000	Greece	All Ages	21.7	25.1	18.6
2005	Greece	Under 5	30.6	41.5	21.0
2005	Greece	5-14 years	12.8	19.0	7.83
2005	Greece	15-49 years	17.4	22.9	12.8
2005	Greece	50-69 years	38.0	52.3	26.8
2005	Greece	70+ years	73.1	108.	50.6
2005	Greece	All Ages	29.2	35.0	24.5
2010	Greece	Under 5	30.4	41.6	21.0
2010	Greece	5-14 years	12.7	19.3	7.93
2010	Greece	15-49 years	17.4	22.5	12.9
2010	Greece	50-69 years	37.1	50.6	26.3
2010	Greece	70+ years	73.8	104.	51.7
2010	Greece	All Ages	30.2	36.0	25.3
2015	Greece	Under 5	29.8	40.7	20.8
2015	Greece	5-14 years	12.5	18.9	7.72
2015	Greece	15-49 years	17.4	22.7	13.0
2015	Greece	50-69 years	37.3	51.7	26.6
2015	Greece	70+ years	75.6	109.	52.9
2015	Greece	All Ages	31.4	38.9	26.0
2019	Greece	Under 5	30.1	40.7	21.2
2019	Greece	5-14 years	12.7	19.2	7.79
2019	Greece	15-49 years	17.8	23.1	13.2
2019	Greece	50-69 years	37.5	52.5	25.9
2019	Greece	70+ years	78.5	110.	52.8
2019	Greece	All Ages	33.0	39.9	27.0
2000	Italy	Under 5	119.	165.	80.2
2000	Italy	5-14 years	75.2	105.	47.6
2000	Italy	15-49 years	68.9	91.3	49.6
2000	Italy	50-69 years	171.	239.	115.
2000	Italy	70+ years	173.	262.	111.
2000	Italy	All Ages	110.	132.	93.0
2005	Italy	Under 5	102.	141.	69.2
2005	Italy	5-14 years	56.0	80.8	34.3
2005	Italy	15-49 years	58.3	77.1	41.7
2005	Italy	50-69 years	145.	203.	97.3
2005	Italy	70+ years	160.	241.	104.
2005	Italy	All Ages	95.3	114.	80.8
2010	Italy	Under 5	104.	145.	70.8
2010	Italy	5-14 years	56.6	83.5	34.8

2010	Italy	15-49 years	59.2	78.6	42.3
2010	Italy	50-69 years	125.	178.	82.5
2010	Italy	70+ years	151.	231.	97.5
2010	Italy	All Ages	90.8	109.	76.0
2015	Italy	Under 5	94.3	128.	64.9
2015	Italy	5-14 years	47.6	69.1	29.4
2015	Italy	15-49 years	49.0	64.9	35.6
2015	Italy	50-69 years	106.	150.	71.4
2015	Italy	70+ years	146.	219.	94.1
2015	Italy	All Ages	81.3	98.4	67.2
2019	Italy	Under 5	97.1	132.	66.2
2019	Italy	5-14 years	48.7	71.5	30.1
2019	Italy	15-49 years	50.0	66.1	35.7
2019	Italy	50-69 years	107.	155.	71.7
2019	Italy	70+ years	150.	227.	97.1
2019	Italy	All Ages	84.8	103.	70.1
2000	Portugal	Under 5	55.4	75.7	37.2
2000	Portugal	5-14 years	27.0	40.2	16.6
2000	Portugal	15-49 years	33.0	42.7	25.1
2000	Portugal	50-69 years	59.6	82.3	41.1
2000	Portugal	70+ years	91.7	132.	61.0
2000	Portugal	All Ages	45.6	53.6	38.3
2005	Portugal	Under 5	53.6	73.1	36.6
2005	Portugal	5-14 years	25.4	37.3	15.5
2005	Portugal	15-49 years	32.0	41.9	24.6
2005	Portugal	50-69 years	58.3	80.4	40.9
2005	Portugal	70+ years	89.9	129.	61.7
2005	Portugal	All Ages	45.4	53.4	38.8
2010	Portugal	Under 5	52.6	72.5	35.8
2010	Portugal	5-14 years	24.4	36.3	15.1
2010	Portugal	15-49 years	30.5	39.2	23.3
2010	Portugal	50-69 years	58.0	80.6	40.8
2010	Portugal	70+ years	92.1	129.	62.5
2010	Portugal	All Ages	45.8	54.5	39.0
2015	Portugal	Under 5	50.8	69.1	34.4
2015	Portugal	5-14 years	22.8	33.1	14.4
2015	Portugal	15-49 years	29.8	38.8	22.4
2015	Portugal	50-69 years	58.1	80.5	41.7
2015	Portugal	70+ years	93.1	138.	64.0
2015	Portugal	All Ages	46.6	56.9	39.3
2019	Portugal	Under 5	51.5	71.7	35.5
2019	Portugal	5-14 years	22.7	34.4	13.7
2019	Portugal	15-49 years	29.4	37.7	22.2
2019	Portugal	50-69 years	58.1	80.1	41.0
2019	Portugal	70+ years	96.0	143.	65.4
2019	Portugal	All Ages	48.0	57.3	40.7
2000	Spain	Under 5	53.9	72.9	36.9
2000	Spain	5-14 years	25.7	35.6	17.2
2000	Spain	15-49 years	32.5	39.1	26.5
2000	Spain	50-69 years	67.8	83.4	54.2

2000	Spain	70+ years	95.2	129.	70.3
2000	Spain	All Ages	47.5	52.1	43.1
2005	Spain	Under 5	47.4	63.2	33.4
2005	Spain	5-14 years	20.1	28.3	13.2
2005	Spain	15-49 years	26.7	32.1	21.3
2005	Spain	50-69 years	57.6	73.0	45.0
2005	Spain	70+ years	100.	132.	74.5
2005	Spain	All Ages	42.5	46.9	38.7
2010	Spain	Under 5	51.6	72.9	34.7
2010	Spain	5-14 years	24.1	35.6	14.9
2010	Spain	15-49 years	33.9	44.9	25.3
2010	Spain	50-69 years	72.8	100.	51.4
2010	Spain	70+ years	117.	172.	82.5
2010	Spain	All Ages	52.7	62.7	45.1
2015	Spain	Under 5	48.5	67.1	33.2
2015	Spain	5-14 years	22.3	33.4	13.4
2015	Spain	15-49 years	32.6	43.0	23.9
2015	Spain	50-69 years	69.3	92.3	51.8
2015	Spain	70+ years	115.	166.	76.1
2015	Spain	All Ages	52.1	62.5	44.9
2019	Spain	Under 5	48.4	66.3	33.6
2019	Spain	5-14 years	21.9	32.5	13.8
2019	Spain	15-49 years	32.0	42.4	23.8
2019	Spain	50-69 years	69.4	93.7	49.8
2019	Spain	70+ years	119.	175.	83.5
2019	Spain	All Ages	54.0	63.3	45.7

Table S45- Rates and 95% uncertainty levels (UL) of **acute HCV deaths** per 100,000 population in Greece, Italy, Portugal and Spain from 2000 to 2019 by age group (Global Burden of Disease Study 2019).

Year	Country	Age	Deaths (95% UL)	95% UL (upper)	95% UL (lower)
2000	Greece	Under 5	0.007	0.013	0.002
2000	Greece	5-14 years	5.215	0.000	7.356
2000	Greece	15-49 years	0.002	0.005	0.000
2000	Greece	50-69 years	0.010	0.030	0.002
2000	Greece	70+ years	0.064	0.201	0.017
2000	Greece	All Ages	0.010	0.031	0.003
2005	Greece	Under 5	0.010	0.019	0.002
2005	Greece	5-14 years	7.257	0.000	1.002
2005	Greece	15-49 years	0.003	0.008	0.001
2005	Greece	50-69 years	0.014	0.043	0.003
2005	Greece	70+ years	0.105	0.336	0.027
2005	Greece	All Ages	0.018	0.055	0.005
2010	Greece	Under 5	0.020	0.037	0.004
2010	Greece	5-14 years	0.000	0.000	2.503
2010	Greece	15-49 years	0.006	0.016	0.002
2010	Greece	50-69 years	0.027	0.085	0.009
2010	Greece	70+ years	0.291	0.881	0.098
2010	Greece	All Ages	0.051	0.149	0.020
2015	Greece	Under 5	0.009	0.017	0.002
2015	Greece	5-14 years	6.362	0.000	2.051
2015	Greece	15-49 years	0.003	0.007	0.001
2015	Greece	50-69 years	0.014	0.037	0.006
2015	Greece	70+ years	0.122	0.273	0.065
2015	Greece	All Ages	0.024	0.055	0.014
2019	Greece	Under 5	0.007	0.015	0.001
2019	Greece	5-14 years	7.033	0.000	2.208
2019	Greece	15-49 years	0.003	0.007	0.001
2019	Greece	50-69 years	0.013	0.036	0.005
2019	Greece	70+ years	0.110	0.274	0.055
2019	Greece	All Ages	0.023	0.057	0.012
2000	Italy	Under 5	0.005	0.018	0.002
2000	Italy	5-14 years	0.000	0.001	7.361
2000	Italy	15-49 years	0.006	0.009	0.003
2000	Italy	50-69 years	0.035	0.054	0.013
2000	Italy	70+ years	0.115	0.164	0.033
2000	Italy	All Ages	0.026	0.038	0.009
2005	Italy	Under 5	0.004	0.018	0.001
2005	Italy	5-14 years	0.000	0.000	6.987
2005	Italy	15-49 years	0.005	0.010	0.002
2005	Italy	50-69 years	0.029	0.044	0.012
2005	Italy	70+ years	0.102	0.135	0.034
2005	Italy	All Ages	0.024	0.033	0.009
2010	Italy	Under 5	0.003	0.015	0.001
2010	Italy	5-14 years	0.000	0.000	5.054

2010	Italy	15-49 years	0.002	0.009	0.001
2010	Italy	50-69 years	0.012	0.034	0.007
2010	Italy	70+ years	0.043	0.109	0.025
2010	Italy	All Ages	0.010	0.029	0.006
2015	Italy	Under 5	0.002	0.010	0.000
2015	Italy	5-14 years	6.610	0.000	3.693
2015	Italy	15-49 years	0.001	0.007	0.000
2015	Italy	50-69 years	0.009	0.027	0.005
2015	Italy	70+ years	0.031	0.089	0.018
2015	Italy	All Ages	0.008	0.024	0.005
2019	Italy	Under 5	0.002	0.007	0.000
2019	Italy	5-14 years	7.568	0.000	3.730
2019	Italy	15-49 years	0.002	0.006	0.000
2019	Italy	50-69 years	0.009	0.024	0.005
2019	Italy	70+ years	0.035	0.081	0.022
2019	Italy	All Ages	0.009	0.023	0.006
2000	Portugal	Under 5	0.012	0.028	0.005
2000	Portugal	5-14 years	0.000	0.002	0.000
2000	Portugal	15-49 years	0.024	0.059	0.012
2000	Portugal	50-69 years	0.037	0.084	0.018
2000	Portugal	70+ years	0.058	0.118	0.030
2000	Portugal	All Ages	0.028	0.057	0.016
2005	Portugal	Under 5	0.004	0.007	0.002
2005	Portugal	5-14 years	0.000	0.000	8.480
2005	Portugal	15-49 years	0.011	0.016	0.002
2005	Portugal	50-69 years	0.020	0.030	0.007
2005	Portugal	70+ years	0.034	0.048	0.015
2005	Portugal	All Ages	0.014	0.019	0.005
2010	Portugal	Under 5	0.002	0.004	0.000
2010	Portugal	5-14 years	8.597	0.000	3.617
2010	Portugal	15-49 years	0.006	0.009	0.001
2010	Portugal	50-69 years	0.012	0.017	0.005
2010	Portugal	70+ years	0.021	0.030	0.008
2010	Portugal	All Ages	0.009	0.012	0.003
2015	Portugal	Under 5	0.001	0.003	0.000
2015	Portugal	5-14 years	8.864	0.000	2.717
2015	Portugal	15-49 years	0.003	0.005	0.001
2015	Portugal	50-69 years	0.007	0.011	0.002
2015	Portugal	70+ years	0.013	0.022	0.005
2015	Portugal	All Ages	0.005	0.007	0.002
2019	Portugal	Under 5	0.001	0.003	0.000
2019	Portugal	5-14 years	8.393	0.000	2.586
2019	Portugal	15-49 years	0.003	0.005	0.001
2019	Portugal	50-69 years	0.006	0.011	0.002
2019	Portugal	70+ years	0.013	0.022	0.004
2019	Portugal	All Ages	0.005	0.008	0.002
2000	Spain	Under 5	0.008	0.028	0.003
2000	Spain	5-14 years	0.000	0.000	0.000
2000	Spain	15-49 years	0.010	0.013	0.005
2000	Spain	50-69 years	0.037	0.046	0.020

2000	Spain	70+ years	0.073	0.092	0.047
2000	Spain	All Ages	0.022	0.026	0.013
2005	Spain	Under 5	0.006	0.020	0.002
2005	Spain	5-14 years	0.000	0.000	0.000
2005	Spain	15-49 years	0.009	0.011	0.004
2005	Spain	50-69 years	0.030	0.038	0.015
2005	Spain	70+ years	0.063	0.079	0.036
2005	Spain	All Ages	0.019	0.023	0.011
2010	Spain	Under 5	0.005	0.012	0.002
2010	Spain	5-14 years	0.000	0.000	0.000
2010	Spain	15-49 years	0.006	0.008	0.003
2010	Spain	50-69 years	0.020	0.027	0.012
2010	Spain	70+ years	0.043	0.058	0.028
2010	Spain	All Ages	0.013	0.017	0.008
2015	Spain	Under 5	0.003	0.008	0.001
2015	Spain	5-14 years	0.000	0.000	8.281
2015	Spain	15-49 years	0.004	0.006	0.002
2015	Spain	50-69 years	0.015	0.022	0.009
2015	Spain	70+ years	0.031	0.051	0.021
2015	Spain	All Ages	0.010	0.015	0.007
2019	Spain	Under 5	0.003	0.007	0.001
2019	Spain	5-14 years	0.000	0.000	7.995
2019	Spain	15-49 years	0.005	0.007	0.002
2019	Spain	50-69 years	0.014	0.021	0.008
2019	Spain	70+ years	0.032	0.049	0.021
2019	Spain	All Ages	0.010	0.015	0.007

Table S46- Rates and 95% uncertainty levels (UL) of **acute HCV years of life lost** (YLLs) per 100,000 population in Greece, Italy, Portugal and Spain from 2000 to 2019 by age group (Global Burden of Disease Study 2019).

Year	Country	Age	YLLs (95% UL)	95% UL (upper)	95% UL (lower)
2000	Greece	Under 5	0.651	1.204	0.199
2000	Greece	5-14 years	0.004	0.015	0.000
2000	Greece	15-49 years	0.097	0.263	0.044
2000	Greece	50-69 years	0.299	0.880	0.090
2000	Greece	70+ years	0.843	2.634	0.209
2000	Greece	All Ages	0.239	0.632	0.100
2005	Greece	Under 5	0.929	1.755	0.241
2005	Greece	5-14 years	0.005	0.023	0.000
2005	Greece	15-49 years	0.139	0.407	0.060
2005	Greece	50-69 years	0.408	1.272	0.116
2005	Greece	70+ years	1.391	4.540	0.340
2005	Greece	All Ages	0.380	1.070	0.154
2010	Greece	Under 5	1.802	3.356	0.356
2010	Greece	5-14 years	0.010	0.046	0.001
2010	Greece	15-49 years	0.301	0.769	0.137
2010	Greece	50-69 years	0.800	2.482	0.277
2010	Greece	70+ years	3.716	11.22	1.265
2010	Greece	All Ages	0.949	2.476	0.441
2015	Greece	Under 5	0.810	1.511	0.178
2015	Greece	5-14 years	0.004	0.015	0.001
2015	Greece	15-49 years	0.178	0.344	0.083
2015	Greece	50-69 years	0.416	1.106	0.204
2015	Greece	70+ years	1.497	3.265	0.805
2015	Greece	All Ages	0.453	0.948	0.285
2019	Greece	Under 5	0.686	1.377	0.143
2019	Greece	5-14 years	0.005	0.014	0.001
2019	Greece	15-49 years	0.178	0.349	0.079
2019	Greece	50-69 years	0.387	1.058	0.172
2019	Greece	70+ years	1.309	3.218	0.624
2019	Greece	All Ages	0.425	0.972	0.248
2000	Italy	Under 5	0.472	1.593	0.199
2000	Italy	5-14 years	0.047	0.081	0.005
2000	Italy	15-49 years	0.305	0.470	0.143
2000	Italy	50-69 years	1.008	1.548	0.397
2000	Italy	70+ years	1.621	2.334	0.489
2000	Italy	All Ages	0.626	0.911	0.266
2005	Italy	Under 5	0.403	1.621	0.166
2005	Italy	5-14 years	0.025	0.037	0.005
2005	Italy	15-49 years	0.250	0.511	0.124
2005	Italy	50-69 years	0.843	1.289	0.375
2005	Italy	70+ years	1.441	1.893	0.482
2005	Italy	All Ages	0.544	0.829	0.245
2010	Italy	Under 5	0.284	1.338	0.091

2010	Italy	5-14 years	0.008	0.018	0.003
2010	Italy	15-49 years	0.120	0.419	0.059
2010	Italy	50-69 years	0.359	1.012	0.217
2010	Italy	70+ years	0.591	1.459	0.354
2010	Italy	All Ages	0.246	0.704	0.150
2015	Italy	Under 5	0.215	0.914	0.080
2015	Italy	5-14 years	0.005	0.012	0.002
2015	Italy	15-49 years	0.087	0.324	0.038
2015	Italy	50-69 years	0.257	0.790	0.140
2015	Italy	70+ years	0.425	1.155	0.255
2015	Italy	All Ages	0.183	0.557	0.104
2019	Italy	Under 5	0.193	0.705	0.085
2019	Italy	5-14 years	0.005	0.012	0.002
2019	Italy	15-49 years	0.094	0.303	0.044
2019	Italy	50-69 years	0.271	0.713	0.159
2019	Italy	70+ years	0.471	1.053	0.294
2019	Italy	All Ages	0.203	0.541	0.124
2000	Portugal	Under 5	1.107	2.483	0.444
2000	Portugal	5-14 years	0.052	0.176	0.015
2000	Portugal	15-49 years	1.230	3.011	0.609
2000	Portugal	50-69 years	1.135	2.596	0.544
2000	Portugal	70+ years	0.874	1.762	0.457
2000	Portugal	All Ages	1.034	2.245	0.585
2005	Portugal	Under 5	0.433	0.695	0.175
2005	Portugal	5-14 years	0.017	0.039	0.006
2005	Portugal	15-49 years	0.548	0.806	0.142
2005	Portugal	50-69 years	0.627	0.917	0.231
2005	Portugal	70+ years	0.507	0.727	0.216
2005	Portugal	All Ages	0.499	0.674	0.171
2010	Portugal	Under 5	0.243	0.391	0.085
2010	Portugal	5-14 years	0.006	0.017	0.002
2010	Portugal	15-49 years	0.319	0.473	0.090
2010	Portugal	50-69 years	0.381	0.543	0.155
2010	Portugal	70+ years	0.305	0.443	0.123
2010	Portugal	All Ages	0.296	0.402	0.107
2015	Portugal	Under 5	0.167	0.291	0.063
2015	Portugal	5-14 years	0.007	0.013	0.002
2015	Portugal	15-49 years	0.167	0.254	0.051
2015	Portugal	50-69 years	0.213	0.343	0.088
2015	Portugal	70+ years	0.189	0.311	0.073
2015	Portugal	All Ages	0.166	0.232	0.068
2019	Portugal	Under 5	0.139	0.276	0.046
2019	Portugal	5-14 years	0.006	0.014	0.002
2019	Portugal	15-49 years	0.177	0.280	0.052
2019	Portugal	50-69 years	0.204	0.336	0.084
2019	Portugal	70+ years	0.182	0.303	0.070
2019	Portugal	All Ages	0.168	0.239	0.067
2000	Spain	Under 5	0.753	2.530	0.271
2000	Spain	5-14 years	0.035	0.060	0.018
2000	Spain	15-49 years	0.517	0.674	0.275

2000	Spain	50-69 years	1.099	1.387	0.618
2000	Spain	70+ years	1.059	1.336	0.679
2000	Spain	All Ages	0.664	0.812	0.413
2005	Spain	Under 5	0.595	1.777	0.244
2005	Spain	5-14 years	0.023	0.042	0.012
2005	Spain	15-49 years	0.441	0.577	0.214
2005	Spain	50-69 years	0.925	1.168	0.476
2005	Spain	70+ years	0.892	1.124	0.515
2005	Spain	All Ages	0.565	0.688	0.317
2010	Spain	Under 5	0.449	1.138	0.210
2010	Spain	5-14 years	0.016	0.033	0.008
2010	Spain	15-49 years	0.309	0.404	0.165
2010	Spain	50-69 years	0.629	0.821	0.367
2010	Spain	70+ years	0.595	0.797	0.379
2010	Spain	All Ages	0.394	0.516	0.241
2015	Spain	Under 5	0.304	0.722	0.134
2015	Spain	5-14 years	0.013	0.028	0.006
2015	Spain	15-49 years	0.219	0.330	0.127
2015	Spain	50-69 years	0.451	0.678	0.288
2015	Spain	70+ years	0.421	0.671	0.286
2015	Spain	All Ages	0.285	0.433	0.194
2019	Spain	Under 5	0.284	0.640	0.122
2019	Spain	5-14 years	0.014	0.031	0.006
2019	Spain	15-49 years	0.237	0.347	0.134
2019	Spain	50-69 years	0.434	0.650	0.254
2019	Spain	70+ years	0.423	0.637	0.277
2019	Spain	All Ages	0.294	0.418	0.188

Table S47- Rates and 95% uncertainty levels (UL) of **acute HCV years lived with disability** (YLDs) per 100,000 population in Greece, Italy, Portugal and Spain from 2000 to 2019 by age group (Global Burden of Disease Study 2019).

Year	Country	Age	YLDs (95% UL)	95% UL (upper)	95% UL (lower)
2000	Greece	Under 5	0.04	0.08	0.01
2000	Greece	5-14 years	0.01	0.03	0.00
2000	Greece	15-49 years	0.02	0.04	0.00
2000	Greece	50-69 years	0.04	0.09	0.02
2000	Greece	70+ years	0.10	0.20	0.04
2000	Greece	All Ages	0.03	0.06	0.01
2005	Greece	Under 5	0.04	0.10	0.02
2005	Greece	5-14 years	0.02	0.04	0.00
2005	Greece	15-49 years	0.02	0.05	0.01
2005	Greece	50-69 years	0.06	0.12	0.02
2005	Greece	70+ years	0.11	0.25	0.05
2005	Greece	All Ages	0.04	0.09	0.02
2010	Greece	Under 5	0.04	0.10	0.02
2010	Greece	5-14 years	0.02	0.04	0.00
2010	Greece	15-49 years	0.02	0.05	0.01
2010	Greece	50-69 years	0.06	0.12	0.02
2010	Greece	70+ years	0.12	0.24	0.05
2010	Greece	All Ages	0.04	0.09	0.02
2015	Greece	Under 5	0.04	0.10	0.02
2015	Greece	5-14 years	0.02	0.04	0.00
2015	Greece	15-49 years	0.02	0.05	0.01
2015	Greece	50-69 years	0.06	0.12	0.02
2015	Greece	70+ years	0.12	0.24	0.05
2015	Greece	All Ages	0.05	0.10	0.02
2019	Greece	Under 5	0.04	0.10	0.02
2019	Greece	5-14 years	0.02	0.04	0.00
2019	Greece	15-49 years	0.02	0.05	0.01
2019	Greece	50-69 years	0.06	0.12	0.02
2019	Greece	70+ years	0.12	0.26	0.05
2019	Greece	All Ages	0.05	0.10	0.02
2000	Italy	Under 5	0.19	0.41	0.08
2000	Italy	5-14 years	0.12	0.26	0.05
2000	Italy	15-49 years	0.11	0.23	0.05
2000	Italy	50-69 years	0.27	0.58	0.11
2000	Italy	70+ years	0.28	0.57	0.11
2000	Italy	All Ages	0.17	0.35	0.08
2005	Italy	Under 5	0.16	0.35	0.06
2005	Italy	5-14 years	0.09	0.19	0.03
2005	Italy	15-49 years	0.09	0.19	0.04
2005	Italy	50-69 years	0.23	0.48	0.10
2005	Italy	70+ years	0.26	0.53	0.10
2005	Italy	All Ages	0.15	0.31	0.07
2010	Italy	Under 5	0.16	0.36	0.07

2010	Italy	5-14 years	0.09	0.19	0.03
2010	Italy	15-49 years	0.09	0.20	0.04
2010	Italy	50-69 years	0.20	0.42	0.08
2010	Italy	70+ years	0.24	0.50	0.10
2010	Italy	All Ages	0.14	0.29	0.07
2015	Italy	Under 5	0.15	0.32	0.06
2015	Italy	5-14 years	0.07	0.16	0.03
2015	Italy	15-49 years	0.07	0.16	0.03
2015	Italy	50-69 years	0.17	0.35	0.07
2015	Italy	70+ years	0.23	0.48	0.10
2015	Italy	All Ages	0.13	0.26	0.06
2019	Italy	Under 5	0.15	0.33	0.06
2019	Italy	5-14 years	0.07	0.17	0.03
2019	Italy	15-49 years	0.08	0.16	0.03
2019	Italy	50-69 years	0.17	0.35	0.07
2019	Italy	70+ years	0.24	0.50	0.10
2019	Italy	All Ages	0.13	0.27	0.06
2000	Portugal	Under 5	0.09	0.19	0.03
2000	Portugal	5-14 years	0.04	0.09	0.01
2000	Portugal	15-49 years	0.05	0.10	0.02
2000	Portugal	50-69 years	0.09	0.20	0.04
2000	Portugal	70+ years	0.14	0.29	0.06
2000	Portugal	All Ages	0.07	0.14	0.03
2005	Portugal	Under 5	0.08	0.18	0.03
2005	Portugal	5-14 years	0.04	0.09	0.01
2005	Portugal	15-49 years	0.05	0.10	0.02
2005	Portugal	50-69 years	0.09	0.19	0.04
2005	Portugal	70+ years	0.14	0.30	0.06
2005	Portugal	All Ages	0.07	0.14	0.03
2010	Portugal	Under 5	0.08	0.17	0.03
2010	Portugal	5-14 years	0.03	0.08	0.01
2010	Portugal	15-49 years	0.04	0.10	0.02
2010	Portugal	50-69 years	0.09	0.20	0.04
2010	Portugal	70+ years	0.14	0.30	0.06
2010	Portugal	All Ages	0.07	0.15	0.03
2015	Portugal	Under 5	0.08	0.17	0.03
2015	Portugal	5-14 years	0.03	0.08	0.01
2015	Portugal	15-49 years	0.04	0.09	0.02
2015	Portugal	50-69 years	0.09	0.20	0.04
2015	Portugal	70+ years	0.15	0.30	0.06
2015	Portugal	All Ages	0.07	0.15	0.03
2019	Portugal	Under 5	0.08	0.17	0.03
2019	Portugal	5-14 years	0.03	0.07	0.01
2019	Portugal	15-49 years	0.04	0.09	0.02
2019	Portugal	50-69 years	0.09	0.19	0.04
2019	Portugal	70+ years	0.15	0.31	0.06
2019	Portugal	All Ages	0.07	0.15	0.03
2000	Spain	Under 5	0.08	0.18	0.03
2000	Spain	5-14 years	0.04	0.08	0.01
2000	Spain	15-49 years	0.05	0.10	0.02

2000	Spain	50-69 years	0.11	0.22	0.05
2000	Spain	70+ years	0.15	0.30	0.06
2000	Spain	All Ages	0.07	0.14	0.03
2005	Spain	Under 5	0.07	0.16	0.03
2005	Spain	5-14 years	0.03	0.07	0.01
2005	Spain	15-49 years	0.04	0.08	0.02
2005	Spain	50-69 years	0.09	0.18	0.04
2005	Spain	70+ years	0.16	0.32	0.07
2005	Spain	All Ages	0.06	0.13	0.03
2010	Spain	Under 5	0.08	0.17	0.03
2010	Spain	5-14 years	0.03	0.08	0.01
2010	Spain	15-49 years	0.05	0.10	0.02
2010	Spain	50-69 years	0.11	0.24	0.05
2010	Spain	70+ years	0.19	0.39	0.08
2010	Spain	All Ages	0.08	0.16	0.04
2015	Spain	Under 5	0.07	0.16	0.03
2015	Spain	5-14 years	0.03	0.08	0.01
2015	Spain	15-49 years	0.05	0.10	0.02
2015	Spain	50-69 years	0.11	0.22	0.05
2015	Spain	70+ years	0.18	0.38	0.08
2015	Spain	All Ages	0.08	0.16	0.04
2019	Spain	Under 5	0.07	0.16	0.03
2019	Spain	5-14 years	0.03	0.07	0.01
2019	Spain	15-49 years	0.05	0.10	0.02
2019	Spain	50-69 years	0.11	0.22	0.05
2019	Spain	70+ years	0.19	0.39	0.08
2019	Spain	All Ages	0.08	0.17	0.04

Table S48- Rates and 95% uncertainty levels (UL) of **acute HCV disability-adjusted life years** (DALYs) per 100,000 population in Greece, Italy, Portugal and Spain from 2000 to 2019 by age group (Global Burden of Disease Study 2019).

Year	Country	Age	DALYs (95% UL)	95% UL (upper)	95% UL (lower)
2000	Greece	Under 5	0.693	1.250	0.236
2000	Greece	5-14 years	0.020	0.040	0.009
2000	Greece	15-49 years	0.118	0.281	0.061
2000	Greece	50-69 years	0.344	0.931	0.125
2000	Greece	70+ years	0.945	2.758	0.295
2000	Greece	All Ages	0.274	0.661	0.129
2005	Greece	Under 5	0.979	1.789	0.283
2005	Greece	5-14 years	0.026	0.053	0.011
2005	Greece	15-49 years	0.167	0.433	0.082
2005	Greece	50-69 years	0.470	1.332	0.163
2005	Greece	70+ years	1.511	4.642	0.446
2005	Greece	All Ages	0.427	1.107	0.194
2010	Greece	Under 5	1.851	3.437	0.399
2010	Greece	5-14 years	0.031	0.066	0.013
2010	Greece	15-49 years	0.329	0.805	0.166
2010	Greece	50-69 years	0.860	2.528	0.328
2010	Greece	70+ years	3.836	11.35	1.382
2010	Greece	All Ages	0.998	2.513	0.491
2015	Greece	Under 5	0.859	1.561	0.229
2015	Greece	5-14 years	0.025	0.051	0.012
2015	Greece	15-49 years	0.206	0.367	0.105
2015	Greece	50-69 years	0.477	1.154	0.258
2015	Greece	70+ years	1.620	3.381	0.910
2015	Greece	All Ages	0.504	1.011	0.323
2019	Greece	Under 5	0.735	1.433	0.187
2019	Greece	5-14 years	0.026	0.052	0.012
2019	Greece	15-49 years	0.207	0.378	0.108
2019	Greece	50-69 years	0.448	1.116	0.222
2019	Greece	70+ years	1.436	3.399	0.740
2019	Greece	All Ages	0.479	1.029	0.289
2000	Italy	Under 5	0.665	1.729	0.338
2000	Italy	5-14 years	0.169	0.317	0.078
2000	Italy	15-49 years	0.416	0.619	0.237
2000	Italy	50-69 years	1.287	1.838	0.640
2000	Italy	70+ years	1.904	2.682	0.721
2000	Italy	All Ages	0.805	1.104	0.426
2005	Italy	Under 5	0.569	1.779	0.271
2005	Italy	5-14 years	0.116	0.225	0.057
2005	Italy	15-49 years	0.345	0.601	0.208
2005	Italy	50-69 years	1.080	1.543	0.567
2005	Italy	70+ years	1.703	2.288	0.691
2005	Italy	All Ages	0.699	1.018	0.379
2010	Italy	Under 5	0.454	1.475	0.191

2010	Italy	5-14 years	0.100	0.212	0.046
2010	Italy	15-49 years	0.216	0.523	0.117
2010	Italy	50-69 years	0.563	1.238	0.338
2010	Italy	70+ years	0.836	1.741	0.536
2010	Italy	All Ages	0.393	0.869	0.243
2015	Italy	Under 5	0.368	1.032	0.174
2015	Italy	5-14 years	0.082	0.177	0.037
2015	Italy	15-49 years	0.167	0.409	0.084
2015	Italy	50-69 years	0.430	0.976	0.240
2015	Italy	70+ years	0.663	1.436	0.398
2015	Italy	All Ages	0.315	0.714	0.181
2019	Italy	Under 5	0.351	0.807	0.182
2019	Italy	5-14 years	0.085	0.181	0.039
2019	Italy	15-49 years	0.175	0.379	0.093
2019	Italy	50-69 years	0.447	0.894	0.265
2019	Italy	70+ years	0.715	1.341	0.452
2019	Italy	All Ages	0.341	0.684	0.210
2000	Portugal	Under 5	1.197	2.582	0.534
2000	Portugal	5-14 years	0.096	0.221	0.046
2000	Portugal	15-49 years	1.283	3.078	0.649
2000	Portugal	50-69 years	1.232	2.686	0.641
2000	Portugal	70+ years	1.023	1.911	0.604
2000	Portugal	All Ages	1.108	2.323	0.649
2005	Portugal	Under 5	0.520	0.795	0.253
2005	Portugal	5-14 years	0.058	0.111	0.031
2005	Portugal	15-49 years	0.600	0.865	0.186
2005	Portugal	50-69 years	0.722	1.037	0.309
2005	Portugal	70+ years	0.653	0.923	0.331
2005	Portugal	All Ages	0.573	0.762	0.233
2010	Portugal	Under 5	0.329	0.505	0.149
2010	Portugal	5-14 years	0.046	0.094	0.023
2010	Portugal	15-49 years	0.369	0.529	0.137
2010	Portugal	50-69 years	0.475	0.664	0.231
2010	Portugal	70+ years	0.455	0.672	0.241
2010	Portugal	All Ages	0.370	0.501	0.174
2015	Portugal	Under 5	0.250	0.397	0.125
2015	Portugal	5-14 years	0.044	0.090	0.021
2015	Portugal	15-49 years	0.215	0.312	0.090
2015	Portugal	50-69 years	0.308	0.468	0.170
2015	Portugal	70+ years	0.340	0.535	0.193
2015	Portugal	All Ages	0.242	0.351	0.129
2019	Portugal	Under 5	0.222	0.372	0.113
2019	Portugal	5-14 years	0.043	0.087	0.020
2019	Portugal	15-49 years	0.224	0.346	0.090
2019	Portugal	50-69 years	0.298	0.457	0.165
2019	Portugal	70+ years	0.338	0.531	0.187
2019	Portugal	All Ages	0.246	0.352	0.129
2000	Spain	Under 5	0.840	2.581	0.350
2000	Spain	5-14 years	0.077	0.129	0.047
2000	Spain	15-49 years	0.569	0.728	0.328

2000	Spain	50-69 years	1.209	1.520	0.701
2000	Spain	70+ years	1.213	1.520	0.802
2000	Spain	All Ages	0.741	0.905	0.479
2005	Spain	Under 5	0.672	1.851	0.312
2005	Spain	5-14 years	0.055	0.094	0.033
2005	Spain	15-49 years	0.484	0.626	0.253
2005	Spain	50-69 years	1.019	1.283	0.552
2005	Spain	70+ years	1.055	1.355	0.666
2005	Spain	All Ages	0.634	0.775	0.375
2010	Spain	Under 5	0.532	1.233	0.279
2010	Spain	5-14 years	0.056	0.105	0.031
2010	Spain	15-49 years	0.364	0.476	0.212
2010	Spain	50-69 years	0.747	0.964	0.460
2010	Spain	70+ years	0.786	1.060	0.543
2010	Spain	All Ages	0.480	0.614	0.319
2015	Spain	Under 5	0.383	0.783	0.207
2015	Spain	5-14 years	0.049	0.096	0.026
2015	Spain	15-49 years	0.271	0.388	0.176
2015	Spain	50-69 years	0.563	0.795	0.379
2015	Spain	70+ years	0.608	0.891	0.419
2015	Spain	All Ages	0.369	0.517	0.265
2019	Spain	Under 5	0.362	0.704	0.189
2019	Spain	5-14 years	0.050	0.094	0.026
2019	Spain	15-49 years	0.289	0.414	0.182
2019	Spain	50-69 years	0.547	0.761	0.351
2019	Spain	70+ years	0.617	0.884	0.425
2019	Spain	All Ages	0.382	0.528	0.262

Table S49- Rates and 95% uncertainty levels (UL) of **prevalence of cirrhosis and other chronic liver diseases due to hepatitis B** per 100,000 population in Greece, Italy, Portugal and Spain from 2000 to 2019 by age group (Global Burden of Disease Study 2019).

Year	Country	Age	Prevalence (95% UL)	95% UL (upper)	95% UL (lower)
2000	Greece	Under 5	110.9	143.2	85.29
2000	Greece	5-14 years	1329.3	1636.9	1092.7
2000	Greece	15-49 years	2110.9	2342.3	1904.2
2000	Greece	50-69 years	2500.5	2793.6	2231.0
2000	Greece	70+ years	2503.6	2864.0	2179.7
2000	Greece	All Ages	2063.5	2269.2	1879.9
2005	Greece	Under 5	74.32	98.87	52.96
2005	Greece	5-14 years	824.5	1019.	667.3
2005	Greece	15-49 years	2053.7	2291.5	1840.7
2005	Greece	50-69 years	2422.2	2686.4	2163.5
2005	Greece	70+ years	2431.8	2764.0	2086.1
2005	Greece	All Ages	1967.9	2160.3	1775.7
2010	Greece	Under 5	57.8	77.3	42.7
2010	Greece	5-14 years	176.9	220.2	145.0
2010	Greece	15-49 years	2016.1	2245.5	1808.7
2010	Greece	50-69 years	2379.9	2650.1	2129.6
2010	Greece	70+ years	2372.0	2752.6	2038.5
2010	Greece	All Ages	1874.8	2061.8	1705.9
2015	Greece	Under 5	52.7	69.3	38.2
2015	Greece	5-14 years	129.0	160.9	106.3
2015	Greece	15-49 years	1877.9	2083.9	1680.6
2015	Greece	50-69 years	2357.3	2636.3	2082.1
2015	Greece	70+ years	2334.7	2726.2	2010.9
2015	Greece	All Ages	1812.7	1997.3	1637.0
2019	Greece	Under 5	50.5	65.8	37.3
2019	Greece	5-14 years	115.8	141.6	94.18
2019	Greece	15-49 years	1836.6	2047.8	1664.2
2019	Greece	50-69 years	2332.5	2587.3	2110.0
2019	Greece	70+ years	2315.6	2649.9	2007.0
2019	Greece	All Ages	1798.9	1971.6	1645.3
2000	Italy	Under 5	34.9	44.3	26.6
2000	Italy	5-14 years	250.04	305.99	205.89
2000	Italy	15-49 years	1162.0	1302.9	1031.5
2000	Italy	50-69 years	1223.5	1354.8	1092.8
2000	Italy	70+ years	1045.5	1183.9	918.30
2000	Italy	All Ages	1021.4	1125.4	916.42
2005	Italy	Under 5	27.49	35.58	21.06
2005	Italy	5-14 years	106.49	131.86	87.153
2005	Italy	15-49 years	1066.5	1192.8	940.71
2005	Italy	50-69 years	1180.4	1318.6	1052.6
2005	Italy	70+ years	1022.4	1166.7	894.66
2005	Italy	All Ages	948.3	1048.	848.0
2010	Italy	Under 5	27.2	34.8	20.6
2010	Italy	5-14 years	60.8	75.5	48.7
2010	Italy	15-49 years	906.9	1016.	794.0

2010	Italy	50-69 years	1118.2	1253.1	988.78
2010	Italy	70+ years	988.4	1134.	860.1
2010	Italy	All Ages	849.8	945.4	752.7
2015	Italy	Under 5	26.9	34.8	20.5
2015	Italy	5-14 years	51.5	64.0	41.5
2015	Italy	15-49 years	732.5	824.9	644.1
2015	Italy	50-69 years	1075.2	1214.7	946.08
2015	Italy	70+ years	967.2	1115.	836.3
2015	Italy	All Ages	764.2	852.1	671.9
2019	Italy	Under 5	26.6	33.1	20.6
2019	Italy	5-14 years	52.1	63.6	42.1
2019	Italy	15-49 years	581.4	658.2	507.3
2019	Italy	50-69 years	1076.7	1224.5	937.08
2019	Italy	70+ years	964.2	1127.	827.3
2019	Italy	All Ages	713.2	803.6	623.0
2000	Portugal	Under 5	184.4	265.1	119.2
2000	Portugal	5-14 years	996.9	1331.	704.0
2000	Portugal	15-49 years	1328.7	1595.9	1077.3
2000	Portugal	50-69 years	1219.3	1451.5	993.53
2000	Portugal	70+ years	1079.6	1323.5	857.14
2000	Portugal	All Ages	1181.4	1420.3	968.70
2005	Portugal	Under 5	56.1	76.7	38.9
2005	Portugal	5-14 years	602.8	789.1	454.4
2005	Portugal	15-49 years	1277.1	1511.6	1040.7
2005	Portugal	50-69 years	1192.6	1396.0	974.87
2005	Portugal	70+ years	1057.8	1271.7	831.83
2005	Portugal	All Ages	1097.7	1280.4	899.04
2010	Portugal	Under 5	46.5	65.4	31.3
2010	Portugal	5-14 years	235.4	312.5	172.0
2010	Portugal	15-49 years	1261.0	1499.5	1040.9
2010	Portugal	50-69 years	1185.5	1410.3	986.02
2010	Portugal	70+ years	1044.3	1262.9	841.75
2010	Portugal	All Ages	1049.7	1238.1	875.86
2015	Portugal	Under 5	38.7	53.3	25.9
2015	Portugal	5-14 years	99.97	130.9	72.61
2015	Portugal	15-49 years	1141.5	1350.4	937.29
2015	Portugal	50-69 years	1164.7	1375.5	928.99
2015	Portugal	70+ years	1019.7	1229.6	792.15
2015	Portugal	All Ages	980.8	1150.	795.2
2019	Portugal	Under 5	36.3	49.5	24.2
2019	Portugal	5-14 years	82.8	108.	59.3
2019	Portugal	15-49 years	985.3	1188.	809.8
2019	Portugal	50-69 years	1131.4	1372.2	905.20
2019	Portugal	70+ years	992.6	1242.	769.2
2019	Portugal	All Ages	905.8	1090.	735.6
2000	Spain	Under 5	98.6	129.	68.5
2000	Spain	5-14 years	507.3	627.5	404.6
2000	Spain	15-49 years	1056.0	1180.9	931.48
2000	Spain	50-69 years	1111.7	1248.7	977.23
2000	Spain	70+ years	1027.4	1194.1	876.20
2000	Spain	All Ages	963.9	1069.	854.7
2005	Spain	Under 5	35.6	48.0	26.1
2005	Spain	5-14 years	193.0	241.6	155.4

2005	Spain	15-49 years	1037.0	1162.4	917.22
2005	Spain	50-69 years	1088.0	1240.1	970.32
2005	Spain	70+ years	999.6	1193.	863.1
2005	Spain	All Ages	913.2	1017.	817.7
2010	Spain	Under 5	32.3	42.4	23.1
2010	Spain	5-14 years	132.9	164.8	107.2
2010	Spain	15-49 years	960.7	1082.	856.0
2010	Spain	50-69 years	1058.4	1183.5	942.06
2010	Spain	70+ years	969.0	1124.	824.1
2010	Spain	All Ages	855.5	952.6	763.6
2015	Spain	Under 5	31.7	43.7	23.2
2015	Spain	5-14 years	66.5	82.5	54.1
2015	Spain	15-49 years	880.5	980.3	781.7
2015	Spain	50-69 years	1054.2	1189.1	927.19
2015	Spain	70+ years	967.9	1140.	826.8
2015	Spain	All Ages	809.7	900.5	724.1
2019	Spain	Under 5	29.2	39.4	21.4
2019	Spain	5-14 years	61.5	76.9	50.1
2019	Spain	15-49 years	759.6	847.3	669.6
2019	Spain	50-69 years	1028.3	1152.1	908.51
2019	Spain	70+ years	939.8	1106.	798.3
2019	Spain	All Ages	751.1	830.5	669.0

Table S50- Rates and 95% uncertainty levels (UL) of **incidence of cirrhosis and other chronic liver diseases due to hepatitis B** per 100,000 population in Greece, Italy, Portugal and Spain from 2000 to 2019 by age group (Global Burden of Disease Study 2019).

Year	Country	Age	incidence (95% UL)	95% UL (upper)	95% UL (lower)
2000	Greece	Under 5	0.00	0.02	0.00
2000	Greece	5-14 years	0.03	0.08	0.00
2000	Greece	15-49 years	5.40	7.99	3.25
2000	Greece	50-69 years	4.63	8.44	2.17
2000	Greece	70+ years	0.08	0.32	0.0
2000	Greece	All Ages	3.82	5.52	2.56
2005	Greece	Under 5	0.01	0.02	0.00
2005	Greece	5-14 years	0.03	0.09	0.00
2005	Greece	15-49 years	5.37	7.90	3.23
2005	Greece	50-69 years	3.56	6.95	1.47
2005	Greece	70+ years	0.07	0.27	0.0
2005	Greece	All Ages	3.51	4.99	2.30
2010	Greece	Under 5	0.00	0.01	0.00
2010	Greece	5-14 years	0.01	0.04	0.00
2010	Greece	15-49 years	2.99	4.69	1.76
2010	Greece	50-69 years	1.44	3.04	0.55
2010	Greece	70+ years	0.02	0.09	0.0
2010	Greece	All Ages	1.78	2.68	1.11
2015	Greece	Under 5	0.00	0.01	0.00
2015	Greece	5-14 years	0.01	0.04	0.00
2015	Greece	15-49 years	2.99	4.60	1.69
2015	Greece	50-69 years	1.19	2.57	0.45
2015	Greece	70+ years	0.01	0.07	0.0
2015	Greece	All Ages	1.65	2.44	1.00
2019	Greece	Under 5	0.00	0.01	0.00
2019	Greece	5-14 years	0.01	0.05	0.00
2019	Greece	15-49 years	2.97	4.58	1.70
2019	Greece	50-69 years	1.01	2.02	0.37
2019	Greece	70+ years	0.02	0.11	0.0
2019	Greece	All Ages	1.55	2.36	0.97
2000	Italy	Under 5	0.01	0.02	0.00
2000	Italy	5-14 years	0.03	0.06	0.01
2000	Italy	15-49 years	10.0	12.9	7.51
2000	Italy	50-69 years	5.95	9.42	2.97
2000	Italy	70+ years	0.01	0.13	0.0
2000	Italy	All Ages	6.31	7.85	4.91
2005	Italy	Under 5	0.01	0.02	0.00
2005	Italy	5-14 years	0.02	0.05	0.01
2005	Italy	15-49 years	9.39	12.0	7.04
2005	Italy	50-69 years	5.30	8.19	2.81
2005	Italy	70+ years	0.01	0.09	0.0
2005	Italy	All Ages	5.78	7.17	4.54
2010	Italy	Under 5	0.01	0.02	0.00
2010	Italy	5-14 years	0.02	0.04	0.00
2010	Italy	15-49 years	7.77	10.0	5.74
2010	Italy	50-69 years	4.48	6.97	2.49
2010	Italy	70+ years	0.00	0.06	0.0

2010	Italy	All Ages	4.72	5.91	3.71
2015	Italy	Under 5	0.01	0.02	0.00
2015	Italy	5-14 years	0.02	0.04	0.00
2015	Italy	15-49 years	7.34	9.58	5.31
2015	Italy	50-69 years	4.26	6.73	2.36
2015	Italy	70+ years	0.00	0.06	0.0
2015	Italy	All Ages	4.36	5.54	3.39
2019	Italy	Under 5	0.01	0.02	0.00
2019	Italy	5-14 years	0.02	0.05	0.01
2019	Italy	15-49 years	6.90	9.23	4.85
2019	Italy	50-69 years	4.10	6.60	2.05
2019	Italy	70+ years	0.01	0.13	0.0
2019	Italy	All Ages	4.04	5.32	2.99
2000	Portugal	Under 5	0.00	0.01	0.00
2000	Portugal	5-14 years	0.02	0.05	0.00
2000	Portugal	15-49 years	7.77	11.9	4.70
2000	Portugal	50-69 years	2.30	4.80	0.66
2000	Portugal	70+ years	0.00	0.02	0.0
2000	Portugal	All Ages	4.47	6.65	2.80
2005	Portugal	Under 5	0.00	0.01	0.00
2005	Portugal	5-14 years	0.01	0.04	0.00
2005	Portugal	15-49 years	6.50	10.2	3.79
2005	Portugal	50-69 years	1.47	3.24	0.35
2005	Portugal	70+ years	0.00	0.03	0.0
2005	Portugal	All Ages	3.56	5.39	2.19
2010	Portugal	Under 5	0.00	0.00	0.00
2010	Portugal	5-14 years	0.01	0.02	0.00
2010	Portugal	15-49 years	3.90	6.22	2.22
2010	Portugal	50-69 years	0.80	1.90	0.19
2010	Portugal	70+ years	0.01	0.05	0.0
2010	Portugal	All Ages	2.05	3.16	1.22
2015	Portugal	Under 5	0.00	0.00	0.00
2015	Portugal	5-14 years	0.00	0.02	0.00
2015	Portugal	15-49 years	3.11	4.95	1.75
2015	Portugal	50-69 years	0.66	1.48	0.16
2015	Portugal	70+ years	0.01	0.05	0.0
2015	Portugal	All Ages	1.59	2.49	0.95
2019	Portugal	Under 5	0.00	0.00	0.00
2019	Portugal	5-14 years	0.00	0.02	0.00
2019	Portugal	15-49 years	2.72	4.35	1.52
2019	Portugal	50-69 years	0.57	1.37	0.11
2019	Portugal	70+ years	0.02	0.10	0.0
2019	Portugal	All Ages	1.36	2.13	0.79
2000	Spain	Under 5	0.01	0.02	0.00
2000	Spain	5-14 years	0.04	0.09	0.01
2000	Spain	15-49 years	7.64	10.7	5.13
2000	Spain	50-69 years	4.17	7.35	1.86
2000	Spain	70+ years	0.08	0.29	0.0
2000	Spain	All Ages	4.89	6.59	3.57
2005	Spain	Under 5	0.00	0.01	0.00
2005	Spain	5-14 years	0.02	0.05	0.00
2005	Spain	15-49 years	4.48	6.33	3.06
2005	Spain	50-69 years	1.81	3.24	0.86

2005	Spain	70+ years	0.04	0.15	0.0
2005	Spain	All Ages	2.74	3.75	1.95
2010	Spain	Under 5	0.00	0.01	0.00
2010	Spain	5-14 years	0.02	0.05	0.00
2010	Spain	15-49 years	4.25	5.96	2.86
2010	Spain	50-69 years	1.75	3.12	0.76
2010	Spain	70+ years	0.05	0.20	0.0
2010	Spain	All Ages	2.56	3.46	1.86
2015	Spain	Under 5	0.00	0.01	0.00
2015	Spain	5-14 years	0.01	0.04	0.00
2015	Spain	15-49 years	3.68	5.32	2.42
2015	Spain	50-69 years	1.43	2.53	0.62
2015	Spain	70+ years	0.07	0.27	0.00
2015	Spain	All Ages	2.10	2.92	1.49
2019	Spain	Under 5	0.00	0.01	0.00
2019	Spain	5-14 years	0.02	0.05	0.00
2019	Spain	15-49 years	3.40	4.88	2.29
2019	Spain	50-69 years	1.27	2.35	0.59
2019	Spain	70+ years	0.09	0.32	0.0
2019	Spain	All Ages	1.87	2.59	1.34

Table S51- Rates and 95% uncertainty levels (UL) of **deaths due to cirrhosis and other chronic liver diseases due to hepatitis B** per 100,000 population in Greece, Italy, Portugal and Spain from 2000 to 2019 by age group (Global Burden of Disease Study 2019).

Year	Country	Age	Deaths (95% UL)	95% UL (upper)	95% UL (lower)
2000	Greece	Under 5	0.000	0.000	0.000
2000	Greece	5-14 years	0.000	0.001	0.000
2000	Greece	15-49 years	0.351	0.541	0.220
2000	Greece	50-69 years	2.274	3.563	1.362
2000	Greece	70+ years	7.714	11.54	4.609
2000	Greece	All Ages	1.522	2.119	1.053
2005	Greece	Under 5	0.000	0.000	7.939
2005	Greece	5-14 years	0.000	0.001	0.000
2005	Greece	15-49 years	0.398	0.609	0.243
2005	Greece	50-69 years	2.171	3.425	1.297
2005	Greece	70+ years	5.954	9.082	3.478
2005	Greece	All Ages	1.433	1.979	1.006
2010	Greece	Under 5	0.000	0.000	2.858
2010	Greece	5-14 years	0.000	0.000	5.279
2010	Greece	15-49 years	0.249	0.399	0.144
2010	Greece	50-69 years	1.212	1.946	0.683
2010	Greece	70+ years	2.768	4.375	1.538
2010	Greece	All Ages	0.792	1.115	0.542
2015	Greece	Under 5	0.000	0.000	2.855
2015	Greece	5-14 years	0.000	0.000	4.117
2015	Greece	15-49 years	0.240	0.388	0.137
2015	Greece	50-69 years	1.234	2.001	0.692
2015	Greece	70+ years	2.596	4.040	1.488
2015	Greece	All Ages	0.819	1.139	0.566
2019	Greece	Under 5	0.000	0.000	2.346
2019	Greece	5-14 years	0.000	0.000	4.033
2019	Greece	15-49 years	0.243	0.403	0.140
2019	Greece	50-69 years	1.171	1.906	0.669
2019	Greece	70+ years	2.812	4.380	1.587
2019	Greece	All Ages	0.877	1.225	0.612
2000	Italy	Under 5	0.000	0.000	0.000
2000	Italy	5-14 years	0.000	0.001	0.000
2000	Italy	15-49 years	0.733	0.838	0.646
2000	Italy	50-69 years	4.375	5.066	3.763
2000	Italy	70+ years	10.29	12.29	8.422
2000	Italy	All Ages	2.733	3.077	2.417
2005	Italy	Under 5	0.000	0.000	0.000
2005	Italy	5-14 years	0.000	0.000	0.000
2005	Italy	15-49 years	0.632	0.745	0.539
2005	Italy	50-69 years	3.151	3.726	2.657
2005	Italy	70+ years	8.247	10.01	6.663
2005	Italy	All Ages	2.207	2.517	1.919
2010	Italy	Under 5	0.000	0.000	0.000
2010	Italy	5-14 years	0.000	0.000	0.000
2010	Italy	15-49 years	0.486	0.589	0.401
2010	Italy	50-69 years	2.265	2.731	1.872
2010	Italy	70+ years	6.630	8.106	5.276

2010	Italy	All Ages	1.769	2.044	1.525
2015	Italy	Under 5	0.000	0.000	7.636
2015	Italy	5-14 years	0.000	0.000	0.000
2015	Italy	15-49 years	0.416	0.504	0.342
2015	Italy	50-69 years	2.045	2.450	1.691
2015	Italy	70+ years	5.942	7.313	4.682
2015	Italy	All Ages	1.661	1.921	1.421
2019	Italy	Under 5	0.000	0.000	6.016
2019	Italy	5-14 years	0.000	0.000	0.000
2019	Italy	15-49 years	0.401	0.486	0.323
2019	Italy	50-69 years	1.943	2.331	1.600
2019	Italy	70+ years	5.817	7.211	4.602
2019	Italy	All Ages	1.699	1.985	1.443
2000	Portugal	Under 5	0.000	0.001	0.000
2000	Portugal	5-14 years	0.001	0.003	0.000
2000	Portugal	15-49 years	1.573	2.437	0.956
2000	Portugal	50-69 years	6.001	9.547	3.658
2000	Portugal	70+ years	8.772	13.43	5.099
2000	Portugal	All Ages	3.060	4.190	2.151
2005	Portugal	Under 5	0.000	0.000	8.145
2005	Portugal	5-14 years	0.001	0.002	0.000
2005	Portugal	15-49 years	1.292	2.025	0.773
2005	Portugal	50-69 years	4.629	7.352	2.771
2005	Portugal	70+ years	6.399	9.993	3.692
2005	Portugal	All Ages	2.462	3.373	1.739
2010	Portugal	Under 5	9.515	0.000	2.299
2010	Portugal	5-14 years	0.000	0.000	0.000
2010	Portugal	15-49 years	0.769	1.234	0.440
2010	Portugal	50-69 years	2.822	4.534	1.627
2010	Portugal	70+ years	3.836	5.994	2.206
2010	Portugal	All Ages	1.556	2.147	1.074
2015	Portugal	Under 5	7.076	0.000	1.614
2015	Portugal	5-14 years	0.000	0.000	4.931
2015	Portugal	15-49 years	0.471	0.782	0.270
2015	Portugal	50-69 years	2.055	3.337	1.184
2015	Portugal	70+ years	2.875	4.560	1.635
2015	Portugal	All Ages	1.166	1.641	0.783
2019	Portugal	Under 5	5.305	0.000	1.185
2019	Portugal	5-14 years	0.000	0.000	5.027
2019	Portugal	15-49 years	0.512	0.842	0.294
2019	Portugal	50-69 years	1.983	3.205	1.119
2019	Portugal	70+ years	2.924	4.567	1.641
2019	Portugal	All Ages	1.226	1.722	0.848
2000	Spain	Under 5	0.000	0.000	0.000
2000	Spain	5-14 years	0.001	0.002	0.000
2000	Spain	15-49 years	0.851	1.164	0.601
2000	Spain	50-69 years	4.168	5.809	2.967
2000	Spain	70+ years	10.36	15.22	6.514
2000	Spain	All Ages	2.531	3.274	1.962
2005	Spain	Under 5	0.000	0.000	6.663
2005	Spain	5-14 years	0.000	0.001	0.000
2005	Spain	15-49 years	0.497	0.700	0.341
2005	Spain	50-69 years	2.291	3.266	1.587

2005	Spain	70+ years	5.652	8.454	3.486
2005	Spain	All Ages	1.432	1.892	1.091
2010	Spain	Under 5	0.000	0.000	4.997
2010	Spain	5-14 years	0.000	0.000	0.000
2010	Spain	15-49 years	0.388	0.549	0.266
2010	Spain	50-69 years	1.917	2.712	1.328
2010	Spain	70+ years	4.647	6.949	2.852
2010	Spain	All Ages	1.193	1.549	0.897
2015	Spain	Under 5	0.000	0.000	3.022
2015	Spain	5-14 years	0.000	0.000	8.378
2015	Spain	15-49 years	0.262	0.372	0.176
2015	Spain	50-69 years	1.556	2.196	1.091
2015	Spain	70+ years	3.878	5.878	2.360
2015	Spain	All Ages	1.016	1.360	0.757
2019	Spain	Under 5	9.685	0.000	2.567
2019	Spain	5-14 years	0.000	0.000	7.941
2019	Spain	15-49 years	0.279	0.405	0.189
2019	Spain	50-69 years	1.421	2.037	0.997
2019	Spain	70+ years	3.801	5.874	2.260
2019	Spain	All Ages	1.042	1.399	0.767

Table S52- Rates and 95% uncertainty levels (UL) of **years of life lost** (YLLs) **due to cirrhosis and other chronic liver diseases due to hepatitis B** per 100,000 population in Greece, Italy, Portugal and Spain from 2000 to 2019 by age group (Global Burden of Disease Study 2019).

Year	Country	Age	YLLs (95% UL)	95% UL (upper)	95% UL (lower)
2000	Greece	Under 5	0.02	0.05	0.00
2000	Greece	5-14 years	0.04	0.08	0.01
2000	Greece	15-49 years	16.3	25.1	10.2
2000	Greece	50-69 years	65.9	104.1	39.8
2000	Greece	70+ years	102.	156.2	59.5
2000	Greece	All Ages	34.4	47.2	24.2
2005	Greece	Under 5	0.02	0.04	0.00
2005	Greece	5-14 years	0.04	0.08	0.01
2005	Greece	15-49 years	18.4	28.1	11.3
2005	Greece	50-69 years	64.5	101.7	38.1
2005	Greece	70+ years	80.5	122.8	45.9
2005	Greece	All Ages	33.9	46.2	23.9
2010	Greece	Under 5	0.01	0.02	0.00
2010	Greece	5-14 years	0.01	0.03	0.00
2010	Greece	15-49 years	11.4	18.2	6.74
2010	Greece	50-69 years	36.8	59.6	20.6
2010	Greece	70+ years	36.9	57.8	20.1
2010	Greece	All Ages	19.2	26.8	13.3
2015	Greece	Under 5	0.01	0.02	0.00
2015	Greece	5-14 years	0.01	0.02	0.00
2015	Greece	15-49 years	10.9	17.4	6.26
2015	Greece	50-69 years	37.2	59.7	21.0
2015	Greece	70+ years	32.8	50.4	18.8
2015	Greece	All Ages	19.3	26.9	13.0
2019	Greece	Under 5	0.00	0.02	0.00
2019	Greece	5-14 years	0.01	0.03	0.00
2019	Greece	15-49 years	11.0	18.1	6.39
2019	Greece	50-69 years	35.3	57.2	20.4
2019	Greece	70+ years	34.6	54.4	19.7
2019	Greece	All Ages	19.7	27.3	13.4
2000	Italy	Under 5	0.03	0.06	0.01
2000	Italy	5-14 years	0.06	0.08	0.03
2000	Italy	15-49 years	34.6	39.4	30.5
2000	Italy	50-69 years	125.8	144.8	108.1
2000	Italy	70+ years	147.7	175.4	121.1
2000	Italy	All Ages	66.1	72.9	59.5
2005	Italy	Under 5	0.02	0.03	0.01
2005	Italy	5-14 years	0.03	0.05	0.02
2005	Italy	15-49 years	29.3	34.4	25.0
2005	Italy	50-69 years	91.2	107.7	76.8
2005	Italy	70+ years	115.7	140.8	93.24
2005	Italy	All Ages	52.0	58.4	46.2
2010	Italy	Under 5	0.01	0.03	0.00
2010	Italy	5-14 years	0.03	0.04	0.02
2010	Italy	15-49 years	22.2	26.7	18.3
2010	Italy	50-69 years	66.5	79.9	54.7
2010	Italy	70+ years	88.4	108.7	70.3

2010	Italy	All Ages	39.7	44.8	34.5
2015	Italy	Under 5	0.01	0.02	0.00
2015	Italy	5-14 years	0.02	0.03	0.01
2015	Italy	15-49 years	18.8	22.6	15.5
2015	Italy	50-69 years	60.6	72.9	49.9
2015	Italy	70+ years	76.1	93.1	60.5
2015	Italy	All Ages	36.2	41.5	31.7
2019	Italy	Under 5	0.00	0.01	0.00
2019	Italy	5-14 years	0.02	0.03	0.01
2019	Italy	15-49 years	18.1	21.9	14.6
2019	Italy	50-69 years	57.8	69.7	47.3
2019	Italy	70+ years	74.0	91.7	58.7
2019	Italy	All Ages	36.3	41.6	31.5
2000	Portugal	Under 5	0.04	0.10	0.01
2000	Portugal	5-14 years	0.13	0.27	0.04
2000	Portugal	15-49 years	74.2	113.8	45.2
2000	Portugal	50-69 years	180.0	282.6	107.5
2000	Portugal	70+ years	127.5	199.2	70.25
2000	Portugal	All Ages	91.0	123.6	63.9
2005	Portugal	Under 5	0.02	0.05	0.00
2005	Portugal	5-14 years	0.08	0.16	0.02
2005	Portugal	15-49 years	59.9	93.9	35.7
2005	Portugal	50-69 years	140.9	224.1	83.53
2005	Portugal	70+ years	92.0	144.6	51.3
2005	Portugal	All Ages	72.8	101.8	50.4
2010	Portugal	Under 5	0.00	0.01	0.00
2010	Portugal	5-14 years	0.03	0.06	0.00
2010	Portugal	15-49 years	35.3	56.8	20.3
2010	Portugal	50-69 years	87.0	140.1	49.4
2010	Portugal	70+ years	53.4	85.8	29.8
2010	Portugal	All Ages	44.8	63.5	29.8
2015	Portugal	Under 5	0.00	0.01	0.00
2015	Portugal	5-14 years	0.01	0.03	0.00
2015	Portugal	15-49 years	21.3	35.0	12.2
2015	Portugal	50-69 years	63.1	101.7	35.5
2015	Portugal	70+ years	38.4	61.0	21.5
2015	Portugal	All Ages	31.6	45.2	21.0
2019	Portugal	Under 5	0.00	0.01	0.00
2019	Portugal	5-14 years	0.01	0.03	0.00
2019	Portugal	15-49 years	23.2	38.1	13.3
2019	Portugal	50-69 years	60.6	96.9	33.6
2019	Portugal	70+ years	38.6	62.1	21.3
2019	Portugal	All Ages	32.7	46.3	22.0
2000	Spain	Under 5	0.04	0.08	0.01
2000	Spain	5-14 years	0.09	0.17	0.03
2000	Spain	15-49 years	40.2	55.0	28.6
2000	Spain	50-69 years	122.0	171.0	86.77
2000	Spain	70+ years	141.3	213.1	88.56
2000	Spain	All Ages	63.3	79.9	49.9
2005	Spain	Under 5	0.02	0.04	0.00
2005	Spain	5-14 years	0.04	0.08	0.01
2005	Spain	15-49 years	23.0	32.4	15.8
2005	Spain	50-69 years	68.8	97.7	48.1

2005	Spain	70+ years	75.7	116.2	45.7
2005	Spain	All Ages	35.6	45.6	27.1
2010	Spain	Under 5	0.01	0.03	0.00
2010	Spain	5-14 years	0.03	0.06	0.01
2010	Spain	15-49 years	17.6	24.6	12.1
2010	Spain	50-69 years	58.2	83.6	40.6
2010	Spain	70+ years	59.3	88.7	36.6
2010	Spain	All Ages	29.0	37.6	22.3
2015	Spain	Under 5	0.00	0.02	0.00
2015	Spain	5-14 years	0.02	0.04	0.00
2015	Spain	15-49 years	11.7	16.7	7.94
2015	Spain	50-69 years	47.4	67.6	33.2
2015	Spain	70+ years	47.4	72.4	28.6
2015	Spain	All Ages	23.3	30.7	17.7
2019	Spain	Under 5	0.00	0.01	0.00
2019	Spain	5-14 years	0.02	0.04	0.00
2019	Spain	15-49 years	12.5	18.1	8.58
2019	Spain	50-69 years	43.2	61.9	30.0
2019	Spain	70+ years	45.7	70.1	27.6
2019	Spain	All Ages	23.4	30.9	17.7

Table S53- Rates and 95% uncertainty levels (UL) of **years lived with disability** (YLDs) **due to cirrhosis and other chronic liver diseases due to hepatitis B** per 100,000 population in Greece, Italy, Portugal and Spain from 2000 to 2019 by age group (Global Burden of Disease Study 2019).

Year	Country	Age	YLDs (95% UL)	95% UL (upper)	95% UL (lower)
2000	Greece	Under 5	0.005	0.013	0.001
2000	Greece	5-14 years	0.027	0.062	0.008
2000	Greece	15-49 years	0.494	0.831	0.272
2000	Greece	50-69 years	1.450	2.759	0.627
2000	Greece	70+ years	1.941	3.676	0.895
2000	Greece	All Ages	0.793	1.260	0.463
2005	Greece	Under 5	0.005	0.013	0.001
2005	Greece	5-14 years	0.028	0.065	0.008
2005	Greece	15-49 years	0.515	0.933	0.256
2005	Greece	50-69 years	1.385	2.535	0.592
2005	Greece	70+ years	1.687	3.188	0.782
2005	Greece	All Ages	0.785	1.228	0.458
2010	Greece	Under 5	0.003	0.007	0.000
2010	Greece	5-14 years	0.014	0.035	0.003
2010	Greece	15-49 years	0.295	0.517	0.159
2010	Greece	50-69 years	0.792	1.455	0.408
2010	Greece	70+ years	0.843	1.573	0.422
2010	Greece	All Ages	0.446	0.702	0.269
2015	Greece	Under 5	0.003	0.007	0.000
2015	Greece	5-14 years	0.014	0.034	0.003
2015	Greece	15-49 years	0.294	0.516	0.157
2015	Greece	50-69 years	0.757	1.377	0.390
2015	Greece	70+ years	0.733	1.367	0.365
2015	Greece	All Ages	0.436	0.688	0.263
2019	Greece	Under 5	0.003	0.007	0.000
2019	Greece	5-14 years	0.015	0.037	0.003
2019	Greece	15-49 years	0.308	0.535	0.159
2019	Greece	50-69 years	0.751	1.395	0.387
2019	Greece	70+ years	0.693	1.279	0.345
2019	Greece	All Ages	0.445	0.697	0.267
2000	Italy	Under 5	0.005	0.009	0.002
2000	Italy	5-14 years	0.026	0.047	0.013
2000	Italy	15-49 years	0.752	1.145	0.470
2000	Italy	50-69 years	2.251	3.450	1.379
2000	Italy	70+ years	2.749	4.285	1.680
2000	Italy	All Ages	1.264	1.823	0.856
2005	Italy	Under 5	0.004	0.007	0.002
2005	Italy	5-14 years	0.019	0.034	0.009
2005	Italy	15-49 years	0.689	1.070	0.430
2005	Italy	50-69 years	1.964	3.005	1.188
2005	Italy	70+ years	2.571	4.046	1.597
2005	Italy	All Ages	1.161	1.689	0.792
2010	Italy	Under 5	0.004	0.007	0.001
2010	Italy	5-14 years	0.018	0.032	0.009
2010	Italy	15-49 years	0.548	0.828	0.348

2010	Italy	50-69 years	1.652	2.497	0.999
2010	Italy	70+ years	2.237	3.492	1.414
2010	Italy	All Ages	0.993	1.461	0.677
2015	Italy	Under 5	0.004	0.007	0.001
2015	Italy	5-14 years	0.017	0.032	0.009
2015	Italy	15-49 years	0.480	0.750	0.302
2015	Italy	50-69 years	1.463	2.234	0.907
2015	Italy	70+ years	1.969	3.091	1.239
2015	Italy	All Ages	0.909	1.319	0.615
2019	Italy	Under 5	0.004	0.008	0.002
2019	Italy	5-14 years	0.020	0.038	0.010
2019	Italy	15-49 years	0.454	0.711	0.275
2019	Italy	50-69 years	1.394	2.184	0.842
2019	Italy	70+ years	1.771	2.812	1.088
2019	Italy	All Ages	0.881	1.287	0.589
2000	Portugal	Under 5	0.002	0.006	0.000
2000	Portugal	5-14 years	0.012	0.030	0.003
2000	Portugal	15-49 years	0.992	1.863	0.427
2000	Portugal	50-69 years	2.533	4.805	1.151
2000	Portugal	70+ years	1.691	3.245	0.753
2000	Portugal	All Ages	1.244	2.074	0.687
2005	Portugal	Under 5	0.002	0.005	0.000
2005	Portugal	5-14 years	0.011	0.027	0.003
2005	Portugal	15-49 years	0.853	1.583	0.377
2005	Portugal	50-69 years	1.975	3.722	0.843
2005	Portugal	70+ years	1.334	2.624	0.586
2005	Portugal	All Ages	1.033	1.669	0.590
2010	Portugal	Under 5	0.001	0.004	0.000
2010	Portugal	5-14 years	0.008	0.019	0.002
2010	Portugal	15-49 years	0.486	0.895	0.227
2010	Portugal	50-69 years	1.178	2.237	0.490
2010	Portugal	70+ years	0.798	1.479	0.398
2010	Portugal	All Ages	0.620	1.006	0.346
2015	Portugal	Under 5	0.001	0.003	0.000
2015	Portugal	5-14 years	0.007	0.017	0.001
2015	Portugal	15-49 years	0.362	0.644	0.183
2015	Portugal	50-69 years	0.947	1.748	0.484
2015	Portugal	70+ years	0.613	1.123	0.301
2015	Portugal	All Ages	0.500	0.793	0.295
2019	Portugal	Under 5	0.001	0.004	0.000
2019	Portugal	5-14 years	0.007	0.019	0.001
2019	Portugal	15-49 years	0.322	0.573	0.166
2019	Portugal	50-69 years	0.862	1.581	0.440
2019	Portugal	70+ years	0.568	1.037	0.283
2019	Portugal	All Ages	0.465	0.748	0.272
2000	Spain	Under 5	0.005	0.013	0.001
2000	Spain	5-14 years	0.028	0.064	0.008
2000	Spain	15-49 years	0.778	1.343	0.409
2000	Spain	50-69 years	1.995	3.446	0.991
2000	Spain	70+ years	2.063	3.907	0.847
2000	Spain	All Ages	1.072	1.649	0.659
2005	Spain	Under 5	0.003	0.008	0.000
2005	Spain	5-14 years	0.016	0.037	0.004

2005	Spain	15-49 years	0.419	0.685	0.244
2005	Spain	50-69 years	1.068	1.775	0.630
2005	Spain	70+ years	1.196	2.091	0.654
2005	Spain	All Ages	0.590	0.887	0.374
2010	Spain	Under 5	0.003	0.008	0.000
2010	Spain	5-14 years	0.015	0.036	0.004
2010	Spain	15-49 years	0.380	0.619	0.224
2010	Spain	50-69 years	0.981	1.625	0.588
2010	Spain	70+ years	1.051	1.855	0.571
2010	Spain	All Ages	0.540	0.813	0.345
2015	Spain	Under 5	0.003	0.007	0.000
2015	Spain	5-14 years	0.014	0.033	0.003
2015	Spain	15-49 years	0.291	0.469	0.168
2015	Spain	50-69 years	0.803	1.362	0.471
2015	Spain	70+ years	0.831	1.463	0.454
2015	Spain	All Ages	0.444	0.681	0.283
2019	Spain	Under 5	0.003	0.008	0.000
2019	Spain	5-14 years	0.015	0.036	0.003
2019	Spain	15-49 years	0.275	0.455	0.162
2019	Spain	50-69 years	0.759	1.275	0.442
2019	Spain	70+ years	0.777	1.376	0.430
2019	Spain	All Ages	0.434	0.657	0.276

Table S54- Rates and 95% uncertainty levels (UL) of **disability-adjusted life years** (DALYs) **due to cirrhosis and other chronic liver diseases due to hepatitis B** per 100,000 population in Greece, Italy, Portugal and Spain from 2000 to 2019 by age group (Global Burden of Disease Study 2019).

Year	Country	Age	DALYs (95% UL)	95% UL (upper)	95% UL (lower)
2000	Greece	Under 5	0.03	0.06	0.01
2000	Greece	5-14 years	0.07	0.12	0.03
2000	Greece	15-49 years	16.8	25.6	10.7
2000	Greece	50-69 years	67.4	105.1	41.1
2000	Greece	70+ years	104.8	158.4	61.0
2000	Greece	All Ages	35.2	48.0	25.1
2005	Greece	Under 5	0.02	0.05	0.01
2005	Greece	5-14 years	0.07	0.12	0.03
2005	Greece	15-49 years	18.9	28.6	11.8
2005	Greece	50-69 years	65.9	102.8	39.7
2005	Greece	70+ years	82.2	124.6	47.6
2005	Greece	All Ages	34.6	47.0	24.8
2010	Greece	Under 5	0.01	0.03	0.00
2010	Greece	5-14 years	0.03	0.05	0.01
2010	Greece	15-49 years	11.7	18.4	7.01
2010	Greece	50-69 years	37.6	60.4	21.3
2010	Greece	70+ years	37.7	59.1	20.9
2010	Greece	All Ages	19.6	27.1	13.7
2015	Greece	Under 5	0.01	0.02	0.00
2015	Greece	5-14 years	0.02	0.05	0.01
2015	Greece	15-49 years	11.1	17.7	6.61
2015	Greece	50-69 years	38.0	60.3	21.4
2015	Greece	70+ years	33.6	51.1	19.2
2015	Greece	All Ages	19.7	27.3	13.4
2019	Greece	Under 5	0.01	0.02	0.00
2019	Greece	5-14 years	0.02	0.05	0.01
2019	Greece	15-49 years	11.3	18.4	6.64
2019	Greece	50-69 years	36.1	57.6	21.0
2019	Greece	70+ years	35.3	55.1	20.1
2019	Greece	All Ages	20.1	27.8	13.8
2000	Italy	Under 5	0.04	0.06	0.02
2000	Italy	5-14 years	0.08	0.12	0.05
2000	Italy	15-49 years	35.3	40.1	31.2
2000	Italy	50-69 years	128.1	147.0	110.1
2000	Italy	70+ years	150.5	178.2	123.4
2000	Italy	All Ages	67.4	74.0	60.7
2005	Italy	Under 5	0.02	0.04	0.01
2005	Italy	5-14 years	0.05	0.07	0.03
2005	Italy	15-49 years	30.0	35.1	25.8
2005	Italy	50-69 years	93.1	109.3	78.8
2005	Italy	70+ years	118.3	143.4	95.4
2005	Italy	All Ages	53.2	59.5	47.4
2010	Italy	Under 5	0.02	0.03	0.01
2010	Italy	5-14 years	0.05	0.07	0.03
2010	Italy	15-49 years	22.7	27.3	18.9
2010	Italy	50-69 years	68.2	81.5	56.7
2010	Italy	70+ years	90.7	110.9	72.1
2010	Italy	All Ages	40.7	45.7	35.4

2015	Italy	Under 5	0.01	0.02	0.00
2015	Italy	5-14 years	0.04	0.05	0.02
2015	Italy	15-49 years	19.2	23.1	15.9
2015	Italy	50-69 years	62.1	74.3	51.4
2015	Italy	70+ years	78.0	94.9	61.8
2015	Italy	All Ages	37.1	42.3	32.5
2019	Italy	Under 5	0.01	0.02	0.00
2019	Italy	5-14 years	0.04	0.06	0.02
2019	Italy	15-49 years	18.6	22.4	15.1
2019	Italy	50-69 years	59.2	70.9	48.9
2019	Italy	70+ years	75.7	93.2	60.5
2019	Italy	All Ages	37.1	42.5	32.4
2000	Portugal	Under 5	0.05	0.10	0.01
2000	Portugal	5-14 years	0.14	0.28	0.05
2000	Portugal	15-49 years	75.2	114.9	46.4
2000	Portugal	50-69 years	182.	285.	109.
2000	Portugal	70+ years	129.1	200.6	72.1
2000	Portugal	All Ages	92.3	124.9	65.1
2005	Portugal	Under 5	0.02	0.05	0.00
2005	Portugal	5-14 years	0.09	0.17	0.03
2005	Portugal	15-49 years	60.8	94.6	36.6
2005	Portugal	50-69 years	142.9	226.3	84.9
2005	Portugal	70+ years	93.4	145.7	52.7
2005	Portugal	All Ages	73.8	102.6	51.5
2010	Portugal	Under 5	0.00	0.02	0.00
2010	Portugal	5-14 years	0.04	0.07	0.01
2010	Portugal	15-49 years	35.8	57.4	20.9
2010	Portugal	50-69 years	88.2	141.3	50.2
2010	Portugal	70+ years	54.2	86.7	30.6
2010	Portugal	All Ages	45.4	64.1	30.5
2015	Portugal	Under 5	0.00	0.01	0.00
2015	Portugal	5-14 years	0.02	0.04	0.00
2015	Portugal	15-49 years	21.6	35.2	12.6
2015	Portugal	50-69 years	64.0	102.6	36.4
2015	Portugal	70+ years	39.0	61.4	22.1
2015	Portugal	All Ages	32.1	45.6	21.5
2019	Portugal	Under 5	0.00	0.01	0.00
2019	Portugal	5-14 years	0.02	0.04	0.00
2019	Portugal	15-49 years	23.5	38.5	13.6
2019	Portugal	50-69 years	61.5	97.7	34.4
2019	Portugal	70+ years	39.2	62.5	21.6
2019	Portugal	All Ages	33.2	46.8	22.5
2000	Spain	Under 5	0.04	0.08	0.01
2000	Spain	5-14 years	0.12	0.20	0.05
2000	Spain	15-49 years	41.0	55.5	29.3
2000	Spain	50-69 years	124.0	172.7	88.6
2000	Spain	70+ years	143.3	214.9	90.5
2000	Spain	All Ages	64.3	81.1	50.8
2005	Spain	Under 5	0.02	0.04	0.00
2005	Spain	5-14 years	0.05	0.10	0.02
2005	Spain	15-49 years	23.4	32.9	16.1
2005	Spain	50-69 years	69.8	98.8	49.3
2005	Spain	70+ years	76.9	117.3	47.0

2005	Spain	All Ages	36.2	46.2	27.7
2010	Spain	Under 5	0.01	0.03	0.00
2010	Spain	5-14 years	0.05	0.08	0.02
2010	Spain	15-49 years	18.0	25.0	12.4
2010	Spain	50-69 years	59.2	84.4	41.4
2010	Spain	70+ years	60.4	89.9	37.9
2010	Spain	All Ages	29.6	38.1	22.8
2015	Spain	Under 5	0.01	0.02	0.00
2015	Spain	5-14 years	0.03	0.06	0.01
2015	Spain	15-49 years	12.0	17.0	8.26
2015	Spain	50-69 years	48.2	68.4	33.7
2015	Spain	70+ years	48.2	73.2	29.4
2015	Spain	All Ages	23.7	31.1	18.2
2019	Spain	Under 5	0.01	0.02	0.00
2019	Spain	5-14 years	0.03	0.06	0.01
2019	Spain	15-49 years	12.8	18.4	8.88
2019	Spain	50-69 years	44.0	62.6	30.7
2019	Spain	70+ years	46.5	70.8	28.2
2019	Spain	All Ages	23.9	31.3	18.3

Table S55- Rates and 95% uncertainty levels (UL) of **prevalence of cirrhosis and other chronic liver diseases due to hepatitis C** per 100,000 population in Greece, Italy, Portugal and Spain from 2000 to 2019 by age group (Global Burden of Disease Study 2019).

Year	Country	Age	Prevalence (95% UL)	95% UL (upper)	95% UL (lower)
2000	Greece	Under 5	50.869	70.542	34.532
2000	Greece	5-14 years	121.30	159.57	86.694
2000	Greece	15-49 years	277.80	342.39	220.46
2000	Greece	50-69 years	579.31	711.44	471.27
2000	Greece	70+ years	976.63	1202.8	782.49
2000	Greece	All Ages	393.68	475.67	321.74
2005	Greece	Under 5	58.537	84.007	38.538
2005	Greece	5-14 years	146.75	201.09	101.94
2005	Greece	15-49 years	364.01	467.43	282.72
2005	Greece	50-69 years	768.89	976.36	613.20
2005	Greece	70+ years	1326.1	1670.5	1047.9
2005	Greece	All Ages	539.42	673.16	441.29
2010	Greece	Under 5	58.517	83.676	39.111
2010	Greece	5-14 years	144.92	198.75	103.12
2010	Greece	15-49 years	366.32	465.51	285.38
2010	Greece	50-69 years	753.44	945.68	603.62
2010	Greece	70+ years	1335.5	1680.9	1054.0
2010	Greece	All Ages	556.33	680.36	457.06
2015	Greece	Under 5	57.638	82.621	38.605
2015	Greece	5-14 years	141.84	192.88	100.68
2015	Greece	15-49 years	366.93	465.15	286.79
2015	Greece	50-69 years	747.28	941.22	601.37
2015	Greece	70+ years	1370.3	1740.9	1086.5
2015	Greece	All Ages	581.27	717.88	474.12
2019	Greece	Under 5	58.864	83.804	39.229
2019	Greece	5-14 years	145.69	197.30	103.87
2019	Greece	15-49 years	375.79	475.75	292.48
2019	Greece	50-69 years	758.81	944.78	609.07
2019	Greece	70+ years	1404.0	1764.8	1093.5
2019	Greece	All Ages	609.84	746.84	496.81
2000	Italy	Under 5	205.94	298.08	134.61
2000	Italy	5-14 years	666.47	917.15	474.52
2000	Italy	15-49 years	1606.0	2032.7	1268.6
2000	Italy	50-69 years	3406.3	4238.4	2723.0
2000	Italy	70+ years	5290.6	6671.1	4120.7
2000	Italy	All Ages	2357.1	2842.5	1923.0
2005	Italy	Under 5	181.63	262.88	118.67
2005	Italy	5-14 years	546.93	752.70	387.57
2005	Italy	15-49 years	1323.9	1681.9	1047.5
2005	Italy	50-69 years	2777.3	3440.6	2231.5
2005	Italy	70+ years	4421.7	5615.8	3478.0
2005	Italy	All Ages	1976.5	2391.5	1621.5
2010	Italy	Under 5	186.39	269.08	120.48
2010	Italy	5-14 years	552.86	765.88	388.36
2010	Italy	15-49 years	1351.6	1721.6	1055.1
2010	Italy	50-69 years	2688.5	3373.4	2146.3

2010	Italy	70+ years	4102.2	5198.6	3199.3
2010	Italy	All Ages	1957.8	2392.6	1593.4
2015	Italy	Under 5	173.28	247.45	113.66
2015	Italy	5-14 years	487.23	661.62	345.54
2015	Italy	15-49 years	1152.6	1477.4	900.95
2015	Italy	50-69 years	2226.9	2784.4	1790.4
2015	Italy	70+ years	3498.1	4456.9	2713.9
2015	Italy	All Ages	1700.0	2067.2	1380.3
2019	Italy	Under 5	177.53	253.16	115.57
2019	Italy	5-14 years	503.54	686.47	359.17
2019	Italy	15-49 years	1178.9	1511.7	917.81
2019	Italy	50-69 years	2260.3	2827.9	1807.9
2019	Italy	70+ years	3569.0	4553.0	2773.1
2019	Italy	All Ages	1784.6	2189.3	1444.1
2000	Portugal	Under 5	101.36	148.12	65.590
2000	Portugal	5-14 years	279.60	382.86	196.72
2000	Portugal	15-49 years	695.30	871.61	552.54
2000	Portugal	50-69 years	1360.6	1697.1	1085.3
2000	Portugal	70+ years	2054.1	2571.5	1630.9
2000	Portugal	All Ages	909.87	1104.8	745.50
2005	Portugal	Under 5	100.59	146.36	65.530
2005	Portugal	5-14 years	267.52	365.87	185.24
2005	Portugal	15-49 years	678.13	855.09	540.01
2005	Portugal	50-69 years	1314.5	1632.0	1062.9
2005	Portugal	70+ years	2019.1	2530.3	1614.6
2005	Portugal	All Ages	911.23	1105.3	748.92
2010	Portugal	Under 5	98.741	143.94	64.548
2010	Portugal	5-14 years	261.43	357.78	184.60
2010	Portugal	15-49 years	659.31	829.28	515.68
2010	Portugal	50-69 years	1269.6	1569.9	1028.3
2010	Portugal	70+ years	2013.1	2532.8	1608.3
2010	Portugal	All Ages	919.57	1116.6	751.89
2015	Portugal	Under 5	95.934	141.51	64.468
2015	Portugal	5-14 years	251.50	340.02	179.57
2015	Portugal	15-49 years	630.17	787.80	496.37
2015	Portugal	50-69 years	1242.5	1555.4	981.49
2015	Portugal	70+ years	2016.2	2568.7	1590.6
2015	Portugal	All Ages	932.12	1139.9	759.92
2019	Portugal	Under 5	97.763	144.04	63.945
2019	Portugal	5-14 years	254.46	346.96	181.19
2019	Portugal	15-49 years	631.52	793.24	496.70
2019	Portugal	50-69 years	1233.2	1531.5	986.30
2019	Portugal	70+ years	2020.9	2520.8	1613.1
2019	Portugal	All Ages	960.17	1163.2	788.97
2000	Spain	Under 5	99.480	142.47	67.246
2000	Spain	5-14 years	270.10	353.97	198.26
2000	Spain	15-49 years	677.23	803.26	566.02
2000	Spain	50-69 years	1381.6	1622.9	1162.8
2000	Spain	70+ years	1983.9	2375.8	1660.6
2000	Spain	All Ages	909.46	1059.9	773.65
2005	Spain	Under 5	88.789	124.85	59.792
2005	Spain	5-14 years	228.78	302.11	170.16
2005	Spain	15-49 years	557.60	663.31	461.43

2005	Spain	50-69 years	1120.2	1318.8	942.83
2005	Spain	70+ years	1732.7	2082.1	1432.3
2005	Spain	All Ages	764.40	890.57	652.21
2010	Spain	Under 5	97.363	142.96	63.275
2010	Spain	5-14 years	254.16	352.24	176.94
2010	Spain	15-49 years	676.66	853.45	532.26
2010	Spain	50-69 years	1401.0	1759.2	1109.8
2010	Spain	70+ years	2308.0	2965.8	1823.3
2010	Spain	All Ages	966.54	1178.8	779.57
2015	Spain	Under 5	92.894	133.14	62.115
2015	Spain	5-14 years	238.16	328.30	169.02
2015	Spain	15-49 years	639.37	809.19	498.81
2015	Spain	50-69 years	1324.9	1675.8	1049.5
2015	Spain	70+ years	2222.7	2854.1	1768.2
2015	Spain	All Ages	948.30	1171.7	773.38
2019	Spain	Under 5	93.088	135.66	62.265
2019	Spain	5-14 years	239.91	328.12	168.90
2019	Spain	15-49 years	635.89	796.50	501.86
2019	Spain	50-69 years	1306.1	1631.8	1044.6
2019	Spain	70+ years	2233.6	2799.1	1750.1
2019	Spain	All Ages	975.33	1182.6	789.56

Table S56- Rates and 95% uncertainty levels (UL) of **incidence of cirrhosis and other chronic liver diseases due to hepatitis C** per 100,000 population in Greece, Italy, Portugal and Spain from 2000 to 2019 by age group (Global Burden of Disease Study 2019).

Year	Country	Age	Incidence (95% UL)	95% UL (upper)	95% UL (lower)
2000	Greece	Under 5	0.01	0.04	0.00
2000	Greece	5-14 years	0.05	0.13	0.01
2000	Greece	15-49 years	5.86	9.07	3.54
2000	Greece	50-69 years	4.55	7.97	2.02
2000	Greece	70+ years	0.10	0.37	0.0
2000	Greece	All Ages	4.04	6.06	2.54
2005	Greece	Under 5	0.01	0.04	0.00
2005	Greece	5-14 years	0.05	0.13	0.01
2005	Greece	15-49 years	5.73	8.87	3.47
2005	Greece	50-69 years	3.46	6.36	1.45
2005	Greece	70+ years	0.08	0.31	0.0
2005	Greece	All Ages	3.67	5.51	2.34
2010	Greece	Under 5	0.01	0.04	0.00
2010	Greece	5-14 years	0.05	0.13	0.01
2010	Greece	15-49 years	6.47	9.80	3.99
2010	Greece	50-69 years	2.83	5.43	1.12
2010	Greece	70+ years	0.04	0.19	0.0
2010	Greece	All Ages	3.78	5.58	2.42
2015	Greece	Under 5	0.01	0.04	0.00
2015	Greece	5-14 years	0.05	0.13	0.01
2015	Greece	15-49 years	6.59	10.2	3.97
2015	Greece	50-69 years	2.42	4.60	0.93
2015	Greece	70+ years	0.04	0.17	0.0
2015	Greece	All Ages	3.59	5.36	2.25
2019	Greece	Under 5	0.01	0.04	0.00
2019	Greece	5-14 years	0.06	0.14	0.02
2019	Greece	15-49 years	6.57	10.1	3.97
2019	Greece	50-69 years	2.08	3.98	0.75
2019	Greece	70+ years	0.05	0.24	0.0
2019	Greece	All Ages	3.40	5.16	2.09
2000	Italy	Under 5	0.08	0.15	0.02
2000	Italy	5-14 years	0.21	0.40	0.09
2000	Italy	15-49 years	31.6	40.5	23.8
2000	Italy	50-69 years	20.3	31.8	10.5
2000	Italy	70+ years	0.08	0.58	0.0
2000	Italy	All Ages	20.3	24.9	16.1
2005	Italy	Under 5	0.07	0.14	0.03
2005	Italy	5-14 years	0.16	0.31	0.07
2005	Italy	15-49 years	30.6	39.0	23.5
2005	Italy	50-69 years	19.8	29.8	10.6
2005	Italy	70+ years	0.05	0.44	0.0
2005	Italy	All Ages	19.4	23.6	15.6
2010	Italy	Under 5	0.07	0.13	0.03
2010	Italy	5-14 years	0.14	0.27	0.06
2010	Italy	15-49 years	28.3	36.0	21.4
2010	Italy	50-69 years	18.2	27.3	10.4
2010	Italy	70+ years	0.04	0.40	0.0

2010	Italy	All Ages	17.6	21.5	14.2
2015	Italy	Under 5	0.06	0.13	0.02
2015	Italy	5-14 years	0.14	0.26	0.06
2015	Italy	15-49 years	26.6	34.3	19.8
2015	Italy	50-69 years	17.3	26.3	9.72
2015	Italy	70+ years	0.04	0.35	0.0
2015	Italy	All Ages	16.3	20.2	12.9
2019	Italy	Under 5	0.06	0.13	0.02
2019	Italy	5-14 years	0.16	0.30	0.06
2019	Italy	15-49 years	25.2	33.5	17.8
2019	Italy	50-69 years	16.6	26.2	8.40
2019	Italy	70+ years	0.09	0.77	0.0
2019	Italy	All Ages	15.2	19.5	11.4
2000	Portugal	Under 5	0.01	0.03	0.00
2000	Portugal	5-14 years	0.05	0.12	0.01
2000	Portugal	15-49 years	12.8	19.3	7.92
2000	Portugal	50-69 years	3.48	6.69	1.05
2000	Portugal	70+ years	0.00	0.04	0.0
2000	Portugal	All Ages	7.35	10.6	4.74
2005	Portugal	Under 5	0.01	0.03	0.00
2005	Portugal	5-14 years	0.04	0.10	0.01
2005	Portugal	15-49 years	12.3	18.4	7.51
2005	Portugal	50-69 years	2.55	5.24	0.69
2005	Portugal	70+ years	0.01	0.08	0.0
2005	Portugal	All Ages	6.70	9.87	4.18
2010	Portugal	Under 5	0.01	0.02	0.00
2010	Portugal	5-14 years	0.04	0.10	0.01
2010	Portugal	15-49 years	11.1	17.0	6.68
2010	Portugal	50-69 years	2.11	4.47	0.53
2010	Portugal	70+ years	0.03	0.15	0.0
2010	Portugal	All Ages	5.81	8.68	3.63
2015	Portugal	Under 5	0.01	0.02	0.00
2015	Portugal	5-14 years	0.04	0.10	0.01
2015	Portugal	15-49 years	10.4	16.0	6.30
2015	Portugal	50-69 years	2.04	4.16	0.55
2015	Portugal	70+ years	0.05	0.20	0.0
2015	Portugal	All Ages	5.29	7.87	3.31
2019	Portugal	Under 5	0.01	0.02	0.00
2019	Portugal	5-14 years	0.04	0.10	0.01
2019	Portugal	15-49 years	9.15	14.0	5.47
2019	Portugal	50-69 years	1.81	3.94	0.37
2019	Portugal	70+ years	0.10	0.36	0.0
2019	Portugal	All Ages	4.53	6.82	2.79
2000	Spain	Under 5	0.03	0.08	0.00
2000	Spain	5-14 years	0.13	0.30	0.04
2000	Spain	15-49 years	16.6	23.4	11.2
2000	Spain	50-69 years	9.69	16.9	3.96
2000	Spain	70+ years	0.21	0.72	0.0
2000	Spain	All Ages	10.8	14.3	7.65
2005	Spain	Under 5	0.03	0.08	0.00
2005	Spain	5-14 years	0.12	0.28	0.04
2005	Spain	15-49 years	17.7	24.7	12.2
2005	Spain	50-69 years	7.66	12.9	3.55

2005	Spain	70+ years	0.22	0.75	0.0
2005	Spain	All Ages	10.9	14.7	7.75
2010	Spain	Under 5	0.03	0.08	0.00
2010	Spain	5-14 years	0.12	0.27	0.04
2010	Spain	15-49 years	16.7	23.4	11.2
2010	Spain	50-69 years	7.41	12.8	3.34
2010	Spain	70+ years	0.28	0.86	0.0
2010	Spain	All Ages	10.2	13.6	7.00
2015	Spain	Under 5	0.03	0.08	0.00
2015	Spain	5-14 years	0.11	0.26	0.04
2015	Spain	15-49 years	16.5	23.4	10.9
2015	Spain	50-69 years	6.88	11.6	2.79
2015	Spain	70+ years	0.39	1.38	0.00
2015	Spain	All Ages	9.59	13.0	6.60
2019	Spain	Under 5	0.03	0.08	0.00
2019	Spain	5-14 years	0.12	0.28	0.04
2019	Spain	15-49 years	15.5	22.5	10.1
2019	Spain	50-69 years	6.20	10.9	2.90
2019	Spain	70+ years	0.50	1.62	0.0
2019	Spain	All Ages	8.68	11.9	5.89

Table S57- Rates and 95% uncertainty levels (UL) of **deaths due to cirrhosis and other chronic liver diseases due to hepatitis C** per 100,000 population in Greece, Italy, Portugal and Spain from 2000 to 2019 by age group (Global Burden of Disease Study 2019).

Year	Country	Age	Deaths (95% UL)	95% UL (upper)	95% UL (lower)
2000	Greece	Under 5	0.000	0.001	0.000
2000	Greece	5-14 years	0.001	0.002	0.000
2000	Greece	15-49 years	0.433	0.644	0.277
2000	Greece	50-69 years	2.935	4.678	1.753
2000	Greece	70+ years	10.63	15.46	6.562
2000	Greece	All Ages	2.027	2.777	1.433
2005	Greece	Under 5	0.000	0.001	0.000
2005	Greece	5-14 years	0.001	0.002	0.000
2005	Greece	15-49 years	0.481	0.721	0.309
2005	Greece	50-69 years	2.713	4.310	1.643
2005	Greece	70+ years	8.032	11.88	4.973
2005	Greece	All Ages	1.857	2.535	1.317
2010	Greece	Under 5	0.000	0.001	0.000
2010	Greece	5-14 years	0.000	0.001	0.000
2010	Greece	15-49 years	0.560	0.825	0.360
2010	Greece	50-69 years	2.828	4.472	1.736
2010	Greece	70+ years	7.077	10.95	4.248
2010	Greece	All Ages	1.926	2.663	1.369
2015	Greece	Under 5	0.000	0.001	0.000
2015	Greece	5-14 years	0.000	0.001	0.000
2015	Greece	15-49 years	0.548	0.804	0.346
2015	Greece	50-69 years	2.948	4.774	1.768
2015	Greece	70+ years	6.831	10.41	4.147
2015	Greece	All Ages	2.042	2.803	1.455
2019	Greece	Under 5	0.000	0.000	0.000
2019	Greece	5-14 years	0.000	0.001	0.000
2019	Greece	15-49 years	0.560	0.838	0.351
2019	Greece	50-69 years	2.825	4.524	1.720
2019	Greece	70+ years	7.471	11.38	4.549
2019	Greece	All Ages	2.218	3.000	1.558
2000	Italy	Under 5	0.002	0.003	0.001
2000	Italy	5-14 years	0.003	0.005	0.002
2000	Italy	15-49 years	1.974	2.188	1.777
2000	Italy	50-69 years	12.55	14.56	10.80
2000	Italy	70+ years	33.94	40.08	27.87
2000	Italy	All Ages	8.347	9.376	7.401
2005	Italy	Under 5	0.001	0.002	0.000
2005	Italy	5-14 years	0.002	0.003	0.001
2005	Italy	15-49 years	1.679	1.908	1.463
2005	Italy	50-69 years	9.700	11.57	8.149
2005	Italy	70+ years	29.29	35.26	23.56
2005	Italy	All Ages	7.208	8.214	6.285
2010	Italy	Under 5	0.000	0.001	0.000
2010	Italy	5-14 years	0.002	0.003	0.001
2010	Italy	15-49 years	1.525	1.760	1.293
2010	Italy	50-69 years	8.390	10.14	6.876
2010	Italy	70+ years	26.96	32.45	21.22

2010	Italy	All Ages	6.783	7.790	5.818
2015	Italy	Under 5	0.000	0.001	0.000
2015	Italy	5-14 years	0.001	0.002	0.000
2015	Italy	15-49 years	1.323	1.544	1.125
2015	Italy	50-69 years	7.572	9.114	6.279
2015	Italy	70+ years	24.69	29.89	19.55
2015	Italy	All Ages	6.483	7.445	5.562
2019	Italy	Under 5	0.000	0.000	0.000
2019	Italy	5-14 years	0.001	0.002	0.000
2019	Italy	15-49 years	1.263	1.468	1.062
2019	Italy	50-69 years	7.191	8.659	5.961
2019	Italy	70+ years	24.19	29.26	19.19
2019	Italy	All Ages	6.652	7.680	5.707
2000	Portugal	Under 5	0.001	0.002	0.000
2000	Portugal	5-14 years	0.004	0.008	0.001
2000	Portugal	15-49 years	2.231	3.267	1.436
2000	Portugal	50-69 years	8.846	14.06	5.354
2000	Portugal	70+ years	13.66	20.84	8.610
2000	Portugal	All Ages	4.545	6.188	3.279
2005	Portugal	Under 5	0.000	0.001	0.000
2005	Portugal	5-14 years	0.002	0.005	0.001
2005	Portugal	15-49 years	2.023	2.999	1.276
2005	Portugal	50-69 years	7.487	11.87	4.499
2005	Portugal	70+ years	11.03	16.93	6.918
2005	Portugal	All Ages	4.032	5.532	2.942
2010	Portugal	Under 5	0.000	0.000	0.000
2010	Portugal	5-14 years	0.001	0.003	0.000
2010	Portugal	15-49 years	1.730	2.553	1.099
2010	Portugal	50-69 years	6.580	10.49	4.030
2010	Portugal	70+ years	9.689	14.65	6.121
2010	Portugal	All Ages	3.699	5.047	2.692
2015	Portugal	Under 5	0.000	0.000	0.000
2015	Portugal	5-14 years	0.000	0.001	0.000
2015	Portugal	15-49 years	1.220	1.816	0.757
2015	Portugal	50-69 years	5.530	8.813	3.317
2015	Portugal	70+ years	8.416	12.55	5.210
2015	Portugal	All Ages	3.217	4.404	2.306
2019	Portugal	Under 5	0.000	0.000	8.323
2019	Portugal	5-14 years	0.000	0.001	0.000
2019	Portugal	15-49 years	1.323	1.970	0.823
2019	Portugal	50-69 years	5.370	8.500	3.278
2019	Portugal	70+ years	8.667	12.71	5.270
2019	Portugal	All Ages	3.413	4.643	2.470
2000	Spain	Under 5	0.001	0.003	0.000
2000	Spain	5-14 years	0.003	0.007	0.001
2000	Spain	15-49 years	1.718	2.313	1.213
2000	Spain	50-69 years	9.324	13.52	6.060
2000	Spain	70+ years	23.54	33.52	15.27
2000	Spain	All Ages	5.607	7.278	4.230
2005	Spain	Under 5	0.001	0.002	0.000
2005	Spain	5-14 years	0.002	0.005	0.001
2005	Spain	15-49 years	1.719	2.305	1.213
2005	Spain	50-69 years	8.712	12.62	5.736

2005	Spain	70+ years	21.76	30.35	14.07
2005	Spain	All Ages	5.390	7.061	4.091
2010	Spain	Under 5	0.000	0.002	0.000
2010	Spain	5-14 years	0.002	0.004	0.001
2010	Spain	15-49 years	1.362	1.847	0.928
2010	Spain	50-69 years	7.402	10.78	4.879
2010	Spain	70+ years	18.28	26.61	11.73
2010	Spain	All Ages	4.579	5.934	3.447
2015	Spain	Under 5	0.000	0.001	0.000
2015	Spain	5-14 years	0.001	0.003	0.000
2015	Spain	15-49 years	1.025	1.393	0.704
2015	Spain	50-69 years	6.698	9.662	4.424
2015	Spain	70+ years	17.32	24.60	11.13
2015	Spain	All Ages	4.411	5.745	3.323
2019	Spain	Under 5	0.000	0.001	0.000
2019	Spain	5-14 years	0.001	0.003	0.000
2019	Spain	15-49 years	1.096	1.523	0.735
2019	Spain	50-69 years	6.108	8.856	3.959
2019	Spain	70+ years	16.89	23.89	10.81
2019	Spain	All Ages	4.512	5.865	3.364

Table S58- Rates and 95% uncertainty levels (UL) of **years of life lost** (YLLs) **due to cirrhosis and other chronic liver diseases due to hepatitis C** per 100,000 population in Greece, Italy, Portugal and Spain from 2000 to 2019 by age group (Global Burden of Disease Study 2019).

Year	Country	Age	YLLs (95% UL)	95% UL (upper)	95% UL (lower)
2000	Greece	Under 5	0.057	0.121	0.020
2000	Greece	5-14 years	0.092	0.181	0.039
2000	Greece	15-49 years	20.41	30.12	13.17
2000	Greece	50-69 years	84.37	135.8	50.26
2000	Greece	70+ years	139.9	210.1	86.15
2000	Greece	All Ages	44.63	60.70	32.38
2005	Greece	Under 5	0.047	0.098	0.017
2005	Greece	5-14 years	0.091	0.176	0.037
2005	Greece	15-49 years	22.47	33.60	14.60
2005	Greece	50-69 years	80.04	127.4	48.51
2005	Greece	70+ years	107.1	162.9	65.69
2005	Greece	All Ages	42.76	58.65	30.60
2010	Greece	Under 5	0.047	0.093	0.018
2010	Greece	5-14 years	0.073	0.142	0.030
2010	Greece	15-49 years	25.95	38.13	16.87
2010	Greece	50-69 years	85.10	134.6	52.31
2010	Greece	70+ years	93.26	143.8	56.26
2010	Greece	All Ages	45.32	61.53	33.04
2015	Greece	Under 5	0.043	0.093	0.016
2015	Greece	5-14 years	0.054	0.109	0.022
2015	Greece	15-49 years	25.01	36.55	16.02
2015	Greece	50-69 years	88.23	140.8	53.45
2015	Greece	70+ years	85.13	131.4	51.27
2015	Greece	All Ages	46.45	64.18	33.49
2019	Greece	Under 5	0.035	0.076	0.013
2019	Greece	5-14 years	0.055	0.113	0.022
2019	Greece	15-49 years	25.59	38.02	16.16
2019	Greece	50-69 years	84.54	134.5	51.18
2019	Greece	70+ years	90.39	136.6	55.19
2019	Greece	All Ages	48.06	65.48	35.03
2000	Italy	Under 5	0.173	0.288	0.092
2000	Italy	5-14 years	0.306	0.437	0.201
2000	Italy	15-49 years	93.39	103.1	84.25
2000	Italy	50-69 years	360.2	415.6	310.6
2000	Italy	70+ years	470.9	555.4	386.6
2000	Italy	All Ages	192.8	214.0	174.4
2005	Italy	Under 5	0.115	0.206	0.063
2005	Italy	5-14 years	0.209	0.299	0.134
2005	Italy	15-49 years	77.96	88.44	68.14
2005	Italy	50-69 years	279.1	332.1	236.1
2005	Italy	70+ years	397.3	481.6	320.4
2005	Italy	All Ages	159.6	179.7	142.3
2010	Italy	Under 5	0.083	0.146	0.044
2010	Italy	5-14 years	0.164	0.245	0.105

2010	Italy	15-49 years	69.65	80.25	59.43
2010	Italy	50-69 years	244.6	294.5	202.4
2010	Italy	70+ years	351.6	427.5	280.2
2010	Italy	All Ages	144.4	163.5	127.7
2015	Italy	Under 5	0.062	0.111	0.033
2015	Italy	5-14 years	0.123	0.180	0.077
2015	Italy	15-49 years	59.89	69.59	51.33
2015	Italy	50-69 years	222.2	266.2	185.1
2015	Italy	70+ years	308.1	374.0	244.9
2015	Italy	All Ages	133.4	151.5	117.6
2019	Italy	Under 5	0.047	0.083	0.026
2019	Italy	5-14 years	0.118	0.174	0.075
2019	Italy	15-49 years	57.28	66.36	48.37
2019	Italy	50-69 years	211.9	254.2	175.3
2019	Italy	70+ years	299.6	362.7	239.5
2019	Italy	All Ages	133.9	152.4	118.0
2000	Portugal	Under 5	0.119	0.249	0.043
2000	Portugal	5-14 years	0.332	0.664	0.145
2000	Portugal	15-49 years	106.4	154.6	69.30
2000	Portugal	50-69 years	263.0	420.4	159.4
2000	Portugal	70+ years	196.8	302.2	122.4
2000	Portugal	All Ages	133.1	181.2	95.09
2005	Portugal	Under 5	0.070	0.154	0.025
2005	Portugal	5-14 years	0.226	0.451	0.099
2005	Portugal	15-49 years	94.68	139.2	60.05
2005	Portugal	50-69 years	226.0	359.1	135.4
2005	Portugal	70+ years	157.1	243.5	96.66
2005	Portugal	All Ages	117.1	159.7	83.29
2010	Portugal	Under 5	0.031	0.066	0.012
2010	Portugal	5-14 years	0.124	0.243	0.052
2010	Portugal	15-49 years	80.11	118.6	51.28
2010	Portugal	50-69 years	201.0	317.0	123.5
2010	Portugal	70+ years	133.5	201.0	82.44
2010	Portugal	All Ages	104.1	142.3	75.40
2015	Portugal	Under 5	0.026	0.057	0.009
2015	Portugal	5-14 years	0.070	0.140	0.028
2015	Portugal	15-49 years	55.53	82.96	34.87
2015	Portugal	50-69 years	168.4	266.7	101.9
2015	Portugal	70+ years	110.6	169.0	69.66
2015	Portugal	All Ages	84.94	118.8	60.84
2019	Portugal	Under 5	0.020	0.046	0.007
2019	Portugal	5-14 years	0.072	0.143	0.028
2019	Portugal	15-49 years	60.42	90.27	38.03
2019	Portugal	50-69 years	162.8	261.0	98.87
2019	Portugal	70+ years	112.6	170.0	67.44
2019	Portugal	All Ages	88.40	124.0	62.33
2000	Spain	Under 5	0.135	0.278	0.055
2000	Spain	5-14 years	0.306	0.591	0.143
2000	Spain	15-49 years	81.57	109.7	57.80
2000	Spain	50-69 years	271.9	392.7	178.3
2000	Spain	70+ years	317.4	454.5	205.0

2000	Spain	All Ages	137.1	175.3	106.3
2005	Spain	Under 5	0.110	0.215	0.046
2005	Spain	5-14 years	0.233	0.455	0.107
2005	Spain	15-49 years	79.68	106.6	56.00
2005	Spain	50-69 years	260.7	375.5	171.5
2005	Spain	70+ years	288.0	408.2	183.5
2005	Spain	All Ages	131.3	166.7	100.5
2010	Spain	Under 5	0.085	0.176	0.034
2010	Spain	5-14 years	0.189	0.356	0.086
2010	Spain	15-49 years	61.91	83.12	42.57
2010	Spain	50-69 years	224.2	326.0	148.4
2010	Spain	70+ years	230.7	336.1	149.0
2010	Spain	All Ages	109.2	139.6	84.19
2015	Spain	Under 5	0.057	0.121	0.021
2015	Spain	5-14 years	0.140	0.271	0.062
2015	Spain	15-49 years	46.08	62.29	31.77
2015	Spain	50-69 years	203.0	291.3	135.2
2015	Spain	70+ years	208.2	301.4	135.2
2015	Spain	All Ages	98.63	127.9	76.18
2019	Spain	Under 5	0.051	0.111	0.020
2019	Spain	5-14 years	0.136	0.259	0.062
2019	Spain	15-49 years	49.44	68.47	33.50
2019	Spain	50-69 years	185.2	267.3	120.7
2019	Spain	70+ years	199.7	286.4	127.7
2019	Spain	All Ages	99.08	128.6	75.84

Table S59- Rates and 95% uncertainty levels (UL) of **years lived with disability** (YLDs) **due to cirrhosis and other chronic liver diseases due to hepatitis C** per 100,000 population in Greece, Italy, Portugal and Spain from 2000 to 2019 by age group (Global Burden of Disease Study 2019).

Year	Country	Age	YLDs (95% UL)	95% UL (upper)	95% UL (lower)
2000	Greece	Under 5	0.009	0.021	0.003
2000	Greece	5-14 years	0.045	0.102	0.016
2000	Greece	15-49 years	0.555	0.904	0.310
2000	Greece	50-69 years	1.389	2.549	0.568
2000	Greece	70+ years	2.017	3.715	0.934
2000	Greece	All Ages	0.820	1.307	0.472
2005	Greece	Under 5	0.009	0.021	0.003
2005	Greece	5-14 years	0.046	0.102	0.016
2005	Greece	15-49 years	0.571	1.015	0.287
2005	Greece	50-69 years	1.326	2.531	0.555
2005	Greece	70+ years	1.709	3.056	0.820
2005	Greece	All Ages	0.805	1.294	0.469
2010	Greece	Under 5	0.009	0.022	0.003
2010	Greece	5-14 years	0.046	0.103	0.016
2010	Greece	15-49 years	0.641	1.116	0.328
2010	Greece	50-69 years	1.400	2.712	0.577
2010	Greece	70+ years	1.610	2.872	0.758
2010	Greece	All Ages	0.864	1.384	0.507
2015	Greece	Under 5	0.009	0.021	0.003
2015	Greece	5-14 years	0.045	0.096	0.016
2015	Greece	15-49 years	0.650	1.151	0.324
2015	Greece	50-69 years	1.393	2.755	0.540
2015	Greece	70+ years	1.490	2.591	0.743
2015	Greece	All Ages	0.876	1.429	0.490
2019	Greece	Under 5	0.010	0.023	0.003
2019	Greece	5-14 years	0.050	0.110	0.018
2019	Greece	15-49 years	0.688	1.200	0.341
2019	Greece	50-69 years	1.373	2.560	0.595
2019	Greece	70+ years	1.446	2.532	0.763
2019	Greece	All Ages	0.900	1.409	0.528
2000	Italy	Under 5	0.031	0.056	0.015
2000	Italy	5-14 years	0.153	0.271	0.079
2000	Italy	15-49 years	2.363	3.551	1.529
2000	Italy	50-69 years	7.164	10.73	4.563
2000	Italy	70+ years	9.769	14.41	6.087
2000	Italy	All Ages	4.148	5.973	2.830
2005	Italy	Under 5	0.028	0.053	0.014
2005	Italy	5-14 years	0.128	0.226	0.066
2005	Italy	15-49 years	2.216	3.306	1.453
2005	Italy	50-69 years	6.828	10.17	4.258
2005	Italy	70+ years	10.21	15.27	6.408
2005	Italy	All Ages	4.135	5.868	2.851
2010	Italy	Under 5	0.027	0.050	0.013
2010	Italy	5-14 years	0.115	0.200	0.059
2010	Italy	15-49 years	1.972	2.965	1.278
2010	Italy	50-69 years	6.275	9.533	3.967
2010	Italy	70+ years	9.651	14.74	6.007

2010	Italy	All Ages	3.899	5.629	2.653
2015	Italy	Under 5	0.026	0.048	0.012
2015	Italy	5-14 years	0.111	0.197	0.058
2015	Italy	15-49 years	1.753	2.614	1.134
2015	Italy	50-69 years	5.610	8.481	3.542
2015	Italy	70+ years	8.407	12.80	5.192
2015	Italy	All Ages	3.586	5.168	2.452
2019	Italy	Under 5	0.027	0.051	0.013
2019	Italy	5-14 years	0.129	0.232	0.065
2019	Italy	15-49 years	1.680	2.565	1.083
2019	Italy	50-69 years	5.267	8.061	3.230
2019	Italy	70+ years	7.447	11.34	4.596
2019	Italy	All Ages	3.450	4.991	2.307
2000	Portugal	Under 5	0.006	0.015	0.002
2000	Portugal	5-14 years	0.031	0.071	0.011
2000	Portugal	15-49 years	1.607	2.905	0.787
2000	Portugal	50-69 years	3.522	6.449	1.657
2000	Portugal	70+ years	2.544	4.525	1.170
2000	Portugal	All Ages	1.869	3.026	1.079
2005	Portugal	Under 5	0.006	0.016	0.002
2005	Portugal	5-14 years	0.032	0.071	0.011
2005	Portugal	15-49 years	1.543	2.735	0.755
2005	Portugal	50-69 years	3.269	5.909	1.501
2005	Portugal	70+ years	2.306	4.166	1.115
2005	Portugal	All Ages	1.788	2.872	1.051
2010	Portugal	Under 5	0.007	0.016	0.002
2010	Portugal	5-14 years	0.033	0.073	0.011
2010	Portugal	15-49 years	1.354	2.304	0.653
2010	Portugal	50-69 years	2.799	5.111	1.308
2010	Portugal	70+ years	1.975	3.455	1.028
2010	Portugal	All Ages	1.582	2.560	0.926
2015	Portugal	Under 5	0.007	0.017	0.002
2015	Portugal	5-14 years	0.034	0.075	0.012
2015	Portugal	15-49 years	1.179	1.994	0.566
2015	Portugal	50-69 years	2.621	4.592	1.249
2015	Portugal	70+ years	1.840	3.259	0.932
2015	Portugal	All Ages	1.486	2.359	0.861
2019	Portugal	Under 5	0.007	0.017	0.002
2019	Portugal	5-14 years	0.035	0.079	0.013
2019	Portugal	15-49 years	1.043	1.942	0.481
2019	Portugal	50-69 years	2.446	4.503	1.160
2019	Portugal	70+ years	1.717	3.033	0.828
2019	Portugal	All Ages	1.395	2.266	0.812
2000	Spain	Under 5	0.019	0.041	0.006
2000	Spain	5-14 years	0.091	0.200	0.035
2000	Spain	15-49 years	1.665	2.631	0.891
2000	Spain	50-69 years	4.298	7.298	2.246
2000	Spain	70+ years	4.800	8.061	2.521
2000	Spain	All Ages	2.348	3.524	1.482
2005	Spain	Under 5	0.018	0.041	0.006
2005	Spain	5-14 years	0.090	0.194	0.035
2005	Spain	15-49 years	1.603	2.573	0.883
2005	Spain	50-69 years	3.981	6.896	1.999

2005	Spain	70+ years	4.353	7.759	2.084
2005	Spain	All Ages	2.211	3.341	1.377
2010	Spain	Under 5	0.019	0.042	0.007
2010	Spain	5-14 years	0.089	0.190	0.035
2010	Spain	15-49 years	1.479	2.434	0.788
2010	Spain	50-69 years	3.681	6.298	1.902
2010	Spain	70+ years	3.872	6.756	1.980
2010	Spain	All Ages	2.048	3.070	1.269
2015	Spain	Under 5	0.019	0.044	0.007
2015	Spain	5-14 years	0.089	0.194	0.034
2015	Spain	15-49 years	1.304	2.206	0.686
2015	Spain	50-69 years	3.476	6.132	1.713
2015	Spain	70+ years	3.422	5.783	1.722
2015	Spain	All Ages	1.924	2.949	1.166
2019	Spain	Under 5	0.020	0.045	0.007
2019	Spain	5-14 years	0.097	0.210	0.038
2019	Spain	15-49 years	1.247	2.059	0.659
2019	Spain	50-69 years	3.323	5.811	1.618
2019	Spain	70+ years	3.281	5.438	1.659
2019	Spain	All Ages	1.906	2.902	1.169

Table S60- Rates and 95% uncertainty levels (UL) of **disability-adjusted life years** (DALYs) **due to cirrhosis and other chronic liver diseases due to hepatitis C** per 100,000 population in Greece, Italy, Portugal and Spain from 2000 to 2019 by age group (Global Burden of Disease Study 2019).

Year	Country	Age	DALYs (95% UL)	95% UL (upper)	95% UL (lower)
2000	Greece	Under 5	0.066	0.128	0.029
2000	Greece	5-14 years	0.138	0.235	0.073
2000	Greece	15-49 years	20.97	30.80	13.69
2000	Greece	50-69 years	85.76	137.6	51.58
2000	Greece	70+ years	141.9	211.9	87.79
2000	Greece	All Ages	45.45	61.55	33.16
2005	Greece	Under 5	0.056	0.107	0.025
2005	Greece	5-14 years	0.138	0.238	0.074
2005	Greece	15-49 years	23.04	34.09	15.08
2005	Greece	50-69 years	81.37	128.3	49.64
2005	Greece	70+ years	108.9	163.9	67.47
2005	Greece	All Ages	43.56	59.49	31.41
2010	Greece	Under 5	0.056	0.103	0.026
2010	Greece	5-14 years	0.119	0.203	0.064
2010	Greece	15-49 years	26.59	38.71	17.48
2010	Greece	50-69 years	86.50	135.7	53.62
2010	Greece	70+ years	94.87	146.2	57.73
2010	Greece	All Ages	46.18	62.73	34.17
2015	Greece	Under 5	0.053	0.101	0.023
2015	Greece	5-14 years	0.100	0.168	0.053
2015	Greece	15-49 years	25.66	37.33	16.61
2015	Greece	50-69 years	89.63	142.4	54.42
2015	Greece	70+ years	86.62	133.0	52.66
2015	Greece	All Ages	47.33	64.80	34.44
2019	Greece	Under 5	0.045	0.088	0.021
2019	Greece	5-14 years	0.105	0.180	0.055
2019	Greece	15-49 years	26.27	38.49	16.79
2019	Greece	50-69 years	85.91	135.7	52.66
2019	Greece	70+ years	91.83	137.8	56.74
2019	Greece	All Ages	48.96	66.45	35.92
2000	Italy	Under 5	0.204	0.327	0.120
2000	Italy	5-14 years	0.459	0.635	0.324
2000	Italy	15-49 years	95.75	105.5	86.59
2000	Italy	50-69 years	367.4	422.3	317.9
2000	Italy	70+ years	480.6	564.5	396.0
2000	Italy	All Ages	196.9	217.8	178.5
2005	Italy	Under 5	0.143	0.236	0.084
2005	Italy	5-14 years	0.337	0.465	0.236
2005	Italy	15-49 years	80.18	90.75	70.35
2005	Italy	50-69 years	285.9	338.2	242.6
2005	Italy	70+ years	407.6	489.9	329.1
2005	Italy	All Ages	163.7	183.7	146.8
2010	Italy	Under 5	0.110	0.173	0.068
2010	Italy	5-14 years	0.279	0.387	0.195
2010	Italy	15-49 years	71.63	82.51	61.35
2010	Italy	50-69 years	250.8	301.0	208.3
2010	Italy	70+ years	361.3	435.8	289.0

2010	Italy	All Ages	148.3	167.3	131.1
2015	Italy	Under 5	0.088	0.140	0.054
2015	Italy	5-14 years	0.234	0.330	0.158
2015	Italy	15-49 years	61.64	71.37	52.96
2015	Italy	50-69 years	227.8	270.6	190.5
2015	Italy	70+ years	316.5	382.4	251.7
2015	Italy	All Ages	137.0	155.3	121.1
2019	Italy	Under 5	0.075	0.115	0.046
2019	Italy	5-14 years	0.248	0.358	0.165
2019	Italy	15-49 years	58.96	68.07	49.99
2019	Italy	50-69 years	217.1	259.1	179.5
2019	Italy	70+ years	307.0	370.3	247.0
2019	Italy	All Ages	137.3	155.5	120.9
2000	Portugal	Under 5	0.125	0.259	0.051
2000	Portugal	5-14 years	0.364	0.699	0.175
2000	Portugal	15-49 years	108.0	157.1	70.79
2000	Portugal	50-69 years	266.5	422.0	161.5
2000	Portugal	70+ years	199.4	303.9	124.8
2000	Portugal	All Ages	135.0	183.3	96.87
2005	Portugal	Under 5	0.077	0.164	0.030
2005	Portugal	5-14 years	0.258	0.481	0.125
2005	Portugal	15-49 years	96.22	141.0	61.65
2005	Portugal	50-69 years	229.3	362.1	138.7
2005	Portugal	70+ years	159.4	245.1	99.01
2005	Portugal	All Ages	118.9	161.8	85.26
2010	Portugal	Under 5	0.038	0.073	0.018
2010	Portugal	5-14 years	0.157	0.281	0.079
2010	Portugal	15-49 years	81.46	119.7	52.85
2010	Portugal	50-69 years	203.8	319.8	125.6
2010	Portugal	70+ years	135.4	203.2	84.10
2010	Portugal	All Ages	105.6	144.2	76.72
2015	Portugal	Under 5	0.033	0.065	0.015
2015	Portugal	5-14 years	0.105	0.183	0.055
2015	Portugal	15-49 years	56.71	84.59	35.56
2015	Portugal	50-69 years	171.0	269.4	103.2
2015	Portugal	70+ years	112.5	172.1	70.82
2015	Portugal	All Ages	86.43	120.0	62.13
2019	Portugal	Under 5	0.027	0.052	0.012
2019	Portugal	5-14 years	0.108	0.186	0.057
2019	Portugal	15-49 years	61.46	91.66	39.02
2019	Portugal	50-69 years	165.3	262.8	101.4
2019	Portugal	70+ years	114.3	171.8	69.29
2019	Portugal	All Ages	89.80	125.7	63.51
2000	Spain	Under 5	0.154	0.297	0.070
2000	Spain	5-14 years	0.397	0.667	0.216
2000	Spain	15-49 years	83.23	110.9	59.44
2000	Spain	50-69 years	276.2	397.2	183.2
2000	Spain	70+ years	322.2	459.9	209.4
2000	Spain	All Ages	139.4	177.7	108.5
2005	Spain	Under 5	0.129	0.234	0.062
2005	Spain	5-14 years	0.324	0.536	0.179
2005	Spain	15-49 years	81.29	108.0	57.56
2005	Spain	50-69 years	264.7	380.4	174.8

2005	Spain	70+ years	292.4	413.6	186.9
2005	Spain	All Ages	133.5	168.7	102.5
2010	Spain	Under 5	0.104	0.195	0.051
2010	Spain	5-14 years	0.278	0.450	0.153
2010	Spain	15-49 years	63.39	85.37	44.23
2010	Spain	50-69 years	227.9	329.4	153.0
2010	Spain	70+ years	234.6	340.7	153.0
2010	Spain	All Ages	111.2	142.0	86.17
2015	Spain	Under 5	0.076	0.140	0.036
2015	Spain	5-14 years	0.229	0.380	0.125
2015	Spain	15-49 years	47.38	63.86	32.96
2015	Spain	50-69 years	206.5	295.0	138.4
2015	Spain	70+ years	211.7	304.4	138.4
2015	Spain	All Ages	100.5	130.1	77.95
2019	Spain	Under 5	0.072	0.135	0.035
2019	Spain	5-14 years	0.233	0.391	0.126
2019	Spain	15-49 years	50.69	69.57	34.65
2019	Spain	50-69 years	188.5	271.3	125.0
2019	Spain	70+ years	203.0	289.7	131.0
2019	Spain	All Ages	100.9	129.8	77.92

Table S61- Rates and 95% uncertainty levels (UL) of **prevalence of liver cancer due to hepatitis B** per 100,000 population in Greece, Italy, Portugal and Spain from 2000 to 2019 by age group (Global Burden of Disease Study 2019).

Year	Country	Age	Prevalence (95% UL)	95% UL (upper)	95% UL (lower)
2000	Greece	Under 5	0.0	0.0	0.0
2000	Greece	5-14 years	0.08	0.12	0.06
2000	Greece	15-49 years	0.66	0.81	0.51
2000	Greece	50-69 years	4.55	6.00	3.30
2000	Greece	70+ years	6.90	10.0	4.42
2000	Greece	All Ages	2.12	2.70	1.63
2005	Greece	Under 5	0.0	0.0	0.0
2005	Greece	5-14 years	0.10	0.13	0.07
2005	Greece	15-49 years	0.81	1.01	0.63
2005	Greece	50-69 years	5.00	6.61	3.60
2005	Greece	70+ years	7.11	10.1	4.47
2005	Greece	All Ages	2.43	3.07	1.88
2010	Greece	Under 5	0.0	0.0	0.0
2010	Greece	5-14 years	0.10	0.14	0.06
2010	Greece	15-49 years	1.00	1.27	0.74
2010	Greece	50-69 years	5.65	7.50	4.02
2010	Greece	70+ years	7.62	11.0	4.81
2010	Greece	All Ages	2.87	3.65	2.20
2015	Greece	Under 5	0.0	0.0	0.0
2015	Greece	5-14 years	0.09	0.12	0.06
2015	Greece	15-49 years	1.18	1.49	0.88
2015	Greece	50-69 years	7.14	9.54	5.02
2015	Greece	70+ years	8.28	12.1	5.28
2015	Greece	All Ages	3.60	4.62	2.71
2019	Greece	Under 5	0.0	0.0	0.0
2019	Greece	5-14 years	0.09	0.13	0.06
2019	Greece	15-49 years	1.19	1.68	0.82
2019	Greece	50-69 years	6.62	9.84	4.24
2019	Greece	70+ years	8.02	12.0	4.85
2019	Greece	All Ages	3.58	5.00	2.46
2000	Italy	Under 5	0.0	0.0	0.0
2000	Italy	5-14 years	0.11	0.16	0.07
2000	Italy	15-49 years	1.14	1.41	0.91
2000	Italy	50-69 years	5.68	7.09	4.40
2000	Italy	70+ years	4.29	5.55	3.26
2000	Italy	All Ages	2.48	2.89	2.09
2005	Italy	Under 5	0.0	0.0	0.0
2005	Italy	5-14 years	0.12	0.18	0.08
2005	Italy	15-49 years	1.24	1.51	1.01
2005	Italy	50-69 years	4.68	5.91	3.63
2005	Italy	70+ years	3.64	4.73	2.75
2005	Italy	All Ages	2.23	2.61	1.87
2010	Italy	Under 5	0.0	0.0	0.0
2010	Italy	5-14 years	0.12	0.19	0.08
2010	Italy	15-49 years	1.35	1.66	1.10
2010	Italy	50-69 years	4.69	5.91	3.68
2010	Italy	70+ years	3.74	4.81	2.87

2010	Italy	All Ages	2.34	2.74	1.99
2015	Italy	Under 5	0.0	0.0	0.0
2015	Italy	5-14 years	0.13	0.20	0.08
2015	Italy	15-49 years	1.42	1.74	1.14
2015	Italy	50-69 years	5.09	6.39	4.00
2015	Italy	70+ years	3.67	4.75	2.77
2015	Italy	All Ages	2.55	3.00	2.16
2019	Italy	Under 5	0.0	0.0	0.0
2019	Italy	5-14 years	0.13	0.21	0.08
2019	Italy	15-49 years	1.37	1.85	1.01
2019	Italy	50-69 years	5.24	7.35	3.52
2019	Italy	70+ years	3.80	5.11	2.68
2019	Italy	All Ages	2.69	3.55	1.99
2000	Portugal	Under 5	0.0	0.0	0.0
2000	Portugal	5-14 years	0.05	0.07	0.03
2000	Portugal	15-49 years	0.50	0.69	0.35
2000	Portugal	50-69 years	2.02	3.23	1.20
2000	Portugal	70+ years	1.84	3.11	1.03
2000	Portugal	All Ages	0.90	1.25	0.64
2005	Portugal	Under 5	0.0	0.0	0.0
2005	Portugal	5-14 years	0.05	0.08	0.03
2005	Portugal	15-49 years	0.66	0.94	0.43
2005	Portugal	50-69 years	2.37	3.72	1.42
2005	Portugal	70+ years	1.84	3.09	1.03
2005	Portugal	All Ages	1.09	1.54	0.75
2010	Portugal	Under 5	0.0	0.0	0.0
2010	Portugal	5-14 years	0.05	0.08	0.03
2010	Portugal	15-49 years	0.82	1.17	0.53
2010	Portugal	50-69 years	3.09	4.87	1.90
2010	Portugal	70+ years	2.16	3.59	1.23
2010	Portugal	All Ages	1.42	1.99	0.99
2015	Portugal	Under 5	0.0	0.0	0.0
2015	Portugal	5-14 years	0.05	0.08	0.03
2015	Portugal	15-49 years	0.84	1.22	0.54
2015	Portugal	50-69 years	3.70	5.86	2.24
2015	Portugal	70+ years	2.34	4.01	1.33
2015	Portugal	All Ages	1.68	2.35	1.16
2019	Portugal	Under 5	0.0	0.0	0.0
2019	Portugal	5-14 years	0.06	0.10	0.03
2019	Portugal	15-49 years	0.94	1.52	0.54
2019	Portugal	50-69 years	3.65	6.28	2.05
2019	Portugal	70+ years	2.36	3.94	1.27
2019	Portugal	All Ages	1.78	2.77	1.10
2000	Spain	Under 5	0.0	0.0	0.0
2000	Spain	5-14 years	0.07	0.11	0.04
2000	Spain	15-49 years	0.74	1.03	0.50
2000	Spain	50-69 years	3.83	6.02	2.32
2000	Spain	70+ years	3.40	5.75	1.87
2000	Spain	All Ages	1.60	2.22	1.12
2005	Spain	Under 5	0.0	0.0	0.0
2005	Spain	5-14 years	0.08	0.12	0.04
2005	Spain	15-49 years	1.16	1.66	0.75
2005	Spain	50-69 years	4.56	7.33	2.74

2005	Spain	70+ years	3.65	6.17	2.01
2005	Spain	All Ages	2.01	2.77	1.43
2010	Spain	Under 5	0.0	0.0	0.0
2010	Spain	5-14 years	0.08	0.14	0.05
2010	Spain	15-49 years	1.33	1.96	0.84
2010	Spain	50-69 years	4.66	7.42	2.80
2010	Spain	70+ years	3.31	5.70	1.85
2010	Spain	All Ages	2.12	2.94	1.49
2015	Spain	Under 5	0.0	0.0	0.0
2015	Spain	5-14 years	0.08	0.14	0.04
2015	Spain	15-49 years	1.22	1.85	0.74
2015	Spain	50-69 years	5.12	8.24	3.07
2015	Spain	70+ years	3.28	5.56	1.85
2015	Spain	All Ages	2.26	3.20	1.57
2019	Spain	Under 5	0.0	0.0	0.0
2019	Spain	5-14 years	0.09	0.15	0.05
2019	Spain	15-49 years	1.27	2.08	0.70
2019	Spain	50-69 years	4.59	7.90	2.52
2019	Spain	70+ years	3.08	5.34	1.62
2019	Spain	All Ages	2.22	3.37	1.40

Table S62- Rates and 95% uncertainty levels (UL) of **incidence of liver cancer due to hepatitis B** per 100,000 population in Greece, Italy, Portugal and Spain from 2000 to 2019 by age group (Global Burden of Disease Study 2019).

Year	Country	Age	Incidence (95% UL)	95% UL (upper)	95% UL (lower)
2000	Greece	Under 5	0.0	0.0	0.0
2000	Greece	5-14 years	0.03	0.04	0.02
2000	Greece	15-49 years	0.30	0.36	0.24
2000	Greece	50-69 years	3.20	4.25	2.31
2000	Greece	70+ years	6.74	9.66	4.38
2000	Greece	All Ages	1.61	2.06	1.21
2005	Greece	Under 5	0.0	0.0	0.0
2005	Greece	5-14 years	0.03	0.04	0.02
2005	Greece	15-49 years	0.34	0.42	0.27
2005	Greece	50-69 years	3.32	4.38	2.38
2005	Greece	70+ years	6.67	9.57	4.23
2005	Greece	All Ages	1.76	2.26	1.32
2010	Greece	Under 5	0.0	0.0	0.0
2010	Greece	5-14 years	0.03	0.04	0.02
2010	Greece	15-49 years	0.41	0.51	0.31
2010	Greece	50-69 years	3.63	4.81	2.59
2010	Greece	70+ years	7.01	10.2	4.47
2010	Greece	All Ages	2.03	2.63	1.52
2015	Greece	Under 5	0.0	0.0	0.0
2015	Greece	5-14 years	0.03	0.04	0.02
2015	Greece	15-49 years	0.50	0.62	0.38
2015	Greece	50-69 years	4.65	6.15	3.26
2015	Greece	70+ years	7.81	11.3	5.04
2015	Greece	All Ages	2.60	3.37	1.92
2019	Greece	Under 5	0.0	0.0	0.0
2019	Greece	5-14 years	0.03	0.04	0.02
2019	Greece	15-49 years	0.50	0.70	0.35
2019	Greece	50-69 years	4.30	6.34	2.80
2019	Greece	70+ years	7.62	11.4	4.66
2019	Greece	All Ages	2.60	3.68	1.77
2000	Italy	Under 5	0.0	0.0	0.0
2000	Italy	5-14 years	0.03	0.04	0.02
2000	Italy	15-49 years	0.42	0.50	0.35
2000	Italy	50-69 years	3.34	4.16	2.67
2000	Italy	70+ years	3.87	4.98	2.96
2000	Italy	All Ages	1.51	1.77	1.27
2005	Italy	Under 5	0.0	0.0	0.0
2005	Italy	5-14 years	0.03	0.05	0.02
2005	Italy	15-49 years	0.41	0.49	0.34
2005	Italy	50-69 years	2.40	3.00	1.90
2005	Italy	70+ years	3.04	3.91	2.35
2005	Italy	All Ages	1.20	1.41	1.01
2010	Italy	Under 5	0.0	0.0	0.0
2010	Italy	5-14 years	0.03	0.05	0.02
2010	Italy	15-49 years	0.43	0.52	0.36
2010	Italy	50-69 years	2.27	2.82	1.79
2010	Italy	70+ years	3.09	3.92	2.42

2010	Italy	All Ages	1.22	1.44	1.04
2015	Italy	Under 5	0.0	0.0	0.0
2015	Italy	5-14 years	0.03	0.05	0.02
2015	Italy	15-49 years	0.45	0.54	0.37
2015	Italy	50-69 years	2.40	2.99	1.94
2015	Italy	70+ years	3.04	3.89	2.36
2015	Italy	All Ages	1.31	1.54	1.11
2019	Italy	Under 5	0.0	0.0	0.0
2019	Italy	5-14 years	0.03	0.05	0.02
2019	Italy	15-49 years	0.43	0.58	0.32
2019	Italy	50-69 years	2.48	3.46	1.71
2019	Italy	70+ years	3.12	4.18	2.26
2019	Italy	All Ages	1.40	1.82	1.04
2000	Portugal	Under 5	0.0	0.0	0.0
2000	Portugal	5-14 years	0.02	0.04	0.01
2000	Portugal	15-49 years	0.31	0.43	0.21
2000	Portugal	50-69 years	1.65	2.64	0.97
2000	Portugal	70+ years	2.06	3.48	1.16
2000	Portugal	All Ages	0.74	1.04	0.50
2005	Portugal	Under 5	0.0	0.0	0.0
2005	Portugal	5-14 years	0.02	0.03	0.01
2005	Portugal	15-49 years	0.37	0.52	0.24
2005	Portugal	50-69 years	1.81	2.86	1.09
2005	Portugal	70+ years	2.02	3.36	1.13
2005	Portugal	All Ages	0.84	1.20	0.57
2010	Portugal	Under 5	0.0	0.0	0.0
2010	Portugal	5-14 years	0.02	0.03	0.01
2010	Portugal	15-49 years	0.43	0.62	0.27
2010	Portugal	50-69 years	2.27	3.58	1.40
2010	Portugal	70+ years	2.33	3.89	1.34
2010	Portugal	All Ages	1.06	1.49	0.74
2015	Portugal	Under 5	0.0	0.0	0.0
2015	Portugal	5-14 years	0.02	0.03	0.01
2015	Portugal	15-49 years	0.41	0.60	0.26
2015	Portugal	50-69 years	2.62	4.13	1.57
2015	Portugal	70+ years	2.48	4.21	1.42
2015	Portugal	All Ages	1.23	1.75	0.84
2019	Portugal	Under 5	0.0	0.0	0.0
2019	Portugal	5-14 years	0.02	0.03	0.01
2019	Portugal	15-49 years	0.45	0.72	0.25
2019	Portugal	50-69 years	2.56	4.34	1.41
2019	Portugal	70+ years	2.49	4.17	1.35
2019	Portugal	All Ages	1.28	1.99	0.78
2000	Spain	Under 5	0.0	0.0	0.0
2000	Spain	5-14 years	0.02	0.04	0.01
2000	Spain	15-49 years	0.33	0.46	0.23
2000	Spain	50-69 years	2.42	3.78	1.46
2000	Spain	70+ years	3.21	5.34	1.79
2000	Spain	All Ages	1.06	1.51	0.73
2005	Spain	Under 5	0.0	0.0	0.0
2005	Spain	5-14 years	0.02	0.04	0.01
2005	Spain	15-49 years	0.45	0.64	0.29
2005	Spain	50-69 years	2.50	3.90	1.51

2005	Spain	70+ years	3.21	5.42	1.79
2005	Spain	All Ages	1.15	1.62	0.81
2010	Spain	Under 5	0.0	0.0	0.0
2010	Spain	5-14 years	0.02	0.04	0.01
2010	Spain	15-49 years	0.48	0.70	0.30
2010	Spain	50-69 years	2.48	3.93	1.47
2010	Spain	70+ years	2.95	5.00	1.67
2010	Spain	All Ages	1.15	1.61	0.80
2015	Spain	Under 5	0.0	0.0	0.0
2015	Spain	5-14 years	0.02	0.03	0.01
2015	Spain	15-49 years	0.42	0.63	0.27
2015	Spain	50-69 years	2.76	4.40	1.62
2015	Spain	70+ years	2.98	5.05	1.71
2015	Spain	All Ages	1.27	1.82	0.87
2019	Spain	Under 5	0.0	0.0	0.0
2019	Spain	5-14 years	0.02	0.04	0.01
2019	Spain	15-49 years	0.44	0.72	0.24
2019	Spain	50-69 years	2.51	4.37	1.38
2019	Spain	70+ years	2.81	4.86	1.49
2019	Spain	All Ages	1.25	1.95	0.78

Table S63- Rates and 95% uncertainty levels (UL) of **deaths due to liver cancer due to hepatitis B** per 100,000 population in Greece, Italy, Portugal and Spain from 2000 to 2019 by age group (Global Burden of Disease Study 2019).

Year	Country	Age	Deaths (95% UL)	95% UL (upper)	95% UL (lower)
2000	Greece	5-14 years	0.007	0.009	0.005
2000	Greece	15-49 years	0.198	0.240	0.157
2000	Greece	50-69 years	2.757	3.691	1.958
2000	Greece	70+ years	7.191	10.29	4.721
2000	Greece	All Ages	1.499	1.948	1.117
2005	Greece	5-14 years	0.007	0.009	0.005
2005	Greece	15-49 years	0.212	0.259	0.167
2005	Greece	50-69 years	2.761	3.619	1.989
2005	Greece	70+ years	6.946	9.886	4.429
2005	Greece	All Ages	1.598	2.091	1.172
2010	Greece	5-14 years	0.006	0.009	0.004
2010	Greece	15-49 years	0.246	0.306	0.187
2010	Greece	50-69 years	2.987	3.970	2.140
2010	Greece	70+ years	7.311	10.60	4.715
2010	Greece	All Ages	1.845	2.411	1.365
2015	Greece	5-14 years	0.006	0.009	0.004
2015	Greece	15-49 years	0.308	0.387	0.235
2015	Greece	50-69 years	3.831	5.098	2.706
2015	Greece	70+ years	8.262	12.05	5.374
2015	Greece	All Ages	2.376	3.116	1.738
2019	Greece	5-14 years	0.006	0.009	0.004
2019	Greece	15-49 years	0.309	0.389	0.234
2019	Greece	50-69 years	3.551	4.782	2.477
2019	Greece	70+ years	8.135	11.90	5.255
2019	Greece	All Ages	2.409	3.224	1.764
2000	Italy	5-14 years	0.009	0.010	0.007
2000	Italy	15-49 years	0.229	0.270	0.193
2000	Italy	50-69 years	2.609	3.237	2.084
2000	Italy	70+ years	3.960	5.056	3.053
2000	Italy	All Ages	1.249	1.479	1.042
2005	Italy	5-14 years	0.006	0.007	0.005
2005	Italy	15-49 years	0.200	0.238	0.168
2005	Italy	50-69 years	1.723	2.167	1.367
2005	Italy	70+ years	3.014	3.852	2.338
2005	Italy	All Ages	0.929	1.099	0.775
2010	Italy	5-14 years	0.005	0.006	0.004
2010	Italy	15-49 years	0.209	0.251	0.174
2010	Italy	50-69 years	1.579	1.981	1.241
2010	Italy	70+ years	3.075	3.915	2.421
2010	Italy	All Ages	0.941	1.118	0.799
2015	Italy	5-14 years	0.005	0.006	0.004
2015	Italy	15-49 years	0.210	0.254	0.174
2015	Italy	50-69 years	1.615	2.017	1.283
2015	Italy	70+ years	2.999	3.838	2.331
2015	Italy	All Ages	0.991	1.172	0.836
2019	Italy	5-14 years	0.005	0.006	0.004
2019	Italy	15-49 years	0.203	0.254	0.161
2019	Italy	50-69 years	1.690	2.180	1.297

2019	Italy	70+ years	3.107	4.043	2.378
2019	Italy	All Ages	1.084	1.324	0.888
2000	Portugal	5-14 years	0.012	0.020	0.007
2000	Portugal	15-49 years	0.242	0.338	0.163
2000	Portugal	50-69 years	1.546	2.464	0.916
2000	Portugal	70+ years	2.367	3.970	1.336
2000	Portugal	All Ages	0.718	1.020	0.485
2005	Portugal	5-14 years	0.010	0.016	0.006
2005	Portugal	15-49 years	0.275	0.394	0.181
2005	Portugal	50-69 years	1.655	2.594	0.973
2005	Portugal	70+ years	2.318	3.921	1.312
2005	Portugal	All Ages	0.792	1.125	0.539
2010	Portugal	5-14 years	0.009	0.015	0.005
2010	Portugal	15-49 years	0.310	0.449	0.197
2010	Portugal	50-69 years	2.003	3.175	1.226
2010	Portugal	70+ years	2.638	4.447	1.508
2010	Portugal	All Ages	0.982	1.393	0.667
2015	Portugal	5-14 years	0.008	0.013	0.004
2015	Portugal	15-49 years	0.289	0.419	0.182
2015	Portugal	50-69 years	2.303	3.648	1.375
2015	Portugal	70+ years	2.829	4.783	1.623
2015	Portugal	All Ages	1.141	1.642	0.779
2019	Portugal	5-14 years	0.008	0.015	0.004
2019	Portugal	15-49 years	0.305	0.460	0.186
2019	Portugal	50-69 years	2.198	3.533	1.313
2019	Portugal	70+ years	2.793	4.726	1.607
2019	Portugal	All Ages	1.173	1.719	0.790
2000	Spain	5-14 years	0.009	0.014	0.005
2000	Spain	15-49 years	0.216	0.299	0.148
2000	Spain	50-69 years	1.972	3.080	1.169
2000	Spain	70+ years	3.366	5.644	1.891
2000	Spain	All Ages	0.922	1.332	0.624
2005	Spain	5-14 years	0.008	0.012	0.004
2005	Spain	15-49 years	0.256	0.365	0.166
2005	Spain	50-69 years	1.853	2.916	1.113
2005	Spain	70+ years	3.245	5.444	1.845
2005	Spain	All Ages	0.918	1.329	0.626
2010	Spain	5-14 years	0.007	0.011	0.004
2010	Spain	15-49 years	0.257	0.375	0.161
2010	Spain	50-69 years	1.817	2.891	1.077
2010	Spain	70+ years	3.039	5.166	1.719
2010	Spain	All Ages	0.905	1.297	0.621
2015	Spain	5-14 years	0.005	0.010	0.003
2015	Spain	15-49 years	0.222	0.331	0.141
2015	Spain	50-69 years	2.026	3.196	1.180
2015	Spain	70+ years	3.085	5.200	1.770
2015	Spain	All Ages	1.006	1.460	0.682
2019	Spain	5-14 years	0.006	0.011	0.003
2019	Spain	15-49 years	0.229	0.350	0.140
2019	Spain	50-69 years	1.872	3.005	1.081
2019	Spain	70+ years	2.953	4.973	1.687
2019	Spain	All Ages	1.015	1.488	0.673

Table S64- Rates and 95% uncertainty levels (UL) of **years of life lost** (YLLs) **due to liver cancer due to hepatitis B** per 100,000 population in Greece, Italy, Portugal and Spain from 2000 to 2019 by age group (Global Burden of Disease Study 2019).

Year	Country	Age	YLLs (95% UL)	95% UL (upper)	95% UL (lower)
2000	Greece	5-14 years	0.54	0.72	0.40
2000	Greece	15-49 years	9.46	11.3	7.60
2000	Greece	50-69 years	78.1	103.6	56.7
2000	Greece	70+ years	104.3	151.6	66.9
2000	Greece	All Ages	33.8	43.2	26.0
2005	Greece	5-14 years	0.55	0.74	0.40
2005	Greece	15-49 years	10.0	12.1	8.04
2005	Greece	50-69 years	79.5	104.1	57.3
2005	Greece	70+ years	100.2	143.5	64.4
2005	Greece	All Ages	35.6	45.2	27.5
2010	Greece	5-14 years	0.51	0.71	0.36
2010	Greece	15-49 years	11.5	14.2	8.97
2010	Greece	50-69 years	86.6	113.	62.9
2010	Greece	70+ years	102.5	149.6	65.4
2010	Greece	All Ages	40.1	50.8	30.7
2015	Greece	5-14 years	0.49	0.69	0.34
2015	Greece	15-49 years	14.1	17.5	10.9
2015	Greece	50-69 years	110.2	145.4	78.0
2015	Greece	70+ years	111.0	162.5	70.3
2015	Greece	All Ages	51.0	65.2	39.2
2019	Greece	5-14 years	0.51	0.71	0.35
2019	Greece	15-49 years	14.2	17.7	10.9
2019	Greece	50-69 years	102.3	137.4	71.8
2019	Greece	70+ years	107.7	155.4	69.6
2019	Greece	All Ages	50.7	65.9	38.0
2000	Italy	5-14 years	0.69	0.83	0.57
2000	Italy	15-49 years	10.9	12.8	9.33
2000	Italy	50-69 years	74.5	92.2	59.7
2000	Italy	70+ years	61.0	78.9	46.5
2000	Italy	All Ages	31.2	36.7	26.3
2005	Italy	5-14 years	0.50	0.60	0.42
2005	Italy	15-49 years	9.53	11.2	8.05
2005	Italy	50-69 years	49.3	61.5	39.2
2005	Italy	70+ years	44.8	57.6	34.0
2005	Italy	All Ages	22.6	26.6	19.1
2010	Italy	5-14 years	0.44	0.53	0.36
2010	Italy	15-49 years	9.78	11.6	8.22
2010	Italy	50-69 years	45.8	57.1	36.3
2010	Italy	70+ years	44.2	56.5	34.6
2010	Italy	All Ages	22.3	26.0	19.1
2015	Italy	5-14 years	0.40	0.49	0.32
2015	Italy	15-49 years	9.75	11.6	8.12
2015	Italy	50-69 years	47.5	59.0	37.8
2015	Italy	70+ years	42.1	53.3	32.7
2015	Italy	All Ages	23.4	27.3	19.9
2019	Italy	5-14 years	0.41	0.52	0.32
2019	Italy	15-49 years	9.44	11.6	7.52

2019	Italy	50-69 years	49.6	64.0	37.9
2019	Italy	70+ years	43.9	57.7	33.2
2019	Italy	All Ages	25.2	30.6	20.8
2000	Portugal	5-14 years	0.95	1.56	0.56
2000	Portugal	15-49 years	11.8	16.2	8.09
2000	Portugal	50-69 years	44.9	71.2	26.8
2000	Portugal	70+ years	36.5	60.8	20.5
2000	Portugal	All Ages	19.9	27.4	14.1
2005	Portugal	5-14 years	0.81	1.27	0.47
2005	Portugal	15-49 years	13.1	18.4	8.79
2005	Portugal	50-69 years	49.1	77.0	29.5
2005	Portugal	70+ years	35.1	59.5	19.1
2005	Portugal	All Ages	21.9	30.3	15.4
2010	Portugal	5-14 years	0.72	1.19	0.40
2010	Portugal	15-49 years	14.6	20.8	9.51
2010	Portugal	50-69 years	60.5	96.5	37.1
2010	Portugal	70+ years	39.2	66.0	22.2
2010	Portugal	All Ages	26.7	37.7	18.3
2015	Portugal	5-14 years	0.61	1.00	0.32
2015	Portugal	15-49 years	13.4	19.2	8.65
2015	Portugal	50-69 years	69.4	109.0	41.4
2015	Portugal	70+ years	41.0	69.8	23.3
2015	Portugal	All Ages	30.1	43.0	20.6
2019	Portugal	5-14 years	0.67	1.20	0.35
2019	Portugal	15-49 years	14.3	21.1	8.96
2019	Portugal	50-69 years	65.7	105.2	39.1
2019	Portugal	70+ years	40.3	68.5	22.7
2019	Portugal	All Ages	30.4	44.6	20.6
2000	Spain	5-14 years	0.74	1.13	0.43
2000	Spain	15-49 years	10.5	14.4	7.34
2000	Spain	50-69 years	56.4	87.4	33.8
2000	Spain	70+ years	51.1	85.3	28.3
2000	Spain	All Ages	23.4	32.2	16.5
2005	Spain	5-14 years	0.63	0.98	0.36
2005	Spain	15-49 years	12.1	17.0	8.07
2005	Spain	50-69 years	54.6	85.5	33.4
2005	Spain	70+ years	48.2	82.1	26.6
2005	Spain	All Ages	23.6	33.0	16.9
2010	Spain	5-14 years	0.54	0.86	0.31
2010	Spain	15-49 years	11.9	17.0	7.70
2010	Spain	50-69 years	54.3	85.1	32.7
2010	Spain	70+ years	43.0	72.9	23.9
2010	Spain	All Ages	23.3	32.6	16.3
2015	Spain	5-14 years	0.45	0.77	0.24
2015	Spain	15-49 years	10.3	14.9	6.58
2015	Spain	50-69 years	60.8	96.2	35.6
2015	Spain	70+ years	42.9	72.4	24.4
2015	Spain	All Ages	25.3	35.9	17.5
2019	Spain	5-14 years	0.49	0.89	0.25
2019	Spain	15-49 years	10.6	15.9	6.65
2019	Spain	50-69 years	56.0	90.3	32.5
2019	Spain	70+ years	40.9	69.7	22.7
2019	Spain	All Ages	25.3	36.2	17.0

Table S65- Rates and 95% uncertainty levels (UL) of **years lived with disability** (YLDs) **due to liver cancer due to hepatitis B** per 100,000 population in Greece, Italy, Portugal and Spain from 2000 to 2019 by age group (Global Burden of Disease Study 2019).

Year	Country	Age	YLDs 95% UL)	95% UL (upper)	95% UL (lower)
2000	Greece	Under 5	0.0	0.0	0.0
2000	Greece	5-14 years	0.009	0.014	0.006
2000	Greece	15-49 years	0.083	0.115	0.056
2000	Greece	50-69 years	0.780	1.124	0.490
2000	Greece	70+ years	1.536	2.367	0.870
2000	Greece	All Ages	0.386	0.553	0.249
2005	Greece	Under 5	0.0	0.0	0.0
2005	Greece	5-14 years	0.010	0.015	0.006
2005	Greece	15-49 years	0.098	0.135	0.064
2005	Greece	50-69 years	0.823	1.177	0.521
2005	Greece	70+ years	1.536	2.384	0.866
2005	Greece	All Ages	0.428	0.610	0.273
2010	Greece	Under 5	0.0	0.0	0.0
2010	Greece	5-14 years	0.010	0.015	0.006
2010	Greece	15-49 years	0.118	0.169	0.076
2010	Greece	50-69 years	0.908	1.340	0.564
2010	Greece	70+ years	1.623	2.558	0.911
2010	Greece	All Ages	0.497	0.713	0.317
2015	Greece	Under 5	0.0	0.0	0.0
2015	Greece	5-14 years	0.009	0.013	0.005
2015	Greece	15-49 years	0.143	0.202	0.093
2015	Greece	50-69 years	1.157	1.739	0.716
2015	Greece	70+ years	1.798	2.874	1.009
2015	Greece	All Ages	0.632	0.915	0.403
2019	Greece	Under 5	0.0	0.0	0.0
2019	Greece	5-14 years	0.010	0.014	0.005
2019	Greece	15-49 years	0.144	0.220	0.088
2019	Greece	50-69 years	1.072	1.722	0.628
2019	Greece	70+ years	1.751	2.892	0.963
2019	Greece	All Ages	0.633	0.977	0.373
2000	Italy	Under 5	0.0	0.0	0.0
2000	Italy	5-14 years	0.011	0.016	0.006
2000	Italy	15-49 years	0.126	0.177	0.087
2000	Italy	50-69 years	0.856	1.217	0.586
2000	Italy	70+ years	0.899	1.301	0.566
2000	Italy	All Ages	0.384	0.523	0.271
2005	Italy	Under 5	0.0	0.0	0.0
2005	Italy	5-14 years	0.011	0.018	0.007
2005	Italy	15-49 years	0.129	0.179	0.089
2005	Italy	50-69 years	0.646	0.925	0.438
2005	Italy	70+ years	0.722	1.034	0.455
2005	Italy	All Ages	0.318	0.434	0.222
2010	Italy	Under 5	0.0	0.0	0.0
2010	Italy	5-14 years	0.011	0.019	0.006
2010	Italy	15-49 years	0.139	0.196	0.095
2010	Italy	50-69 years	0.622	0.881	0.428

2010	Italy	70+ years	0.735	1.038	0.472
2010	Italy	All Ages	0.327	0.444	0.231
2015	Italy	Under 5	0.0	0.0	0.0
2015	Italy	5-14 years	0.011	0.019	0.006
2015	Italy	15-49 years	0.144	0.204	0.098
2015	Italy	50-69 years	0.662	0.939	0.451
2015	Italy	70+ years	0.724	1.038	0.459
2015	Italy	All Ages	0.353	0.481	0.244
2019	Italy	Under 5	0.0	0.0	0.0
2019	Italy	5-14 years	0.012	0.019	0.007
2019	Italy	15-49 years	0.139	0.213	0.088
2019	Italy	50-69 years	0.683	1.098	0.425
2019	Italy	70+ years	0.744	1.111	0.459
2019	Italy	All Ages	0.376	0.568	0.244
2000	Portugal	Under 5	0.0	0.0	0.0
2000	Portugal	5-14 years	0.006	0.011	0.003
2000	Portugal	15-49 years	0.078	0.121	0.047
2000	Portugal	50-69 years	0.386	0.645	0.211
2000	Portugal	70+ years	0.455	0.780	0.232
2000	Portugal	All Ages	0.174	0.265	0.106
2005	Portugal	Under 5	0.0	0.0	0.0
2005	Portugal	5-14 years	0.006	0.011	0.003
2005	Portugal	15-49 years	0.095	0.152	0.056
2005	Portugal	50-69 years	0.432	0.721	0.231
2005	Portugal	70+ years	0.448	0.786	0.228
2005	Portugal	All Ages	0.200	0.307	0.121
2010	Portugal	Under 5	0.0	0.0	0.0
2010	Portugal	5-14 years	0.006	0.010	0.003
2010	Portugal	15-49 years	0.114	0.184	0.066
2010	Portugal	50-69 years	0.547	0.950	0.296
2010	Portugal	70+ years	0.519	0.909	0.265
2010	Portugal	All Ages	0.255	0.391	0.153
2015	Portugal	Under 5	0.0	0.0	0.0
2015	Portugal	5-14 years	0.006	0.010	0.003
2015	Portugal	15-49 years	0.111	0.174	0.064
2015	Portugal	50-69 years	0.637	1.083	0.345
2015	Portugal	70+ years	0.554	0.976	0.290
2015	Portugal	All Ages	0.297	0.457	0.175
2019	Portugal	Under 5	0.0	0.0	0.0
2019	Portugal	5-14 years	0.007	0.012	0.003
2019	Portugal	15-49 years	0.122	0.213	0.066
2019	Portugal	50-69 years	0.624	1.134	0.312
2019	Portugal	70+ years	0.557	0.992	0.263
2019	Portugal	All Ages	0.311	0.526	0.173
2000	Spain	Under 5	0.0	0.0	0.0
2000	Spain	5-14 years	0.007	0.012	0.004
2000	Spain	15-49 years	0.093	0.141	0.057
2000	Spain	50-69 years	0.609	1.033	0.333
2000	Spain	70+ years	0.736	1.289	0.374
2000	Spain	All Ages	0.264	0.404	0.163
2005	Spain	Under 5	0.0	0.0	0.0
2005	Spain	5-14 years	0.008	0.013	0.004
2005	Spain	15-49 years	0.132	0.203	0.078

2005	Spain	50-69 years	0.657	1.129	0.354
2005	Spain	70+ years	0.750	1.303	0.372
2005	Spain	All Ages	0.298	0.457	0.185
2010	Spain	Under 5	0.0	0.0	0.0
2010	Spain	5-14 years	0.008	0.014	0.004
2010	Spain	15-49 years	0.146	0.236	0.082
2010	Spain	50-69 years	0.659	1.121	0.353
2010	Spain	70+ years	0.688	1.234	0.349
2010	Spain	All Ages	0.304	0.458	0.186
2015	Spain	Under 5	0.0	0.0	0.0
2015	Spain	5-14 years	0.008	0.013	0.004
2015	Spain	15-49 years	0.131	0.209	0.076
2015	Spain	50-69 years	0.728	1.237	0.398
2015	Spain	70+ years	0.692	1.205	0.351
2015	Spain	All Ages	0.331	0.513	0.200
2019	Spain	Under 5	0.0	0.0	0.0
2019	Spain	5-14 years	0.008	0.015	0.004
2019	Spain	15-49 years	0.136	0.235	0.073
2019	Spain	50-69 years	0.659	1.185	0.338
2019	Spain	70+ years	0.652	1.183	0.312
2019	Spain	All Ages	0.327	0.557	0.187

Note: Estimates of liver cancer due to HBV or HCV were not generated for the group under the age of 5 years.

Table S66- Rates and 95% uncertainty levels (UL) of **disability-adjusted life years** (DALYs) **due to liver cancer due to hepatitis B** per 100,000 population in Greece, Italy, Portugal and Spain from 2000 to 2019 by age group (Global Burden of Disease Study 2019).

Year	Country	Age	DALYs (95% UL)	95% UL (upper)	95% UL (lower)
2000	Greece	Under 5	0.0	0.0	0.0
2000	Greece	5-14 years	0.55	0.73	0.41
2000	Greece	15-49 years	9.55	11.4	7.67
2000	Greece	50-69 years	78.9	104.	57.2
2000	Greece	70+ years	105.	153.	68.0
2000	Greece	All Ages	34.2	43.7	26.3
2005	Greece	Under 5	0.0	0.0	0.0
2005	Greece	5-14 years	0.56	0.76	0.40
2005	Greece	15-49 years	10.1	12.2	8.12
2005	Greece	50-69 years	80.3	105.	57.9
2005	Greece	70+ years	101.	145.	65.3
2005	Greece	All Ages	36.0	45.6	27.8
2010	Greece	Under 5	0.0	0.0	0.0
2010	Greece	5-14 years	0.52	0.72	0.36
2010	Greece	15-49 years	11.6	14.4	9.04
2010	Greece	50-69 years	87.5	114.	63.4
2010	Greece	70+ years	104.	152.	66.3
2010	Greece	All Ages	40.6	51.4	31.1
2015	Greece	Under 5	0.0	0.0	0.0
2015	Greece	5-14 years	0.50	0.70	0.35
2015	Greece	15-49 years	14.3	17.6	11.0
2015	Greece	50-69 years	111.	146.	78.8
2015	Greece	70+ years	112.	165.	71.4
2015	Greece	All Ages	51.7	66.1	39.6
2019	Greece	Under 5	0.0	0.0	0.0
2019	Greece	5-14 years	0.52	0.72	0.36
2019	Greece	15-49 years	14.3	17.9	10.9
2019	Greece	50-69 years	103.	138.	72.4
2019	Greece	70+ years	109.	158.	70.9
2019	Greece	All Ages	51.3	66.8	38.4
2000	Italy	Under 5	0.0	0.0	0.0
2000	Italy	5-14 years	0.70	0.84	0.58
2000	Italy	15-49 years	11.1	13.0	9.44
2000	Italy	50-69 years	75.4	93.1	60.5
2000	Italy	70+ years	61.9	80.1	47.2
2000	Italy	All Ages	31.5	37.2	26.6
2005	Italy	Under 5	0.0	0.0	0.0
2005	Italy	5-14 years	0.51	0.61	0.43
2005	Italy	15-49 years	9.66	11.3	8.17
2005	Italy	50-69 years	49.9	62.2	39.6
2005	Italy	70+ years	45.5	58.5	34.5
2005	Italy	All Ages	23.0	26.9	19.3
2010	Italy	Under 5	0.0	0.0	0.0
2010	Italy	5-14 years	0.45	0.54	0.37
2010	Italy	15-49 years	9.92	11.8	8.34
2010	Italy	50-69 years	46.4	58.1	36.7
2010	Italy	70+ years	45.0	57.5	35.1

2010	Italy	All Ages	22.6	26.4	19.4
2015	Italy	Under 5	0.0	0.0	0.0
2015	Italy	5-14 years	0.41	0.51	0.33
2015	Italy	15-49 years	9.89	11.8	8.24
2015	Italy	50-69 years	48.1	59.9	38.4
2015	Italy	70+ years	42.8	54.2	33.3
2015	Italy	All Ages	23.7	27.7	20.2
2019	Italy	Under 5	0.0	0.0	0.0
2019	Italy	5-14 years	0.42	0.54	0.34
2019	Italy	15-49 years	9.58	11.7	7.63
2019	Italy	50-69 years	50.3	64.8	38.5
2019	Italy	70+ years	44.6	58.7	33.7
2019	Italy	All Ages	25.6	31.0	21.1
2000	Portugal	Under 5	0.0	0.0	0.0
2000	Portugal	5-14 years	0.95	1.57	0.56
2000	Portugal	15-49 years	11.9	16.3	8.15
2000	Portugal	50-69 years	45.3	71.9	27.0
2000	Portugal	70+ years	36.9	61.6	20.7
2000	Portugal	All Ages	20.1	27.6	14.2
2005	Portugal	Under 5	0.0	0.0	0.0
2005	Portugal	5-14 years	0.82	1.28	0.48
2005	Portugal	15-49 years	13.2	18.5	8.87
2005	Portugal	50-69 years	49.5	77.7	29.8
2005	Portugal	70+ years	35.6	60.2	19.3
2005	Portugal	All Ages	22.1	30.6	15.5
2010	Portugal	Under 5	0.0	0.0	0.0
2010	Portugal	5-14 years	0.73	1.20	0.41
2010	Portugal	15-49 years	14.7	20.9	9.59
2010	Portugal	50-69 years	61.1	97.4	37.4
2010	Portugal	70+ years	39.7	66.8	22.5
2010	Portugal	All Ages	27.0	38.1	18.5
2015	Portugal	Under 5	0.0	0.0	0.0
2015	Portugal	5-14 years	0.61	1.01	0.33
2015	Portugal	15-49 years	13.5	19.4	8.70
2015	Portugal	50-69 years	70.1	110.	41.8
2015	Portugal	70+ years	41.5	70.6	23.7
2015	Portugal	All Ages	30.4	43.4	20.8
2019	Portugal	Under 5	0.0	0.0	0.0
2019	Portugal	5-14 years	0.68	1.21	0.35
2019	Portugal	15-49 years	14.4	21.3	9.02
2019	Portugal	50-69 years	66.4	106.	39.4
2019	Portugal	70+ years	40.8	69.4	23.0
2019	Portugal	All Ages	30.8	45.1	20.8
2000	Spain	Under 5	0.0	0.0	0.0
2000	Spain	5-14 years	0.75	1.13	0.43
2000	Spain	15-49 years	10.6	14.5	7.40
2000	Spain	50-69 years	57.0	88.3	34.1
2000	Spain	70+ years	51.9	87.0	28.8
2000	Spain	All Ages	23.7	32.5	16.7
2005	Spain	Under 5	0.0	0.0	0.0
2005	Spain	5-14 years	0.64	0.99	0.37
2005	Spain	15-49 years	12.3	17.1	8.17
2005	Spain	50-69 years	55.2	86.5	33.9

2005	Spain	70+ years	49.0	83.2	27.0
2005	Spain	All Ages	23.9	33.4	17.1
2010	Spain	Under 5	0.0	0.0	0.0
2010	Spain	5-14 years	0.55	0.87	0.31
2010	Spain	15-49 years	12.1	17.3	7.80
2010	Spain	50-69 years	55.0	85.9	33.1
2010	Spain	70+ years	43.7	74.2	24.3
2010	Spain	All Ages	23.6	33.1	16.6
2015	Spain	Under 5	0.0	0.0	0.0
2015	Spain	5-14 years	0.45	0.79	0.25
2015	Spain	15-49 years	10.4	15.1	6.68
2015	Spain	50-69 years	61.5	97.2	36.1
2015	Spain	70+ years	43.6	73.7	24.8
2015	Spain	All Ages	25.6	36.3	17.7
2019	Spain	Under 5	0.0	0.0	0.0
2019	Spain	5-14 years	0.50	0.91	0.25
2019	Spain	15-49 years	10.8	16.1	6.75
2019	Spain	50-69 years	56.7	91.5	32.9
2019	Spain	70+ years	41.6	70.9	23.2
2019	Spain	All Ages	25.6	36.7	17.2

Note: Estimates of liver cancer due to HBV or HCV were not generated for the group under the age of 5 years.

Table S67- Rates and 95% uncertainty levels (UL) of **prevalence of liver cancer due to hepatitis C** per 100,000 population in Greece, Italy, Portugal and Spain from 2000 to 2019 by age group (Global Burden of Disease Study 2019).

Year	Country	Age	Prevalence (95% UL)	95% UL (upper)	95% UL (lower)
2000	Greece	Under 5	0.0	0.0	0.0
2000	Greece	5-14 years	0.000	0.002	0.000
2000	Greece	15-49 years	0.060	0.102	0.032
2000	Greece	50-69 years	1.168	1.812	0.672
2000	Greece	70+ years	5.216	7.190	3.413
2000	Greece	All Ages	0.854	1.144	0.592
2005	Greece	Under 5	0.0	0.0	0.0
2005	Greece	5-14 years	0.000	0.002	0.000
2005	Greece	15-49 years	0.078	0.136	0.041
2005	Greece	50-69 years	1.247	1.924	0.717
2005	Greece	70+ years	5.438	7.637	3.509
2005	Greece	All Ages	1.000	1.361	0.688
2010	Greece	Under 5	0.0	0.0	0.0
2010	Greece	5-14 years	0.001	0.002	0.000
2010	Greece	15-49 years	0.106	0.178	0.055
2010	Greece	50-69 years	1.541	2.404	0.896
2010	Greece	70+ years	6.433	9.076	4.085
2010	Greece	All Ages	1.320	1.790	0.906
2015	Greece	Under 5	0.0	0.0	0.0
2015	Greece	5-14 years	0.000	0.002	0.000
2015	Greece	15-49 years	0.137	0.230	0.071
2015	Greece	50-69 years	1.920	3.050	1.098
2015	Greece	70+ years	6.994	9.867	4.487
2015	Greece	All Ages	1.627	2.245	1.131
2019	Greece	Under 5	0.0	0.0	0.0
2019	Greece	5-14 years	0.001	0.002	0.000
2019	Greece	15-49 years	0.138	0.244	0.069
2019	Greece	50-69 years	1.813	3.175	0.986
2019	Greece	70+ years	7.013	10.68	4.338
2019	Greece	All Ages	1.696	2.501	1.104
2000	Italy	Under 5	0.0	0.0	0.0
2000	Italy	5-14 years	0.010	0.016	0.006
2000	Italy	15-49 years	1.160	1.446	0.911
2000	Italy	50-69 years	18.23	21.29	15.52
2000	Italy	70+ years	33.66	37.41	29.85
2000	Italy	All Ages	9.280	10.24	8.374
2005	Italy	Under 5	0.0	0.0	0.0
2005	Italy	5-14 years	0.011	0.018	0.006
2005	Italy	15-49 years	1.341	1.653	1.065
2005	Italy	50-69 years	16.97	19.66	14.43
2005	Italy	70+ years	31.04	34.77	27.04
2005	Italy	All Ages	9.035	9.900	8.087
2010	Italy	Under 5	0.0	0.0	0.0
2010	Italy	5-14 years	0.011	0.019	0.006
2010	Italy	15-49 years	1.587	1.938	1.245
2010	Italy	50-69 years	17.87	20.91	15.08
2010	Italy	70+ years	32.84	36.76	28.37

2010	Italy	All Ages	9.990	11.02	8.942
2015	Italy	Under 5	0.0	0.0	0.0
2015	Italy	5-14 years	0.012	0.020	0.007
2015	Italy	15-49 years	1.719	2.101	1.344
2015	Italy	50-69 years	18.41	21.89	15.38
2015	Italy	70+ years	32.42	36.90	27.71
2015	Italy	All Ages	10.71	11.93	9.550
2019	Italy	Under 5	0.0	0.0	0.0
2019	Italy	5-14 years	0.012	0.021	0.007
2019	Italy	15-49 years	1.617	2.175	1.123
2019	Italy	50-69 years	17.57	24.09	12.82
2019	Italy	70+ years	31.80	39.17	24.78
2019	Italy	All Ages	10.97	13.89	8.632
2000	Portugal	Under 5	0.0	0.0	0.0
2000	Portugal	5-14 years	0.003	0.007	0.000
2000	Portugal	15-49 years	0.250	0.391	0.147
2000	Portugal	50-69 years	3.843	5.568	2.424
2000	Portugal	70+ years	8.702	11.31	6.067
2000	Portugal	All Ages	1.904	2.478	1.410
2005	Portugal	Under 5	0.0	0.0	0.0
2005	Portugal	5-14 years	0.003	0.007	0.000
2005	Portugal	15-49 years	0.349	0.553	0.195
2005	Portugal	50-69 years	4.258	6.173	2.640
2005	Portugal	70+ years	8.853	11.79	6.148
2005	Portugal	All Ages	2.207	2.894	1.612
2010	Portugal	Under 5	0.0	0.0	0.0
2010	Portugal	5-14 years	0.003	0.007	0.000
2010	Portugal	15-49 years	0.478	0.746	0.268
2010	Portugal	50-69 years	5.532	7.882	3.478
2010	Portugal	70+ years	10.93	14.21	7.749
2010	Portugal	All Ages	3.021	3.878	2.233
2015	Portugal	Under 5	0.0	0.0	0.0
2015	Portugal	5-14 years	0.003	0.008	0.000
2015	Portugal	15-49 years	0.536	0.854	0.300
2015	Portugal	50-69 years	6.833	9.919	4.311
2015	Portugal	70+ years	12.48	16.24	8.988
2015	Portugal	All Ages	3.840	4.958	2.848
2019	Portugal	Under 5	0.0	0.0	0.0
2019	Portugal	5-14 years	0.003	0.009	0.001
2019	Portugal	15-49 years	0.575	0.977	0.298
2019	Portugal	50-69 years	6.706	10.80	3.887
2019	Portugal	70+ years	12.44	17.36	8.199
2019	Portugal	All Ages	4.047	5.642	2.727
2000	Spain	Under 5	0.0	0.0	0.0
2000	Spain	5-14 years	0.006	0.013	0.002
2000	Spain	15-49 years	0.631	0.926	0.394
2000	Spain	50-69 years	12.21	16.35	8.461
2000	Spain	70+ years	25.85	32.06	19.25
2000	Spain	All Ages	5.917	7.224	4.631
2005	Spain	Under 5	0.0	0.0	0.0
2005	Spain	5-14 years	0.007	0.016	0.002
2005	Spain	15-49 years	1.045	1.547	0.626
2005	Spain	50-69 years	13.69	18.43	9.263

2005	Spain	70+ years	29.57	36.75	22.37
2005	Spain	All Ages	7.031	8.588	5.518
2010	Spain	Under 5	0.0	0.0	0.0
2010	Spain	5-14 years	0.008	0.018	0.002
2010	Spain	15-49 years	1.447	2.163	0.876
2010	Spain	50-69 years	15.02	20.07	10.42
2010	Spain	70+ years	29.35	35.91	22.34
2010	Spain	All Ages	7.661	9.319	6.038
2015	Spain	Under 5	0.0	0.0	0.0
2015	Spain	5-14 years	0.008	0.018	0.002
2015	Spain	15-49 years	1.344	2.061	0.788
2015	Spain	50-69 years	15.78	21.42	10.70
2015	Spain	70+ years	28.88	35.77	22.23
2015	Spain	All Ages	8.295	10.15	6.465
2019	Spain	Under 5	0.0	0.0	0.0
2019	Spain	5-14 years	0.008	0.019	0.002
2019	Spain	15-49 years	1.368	2.350	0.713
2019	Spain	50-69 years	14.26	21.53	8.742
2019	Spain	70+ years	27.30	36.43	19.02
2019	Spain	All Ages	8.252	11.37	5.803

Note: Estimates of liver cancer due to HBV or HCV were not generated for the group under the age of 5 years.

Table S68- Rates and 95% uncertainty levels (UL) of **incidence of liver cancer due to hepatitis C** per 100,000 population in Greece, Italy, Portugal and Spain from 2000 to 2019 by age group (Global Burden of Disease Study 2019).

Year	Country	Age	Incidence (95% UL)	95% UL (upper)	95% UL (lower)
2000	Greece	Under 5	0.0	0.0	0.0
2000	Greece	5-14 years	0.000	0.000	7.341
2000	Greece	15-49 years	0.029	0.049	0.015
2000	Greece	50-69 years	0.847	1.312	0.488
2000	Greece	70+ years	5.370	7.307	3.583
2000	Greece	All Ages	0.782	1.043	0.546
2005	Greece	Under 5	0.0	0.0	0.0
2005	Greece	5-14 years	0.000	0.000	7.546
2005	Greece	15-49 years	0.035	0.059	0.018
2005	Greece	50-69 years	0.857	1.329	0.492
2005	Greece	70+ years	5.389	7.491	3.523
2005	Greece	All Ages	0.884	1.196	0.611
2010	Greece	Under 5	0.0	0.0	0.0
2010	Greece	5-14 years	0.000	0.000	8.115
2010	Greece	15-49 years	0.045	0.074	0.023
2010	Greece	50-69 years	1.022	1.623	0.603
2010	Greece	70+ years	6.236	8.601	4.042
2010	Greece	All Ages	1.143	1.549	0.798
2015	Greece	Under 5	0.0	0.0	0.0
2015	Greece	5-14 years	0.000	0.000	7.604
2015	Greece	15-49 years	0.060	0.101	0.031
2015	Greece	50-69 years	1.290	2.072	0.736
2015	Greece	70+ years	6.965	9.795	4.569
2015	Greece	All Ages	1.431	1.948	0.999
2019	Greece	Under 5	0.0	0.0	0.0
2019	Greece	5-14 years	0.000	0.000	7.409
2019	Greece	15-49 years	0.060	0.107	0.029
2019	Greece	50-69 years	1.215	2.128	0.655
2019	Greece	70+ years	7.122	10.73	4.530
2019	Greece	All Ages	1.525	2.218	1.006
2000	Italy	Under 5	0.0	0.0	0.0
2000	Italy	5-14 years	0.003	0.005	0.002
2000	Italy	15-49 years	0.457	0.549	0.373
2000	Italy	50-69 years	11.24	12.89	9.716
2000	Italy	70+ years	31.69	34.69	28.56
2000	Italy	All Ages	7.002	7.628	6.364
2005	Italy	Under 5	0.0	0.0	0.0
2005	Italy	5-14 years	0.003	0.005	0.002
2005	Italy	15-49 years	0.468	0.561	0.380
2005	Italy	50-69 years	9.142	10.41	7.922
2005	Italy	70+ years	27.15	29.84	24.07
2005	Italy	All Ages	6.195	6.717	5.597
2010	Italy	Under 5	0.0	0.0	0.0
2010	Italy	5-14 years	0.003	0.005	0.001
2010	Italy	15-49 years	0.537	0.645	0.432
2010	Italy	50-69 years	9.112	10.45	7.901
2010	Italy	70+ years	28.43	31.28	24.91

2010	Italy	All Ages	6.717	7.295	6.023
2015	Italy	Under 5	0.0	0.0	0.0
2015	Italy	5-14 years	0.003	0.005	0.001
2015	Italy	15-49 years	0.572	0.690	0.461
2015	Italy	50-69 years	9.195	10.60	7.943
2015	Italy	70+ years	28.17	31.14	24.44
2015	Italy	All Ages	7.127	7.775	6.349
2019	Italy	Under 5	0.0	0.0	0.0
2019	Italy	5-14 years	0.003	0.005	0.001
2019	Italy	15-49 years	0.535	0.717	0.378
2019	Italy	50-69 years	8.772	11.63	6.556
2019	Italy	70+ years	27.55	33.34	21.79
2019	Italy	All Ages	7.358	9.057	5.915
2000	Portugal	Under 5	0.0	0.0	0.0
2000	Portugal	5-14 years	0.001	0.003	0.000
2000	Portugal	15-49 years	0.164	0.259	0.097
2000	Portugal	50-69 years	3.211	4.652	2.033
2000	Portugal	70+ years	10.07	13.06	7.165
2000	Portugal	All Ages	1.869	2.410	1.378
2005	Portugal	Under 5	0.0	0.0	0.0
2005	Portugal	5-14 years	0.001	0.003	0.000
2005	Portugal	15-49 years	0.208	0.327	0.116
2005	Portugal	50-69 years	3.376	4.902	2.119
2005	Portugal	70+ years	10.03	13.25	7.024
2005	Portugal	All Ages	2.079	2.714	1.515
2010	Portugal	Under 5	0.0	0.0	0.0
2010	Portugal	5-14 years	0.001	0.003	0.000
2010	Portugal	15-49 years	0.270	0.418	0.152
2010	Portugal	50-69 years	4.224	6.028	2.684
2010	Portugal	70+ years	12.25	15.76	8.663
2010	Portugal	All Ages	2.788	3.538	2.069
2015	Portugal	Under 5	0.0	0.0	0.0
2015	Portugal	5-14 years	0.001	0.003	0.000
2015	Portugal	15-49 years	0.283	0.452	0.160
2015	Portugal	50-69 years	5.038	7.240	3.207
2015	Portugal	70+ years	13.80	17.72	10.11
2015	Portugal	All Ages	3.456	4.390	2.586
2019	Portugal	Under 5	0.0	0.0	0.0
2019	Portugal	5-14 years	0.001	0.003	0.000
2019	Portugal	15-49 years	0.295	0.504	0.152
2019	Portugal	50-69 years	4.898	7.808	2.852
2019	Portugal	70+ years	13.71	19.07	9.239
2019	Portugal	All Ages	3.640	5.061	2.516
2000	Spain	Under 5	0.0	0.0	0.0
2000	Spain	5-14 years	0.002	0.005	0.000
2000	Spain	15-49 years	0.310	0.446	0.195
2000	Spain	50-69 years	8.073	10.74	5.753
2000	Spain	70+ years	25.49	31.28	19.11
2000	Spain	All Ages	4.831	5.857	3.794
2005	Spain	Under 5	0.0	0.0	0.0
2005	Spain	5-14 years	0.002	0.005	0.000
2005	Spain	15-49 years	0.435	0.635	0.265
2005	Spain	50-69 years	7.897	10.49	5.479

2005	Spain	70+ years	27.30	33.54	20.88
2005	Spain	All Ages	5.232	6.323	4.118
2010	Spain	Under 5	0.0	0.0	0.0
2010	Spain	5-14 years	0.002	0.005	0.000
2010	Spain	15-49 years	0.554	0.808	0.339
2010	Spain	50-69 years	8.439	11.16	5.890
2010	Spain	70+ years	27.46	33.35	21.09
2010	Spain	All Ages	5.531	6.591	4.424
2015	Spain	Under 5	0.0	0.0	0.0
2015	Spain	5-14 years	0.002	0.005	0.000
2015	Spain	15-49 years	0.502	0.747	0.303
2015	Spain	50-69 years	8.987	11.91	6.206
2015	Spain	70+ years	27.66	33.86	21.28
2015	Spain	All Ages	6.093	7.344	4.894
2019	Spain	Under 5	0.0	0.0	0.0
2019	Spain	5-14 years	0.002	0.005	0.000
2019	Spain	15-49 years	0.507	0.842	0.277
2019	Spain	50-69 years	8.231	12.30	5.152
2019	Spain	70+ years	26.41	34.79	18.76
2019	Spain	All Ages	6.169	8.150	4.394

Note: Estimates of liver cancer due to HBV or HCV were not generated for the group under the age of 5 years.

Table S69- Rates and 95% uncertainty levels (UL) of **deaths due to liver cancer due to hepatitis C** per 100,000 population in Greece, Italy, Portugal and Spain from 2000 to 2019 by age group (Global Burden of Disease Study 2019).

Year	Country	Age	Deaths (95% UL)	95% UL (upper)	95% UL (lower)
2000	Greece	5-14 years	5.201	0.000	1.213
2000	Greece	15-49 years	0.019	0.032	0.010
2000	Greece	50-69 years	0.743	1.149	0.435
2000	Greece	70+ years	6.018	8.171	4.041
2000	Greece	All Ages	0.822	1.099	0.577
2005	Greece	5-14 years	5.199	0.000	1.235
2005	Greece	15-49 years	0.022	0.037	0.011
2005	Greece	50-69 years	0.724	1.127	0.415
2005	Greece	70+ years	5.862	8.125	3.870
2005	Greece	All Ages	0.906	1.223	0.618
2010	Greece	5-14 years	5.662	0.000	1.301
2010	Greece	15-49 years	0.027	0.046	0.014
2010	Greece	50-69 years	0.850	1.358	0.488
2010	Greece	70+ years	6.749	9.279	4.454
2010	Greece	All Ages	1.169	1.587	0.804
2015	Greece	5-14 years	5.419	0.000	1.247
2015	Greece	15-49 years	0.037	0.063	0.019
2015	Greece	50-69 years	1.081	1.751	0.611
2015	Greece	70+ years	7.710	10.82	5.094
2015	Greece	All Ages	1.485	2.018	1.030
2019	Greece	5-14 years	5.619	0.000	1.220
2019	Greece	15-49 years	0.037	0.063	0.019
2019	Greece	50-69 years	1.013	1.650	0.579
2019	Greece	70+ years	7.960	11.46	5.286
2019	Greece	All Ages	1.602	2.192	1.115
2000	Italy	5-14 years	0.000	0.001	0.000
2000	Italy	15-49 years	0.264	0.315	0.217
2000	Italy	50-69 years	9.005	10.31	7.816
2000	Italy	70+ years	33.48	36.39	30.22
2000	Italy	All Ages	6.599	7.151	6.013
2005	Italy	5-14 years	0.000	0.000	0.000
2005	Italy	15-49 years	0.243	0.289	0.200
2005	Italy	50-69 years	6.747	7.661	5.868
2005	Italy	70+ years	27.79	30.45	24.51
2005	Italy	All Ages	5.603	6.071	5.041
2010	Italy	5-14 years	0.000	0.000	0.000
2010	Italy	15-49 years	0.273	0.325	0.223
2010	Italy	50-69 years	6.534	7.463	5.700
2010	Italy	70+ years	29.23	32.11	25.41
2010	Italy	All Ages	6.089	6.617	5.439
2015	Italy	5-14 years	0.000	0.000	0.000
2015	Italy	15-49 years	0.282	0.339	0.227
2015	Italy	50-69 years	6.412	7.343	5.566
2015	Italy	70+ years	28.74	31.68	24.61
2015	Italy	All Ages	6.362	6.919	5.620
2019	Italy	5-14 years	0.000	0.000	0.000
2019	Italy	15-49 years	0.266	0.333	0.210
2019	Italy	50-69 years	6.198	7.205	5.228

2019	Italy	70+ years	28.45	31.77	24.13
2019	Italy	All Ages	6.685	7.381	5.861
2000	Portugal	5-14 years	0.000	0.001	0.000
2000	Portugal	15-49 years	0.134	0.211	0.079
2000	Portugal	50-69 years	3.052	4.389	1.927
2000	Portugal	70+ years	11.89	15.40	8.548
2000	Portugal	All Ages	2.014	2.583	1.485
2005	Portugal	5-14 years	0.000	0.001	0.000
2005	Portugal	15-49 years	0.162	0.251	0.091
2005	Portugal	50-69 years	3.144	4.567	1.985
2005	Portugal	70+ years	11.86	15.35	8.437
2005	Portugal	All Ages	2.224	2.844	1.646
2010	Portugal	5-14 years	0.000	0.001	0.000
2010	Portugal	15-49 years	0.200	0.311	0.112
2010	Portugal	50-69 years	3.797	5.425	2.409
2010	Portugal	70+ years	14.27	18.17	10.16
2010	Portugal	All Ages	2.925	3.703	2.184
2015	Portugal	5-14 years	0.000	0.001	0.000
2015	Portugal	15-49 years	0.204	0.325	0.114
2015	Portugal	50-69 years	4.515	6.474	2.876
2015	Portugal	70+ years	16.22	20.78	11.84
2015	Portugal	All Ages	3.640	4.602	2.716
2019	Portugal	5-14 years	0.000	0.001	0.000
2019	Portugal	15-49 years	0.208	0.350	0.114
2019	Portugal	50-69 years	4.301	6.350	2.697
2019	Portugal	70+ years	15.92	20.58	11.45
2019	Portugal	All Ages	3.796	4.808	2.832
2000	Spain	5-14 years	0.000	0.001	0.000
2000	Spain	15-49 years	0.211	0.305	0.131
2000	Spain	50-69 years	6.752	8.927	4.844
2000	Spain	70+ years	27.71	33.81	21.07
2000	Spain	All Ages	4.759	5.753	3.734
2005	Spain	5-14 years	0.000	0.001	0.000
2005	Spain	15-49 years	0.262	0.375	0.160
2005	Spain	50-69 years	6.056	8.018	4.215
2005	Spain	70+ years	28.68	34.78	22.20
2005	Spain	All Ages	4.931	5.941	3.902
2010	Spain	5-14 years	0.000	0.001	0.000
2010	Spain	15-49 years	0.313	0.461	0.190
2010	Spain	50-69 years	6.392	8.406	4.490
2010	Spain	70+ years	29.28	35.38	22.53
2010	Spain	All Ages	5.186	6.160	4.188
2015	Spain	5-14 years	0.000	0.001	0.000
2015	Spain	15-49 years	0.277	0.402	0.171
2015	Spain	50-69 years	6.837	8.947	4.721
2015	Spain	70+ years	29.81	36.34	22.92
2015	Spain	All Ages	5.753	6.937	4.610
2019	Spain	5-14 years	0.000	0.001	0.000
2019	Spain	15-49 years	0.281	0.425	0.168
2019	Spain	50-69 years	6.363	8.601	4.267
2019	Spain	70+ years	28.92	35.36	22.32
2019	Spain	All Ages	5.943	7.116	4.742

Table S70- Rates and 95% uncertainty levels (UL) of **years of life lost** (YLLs) **due to liver cancer due to hepatitis C** per 100,000 population in Greece, Italy, Portugal and Spain from 2000 to 2019 by age group (Global Burden of Disease Study 2019).

Year	Country	Age	YLLs (95% UL)	95% UL (upper)	95% UL (lower)
2000	Greece	5-14 years	0.003	0.010	0.000
2000	Greece	15-49 years	0.887	1.471	0.481
2000	Greece	50-69 years	20.14	31.14	11.75
2000	Greece	70+ years	75.62	105.1	50.55
2000	Greece	All Ages	13.12	17.64	9.168
2005	Greece	5-14 years	0.003	0.009	0.000
2005	Greece	15-49 years	0.992	1.648	0.539
2005	Greece	50-69 years	19.93	31.24	11.46
2005	Greece	70+ years	74.17	104.1	48.00
2005	Greece	All Ages	14.26	19.63	9.777
2010	Greece	5-14 years	0.004	0.010	0.000
2010	Greece	15-49 years	1.243	2.048	0.653
2010	Greece	50-69 years	23.71	37.68	13.77
2010	Greece	70+ years	83.57	116.9	53.92
2010	Greece	All Ages	17.93	24.50	12.30
2015	Greece	5-14 years	0.004	0.010	0.000
2015	Greece	15-49 years	1.673	2.797	0.862
2015	Greece	50-69 years	29.95	48.35	17.02
2015	Greece	70+ years	90.86	127.5	58.38
2015	Greece	All Ages	22.33	31.01	15.38
2019	Greece	5-14 years	0.004	0.011	0.000
2019	Greece	15-49 years	1.662	2.776	0.872
2019	Greece	50-69 years	28.13	45.92	16.34
2019	Greece	70+ years	90.72	128.9	59.31
2019	Greece	All Ages	23.10	31.96	15.94
2000	Italy	5-14 years	0.066	0.094	0.043
2000	Italy	15-49 years	11.89	14.12	9.821
2000	Italy	50-69 years	246.8	282.5	214.1
2000	Italy	70+ years	476.6	520.4	429.8
2000	Italy	All Ages	126.4	138.0	115.0
2005	Italy	5-14 years	0.045	0.064	0.029
2005	Italy	15-49 years	10.88	12.93	8.982
2005	Italy	50-69 years	185.4	210.2	161.1
2005	Italy	70+ years	383.5	421.5	341.4
2005	Italy	All Ages	103.0	111.8	93.53
2010	Italy	5-14 years	0.039	0.056	0.026
2010	Italy	15-49 years	12.07	14.39	9.957
2010	Italy	50-69 years	181.6	207.1	158.3
2010	Italy	70+ years	390.9	430.2	345.5
2010	Italy	All Ages	108.2	117.2	98.28
2015	Italy	5-14 years	0.036	0.051	0.023
2015	Italy	15-49 years	12.42	14.91	10.06
2015	Italy	50-69 years	179.8	205.6	156.1
2015	Italy	70+ years	373.7	411.8	325.1
2015	Italy	All Ages	111.7	121.5	100.5
2019	Italy	5-14 years	0.037	0.053	0.024
2019	Italy	15-49 years	11.74	14.61	9.319
2019	Italy	50-69 years	174.0	202.3	146.8

2019	Italy	70+ years	369.0	412.2	315.0
2019	Italy	All Ages	116.1	128.4	102.3
2000	Portugal	5-14 years	0.058	0.136	0.016
2000	Portugal	15-49 years	6.109	9.511	3.598
2000	Portugal	50-69 years	84.98	122.3	53.84
2000	Portugal	70+ years	166.9	219.3	115.2
2000	Portugal	All Ages	39.66	51.88	29.14
2005	Portugal	5-14 years	0.052	0.119	0.015
2005	Portugal	15-49 years	7.269	11.08	4.172
2005	Portugal	50-69 years	88.85	128.3	56.10
2005	Portugal	70+ years	163.8	215.3	112.0
2005	Portugal	All Ages	43.52	56.33	31.65
2010	Portugal	5-14 years	0.045	0.109	0.012
2010	Portugal	15-49 years	8.943	13.72	5.104
2010	Portugal	50-69 years	109.1	156.3	69.43
2010	Portugal	70+ years	192.0	249.8	133.0
2010	Portugal	All Ages	56.20	71.89	41.31
2015	Portugal	5-14 years	0.040	0.096	0.011
2015	Portugal	15-49 years	9.068	14.38	5.087
2015	Portugal	50-69 years	129.6	186.9	82.93
2015	Portugal	70+ years	211.2	277.2	149.4
2015	Portugal	All Ages	68.59	88.65	50.81
2019	Portugal	5-14 years	0.043	0.102	0.012
2019	Portugal	15-49 years	9.243	15.28	5.128
2019	Portugal	50-69 years	122.6	181.2	75.98
2019	Portugal	70+ years	205.4	271.5	143.4
2019	Portugal	All Ages	69.91	90.95	50.43
2000	Spain	5-14 years	0.064	0.144	0.021
2000	Spain	15-49 years	9.567	13.76	6.033
2000	Spain	50-69 years	184.5	243.2	131.7
2000	Spain	70+ years	387.1	478.9	288.7
2000	Spain	All Ages	89.00	108.2	69.85
2005	Spain	5-14 years	0.057	0.126	0.017
2005	Spain	15-49 years	11.71	16.83	7.200
2005	Spain	50-69 years	170.1	225.2	118.1
2005	Spain	70+ years	391.0	484.4	296.9
2005	Spain	All Ages	89.58	108.6	70.69
2010	Spain	5-14 years	0.050	0.109	0.016
2010	Spain	15-49 years	13.83	20.28	8.524
2010	Spain	50-69 years	182.4	239.9	128.2
2010	Spain	70+ years	380.7	463.9	290.5
2010	Spain	All Ages	94.14	112.8	74.28
2015	Spain	5-14 years	0.042	0.095	0.013
2015	Spain	15-49 years	12.19	17.73	7.583
2015	Spain	50-69 years	195.7	256.3	136.8
2015	Spain	70+ years	375.3	462.7	284.8
2015	Spain	All Ages	103.0	124.4	81.29
2019	Spain	5-14 years	0.045	0.106	0.013
2019	Spain	15-49 years	12.39	18.55	7.397
2019	Spain	50-69 years	182.0	245.2	123.1
2019	Spain	70+ years	360.6	448.0	278.0
2019	Spain	All Ages	104.7	129.4	80.77

Table S71- Rates and 95% uncertainty levels (UL) of **years lived with disability** (YLDs) **due to liver cancer due to hepatitis C** per 100,000 population in Greece, Italy, Portugal and Spain from 2000 to 2019 by age group (Global Burden of Disease Study 2019).

Year	Country	Age	YLDs (95% UL)	95% UL (upper)	95% UL (lower)
2000	Greece	Under 5	0.0	0.0	0.0
2000	Greece	5-14 years	9.086	0.000	2.005
2000	Greece	15-49 years	0.007	0.014	0.003
2000	Greece	50-69 years	0.205	0.342	0.106
2000	Greece	70+ years	1.210	1.833	0.702
2000	Greece	All Ages	0.180	0.269	0.107
2005	Greece	Under 5	0.0	0.0	0.0
2005	Greece	5-14 years	9.592	0.000	2.354
2005	Greece	15-49 years	0.009	0.017	0.004
2005	Greece	50-69 years	0.210	0.352	0.108
2005	Greece	70+ years	1.226	1.894	0.699
2005	Greece	All Ages	0.205	0.306	0.121
2010	Greece	Under 5	0.0	0.0	0.0
2010	Greece	5-14 years	0.000	0.000	2.439
2010	Greece	15-49 years	0.012	0.023	0.006
2010	Greece	50-69 years	0.253	0.435	0.129
2010	Greece	70+ years	1.427	2.199	0.812
2010	Greece	All Ages	0.267	0.406	0.160
2015	Greece	Under 5	0.0	0.0	0.0
2015	Greece	5-14 years	9.652	0.000	2.149
2015	Greece	15-49 years	0.016	0.030	0.008
2015	Greece	50-69 years	0.318	0.546	0.162
2015	Greece	70+ years	1.584	2.444	0.927
2015	Greece	All Ages	0.333	0.499	0.198
2019	Greece	Under 5	0.0	0.0	0.0
2019	Greece	5-14 years	0.000	0.000	2.203
2019	Greece	15-49 years	0.016	0.031	0.007
2019	Greece	50-69 years	0.300	0.548	0.151
2019	Greece	70+ years	1.612	2.592	0.892
2019	Greece	All Ages	0.353	0.558	0.198
2000	Italy	Under 5	0.0	0.0	0.0
2000	Italy	5-14 years	0.001	0.001	0.000
2000	Italy	15-49 years	0.133	0.186	0.089
2000	Italy	50-69 years	2.817	3.858	1.909
2000	Italy	70+ years	7.001	9.300	4.946
2000	Italy	All Ages	1.643	2.183	1.158
2005	Italy	Under 5	0.0	0.0	0.0
2005	Italy	5-14 years	0.001	0.001	0.000
2005	Italy	15-49 years	0.144	0.199	0.095
2005	Italy	50-69 years	2.387	3.237	1.645
2005	Italy	70+ years	6.196	8.278	4.316
2005	Italy	All Ages	1.504	1.990	1.068
2010	Italy	Under 5	0.0	0.0	0.0
2010	Italy	5-14 years	0.001	0.001	0.000
2010	Italy	15-49 years	0.167	0.235	0.109
2010	Italy	50-69 years	2.422	3.271	1.659
2010	Italy	70+ years	6.490	8.712	4.561

2010	Italy	All Ages	1.637	2.186	1.172
2015	Italy	Under 5	0.0	0.0	0.0
2015	Italy	5-14 years	0.001	0.001	0.000
2015	Italy	15-49 years	0.180	0.252	0.118
2015	Italy	50-69 years	2.464	3.380	1.666
2015	Italy	70+ years	6.417	8.609	4.504
2015	Italy	All Ages	1.741	2.338	1.242
2019	Italy	Under 5	0.0	0.0	0.0
2019	Italy	5-14 years	0.001	0.001	0.000
2019	Italy	15-49 years	0.168	0.258	0.101
2019	Italy	50-69 years	2.350	3.499	1.500
2019	Italy	70+ years	6.297	8.978	4.212
2019	Italy	All Ages	1.796	2.568	1.183
2000	Portugal	Under 5	0.0	0.0	0.0
2000	Portugal	5-14 years	0.000	0.000	0.000
2000	Portugal	15-49 years	0.040	0.066	0.021
2000	Portugal	50-69 years	0.748	1.199	0.433
2000	Portugal	70+ years	2.207	3.202	1.349
2000	Portugal	All Ages	0.421	0.605	0.263
2005	Portugal	Under 5	0.0	0.0	0.0
2005	Portugal	5-14 years	0.000	0.000	0.000
2005	Portugal	15-49 years	0.052	0.088	0.027
2005	Portugal	50-69 years	0.797	1.287	0.448
2005	Portugal	70+ years	2.209	3.208	1.331
2005	Portugal	All Ages	0.473	0.679	0.295
2010	Portugal	Under 5	0.0	0.0	0.0
2010	Portugal	5-14 years	0.000	0.000	0.000
2010	Portugal	15-49 years	0.069	0.119	0.035
2010	Portugal	50-69 years	1.008	1.585	0.566
2010	Portugal	70+ years	2.705	3.921	1.670
2010	Portugal	All Ages	0.638	0.903	0.394
2015	Portugal	Under 5	0.0	0.0	0.0
2015	Portugal	5-14 years	0.000	0.000	0.000
2015	Portugal	15-49 years	0.074	0.129	0.038
2015	Portugal	50-69 years	1.213	1.952	0.693
2015	Portugal	70+ years	3.057	4.370	1.872
2015	Portugal	All Ages	0.796	1.123	0.505
2019	Portugal	Under 5	0.0	0.0	0.0
2019	Portugal	5-14 years	0.000	0.000	0.000
2019	Portugal	15-49 years	0.078	0.143	0.037
2019	Portugal	50-69 years	1.182	1.997	0.613
2019	Portugal	70+ years	3.038	4.595	1.762
2019	Portugal	All Ages	0.838	1.263	0.480
2000	Spain	Under 5	0.0	0.0	0.0
2000	Spain	5-14 years	0.000	0.001	0.000
2000	Spain	15-49 years	0.083	0.134	0.045
2000	Spain	50-69 years	1.989	2.962	1.194
2000	Spain	70+ years	5.739	8.070	3.685
2000	Spain	All Ages	1.131	1.563	0.724
2005	Spain	Under 5	0.0	0.0	0.0
2005	Spain	5-14 years	0.000	0.001	0.000
2005	Spain	15-49 years	0.124	0.193	0.066
2005	Spain	50-69 years	2.028	3.080	1.213

2005	Spain	70+ years	6.185	8.751	4.041
2005	Spain	All Ages	1.248	1.737	0.819
2010	Spain	Under 5	0.0	0.0	0.0
2010	Spain	5-14 years	0.000	0.001	0.000
2010	Spain	15-49 years	0.164	0.257	0.088
2010	Spain	50-69 years	2.178	3.269	1.353
2010	Spain	70+ years	6.196	8.759	3.978
2010	Spain	All Ages	1.328	1.837	0.869
2015	Spain	Under 5	0.0	0.0	0.0
2015	Spain	5-14 years	0.000	0.001	0.000
2015	Spain	15-49 years	0.150	0.238	0.080
2015	Spain	50-69 years	2.312	3.492	1.389
2015	Spain	70+ years	6.167	8.697	3.989
2015	Spain	All Ages	1.451	1.995	0.962
2019	Spain	Under 5	0.0	0.0	0.0
2019	Spain	5-14 years	0.000	0.001	0.000
2019	Spain	15-49 years	0.152	0.265	0.076
2019	Spain	50-69 years	2.107	3.433	1.155
2019	Spain	70+ years	5.898	8.561	3.556
2019	Spain	All Ages	1.465	2.166	0.892

Note: Estimates of liver cancer due to HBV or HCV were not generated for the group under the age of 5 years.

Table S72- Rates and 95% uncertainty levels (UL) of **disability-adjusted life years** (DALYs) **due to liver cancer due to hepatitis C** per 100,000 population in Greece, Italy, Portugal and Spain from 2000 to 2019 by age group (Global Burden of Disease Study 2019).

Year	Country	Age	DALYs (95% UL)	95% UL (upper)	95% UL (lower)
2000	Greece	Under 5	0.0	0.0	0.0
2000	Greece	5-14 years	0.004	0.010	0.000
2000	Greece	15-49 years	0.895	1.484	0.485
2000	Greece	50-69 years	20.35	31.48	11.87
2000	Greece	70+ years	76.83	107.0	51.42
2000	Greece	All Ages	13.30	17.89	9.279
2005	Greece	Under 5	0.0	0.0	0.0
2005	Greece	5-14 years	0.004	0.009	0.000
2005	Greece	15-49 years	1.002	1.666	0.544
2005	Greece	50-69 years	20.14	31.51	11.57
2005	Greece	70+ years	75.40	105.8	48.79
2005	Greece	All Ages	14.46	19.90	9.906
2010	Greece	Under 5	0.0	0.0	0.0
2010	Greece	5-14 years	0.004	0.010	0.001
2010	Greece	15-49 years	1.255	2.072	0.660
2010	Greece	50-69 years	23.97	38.04	13.89
2010	Greece	70+ years	85.00	118.6	54.77
2010	Greece	All Ages	18.20	24.88	12.48
2015	Greece	Under 5	0.0	0.0	0.0
2015	Greece	5-14 years	0.004	0.010	0.000
2015	Greece	15-49 years	1.690	2.830	0.870
2015	Greece	50-69 years	30.27	48.91	17.18
2015	Greece	70+ years	92.44	129.9	59.20
2015	Greece	All Ages	22.66	31.49	15.59
2019	Greece	Under 5	0.0	0.0	0.0
2019	Greece	5-14 years	0.004	0.011	0.000
2019	Greece	15-49 years	1.679	2.805	0.881
2019	Greece	50-69 years	28.43	46.38	16.49
2019	Greece	70+ years	92.33	131.5	60.25
2019	Greece	All Ages	23.45	32.56	16.16
2000	Italy	Under 5	0.0	0.0	0.0
2000	Italy	5-14 years	0.067	0.096	0.043
2000	Italy	15-49 years	12.03	14.29	9.916
2000	Italy	50-69 years	249.7	285.9	216.5
2000	Italy	70+ years	483.6	528.2	435.8
2000	Italy	All Ages	128.1	140.0	116.5
2005	Italy	Under 5	0.0	0.0	0.0
2005	Italy	5-14 years	0.046	0.066	0.030
2005	Italy	15-49 years	11.02	13.11	9.112
2005	Italy	50-69 years	187.7	213.0	163.2
2005	Italy	70+ years	389.7	427.9	346.8
2005	Italy	All Ages	104.5	113.5	94.87
2010	Italy	Under 5	0.0	0.0	0.0
2010	Italy	5-14 years	0.041	0.057	0.026
2010	Italy	15-49 years	12.24	14.58	10.09
2010	Italy	50-69 years	184.1	209.9	160.5
2010	Italy	70+ years	397.4	437.3	351.1

2010	Italy	All Ages	109.8	119.2	99.66
2015	Italy	Under 5	0.0	0.0	0.0
2015	Italy	5-14 years	0.037	0.053	0.024
2015	Italy	15-49 years	12.60	15.11	10.21
2015	Italy	50-69 years	182.3	208.7	158.1
2015	Italy	70+ years	380.1	419.3	329.9
2015	Italy	All Ages	113.5	123.5	102.0
2019	Italy	Under 5	0.0	0.0	0.0
2019	Italy	5-14 years	0.038	0.055	0.024
2019	Italy	15-49 years	11.91	14.87	9.452
2019	Italy	50-69 years	176.4	205.2	148.7
2019	Italy	70+ years	375.3	419.3	319.4
2019	Italy	All Ages	117.9	130.6	103.9
2000	Portugal	Under 5	0.0	0.0	0.0
2000	Portugal	5-14 years	0.059	0.137	0.016
2000	Portugal	15-49 years	6.149	9.582	3.621
2000	Portugal	50-69 years	85.73	123.6	54.31
2000	Portugal	70+ years	169.1	222.3	116.7
2000	Portugal	All Ages	40.09	52.45	29.44
2005	Portugal	Under 5	0.0	0.0	0.0
2005	Portugal	5-14 years	0.052	0.120	0.015
2005	Portugal	15-49 years	7.322	11.17	4.202
2005	Portugal	50-69 years	89.64	129.3	56.53
2005	Portugal	70+ years	166.0	218.1	113.6
2005	Portugal	All Ages	43.99	56.98	31.99
2010	Portugal	Under 5	0.0	0.0	0.0
2010	Portugal	5-14 years	0.046	0.109	0.012
2010	Portugal	15-49 years	9.013	13.86	5.148
2010	Portugal	50-69 years	110.1	158.1	70.06
2010	Portugal	70+ years	194.7	253.1	134.9
2010	Portugal	All Ages	56.83	72.78	41.81
2015	Portugal	Under 5	0.0	0.0	0.0
2015	Portugal	5-14 years	0.040	0.096	0.011
2015	Portugal	15-49 years	9.142	14.49	5.128
2015	Portugal	50-69 years	130.9	188.8	83.73
2015	Portugal	70+ years	214.2	280.9	151.4
2015	Portugal	All Ages	69.39	89.76	51.29
2019	Portugal	Under 5	0.0	0.0	0.0
2019	Portugal	5-14 years	0.044	0.103	0.012
2019	Portugal	15-49 years	9.321	15.45	5.168
2019	Portugal	50-69 years	123.8	182.9	76.67
2019	Portugal	70+ years	208.4	275.6	145.1
2019	Portugal	All Ages	70.75	92.26	51.06
2000	Spain	Under 5	0.0	0.0	0.0
2000	Spain	5-14 years	0.065	0.145	0.021
2000	Spain	15-49 years	9.651	13.86	6.082
2000	Spain	50-69 years	186.5	245.6	133.1
2000	Spain	70+ years	392.8	485.9	292.8
2000	Spain	All Ages	90.13	109.6	70.73
2005	Spain	Under 5	0.0	0.0	0.0
2005	Spain	5-14 years	0.058	0.127	0.017
2005	Spain	15-49 years	11.83	17.00	7.285
2005	Spain	50-69 years	172.1	227.8	119.4

2005	Spain	70+ years	397.2	492.2	302.0
2005	Spain	All Ages	90.83	110.1	71.47
2010	Spain	Under 5	0.0	0.0	0.0
2010	Spain	5-14 years	0.051	0.110	0.016
2010	Spain	15-49 years	14.00	20.50	8.622
2010	Spain	50-69 years	184.6	242.3	130.0
2010	Spain	70+ years	386.9	472.0	294.8
2010	Spain	All Ages	95.47	114.5	75.30
2015	Spain	Under 5	0.0	0.0	0.0
2015	Spain	5-14 years	0.043	0.097	0.013
2015	Spain	15-49 years	12.34	17.95	7.690
2015	Spain	50-69 years	198.1	259.5	138.4
2015	Spain	70+ years	381.4	470.8	289.8
2015	Spain	All Ages	104.4	126.1	82.33
2019	Spain	Under 5	0.0	0.0	0.0
2019	Spain	5-14 years	0.046	0.107	0.014
2019	Spain	15-49 years	12.54	18.76	7.468
2019	Spain	50-69 years	184.1	248.0	124.4
2019	Spain	70+ years	366.5	455.5	280.7
2019	Spain	All Ages	106.2	131.1	81.82

Note: Estimates of liver cancer due to HBV or HCV were not generated for the group under the age of 5 years.

Table S73- Prevalence of acute and chronic hepatitis B for Greece, Italy, Portugal and Spain, Western Europe, and globally in 2000, 2010, and 2019 (Global Burden of Disease Study 2019).

**Acute hepatitis B**									
	**Number of prevalent cases (all ages)**	**95% UL (lower)**	**95% UL (upper)**	**Rate of prevalent cases (age-standardized, per 100 000)**	**95% UL (lower)**	**95% UL (upper)**	**Rate of prevalent cases (all ages, per 100 000)**	**95% UL (lower)**	**95% UL (upper)**
**Global**									
2000	10 654 640	8 629 245	12 803 529	169,82	137,69	202,38	173,09	140,19	208,00
2010	10 264 164	8 418 468	12 444 295	143,31	117,99	173,39	146,90	120,48	178,10
2019	9 233 458	7 384 581	11 341 191	115,66	92,73	141,62	119,33	95,44	146,58
**Greece**									
2000	8 088	6 388	9 920	71,10	57,85	85,75	72,92	57,60	89,44
2010	7 040	5 453	9 024	59,47	46,78	74,42	63,56	49,23	81,47
2019	5 735	4 365	7 146	51,80	40,88	64,06	55,48	42,23	69,13
**Italy**									
2000	15 833	12 024	20 199	25,73	19,67	32,71	27,94	21,22	35,65
2010	11 392	8 066	15 064	16,04	11,69	21,06	18,88	13,37	24,97
2019	7 912	5 054	11 153	9,95	6,82	13,49	13,12	8,38	18,49
**Portugal**									
2000	3 145	2 262	4 214	31,38	23,43	41,37	29,82	21,45	39,97
2010	2 668	1 825	3 669	24,88	17,29	34,05	24,71	16,91	33,98
2019	2 013	1 262	2 875	17,96	12,04	24,96	18,90	11,84	26,99
**Spain**									
2000	12 888	10 000	16 226	30,37	23,80	37,65	31,58	24,50	39,76
2010	12 485	9 217	16 251	23,79	18,19	30,67	26,58	19,62	34,59
2019	9 606	6 926	12 660	17,50	13,17	22,48	20,87	15,05	27,51
**Western Europe**									
2000	119 073	96 032	144 678	30,28	24,98	36,29	29,84	24,06	36,25
2010	109 709	87 281	135 071	25,92	21,32	31,43	25,96	20,66	31,97
2019	96 003	73 710	118 572	21,19	16,84	25,93	22,00	16,89	27,18

Cirrhosis and other chronic liver diseases due to hepatitis B									
	**Number of prevalent cases (all ages)**	**95% UL (lower)**	**95% UL (upper)**	**Rate of prevalent cases (age-standardized, per 100 000)**	**95% UL (lower)**	**95% UL (upper)**	**Rate of prevalent cases (all ages, per 100 000)**	**95% UL (lower)**	**95% UL (upper)**
**Global**									
2000	352 957 126	312 419 788	393 065 113	5 719,78	5 088,92	6 351,01	5 734,10	5 075,53	6 385,69
2010	333 778 378	300 910 157	369 350 851	4 717,49	4 257,66	5 217,29	4 776,92	4 306,52	5 286,02
2019	316 689 072	283 569 362	350 879 812	3 951,47	3 538,09	4 384,71	4 092,93	3 664,89	4 534,82
**Greece**									
2000	228 879	208 513	251 696	1 835,83	1 668,07	2 029,19	2 063,54	1 879,93	2 269,26
2010	207 673	188 965	228 397	1 530,15	1 387,84	1 679,96	1 874,81	1 705,92	2 061,90
2019	185 964	170 082	203 812	1 374,57	1 261,09	1 505,64	1 798,98	1 645,34	1 971,64
**Italy**									
2000	578 811	519 274	637 692	856,78	765,72	951,18	1 021,49	916,42	1 125,41
2010	512 689	454 142	570 370	639,74	565,25	713,30	849,84	752,79	945,45
2019	430 183	375 764	484 727	463,96	408,04	520,20	713,25	623,02	803,68
**Portugal**									
2000	124 574	102 139	149 761	1 118,02	907,74	1 367,33	1 181,48	968,70	1 420,36
2010	113 324	94 553	133 661	915,56	765,34	1 083,90	1 049,75	875,87	1 238,13
2019	96 485	78 360	116 117	706,41	580,52	845,94	905,86	735,69	1 090,17
**Spain**									
2000	393 389	348 828	436 272	857,12	757,76	954,48	963,95	854,76	1 069,03
2010	401 909	358 739	447 513	697,86	622,20	776,34	855,56	763,66	952,64
2019	345 692	307 911	382 208	548,45	489,14	605,02	751,16	669,06	830,50
**Western Europe**									
2000	3 705 036	3 340 096	4 051 616	855,33	767,39	945,71	928,42	836,97	1 015,26
2010	3 623 186	3 265 008	3 962 979	756,12	677,75	831,04	857,45	772,68	937,86
2019	3 383 203	3 041 504	3 696 225	643,72	577,98	706,76	775,42	697,10	847,16

Liver cancer due to hepatitis B									
	**Number of prevalent cases (all ages)**	**95% UL (lower)**	**95% UL (upper)**	**Rate of prevalent cases (age-**	**95% UL (lower)**	**95% UL (upper)**	**Rate of prevalent cases (all ages, per 100 000)**	**95% UL (lower)**	**95% UL (upper)**

				**standardized, per 100 000)**					
**Global**									
2000	306 424	275 258	338 364	5,68	5,10	6,30	4,98	4,47	5,50
2010	233 754	209 851	259 087	3,44	3,09	3,83	3,35	3,00	3,71
2019	314 204	266 865	364 822	3,76	3,19	4,36	4,06	3,45	4,72
**Greece**									
2000	236	181	300	1,43	1,13	1,77	2,12	1,63	2,70
2010	319	245	405	1,78	1,39	2,21	2,88	2,21	3,65
2019	370	255	518	2,00	1,41	2,77	3,58	2,47	5,01
**Italy**									
2000	1 410	1 190	1 638	1,67	1,41	1,93	2,49	2,10	2,89
2010	1 414	1 206	1 657	1,53	1,30	1,79	2,34	2,00	2,75
2019	1 625	1 202	2 147	1,63	1,21	2,13	2,69	1,99	3,56
**Portugal**									
2000	96	68	132	0,67	0,48	0,91	0,91	0,65	1,25
2010	154	107	216	0,98	0,69	1,35	1,43	0,99	2,00
2019	190	117	295	1,12	0,71	1,72	1,78	1,10	2,77
**Spain**									
2000	655	460	907	1,17	0,83	1,61	1,60	1,13	2,22
2010	996	702	1 384	1,51	1,08	2,08	2,12	1,49	2,95
2019	1 022	645	1 551	1,43	0,92	2,14	2,22	1,40	3,37
**Western Europe**									
2000	5 831	4 535	7 334	1,04	0,82	1,29	1,46	1,14	1,84
2010	8 104	6 299	10 375	1,29	1,02	1,62	1,92	1,49	2,46
2019	9 297	6 870	12 635	1,36	1,02	1,80	2,13	1,57	2,90

Table S74- Prevalence of acute and chronic hepatitis C for Greece, Italy, Portugal and Spain, Western Europe, and globally in 2000, 2010, and 2019 (Global Burden of Disease Study 2019).

**Acute hepatitis C**									
	**Number of prevalent cases (all ages)**	**95% UL (lower)**	**95% UL (upper)**	**Rate of prevalent cases (age-standardized, per 100 000)**	**95% UL (lower)**	**95% UL (upper)**	**Rate of prevalent cases (all ages, per 100 000)**	**95% UL (lower)**	**95% UL (upper)**
**Global**									
2000	504 112	448 116	580 739	8,36	7,44	9,58	8,19	7,28	9,43
2010	570 451	506 281	655 793	8,43	7,49	9,67	8,16	7,25	9,39
2019	636 316	560 704	736 992	8,53	7,54	9,88	8,22	7,25	9,52
**Greece**									
2000	278	239	322	2,17	1,90	2,49	2,51	2,15	2,90
2010	386	323	461	2,77	2,38	3,26	3,49	2,92	4,16
2019	395	323	476	2,78	2,38	3,24	3,82	3,12	4,61
**Italy**									
2000	7 197	6 081	8 657	11,13	9,66	13,00	12,70	10,73	15,28
2010	6 327	5 290	7 620	8,90	7,65	10,53	10,49	8,77	12,63
2019	5 907	4 878	7 206	7,81	6,69	9,28	9,79	8,09	11,95
**Portugal**									
2000	556	467	652	4,81	4,17	5,60	5,27	4,43	6,19
2010	571	486	679	4,50	3,92	5,24	5,29	4,50	6,29
2019	591	501	705	4,37	3,84	5,02	5,55	4,71	6,62
**Spain**									
2000	2 240	2 033	2 457	4,90	4,55	5,29	5,49	4,98	6,02
2010	2 860	2 447	3 401	5,06	4,42	5,85	6,09	5,21	7,24
2019	2 869	2 431	3 363	4,76	4,15	5,47	6,23	5,28	7,31
**Western Europe**									
2000	22 356	19 588	25 691	4,92	4,40	5,60	5,60	4,91	6,44
2010	26 671	22 910	30 924	5,38	4,74	6,16	6,31	5,42	7,32
2019	25 856	22 076	30 151	4,72	4,13	5,45	5,93	5,06	6,91
**Cirrhosis and other chronic liver diseases due to hepatitis C**									

	Number of prevalent cases (all ages)	95% UL (lower)	95% UL (upper)	Rate of prevalent cases (age-standardized, per 100 000)	95% UL (lower)	95% UL (upper)	Rate of prevalent cases (all ages, per 100 000)	95% UL (lower)	95% UL (upper)
**Global**									
2000	84 035 986	68 856 289	102 093 292	1 415,82	1 161,59	1 716,37	1 365,24	1 118,63	1 658,59
2010	97 072 233	79 836 573	118 103 656	1 394,62	1 146,85	1 693,71	1 389,26	1 142,59	1 690,26
2019	112 371 524	91 178 709	138 096 124	1 414,72	1 146,77	1 744,72	1 452,30	1 178,41	1 784,77
**Greece**									
2000	43 666	35 686	52 760	310,86	253,68	375,62	393,68	321,74	475,68
2010	61 625	50 629	75 364	401,63	326,88	495,57	556,33	457,07	680,37
2019	63 041	51 357	77 202	402,23	328,88	494,80	609,84	496,82	746,84
**Italy**									
2000	1 335 644	1 089 673	1 610 681	1 750,37	1 432,02	2 122,27	2 357,16	1 923,07	2 842,55
2010	1 181 106	961 282	1 443 448	1 401,95	1 140,94	1 720,50	1 957,82	1 593,43	2 392,68
2019	1 076 366	870 985	1 320 494	1 214,18	981,91	1 497,73	1 784,63	1 444,10	2 189,40
**Portugal**									
2000	95 937	78 606	116 494	734,35	601,29	890,75	909,88	745,51	1 104,84
2010	99 271	81 170	120 541	683,13	553,41	840,25	919,58	751,90	1 116,60
2019	102 270	84 036	123 899	656,27	536,22	806,93	960,17	788,98	1 163,24
**Spain**									
2000	371 155	315 731	432 581	721,22	614,35	838,36	909,47	773,66	1 059,98
2010	454 046	366 214	553 771	723,17	583,64	889,13	966,55	779,58	1 178,84
2019	448 859	363 367	544 260	672,92	547,20	825,97	975,33	789,56	1 182,63
**Western Europe**									
2000	3 838 079	3 207 698	4 547 511	753,71	625,25	900,40	961,76	803,79	1 139,53
2010	4 642 248	3 842 298	5 612 937	823,41	680,12	997,83	1 098,61	909,30	1 328,33
2019	4 360 487	3 595 839	5 272 332	710,17	582,63	871,33	999,41	824,15	1 208,40
**Liver cancer due to hepatitis C**									
	**Number of prevalent cases (all ages)**	**95% UL (lower)**	**95% UL (upper)**	**Rate of prevalent cases (age-standardized, per 100 000)**	**95% UL (lower)**	**95% UL (upper)**	**Rate of prevalent cases (all ages, per 100 000)**	**95% UL (lower)**	**95% UL (upper)**

**Global**									
2000	146 793	132 050	161 792	3,01	2,71	3,31	2,38	2,15	2,63
2010	172 208	152 671	192 001	2,75	2,43	3,06	2,46	2,18	2,75
2019	211 843	183 671	241 283	2,62	2,27	2,98	2,74	2,37	3,12
**Greece**									
2000	95	66	127	0,52	0,37	0,69	0,85	0,59	1,14
2010	146	100	198	0,66	0,46	0,88	1,32	0,91	1,79
2019	175	114	259	0,71	0,46	1,06	1,70	1,10	2,50
**Italy**									
2000	5 259	4 745	5 807	5,24	4,72	5,82	9,28	8,37	10,25
2010	6 027	5 395	6 651	5,27	4,68	5,87	9,99	8,94	11,03
2019	6 621	5 207	8 379	5,20	4,05	6,65	10,98	8,63	13,89
**Portugal**									
2000	201	149	261	1,22	0,90	1,58	1,90	1,41	2,48
2010	326	241	419	1,69	1,24	2,17	3,02	2,23	3,88
2019	431	291	601	1,97	1,31	2,80	4,05	2,73	5,64
**Spain**									
2000	2 415	1 890	2 948	3,67	2,87	4,50	5,92	4,63	7,22
2010	3 599	2 837	4 378	4,64	3,60	5,75	7,66	6,04	9,32
2019	3 798	2 671	5 237	4,31	2,99	5,92	8,25	5,80	11,38
**Western Europe**									
2000	16 113	13 545	18 917	2,47	2,07	2,91	4,04	3,39	4,74
2010	22 968	19 080	26 990	3,04	2,51	3,60	5,44	4,52	6,39
2019	26 710	21 279	33 094	3,11	2,47	3,86	6,12	4,88	7,59

Table S75- Incidence of acute and chronic hepatitis B for Greece, Italy, Portugal and Spain, Western Europe, and globally in 2000, 2010, and 2019 (Global Burden of Disease Study 2019).

	Acute hepatitis B								
	**Number of incident cases (all ages)**	**95% UL (lower)**	**95% UL (upper)**	**Rate of incident cases (age-standardized, per 100 000)**	**95% UL (lower)**	**95% UL (upper)**	**Rate of incident cases (all ages, per 100 000)**	**95% UL (lower)**	**95% UL (upper)**
**Global**									
2000	92 340 214	74 786 789	110 963 916	1 471,77	1 193,33	1 753,96	1 500,15	1 214,98	1 802,71
2010	88 956 092	72 960 056	107 850 559	1 242,00	1 022,57	1 502,75	1 273,11	1 044,18	1 543,52
2019	80 023 302	63 999 705	98 290 318	1 002,43	803,64	1 227,35	1 034,23	827,14	1 270,32
**Greece**									
2000	70 093	55 365	85 971	616,24	501,39	743,12	631,95	499,17	775,10
2010	61 013	47 258	78 210	515,44	405,39	644,96	550,81	426,63	706,06
2019	49 707	37 829	61 928	448,93	354,30	555,22	480,86	365,95	599,08
**Italy**									
2000	137 221	104 210	175 056	223,01	170,46	283,51	242,17	183,91	308,94
2010	98 727	69 908	130 551	139,01	101,29	182,50	163,65	115,88	216,40
2019	68 572	43 800	96 662	86,26	59,10	116,88	113,69	72,62	160,27
**Portugal**									
2000	27 253	19 601	36 524	271,94	203,07	358,52	258,47	185,90	346,40
2010	23 122	15 820	31 795	215,65	149,86	295,10	214,18	146,54	294,53
2019	17 444	10 933	24 916	155,70	104,37	216,35	163,77	102,65	233,92
**Spain**									
2000	111 695	86 669	140 629	263,22	206,27	326,31	273,69	212,37	344,59
2010	108 203	79 878	140 841	206,20	157,67	265,77	230,34	170,04	299,82
2019	83 250	60 028	109 722	151,71	114,11	194,81	180,89	130,43	238,42
**Western Europe**									
2000	1 031 969	832 274	1 253 875	262,46	216,51	314,51	258,59	208,55	314,20
2010	950 812	756 435	1 170 614	224,64	184,78	272,38	225,01	179,01	277,03
2019	832 029	638 821	1 027 626	183,68	145,91	224,75	190,70	146,42	235,53

Cirrhosis and other chronic liver diseases due to hepatitis B									
	**Number of incident cases (all ages)**	**95% UL (lower)**	**95% UL (upper)**	**Rate of incident cases (age-standardized, per 100 000)**	**95% UL (lower)**	**95% UL (upper)**	**Rate of incident cases (all ages, per 100 000)**	**95% UL (lower)**	**95% UL (upper)**
**Global**									
2000	369 691	273 026	475 122	6,15	4,51	7,89	6,01	4,44	7,72
2010	420 313	315 689	535 854	5,78	4,35	7,33	6,02	4,52	7,67
2019	405 891	285 248	536 699	4,91	3,46	6,47	5,25	3,69	6,94
**Greece**									
2000	425	285	613	3,30	2,22	4,77	3,83	2,57	5,53
2010	197	123	297	1,49	0,94	2,24	1,78	1,11	2,69
2019	161	100	244	1,32	0,82	1,98	1,56	0,97	2,36
**Italy**									
2000	3 580	2 785	4 449	5,34	4,16	6,66	6,32	4,91	7,85
2010	2 851	2 239	3 570	3,70	2,93	4,60	4,73	3,71	5,92
2019	2 440	1 808	3 214	3,20	2,43	4,15	4,05	3,00	5,33
**Portugal**									
2000	472	296	702	3,99	2,51	5,92	4,48	2,80	6,65
2010	222	133	341	1,74	1,04	2,71	2,05	1,23	3,16
2019	145	85	227	1,16	0,69	1,81	1,36	0,80	2,13
**Spain**									
2000	1 999	1 460	2 690	4,27	3,12	5,73	4,90	3,58	6,59
2010	1 203	875	1 629	1,99	1,46	2,69	2,56	1,86	3,47
2019	864	620	1 196	1,45	1,05	1,99	1,88	1,35	2,60
**Western Europe**									
2000	16 385	11 739	22 127	3,51	2,53	4,75	4,11	2,94	5,54
2010	12 402	8 777	16 776	2,45	1,74	3,31	2,93	2,08	3,97
2019	9 321	6 695	12 502	1,90	1,36	2,53	2,14	1,53	2,87
**Liver cancer due to hepatitis B**									
	**Number of incident cases (all ages)**	**95% UL (lower)**	**95% UL (upper)**	**Rate of incident cases (age-**	**95% UL (lower)**	**95% UL (upper)**	**Rate of incident cases (all ages, per 100 000)**	**95% UL (lower)**	**95% UL (upper)**

				**standardized, per 100 000)**					
**Global**									
2000	252 386	226 383	279 409	4,75	4,24	5,28	4,10	3,68	4,54
2010	172 897	154 745	192 114	2,57	2,30	2,86	2,47	2,21	2,75
2019	218 855	186 488	254 886	2,62	2,24	3,05	2,83	2,41	3,29
**Greece**									
2000	179	135	229	1,04	0,81	1,31	1,61	1,22	2,07
2010	225	169	292	1,16	0,90	1,47	2,04	1,52	2,64
2019	269	183	381	1,31	0,92	1,84	2,61	1,77	3,69
**Italy**									
2000	858	723	1 008	0,94	0,80	1,09	1,51	1,28	1,78
2010	738	630	870	0,72	0,62	0,83	1,22	1,04	1,44
2019	849	633	1 101	0,75	0,56	0,98	1,41	1,05	1,83
**Portugal**									
2000	79	54	110	0,53	0,37	0,72	0,74	0,51	1,04
2010	115	80	162	0,68	0,48	0,95	1,07	0,74	1,50
2019	137	84	213	0,74	0,46	1,14	1,29	0,79	2,00
**Spain**									
2000	435	300	617	0,73	0,51	1,00	1,07	0,73	1,51
2010	544	379	758	0,77	0,54	1,07	1,16	0,81	1,61
2019	579	362	899	0,74	0,47	1,14	1,26	0,79	1,95
**Western Europe**									
2000	3 825	2 899	4 983	0,64	0,50	0,82	0,96	0,73	1,25
2010	4 729	3 582	6 174	0,70	0,54	0,90	1,12	0,85	1,46
2019	5 341	3 865	7 410	0,71	0,52	0,97	1,22	0,89	1,70

Table S76- Incidence of acute and chronic hepatitis C for Greece, Italy, Portugal and Spain, Western Europe, and globally in 2000, 2010, and 2019 (Global Burden of Disease Study 2019).

**Acute hepatitis C**									
	**Number of incident cases (all ages)**	**95% UL (lower)**	**95% UL (upper)**	**Rate of incident cases (age-standardized, per 100 000)**	**95% UL (lower)**	**95% UL (upper)**	**Rate of incident cases (all ages, per 100 000)**	**95% UL (lower)**	**95% UL (upper)**
**Global**									
2000	4 368 970	3 883 676	5 033 072	72,46	64,47	83,00	70,98	63,09	81,77
2010	4 943 910	4 387 765	5 683 541	73,03	64,92	83,79	70,76	62,80	81,34
2019	5 514 735	4 859 438	6 387 264	73,93	65,33	85,60	71,27	62,80	82,55
**Greece**									
2000	2 412	2 070	2 791	18,81	16,50	21,59	21,75	18,66	25,16
2010	3 346	2 803	3 996	24,01	20,60	28,27	30,21	25,30	36,07
2019	3 421	2 799	4 129	24,06	20,65	28,12	33,09	27,07	39,94
**Italy**									
2000	62 376	52 705	75 026	96,50	83,71	112,67	110,08	93,01	132,41
2010	54 835	45 850	66 041	77,17	66,29	91,24	90,90	76,00	109,47
2019	51 197	42 280	62 456	67,72	57,95	80,41	84,89	70,10	103,55
**Portugal**									
2000	4 816	4 044	5 653	41,65	36,13	48,54	45,67	38,36	53,61
2010	4 947	4 214	5 886	39,02	33,96	45,41	45,83	39,04	54,52
2019	5 120	4 345	6 114	37,87	33,31	43,55	48,07	40,79	57,40
**Spain**									
2000	19 417	17 621	21 292	42,49	39,43	45,83	47,58	43,18	52,17
2010	24 791	21 208	29 478	43,87	38,35	50,73	52,77	45,15	62,75
2019	24 863	21 064	29 144	41,29	36,00	47,40	54,02	45,77	63,33
**Western Europe**									
2000	193 748	169 763	222 657	42,66	38,14	48,52	48,55	42,54	55,79
2010	231 146	198 554	268 012	46,59	41,10	53,41	54,70	46,99	63,43
2019	224 081	191 322	261 304	40,91	35,83	47,24	51,36	43,85	59,89
**Cirrhosis and other chronic liver diseases due to hepatitis C**									

	Number of incident cases (all ages)	95% UL (lower)	95% UL (upper)	Rate of incident cases (age-standardized, per 100 000)	95% UL (lower)	95% UL (upper)	Rate of incident cases (all ages, per 100 000)	95% UL (lower)	95% UL (upper)
**Global**									
2000	370 612	279 981	474 441	6,17	4,63	7,87	6,02	4,55	7,71
2010	459 396	353 853	575 992	6,31	4,88	7,91	6,57	5,06	8,24
2019	551 689	409 296	711 000	6,67	4,98	8,56	7,13	5,29	9,19
**Greece**									
2000	449	282	672	3,52	2,23	5,25	4,05	2,55	6,06
2010	419	269	618	3,20	2,09	4,69	3,78	2,42	5,58
2019	352	216	534	2,95	1,87	4,38	3,40	2,09	5,17
**Italy**									
2000	11 533	9 168	14 153	17,21	13,69	21,00	20,35	16,18	24,98
2010	10 678	8 601	13 030	13,97	11,33	16,83	17,70	14,26	21,60
2019	9 190	6 899	11 781	12,04	9,30	15,02	15,24	11,44	19,53
**Portugal**									
2000	776	500	1 126	6,59	4,26	9,56	7,36	4,74	10,68
2010	627	392	938	4,98	3,15	7,36	5,81	3,63	8,69
2019	483	298	727	3,92	2,47	5,87	4,54	2,80	6,82
**Spain**									
2000	4 421	3 123	5 848	9,47	6,69	12,50	10,83	7,65	14,33
2010	4 800	3 289	6 411	8,00	5,56	10,67	10,22	7,00	13,65
2019	3 996	2 713	5 491	6,75	4,64	9,14	8,68	5,90	11,93
**Western Europe**									
2000	32 793	24 451	42 870	7,05	5,29	9,20	8,22	6,13	10,74
2010	31 614	23 030	41 602	6,26	4,63	8,26	7,48	5,45	9,85
2019	26 967	19 566	35 783	5,45	3,98	7,22	6,18	4,48	8,20
**Liver cancer due to hepatitis C**									
	**Number of incident cases (all ages)**	**95% UL (lower)**	**95% UL (upper)**	**Rate of incident cases (age-standardized, per 100 000)**	**95% UL (lower)**	**95% UL (upper)**	**Rate of incident cases (all ages, per 100 000)**	**95% UL (lower)**	**95% UL (upper)**

**Global**									
2000	118 950	106 392	131 576	2,48	2,22	2,74	1,93	1,73	2,14
2010	123 598	108 700	138 172	2,00	1,75	2,24	1,77	1,56	1,98
2019	152 225	131 581	174 627	1,90	1,64	2,17	1,97	1,70	2,26
**Greece**									
2000	87	61	116	0,48	0,34	0,63	0,78	0,55	1,04
2010	127	88	172	0,55	0,39	0,73	1,14	0,80	1,55
2019	158	104	229	0,59	0,38	0,86	1,53	1,01	2,22
**Italy**									
2000	3 968	3 606	4 322	3,75	3,43	4,10	7,00	6,36	7,63
2010	4 052	3 634	4 401	3,23	2,93	3,52	6,72	6,02	7,30
2019	4 438	3 568	5 463	3,14	2,50	3,92	7,36	5,92	9,06
**Portugal**									
2000	197	145	254	1,17	0,88	1,50	1,87	1,38	2,41
2010	301	223	382	1,48	1,10	1,88	2,79	2,07	3,54
2019	388	268	539	1,64	1,11	2,29	3,64	2,52	5,06
**Spain**									
2000	1 972	1 548	2 390	2,86	2,26	3,46	4,83	3,79	5,86
2010	2 599	2 079	3 096	3,11	2,47	3,75	5,53	4,42	6,59
2019	2 839	2 022	3 751	2,97	2,07	3,98	6,17	4,39	8,15
**Western Europe**									
2000	12 735	10 633	14 841	1,88	1,58	2,19	3,19	2,66	3,72
2010	16 476	13 765	19 301	2,06	1,72	2,42	3,90	3,26	4,57
2019	18 947	15 193	23 282	2,05	1,63	2,55	4,34	3,48	5,34

Table S77 - YLDs of acute and chronic hepatitis B for Greece, Italy, Portugal and Spain, Western Europe, and globally in 2000, 2010, and 2019 (Global Burden of Disease Study 2019).

Acute hepatitis B									
	**Number of YLDs (all ages)**	**95% UL (lower)**	**95% UL (upper)**	**Rate of YLDs (age-standardized, per 100 000)**	**95% UL (lower)**	**95% UL (upper)**	**Rate of YLDs (all ages, per 100 000)**	**95% UL (lower)**	**95% UL (upper)**
**Global**									
2000	155 607	96 977	244 284	2,55	1,59	4,01	2,53	1,58	3,97
2010	162 527	97 601	250 101	2,28	1,36	3,47	2,33	1,40	3,58
2019	159 319	95 874	247 151	1,96	1,19	3,02	2,06	1,24	3,19
**Greece**									
2000	141	80	225	1,13	0,65	1,79	1,27	0,73	2,03
2010	133	78	216	1,01	0,60	1,66	1,20	0,70	1,95
2019	117	70	182	0,95	0,56	1,49	1,14	0,67	1,76
**Italy**									
2000	303	176	494	0,45	0,26	0,73	0,54	0,31	0,87
2010	247	139	400	0,32	0,18	0,51	0,41	0,23	0,66
2019	182	96	307	0,21	0,12	0,36	0,30	0,16	0,51
**Portugal**									
2000	50	27	87	0,45	0,25	0,77	0,47	0,25	0,83
2010	49	25	83	0,40	0,20	0,68	0,45	0,23	0,77
2019	42	21	73	0,34	0,17	0,58	0,39	0,20	0,69
**Spain**									
2000	224	129	369	0,48	0,28	0,79	0,55	0,32	0,90
2010	245	135	401	0,42	0,24	0,69	0,52	0,29	0,85
2019	213	121	353	0,36	0,20	0,58	0,46	0,26	0,77
**Western Europe**									
2000	2 084	1 241	3 319	0,47	0,28	0,75	0,52	0,31	0,83
2010	2 044	1 202	3 219	0,42	0,25	0,66	0,48	0,28	0,76
2019	1 904	1 111	3 094	0,37	0,22	0,59	0,44	0,25	0,71

Cirrhosis and other chronic liver diseases due to hepatitis B

	Number of YLDs (all ages)	95% UL (lower)	95% UL (upper)	Rate of YLDs (age-standardized, per 100 000)	95% UL (lower)	95% UL (upper)	Rate of YLDs (all ages, per 100 000)	95% UL (lower)	95% UL (upper)
**Global**									
2000	119 573	80 664	170 860	2,15	1,45	3,08	1,94	1,31	2,78
2010	132 233	88 487	188 004	1,91	1,28	2,72	1,89	1,27	2,69
2019	135 130	87 944	193 345	1,63	1,06	2,33	1,75	1,14	2,50
**Greece**									
2000	88	51	140	0,58	0,34	0,92	0,79	0,46	1,26
2010	49	30	78	0,31	0,18	0,48	0,45	0,27	0,70
2019	46	28	72	0,29	0,17	0,45	0,45	0,27	0,70
**Italy**									
2000	717	485	1 033	0,85	0,58	1,22	1,26	0,86	1,82
2010	600	409	882	0,61	0,41	0,88	0,99	0,68	1,46
2019	532	356	777	0,51	0,34	0,73	0,88	0,59	1,29
**Portugal**									
2000	131	73	219	0,96	0,52	1,63	1,24	0,69	2,07
2010	67	37	109	0,43	0,24	0,71	0,62	0,35	1,01
2019	50	29	80	0,30	0,17	0,48	0,47	0,27	0,75
**Spain**									
2000	438	269	673	0,81	0,50	1,25	1,07	0,66	1,65
2010	254	163	382	0,38	0,24	0,57	0,54	0,35	0,81
2019	200	127	302	0,28	0,17	0,43	0,43	0,28	0,66
**Western Europe**									
2000	4 046	2 576	6 122	0,75	0,48	1,14	1,01	0,65	1,53
2010	3 139	2 027	4 734	0,51	0,33	0,77	0,74	0,48	1,12
2019	2 627	1 680	3 948	0,40	0,26	0,60	0,60	0,38	0,90

Liver cancer due to hepatitis B

	Number of YLDs (all ages)	95% UL (lower)	95% UL (upper)	Rate of YLDs (age-standardized, per 100 000)	95% UL (lower)	95% UL (upper)	Rate of YLDs (all ages, per 100 000)	95% UL (lower)	95% UL (upper)
**Global**									

2000	56 235	39 780	73 884	1,05	0,74	1,38	0,91	0,65	1,20
2010	40 624	28 588	53 937	0,60	0,42	0,80	0,58	0,41	0,77
2019	52 062	36 336	71 490	0,62	0,44	0,86	0,67	0,47	0,92
**Greece**									
2000	43	28	61	0,25	0,16	0,35	0,39	0,25	0,55
2010	55	35	79	0,29	0,19	0,41	0,50	0,32	0,71
2019	65	39	101	0,33	0,20	0,51	0,63	0,37	0,98
**Italy**									
2000	218	154	297	0,24	0,17	0,33	0,38	0,27	0,52
2010	197	139	268	0,20	0,14	0,27	0,33	0,23	0,44
2019	227	148	343	0,21	0,14	0,32	0,38	0,24	0,57
**Portugal**									
2000	18	11	28	0,12	0,08	0,19	0,17	0,11	0,27
2010	28	17	42	0,17	0,10	0,26	0,26	0,15	0,39
2019	33	19	56	0,18	0,10	0,31	0,31	0,17	0,53
**Spain**									
2000	108	67	165	0,18	0,11	0,28	0,26	0,16	0,40
2010	143	88	216	0,21	0,13	0,31	0,30	0,19	0,46
2019	151	86	257	0,20	0,12	0,33	0,33	0,19	0,56
**Western Europe**									
2000	952	611	1 366	0,16	0,11	0,23	0,24	0,15	0,34
2010	1 221	800	1 751	0,18	0,12	0,26	0,29	0,19	0,41
2019	1 388	882	2 062	0,19	0,12	0,28	0,32	0,20	0,47

Table S78- YLDs of acute and chronic hepatitis C for Greece, Italy, Portugal and Spain, Western Europe, and globally in 2000, 2010, and 2019 (Global Burden of Disease Study 2019).

Acute hepatitis C									
	**Number of YLDs (all ages)**	**95% UL (lower)**	**95% UL (upper)**	**Rate of YLDs (age-standardized, per 100 000)**	**95% UL (lower)**	**95% UL (upper)**	**Rate of YLDs (all ages, per 100 000)**	**95% UL (lower)**	**95% UL (upper)**
**Global**									
2000	7 061	3 403	13 846	0,12	0,06	0,23	0,11	0,06	0,22
2010	7 985	3 875	15 602	0,12	0,06	0,23	0,11	0,06	0,22
2019	8 915	4 257	17 539	0,12	0,06	0,24	0,12	0,06	0,23
**Greece**									
2000	4	2	8	0,03	0,01	0,06	0,04	0,02	0,07
2010	5	3	11	0,04	0,02	0,08	0,05	0,02	0,10
2019	6	3	11	0,04	0,02	0,08	0,05	0,03	0,11
**Italy**									
2000	101	49	203	0,16	0,08	0,31	0,18	0,09	0,36
2010	89	43	179	0,13	0,06	0,25	0,15	0,07	0,30
2019	83	40	167	0,11	0,05	0,22	0,14	0,07	0,28
**Portugal**									
2000	8	4	16	0,07	0,03	0,13	0,07	0,04	0,15
2010	8	4	16	0,06	0,03	0,12	0,07	0,04	0,15
2019	8	4	17	0,06	0,03	0,12	0,08	0,04	0,16
**Spain**									
2000	31	15	60	0,07	0,03	0,13	0,08	0,04	0,15
2010	40	19	79	0,07	0,03	0,14	0,09	0,04	0,17
2019	40	20	80	0,07	0,03	0,13	0,09	0,04	0,17
**Western Europe**									
2000	314	155	617	0,07	0,03	0,14	0,08	0,04	0,15
2010	375	183	746	0,08	0,04	0,15	0,09	0,04	0,18
2019	364	178	738	0,07	0,03	0,13	0,08	0,04	0,17
Cirrhosis and other chronic liver diseases due to hepatitis C									

	Number of YLDs (all ages)	95% UL (lower)	95% UL (upper)	Rate of YLDs (age-standardized, per 100 000)	95% UL (lower)	95% UL (upper)	Rate of YLDs (all ages, per 100 000)	95% UL (lower)	95% UL (upper)
**Global**									
2000	103 673	69 702	150 306	1,88	1,27	2,71	1,68	1,13	2,44
2010	124 703	84 414	180 000	1,81	1,23	2,61	1,78	1,21	2,58
2019	157 125	105 538	227 914	1,90	1,27	2,75	2,03	1,36	2,95
**Greece**									
2000	91	52	145	0,61	0,36	0,97	0,82	0,47	1,31
2010	96	56	153	0,61	0,35	0,99	0,86	0,51	1,38
2019	93	55	146	0,60	0,35	0,95	0,90	0,53	1,41
**Italy**									
2000	2 351	1 604	3 385	2,77	1,88	3,99	4,15	2,83	5,97
2010	2 352	1 601	3 396	2,37	1,62	3,46	3,90	2,65	5,63
2019	2 081	1 391	3 010	1,97	1,31	2,88	3,45	2,31	4,99
**Portugal**									
2000	197	114	319	1,46	0,84	2,36	1,87	1,08	3,03
2010	171	100	276	1,13	0,65	1,84	1,58	0,93	2,56
2019	149	87	241	0,91	0,54	1,50	1,40	0,81	2,27
**Spain**									
2000	958	605	1 438	1,76	1,11	2,67	2,35	1,48	3,52
2010	962	596	1 442	1,45	0,91	2,17	2,05	1,27	3,07
2019	878	538	1 336	1,24	0,76	1,87	1,91	1,17	2,90
**Western Europe**									
2000	7 490	4 858	10 994	1,39	0,90	2,04	1,88	1,22	2,75
2010	7 329	4 744	10 976	1,19	0,77	1,79	1,73	1,12	2,60
2019	6 860	4 421	10 277	1,04	0,67	1,56	1,57	1,01	2,36

Liver cancer due to hepatitis C

	Number of YLDs (all ages)	95% UL (lower)	95% UL (upper)	Rate of YLDs (age-standardized, per 100 000)	95% UL (lower)	95% UL (upper)	Rate of YLDs (all ages, per 100 000)	95% UL (lower)	95% UL (upper)
**Global**									
2000	26 937	18 698	35 463	0,56	0,39	0,73	0,44	0,30	0,58

2010	29 028	20 007	38 379	0,47	0,32	0,62	0,42	0,29	0,55
2019	35 910	24 498	48 391	0,45	0,31	0,60	0,46	0,32	0,63
**Greece**									
2000	20	12	30	0,11	0,07	0,16	0,18	0,11	0,27
2010	30	18	45	0,13	0,08	0,19	0,27	0,16	0,41
2019	37	21	58	0,14	0,08	0,22	0,35	0,20	0,56
**Italy**									
2000	931	656	1 237	0,89	0,63	1,18	1,64	1,16	2,18
2010	988	707	1 319	0,81	0,57	1,09	1,64	1,17	2,19
2019	1 084	714	1 549	0,79	0,52	1,14	1,80	1,18	2,57
**Portugal**									
2000	44	28	64	0,26	0,17	0,38	0,42	0,26	0,61
2010	69	43	98	0,34	0,21	0,49	0,64	0,39	0,90
2019	89	51	135	0,38	0,22	0,59	0,84	0,48	1,26
**Spain**									
2000	462	295	638	0,68	0,44	0,94	1,13	0,72	1,56
2010	624	408	863	0,76	0,50	1,05	1,33	0,87	1,84
2019	674	411	997	0,72	0,43	1,10	1,47	0,89	2,17
**Western Europe**									
2000	3 001	2 017	4 049	0,45	0,30	0,61	0,75	0,51	1,01
2010	3 982	2 704	5 415	0,51	0,34	0,69	0,94	0,64	1,28
2019	4 599	3 012	6 460	0,51	0,33	0,72	1,05	0,69	1,48

Table S79- YLLs of acute and chronic hepatitis B for Greece, Italy, Portugal and Spain, Western Europe, and globally in 2000, 2010, and 2019 (Global Burden of Disease Study 2019).

**Acute hepatitis B**									
	**Number of YLLs (all ages)**	**95% UL (lower)**	**95% UL (upper)**	**Rate of YLLs (age-standardized, per 100 000)**	**95% UL (lower)**	**95% UL (upper)**	**Rate of YLLs (all ages, per 100 000)**	**95% UL (lower)**	**95% UL (upper)**
**Global**									
2000	2 100 475	1 312 225	3 167 498	34,86	21,97	51,75	34,12	21,32	51,46
2010	1 747 538	1 150 674	2 566 405	25,32	16,64	37,43	25,01	16,47	36,73
2019	1 455 059	1 030 984	2 039 206	18,98	13,36	26,74	18,81	13,32	26,35
**Greece**									
2000	410	343	465	2,70	2,32	3,01	3,70	3,09	4,19
2010	1 608	1 307	1 824	9,03	7,71	10,00	14,51	11,80	16,47
2019	659	551	758	3,78	3,28	4,30	6,37	5,33	7,33
**Italy**									
2000	995	694	1 169	1,52	1,03	1,81	1,76	1,22	2,06
2010	898	567	1 416	1,17	0,70	1,89	1,49	0,94	2,35
2019	865	612	1 139	1,05	0,73	1,41	1,43	1,01	1,89
**Portugal**									
2000	528	356	639	4,61	3,06	5,62	5,01	3,38	6,06
2010	93	79	119	0,73	0,61	0,96	0,86	0,73	1,10
2019	77	64	95	0,59	0,48	0,72	0,73	0,60	0,89
**Spain**									
2000	213	163	321	0,53	0,38	0,78	0,52	0,40	0,79
2010	227	178	305	0,45	0,35	0,58	0,48	0,38	0,65
2019	224	156	276	0,42	0,30	0,53	0,49	0,34	0,60
**Western Europe**									
2000	4 773	3 760	5 446	1,01	0,79	1,15	1,20	0,94	1,36
2010	4 728	3 939	5 616	0,85	0,72	1,03	1,12	0,93	1,33
2019	3 670	3 115	4 239	0,64	0,55	0,74	0,84	0,71	0,97
**Cirrhosis and other chronic liver diseases due to hepatitis B**									

	Number of YLLs (all ages)	95% UL (lower)	95% UL (upper)	Rate of YLLs (age-standardized, per 100 000)	95% UL (lower)	95% UL (upper)	Rate of YLLs (all ages, per 100 000)	95% UL (lower)	95% UL (upper)
**Global**									
2000	11 121 447	9 822 768	12 576 800	199,56	176,80	225,95	180,68	159,58	204,32
2010	11 608 979	10 109 158	13 175 684	166,71	145,20	189,60	166,14	144,68	188,57
2019	10 651 625	8 848 310	12 574 969	128,22	106,60	151,52	137,66	114,36	162,52
**Greece**									
2000	3 816	2 688	5 245	23,96	17,08	32,10	34,41	24,24	47,29
2010	2 131	1 481	2 973	12,70	8,65	17,79	19,24	13,37	26,84
2019	2 040	1 394	2 829	11,67	8,02	16,28	19,74	13,49	27,37
**Italy**									
2000	37 494	33 716	41 336	43,12	39,15	47,31	66,17	59,50	72,95
2010	23 992	20 832	27 064	23,85	20,81	26,97	39,77	34,53	44,86
2019	21 896	19 043	25 132	19,97	17,52	22,77	36,30	31,57	41,67
**Portugal**									
2000	9 602	6 748	13 036	70,20	49,33	96,61	91,06	64,00	123,63
2010	4 838	3 221	6 858	31,16	20,72	44,71	44,81	29,84	63,53
2019	3 487	2 353	4 940	20,57	13,76	29,93	32,74	22,10	46,38
**Spain**									
2000	25 838	20 382	32 615	46,28	36,71	58,61	63,31	49,94	79,92
2010	13 667	10 504	17 667	19,69	15,26	25,64	29,09	22,36	37,61
2019	10 804	8 173	14 236	13,87	10,60	18,25	23,48	17,76	30,93
**Western Europe**									
2000	244 759	188 241	314 202	44,86	34,02	58,65	61,33	47,17	78,73
2010	170 775	126 963	224 702	27,36	20,53	36,19	40,41	30,05	53,18
2019	139 377	105 544	182 040	20,57	15,61	27,09	31,94	24,19	41,72
**Liver cancer due to hepatitis B**									
	Number of YLLs (all ages)	95% UL (lower)	95% UL (upper)	Rate of YLLs (age-standardized, per 100 000)	95% UL (lower)	95% UL (upper)	Rate of YLLs (all ages, per 100 000)	95% UL (lower)	95% UL (upper)
**Global**									
2000	7 844 140	7 067 258	8 647 002	142,69	128,39	157,68	127,43	114,81	140,48

2010	4 845 714	4 383 553	5 383 778	70,33	63,59	78,23	69,35	62,74	77,05
2019	5 745 036	4 851 853	6 711 876	68,53	57,99	79,87	74,25	62,71	86,75
**Greece**									
2000	3 758	2 893	4 795	22,60	17,95	28,21	33,89	26,08	43,23
2010	4 442	3 407	5 632	24,60	19,21	30,66	40,11	30,76	50,84
2019	5 242	3 930	6 818	28,06	21,78	35,44	50,71	38,01	65,95
**Italy**									
2000	17 686	14 946	20 811	20,15	17,13	23,41	31,21	26,38	36,73
2010	13 487	11 549	15 741	13,77	11,82	15,98	22,36	19,14	26,09
2019	15 238	12 601	18 487	14,23	11,82	16,96	25,26	20,89	30,65
**Portugal**									
2000	2 103	1 488	2 892	14,86	10,71	20,23	19,94	14,12	27,43
2010	2 891	1 985	4 077	18,20	12,68	25,52	26,78	18,39	37,77
2019	3 248	2 195	4 753	18,73	12,99	26,83	30,50	20,61	44,63
**Spain**									
2000	9 576	6 754	13 162	16,91	12,07	23,02	23,46	16,55	32,25
2010	10 983	7 690	15 342	16,27	11,43	22,49	23,38	16,37	32,66
2019	11 648	7 845	16 701	15,60	10,79	22,19	25,31	17,05	36,29
**Western Europe**									
2000	82 893	63 323	107 047	14,60	11,29	18,51	20,77	15,87	26,82
2010	93 898	71 611	122 192	14,66	11,33	18,75	22,22	16,95	28,92
2019	101 908	75 227	135 632	14,42	10,96	18,72	23,36	17,24	31,09

Table S80- YLLs of acute and chronic hepatitis C for Greece, Italy, Portugal and Spain, Western Europe, and globally in 2000, 2010, and 2019 (Global Burden of Disease Study 2019).

Acute hepatitis C									
	**Number of YLLs (all ages)**	**95% UL (lower)**	**95% UL (upper)**	**Rate of YLLs (age-standardized, per 100 000)**	**95% UL (lower)**	**95% UL (upper)**	**Rate of YLLs (all ages, per 100 000)**	**95% UL (lower)**	**95% UL (upper)**
**Global**									
2000	465 251	235 335	764 659	7,73	3,98	12,54	7,56	3,82	12,42
2010	351 095	179 051	573 368	5,12	2,59	8,37	5,02	2,56	8,21
2019	244 437	136 210	367 137	3,18	1,72	4,76	3,16	1,76	4,74
**Greece**									
2000	27	11	70	0,21	0,11	0,49	0,24	0,10	0,63
2010	105	49	274	0,64	0,35	1,42	0,95	0,44	2,48
2019	44	26	101	0,27	0,17	0,54	0,43	0,25	0,97
**Italy**									
2000	355	151	516	0,44	0,23	0,64	0,63	0,27	0,91
2010	148	91	425	0,17	0,09	0,52	0,25	0,15	0,70
2019	123	75	327	0,13	0,07	0,36	0,20	0,12	0,54
**Portugal**									
2000	109	62	237	0,92	0,51	2,03	1,03	0,59	2,25
2010	32	12	43	0,24	0,08	0,32	0,30	0,11	0,40
2019	18	7	26	0,13	0,05	0,19	0,17	0,07	0,24
**Spain**									
2000	271	169	332	0,56	0,36	0,76	0,66	0,41	0,81
2010	185	114	243	0,31	0,20	0,43	0,39	0,24	0,52
2019	135	87	193	0,22	0,14	0,32	0,29	0,19	0,42
**Western Europe**									
2000	1 617	883	2 229	0,32	0,19	0,46	0,41	0,22	0,56
2010	914	601	1 624	0,17	0,11	0,29	0,22	0,14	0,38
2019	656	433	1 239	0,11	0,07	0,21	0,15	0,10	0,28
**Cirrhosis and other chronic liver diseases due to hepatitis C**									

	Number of YLLs (all ages)	95% UL (lower)	95% UL (upper)	Rate of YLLs (age-standardized, per 100 000)	95% UL (lower)	95% UL (upper)	Rate of YLLs (all ages, per 100 000)	95% UL (lower)	95% UL (upper)
**Global**									
2000	9 431 274	8 216 794	10 825 758	170,45	148,51	195,50	153,22	133,49	175,87
2010	10 880 646	9 481 714	12 439 550	157,43	137,25	180,10	155,72	135,70	178,03
2019	12 000 036	10 183 803	13 990 365	144,35	122,69	167,81	155,09	131,62	180,81
**Greece**									
2000	4 950	3 592	6 733	30,90	22,75	41,66	44,63	32,38	60,71
2010	5 020	3 661	6 816	29,69	21,34	40,70	45,32	33,05	61,54
2019	4 969	3 621	6 769	28,12	20,60	38,35	48,07	35,03	65,48
**Italy**									
2000	109 248	98 864	121 305	123,94	113,08	136,30	192,80	174,48	214,08
2010	87 169	77 059	98 666	84,80	75,12	95,66	144,49	127,73	163,55
2019	80 782	71 180	91 944	71,47	63,33	80,86	133,94	118,02	152,44
**Portugal**									
2000	14 041	10 027	19 111	102,40	73,76	138,39	133,16	95,09	181,25
2010	11 239	8 140	15 372	72,11	52,12	98,89	104,11	75,41	142,40
2019	9 417	6 640	13 212	55,19	39,59	77,56	88,41	62,34	124,05
**Spain**									
2000	55 952	43 383	71 556	99,51	76,97	126,74	137,10	106,31	175,34
2010	51 311	39 551	65 594	73,70	55,92	95,73	109,23	84,19	139,63
2019	45 602	34 906	59 196	58,20	43,90	75,58	99,09	75,85	128,63
**Western Europe**									
2000	422 200	337 413	534 159	76,21	60,20	96,41	105,80	84,55	133,85
2010	365 527	288 116	470 691	57,36	44,75	73,62	86,50	68,18	111,39
2019	339 528	265 800	434 360	48,56	37,57	62,24	77,82	60,92	99,55

Liver cancer due to hepatitis C

	Number of YLLs (all ages)	95% UL (lower)	95% UL (upper)	Rate of YLLs (age-standardized, per 100 000)	95% UL (lower)	95% UL (upper)	Rate of YLLs (all ages, per 100 000)	95% UL (lower)	95% UL (upper)
**Global**									
2000	2 562 046	2 255 002	2 862 120	51,25	45,30	57,26	41,62	36,63	46,50

2010	2 373 869	2 049 197	2 676 192	37,05	32,03	41,81	33,97	29,33	38,30
2019	2 842 114	2 406 557	3 279 440	34,54	29,36	39,77	36,73	31,10	42,38
**Greece**									
2000	1 456	1 017	1 958	8,04	5,71	10,70	13,13	9,17	17,65
2010	1 987	1 363	2 714	9,11	6,34	12,37	17,94	12,31	24,50
2019	2 388	1 649	3 304	9,97	6,84	13,98	23,10	15,95	31,96
**Italy**									
2000	71 665	65 181	78 199	70,42	63,85	77,10	126,47	115,03	138,01
2010	65 278	59 294	70 763	55,19	50,07	60,04	108,21	98,29	117,30
2019	70 053	61 737	77 500	53,11	46,67	59,39	116,15	102,36	128,50
**Portugal**									
2000	4 183	3 073	5 471	25,59	18,84	33,15	39,67	29,14	51,89
2010	6 067	4 460	7 762	31,88	23,39	41,01	56,20	41,31	71,90
2019	7 447	5 372	9 688	34,44	24,40	45,89	69,91	50,43	90,96
**Spain**									
2000	36 323	28 509	44 164	55,12	43,53	67,08	89,01	69,86	108,22
2010	44 225	34 897	53 017	56,30	43,42	68,22	94,14	74,29	112,86
2019	48 228	37 172	59 581	54,25	41,46	67,30	104,80	80,77	129,46
**Western Europe**									
2000	229 851	191 789	270 102	35,48	29,58	41,86	57,60	48,06	67,68
2010	273 026	225 747	322 487	36,29	29,70	43,18	64,61	53,42	76,32
2019	304 241	247 745	365 068	35,50	28,63	42,95	69,73	56,78	83,67

Table S81- DALYs of acute and chronic hepatitis B for Greece, Italy, Portugal and Spain, Western Europe, and globally in 2000, 2010, and 2019 (Global Burden of Disease Study 2019).

**Acute hepatitis B**									
	**Number of DALYs (all ages)**	**95% UL (lower)**	**95% UL (upper)**	**Rate of DALYs (age-standardized, per 100 000)**	**95% UL (lower)**	**95% UL (upper)**	**Rate of DALYs (all ages, per 100 000)**	**95% UL (lower)**	**95% UL (upper)**
**Global**									
2000	2 256 082	1 460 332	3 307 259	37,42	24,18	54,17	36,65	23,72	53,73
2010	1 910 064	1 297 857	2 732 958	27,60	18,67	39,62	27,34	18,57	39,11
2019	1 614 378	1 172 653	2 206 578	20,94	15,06	28,84	20,86	15,16	28,52
**Greece**									
2000	551	462	652	3,83	3,25	4,54	4,97	4,17	5,88
2010	1 740	1 428	1 984	10,04	8,56	11,23	15,71	12,89	17,91
2019	776	651	887	4,73	4,09	5,43	7,51	6,30	8,58
**Italy**									
2000	1 298	976	1 568	1,97	1,46	2,41	2,29	1,72	2,77
2010	1 144	794	1 714	1,49	1,00	2,27	1,90	1,32	2,84
2019	1 047	774	1 355	1,26	0,93	1,65	1,74	1,28	2,25
**Portugal**									
2000	578	402	697	5,05	3,50	6,13	5,48	3,81	6,61
2010	142	111	185	1,14	0,89	1,50	1,31	1,03	1,71
2019	119	93	155	0,93	0,72	1,19	1,12	0,88	1,46
**Spain**									
2000	436	319	593	1,02	0,77	1,36	1,07	0,78	1,45
2010	473	350	640	0,87	0,66	1,16	1,01	0,75	1,36
2019	437	319	578	0,78	0,58	1,03	0,95	0,69	1,26
**Western Europe**									
2000	6 857	5 571	8 359	1,48	1,20	1,80	1,72	1,40	2,09
2010	6 772	5 712	8 228	1,28	1,06	1,58	1,60	1,35	1,95
2019	5 574	4 679	6 787	1,02	0,85	1,25	1,28	1,07	1,56
**Cirrhosis and other chronic liver diseases due to hepatitis B**									

	Number of DALYs (all ages)	95% UL (lower)	95% UL (upper)	Rate of DALYs (age-standardized, per 100 000)	95% UL (lower)	95% UL (upper)	Rate of DALYs (all ages, per 100 000)	95% UL (lower)	95% UL (upper)
**Global**									
2000	11 241 020	9 964 071	12 686 698	201,72	178,96	227,79	182,62	161,88	206,11
2010	11 741 212	10 229 886	13 322 345	168,62	146,87	191,28	168,04	146,41	190,66
2019	10 786 755	8 991 170	12 715 662	129,85	108,27	152,95	139,41	116,20	164,34
**Greece**									
2000	3 904	2 789	5 333	24,54	17,64	32,79	35,20	25,14	48,09
2010	2 180	1 523	3 008	13,00	8,97	18,10	19,68	13,75	27,16
2019	2 086	1 436	2 881	11,96	8,30	16,54	20,18	13,89	27,87
**Italy**									
2000	38 211	34 444	41 971	43,96	39,97	48,20	67,44	60,79	74,07
2010	24 591	21 413	27 626	24,46	21,45	27,59	40,76	35,49	45,79
2019	22 427	19 548	25 687	20,48	17,97	23,30	37,19	32,41	42,59
**Portugal**									
2000	9 733	6 874	13 171	71,16	50,29	97,43	92,31	65,19	124,91
2010	4 905	3 299	6 925	31,60	21,09	45,12	45,43	30,56	64,15
2019	3 536	2 403	4 993	20,86	14,06	30,10	33,20	22,56	46,88
**Spain**									
2000	26 276	20 766	33 129	47,08	37,54	59,43	64,39	50,88	81,18
2010	13 921	10 746	17 942	20,06	15,60	26,02	29,63	22,87	38,19
2019	11 004	8 435	14 444	14,15	10,84	18,54	23,91	18,33	31,38
**Western Europe**									
2000	248 805	192 300	318 457	45,61	34,73	59,43	62,35	48,19	79,80
2010	173 914	130 584	227 545	27,87	20,96	36,77	41,16	30,90	53,85
2019	142 005	108 656	184 709	20,97	16,00	27,47	32,55	24,90	42,33
**Liver cancer due to hepatitis B**									
	**Number of DALYs (all ages)**	**95% UL (lower)**	**95% UL (upper)**	**Rate of DALYs (age-standardized, per 100 000)**	**95% UL (lower)**	**95% UL (upper)**	**Rate of DALYs (all ages, per 100 000)**	**95% UL (lower)**	**95% UL (upper)**
**Global**									
2000	7 900 375	7 129 778	8 703 690	143,74	129,39	158,71	128,35	115,83	141,40

2010	4 886 338	4 419 819	5 436 455	70,93	64,12	78,90	69,93	63,25	77,80
2019	5 797 099	4 911 756	6 771 065	69,15	58,71	80,61	74,92	63,48	87,51
**Greece**									
2000	3 801	2 923	4 848	22,85	18,15	28,48	34,27	26,35	43,71
2010	4 498	3 451	5 701	24,89	19,42	31,05	40,60	31,15	51,47
2019	5 307	3 972	6 906	28,39	21,97	35,89	51,34	38,43	66,81
**Italy**									
2000	17 904	15 124	21 079	20,39	17,34	23,70	31,60	26,69	37,20
2010	13 685	11 714	15 963	13,97	12,01	16,21	22,68	19,42	26,46
2019	15 465	12 765	18 745	14,44	12,00	17,28	25,64	21,17	31,08
**Portugal**									
2000	2 121	1 501	2 914	14,99	10,81	20,40	20,12	14,24	27,64
2010	2 918	2 003	4 119	18,37	12,82	25,73	27,03	18,55	38,15
2019	3 281	2 217	4 809	18,92	13,09	27,06	30,81	20,81	45,15
**Spain**									
2000	9 684	6 841	13 291	17,10	12,20	23,26	23,73	16,76	32,57
2010	11 126	7 819	15 557	16,48	11,58	22,75	23,68	16,65	33,12
2019	11 798	7 930	16 934	15,80	10,92	22,51	25,64	17,23	36,80
**Western Europe**									
2000	83 845	63 991	108 189	14,77	11,43	18,69	21,01	16,03	27,11
2010	95 120	72 511	123 732	14,84	11,47	18,99	22,51	17,16	29,28
2019	103 296	76 204	137 069	14,60	11,09	18,96	23,67	17,47	31,42

Table S82- DALYs of acute and chronic hepatitis C for Greece, Italy, Portugal and Spain, Western Europe, and globally in 2000, 2010, and 2019 (Global Burden of Disease Study 2019).

Acute hepatitis C									
	**Number of DALYs (all ages)**	**95% UL (lower)**	**95% UL (upper)**	**Rate of DALYs (age-standardized, per 100 000)**	**95% UL (lower)**	**95% UL (upper)**	**Rate of DALYs (all ages, per 100 000)**	**95% UL (lower)**	**95% UL (upper)**
**Global**									
2000	472 311	243 903	771 423	7,85	4,12	12,64	7,67	3,96	12,53
2010	359 080	187 299	580 694	5,24	2,71	8,47	5,14	2,68	8,31
2019	253 352	145 071	375 451	3,30	1,85	4,89	3,27	1,87	4,85
**Greece**									
2000	30	14	73	0,24	0,13	0,51	0,27	0,13	0,66
2010	111	54	278	0,68	0,39	1,46	1,00	0,49	2,51
2019	50	30	106	0,31	0,20	0,57	0,48	0,29	1,03
**Italy**									
2000	456	242	626	0,60	0,36	0,83	0,81	0,43	1,10
2010	238	147	525	0,29	0,17	0,67	0,39	0,24	0,87
2019	206	127	413	0,24	0,14	0,47	0,34	0,21	0,68
**Portugal**									
2000	117	68	245	0,99	0,58	2,10	1,11	0,65	2,32
2010	40	19	54	0,30	0,14	0,41	0,37	0,17	0,50
2019	26	14	38	0,19	0,10	0,28	0,25	0,13	0,35
**Spain**									
2000	303	196	370	0,63	0,43	0,83	0,74	0,48	0,91
2010	226	150	289	0,38	0,27	0,50	0,48	0,32	0,61
2019	176	121	243	0,28	0,20	0,41	0,38	0,26	0,53
**Western Europe**									
2000	1 931	1 145	2 607	0,39	0,24	0,54	0,48	0,29	0,65
2010	1 289	918	2 053	0,24	0,17	0,38	0,31	0,22	0,49
2019	1 020	709	1 652	0,18	0,12	0,28	0,23	0,16	0,38
**Cirrhosis and other chronic liver diseases due to hepatitis C**									

	Number of DALYs (all ages)	95% UL (lower)	95% UL (upper)	Rate of DALYs (age-standardized, per 100 000)	95% UL (lower)	95% UL (upper)	Rate of DALYs (all ages, per 100 000)	95% UL (lower)	95% UL (upper)
**Global**									
2000	9 534 947	8 329 403	10 929 338	172,33	150,36	197,29	154,90	135,32	177,56
2010	11 005 349	9 594 732	12 578 328	159,24	138,91	182,14	157,50	137,32	180,02
2019	12 157 161	10 325 549	14 166 608	146,25	124,43	169,84	157,12	133,45	183,09
**Greece**									
2000	5 041	3 679	6 828	31,51	23,26	42,46	45,45	33,17	61,56
2010	5 116	3 785	6 950	30,30	21,87	41,21	46,19	34,17	62,74
2019	5 062	3 714	6 869	28,72	21,09	39,15	48,97	35,93	66,45
**Italy**									
2000	111 599	101 163	123 457	126,72	115,90	138,87	196,95	178,53	217,88
2010	89 522	79 114	100 977	87,17	77,49	98,31	148,39	131,14	167,38
2019	82 863	72 955	93 802	73,44	65,13	82,77	137,39	120,96	155,53
**Portugal**									
2000	14 238	10 215	19 329	103,86	75,13	140,71	135,03	96,88	183,32
2010	11 410	8 283	15 572	73,24	52,94	99,82	105,69	76,73	144,24
2019	9 565	6 765	13 396	56,10	40,46	78,53	89,80	63,52	125,77
**Spain**									
2000	56 911	44 296	72 537	101,27	78,26	128,56	139,45	108,54	177,74
2010	52 273	40 483	66 742	75,15	57,12	97,24	111,28	86,18	142,08
2019	46 480	35 863	59 763	59,44	45,25	76,83	101,00	77,93	129,86
**Western Europe**									
2000	429 691	345 187	540 599	77,60	61,74	97,77	107,67	86,50	135,46
2010	372 856	294 629	477 838	58,55	45,83	74,98	88,24	69,73	113,08
2019	346 388	272 369	440 861	49,60	38,73	63,24	79,39	62,43	101,04

Liver cancer due to hepatitis C

	Number of DALYs (all ages)	95% UL (lower)	95% UL (upper)	Rate of DALYs (age-standardized, per 100 000)	95% UL (lower)	95% UL (upper)	Rate of DALYs (all ages, per 100 000)	95% UL (lower)	95% UL (upper)
**Global**									
2000	2 588 983	2 284 250	2 893 569	51,81	45,81	57,85	42,06	37,11	47,01

2010	2 402 897	2 071 103	2 712 253	37,52	32,41	42,30	34,39	29,64	38,82
2019	2 878 024	2 439 911	3 323 494	34,99	29,71	40,28	37,20	31,53	42,95
**Greece**									
2000	1 476	1 029	1 985	8,15	5,78	10,87	13,31	9,28	17,90
2010	2 017	1 383	2 757	9,24	6,43	12,56	18,21	12,49	24,89
2019	2 425	1 671	3 367	10,11	6,93	14,17	23,46	16,16	32,57
**Italy**									
2000	72 596	66 035	79 371	71,32	64,63	78,17	128,12	116,54	140,08
2010	66 265	60 126	71 953	56,00	50,89	61,00	109,84	99,67	119,27
2019	71 136	62 707	78 795	53,90	47,26	60,26	117,95	103,97	130,64
**Portugal**									
2000	4 227	3 104	5 531	25,85	19,05	33,43	40,09	29,44	52,45
2010	6 136	4 514	7 857	32,22	23,59	41,45	56,84	41,81	72,78
2019	7 536	5 439	9 828	34,82	24,71	46,34	70,75	51,07	92,27
**Spain**									
2000	36 785	28 867	44 731	55,80	43,98	67,74	90,14	70,74	109,61
2010	44 849	35 375	53 832	57,06	43,80	69,18	95,47	75,30	114,59
2019	48 902	37 655	60 373	54,97	41,91	68,27	106,26	81,82	131,18
**Western Europe**									
2000	232 852	194 440	274 077	35,93	29,93	42,36	58,35	48,72	68,68
2010	277 008	229 307	326 956	36,80	30,11	43,73	65,56	54,27	77,38
2019	308 839	250 962	370 796	36,01	28,98	43,60	70,78	57,52	84,99
